# Bis(phosphazenyl)phosphines: From Superbases to Superhydrides †

**DOI:** 10.3390/molecules31091535

**Published:** 2026-05-05

**Authors:** Mario Damjanović, Borislav Kovačević

**Affiliations:** 1 Division of Physical Chemistry, Ruđer Bošković Institute, 10000 Zagreb, Croatia; 2 Faculty of Science, University of Zagreb, Horvatovac 102A, 10000 Zagreb, Croatia; 3 Faculty of Health Sciences, Libertas University, Trg John F. Kennedy 6B, 10000 Zagreb, Croatia

**Keywords:** DFT study, hydricity, basicity, phosphazenyl phosphines, proton sponges

## Abstract

By incorporating phosphazenes as substituents on phosphines and employing rigid cage-like scaffolds, pentacyclo [5.4.0.0^2,6^.0^3,10^.0^5.9^]undecane, pentacyclo [6.4.0.0^2,7^.0^3,11^.0^6,10^] dodecane, and seco-dodecahedradiene, we designed and computationally investigated proton-sponge-like bisphosphines that act as exceptionally strong hydride donors in their monoprotonated forms. These systems exhibit hydricities that significantly surpass that of the paradigmatic superhydride LiEt_3_BH, one of the strongest commercially available hydride donors widely used in organic reductions. The remarkable hydride-donating ability of these systems originates from strongly electron-donating phosphazenyl substituents at phosphorus, which stabilize the dication formed upon hydride release, as well as from a stabilizing intramolecular dative P→P interaction within the bisphosphine framework. In addition, the neutral forms of the corresponding bisphosphines are shown to exhibit superbasic properties.

## 1. Introduction

**Scheme 1:**
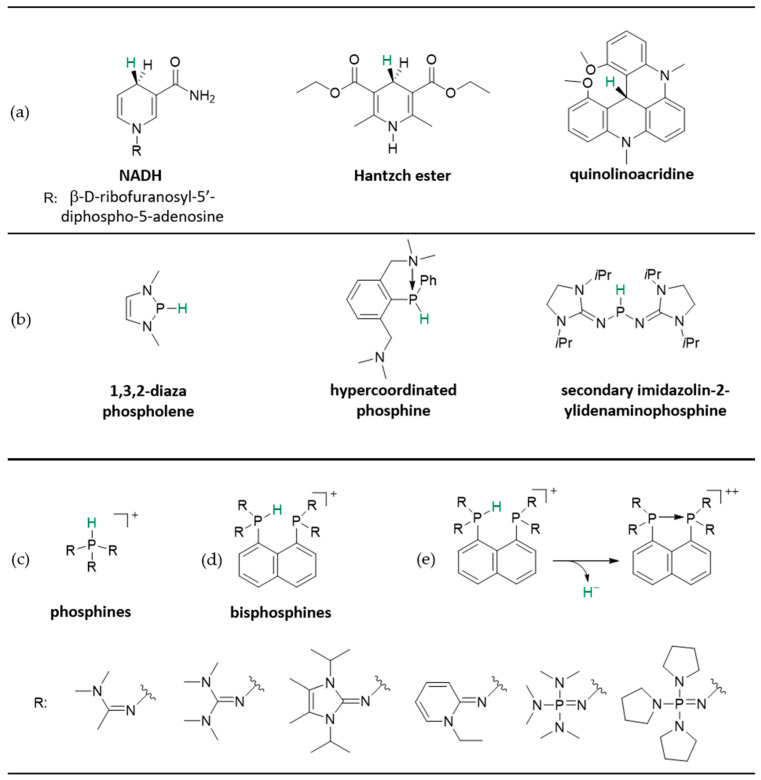
Representative classes of metal-free hydride donors: (**a**) biomimetic hydride donors; (**b**) systems based on secondary phosphines; (**c**) protonated phosphine–based systems; (**d**) bisphosphine architectures with closely positioned phosphine groups enabling enhanced hydricity; (**e**) Stabilization of the bisphosphine dication via intramolecular P→P dative bond formation.

Hydride transfer reactions lie at the heart of synthetic chemistry, biological redox processes, and emerging energy-conversion technologies [1,2,3,4]. The ability to deliver a hydride (H^−^) to an electrophilic substrate governs key transformations, including carbonyl reduction [5], imine hydrogenation [6], and carbon dioxide conversion [7]. Historically, transition-metal hydrides have dominated hydride transfer catalysis [8,9]. Metal hydride complexes, such as those of ruthenium [10], iron [11], or nickel [12], have been widely used to catalyze organic transformations, including the hydrogenation of carbonyl compounds and the reduction of imines via hydride transfer from the metal to the substrate. Recently, the hydrogenation of CO_2_ has attracted significant attention, with most catalytic systems relying on noble-metal hydride complexes, such as those of iridium, ruthenium, rhodium, and osmium, which transfer hydride to CO_2_ in key reduction steps [13,14,15]. The thermodynamic hydricity of metal complexes can be tuned over a wide energy range (>50 kcal mol^−1^) by varying metal identity, oxidation state, ligand electronic properties, and solvent environment, enabling precise matching of donor strength to substrate requirements. This tunability has underpinned highly efficient catalytic systems for hydrogenation and CO_2_ reduction. Despite some advantages, metal hydrides present inherent limitations. Some high-performance systems rely on noble metals that are naturally abundant in limited quantities and are costly. Catalyst stability can be compromised by aggregation, ligand dissociation, or metal nanoparticle formation. In addition, metal-centered reactivity may introduce competing pathways (e.g., single-electron transfer or undesired coordination chemistry) that reduce chemoselectivity. From a sustainability perspective, the environmental and economic implications of precious metal use motivate the exploration of alternative hydride-donor platforms. In this context, metal-free hydride donors, in which hydride is bound to main-group elements such as carbon, boron, silicon, or nitrogen, have emerged as powerful alternatives. Representative classes include biomimetic dihydropyridine systems (e.g., NADH analogs; Scheme 1a) [7,16], borohydrides and boranes [17,18], silanes [19], and organocatalytic hydride donors activated through Lewis acid-base cooperation [20]. Other well-established hydride donors in which hydride release occurs from the phosphorus atom are 1,3,2-diazaphospholenes [21,22,23] and some secondary phosphines (Scheme 1b). In 1,3,2-diazaphospholenes, as in NADH analogs, their hydridic character arises mainly from aromatic stabilization of the cation formed upon hydride ion loss [24]. Other secondary phosphines are good hydride donors due to π-donating substituents at phosphorus, which polarize the P–H bond via negative hyperconjugation into the σ*(P–H) orbital, leading to P–H bond elongation and polarity inversion (umpolung) [24,25,26]. Although some phosphines exhibit relatively strong hydride-donating ability, they can also behave as exceptionally strong Brønsted bases (superbases), particularly certain tertiary phosphines bearing strong electron-donating groups, as demonstrated primarily by Dielmann [27,28,29] and Sundermeyer [30]. Recently, we investigated whether bringing two phosphazenylphosphine groups into proximity within a proton-sponge-like framework enhances their basicity and whether a proton-chelating effect in the resulting bisphosphines operates similarly to that in nitrogen-based proton sponges. Although the systems studied exhibit superbasic character, it appears that the benefit arising from proton chelation is substantially smaller than that observed in nitrogen proton sponges [31]. In a related computational investigation, we recently showed that, contrary to chemical intuition, protonated phosphines bearing strong electron-donating substituents (Scheme 1c) can act as hydride donors [32]. The origin of their ability to act as hydride donors lies in the resonance stabilization of the dication formed upon hydride release by strongly electron-donating groups [32]. The hydricity of the examined phosphines spans a range from 95.3 to 71.8 kcal mol^−1^ in acetonitrile. It should be noted that hydricity, $\Delta {G{}^{\circ}}_{H^{-}}$ (hydride donor strength), is defined thermodynamically as the Gibbs energy change for the reaction:

A–H → A^+^ + H^−^

Because hydride release is generally endergonic in the absence of a hydride acceptor, $\Delta {G{}^{\circ}}_{H^{-}}$ values are positive; thus, lower hydricity values correspond to stronger hydride donors with a greater tendency to transfer H^−^ to an acceptor.

Glusac and co-workers [7] have proposed a classification of hydrides based on the magnitude of their hydricity, wherein compounds with hydricity values greater than 80 kcal mol^−1^ in acetonitrile are categorized as weak hydride donors, those with values between 80 and 45 kcal mol^−1^ as moderate hydride donors, and those with hydricity values below 45 kcal mol^−1^ as strong hydride donors. According to those classifications, tertiary protonated phosphines (PR_3_) are moderate or weak hydride donors. However, as shown in our study, hydricity of phosphines can be substantially enhanced by arranging two phosphine groups in close proximity in architectures reminiscent of proton sponges (Scheme 1d) [32]. In such monoprotonated bisphosphines, the increased hydricity compared to monophosphines (PR_3_) arises from stabilization of the resulting dication through formation of a dative P→P bond (Scheme 1e). The phosphorus atom that donates the hydride develops an empty p orbital, which is subsequently occupied by a lone pair donated from the neighboring phosphorus atom, thereby stabilizing the dicationic species. The calculated hydricities of all investigated monoprotonated bisphosphines in acetonitrile are below 45 kcal mol^−1^ (lower $∆G_{H^{-}}$ values correspond to stronger hydride donors). Accordingly, these species can be classified as strong hydride donors. Remarkably, two monoprotonated bisphosphines exhibited hydricity even lower than that of triethylborohydride (also called Superhydride), which has a $∆G_{H^{-}}$ of 25 kcal mol^−1^ in acetonitrile [1], underscoring their exceptional hydridic character.

In this work, we examine the extent to which the hydride-donating ability of protonated bis(phosphazenyl)phosphines can be enhanced by employing this novel approach to the design of strong organic hydrides. We further investigate the gas- and solution-phase basicity of the corresponding neutral and monoprotonated species.

## 2. Results and Discussion

### 2.1. Superhydricity of Monoprotonated Bis(phosphazenyl)phosphines

**Figure 1:**
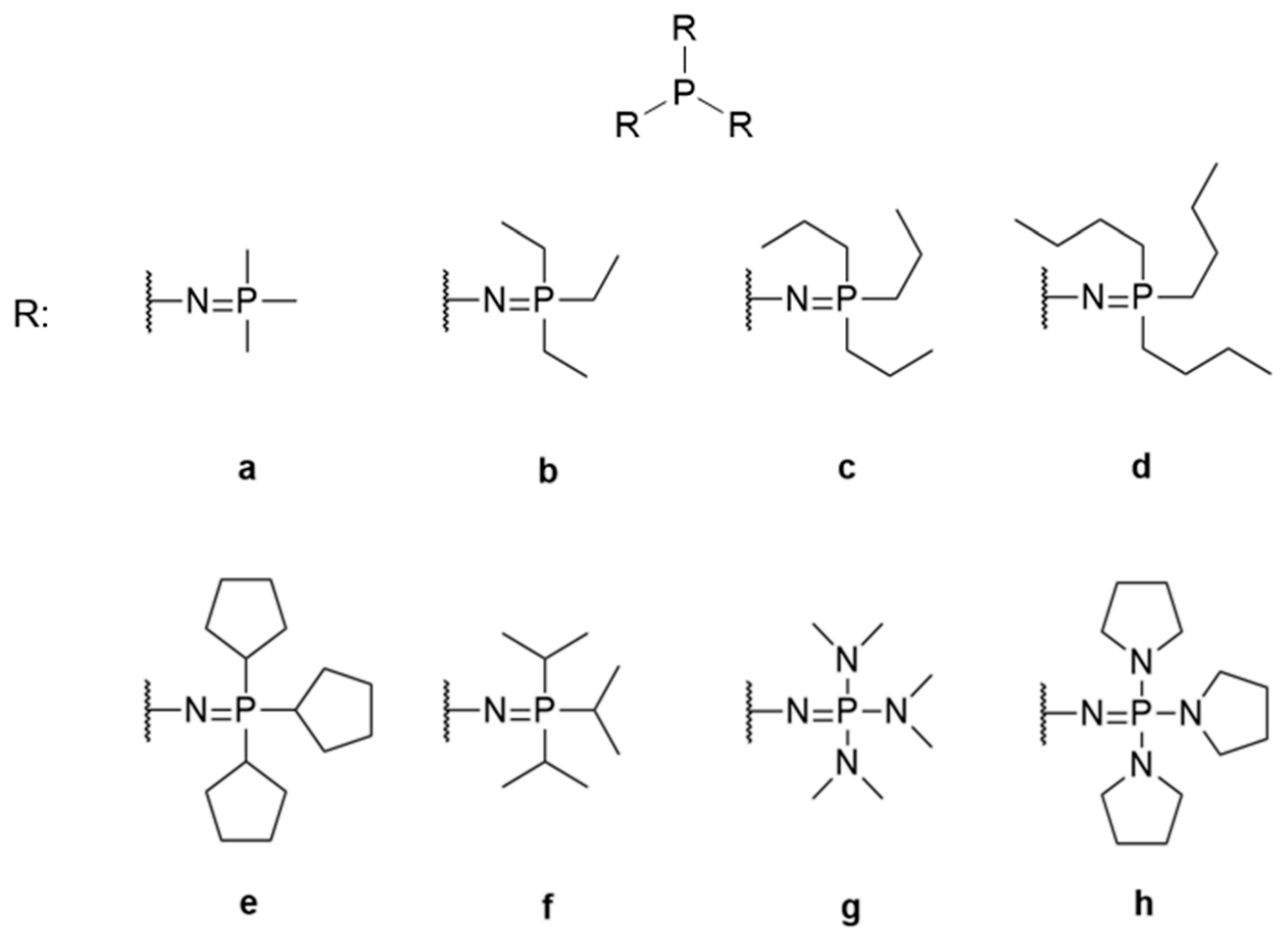
Tertiary phosphines bearing phosphazenyl groups.

As mentioned above, we calculated the hydricity of protonated tertiary phosphines bearing amidino, tetramethylguanidino, imidazolin-2-ylidenamino, pyridin-2-ylidenamino, and phosphazenyl substituents (Scheme 1c) [32], which have previously been synthesized and investigated in the context of exceptionally strong Brønsted bases [27,28,29,30]. Among the systems studied, those containing phosphazenyl groups with dimethylamino or pyrrolidinyl substituents at the phosphorus atom of the phosphazenyl group were identified as the strongest hydride donors and also exhibited the highest Brønsted basicity. However, in one of our previous studies on the basicity of superbasic alkyl-substituted bisphosphazene proton sponges, we demonstrated that alkyl phosphazenes bearing substituents such as methyl, *n*-butyl, isopropyl, and cyclopentyl at phosphorus display basicities comparable to those of systems containing dimethylamino or pyrrolidinyl substituents [33]. To investigate the influence of alkyl substituents within phosphazenyl groups on hydricity, we first calculated the hydricities of the compounds **PR_3_** (R = **a**–) and compared them with the hydricity of **PR_3_** (R = **g**–**h**) (Figure 1). For the calculation of hydricity, the (PCM)ωB97XD/6-311++G(d,p)//(PCM)ωB97XD/6-31G(d,p) computational model was employed, as in our previous study on the hydricity of bisphosphines [31]. Results are presented in Table 1. Although the phosphazenyl phosphine bearing pyrrolidyl substituents on the phosphazenyl group remains the strongest hydride donor, the differences in hydricity are relatively small. In fact, the gap between the strongest and the weakest hydride donor amounts to only 5.6 kcal mol^−1^. Particularly interesting, and somewhat unexpected, is the observation that a methyl substituent on the phosphorus atom of the phosphazenyl group in the examined phosphines results in nearly the same hydricity as ethyl, propyl, butyl, and cyclopentyl substituents. One would expect that longer alkyl chains would act as stronger electron donors and, through the inductive effect, provide greater stabilization of the dication formed after hydride donation. It should be noted, however, that the inductive effect also stabilizes the protonated form of the phosphine. Since hydricity is defined as the difference in Gibbs free energy between the protonated species and the corresponding dication, the observed behavior evidently reflects an interplay of these effects on both forms of the phosphine. The observation that even a sterically undemanding methyl substituent on the phosphazenyl group affords hydricity comparable to that of much bulkier isopropyl, dimethylamino or pyrrolidinyl substituents may be advantageous for the design of strong hydride-donating bisphosphines. Because the phosphine groups become significantly closer in the corresponding dications, bulky substituents could give rise to steric repulsion, which may destabilize the dication and consequently lower hydricity. NBO atomic charges were also computed (Table S1), and they indicate that the hydrogen atom bound to phosphorus bears a partial negative charge. This finding is consistent with an inverted polarity of the P–H bond (i.e., hydride-like character). Such polarity inversion has previously been reported for secondary phosphines (Scheme 1b) and was recently observed for the protonated tertiary phosphines shown in Scheme 1c [32].

We further examined the relationship between the hydrogen atomic charge and hydricity to establish a simple predictive descriptor. However, no correlation was found (*R*^2^ = 0.17), suggesting that local charge alone is insufficient to capture the thermodynamic driving force for hydride transfer.

**Figure 2:**
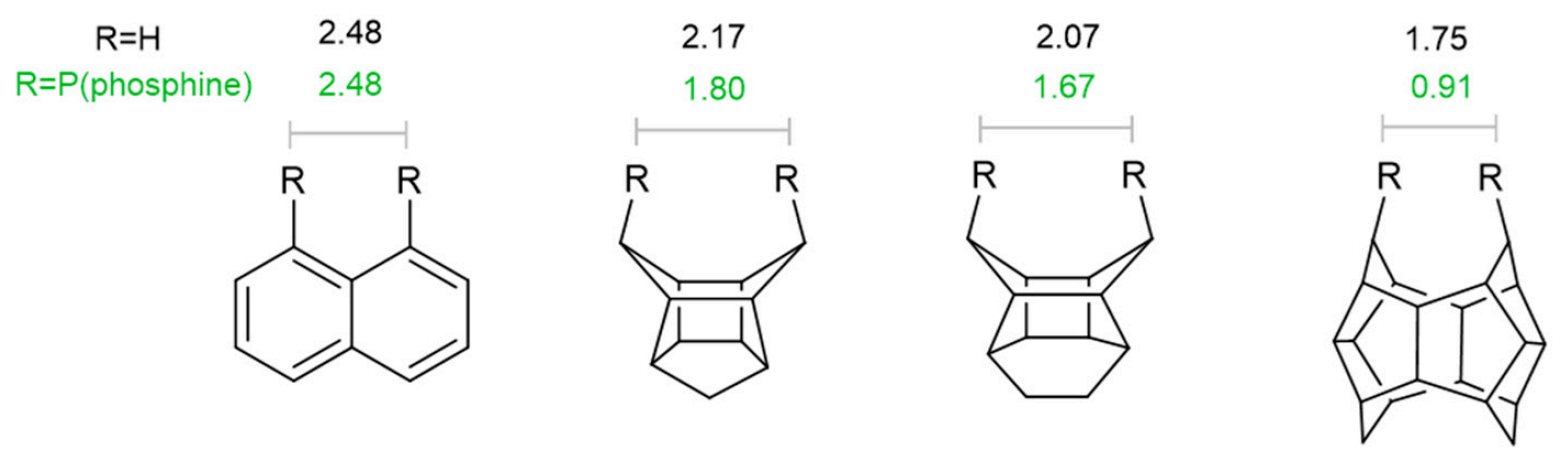
Schematic representation of naphthalene, pentacyclo [5.4.0.0^2,6^.0^3,10^.0^5.9^]undecane, pentacyclo [6.4.0.0^2,7^.0^3,11^.0^6,10^]dodecane, and seco-dodecahedradiene. The distances between R groups (in Å) are shown for R = H (black) and R = P(phosphine) (green). In the latter case, the aryl C–P bond length is fixed at 1.83 Å, corresponding to the average P–C bond length in the studied systems.

In our previous work, we investigated the hydricity of protonated bisphosphines using a naphthalene scaffold as the backbone for the phosphine groups (Scheme 1d) [32]. This motif is also among the most commonly used backbones in the design of proton sponges, i.e., strongly basic Brønsted bases. However, it has been shown that backbones based on aliphatic frameworks such as pentacyclo [5.4.0.0^2,6^.0^3,10^.0^5.9^]undecane [34], pentacyclo [6.4.0.02,7.03,11.06,10]dodecane [34], and seco-dodecahedradiene [35] can, in certain cases, offer advantages over aromatic ones for several reasons. One important factor is that backbone modification alters the distance between substituents, as illustrated in Figure 2, thereby affecting the ability of the protonated species to form intramolecular hydrogen bonds [31]. In cases where protonated bisphosphines act as hydride donors, this imposed distance between substituents may have an even more pronounced effect due to the formation of a stabilizing P→P dative bond upon hydride release. Previous studies have shown that the P– distance in bisphosphine dications based on a naphthalene scaffold is, on average, ~2.2 Å [31]. In contrast, the geometric constraint imposed by the naphthalene backbone corresponds to a separation of approximately 2.5 Å, i.e., somewhat longer than the optimal P–P distance observed in the corresponding dications.

**Figure 3:**
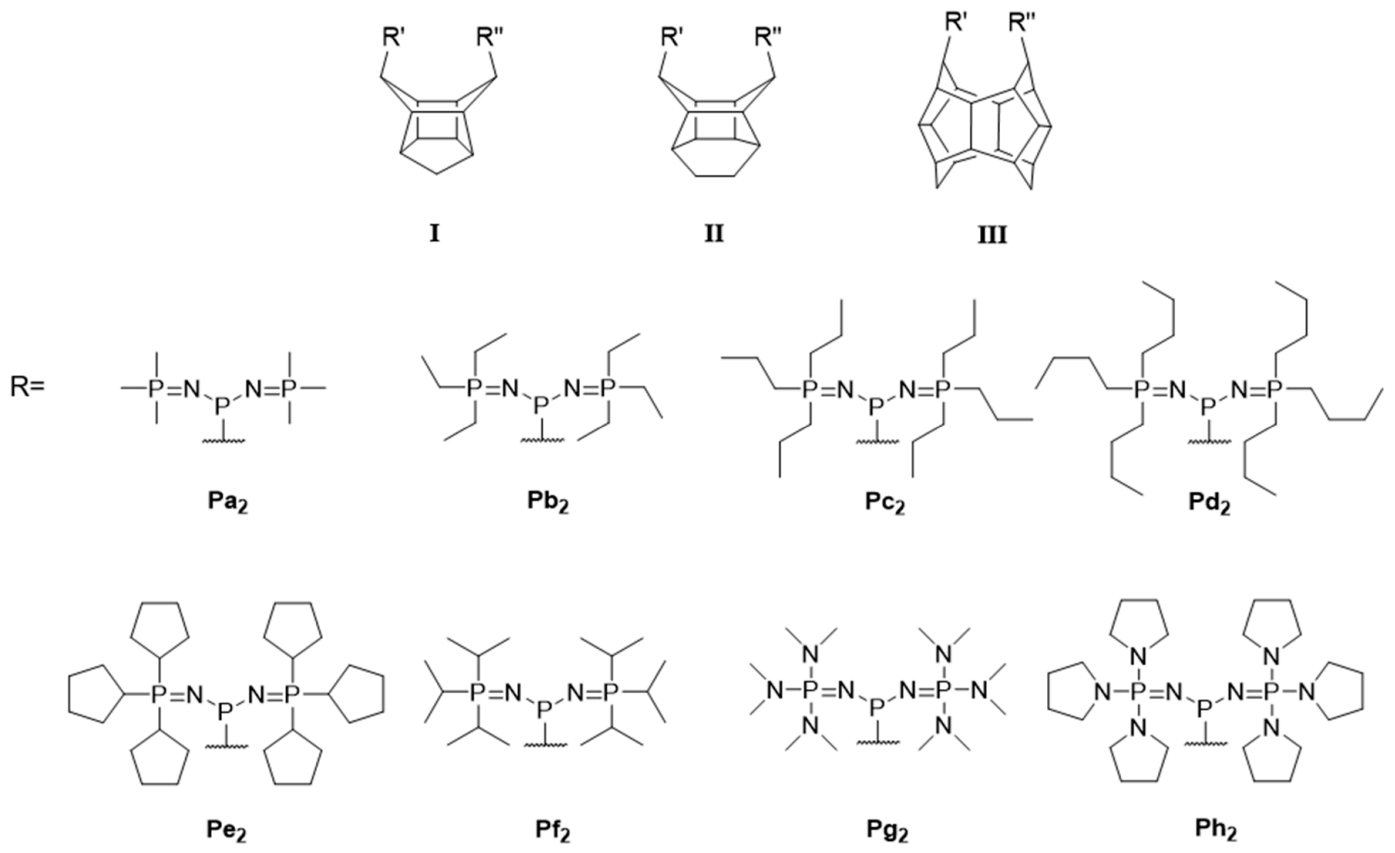
Schematic representation of the bisphosphines and monophosphines studied in this work.

**Scheme 2:**
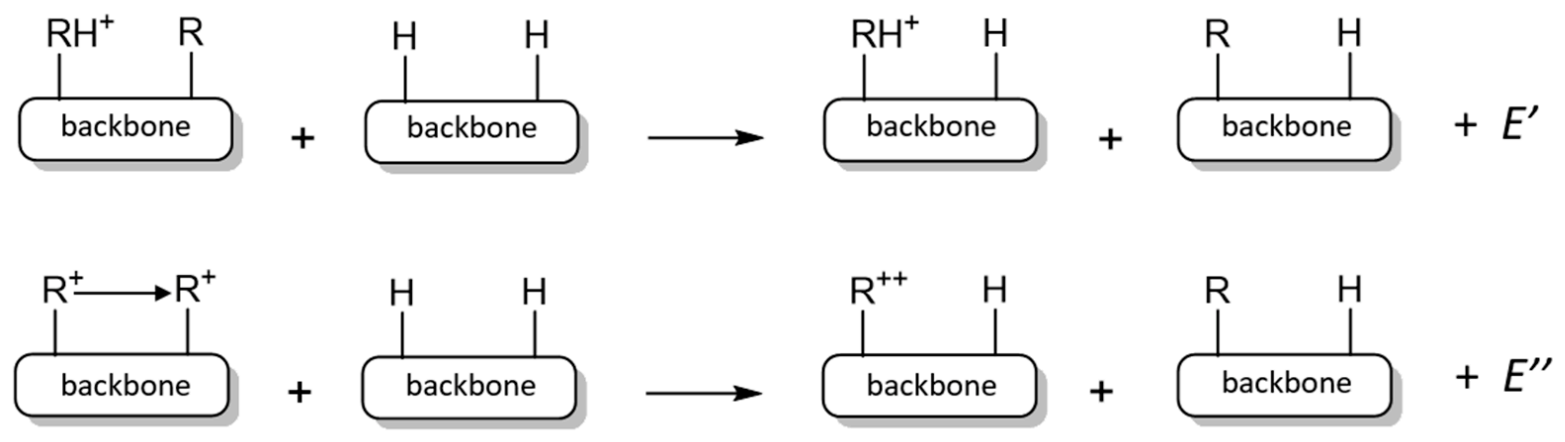
Homodesmic reactions used to estimate the interaction between phosphine groups in protonated bisphosphines and the corresponding dications. *E*′ and *E*″ correspond to Gibbs energy of the respective homodesmic reaction.

As illustrated in Figure 2, the aliphatic backbones employed here enforce significantly shorter separations between the phosphorus centers, which is expected to facilitate the formation of a P–P interaction in the dications, thereby enhancing their stability and, consequently, modulating their hydricity. Therefore, in this work, we investigated the hydricities of bisphosphines in which the phosphine moieties are linked by the aliphatic scaffolds shown in Scheme 2, while the substituents on the phosphines correspond to those depicted in Figure 1. All studied systems are schematically illustrated in Figure 3. Monophosphine systems (R′ = phosphazenylphosphine group; R″ = H) were examined to assess the intrinsic effect of the scaffold on hydricity, as well as to disentangle the contribution of interphosphine interactions in bisphosphines to the overall hydricity. With respect to the former, it should be noted that, in bisphosphine design, one strongly electron-donating phosphazenyl substituent in PR_3_ is necessarily replaced by a less electron-donating alkyl group. This substitution intrinsically reduces the hydride-donating ability relative to tertiary phosphines (PR_3_) bearing three phosphazenyl substituents (Figure 1). This trend is evident in Table 2 for the monosubstituted systems **I**–**III** (R′ = **Pa_2_**–**Pf_2_**; R’’ = H). Comparison of their hydricities with those of the corresponding phosphines **PR_3_** (R = **a**–**f**) shows that the former are, on average, weaker hydride donors by approximately 10 kcal mol^−1^. This decrease in hydride donor strength is substantially smaller than that observed for the naphthalene scaffold, where reductions of 14.9 and 21.4 kcal mol^−1^ are obtained for systems bearing **Pg_2_** and **Ph_2_** phosphine moieties, respectively [32]. This trend reflects the reduced ability of the naphthalene scaffold to stabilize the cationic species formed upon hydride transfer, in contrast to the more strongly σ-donating aliphatic scaffolds **I**–**III**. Comparison of the effects of scaffolds **I**–**III** on the hydricity of monophosphines reveals only minor differences among them. However, a slight trend of decreasing Δ*G*_H_^−^ is observed from **I** to **III**, which can likely be attributed to the stronger electron-donating (+*I*) effect exerted by more highly substituted alkyl groups. Although tertiary phosphine systems PR_3_, similarly to monophosphines (**I**–**III**; R′ = H, R″ = **Pa_2_**-**Ph_2_**), exhibit only small variations in hydricity regardless of the substituent on phosphorus and the nature of the aliphatic scaffold, data in Table 1 shows that in the case of bisphosphines these differences become significantly more pronounced, which is evident from the wide range of hydricities, spanning from 29.9 kcal mol^−1^ for **I** (R′ = R″ = **Pe_2_**) to a remarkably low value of 0.8 kcal mol^−1^ for **III** (R′ = R″ = **Pd_2_**). It can also be observed that scaffolds **I** and **II** yield comparable hydricity values, with scaffold **II** giving slightly lower ones, whereas scaffold **III** results in substantially lower hydricities for the corresponding phosphine moieties. The hydricities of the studied bisphosphines (Figure 3, Table 2) reveal that, except for **I** (R′ = R″ = **Pe_2_**), all systems exhibit hydricities comparable to or lower than that of lithium triethylborohydride (commonly referred to as Superhydride). Although “superhydricity”, in contrast to superbasicity, has not been formally defined yet, we propose that compounds with $∆G_{H^{-}}$ values below 25 kcal mol^−1^ in acetonitrile be classified as superhydrides, and that “superhydride” be adopted as a general term for this class of compounds. A similar definition has recently been introduced for superbasicity in acetonitrile [36].

Given our previous conclusion that scaffolds **I**–**III**, when acting as substituents on phosphines, do not significantly affect hydricity, it follows that the observed differences in hydricity among bisphosphines incorporating scaffolds **I**, **II**, and **III** originate from intramolecular interactions between the two phosphine fragments, both in the protonated form and in the dication.

Therefore, using the homodesmic reactions shown in Scheme 2, we aimed to quantify these intramolecular interactions. The interaction Gibbs energies in the protonated form of the bisphosphines are denoted as *E*′, whereas those in the dicationic form are denoted as *E*″. The calculated values are summarized in Table 3.

It is noteworthy that most *E*′ values reported in Table 3 are positive. This indicates that, in the protonated form of the bisphosphines, interactions between the phosphine groups are overall destabilizing. This behavior contrasts with that typically observed for proton sponge–like systems bearing amino or imino substituents, for which the corresponding *E*′ values are generally negative [37]. This behavior can be attributed to the substantially weaker intramolecular P–H···P interaction [38] compared to the N–H···N hydrogen bonding commonly found in amine- or imine-based systems [39,40]. In addition, the optimal P···P distance required for effective P–H···P interaction is significantly larger than the corresponding N···N distance for N–H···N hydrogen bonding. The scaffolds employed in the present bisphosphine design impose P···P separations considerably shorter than is optimal for a P–H···P hydrogen bond, resulting in a net destabilizing interaction in the protonated form. Additional support for this interpretation is provided by scaffold **III**, which enforces the shortest P···P distances between scaffolds (**I**–**II**), and correspondingly, protonated bisphosphines exhibit the largest *E*′ values on average. In contrast, the *E*″ values are strongly negative. The dominant contribution to these highly stabilizing values arises from the formation of a dative P→P interaction in the bisphosphine dication. It is noteworthy that scaffold **III** provides a smaller stabilization energy than scaffolds **I** and **II.** This is consistent with the observation that P–P distances in bisphosphine dications (Table S2) vary only slightly, spanning a narrow range of 2.197–2.253 Å. This distance range appears to be optimal for the dication; consequently, scaffold III, which enforces the shortest P···P separation among the three scaffolds (Figure 2), provides less stabilization of the dication. However, despite this, scaffold III yields bisphosphines that are the strongest hydride donors. This indicates that the pronounced hydricity of the scaffold **III**–based bisphosphines does not originate from some additional stabilization of the dication through a potentially shorter P→P interaction. Rather, it most likely arises from the release of the above-mentioned destabilizing interactions present in the protonated species, which diminish upon hydride transfer.

### 2.2. CO_2_ Reduction to Formate

**Figure 4:**
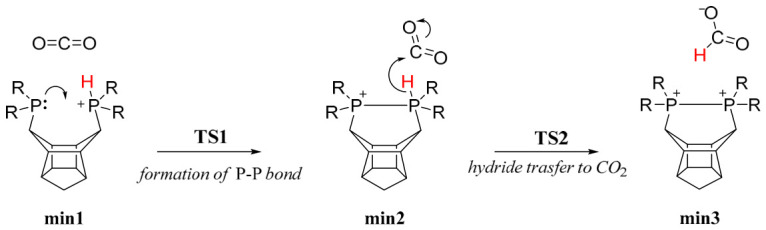
Reaction pathway of CO_2_ reduction to formate.

**Figure 5:**
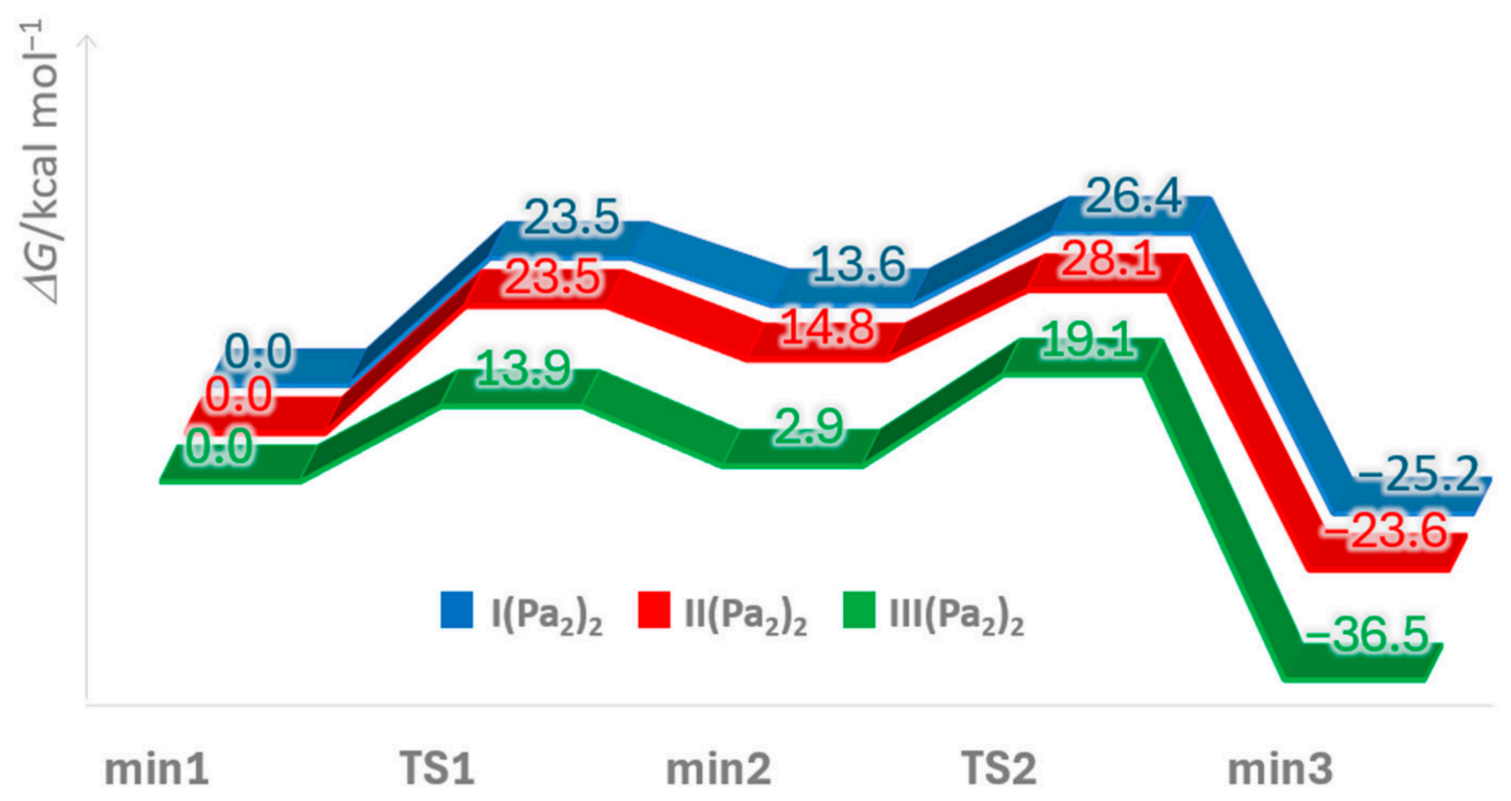
Energy profile for CO_2_ reduction to formate by three selected protonated bisphosphines.

The reducing ability of selected protonated bisphosphines was evaluated with respect to CO_2_. It is well established that CO_2_ is difficult to reduce due to its high thermodynamic stability. In the case of reduction to formic acid, strong hydride donors are required, as the hydricity of the formate anion is 45 kcal mol^−1^. Therefore, an effective reducing agent must possess a hydricity lower than 45 kcal mol^−1^ [7]. All systems studied here meet this criterion and should, from a thermodynamic standpoint, be capable of reducing CO_2_. To examine this in more detail, we calculated the reaction energy profiles for CO_2_ reduction to formate for three representative bisphosphine systems: **I(Pa_2_)_2_**, **II(Pa_2_)_2_**, and **III(Pa_2_)_2_.** Consistent with previous findings [32], the hydride transfer to CO_2_ proceeds in two steps, as depicted in Figure 4. In the first step, a relatively high-energy intermediate (min2), in which the two phosphorus atoms are bonded, is formed via transition state TS1. In the second step, hydride transfer to CO_2_ occurs via transition state TS2, yielding the bisphosphine dication and the formate anion. The corresponding energies are summarized in Figure 5. Transition state TS1 corresponds to P–P bond formation and is associated with relatively low activation barriers, not exceeding 23.5 kcal mol^−1^. In contrast, the barriers for hydride transfer are somewhat higher, with the highest value being 28.1 kcal mol^−1^ for system **II(Pa_2_)_2_**. All three studied reactions are highly exergonic.

### 2.3. Superhydricity of Bis(phosphazenyl)phosphines

In our previous work, we reported that (bis)phosphazenylphosphines based on a naphthalene scaffold exhibit superbasic properties both in the gas phase and in acetonitrile solution. As is well established, superbasicity in the gas phase is defined by proton affinities exceeding that of 1,8-bis(dimethylamino)naphthalene (DMAN), with a PA of 245.7 kcal mol^−1^ [41,42]. In acetonitrile, superbasicity refers to bases whose conjugate acids have p*K*_BH+_ ≥ 25, based on the reference value for 1,8-bis(tetramethylguanidino)naphthalene (TMGN) [36]. In Table 4, we reported gas-phase proton affinity and gas basicity for first and second protonation as well as first and second p*K*_BH+_ values. There are two reasons for examining the basicity. First, we sought to determine whether (bis)phosphazenylphosphines exhibit higher or lower basicity than their analogs based on the naphthalene scaffold. Second, we aimed to assess whether the difference between the first and second p*K*_BH+_ values is sufficiently large, which is an important criterion for the feasibility of isolating monoprotonated bisphosphines. It should be emphasized that only the monoprotonated bisphosphine species exhibit superhydride behavior. In contrast, the diprotonated forms do not, as the formation of a P→P dative interaction is not possible in these species. Although a somewhat different theoretical model was employed in our previous work to compute PA, GB, and p*K*_BH+_ values for naphthalene-based bis(phosphazenyl)phosphines, precluding a direct and rigorous comparison, the data in Table 4 nevertheless indicate that, similarly to bis(phosphazenyl)phosphines based on a naphthalene scaffold [31], those based on aliphatic scaffolds (**I**–**III**) also exhibit superbasic properties both in the gas phase and in solution. Specifically, the proton affinities span the range of 286–300.9 kcal mol^−1^, except for an unresolved anomaly in **I-Pc_2_**. It should be noted that compounds with PA values exceeding 300 kcal mol^−1^ are commonly classified as hyperbases [43]. In contrast, the proton affinities associated with protonation of the second phosphazenylphosphine unit, except in the case of **III-Ph_2_**, do not exceed 245.7 kcal mol^−1^. This indicates that the monoprotonated bis(phosphazenyl)phosphines do not exhibit superbasicity in the gas phase with respect to the second protonation step. Analysis of the p*K*_BH+_ values in acetonitrile reveals additional insights. The first protonation constants reach up to 46.9, indicating exceptionally strong basicity. These values are generally somewhat higher than those reported for bis(phosphazenyl)phosphines based on a naphthalene scaffold, for which the highest p*K*_BH+_ value was 42.0. Notably, the p*K*_BH+_(1) values do not differ significantly among systems bearing equivalent phosphine substituents, regardless of whether scaffold **I**, **II**, or **III** is employed. This behavior contrasts with that observed for hydricity and may be attributed to the strain present in the protonated form, as discussed above. Contrary to the gas phase, the second p*K*_BH+_ value ranges from 25.8 to 36.5, indicating that the monoprotonated form also exhibits superbasicity in acetonitrile according to the definition mentioned above.

## 3. Materials and Methods

### Computational Details

All density functional theory (DFT) calculations were performed using the Gaussian 16 computational chemistry software package [44]. Conformational spaces of the investigated molecules were explored with the CREST (Conformer–Rotamer Ensemble Sampling Tool) program developed by Grimme and co-workers [45]. In this approach, conformer sampling and preliminary energy ranking were carried out using the GFN2-xTB semiempirical tight-binding method [46]. The fifteen lowest-energy conformers obtained from the CREST search were used as initial geometries for DFT geometry optimizations, and the structure with the lowest energy was selected for further analysis. Geometry optimizations of the minima were carried out using the ωB97XD functional [47,48] together with the Pople 6-31G(d,p) basis set [49]. Solvent effects were included through the PCM continuum solvation model [50] for acetonitrile at 298 K. All structures were optimized without imposing geometric constraints. Vibrational frequency calculations were performed at the same level of theory to verify the nature of the stationary points (all positive eigenvalues for minima and one imaginary frequency for transition-state structures). Default convergence criteria for the SCF procedure and geometry optimization were employed, along with the standard integration grid implemented in Gaussian 16. Single-point energy is calculated at ωB97XD/6-311++G(d,p) level of theory; therefore, the applied theoretical model can be written as (PCM)ωB97XD/6-311++G(d,p)//(PCM)ωB97XD/6-31G(d,p).

The thermodynamic hydricity was determined as relative hydricity by calculating *Δ*_r_*G* for the following reaction:

***P***H^+^ + CO_2_ → ***P***^2+^ + HCOO^−^ (1)

and taking an experimental value for the hydricity of HCOO^−^ of 44 kcal mol^−1^. (Here, ***P*** = phosphine framework; ***P***H^+^ = protonated hydride-donating species.)

The Gibbs energy of reaction (1) is easily obtained by calculating the absolute values of the Gibbs energy of solvated reactants and products. However, it cannot directly provide the hydricity of ***P***H^+^.

Equation (1), i.e., hydride transfer from ***P***H^+^ to HCOO^−^, can be written as a combination of two reactions:

***P***H^+^ → ***P***^2+^ + H^−^  *Δ*_r_*G* (2) = *ΔG*_H_^−^(***P***H^+^) (2)

CO_2_ + H^−^ → HCOO^−^ *Δ*_r_*G* (3) = −*ΔG*_H_^−^(HCOO^−^) (3)

The Gibbs energy of reaction (2) is the hydricity of ***P***H^+^, while the Gibbs energy of reaction (3) is a negative value of the hydricity of HCOO^−^. Considering the known hydricity of HCOO^−^, it is possible to combine it with the Gibbs energy of (1) to obtain the hydricity of ***P***H^+^. Namely:

*Δ*_r_*G*(1) = *Δ*_r_*G*(2) + *Δ*_r_*G*(3) = *Δ*_r_*G*_H_^−^(***P***H^+^) − *ΔG*_H_^−^(HCOO^−^) (4)

Thus, the hydricity of ***P***H^+^ can be obtained according to reaction (5):

*Δ*_r_*G*_H_^−^(***P***H^+^) = *Δ*_r_*G*(1) + *ΔG*_H_^−^(HCOO^−^) (5)

Gas-phase basicities (GB) were calculated as the negative Gibbs energy *ΔG* of the reaction

B + H^+^ → BH^+^ (6)

as follows: GB = −{*G*^298^(BH^+^) − [*G*(B^298^) + *G*^298^(H^+^)]}. The Gibbs energy of the proton in the gas phase, *G*^298^(H^+^), has a value of −6.295 kcal·mol^−1^ [51]. Proton affinities (PA) were calculated as the negative enthalpy for the aforementioned reaction (6) as follows: PA = −{*H*^298^(BH^+^) − [*H*(B^298^) + *H*^298^(H^+^)]}. The enthalpy of proton equals 1.48 kcal mol^−1^. *G* and *H* values are obtained using the computational model mentioned above. p*K_BH+_* values in acetonitrile were calculated using the same functional, basis set, and solvation model as in the calculation of hydricities. p*K*_BH+_ values were determined as relative values using an isodesmic reaction approach [52] with trimethylphosphine (experimental p*K*_BH+_ = 15.5) [40] serving as the reference base.

## 4. Conclusions

In summary, the present DFT results indicate that monoprotonated bis(phosphazenyl)phosphines based on aliphatic scaffolds act as exceptionally strong hydride donors, exceeding the hydricity of lithium triethylborohydride (LiBEt_3_H, “Superhydride”) and outperforming their naphthalene-based analogs. This enhanced hydride-donating ability can be rationalized by the strong electron-releasing character of the substituents at phosphorus, combined with stabilization of the dicationic product through intramolecular P→P interaction following hydride transfer. In certain cases, most notably for scaffold III, an additional contribution arises from the relief of destabilizing interactions present in the protonated precursor. On this basis, an operational definition of superhydricity in acetonitrile is proposed, whereby species with *ΔG*°_H_^−^ values below 25 kcal mol^−1^—i.e., exceeding the hydride-donating ability of LiBEt_3_H—may be classified as superhydrides. Furthermore, the corresponding neutral bis(phosphazenyl)phosphines display pronounced superbasicity in both the gas phase and acetonitrile, underscoring the dual superbase/superhydride character of these systems.

## Figures and Tables

**Table 1:** Hydricity of protonated tertiary phosphines (**PR_3_**).

PR_3_ R=	a	b	c	d	e	f	g	h
$∆G_{H^{-}}/kcal{mol}^{-1}$	77.4	78.6	77.9	77.1	77.3	75.1	76.8	73.0

**Table 2:** Calculated hydricity of bisphosphines and their mono-phosphine analogs in acetonitrile.

Backbone	Molecule	$∆G_{H^{-}}/kcal\;{mol}^{-1}$	Molecule	$∆G_{H^{-}}/kcal\;{mol}^{-1}$
I	R′ = R″ = **Pa_2_**	20.4	R′ = H, R″= **Pa_2_**	88.7
	R′ = R″ = **Pb_2_**	14.9	R′ = H, R″= **Pb_2_**	88.8
	R′ = R″ = **Pc_2_**	14.2	R′ = H, R″= **Pc_2_**	87.8
	R′ = R″ = **Pd_2_**	12.6	R′ = H, R″ = **Pd_2_**	86.5
	R′ = R″ = **Pe_2_**	29.3	R′ = H, R″ = **Pe_2_**	88.5
	R′ = R″ = **Pf_2_**	25.9	R′ = H, R″ = **Pf_2_**	88.5
	R′ = R″ = **Pg_2_**	18.8	R′ = H, R″ = **Pg_2_**	88.3
	R′ = R″ = **Ph_2_**	13.9	R′ = H, R″ = **Ph_2_**	88.0
II	R′ = R″ = **Pa_2_**	18.8	R′ = H, R″ = **Pa_2_**	87.9
	R′ = R″ = **Pb_2_**	9.9	R′ = H, R″ = **Pb_2_**	88.2
	R′= R″ = **Pc_2_**	11.9	R′ = H, R″ = **Pc_2_**	88.9
	R′ = R″ = **Pd_2_**	10.0	R′ = H, R″ = **Pd_2_**	87.8
	R′ = R″ = **Pe_2_**	24.0	R′ = H, R″ = **Pe_2_**	87.4
	R′ = R″ = **Pf_2_**	25.1	R′ = H, R″ = **Pf_2_**	87.1
	R′ = R″ = **Pg_2_**	14.9	R′ = H, R″ = **Pg_2_**	86.9
	R′ = R″ = **Ph_2_**	12.3	R′ = H, R″ = **Ph_2_**	85.7
III	R′ = R″ = **Pa_2_**	7.5	R′ = H, R″ = **Pa_2_**	85.2
	R′ = R″ = **Pb_2_**	3.5	R′ = H, R″ = **Pb_2_**	84.9
	R′ = R″ = **Pc_2_**	7.5	R′ = H, R″ = **Pc_2_**	86.2
	R′ = R″ = **Pd_2_**	0.8	R′ = H, R″ = **Pd_2_**	86.3
	R′ = R″ = **Pe_2_**	17.7	R′ = H, R″= **Pe_2_**	86.4
	R′ = R″ = **Pf_2_**	16.8	R′ = H, R″= **Pf_2_**	84.4
	R′ = R″ = **Pg_2_**	7.9	R′ = H, R″= **Pg_2_**	86.7
	R′ = R″ = **Ph_2_**	0.9	R′ = H, R″= **Ph_2_**	84.3

**Table 3:** Energies (in kcal mol^−1^) of the homodesmic reactions depicted in Scheme 2 for all studied bisphosphines.

Bisphosphine	*E*′			*E*″		
R′ = R″	I	II	III	I	II	III
**Pa_2_**	1.1	1.7	8.9	−67.3	−67.3	−68.6
**Pb_2_**	4.5	7.5	11.6	−69.4	−70.9	−69.7
**Pc_2_**	4.1	5.2	7.8	−69.5	−71.8	−70.9
**Pd_2_**	0.5	2.2	6.2	−73.4	−71.5	−79.1
**Pe_2_**	−6.2	−2.3	8.1	−65.3	−65.7	−60.6
**Pf_2_**	3.3	1.5	9.9	−59.3	−60.5	−57.6
**Pg_2_**	2.3	3.3	8.2	−67.2	−68.7	−70.5
**Ph_2_**	−1.5	−1.2	11.1	−75.7	−74.6	−72.3

**Table 4:** First and second proton affinities and gas basicities, as well as first and second p*K*_BH+_ values of bisphosphines.

Backbone	Bisphosphine	Proton Affinity (PA) and Gas Basicity (GB)				p*K*_BH+_ Values	
	R1=R2=	PA(1)	PA(2)	GB(1)	GB(2)	p*K*_BH+_(1)	p*K*_BH+_(2)
**I**	**Pa_2_**	287.6	217.0	280.6	211.8	38.0	27.2
	**Pb_2_**	290.5	230.4	283.7	223.1	41.4	30.5
	**Pc_2_**	279.6	232.4	273.2	226.4	41.1	32.2
	**Pd_2_**	294.8	230.1	285.9	222.5	37.5	31.3
	**Pe_2_**	300.9	236.4	293.2	228.9	47.1	31.4
	**Pf_2_**	296.7	236.0	289.4	229.1	42.8	34.5
	**Pg_2_**	293.2	230.1	285.4	223.9	41.8	30.8
	**Ph_2_**	299.0	239.2	293.0	230.2	44.3	32.8
**II**	**Pa_2_**	287.5	217.0	280.8	211.6	38.4	25.8
	**Pb_2_**	286.2	233.5	279.7	226.2	41.0	32.5
	**Pc_2_**	292.0	231.8	283.4	225.3	38.8	30.7
	**Pd_2_**	288.5	234.7	281.5	226.1	41.5	30.1
	**Pe_2_**	298.1	235.3	290.2	229.4	46.9	30.2
	**Pf_2_**	296.8	233.9	288.4	227.6	44.8	33.4
	**Pg_2_**	294.4	230.3	285.8	225.5	41.3	30.9
	**Ph_2_**	300.9	231.7	291.7	233.2	42.6	30.9
**III**	**Pa_2_**	286.0	219.0	280.0	211.6	37.1	28.7
	**Pb_2_**	291.6	224.6	284.2	218.3	40.7	39.4
	**Pc_2_**	291.0	229.2	286.3	220.9	40.7	28.7
	**Pd_2_**	291.5	231.2	284.1	226.5	39.8	30.1
	**Pe_2_**	298.4	237.5	290.2	230.4	44.9	31.5
	**Pf_2_**	296.2	238.9	288.7	231.9	44.3	36.5
	**Pg_2_**	297.2	232.2	290.3	226.0	40.6	32.5
	**Ph_2_**	295.6	247.9	286.8	242.9	41.6	32.1

## Data Availability

The data are available in this publication and the Supplementary Materials.

## Supplementary Materials

The following supporting information can be downloaded at: https://www.mdpi.com/article/10.3390/molecules31091535/s1. Table S1. NBO charges on the H atom bonded to the central phosphorus atom in protonated tertiary phosphines (PR3), Table S2. P→P distances (in Å) in bisphosphine dications, Cartesian Coordinates of Optimized Structures (in acetonitrile).
