# Supplementary material for: Bis(phosphazenyl)phosphines: From Superbases to Superhydrides
Source: Molecules. 2026 May 5;31(9):1535. doi: 10.3390/molecules31091535 (PMC13164818; doi:10.3390/molecules31091535)
Supplement: Supplementary file 1 [file molecules-31-01535-s001.zip › molecules-4257068-supplementary.pdf]

Electronic supporting information

Bis(phosphazenyl)phosphines: From Superbases to Superhydrides

Mario Damjanović[1,2] and Borislav Kovačević[1,3]*

[1] Division of Physical Chemistry, Ruđer Bošković Institute, HR-10000 Zagreb, Croatia; [2] Faculty of Science, University of Zagreb, Horvatovac 102A, 10000-Zagreb, [3] Libertas University, Trg John F. Kennedy 6B, 10000, Zagreb

*Correspondence: Borislav.Kovacevic@irb.hr

Table of Contents

- Table S1……………………………………………………………………………………………………....1
- Table S2……………………………………………………………………………………………………....1
- Cartesian Coordinates of Optimized Structures (in acetonitrile)……………………………………..2-244

Table S1. NBO charges on the H atom bonded to the central phosphorus atom in protonated tertiary phosphines (**PR**$_3$).

**PR**$_3$ R=	**a**	**b**	**c**	**d**	**e**	**f**	**g**	**h**
**NBO charges**	−0.0650	−0.0762	−0.0702	−0.0816	−0.1005	−0.0964	−0.0711	−0.0843

Table S2. P→P distances (in Å) in bisphosphine dications.

Bisphosphine	P→P distance		
R′= R′′	**I**	**II**	**III**
**Pa**$_2$	2.219	2.211	2.200
**Pb**$_2$	2.213	2.208	2.200
**Pc**$_2$	2.209	2.197	2.202
**Pd**$_2$	2.216	2.217	2.199
**Pe**$_2$	2.253	2.230	2.239
**Pf**$_2$	2.220	2.210	2.217
**Pg**$_2$	2.222	2.213	2.198
**Ph**$_2$	2.218	2.209	2.209

Cartesian Coordinates of Optimized Structures

Pa_3 (dication)

C	2.6069990	-2.0169730	-1.4756760
P	2.7080750	-0.9875220	0.0002290
C	2.5882610	-2.0499630	1.4514330
N	1.5478700	0.1723540	0.0039570
P	-0.0010930	-0.0025250	-0.0093400
N	-0.6247320	-1.4313570	-0.0163420
P	-2.2114480	-1.8493920	-0.0012320
C	-3.0833000	-1.1975590	-1.4382060
C	4.2864190	-0.1305010	0.0194670
C	-2.2648870	-3.6443700	-0.0304730
C	-3.0318570	-1.2510530	1.4879690
H	-1.7691490	-4.0039740	-0.9339180
H	-1.7466040	-4.0313320	0.8489110
H	-3.3036530	-3.9800980	-0.0220530
H	-3.0554710	-0.1061870	-1.4046560
H	-2.5925120	-1.5489500	-2.3477530
H	-4.1207770	-1.5385680	-1.4270230
H	-3.0109570	-0.1591140	1.4905860
H	-4.0673240	-1.5976140	1.5032480
H	-2.5056640	-1.6301920	2.3660380
H	2.6698580	-1.3823710	-2.3617260
H	3.4298940	-2.7348750	-1.4796910
H	1.6541810	-2.5506750	-1.4734640
H	4.3503410	0.4842220	0.9191340
H	5.0990300	-0.8596630	0.0145510
H	4.3593670	0.5064080	-0.8640190
H	2.6444050	-1.4364240	2.3525700
H	1.6339130	-2.5804060	1.4275000
H	3.4082640	-2.7711990	1.4472850
N	-0.9270850	1.2519980	-0.0155770
P	-0.4985440	2.8356150	-0.0000570
C	0.3708140	3.2634570	1.5192010
H	1.3069320	2.7025580	1.5626040
H	-0.2518920	3.0058950	2.3778760
H	0.5851130	4.3342530	1.5286600
C	-2.0245200	3.7779590	-0.1007280
H	-1.7976540	4.8456590	-0.0832820
H	-2.5413010	3.5272380	-1.0290700
H	-2.6593250	3.5240070	0.7500930
C	0.5586300	3.2590780	-1.3968260
H	0.7822150	4.3277340	-1.3741640
H	1.4870830	2.6884420	-1.3259370
H	0.0451420	3.0111600	-2.3275170

Pa_3 (protonated)

C	1.9596520	-1.5208420	1.6687710
P	2.5077040	-0.9088440	0.0514890
C	4.1231210	-0.1420070	0.3039990
N	1.5458320	0.1845000	-0.6087120
P	0.0010490	-0.0044090	-1.0663840
N	-0.6188770	-1.4352820	-0.6189710
P	-2.0459440	-1.7059280	0.0501960
C	-2.2741850	-0.9433470	1.6803460
C	2.7745400	-2.3926080	-0.9557630
C	-3.4616290	-1.1568570	-0.9402720
C	-2.2213850	-3.4876660	0.2843450
H	-4.4000360	-1.4024740	-0.4377600
H	-3.3871080	-0.0752330	-1.0738640
H	-3.4309410	-1.6447840	-1.9168300
H	-3.2259640	-1.2544370	2.1176880
H	-1.4553560	-1.2397410	2.3395670
H	-2.2643340	0.1412140	1.5569980
H	-1.4168330	-3.8453240	0.9302620
H	-3.1853810	-3.7156120	0.7442670
H	-2.1523190	-3.9867900	-0.6841480
H	1.7987710	-0.6771730	2.3433430
H	1.0188520	-2.0569280	1.5306740
H	2.7071230	-2.1934800	2.0964420
H	1.8120320	-2.8888750	-1.0994070
H	3.1808570	-2.1037160	-1.9273030
H	3.4683150	-3.0734770	-0.4568030
H	4.5154180	0.1930690	-0.6581750
H	4.0108310	0.7202270	0.9648400
H	4.8174410	-0.8559470	0.7521590
N	-0.9341410	1.2507610	-0.6390660
P	-0.4627290	2.6156370	0.0490470
C	0.2628230	2.4178510	1.7001800
H	1.1882110	1.8472350	1.6015610
H	-0.4311050	1.8709630	2.3422050
H	0.4769460	3.3935960	2.1428940
C	-1.9158150	3.6694550	0.2439770
H	-2.6519130	3.1553550	0.8656200
H	-1.6364070	4.6139940	0.7157100
H	-2.3513600	3.8667410	-0.7375030
C	0.7565890	3.5633040	-0.9008610
H	0.3518260	3.7857050	-1.8904640
H	1.0005050	4.4961860	-0.3872420
H	1.6568430	2.9539210	-1.0069080
H	0.0119180	-0.0145920	-2.4741530

Pb_3 (dication)

C	0.6909660	3.0150600	-2.1083730
C	1.5225860	1.8831970	-2.7176230
P	0.3302700	2.8067790	-0.3429650
C	1.8864740	2.8368200	0.5917740
C	1.6797420	2.7527820	2.1059050
N	-0.5330600	1.4368540	-0.0922830
P	-0.0833200	-0.0457170	0.0527270
N	1.4279530	-0.4073170	-0.0150010
P	2.2095980	-1.8403090	0.1118920
C	2.4297060	-2.2831280	1.8558200
C	3.1789860	-1.2168010	2.6579100
C	-0.7387710	4.1569630	0.2130380
C	-0.0956530	5.5440750	0.1478310
C	1.3380520	-3.2045510	-0.7090810
C	0.9824570	-2.9242980	-2.1704530
C	3.8228700	-1.5646790	-0.6618300
C	4.7882100	-2.7465950	-0.5525900
H	2.4129830	3.7545890	0.3109830
H	2.4822830	1.9921110	0.2335200
H	-1.0381060	3.9040680	1.2350850
H	-1.6408320	4.1039220	-0.4043770
H	1.2027870	3.9768210	-2.2155150
H	-0.2781710	3.1035850	-2.6096760
H	1.4249830	-2.4485210	2.2578760
H	2.9484630	-3.2468410	1.8858330
H	1.9776200	-4.0883920	-0.6176850
H	0.4363610	-3.3913800	-0.1180270
H	4.2339570	-0.6675170	-0.1883720
H	3.6240580	-1.3038980	-1.7059360
H	2.4887260	1.7723020	-2.2198100
H	0.9986740	0.9259090	-2.6659670
H	1.7089420	2.1031230	-3.7703680
H	0.7853530	5.6128900	0.7910800
H	0.1981720	5.8102130	-0.8703830
H	-0.8154500	6.2893510	0.4909560
H	1.1055940	1.8650730	2.3859610

Pb_3 (protonated)

C	1.7528560	3.0533480	-0.4530280
C	1.8868520	3.2005060	-1.9689660
P	0.0408990	2.7329210	0.0839110
C	0.0684040	2.7305470	1.9089750
C	1.0672090	1.7503150	2.5287730
N	-0.6418140	1.4400680	-0.5608500
P	-0.0602060	-0.0377560	-0.8566150
N	-1.0496690	-1.2215800	-0.3669330
P	-2.6190760	-1.1375650	-0.0768770
C	-3.1676610	-2.7885500	0.4481070
C	-2.9631860	-3.8594700	-0.6243820
C	-0.9804650	4.1573830	-0.4067540
C	-0.4344760	5.5337630	-0.0246250
C	-3.0990410	0.0235690	1.2476970
C	-2.2754450	-0.1367040	2.5246060
C	-3.5995270	-0.6559060	-1.5392690
C	-5.1189680	-0.7224430	-1.3733720
H	-0.9561890	2.4929800	2.2153520
H	0.2753800	3.7515410	2.2462110
H	-1.9678550	3.9866790	0.0351830
H	-1.1052700	4.0739400	-1.4908240
H	2.3399280	2.2005300	-0.1005690
H	2.1084990	3.9468270	0.0710420
H	-2.5906750	-3.0284850	1.3469960
H	-4.2182330	-2.7189450	0.7479510
H	-2.9675070	1.0269730	0.8309320
H	-4.1659530	-0.1205620	1.4473500
H	-3.2655450	-1.3035300	-2.3564450
H	-3.2744250	0.3598940	-1.7900000
H	1.5239560	2.3058030	-2.4831340
H	1.3249030	4.0601110	-2.3460320
H	2.9355730	3.3417780	-2.2399240
H	-0.3117150	5.6408510	1.0562790
H	0.5316570	5.7295020	-0.4979510
H	-1.1281900	6.3097200	-0.3559210
H	0.9088290	1.6916370	3.6081810

H	2.6501010	2.6940540	2.6022640	H	0.9641660	0.7481820	2.1047940
H	1.1569390	3.6313490	2.4924190	H	2.0971630	2.0738740	2.3561940
H	4.2075370	-1.0986340	2.3076120	H	-1.9192620	-3.8914180	-0.9463570
H	2.6784690	-0.2475380	2.5918800	H	-3.2308690	-4.8418200	-0.2290190
H	3.2149880	-1.5101720	3.7084980	H	-3.5872050	-3.6715980	-1.5029060
H	5.0195860	-2.9859800	0.4885260	H	-5.4702140	-0.0582700	-0.5794010
H	4.3902450	-3.6437960	-1.0333270	H	-5.6064200	-0.4149780	-2.3014220
H	5.7253530	-2.4919350	-1.0507030	H	-5.4551960	-1.7373420	-1.1433950
H	0.3083940	-2.0688300	-2.2611550	H	-2.5890610	0.5995440	3.2688460
H	1.8704580	-2.7324020	-2.7776250	H	-2.3938250	-1.1298690	2.9666770
H	0.4719130	-3.7935670	-2.5889950	H	-1.2139600	0.0190620	2.3187440
N	-1.1462800	-1.1680060	0.2333230	N	1.4381360	-0.2231170	-0.2824570
P	-2.7828200	-1.1081540	0.2842590	P	2.3077040	-1.5509240	-0.1067280
C	-3.3390360	-2.7722410	-0.1598140	C	3.8272670	-1.0552220	0.7590430
H	-2.9514280	-2.9717520	-1.1638140	H	4.2743220	-0.2601490	0.1538200
H	-2.8168440	-3.4513330	0.5214920	H	3.5014410	-0.5858340	1.6929520
C	-4.8547560	-2.9741060	-0.0935330	C	4.8276750	-2.1805580	1.0227750
H	-5.3857960	-2.3094330	-0.7794160	H	5.7033890	-1.7830620	1.5404700
H	-5.0918320	-4.0008400	-0.3780540	H	4.3983710	-2.9645820	1.6525330
H	-5.2409080	-2.8111870	0.9155700	H	5.1733960	-2.6407330	0.0929490
C	-3.4909620	0.1095930	-0.8623890	C	2.7845840	-2.3283560	-1.6868000
H	-3.2113150	1.0969270	-0.4835750	H	1.8527350	-2.6786400	-2.1448850
H	-4.5786780	0.0217590	-0.7727700	H	3.3844420	-3.2182350	-1.4694610
C	-3.0383870	-0.0613740	-2.3136570	C	3.5241580	-1.3722480	-2.6237960
H	-3.5167770	0.7008500	-2.9313970	H	3.7048820	-1.8537750	-3.5873290
H	-1.9572350	0.0617990	-2.4148280	H	2.9389330	-0.4653480	-2.7989010
H	-3.3135150	-1.0395960	-2.7154800	H	4.4931150	-1.0776250	-2.2107270
C	-3.3593470	-0.7153390	1.9597760	C	1.5163260	-2.8850890	0.8504520
H	-4.4480860	-0.6168810	1.8999770	H	0.6579670	-3.2098440	0.2546940
H	-3.1448340	-1.5899520	2.5816080	H	2.2232070	-3.7199680	0.9037300
C	-2.7254120	0.5453790	2.5531500	C	1.0553810	-2.4463850	2.2393050
H	-2.8835110	1.4227240	1.9213490	H	1.8890860	-2.1028610	2.8588680
H	-1.6504580	0.4161140	2.6999610	H	0.5776000	-3.2821580	2.7561790
H	-3.1717420	0.7466110	3.5286590	H	0.3255430	-1.6382860	2.1556340
				H	0.0110580	-0.1402380	-2.2640870

**Pc$_3$ (dication)**				**Pc$_3$ (protonated)**			
C	3.5491980	-2.3887710	-0.2385200	C	1.8984450	-1.8783620	1.1692350
C	5.0757090	-2.3684350	-0.3813090	C	2.8485900	-2.8210150	1.9094740
C	5.6252890	-3.7723640	-0.6241400	C	2.2406250	-3.2672260	3.2385270
P	2.8050780	-0.7659600	0.0366730	P	2.3934400	-1.3232610	-0.4934470
C	3.4866130	0.0062230	1.5261570	C	4.1188890	-0.7664080	-0.4067800
C	3.2834330	-0.8261520	2.7980910	C	4.3054960	0.4632730	0.4884480
C	3.7590300	-0.0704240	4.0364870	C	5.7602010	0.9236660	0.5324630
N	1.1914920	-1.0479100	0.1934910	N	1.5195450	-0.0960150	-1.0323540
P	0.0586930	0.0202880	0.3020550	P	-0.0823740	-0.0257380	-1.2552520
N	-1.4318570	-0.4208930	0.1928510	N	-0.6799570	1.4288990	-0.8777860
P	-2.0148090	-1.9202660	-0.1407920	P	0.0323510	2.5703470	-0.0119650
C	-3.8070190	-1.7184260	-0.2528670	C	1.4010270	3.3708530	-0.9098050
C	-4.5796010	-3.0225130	-0.4828830	C	2.1781540	4.4500780	-0.1503830
C	-6.0806270	-2.7617610	-0.5871630	C	3.3164860	5.0171950	-0.9968080
C	3.1286950	0.3300080	-1.3717370	C	2.3346670	-2.8075840	-1.5482050
C	2.5783980	-0.1993580	-2.7030230	C	2.6523410	-2.5331570	-3.0214550
C	2.5978260	0.8784700	-3.7835590	C	2.4759900	-3.7835850	-3.8801040
C	-1.3401400	-2.5545100	-1.7007790	C	-1.2229220	3.8228550	0.3815200
C	-1.5684310	-1.6244550	-2.8989630	C	-1.9329050	4.3999730	-0.8463570
C	-0.8269100	-2.1151290	-4.1396960	C	-3.0593170	5.3519990	-0.4503700
C	-1.5929050	-3.0909120	1.1749120	C	0.7419230	2.0454390	1.5894100
C	-2.1275810	-2.6803540	2.5525280	C	-0.1546280	1.1008770	2.3921890
C	-1.6307250	-3.6242230	3.6447030	C	0.4727710	0.7035220	3.7260740
H	3.0727300	-2.7980920	-1.1363560	H	0.9142800	-2.3367100	1.0265610
H	3.2365740	-3.0203010	0.6002340	H	1.7316460	-0.9681270	1.7547510
H	4.5493130	0.2007000	1.3435250	H	4.7297240	-1.6058670	-0.0559560
H	2.9895430	0.9796850	1.6102970	H	4.4361060	-0.5412970	-1.4306900
H	4.2090920	0.5009450	-1.4271600	H	1.3158230	-3.2002150	-1.4444290
H	2.6645910	1.2886400	-1.1107140	H	3.0170700	-3.5583770	-1.1335260
H	-0.2689840	-2.7053360	-1.5210810	H	-1.9484530	3.3311130	1.0403250
H	-1.7788800	-3.5425400	-1.8773300	H	-0.7428840	4.6151770	0.9670720
H	-1.9715620	-4.0753820	0.8787280	H	1.6969680	1.5586100	1.3624970
H	-0.4983880	-3.1537040	1.1806930	H	0.9671370	2.9505960	2.1652250
H	-3.9904590	-1.0006260	-1.0601190	H	0.9740140	3.7757390	-1.8344960
H	-4.1202350	-1.2310810	0.6770750	H	2.0596520	2.5444590	-1.2030760
H	3.1668320	-1.0645790	-3.0258080	H	1.9985720	-1.7346490	-3.3889440
H	1.5508140	-0.5544810	-2.5674210	H	3.6801520	-2.1656700	-3.1169480
H	2.2234340	-1.0811300	2.9043380	H	3.6721590	1.2732430	0.1112810
H	3.8301200	-1.7714100	2.7114130	H	3.9614260	0.2388370	1.5058340
H	5.3637570	-1.7145760	-1.2116780	H	3.0675020	-3.7005170	1.2938790
H	5.5287550	-1.9502020	0.5240730	H	3.8050230	-2.3188350	2.0931520
H	-2.6399880	-1.5525080	-3.1132820	H	-1.2085750	4.9294270	-1.4758100
H	-1.2320990	-0.6117080	-2.6500750	H	-2.3328360	3.5768070	-1.4467760
H	-4.2291090	-3.5088070	-1.3996790	H	1.5018600	5.2600650	0.1459090
H	-4.3857680	-3.7189900	0.3401690	H	2.5919570	4.0315920	0.7735820
H	-1.8130740	-1.6555390	2.7780630	H	-1.1266530	1.5725550	2.5783280
H	-3.2228960	-2.6788740	2.5370400	H	-0.3495680	0.2073980	1.7908790
H	-0.9999270	-1.4403990	-4.9816940	H	-3.5547680	5.7602760	-1.3353750
H	0.2518500	-2.1623980	-3.9592550	H	-3.8139590	4.8341280	0.1506630
H	-1.1639990	-3.1147650	-4.4292010	H	-2.6773570	6.1906770	0.1404380

H	-6.6236530	-3.6963800	-0.7470090	H	2.9340590	5.4646510	-1.9195940
H	-6.4613890	-2.2997690	0.3286550	H	3.8655180	5.7875930	-0.4487910
H	-6.3036480	-2.0918560	-1.4230260	H	4.0239060	4.2286610	-1.2732600
H	-2.0258450	-3.3273330	4.6193550	H	0.5971200	1.5739850	4.3773210
H	-1.9484730	-4.6527320	3.4487150	H	-0.1531580	-0.0257350	4.2486660
H	-0.5380080	-3.6126750	3.7035040	H	1.4625050	0.2558680	3.5832320
H	3.6137580	1.2456270	-3.9565520	H	2.7057410	-3.5733260	-4.9281570
H	2.2132930	0.4837160	-4.7273150	H	3.1379890	-4.5878110	-3.5435780
H	1.9742510	1.7298920	-3.4923380	H	1.4464250	-4.1517430	-3.8266710
H	4.8219520	0.1777760	3.9604960	H	6.1176700	1.1817100	-0.4693770
H	3.2016830	0.8628600	4.1625080	H	5.8695210	1.8064850	1.1680930
H	3.6156980	-0.6750620	4.9354230	H	6.4097330	0.1373470	0.9296510
H	5.3746370	-4.4392770	0.2062480	H	2.9224410	-3.9246000	3.7843480
H	5.2102270	-4.2015840	-1.5410120	H	2.0184090	-2.4042980	3.8751330
H	6.7131990	-3.7479550	-0.7231310	H	1.3041130	-3.8099960	3.0741510
N	0.4263030	1.5236070	0.4758760	N	-0.8272370	-1.2691720	-0.5307760
P	-0.5269150	2.8597540	0.4230380	P	-2.3465250	-1.5327630	-0.1136910
C	-1.2513130	3.1949690	2.0506310	C	-2.3726630	-2.7484770	1.2388330
H	-0.4375760	3.5443520	2.6955100	H	-3.4100690	-3.0675530	1.3906060
H	-1.9470720	4.0317310	1.9217780	H	-1.8207060	-3.6206980	0.8695080
C	-1.9615210	1.9886840	2.6795490	C	-1.7635850	-2.2463370	2.5518300
H	-1.2336900	1.1871850	2.8428540	H	-2.3308640	-1.3834530	2.9200040
H	-2.7173360	1.5932360	1.9931810	H	-0.7431820	-1.8950470	2.3694090
C	-2.6145920	2.3611110	4.0082000	C	-1.7410790	-3.3345010	3.6220000
H	-1.8718430	2.7358740	4.7190080	H	-1.2828290	-2.9635000	4.5428900
H	-3.3721260	3.1381640	3.8688820	H	-2.7537210	-3.6753140	3.8592410
H	-3.1006540	1.4895940	4.4537910	H	-1.1659170	-4.2028360	3.2844360
C	-1.8596530	2.6977120	-0.7976100	C	-3.3365430	-2.2769790	-1.4497850
H	-2.4936630	1.8719980	-0.4574860	H	-2.8190970	-3.1985940	-1.7399010
H	-2.4600830	3.6128310	-0.7515390	H	-4.3092630	-2.5703780	-1.0383980
C	-1.3552700	2.4350080	-2.2226450	C	-3.5140620	-1.3565140	-2.6615350
H	-0.8248880	3.3167270	-2.5976600	H	-4.0250870	-0.4360110	-2.3563640
H	-0.6249480	1.6167170	-2.2174460	H	-2.5295140	-1.0593660	-3.0399200
C	-2.5039610	2.0809870	-3.1629890	C	-4.3073180	-2.0326870	-3.7774640
H	-2.1307590	1.8813230	-4.1705760	H	-3.8010920	-2.9397010	-4.1224700
H	-3.2280440	2.8986530	-3.2226690	H	-4.4239840	-1.3635120	-4.6340820
H	-3.0313820	1.1878730	-2.8131920	H	-5.3060810	-2.3160650	-3.4306470
C	0.5709910	4.2166590	-0.0481350	C	-3.2438660	-0.0489700	0.4503970
H	1.3547680	4.2570750	0.7162740	H	-2.6738140	0.3524130	1.2941000
H	1.0537100	3.9108730	-0.9833230	H	-3.1508480	0.6966680	-0.3455340
C	-0.1211170	5.5760760	-0.2055410	C	-4.7073370	-0.2646210	0.8439150
H	-0.6133320	5.8553990	0.7320140	H	-4.7811220	-1.0330770	1.6220210
H	-0.9039850	5.5092140	-0.9687940	H	-5.2764060	-0.6325820	-0.0170850
C	0.8805020	6.6594410	-0.5995880	C	-5.3342290	1.0336550	1.3499520
H	0.3798510	7.6241430	-0.7120920	H	-4.8025130	1.4051260	2.2318810
H	1.6596250	6.7675570	0.1609570	H	-5.2925810	1.8114310	0.5806130
H	1.3655320	6.4154600	-1.5495940	H	-6.3817910	0.8835710	1.6240820
				H	-0.3182630	-0.1472870	-2.6394590
**Pd_3 (dication)**				**Pd_3 (protonated)**			
C	0.0739900	4.3607980	-0.5066570	C	-3.4179740	1.0115270	-1.1553380
P	-0.9128370	2.9165150	-0.0559320	P	-2.4694020	-0.5380800	-1.0140720
C	-2.1167360	2.5456050	-1.3615480	C	-3.0939520	-1.7035340	-2.2598770
C	-1.4859340	2.0835360	-2.6807040	C	-4.5646820	-2.1009300	-2.1155510
C	-2.5257010	1.8001740	-3.7691700	C	-5.0085840	-3.0591190	-3.2217370
C	-3.5045840	0.6793120	-3.4242420	C	-6.4725300	-3.4698540	-3.0854240
C	1.0914530	4.7515680	0.5712420	C	-3.1732840	1.7840530	-2.4517030
C	1.9966900	5.8928540	0.1068410	C	-3.8537650	3.1531660	-2.4348750
C	3.0149280	6.2913340	1.1720400	C	-3.6180490	3.9405530	-3.7212170
C	-1.8101350	3.2143020	1.4878280	C	-2.9362130	-1.1786310	0.6276170
C	-2.7485860	4.4274950	1.4524530	C	-2.4184480	-2.5897980	0.9203430
C	-3.4448530	4.6628850	2.7972180	C	-3.0457400	-3.2133980	2.1704950
C	-4.3679130	3.5232030	3.2254840	C	-2.6948620	-2.4968350	3.4718460
N	0.1632810	1.6912430	0.1341370	N	-0.8940290	-0.3953890	-1.2688650
P	-0.1152750	0.1721120	0.3328100	P	0.0820390	0.7211920	-0.6117100
N	1.0608550	-0.8452800	0.2577470	N	1.4995280	0.1296790	-0.1173040
P	2.6466760	-0.5727100	-0.0614520	P	2.5063450	-0.8468310	-0.8776890
C	3.4573160	0.1276160	1.3975730	C	3.0313910	-0.2253560	-2.5074310
C	3.3255850	-0.7712010	2.6332190	C	3.6723040	1.1653330	-2.4107950
C	3.8686060	-0.0975260	3.8931870	C	3.9065780	1.8221630	-3.7728460
C	3.7199360	-0.9849740	5.1264430	C	4.8775790	1.0547740	-4.6684860
N	-1.5735090	-0.3349790	0.5474720	N	-0.6525450	1.5497960	0.5638660
P	-2.1417210	-1.8773100	0.5227830	P	-0.0715600	2.1051730	1.9457200
C	-3.8782120	-1.7335530	0.0428030	C	0.9342100	0.9170170	2.8951140
C	-4.6099380	-3.0673330	-0.1370340	C	0.2670380	-0.4530540	3.0466610
C	-6.0656300	-2.8512380	-0.5542660	C	1.2239030	-1.5279870	3.5661650
C	-6.8135570	-4.1670570	-0.7514070	C	1.7453550	-1.2653860	4.9769240
C	-1.2548620	-2.9016700	-0.6867220	C	0.9624840	3.5986480	1.7695620
C	-1.2859820	-2.3080560	-2.0992190	C	2.0286800	3.4527740	0.6777340
C	-0.2562310	-2.9393560	-3.0333570	C	2.9744900	4.6496010	0.5999850
C	-0.2832060	-2.3028980	-4.4203440	C	3.9973440	4.4934510	-0.5238320
C	-2.0041210	-2.6512070	2.1552820	C	-1.5031220	2.5247620	2.9791010
C	-0.5944380	-2.5639420	2.7574800	C	-2.4726990	3.5160260	2.3211060
C	-0.4869960	-3.2539590	4.1203590	C	-3.8822940	3.4531680	2.9204520
C	-0.7009710	-4.7657370	4.0709990	C	-4.6491260	2.1876750	2.5353720
C	2.8660400	0.5544870	-1.4649140	C	1.8280480	-2.5151230	-1.1392910
C	2.0832530	0.1225740	-2.7124620	C	1.4731770	-3.2009780	0.1831080

C	2.0394640	1.2141480	-3.7841270
C	3.4089500	1.5649840	-4.3613550
C	3.3414790	-2.1967790	-0.4414460
C	4.8546910	-2.2015480	-0.6793540
C	5.3659620	-3.6075300	-0.9974210
C	6.8738240	-3.6360330	-1.2322210
H	-0.2246610	-2.9838240	-0.3259170
H	-1.6940320	-3.9039330	-0.6619060
H	-2.2894630	-2.4211460	-2.5262040
H	-1.0926190	-1.2280700	-2.0545890
H	0.7414800	-2.8245550	-2.5919630
H	-0.4411900	-4.0167300	-3.1084520
H	0.4738490	-2.7446230	-5.0732400
H	-1.2604810	-2.4372760	-4.8947770
H	-0.0894000	-1.2257580	-4.3620200
H	-4.3601390	-1.1156730	0.8083180
H	-3.8868120	-1.1512870	-0.8859650
H	-4.1055800	-3.6727180	-0.8995670
H	-4.5817000	-3.6423490	0.7960150
H	-6.5714130	-2.2460770	0.2074250
H	-6.0881990	-2.2678540	-1.4826770
H	-7.8503460	-3.9889210	-1.0489670
H	-6.3399010	-4.7749060	-1.5291480
H	-6.8247280	-4.7545110	0.1723910
H	-2.7346770	-2.1621460	2.8087280
H	-2.3236490	-3.6911910	2.0272940
H	0.1381940	-3.0027360	2.0702100
H	-0.3249930	-1.5090340	2.8743690
H	0.5104720	-3.0430030	4.5215020
H	-1.2034880	-2.7980310	4.8140800
H	-1.7225650	-5.0257190	3.7768980
H	-0.0160950	-5.2343810	3.3563600
H	-0.5214600	-5.2137410	5.0519240
H	-2.7772510	1.7766520	-0.9462440
H	-2.7183980	3.4487030	-1.5138200
H	-0.7897870	2.8454310	-3.0463840
H	-0.8927980	1.1769970	-2.5068690
H	-3.0784790	2.7219140	-3.9851100
H	-1.9860080	1.5388690	-4.6855900
H	-2.9691160	-0.2459280	-3.1883770
H	-4.1360590	0.9343930	-2.5672750
H	-4.1686060	0.4725460	-4.2673920
H	-0.6127880	5.1847920	-0.7272730
H	0.5838560	4.1044510	-1.4417430
H	0.5675210	5.0541560	1.4859150
H	1.7044600	3.8792530	0.8268560
H	2.5174560	5.5870000	-0.8085770
H	1.3790220	6.7587630	-0.1596570
H	3.6548750	7.1049600	0.8202520
H	2.5153520	6.6275140	2.0864530
H	3.6590250	5.4456000	1.4342660
H	-1.0546220	3.3233930	2.2737800
H	-2.3560550	2.2867040	1.6918190
H	-2.1799130	5.3252460	1.1864640
H	-3.5083960	4.2875440	0.6747090
H	-2.6857000	4.8376530	3.5690160
H	-4.0249880	5.5881190	2.7172300
H	-4.9088530	3.7865540	4.1382420
H	-5.1070690	3.3054120	2.4472170
H	-3.8139390	2.6017550	3.4298700
H	3.9396100	0.6278790	-1.6640180
H	2.5270200	1.5383230	-1.1207290
H	2.5227690	-0.7893900	-3.1338170
H	1.0554210	-0.1328010	-2.4278220
H	1.5748010	2.1117620	-3.3578040
H	1.3785280	0.8754620	-4.5897760
H	3.3135570	2.2959550	-5.1684380
H	3.9005750	0.6751460	-4.7681470
H	4.0697490	1.9971160	-3.6035870
H	2.8025210	-2.5690220	-1.3196590
H	3.0681780	-2.8495610	0.3947330
H	5.3750220	-1.8212830	0.2080340
H	5.1084770	-1.5300950	-1.5082490
H	4.8439610	-3.9864460	-1.8841340
H	5.1052900	-4.2788220	-0.1705440
H	7.4154970	-3.2868630	-0.3471510
H	7.2171890	-4.6489990	-1.4584770
H	7.1524680	-2.9907960	-2.0716700
H	2.9856260	1.1027460	1.5678980
H	4.5072080	0.3157780	1.1479170
H	3.8632130	-1.7122890	2.4658990
H	2.2716340	-1.0323740	2.7870110
H	4.9243090	0.1568660	3.7416980
H	3.3383480	0.8492270	4.0510470
H	4.2629480	-1.9271990	4.9998820
H	2.6676750	-1.2273100	5.3095870
H	4.1107170	-0.4889480	6.0188970
C	0.6301830	-4.4601610	-0.0086540
C	0.2850270	-5.1279960	1.3202140
C	3.9862550	-0.9884930	0.1633720
C	5.0979680	-1.8786350	-0.3951190
C	6.2914320	-1.9672040	0.5569670
C	7.4111300	-2.8484140	0.0092280
H	0.2974840	4.4387150	1.5406370
H	1.4199310	3.8020620	2.7450620
H	2.6116370	2.5379170	0.8364410
H	1.5292050	3.3291750	-0.2910570
H	2.3915440	5.5661810	0.4471560
H	3.4920370	4.7662900	1.5599860
H	3.4997870	4.4110870	-1.4965240
H	4.6782770	5.3480010	-0.5665230
H	4.5992220	3.5890040	-0.3825860
H	1.8770960	0.8110150	2.3496340
H	1.1490780	1.3765050	3.8660900
H	-0.5944660	-0.3750670	3.7208970
H	-0.1208410	-0.7675380	2.0718040
H	0.7092070	-2.4964010	3.5416750
H	2.0666550	-1.6164480	2.8690820
H	2.3769310	-2.0889090	5.3214300
H	2.3435930	-0.3499480	5.0209320
H	0.9161170	-1.1575810	5.6844110
H	-1.9985200	1.5661010	3.1699180
H	-1.1457230	2.8989480	3.9443360
H	-2.0723120	4.5298030	2.4250210
H	-2.5360280	3.3094630	1.2460970
H	-3.8195060	3.5349440	4.0125420
H	-4.4454410	4.3276190	2.5766970
H	-4.1429510	1.2778390	2.8750540
H	-5.6501020	2.1864200	2.9755880
H	-4.7600910	2.1176470	1.4479120
H	-2.9100180	-1.2367070	-3.2340350
H	-2.4438020	-2.5843120	-2.2121920
H	-4.7311500	-2.5778790	-1.1416430
H	-5.2003840	-1.2071820	-2.1388500
H	-4.8458120	-2.5826650	-4.1961590
H	-4.3696840	-3.9505050	-3.2011960
H	-6.7681260	-4.1533600	-3.8861810
H	-6.6509920	-3.9741560	-2.1299380
H	-7.1307330	-2.5958220	-3.1294040
H	-3.1058740	1.6102490	-0.2926710
H	-4.4812620	0.7779020	-1.0288200
H	-2.0949270	1.9180980	-2.6030840
H	-3.5392600	1.2051750	-3.3089310
H	-4.9304550	3.0192200	-2.2729930
H	-3.4801570	3.7250530	-1.5760870
H	-2.5486360	4.1085990	-3.8866180
H	-4.0086070	3.3993840	-4.5894120
H	-4.1098600	4.9166410	-3.6849820
H	-4.0310060	-1.1464130	0.6916220
H	-2.5502860	-0.4489230	1.3478480
H	-2.6271110	-3.2475300	0.0683650
H	-1.3279170	-2.5566240	1.0246400
H	-4.1352360	-3.2384060	2.0429390
H	-2.7194490	-4.2575550	2.2360300
H	-1.6148480	-2.5210110	3.6486940
H	-3.0073670	-1.4473780	3.4572910
H	-3.1832760	-2.9751450	4.3252410
H	2.5346760	-3.1102820	-1.7276030
H	0.9251610	-2.3668660	-1.7428760
H	2.3897220	-3.4573440	0.7297670
H	0.9226970	-2.4931020	0.8149020
H	1.1675840	-5.1667100	-0.6525990
H	-0.2914060	-4.1929810	-0.5408650
H	-0.2504150	-4.4353570	1.9794170
H	1.1908770	-5.4483330	1.8453610
H	-0.3488100	-6.0069020	1.1730690
H	3.6416300	-1.3569160	1.1361090
H	4.3444590	0.0337360	0.3289090
H	5.4390390	-1.4889020	-1.3623890
H	4.7124230	-2.8886850	-0.5824970
H	5.9504070	-2.3578980	1.5234810
H	6.6735290	-0.9568740	0.7475050
H	7.7856250	-2.4596760	-0.9435050
H	8.2526060	-2.8976770	0.7058000
H	7.0571780	-3.8701990	-0.1632720
H	2.1319100	-0.1935610	-3.1350540
H	3.7090010	-0.9570770	-2.9595170
H	4.6296370	1.0896630	-1.8792670
H	3.0300080	1.8138910	-1.8041510
H	2.9433760	1.9396540	-4.2847750
H	4.2922430	2.8337060	-3.6016870
H	4.4820040	0.0728280	-4.9462990
H	5.8355190	0.8978730	-4.1606450
H	5.0736140	1.6034150	-5.5938690

				H	0.3525930	1.6357500	-1.6573830
**Pe$_3$ (dication)**				**Pe$_3$ (protonated)**			
C	3.5083510	4.2924870	0.4461930	C	2.2167460	1.2667100	-2.4822640
C	3.0642390	2.8077780	0.3703650	C	1.5176400	2.5786950	-2.0296140
C	3.9960330	2.1240720	-0.6756500	C	2.4377150	3.7442850	-2.4922680
C	4.8260850	3.2674980	-1.2777280	C	3.5149690	3.0831550	-3.3689480
C	4.9259720	4.2834210	-0.1383280	C	3.6626200	1.6792860	-2.7761930
P	1.3025770	2.5849500	-0.0027070	P	1.1093200	2.5769650	-0.2449160
C	0.3631070	3.3982690	1.3308220	C	0.4515640	4.2345610	0.1280650
C	0.4322510	2.6394230	2.6694000	C	0.0428040	4.4006550	1.6094960
C	-0.6698470	3.3019660	3.5148590	C	-1.1362490	5.3760060	1.5568980
C	-1.7075320	3.8656400	2.5103850	C	-1.9194880	4.8909450	0.3344860
C	-1.1474100	3.5848710	1.1004840	C	-0.8363060	4.5396790	-0.7017600
N	1.0906570	0.9683210	-0.0375080	N	-0.0549400	1.5390510	0.0833850
P	-0.0492580	-0.0774730	0.0568310	P	-0.0591900	0.0002980	0.5526460
N	0.3232050	-1.5856070	0.1337190	N	1.3060340	-0.7655420	0.1693770
P	1.7729590	-2.3522070	0.1353860	P	1.5978120	-2.3250690	-0.0199970
C	1.3983750	-4.0794260	-0.2688880	C	1.4501080	-3.2828910	1.5319330
C	0.3822500	-4.7033250	0.7415870	C	-0.0046770	-3.2919090	2.1014610
C	-0.7818090	-5.2168330	-0.1225400	C	0.1128720	-2.7587070	3.5415510
C	-0.1512910	-5.4781070	-1.4916020	C	1.5619360	-3.0504390	3.9408310
C	0.7818710	-4.2773200	-1.6753650	C	2.3403110	-2.7207290	2.6638250
C	2.4864040	-2.3091980	1.8101170	N	-1.3693160	-0.7882630	0.0335460
C	3.7010590	-3.2683710	1.9826690	P	-2.8821350	-0.2800110	-0.0608220
C	4.7398760	-2.4465620	2.7607150	C	-3.0572990	1.2467580	-1.0429190
C	4.5093360	-1.0142580	2.2743660	C	-4.4881600	1.8451850	-1.1714340
C	2.9822620	-0.8965800	2.2380280	C	-4.6905690	2.0781060	-2.6811130
C	2.9193370	-1.5927600	-1.0629550	C	-3.2706590	2.2132360	-3.2394090
C	2.2476300	-1.1841820	-2.4137900	C	-2.4988960	1.1318200	-2.4777650
C	3.1692510	-1.7422920	-3.5121990	C	-3.6187490	0.0969270	1.5718590
C	4.5293160	-1.8939700	-2.8287180	C	-2.9975920	1.3697470	2.2331010
C	4.1613800	-2.4335250	-1.4434080	C	-2.6243640	0.9517570	3.6709680
C	0.9185400	3.2806440	-1.6430560	C	-3.4587620	-0.3022470	3.9481670
C	0.6469820	4.8164650	-1.6966530	C	-3.4352400	-1.0351260	2.6049680
C	-0.6633720	4.9708080	-2.4977020	C	-3.8078840	-1.6322170	-0.8626580
C	-0.7924230	3.6704720	-3.2952580	C	-5.3473870	-1.5108040	-0.9026940
C	-0.3027300	2.6059880	-2.3126360	C	-5.8142960	-2.9668340	-0.9958480
H	-1.5483750	-4.4382130	-0.2140590	C	-4.8799170	-3.6882340	-0.0212100
H	-1.2601690	-6.0981980	0.3100430	C	-3.5037780	-3.0364850	-0.2505990
H	0.4309350	-6.4060940	-1.4683270	C	3.3090640	-2.4466760	-0.6612080
H	-0.8861090	-5.5642080	-2.2960220	C	4.2380440	-1.3282370	-0.1355830
H	1.5460840	-4.4263290	-2.4414390	C	5.6365440	-1.9072640	-0.3516190
H	0.1833910	-3.4072870	-1.9636940	C	5.4779610	-3.3663270	0.0838490
H	0.0332640	-3.9967850	1.4994130	C	4.0795640	-3.7807920	-0.4242780
H	0.8689320	-5.5247330	1.2720730	C	0.4845220	-3.1286400	-1.2233880
H	3.1984360	-1.0939580	-4.3905770	C	0.3570810	-2.3132440	-2.5367380
H	2.1952770	-0.0936250	-2.4615240	C	0.2711910	-3.3767400	-3.6369970
H	1.2228440	-1.5469570	-2.5212260	C	1.2191730	-4.4708410	-3.1397690
H	4.9687080	-0.2586810	2.9157770	C	0.8977250	-4.5697920	-1.6412620
H	4.9686980	-2.3330720	-0.7148830	C	2.6484190	2.2650910	0.7011460
H	4.9266900	-0.8898180	1.2671830	C	3.7052640	3.3905310	0.7478420
H	5.0138810	-0.9157580	-2.7270400	C	4.4878370	3.0808850	2.0273920
H	2.6281170	-0.0920230	1.5895920	C	3.3872650	2.6960060	3.0187460
H	3.9142850	-3.4978900	-1.5258500	C	2.3896000	1.8672030	2.1886490
H	2.5993020	-0.6865890	3.2406020	H	6.4068180	-1.3731220	0.2107990
H	5.7583710	-2.8033700	2.5916680	H	5.8999810	-1.8551570	-1.4148780
H	3.4082680	-4.1879070	2.4928620	H	6.2670900	-4.0193620	-0.2965700
H	4.5363350	-2.5072730	3.8353380	H	5.5058160	-3.4219420	1.1778800
H	4.1250760	-3.5629830	1.0179340	H	4.1489440	-4.3350590	-1.3640660
H	-1.1118000	2.5885150	4.2148850	H	3.5909900	-4.4423700	0.2941420
H	-0.2414230	4.1121460	4.1103090	H	4.0693880	-1.1436080	0.9307790
H	0.1986220	1.5806890	2.5088480	H	4.0694740	-0.3867260	-0.6610600
H	3.4540580	1.5548080	-1.4367830	H	-0.6214730	-3.2114870	4.2126940
H	4.6462600	1.4145560	-0.1565570	H	-0.3840280	-4.3171160	2.0958880
H	5.6391740	3.9318620	0.6156680	H	-0.7005090	-2.6923220	1.5077970
H	5.7973380	2.9254440	-1.6419050	H	1.0969880	-5.4250100	-3.6586410
H	4.2934620	3.7167370	-2.1243890	H	3.3511480	-3.1372980	2.6431670
H	5.2456470	5.2743920	-0.4684920	H	2.2585580	-4.1480990	-3.2807600
H	3.4585370	4.6768170	1.4669610	H	1.6784410	-4.1124740	4.1871970
H	2.8738340	4.9331960	-0.1756920	H	1.7320930	-4.9800760	-1.0663060
H	-2.6927770	3.4084780	2.6371370	H	2.4234390	-1.6315200	2.5672360
H	-1.5689800	2.6588130	0.7028580	H	0.0493390	-5.2417900	-1.4822910
H	-1.8361890	4.9403270	2.6593510	H	0.5387590	-2.9866440	-4.6225630
H	0.5640390	5.2812620	-0.7110560	H	-0.5028470	-1.6417380	-2.4929830
H	1.4790030	5.3077710	-2.2061740	H	-0.7498460	-3.7723810	-3.6988960
H	-0.6535350	5.8625730	-3.1280020	H	1.2405060	-1.6859060	-2.7059330
H	-1.5140270	5.0649460	-1.8137460	H	-6.8725000	-3.0867490	-0.7500300
H	-1.8119850	3.4795820	-3.6393580	H	-5.6605520	-3.3421090	-2.0145220
H	-0.0336360	1.6602540	-2.7887490	H	-5.7374620	-1.0725170	0.0229750
H	-1.0876860	2.3936860	-1.5806590	H	-2.4563450	-1.5104640	2.4708220
H	-1.3698010	4.3874690	0.3943690	H	-4.2008080	-1.8103910	2.5146130
H	-0.1384450	3.6967000	-4.1740840	H	-2.7993920	1.7528050	4.3932770
H	2.8119700	-2.7250530	-3.8387760	H	-4.4880240	-0.0247430	4.2043930
H	5.2126290	-2.5553720	-3.3661280	H	-3.0609650	-0.9093980	4.7657770
H	1.4174180	2.6885700	3.1389570	H	-1.5590010	0.6966490	3.7178300
H	1.8054690	3.0535490	-2.2454630	H	-3.7364760	2.1755920	2.2311290

H	3.1946880	2.3311850	1.3473720
H	0.8375320	4.3802090	1.4576290
H	1.6602650	-2.6238320	2.4549330
H	2.3479360	-4.6198360	-0.2084740
H	3.2518060	-0.6796480	-0.5584010
N	-1.5291520	0.3727350	0.0835850
P	-3.0352200	-0.2131150	0.2984850
C	-4.1413230	1.2193250	0.1893500
H	-3.8936850	1.8423340	1.0544560
C	-3.9484900	2.0395900	-1.1136210
H	-3.2553020	2.8696550	-0.9647800
H	-3.5411350	1.4212540	-1.9227740
C	-5.3655910	2.4976670	-1.4764360
H	-5.4537100	2.7812020	-2.5277430
H	-5.6414150	3.3667530	-0.8688710
C	-6.2366530	1.2983830	-1.0983190
H	-6.1408510	0.5159210	-1.8601220
H	-7.2975980	1.5438610	-1.0142100
C	-5.6474080	0.8158970	0.2375140
H	-6.1220890	1.3277930	1.0775060
H	-5.8132380	-0.2548560	0.3925180
C	-3.1951890	-0.9172930	1.9675260
H	-4.2383440	-1.2364260	2.0563020
C	-2.8663610	0.1457270	3.0541150
H	-2.2116590	0.9268240	2.6484580
H	-3.7672920	0.6367380	3.4269670
C	-2.1022780	-0.6332080	4.1305490
H	-2.8058720	-1.1691760	4.7765320
H	-1.4961660	0.0204690	4.7627670
C	-1.2718760	-1.6313160	3.3224960
H	-0.8758220	-2.4539460	3.9227770
H	-0.4147500	-1.1187250	2.8674600
C	-2.2374760	-2.1199800	2.2335140
H	-2.8275960	-2.9628700	2.6023310
H	-1.7045870	-2.4604300	1.3420170
C	-3.5247640	-1.3993530	-1.0103070
H	-4.1839590	-0.8256460	-1.6734080
C	-2.3649560	-1.9489820	-1.8539470
H	-1.5978390	-2.3681960	-1.1966920
H	-1.8916310	-1.1855820	-2.4772760
C	-3.0434230	-3.0571900	-2.6636420
H	-2.3236230	-3.7919260	-3.0331170
H	-3.5372690	-2.6190030	-3.5364810
C	-4.0919640	-3.6761590	-1.7106620
H	-3.7523840	-4.6394150	-1.3212420
H	-5.0355620	-3.8561950	-2.2300030
C	-4.2760700	-2.6673550	-0.5470040
H	-3.8283080	-3.0696790	0.3651430
H	-5.3268080	-2.4633220	-0.3291140

H	-2.1205100	1.7460670	1.6978050
H	-5.2156820	-3.5032230	1.0059530
H	-2.9162260	-2.9835690	0.6680550
H	-4.8565350	-4.7708960	-0.1676310
H	-5.2728090	1.2087580	-0.7552890
H	-4.5311580	2.7909740	-0.6234510
H	-5.3228920	2.9448400	-2.8887560
H	-5.1731250	1.2023040	-3.1313690
H	-3.2198380	2.0879650	-4.3240990
H	-1.4138660	1.2602720	-2.4877030
H	-2.7190510	0.1538260	-2.9220930
H	-2.9110010	-3.6154300	-0.9640240
H	-2.8652590	3.2035400	-2.9967400
H	-0.0563830	-1.6747350	3.5539280
H	1.8980690	-2.4669390	4.8019250
H	-5.6913180	-0.8907030	-1.7343220
H	-2.4130090	1.9490700	-0.5056150
H	-4.6886200	0.2684650	1.4155140
H	-3.4339370	-1.6233820	-1.8922030
H	-0.4874480	-3.1376520	-0.7230800
H	3.1986140	-2.2983960	-1.7421780
H	1.7462030	-4.3123620	1.3094450
H	3.0799290	1.3925440	0.2001170
H	4.3362240	3.4096770	-0.1439260
H	3.2336940	4.3757770	0.8437250
H	5.0984400	3.9228680	2.3634430
H	5.1554130	2.2283860	1.8534230
H	2.9004100	3.6047640	3.3909960
H	3.7620260	2.1468570	3.8859980
H	2.5706390	0.7944100	2.2969650
H	1.3593740	2.0416910	2.5069130
H	1.2260850	4.9654070	-0.1233760
H	-0.3116640	3.4435480	2.0090130
H	0.8732350	4.7424400	2.2332290
H	-0.7746000	6.3981790	1.3941820
H	-1.7276780	5.3705570	2.4763290
H	-2.6339910	5.6262770	-0.0437570
H	-2.4829250	3.9892180	0.6027790
H	-0.6449520	5.3822660	-1.3711460
H	-1.1490730	3.6983120	-1.3267480
H	0.5374800	2.6585050	-2.5095960
H	2.9139580	4.2440500	-1.6440950
H	1.8692650	4.5099150	-3.0251200
H	4.4492840	3.6505010	-3.3794800
H	3.1601710	3.0065400	-4.4029590
H	4.2432720	1.7269760	-1.8459290
H	4.1721080	0.9787590	-3.4431500
H	2.1285010	0.4594460	-1.7487450
H	1.7410320	0.9146010	-3.4033450
H	-0.1265590	0.0194590	1.9673080

Pf$_3$ (dication)

C	-1.0288550	4.4047020	1.3807740
C	-0.3320100	4.2784630	0.0203440
C	-1.3612690	4.2436220	-1.1184950
P	0.7337040	2.7933500	-0.0502950
C	1.7235410	2.7805660	-1.6140450
C	2.0831910	4.1929870	-2.1006790
N	-0.3084450	1.5282250	0.0180760
P	-0.0038410	0.0014090	0.0515640
N	1.4761410	-0.4733710	0.0242760
P	2.2091610	-1.9398660	0.0969900
C	1.2029010	-3.2417540	-0.7221000
C	1.1130450	-3.0351380	-2.2387390
C	1.9420480	2.7092060	1.3381180
C	2.7114780	4.0241700	1.5169150
C	3.8573500	-1.6613400	-0.6521340
C	4.6897430	-2.9444390	-0.7513230
C	2.4239620	-2.4031550	1.8588250
C	1.0577010	-2.5428980	2.5436630
C	3.7917440	-0.9055050	-1.9844000
C	3.3168490	-1.4014980	2.6008660
C	1.6341420	-4.6697470	-0.3634830
C	1.3341250	2.2212430	2.6580000
C	1.0586760	1.9629660	-2.7289080
H	2.6402400	1.9374200	0.9897070
H	2.6505650	2.2710370	-1.3242190
H	4.3286410	-0.9917140	0.0770520
H	0.2059340	-3.0644320	-0.3001870
H	0.3371230	5.1341510	-0.1236280
H	4.3057340	-3.6078420	-1.5308610
H	5.7141530	-2.6779770	-1.0220980
H	4.7314340	-3.4980970	0.1905680
H	3.4002210	-1.5274790	-2.7914080
H	3.1761640	-0.0073550	-1.9061290
H	4.8046140	-0.6006120	-2.2592090
H	2.9177160	-3.3815650	1.8459210

Pf$_3$ (protonated)

C	-3.1408560	3.3150970	0.8321060
C	-2.1425140	3.6441790	-0.2849360
C	-2.7172630	3.2510390	-1.6506140
P	-0.5422880	2.8049270	0.0388550
C	0.6820580	3.2797330	-1.2601740
C	0.5874710	4.7527210	-1.6769160
N	-0.8514420	1.2385520	0.0789080
P	0.0311430	-0.0383950	0.5092850
N	-0.6585090	-1.4307520	0.0671710
P	-2.2048350	-1.8377450	0.0192040
C	-3.0054710	-1.8293510	1.6831350
C	-2.0967850	-2.5436700	2.6917020
C	0.0883890	3.4322190	1.6643690
C	1.5604540	3.0516420	1.8852000
C	-3.2007750	-0.7371460	-1.0693950
C	-2.5088110	-0.5427430	-2.4224650
C	-2.3342450	-3.5457690	-0.6836340
C	-3.5102630	-4.3571520	-0.1231980
C	-4.6543170	-1.1936160	-1.2257050
C	-1.0184570	-4.3201690	-0.5684100
C	-3.3488010	-0.4099080	2.1531150
C	-0.1308070	4.9243550	1.9403700
C	0.6410440	2.3354240	-2.4670610
H	-0.5222780	2.8572870	2.3724810
H	1.6452620	3.1255600	-0.7617160
H	-3.1726740	0.2255320	-0.5471360
H	-3.9391980	-2.3935560	1.5889260
H	-1.9349620	4.7204040	-0.2815130
H	-2.4084550	-1.4839220	-2.9733500
H	-3.1071420	0.1339690	-3.0406560
H	-1.5178510	-0.1026450	-2.2923330
H	-4.7229240	-2.1210150	-1.8030230
H	-5.1535920	-1.3495040	-0.2647610
H	-5.2163220	-0.4283990	-1.7687190
H	-2.5245120	-3.3747610	-1.7503690

H	0.5531180	-1.5741440	2.6089200	H	-4.4653680	-3.8328030	-0.2024060
H	0.3917050	-3.2436410	2.0341620	H	-3.5986160	-5.2892080	-0.6880700
H	1.2068890	-2.9045740	3.5634470	H	-3.3503540	-4.6220290	0.9260390
H	3.3353060	-1.6637850	3.6614210	H	-1.1482220	-5.3145020	-1.0056950
H	4.3475980	-1.4146810	2.2408160	H	-0.2172740	-3.8101490	-1.1029650
H	2.9264260	-0.3832670	2.5137170	H	-0.7068580	-4.4450670	0.4727010
H	2.0514210	-3.2972640	-2.7339970	H	-1.1635140	-1.9905410	2.8311440
H	0.3364040	-3.6931010	-2.6369990	H	-2.6025330	-2.6085800	3.6590460
H	0.8515560	-2.0082550	-2.5095470	H	-1.8404410	-3.5594870	2.3782560
H	0.9520860	-5.3669270	-0.8568870	H	-4.1299070	0.0459810	1.5391620
H	2.6450040	-4.8975930	-0.7079770	H	-3.7198810	-0.4516770	3.1812330
H	1.5815340	-4.8659140	0.7095730	H	-2.4764820	0.2503230	2.1354370
H	2.1476290	2.0441330	3.3664300	H	0.4775190	5.5501320	1.2821940
H	0.6589810	2.9545710	3.1016860	H	-1.1742500	5.2303400	1.8376420
H	0.7873670	1.2826820	2.5451040	H	0.1763340	5.1416670	2.9676550
H	3.2681370	4.3079180	0.6217550	H	1.8068090	3.1617370	2.9450750
H	2.0486140	4.8490360	1.7936590	H	1.7850330	2.0255090	1.5821220
H	3.4369010	3.8983480	2.3243370	H	2.2227000	3.7176750	1.3226100
H	-0.3285350	4.6325530	2.1862640	H	-4.0791640	3.8460990	0.6495940
H	-1.7507090	5.2237010	1.3311980	H	-3.3499900	2.2423210	0.8484190
H	-1.5736790	3.4899340	1.6329280	H	-2.7810870	3.6037240	1.8232940
H	-2.0315400	3.3873770	-1.0080110	H	-2.8086090	2.1649450	-1.7338630
H	-1.9632210	5.1543320	-1.0755500	H	-3.7138820	3.6872040	-1.7633390
H	-0.9033140	4.1961590	-2.1083750	H	-2.1043260	3.6075160	-2.4811730
H	1.6893420	2.0135930	-3.6200760	H	1.4337190	2.6202260	-3.1663180
H	0.9600330	0.9078780	-2.4646840	H	0.8094570	1.3024590	-2.1568250
H	0.0702430	2.3463880	-2.9931920	H	-0.3112310	2.3900480	-3.0013190
H	1.2090310	4.7188980	-2.4923560	H	0.6401780	5.4372180	-0.8275490
H	2.5347060	4.8137750	-1.3245890	H	1.4214930	4.9906200	-2.3431230
H	2.8077030	4.1046860	-2.9136200	H	-0.3379210	4.9600610	-2.2217470
N	-1.1653200	-1.0283680	0.0881110	N	1.5569070	0.0768240	0.0008290
P	-2.8012620	-0.8921930	0.0913390	P	2.7350210	-0.9975150	-0.0817920
C	-3.3396800	0.5661330	-0.8851500	C	3.1913710	-1.7041300	1.5610660
H	-2.8398880	1.3790670	-0.3444200	H	4.1640670	-2.1924070	1.4378160
C	-2.8007740	0.5708520	-2.3204890	C	3.3340910	-0.5733280	2.5877050
H	-3.3577810	-0.1166370	-2.9600240	H	3.6733010	-0.9857420	3.5419550
H	-1.7407720	0.3114470	-2.3754490	H	2.3744430	-0.0755080	2.7535050
H	-2.9151640	1.5770010	-2.7324930	H	4.0559660	0.1865720	2.2756810
C	-4.8530060	0.8047150	-0.8401970	C	2.1767190	-2.7572810	2.0287440
H	-5.3979280	0.0425990	-1.4018230	H	1.1512380	-2.3755470	2.0084480
H	-5.2475490	0.8364040	0.1781740	H	2.2093260	-3.6604270	1.4142090
H	-5.0641390	1.7712750	-1.3047550	H	2.4081160	-3.0478660	3.0575240
C	-3.3837800	-0.6728190	1.8169600	C	4.2143540	-0.1571670	-0.8060210
H	-4.4781460	-0.6774890	1.7589000	H	4.1303850	-0.3653830	-1.8802400
C	-2.9292500	-1.8452100	2.6955790	C	4.1553000	1.3618800	-0.6213070
H	-3.2662620	-1.6705830	3.7200580	H	4.1926120	1.6436740	0.4350990
H	-3.3483590	-2.7995240	2.3679240	H	5.0122670	1.8187920	-1.1248520
H	-1.8393260	-1.9302690	2.7070260	H	3.2387430	1.7713230	-1.0463010
C	-2.9300050	0.6757640	2.3911590	C	5.5512120	-0.7286630	-0.3155990
H	-3.3367160	1.5247230	1.8365180	H	6.3640640	-0.2740630	-0.8884980
H	-3.2824870	0.7547860	3.4221560	H	5.7216740	-0.4955290	0.7396310
H	-1.8393560	0.7623270	2.4065950	H	5.6239010	-1.8112590	-0.4422190
C	-3.3658750	-2.5239920	-0.5162700	C	2.3202900	-2.4185240	-1.1710040
H	-2.8370410	-3.1979000	0.1697890	H	1.5311200	-2.9292370	-0.6103110
C	-4.8728480	-2.7583110	-0.3712240	C	1.7078370	-1.9413560	-2.4910380
H	-5.4464130	-2.1298540	-1.0561080	H	1.4151370	-2.8113470	-3.0872450
H	-5.0878240	-3.8000970	-0.6216920	H	0.8183520	-1.3345580	-2.3105310
H	-5.2327750	-2.5832100	0.6455760	H	2.4188590	-1.3585660	-3.0863130
C	-2.8667450	-2.8202000	-1.9354840	C	3.4890700	-3.3831180	-1.3916570
H	-3.4615050	-2.2936120	-2.6850230	H	3.1316990	-4.2666420	-1.9282760
H	-2.9651020	-3.8917790	-2.1260960	H	3.9360280	-3.7256080	-0.4536020
H	-1.8162040	-2.5492160	-2.0656120	H	4.2751080	-2.9258960	-2.0008580
				H	0.0788510	-0.0381210	1.9284240
**Pg$_3$ (dication)**				**Pg$_3$ (protonated)**			
C	3.6435830	-2.9573150	0.5274230	C	-4.8700770	-1.4623360	1.0078020
N	2.8329780	-1.7789200	0.8252560	N	-3.7396350	-0.6804640	0.5231300
P	2.9095630	-0.3641350	-0.0060490	C	-4.0905190	0.6726270	0.1103840
N	3.4033410	-0.7831590	-1.5266370	P	-2.4089560	-1.4646460	-0.1121110
C	2.4894420	-1.5876930	-2.3406630	N	-1.8639570	-2.3733590	1.1696410
C	2.1586740	-1.8218090	2.1209420	C	-1.8876950	-1.8621180	2.5343340
N	3.9568060	0.6937320	0.6885140	N	-1.5308950	-0.3326830	-0.7594260
C	3.8307110	2.1424930	0.5482420	P	0.0130360	-0.0709850	-1.1057800
N	1.4939750	0.4279370	0.0009890	N	0.5013860	1.4070190	-0.6809140
P	0.0129050	-0.0015890	-0.1596450	P	-0.0920030	2.7275110	-0.0668890
N	-1.0928290	1.0769050	-0.3065070	N	-1.5167240	3.1569040	-0.8095530
P	-1.1369020	2.6743710	-0.0266000	C	-1.7922780	2.8532930	-2.2068490
N	0.1744880	3.4496430	-0.6413600	N	-2.7589900	-2.6374400	-1.2506900
C	0.6642450	3.1020870	-1.9737000	C	-2.8410700	-2.2735180	-2.6603090
C	5.2466280	0.2299500	1.1920950	N	-0.3639850	2.6914930	1.5731500
N	-0.3595300	-1.5072750	-0.2097080	C	-1.1884470	1.6240040	2.1182970
P	-1.7613750	-2.3134360	-0.0748150	N	1.0127020	3.9815040	-0.2215290
N	-2.9217950	-1.6890780	-1.0553020	C	2.3517090	3.7060900	0.2997590
C	-4.3570760	-1.7712190	-0.7937550	C	-0.1802130	3.8166250	2.4805270
N	-1.3634530	-3.8539700	-0.4846940	C	1.0426460	4.6686460	-1.5104150
C	-2.4110750	-4.8186440	-0.8067280	C	-2.4150450	4.1428450	-0.2276010
N	-2.4178070	-2.2637200	1.4424380	C	-3.5225250	-3.8366680	-0.9242540

C	-2.0289840	-3.2597240	2.4398930	C	-0.8798490	-3.4354080	0.9793730
C	-0.0467740	-4.4327420	-0.2296860	H	-1.1458570	4.1634510	2.8717480
C	4.2383250	0.1323510	-2.3032820	H	1.6513280	5.5711640	-1.4105210
C	-2.7510280	-0.9579720	2.0147470	H	0.4408290	3.5099380	3.3302810
C	-2.5810460	-1.2932070	-2.4205470	H	1.4750900	4.0471330	-2.3081240
N	-1.1366240	3.0521010	1.5829350	H	0.0368760	4.9724190	-1.8038810
C	-2.3987840	3.1403890	2.3152130	H	-2.2126440	1.9707320	2.3122660
N	-2.5328010	3.1597840	-0.7440350	H	-1.2399580	0.7890300	1.4196750
C	-2.7697890	4.5779870	-0.9986580	H	-0.7593700	1.2663140	3.0606350
C	-0.0086820	2.5983430	2.3993200	H	2.8868880	4.6511680	0.4280230
C	-3.7121970	2.3054970	-0.8565920	H	2.2826660	3.2177760	1.2741410
C	0.7294700	4.6884380	-0.1004970	H	2.9293600	3.0578720	-0.3736510
H	-2.8300540	-3.3420890	3.1784790	H	-2.2812070	5.1342470	-0.6801590
H	-4.8036470	-0.7745730	-0.8704230	H	-2.2515440	4.2252020	0.8467910
H	-1.8950870	-4.2365250	1.9762810	H	-3.4514870	3.8273660	-0.3890990
H	-4.8418590	-2.4253850	-1.5272230	H	-1.8189440	3.7698740	-2.8098140
H	-4.5398970	-2.1638000	0.2053010	H	-2.7606960	2.3484770	-2.2948680
H	-1.9050090	-0.5465270	2.5813160	H	-2.6126680	-1.0544280	2.6270870
H	-3.0210590	-0.2524820	1.2273630	H	-0.9005900	-1.4898570	2.8380320
H	-3.6030460	-1.0683910	2.6903410	H	-2.1704880	-2.6693150	3.2186190
H	-2.9674940	-0.2890350	-2.6184090	H	-1.1457390	-4.2919160	1.6091290
H	-1.5001760	-1.2790780	-2.5630340	H	0.1252770	-3.0872230	1.2430050
H	-3.0162410	-1.9933330	-3.1415000	H	-0.8620890	-3.7555520	-0.0614320
H	-3.3317250	-4.3065260	-1.0883940	H	-4.7820600	0.6735190	-0.7438000
H	-2.6233040	-5.4821900	0.0397960	H	-4.5807880	1.1816300	0.9475420
H	-2.0867250	-5.4268970	-1.6555480	H	-3.1889430	1.2223580	-0.1590420
H	0.6717270	-3.6379860	-0.0314860	H	-4.5323510	-2.4231120	1.3998640
H	0.2774430	-4.9892100	-1.1139580	H	-2.2215770	-1.4000550	-2.8601470
H	5.4602500	0.7325530	2.1393720	H	-5.6132490	-1.6436420	0.2196470
H	6.0579260	0.4488820	0.4879930	H	-2.4785330	-3.1079140	-3.2697240
H	5.2226660	-0.8448390	1.3753270	H	-5.3592750	-0.9210620	1.8239260
H	4.0007360	2.6122840	1.5215670	H	-4.5899540	-3.7119480	-1.1489010
H	2.8258930	2.3928640	0.2088030	H	-3.4125760	-4.0864920	0.1309900
H	4.5622650	2.5382810	-0.1659110	H	-3.1431890	-4.6768680	-1.5155320
H	2.8876640	-1.9538900	2.9273440	H	-3.8739930	-2.0513270	-2.9609150
H	1.6073880	-0.8988650	2.3052830	H	-1.0232070	2.1974870	-2.6136070
H	1.4503470	-2.6558230	2.1389650	H	0.3048370	4.6439620	1.9651770
H	2.9989140	-3.8391900	0.4567820	N	0.9831530	-1.2356190	-0.5701520
H	3.6414530	0.9016000	-2.8087470	P	2.4894730	-1.3055490	-0.1053610
H	4.3780030	-3.1283810	1.3227010	N	2.7558480	-2.8669900	0.3988310
H	4.9755050	0.6160930	-1.6631190	C	2.2967280	-3.9928610	-0.4020890
H	4.1687430	-2.8260460	-0.4172650	H	3.1167610	-4.4484330	-0.9737900
H	1.8945560	-2.2489190	-1.7078610	H	1.8695780	-4.7588330	0.2546400
H	3.0714390	-2.2015420	-3.0324920	H	1.5207680	-3.6601590	-1.0915890
H	1.8087790	-0.9503090	-2.9203570	C	3.7160890	-3.2038010	1.4373220
H	4.7746590	-0.4462600	-3.0592140	H	3.2879680	-3.9728410	2.0890980
H	-0.0727330	-5.1174770	0.6264160	H	4.6568730	-3.5917180	1.0235540
H	-1.1034110	-2.9780270	2.9571460	H	3.9404610	-2.3288110	2.0476110
H	-0.2081700	1.6131770	2.8415240	N	2.8151910	-0.2767410	1.1531600
H	0.8985900	2.5323770	1.7962580	C	4.1126890	0.3274390	1.4026820
H	0.1618660	3.3152380	3.2064860	H	4.5352990	-0.0258360	2.3521350
H	-2.7357810	2.1578710	2.6685240	H	4.8135420	0.0818380	0.6052650
H	-2.2529740	3.7907560	3.1811420	H	4.0168050	1.4182030	1.4556510
H	-3.1757390	3.5789160	1.6897420	C	1.8319480	-0.0534500	2.2004230
H	-3.4276000	1.2641560	-0.7088870	H	2.1652090	-0.4835060	3.1536420
H	-4.4757240	2.5805950	-0.1193840	H	0.8833680	-0.5100570	1.9180680
H	-4.1375130	2.4149860	-1.8585900	H	1.6610770	1.0211690	2.3324390
H	-3.4282640	5.0199630	-0.2417410	N	3.6048490	-0.9575830	-1.2913360
H	-3.2394300	4.6898750	-1.9797440	C	3.5857020	0.3433710	-1.9483410
H	-1.8287740	5.1289220	-1.0083600	H	4.4681940	0.9349220	-1.6687110
H	0.2611310	4.9298930	0.8524160	H	3.5882400	0.2163630	-3.0367950
H	1.8073270	4.5731350	0.0524940	H	2.6959090	0.9094610	-1.6631080
H	0.5654140	5.5175140	-0.7981470	C	4.7879540	-1.7543740	-1.5703760
H	0.1915590	2.1889680	-2.3362980	H	5.7001190	-1.2648960	-1.2017820
H	1.7452540	2.9366970	-1.9354840	H	4.8905920	-1.8949820	-2.6519900
H	0.4499710	3.9087170	-2.6827930	H	4.7121130	-2.7366300	-1.1064480
				H	0.0946150	-0.0853610	-2.5133170
**Ph_3 (dication)**				**Ph_3 (protonated)**			
C	2.7363740	-4.6693590	-0.5619850	C	1.2616910	4.3841850	1.6222350
N	2.4440170	-3.2365400	-0.3736570	N	2.1483210	3.7095830	0.6615280
C	3.6582100	-2.4063800	-0.4595900	C	3.1334840	4.6351880	0.0848600
C	4.6410390	-3.3301850	-1.1745910	C	2.9761230	5.9028170	0.9301860
C	4.2670900	-4.7133860	-0.6329630	C	1.5009230	5.8668180	1.3370000
P	0.9253100	-2.7144350	-0.0744570	P	1.8807730	2.2162360	0.0022050
N	0.4918740	-3.2253730	1.4261550	N	0.8620480	1.5081440	0.9927080
C	-0.8872390	-3.0586960	1.9385890	P	0.0615010	0.1261570	0.7481830
C	-0.6906430	-2.3895240	3.2963550	N	-1.2668350	0.2921290	-0.1476980
C	0.6181090	-3.0111530	3.7902440	P	-2.8023580	0.3821650	0.1977530
C	1.4797020	-3.0995980	2.5256250	N	-3.2748740	1.9598810	0.3161090
N	1.0650560	-1.1053640	-0.2630410	C	-2.3908510	3.0616750	0.6994790
P	0.0062880	0.0299440	-0.2688090	C	-3.0371440	4.2576200	0.0079670
N	0.4618460	1.5149170	-0.2940570	C	-4.5329950	3.9611590	0.1701610
P	1.9221140	2.1792190	-0.0203360	C	-4.6327360	2.4332230	0.0147430
N	1.6285410	3.7626450	-0.3050740	N	1.4517700	2.4878660	-1.5858290
C	0.3674070	4.3159680	-0.8380120	C	0.4342950	3.5134590	-1.8871520
C	0.6123090	5.8219340	-0.7785990	C	-0.6640640	2.7462710	-2.6186750

C	2.1103710	5.9424410	-1.0699640
C	2.7134590	4.7610800	-0.3040530
N	-1.5050500	-0.3193270	-0.3085950
P	-2.8404970	0.5738050	-0.0624730
N	-4.0368950	-0.4772300	-0.4272360
C	-5.4309740	-0.0268920	-0.5929600
C	-6.2160710	-1.3381990	-0.7157950
C	-5.1909520	-2.3186600	-1.2942170
C	-3.9058420	-1.9385710	-0.5633950
N	-2.8343390	1.9396100	-0.9623560
C	-2.3693650	1.9173680	-2.3660630
C	-2.8206760	3.2741040	-2.9160400
C	-4.0630740	3.6047520	-2.0865140
C	-3.6708160	3.1279030	-0.6906520
N	-3.0640900	1.1018570	1.4785530
C	-2.2295460	2.1794130	2.0570980
C	-1.7270650	1.5829320	3.3690260
C	-2.9106930	0.7241870	3.8211820
C	-3.4345490	0.1104710	2.5177910
N	3.0736230	1.4665020	-0.9465450
C	2.7987650	1.1619000	-2.3680570
C	4.1843360	0.8579470	-2.9474780
C	5.1295090	1.6875470	-2.0758770
C	4.5271750	1.5240370	-0.6832520
N	2.5032990	2.0584220	1.5121000
C	1.8062210	2.7997970	2.5935570
C	2.0654430	1.9622810	3.8512960
C	2.2530390	0.5431240	3.3113450
C	3.0445530	0.7783290	2.0286540
N	-0.2174070	-3.3396450	-1.0659530
C	-0.3249670	-2.8834270	-2.4684540
C	-1.2753060	-3.9020150	-3.1050470
C	-1.0277520	-5.1777350	-2.2973240
C	-0.8730230	-4.6507090	-0.8728890
H	-4.3212070	4.6651320	-2.1015880
H	-4.9247670	3.0336350	-2.4476130
H	-3.0139580	3.2324120	-3.9891170
H	-2.0445410	4.0258990	-2.7394710
H	-2.8399890	1.0919210	-2.9132650
H	-1.2837630	1.7945680	-2.4085870
H	-3.0758470	3.8881880	-0.1704420
H	-4.5266680	2.8771860	-0.0609820
H	-7.1054970	-1.2240680	-1.3375120
H	-5.5219380	0.5830370	-1.4997460
H	-5.7603780	0.5742100	0.2609570
H	-1.4472990	2.3483870	4.0955760
H	-2.9944540	-2.1924530	-1.1066820
H	-0.8490030	0.9542980	3.1795800
H	-5.0686090	-2.1527210	-2.3694800
H	-1.4258900	2.4596430	1.3710470
H	-3.8634890	-2.4177220	0.4239210
H	-2.8500670	3.0634670	2.2393180
H	-2.6346520	-0.0436910	4.5462690
H	-4.5152590	-0.0501550	2.5290160
H	-3.6789380	1.3584050	4.2731940
H	-2.9491620	-0.8538280	2.3258460
H	2.5381280	6.8955140	-0.7546480
H	2.2940170	5.8216360	-2.1421180
H	2.9738500	5.0388540	0.7238540
H	0.7320420	2.8632570	2.3868770
H	2.1963470	3.8165880	2.6765110
H	2.7712110	-0.1192660	4.0076730
H	2.9835960	2.2962040	4.3435140
H	1.2465580	2.0474720	4.5680550
H	1.2825800	0.0932530	3.0697090
H	4.1125690	0.8950550	2.2416160
H	2.9240210	-0.0190380	1.2918810
H	0.3904200	6.1958100	0.2260370
H	-0.4858890	3.9909000	-0.2399600
H	-0.0099950	6.3641870	-1.4929700
H	2.1187180	0.3099140	-2.4553930
H	2.3445840	2.0265260	-2.8665220
H	4.2426510	1.1058810	-4.0084520
H	4.4119090	-0.2074990	-2.8360100
H	6.1654890	1.3467730	-2.1192090
H	4.7765320	2.3385450	-0.0003480
H	4.8567200	0.5841030	-0.2239570
H	0.2048200	3.9826620	-1.8700290
H	5.0980270	2.7403410	-2.3762640
H	-6.5333400	-1.6738460	0.2764190
H	-5.4605740	-3.3641940	-1.1342890
H	3.6117640	4.3702920	-0.7922630
H	-0.7216490	-1.8653460	-2.5130500
H	0.6577360	-2.8998640	-2.9549820
H	-2.3131190	-3.5752560	-2.9801840
H	-1.0851110	-4.0211430	-4.1728310
H	-1.8377580	-5.9044410	-2.3809800
C	0.1371370	1.7364020	-3.4431550
C	1.3090310	1.3500700	-2.5256850
N	3.2933920	1.3708600	-0.1570850
C	3.8644760	0.7086500	1.0295680
C	5.2486830	0.2580610	0.5603030
C	5.6329030	1.3294420	-0.4637750
C	4.3150260	1.5798290	-1.1968630
N	-3.3183650	-0.3803630	1.5768410
C	-3.0272720	-1.8064610	1.8170420
C	-2.8057540	-1.8835860	3.3293360
C	-3.7110270	-0.7707290	3.8630530
C	-3.5108740	0.3509780	2.8434460
N	-3.6625820	-0.3470210	-1.0097070
C	-3.3521870	-0.0563940	-2.4184020
C	-4.4156650	-0.8470880	-3.1775890
C	-5.6317070	-0.7504900	-2.2530310
C	-5.0216830	-0.8972910	-0.8567230
H	3.2532950	6.8022000	0.3769950
H	3.6111460	5.8400390	1.8198200
H	1.2748270	6.4978330	2.1990470
H	0.8723970	6.1903830	0.4998430
H	1.5439050	4.1288790	2.6511130
H	0.2213270	4.0853190	1.4812090
H	2.9121160	4.8356570	-0.9723780
H	4.1452620	4.2204870	0.1460260
H	5.1825730	-0.7203220	0.0701560
H	3.2235830	-0.1203710	1.3400240
H	3.9509150	1.4147230	1.8664790
H	-1.2953280	3.3964730	-3.2293810
H	4.1849340	0.8500280	-2.0066280
H	-1.2876800	2.2192290	-1.8905460
H	6.4308270	1.0172960	-1.1407370
H	0.0802630	4.0047680	-0.9772630
H	4.2473020	2.5776370	-1.6358860
H	0.8702510	4.2877620	-2.5313040
H	-0.4495610	0.8625270	-3.7356520
H	2.2334010	1.2053970	-3.0934170
H	0.5116870	2.2099030	-4.3564740
H	1.1014120	0.4243630	-1.9808070
H	-6.3906860	-1.5095390	-2.4524250
H	-6.0997790	0.2346660	-2.3549140
H	-4.9684260	-1.9527090	-0.5626890
H	-2.1556470	-2.1403400	1.2422200
H	-3.8841540	-2.4228740	1.5163710
H	-4.7551980	-1.1001100	3.8570100
H	-3.0396300	-2.8701270	3.7342890
H	-1.7611560	-1.6543980	3.5702330
H	-3.4569930	-0.4539600	4.8763330
H	-4.3699130	1.0240360	2.7794470
H	-2.6279490	0.9505140	3.1027910
H	-4.0997300	-1.8909520	-3.2781860
H	-2.3317350	-0.3632250	-2.6607930
H	-4.5963920	-0.4450420	-4.1762790
H	-2.3786190	3.2041480	1.7902820
H	-1.3695690	2.8506050	0.3767430
H	-2.7592440	4.2626020	-1.0516820
H	-2.7397430	5.2112350	0.4495590
H	-4.8595200	4.2538400	1.1729070
H	-4.9121200	2.1474570	-1.0063570
H	-5.3772770	2.0066170	0.6975850
H	-3.4382980	1.0197300	-2.6286250
H	-5.1590860	4.4861790	-0.5536890
H	5.9559630	0.1726970	1.3875720
H	5.9552790	2.2412830	0.0511960
H	-5.5932580	-0.3666530	-0.0912420
N	1.0320910	-0.9952280	0.1265920
P	1.0643350	-2.5657870	0.0548410
N	2.1075850	-2.9332180	-1.1666480
C	2.4469520	-4.3204160	-1.5138740
H	2.7583630	-4.8778770	-0.6217880
H	1.5872840	-4.8409770	-1.9511420
C	3.6054000	-4.1736360	-2.5120940
H	4.3042620	-5.0100920	-2.4523770
H	3.2151030	-4.1306740	-3.5339280
C	4.2330090	-2.8254120	-2.1402680
H	4.8615530	-2.9334450	-1.2487820
H	4.8409410	-2.3987590	-2.9410270
C	3.0110220	-1.9718680	-1.8126390
H	2.5579800	-1.5735100	-2.7305130
H	3.2077600	-1.1270000	-1.1506140
N	-0.4114890	-3.2819700	-0.1919150
C	-1.1691420	-2.8755810	-1.3929300
H	-1.4639830	-1.8291070	-1.3108770
H	-0.5570500	-3.0048940	-2.2976360
C	-2.3515950	-3.8377160	-1.3993190
H	-3.1280640	-3.4750620	-0.7172030
H	-2.7929810	-3.9441830	-2.3926260

H	-0.0978930	-5.6572520	-2.6207150
H	-0.2766280	-5.3033860	-0.2322350
H	-1.8514270	-4.5085610	-0.3985800
H	-1.4898780	-2.4682400	1.2438310
H	-1.3557890	-4.0425490	2.0506750
H	-0.5735460	-1.3074130	3.1663670
H	-1.5313310	-2.5618090	3.9714620
H	1.1064720	-2.4245800	4.5705750
H	0.4282820	-4.0132560	4.1860470
H	2.1582650	-3.9559160	2.5335080
H	2.0804450	-2.1899930	2.4060150
H	3.4521970	-1.4792120	-0.9962670
H	4.0239130	-2.1505850	0.5438240
H	4.4719340	-3.2856840	-2.2554050
H	5.6792540	-3.0582170	-0.9757120
H	4.6805090	-4.8441710	0.3719190
H	4.6228350	-5.5342980	-1.2576160
H	2.2801600	-5.0253590	-1.4936760
H	2.3418290	-5.2686160	0.2652040

C	-1.7324790	-5.1337860	-0.8691750
H	-2.4688960	-5.8447330	-0.4891330
H	-1.1642010	-5.6253430	-1.6661010
C	-0.7802110	-4.6497870	0.2289660
H	0.0987240	-5.2890720	0.3408680
H	-1.2897160	-4.6099300	1.1999960
N	1.6336000	-3.3660920	1.4102430
C	3.0734350	-3.1728190	1.7141710
H	3.6415290	-4.0712750	1.4556380
H	3.4848200	-2.3431530	1.1288760
C	3.1370500	-2.8602590	3.2192140
H	3.3367010	-3.7744070	3.7866410
H	3.9271920	-2.1425310	3.4499150
C	1.7350040	-2.3402060	3.5466020
H	1.6365750	-1.2913720	3.2441720
H	1.4810920	-2.4201440	4.6057530
C	0.8563860	-3.2164930	2.6578770
H	0.6945720	-4.2011600	3.1120240
H	-0.1264410	-2.7831570	2.4537030
H	-0.3433630	-0.2525600	2.0484040

I-Pa$_2$

C	-4.4343790	0.8061770	-0.5524480
C	-3.1837910	0.3904880	-1.3273130
C	-1.9958980	0.8708780	-0.4482990
C	-2.5700300	0.7013230	1.0148130
C	-3.9980200	0.1480750	0.7567810
C	-1.8990090	-0.4438940	1.7825090
C	-2.2327540	-1.6238970	0.8720930
C	-1.6615720	-1.4532930	-0.5773040
C	-0.8964720	-0.1454740	-0.8028120
C	-3.6651730	-1.3086610	0.3428220
C	-3.1000880	-1.1406190	-1.0980660
P	0.8017160	0.0599140	-0.0589120
N	1.2582800	1.4806810	-0.8864700
P	1.6846160	2.7597930	-0.0510220
C	2.2274730	4.0676320	-1.1804110
N	1.5995240	-1.1776320	-0.9235030
P	2.3032030	-2.3438110	-0.1104030
C	3.5983560	-1.8439420	1.0753960
C	1.1948990	-3.3954690	0.8869700
C	3.1249890	-3.4753400	-1.2612780
C	0.3701200	3.5192080	0.9615030
C	3.0600240	2.5420160	1.1294510
H	-0.8282510	-0.3019930	1.9401330
H	-5.3574570	0.3814860	-0.9621300
H	-4.5501180	1.8925660	-0.4715580
H	-1.1649030	-2.3353960	-0.9854260
H	-2.3816850	-0.5688610	2.7599480
H	-0.7043510	-0.0568500	-1.8832490
H	-4.6737640	0.2423210	1.6104530
H	-2.5689240	1.6520310	1.5557220
H	-4.4617610	-2.0366510	0.5071730
H	-3.5176060	-1.7587250	-1.8953000
H	-3.1180580	0.7061630	-2.3712290
H	-1.7054540	1.9049690	-0.6496030
H	-2.0690910	-2.6080620	1.3171290
H	0.7348780	4.3974440	1.5004480
H	-0.4608390	3.8091710	0.3138800
H	0.0090930	2.7770110	1.6792610
H	2.5075730	4.9636240	-0.6210940
H	3.0872870	3.7158110	-1.7545480
H	1.4152450	4.3104620	-1.8687490
H	3.3072050	3.4845470	1.6243700
H	2.7670240	1.8050840	1.8813850
H	1.7472220	-4.1852900	1.4026560
H	0.6864080	-2.7669640	1.6237040
H	0.4409430	-3.8426230	0.2346850
H	2.3858940	-3.8929760	-1.9481560
H	3.8738050	-2.9255710	-1.8353660
H	3.6117510	-4.2873890	-0.7157000
H	4.3698640	-1.2767770	0.5492200
H	3.1507220	-1.2029520	1.8389720
H	4.0500200	-2.7157840	1.5552490
H	3.9376260	2.1691530	0.5961310

I-Pb$_2$

C	-5.0827730	-0.3360260	-0.9893420
C	-3.7145420	-0.6126660	-1.6127450
C	-2.7071920	0.1552280	-0.7124390
C	-3.3814650	0.0494600	0.7128850
C	-4.6702940	-0.7651600	0.4189470
C	-2.6085020	-0.8736840	1.6614820
C	-2.6591810	-2.1845530	0.8784910
C	-1.9910130	-2.0777830	-0.5366560
C	-1.4303380	-0.6898180	-0.8608330
C	-4.0685800	-2.1790170	0.2111370
C	-3.4074540	-2.0735170	-1.1943110
P	0.1365510	-0.1349750	-0.0203470
N	0.3590040	1.3417410	-0.8479620
P	0.4897570	2.7376530	-0.1101590
C	1.8448140	2.9335360	1.1204270
C	1.6093100	2.2265200	2.4580270
H	-1.5945930	-0.5346220	1.8809420
N	1.1836710	-1.2249690	-0.8106090
P	2.4807440	-1.7302540	-0.0444320
C	3.7083050	-0.4385540	0.3905670
C	4.0824410	0.4529580	-0.7930310
C	2.1920800	-2.5675260	1.5610980
C	1.2280550	-3.7488230	1.4545850
C	3.3340030	-2.9291440	-1.1211600
C	4.5828150	-3.5876340	-0.5344140
C	0.8245040	4.0245390	-1.3587370
C	2.0762550	3.7372510	-2.1893070
C	-1.0272740	3.2768070	0.7683410
C	-0.9823280	4.6462470	1.4479780
H	-5.8830430	-0.9535700	-1.4118820
H	-5.3790390	0.7169750	-1.0497570
H	-1.3220930	-2.9007160	-0.7954850
H	-3.1545340	-0.9755240	2.6078610
H	-1.1568300	-0.6823990	-1.9272610
H	-5.4293680	-0.6971280	1.2022250
H	-3.5842940	1.0393550	1.1318550
H	-4.7483000	-3.0109720	0.4045130
H	-3.6455450	-2.8355940	-1.9390090
H	-3.6031690	-0.4040370	-2.6794530
H	-2.5671200	1.1953940	-1.0161450
H	-2.3813720	-3.0719440	1.4520880
H	0.9075080	4.9899070	-0.8491250
H	-0.0611130	4.0712230	-2.0010880
H	2.0094530	4.0058200	1.2767850
H	2.7460910	2.5440750	0.6349720
H	-1.8293670	3.2376520	0.0232590
H	-1.2473450	2.4842390	1.4938040
H	3.1561680	-2.8727320	1.9816780
H	1.7799610	-1.7994390	2.2247440
H	2.5837250	-3.6809830	-1.3860810
H	3.5722400	-2.3926530	-2.0451670
H	3.2429090	0.1583860	1.1836970
H	4.5903130	-0.9142830	0.8327940
H	-0.1906390	4.7016190	2.2005900
H	-0.8148870	5.4489270	0.7245950
H	-1.9312020	4.8488590	1.9514740
H	1.9929520	2.7659780	-2.6826100
H	2.2094930	4.5053840	-2.9553630
H	2.9763970	3.7260550	-1.5673090
H	1.3841970	1.1668030	2.3103220
H	2.4967130	2.3098030	3.0914460
H	0.7699340	2.6688530	3.0026690
H	4.7601470	1.2499380	-0.4737760
H	4.5862280	-0.1151730	-1.5811210
H	3.1842540	0.9090740	-1.2187610

H	5.0204970	-4.2747570	-1.2627430
H	5.3476640	-2.8497090	-0.2764240
H	4.3507200	-4.1642180	0.3655100
H	1.0374410	-4.1786230	2.4412930
H	0.2719930	-3.4290130	1.0299480
H	1.6324570	-4.5418390	0.8176320

I-Pc2

C	4.1296420	0.8752050	-1.6634960
C	4.0550470	-0.6648900	-1.8305320
C	5.3455980	-1.1646320	-1.1813120
C	5.1223040	-0.4205740	0.1355930
C	4.8710030	1.0434560	-0.3052400
C	3.6919970	-0.8322480	0.5763370
C	2.9394560	-1.0038650	-0.8043130
C	2.7935360	1.3363030	-0.9984930
C	1.8981090	0.0960780	-1.0798690
C	3.5426070	1.5063790	0.3671570
C	3.2104510	0.3948560	1.3598470
P	0.2760200	0.0473790	-0.1584000
N	-0.4189890	1.4385580	-0.8616110
P	-1.3184770	2.3787650	0.0509630
C	-1.8275210	3.8149890	-0.9472100
C	-2.6886420	4.8612610	-0.2351160
C	-3.0184990	6.0400760	-1.1490500
N	-0.4306630	-1.2512030	-1.0074160
P	-1.0239400	-2.5381460	-0.3013700
C	-1.9767790	-2.2231150	1.2314250
C	-2.6534340	-3.4306430	1.8830860
C	-3.3824830	-3.0428120	3.1683950
C	-2.1519990	-3.4040190	-1.4407590
C	-3.3756650	-2.5761790	-1.8405450
C	-4.2877820	-3.3256360	-2.8088850
C	0.2162010	-3.8083550	0.1555220
C	1.0908350	-3.3956770	1.3442170
C	2.3476690	-4.2537550	1.4609350
C	-2.8492670	1.6249300	0.7223590
C	-3.7101820	0.9551830	-0.3507840
C	-4.9592350	0.2939510	0.2276110
C	-0.4966380	3.0383710	1.5493840
C	0.7382960	3.8854240	1.2324430
C	1.5986270	4.1399300	2.4676190
H	6.2491140	-0.8434920	-1.7112060
H	5.3733420	-2.2534180	-1.0614490
H	2.3255490	2.2420980	-1.3873440
H	3.8038610	0.5090740	2.2756660
H	1.5834810	-0.0084860	-2.1295430
H	5.8808110	-0.5455030	0.9123530
H	3.6771980	-1.7578550	1.1579740
H	5.7407440	1.7019400	-0.2658790
H	4.5029730	1.4237510	-2.5305010
H	3.8428850	-1.0103940	-2.8450780
H	2.5413970	-2.0159870	-0.9229240
H	3.5206360	2.5156470	0.7847840
H	0.8324220	-3.9446640	-0.7416990
H	-0.2805590	-4.7655380	0.3521340
H	-2.4580450	-4.3466770	-0.9722230
H	-1.5610570	-3.6655340	-2.3261610
H	-1.2828460	-1.7406410	1.9294190
H	-2.7209860	-1.4625160	0.9708370
H	-1.2186450	3.5958120	2.1570150
H	-0.2058460	2.1508370	2.1251190
H	-0.8995950	4.2668070	-1.3162980
H	-2.3483930	3.4183840	-1.8258480
H	-2.5210100	0.8832350	1.4621170
H	-3.4169020	2.3864470	1.2703930
H	-3.3667440	-3.8789260	1.1818490
H	-1.9080260	-4.2031280	2.1054410
H	1.3791990	-2.3421780	1.2434940
H	0.5065330	-3.4676410	2.2688540
H	-3.9452770	-2.3021350	-0.9439110
H	-3.0376030	-1.6390280	-2.2947300
H	-4.0107390	1.6974090	-1.1001260
H	-3.0974640	0.2142060	-0.8756670
H	-3.6200940	4.4016300	0.1140980
H	-2.1677720	5.2284350	0.6568110
H	1.3367370	3.3715880	0.4723660
H	0.4259450	4.8409170	0.7940950
H	-3.5647690	5.7054010	-2.0367400
H	-3.6361420	6.7786930	-0.6306620
H	-2.1049300	6.5399780	-1.4865870
H	-4.6988640	-0.4647610	0.9744360
H	-5.6035760	1.0300950	0.7190500
H	-5.5426520	-0.1966850	-0.5571200
H	1.9678350	3.1948220	2.8804580
H	2.4654480	4.7610490	2.2231450
H	1.0278250	4.6489330	3.2512630

I-Pd2

C	-2.9462630	-2.4705870	-1.6329780
C	-3.7675530	-1.9725620	-0.4398850
C	-4.5786380	-3.2286890	-0.0260050
C	-3.4836420	-4.3190950	-0.1316010
C	-2.3548650	-3.7521370	-1.0463420
C	-2.6052560	-4.0517740	1.1256990
C	-3.3090200	-2.8397780	1.7908340
C	-4.7886940	-3.0651740	1.4794790
C	-2.8703780	-1.6980680	0.8305740
C	-1.3776870	-2.0126560	0.6239860
C	-1.4676990	-3.4861000	0.2184320
P	-0.3032440	-0.8451990	-0.3606460
N	1.1693770	-1.6119610	0.0111640
P	2.5775940	-0.9110810	-0.1501640
C	2.9549240	0.4116250	1.0634990
C	2.5720750	0.0591360	2.4991660
C	2.7426290	1.2429240	3.4502660
C	2.2626330	0.9352980	4.8658070
H	-2.1904330	-1.7606410	-1.9765530
N	-0.3912590	0.4934330	0.6936150
P	-0.7981740	1.9641470	0.2802990
C	-2.5886010	2.2429500	0.0019930
C	-3.1052720	1.5509300	-1.2612470
C	-4.6267830	1.6053190	-1.3876530
C	-5.1267450	0.9457410	-2.6709560
C	-0.3260810	3.1131090	1.6146160
C	-0.7849250	2.6638810	3.0032490
C	-0.3120050	3.6074780	4.1091100
C	-0.8018400	3.1823960	5.4912020
C	0.0240580	2.6091970	-1.2268600
C	-0.3871710	4.0002800	-1.7124020
C	0.3546640	4.4016710	-2.9886100
C	-0.0329450	5.7930900	-3.4828640
C	3.8710200	-2.1839480	0.0357690
C	5.3096410	-1.7370910	-0.2320980
C	6.3146020	-2.8698870	-0.0164570
C	7.7530250	-2.4382660	-0.2916790
C	2.8859310	-0.0587210	-1.7435330
C	2.7182490	-0.9581250	-2.9688830
C	2.7843400	-0.1918480	-4.2943320
C	1.6789990	0.8532320	-4.4546290
H	-5.1991830	-3.9609210	1.9584260
H	-5.4188160	-2.2063600	1.7357640
H	-1.9176920	-4.4326250	-1.7802030
H	-0.9027640	-1.9739500	1.6157820
H	-3.6105230	-2.7040640	-2.4746390
H	-3.0419710	-2.6666510	2.8360270
H	-3.0447460	-0.6989710	1.2393010
H	-2.3823140	-4.8878560	1.7914790
H	-3.8456350	-5.3339590	-0.3063780
H	-5.4678010	-3.4049000	-0.6364550
H	-4.4066860	-1.1165180	-0.6682720
H	-0.5104550	-4.0094620	0.2296730
H	3.7726750	-2.5766300	1.0543770
H	3.5906220	-2.9992980	-0.6403060
H	5.4019260	-1.3706880	-1.2622120
H	5.5742200	-0.8971190	0.4220910
H	6.2281640	-3.2354920	1.0142280
H	6.0500300	-3.7127910	-0.6668540
H	8.0479630	-1.6142950	0.3667090
H	8.4530940	-3.2630470	-0.1310970
H	7.8685960	-2.0960120	-1.3255490
H	4.0148340	0.6809370	0.9816550
H	2.3739280	1.2829290	0.7406360
H	1.5242490	-0.2583550	2.5004630
H	3.1719290	-0.7888260	2.8557730
H	3.7943300	1.5553880	3.4711150
H	2.1767470	2.0951760	3.0531670
H	1.2098540	0.6321650	4.8587040
H	2.8400100	0.1177050	5.3103640
H	2.3549060	1.8082100	5.5191290
H	2.1579330	0.7586940	-1.7722850
H	3.8794870	0.4047650	-1.7215490
H	3.4920870	-1.7354770	-2.9616130
H	1.7533210	-1.4747540	-2.9002000
H	3.7645020	0.2926150	-4.3877390
H	2.7174170	-0.9153940	-5.1147500
H	0.6926320	0.4083870	-4.2796040
H	1.7977990	1.6831800	-3.7509000

H	-4.1513720	-2.2890720	2.9686540	H	-3.0989330	1.8460130	0.8876700
H	-3.8699570	-3.9096270	3.6232470	H	-2.7903130	3.3200560	-0.0319180
H	-2.6851610	-2.6228820	3.9004130	H	-2.6548790	2.0135250	-2.1488720
H	2.9783780	-4.1373600	0.5724850	H	-2.7801240	0.5033240	-1.2643670
H	2.9411540	-3.9681190	2.3344690	H	-5.0756190	1.1122870	-0.5154320
H	2.0951020	-5.3149210	1.5555150	H	-4.9583440	2.6506480	-1.3542730
H	-3.7534390	-3.5854390	-3.7284640	H	-6.2181880	0.9729620	-2.7353400
H	-4.6562570	-4.2548180	-2.3619740	H	-4.7242560	1.4543040	-3.5533530
H	-5.1538940	-2.7163890	-3.0826410	H	-4.8120250	-0.1025070	-2.7213590
H	2.1540420	0.3552240	1.6341490	H	-0.1471320	1.8600090	-2.0100970
				H	1.0993550	2.5910830	-1.0093380
				H	-1.4665900	4.0297730	-1.9049130
				H	-0.1902580	4.7441570	-0.9304400
				H	1.4357460	4.3609740	-2.8044820
				H	0.1464770	3.6595830	-3.7696800
				H	0.5075420	6.0547800	-4.3969080
				H	-1.1050930	5.8470540	-3.6993290
				H	0.1934350	6.5542450	-2.7287750
				H	-0.7232410	4.1065660	1.3760320
				H	0.7679350	3.1901450	1.5835930
				H	-1.8805670	2.5998380	3.0283430
				H	-0.4086810	1.6515770	3.1893300
				H	0.7848580	3.6475360	4.1036470
				H	-0.6587270	4.6255440	3.8913990
				H	-0.4483090	2.1763640	5.7392850
				H	-0.4432550	3.8643730	6.2675600
				H	-1.8961740	3.1709380	5.5326660
				H	1.6832790	1.2789380	-5.4621240
**I-Pe$_2$**				**I-Pf$_2$**			
C	2.2474610	2.2654010	2.9885150	C	-2.4099210	-2.0086260	-1.7437500
C	2.9617250	2.0751200	1.6338650	C	-3.4863190	-1.2720140	-0.9381500
C	3.2847300	0.5550220	1.5294730	C	-2.9608100	-0.7955500	0.4749310
C	2.5929710	-0.0930510	2.7503900	C	-1.4978190	-1.1824280	0.7567880
C	2.6006800	1.0172000	3.8023500	C	-1.5886900	-2.7028410	0.5967180
P	2.8112240	-0.2040620	-0.0869470	C	-2.1093030	-3.1745430	-0.8042380
C	4.2031880	0.1664630	-1.2233750	C	-2.9740210	-3.0610120	1.2220440
C	4.6373000	1.6496210	-1.1769630	C	-3.7376300	-1.7291670	1.4444420
C	5.2490000	1.8896800	-2.5579970	C	-4.4791500	-2.4065410	-0.5693890
C	4.2712770	1.1642230	-3.4854520	C	-3.4888580	-3.5296270	-0.1703630
C	3.8913450	-0.1217100	-2.7225010	C	-5.0856490	-1.9658210	0.7632690
N	1.4652100	0.2924420	-0.7560410	P	-0.1224580	-0.2432870	-0.0855060
P	-0.0056760	0.5266620	0.0642160	N	-0.3336430	1.2323190	0.7333530
N	-1.0095770	-0.7051570	-0.5403420	P	-0.4532700	2.7049340	0.1734400
P	-2.2029690	-1.4513730	0.1725490	C	-0.4844510	3.8495160	1.6214790
C	-3.8378530	-0.9422530	-0.4974240	C	-1.7360970	3.6321140	2.4784010
C	-5.0634440	-1.7710730	-0.0581460	N	1.1559200	-1.0146670	0.7320880
C	-6.0912520	-1.4853340	-1.1570960	P	2.6016300	-1.2332230	0.1216610
C	-5.2471080	-1.5366710	-2.4351030	C	2.6842780	-2.1261420	-1.5048080
C	-3.9121160	-0.8657610	-2.0510260	C	2.3035580	-1.2358310	-2.6947360
C	-0.5758400	1.9469500	-1.0098050	C	3.5246960	0.3504650	-0.1457150
C	-2.0134480	2.4862120	-0.9012390	C	3.4491520	1.2261520	1.1089070
C	-2.0350120	3.5861850	-1.9998180	C	3.6015440	-2.2178580	1.3379710
C	-0.6694340	4.2722010	-1.7363500	C	2.7154690	-3.0938830	2.2290860
C	0.2044070	3.2620210	-0.9299730	C	4.9647430	0.2002580	-0.6427730
C	-3.0565880	4.6110020	-1.5066770	C	0.9956320	3.2659190	-0.8480010
C	-2.4189850	4.7721610	-0.1261950	C	1.0249370	2.6037860	-2.2312450
C	-0.9345490	5.0902500	-0.4385540	C	-2.0470060	3.0281050	-0.7227110
C	-2.2898420	3.3221640	0.4111430	C	-2.2868310	2.0721820	-1.8966300
C	-1.0565730	3.3988220	1.3163450	C	1.2172550	4.7790090	-0.9552300
C	-0.0646270	4.0848860	0.3779140	C	-2.2961680	4.4818570	-1.1355390
H	-0.7130120	2.4247310	1.6711990	C	0.7843950	3.6678070	2.4618390
C	2.8771070	-2.0101460	0.2495810	C	4.7336470	-3.0453380	0.7156360
C	2.6384980	-2.8935650	-0.9940540	C	1.7862610	-3.3674080	-1.4502210
C	3.2752890	-4.2283650	-0.6031300	H	-5.6824830	-2.7478670	1.2455710
C	4.5806160	-3.7900210	0.0669620	H	-5.6926230	-1.0574060	0.6814320
C	4.1860280	-2.5521720	0.8990010	H	-1.5425490	-3.9927470	-1.2548970
C	-2.0775450	-3.2694610	-0.0560770	H	-1.3087270	-0.9770480	1.8219110
C	-0.7622820	-3.8687730	0.4932380	H	-3.7585820	-1.3753950	2.4780250
C	-0.4696590	-5.1155750	-0.3830240	H	-3.1635650	0.2659660	0.6423440
C	-1.6149360	-5.1900180	-1.4099620	H	-3.0105690	-3.7672110	2.0540290
C	-2.1183640	-3.7482140	-1.5151070	H	-3.8691740	-4.5481920	-0.2683850
C	-2.4343580	-1.2403940	1.9887640	H	-5.1750410	-2.6676030	-1.3706760
C	-1.1517820	-1.4640720	2.8452650	H	-3.9593520	-0.4518690	-1.4848210
C	-1.0104110	-0.2037290	3.7193310	H	-0.7157030	-3.2414290	0.9685920
C	-2.4256820	0.3723870	3.8005110	H	4.0570190	-1.4508180	1.9773050
C	-2.9606670	0.1598640	2.3824470	H	3.7236690	-2.4477440	-1.6350200
H	-3.0577430	5.5366530	-2.0927820	H	1.8259860	2.8481700	-0.2664660
H	-4.0779570	4.2166180	-1.4660460	H	-0.5034340	4.8705460	1.2242080
H	0.8299830	4.4769700	0.8675830	H	2.9333450	0.8436910	-0.9291050
H	-0.4532650	1.5515050	-2.0300640	H	0.3232210	3.0902270	-2.9152380
H	-1.2656600	4.0347970	2.1857190	H	2.0261800	2.7023390	-2.6640090
H	-2.1697860	3.1975080	-3.0121120	H	0.7774740	1.5390690	-2.1798950
H	-2.7732360	1.7163710	-1.0544060	H	1.3497540	5.2531860	0.0201540
H	-0.2290080	4.7854190	-2.5935480	H	2.1263270	4.9660820	-1.5361660
H	-0.6689630	6.1490470	-0.4279350	H	0.3929450	5.2808110	-1.4677020
H	-2.9035110	5.4630420	0.5682840	H	-2.7739580	2.7610440	0.0551570

H	-3.1927990	2.9770160	0.9231120
H	1.2553250	3.1702360	-1.2118830
H	3.4292220	-4.8956100	-1.4557620
H	2.6359630	-4.7493660	0.1209330
H	5.0408390	-4.5727180	0.6754800
H	5.3041490	-3.5052470	-0.7056870
H	3.9926030	-2.8314110	1.9382110
H	4.9935810	-1.8141650	0.9216790
H	3.1655030	-2.4921220	-1.8657690
H	1.5810690	-2.9633190	-1.2588050
H	6.2413750	1.4257370	-2.6140020
H	5.3213810	1.8578570	-0.3499710
H	3.7611110	2.2974260	-1.0576700
H	3.6037730	1.1145100	4.2356690
H	4.4892060	-0.9706500	-3.0671140
H	1.8990260	0.8267050	4.6199030
H	4.6865310	0.9568240	-4.4752690
H	1.5563580	-0.3441560	2.5050060
H	2.8385250	-0.3692970	-2.8733940
H	3.0961080	-1.0052240	3.0807750
H	1.1650650	2.2974260	2.8276070
H	3.8869680	2.6559720	1.5877910
H	2.5352910	3.1965390	3.4845090
H	2.3253670	2.4113720	0.8114400
H	0.4880520	-4.9961560	-0.8981130
H	-0.3979590	-6.0282150	0.2142140
H	0.0450040	-3.1364890	0.3921750
H	-0.2611930	-1.6147360	2.2295540
H	-1.2730880	-2.3593880	3.4621530
H	-3.0302570	-0.1946320	4.5191760
H	-0.5698010	-0.4175520	4.6973110
H	-0.3653260	0.5142000	3.2020790
H	-2.4435970	1.4236080	4.1029410
H	-4.0503300	0.2272470	2.3157600
H	-2.5375180	0.9247600	1.7230790
H	-1.2950430	-5.5999080	-2.3717900
H	-1.4276070	-3.1470120	-2.1186090
H	-2.4226310	-5.8275660	-1.0323690
H	-4.8362290	-2.8434020	-0.0545390
H	-5.4092150	-1.5043860	0.9445660
H	-6.5096260	-0.4807120	-1.0210040
H	-6.9220800	-2.1963460	-1.1602630
H	-5.0765460	-2.5838190	-2.7122800
H	-3.8983950	0.1815740	-2.3656060
H	-3.0581340	-1.3419400	-2.5372550
H	-3.1141500	-3.6740490	-1.9611290
H	-5.7287710	-1.0509570	-3.2877820
H	5.3612500	2.9515800	-2.7942980
H	3.3792410	1.7856100	-3.6251260
H	-0.8476400	-4.1132160	1.5547030
H	-3.9628160	0.0736100	-0.1039490
H	-3.1872030	-1.9871220	2.2691220
H	-2.9222440	-3.7188790	0.4802320
H	4.3675410	0.4103750	1.6278520
H	2.0442030	-2.1751530	0.9452270
H	5.0490380	-0.4521780	-0.9015960

H	-2.0113970	1.0439790	-1.6493240
H	-3.3483970	2.0846350	-2.1641040
H	-1.7199490	2.3708620	-2.7817390
H	-1.6535830	4.7705000	-1.9724550
H	-3.3327030	4.5936480	-1.4692180
H	-2.1340830	5.1903780	-0.3186060
H	-2.6533750	3.9100190	1.9522590
H	-1.8145420	2.5840580	2.7823460
H	-1.6753420	4.2466770	3.3818350
H	1.6978200	3.8498400	1.8879560
H	0.7740270	4.3655390	3.3047150
H	0.8311910	2.6486360	2.8551910
H	5.3497450	-3.4710920	1.5134120
H	4.3376280	-3.8791790	0.1274980
H	5.3896210	-2.4572340	0.0700580
H	1.9087180	-2.5111680	2.6742550
H	2.2639900	-3.9124120	1.6595010
H	3.3233120	-3.5356800	3.0252190
H	5.0432800	-0.4427330	-1.5243300
H	5.3663950	1.1822700	-0.9117520
H	5.6096410	-0.2117730	0.1398650
H	3.8577850	2.2196380	0.8936060
H	2.4146690	1.3353780	1.4434750
H	0.7436770	-3.0785720	-1.2865350
H	1.8496360	-3.9152870	-2.3956510
H	2.0726060	-4.0518700	-0.6462330
H	2.3414540	-1.8261650	-3.6161620
H	1.2884350	-0.8466840	-2.5751460
H	2.9841130	-0.3888190	-2.8181590
H	-1.5341720	-1.3969710	-1.9718350
H	-2.8342990	-2.3754870	-2.6867100
H	4.0383850	0.8040760	1.9303400

I-Pg$_2$

C	0.8610610	3.9762730	-1.2573660
C	2.2344270	3.2629520	-1.3679200
C	3.1838460	4.1840360	-0.6014550
C	2.3269560	4.2260460	0.6643010
C	0.9264530	4.6433580	0.1476430
C	2.0674930	2.7346590	1.0078220
C	2.0003160	2.0560710	-0.4187260
C	-0.1647710	2.9285440	-0.7186850
C	0.5859760	1.5988360	-0.8211600
C	-0.0973280	3.6013870	0.6945980
C	0.7023480	2.7778690	1.7025220
H	0.2788970	1.7900610	1.8955300
P	-0.0913950	0.0642380	-0.0082300
N	-1.5563620	-0.0401260	-0.8708640
P	-2.9420350	-0.2647280	-0.1745260
N	-2.9757340	-1.6032990	0.8513160
C	-2.1601070	-2.7653600	0.5213390
N	0.8811370	-1.0774420	-0.8066840
P	1.9583650	-1.9632170	-0.1011860
N	2.1738480	-1.9431990	1.5668110
C	1.1513050	-2.5560210	2.4084500
N	1.6127220	-3.5875120	-0.3810650
C	2.5153290	-4.6395090	0.0599410
N	3.4987780	-1.5298220	-0.6336300
C	4.6991690	-2.1993860	-0.1552970
C	0.7870260	-3.9696340	-1.5157150
N	-3.6013520	0.9173700	0.8365130
C	-2.9725770	1.1254040	2.1389020
N	-4.0969680	-0.3404510	-1.3960160

I-Ph$_2$

C	1.0992670	-2.4033120	-1.6756390
N	2.2255890	-2.4019790	-0.7394800
C	3.3371040	-2.9419680	-1.5343420
C	3.1660860	-2.3437290	-2.9515850
C	1.6810670	-1.9068540	-3.0088040
P	2.5265860	-1.0059570	0.1777770
N	1.2459150	-0.2736350	0.6649280
P	-0.0601660	0.5508270	-0.0332730
N	-1.4139660	-0.0342090	0.8172720
P	-2.4853370	-0.9359930	0.1059480
N	-1.9617240	-2.4808120	-0.2266320
C	-1.1801680	-3.1814440	0.8096460
C	-1.1565020	-4.6370090	0.3360040
C	-2.4682100	-4.7774470	-0.4411860
C	-2.5764170	-3.4332760	-1.1614700
N	3.4625220	-1.6459270	1.4067600
C	3.9742790	-0.7061440	2.4275330
C	3.6208440	-1.3602450	3.7639880
C	3.6769500	-2.8514270	3.4296710
C	3.0317940	-2.9062890	2.0452120
N	3.6345910	0.0015850	-0.5720070
C	5.0778550	-0.1636560	-0.6900320
C	5.5938090	1.2841770	-0.6567260
C	4.4224130	2.1278520	-1.2191040
C	3.2718830	1.1225400	-1.4255080
C	0.0598860	2.1551670	0.9157340
C	-1.1239820	3.1300130	0.7943510
C	-0.7362240	4.2486610	1.7981110
C	0.7651180	4.4239950	1.4540520

C	-5.4890380	-0.6027990	-1.0571350
C	-3.9911790	2.1847470	0.2257400
C	-3.7149510	-0.8773250	-2.6949760
C	-4.1430930	-1.9438860	1.6541800
C	3.6534580	-0.9531600	-1.9609130
C	2.7594020	-0.7560800	2.1827290
H	3.3077710	5.1657900	-1.0717070
H	4.1730810	3.7425610	-0.4377050
H	-1.1547940	2.9154280	-1.1781410
H	0.7842930	3.3147800	2.6558490
H	0.6202090	1.3227600	-1.8863490
H	2.7052740	4.8175380	1.5017970
H	2.8569790	2.2921730	1.6214740
H	0.6931050	5.7065560	0.2294310
H	0.5824070	4.5943410	-2.1131370
H	2.5280070	2.9784520	-2.3813060
H	2.7521390	1.2693340	-0.5201740
H	-1.0498110	3.9856080	1.0683280
H	3.0403480	-4.3349040	0.9662670
H	4.9868530	-3.0463250	-0.7943600
H	1.9346720	-5.5394340	0.2923720
H	5.5307610	-1.4854050	-0.1464010
H	4.5531680	-2.5613260	0.8629980
H	0.1397450	-3.1374560	-1.7900960
H	1.3952110	-4.2510550	-2.3880850
H	0.1661190	-4.8314520	-1.2429460
H	2.7308430	-0.4484420	-2.2470370
H	4.4700470	-0.2214110	-1.9476590
H	3.8936950	-1.7177760	-2.7146110
H	0.3295610	-1.8566730	2.6175210
H	1.6030900	-2.8598850	3.3590900
H	0.7410090	-3.4386130	1.9177100
H	3.2795830	-1.0445810	3.1030230
H	1.9900610	-0.0124650	2.4319510
H	-4.7879950	-2.6761100	1.1482610
H	-4.7307380	-1.0520720	1.8713020
H	-3.8158910	-2.3812840	2.6047840
H	-1.2472790	-2.4600570	0.0104120
H	-2.7033820	-3.4822950	-0.1123250
H	-1.8767610	-3.2793560	1.4469350
H	-4.7195780	2.6842220	0.8730230
H	-3.1303780	2.8572470	0.0962310
H	-4.4487140	2.0112750	-0.7479650
H	-3.6992180	1.5827630	2.8188590
H	-4.3541070	-0.4367400	-3.4683410
H	-2.1006650	1.7914770	2.0662810
H	-2.6759830	-0.6226120	-2.8991950
H	-2.6451110	0.1762070	2.5623380
H	-5.7444490	-0.1334540	-0.1062670
H	-5.7062020	-1.6788820	-0.9901940
H	-6.1340530	-0.1753470	-1.8324090
H	-3.8315820	-1.9706870	-2.7400650
H	3.4775670	-0.2945320	1.5044050
H	3.2565530	-4.9031040	-0.7081500

C	1.2268240	3.1284250	0.7097680
C	1.1862420	3.9114860	-0.6483950
C	-0.0099160	3.5201230	-1.5118320
C	-1.1657140	3.9070950	-0.5814760
C	-0.7970660	5.3560850	-0.1604250
C	0.7224250	5.1945320	0.1022990
C	-1.3895590	5.5133860	1.2403230
H	-0.0084190	2.4665590	-1.7982910
N	-3.1471400	-0.4860030	-1.3730520
C	-3.9473600	0.7554480	-1.3612860
C	-3.9040920	1.2438920	-2.8100920
C	-2.5208070	0.7876710	-3.2770070
C	-2.3929590	-0.5943010	-2.6382000
N	-3.7915240	-1.0092120	1.1367610
C	-4.9387810	-1.8737250	0.8439340
C	-5.9468430	-1.5170280	1.9383220
C	-5.6677920	-0.0319810	2.1831890
C	-4.1421430	0.0535000	2.0908720
H	-1.0630280	6.4288220	1.7459760
H	-2.4842750	5.4732550	1.2531780
H	2.2044720	2.7214010	0.9804010
H	-0.0427070	4.1299790	-2.4232050
H	0.0988160	1.8164150	1.9622490
H	-1.0585360	6.1170960	-0.8998910
H	-2.1502640	3.8312910	-1.0531370
H	1.3148690	6.1060750	0.0046850
H	1.3898310	4.8193490	2.2573880
H	-0.9464230	3.9992710	2.8408630
H	-2.0834660	2.6590350	1.0193630
H	2.1386310	3.9647850	-1.1805480
H	-4.0543940	2.3234070	-2.8828720
H	-4.6841220	0.7500600	-3.3995600
H	-2.4144650	0.7603970	-4.3639650
H	-1.7517530	1.4507500	-2.8661960
H	-2.8475100	-1.3631550	-3.2768470
H	-1.3524550	-0.8731120	-2.4587310
H	-3.4981360	1.5085000	-0.6973140
H	-4.9660350	0.5600670	-1.0098880
H	-1.0697190	-5.3379770	1.1689990
H	-1.6779940	-3.1012860	1.7857810
H	-0.1815520	-2.7441440	0.8989380
H	-6.1235080	0.5684730	1.3879360
H	-3.6047420	-3.1519080	-1.4009390
H	-6.0484960	0.3283620	3.1414770
H	-3.3059710	-4.8973640	0.2551380
H	-3.6755420	-0.1365000	3.0657300
H	-2.0092420	-3.4553370	-2.1024940
H	-3.7945330	1.0303750	1.7458740
H	-5.7345460	-2.0938080	2.8447170
H	-5.3518040	-1.6640530	-0.1542780
H	-6.9759300	-1.7229930	1.6364480
H	-4.6507530	-2.9302680	0.8705610
H	5.8100780	1.5734440	0.3751460
H	6.5129840	1.3989380	-1.2359660
H	5.4688190	-0.7895330	0.1124230
H	3.3558860	-3.7676790	1.4562970
H	1.9384670	-2.9537320	2.1382310
H	2.6017560	-1.0815770	4.0536290
H	3.1534930	-3.4811310	4.1524570
H	4.7185430	-3.1864710	3.3751870
H	4.2996270	-1.0654470	4.5673680
H	3.5239640	0.2840250	2.3162610
H	5.0614020	-0.5946980	2.3229440
H	4.1270980	2.8992890	-0.5028140
H	2.2929740	1.5195040	-1.1535900
H	4.6809440	2.6286810	-2.1551140
H	4.3004710	-2.7186960	-1.0727020
H	3.2333470	-4.0331520	-1.5675580
H	3.4138290	-3.0775790	-3.7223410
H	3.8289250	-1.4850060	-3.0908000
H	1.5971810	-0.8160460	-3.0614850
H	0.7481950	-3.4377850	-1.7816890
H	0.2666740	-1.8122850	-1.2973320
H	3.2257000	0.8101830	-2.4809400
H	1.1497160	-2.3209440	-3.8694170
H	-0.3047120	-4.8018590	-0.3319560
H	-2.4753610	-5.6235550	-1.1320860
H	5.3417420	-0.6357990	-1.6497280

II-Pa$_2$

C	-2.8150570	-1.4022610	-1.0259130
C	-2.9228690	0.0909760	-1.3832660
C	-4.3339050	0.6522740	-1.2345620
C	-4.8409460	0.4336640	0.2058730
C	-3.7708870	-0.2776480	1.0269870
C	-3.3311070	-1.6252350	0.4267780
C	-2.4034800	0.4487470	1.0797420

II-Pb$_2$

C	-2.2420120	-1.5572480	-0.6543960
C	-2.8392660	-1.7832850	0.7729950
C	-2.4857470	-0.6336980	1.7209100
C	-3.1838420	0.5344980	1.0019700
C	-2.5778570	0.7636010	-0.4421140
C	-1.4556390	-0.2458870	-0.7846240
C	-4.2621940	-1.4235340	0.2354070

C	-1.8789180	0.6735650	-0.3960970
C	-1.3409130	-1.6132950	-0.5380280
C	-0.6820120	-0.2416890	-0.7455020
C	-1.8626790	-1.8380000	0.9182020
C	-1.5804660	-0.6272700	1.8116420
H	-0.5191710	-0.3957290	1.9060880
P	1.0091000	0.1099570	-0.0357710
N	1.8891020	-1.0470810	-0.9307020
P	2.6795140	-2.1792370	-0.1500180
C	1.6570730	-3.3270520	0.8329480
N	1.3096170	1.5688730	-0.8688050
P	1.6416930	2.8744730	-0.0307640
C	1.9647510	4.2521370	-1.1611680
C	0.3174750	3.4658850	1.0765060
C	3.1048300	2.7921600	1.0577990
C	3.5646070	-3.2254850	-1.3342090
C	3.9470180	-1.6150500	1.0365420
H	-0.7820670	-2.4444550	-0.9725050
H	-1.9884620	-0.7838920	2.8173360
H	-0.4922780	-0.1273960	-1.8227910
H	-4.1322150	-0.4242560	2.0518360
H	-2.4545170	1.3981370	1.6219760
H	-4.0475760	-2.4377440	0.5697570
H	-3.2064170	-2.0790020	-1.7896580
H	-2.5724470	0.2603110	-2.4076090
H	-1.6725410	1.7310010	-0.5836400
H	-1.6124480	-2.8045470	1.3619150
H	-4.9945100	0.1645530	-1.9602940
H	-4.3266070	1.7180290	-1.4888650
H	-5.7598140	-0.1633160	0.2119520
H	-5.0877790	1.3885930	0.6830940
H	2.8480540	-3.6708510	-2.0275310
H	4.1054920	-4.0193600	-0.8135140
H	4.2727680	-2.6146660	-1.8980990
H	3.4612010	-1.0207930	1.8145350
H	4.6733650	-0.9860400	0.5164470
H	4.4615470	-2.4621090	1.4971410
H	1.1129860	-2.7514420	1.5872880
H	2.2682040	-4.0867620	1.3270950
H	0.9297140	-3.8122240	0.1776990
H	-0.5801550	3.6687050	0.4873670
H	0.6157560	4.3721490	1.6098670
H	0.0882740	2.6780690	1.7996780
H	2.9444410	2.0119440	1.8061530
H	3.9828220	2.5317320	0.4619330
H	3.2762670	3.7464640	1.5621570
H	2.1840720	5.1639620	-0.6002180
H	2.8166750	4.0053510	-1.7981910
H	1.0866410	4.4166140	-1.7891920

C	-4.6059130	-0.0651140	0.8703560
C	-3.6296800	0.3140750	-1.4890580
C	-3.6704390	-1.1953940	-1.1871490
C	-4.9902660	0.9947370	-1.3709050
C	-5.5749430	0.7680070	0.0385160
H	-1.4120020	-0.4991320	1.8576780
P	0.2353090	-0.0760830	-0.0126950
N	0.9973760	-1.3060050	-0.9011240
P	2.0974820	-2.2913400	-0.3351110
C	3.5604650	-2.3610440	-1.4291460
C	4.3121550	-1.0313420	-1.4970190
N	0.6702550	1.3669680	-0.8122310
P	1.3820280	2.5825680	-0.0905380
C	2.1605370	3.6629120	-1.3368660
C	3.1990940	2.9406680	-2.1943780
C	0.2666660	3.7105090	0.8294790
C	-0.3829910	3.0492900	2.0455110
C	2.7007320	2.1223080	1.1010620
C	3.4058310	3.2761770	1.8139720
C	2.7437120	-1.9187830	1.3418140
C	3.7986090	-2.8906310	1.8715910
C	1.5101800	-4.0231490	-0.2614530
C	0.3910020	-4.2125240	0.7634670
H	-1.7406130	-2.4191260	-1.0986990
H	-2.9410640	-0.7882180	2.7063730
H	-1.2207910	-0.1103410	-1.8502920
H	-5.0194130	-0.2147170	1.8748920
H	-3.1782060	1.4647480	1.5784400
H	-5.0519060	-2.1740500	0.3197020
H	-4.0901570	-1.8051040	-1.9912970
H	-3.2253090	0.4873400	-2.4926450
H	-2.2664970	1.8036090	-0.5771480
H	-2.6959330	-2.7826330	1.1912140
H	-5.6606530	0.5969270	-2.1411930
H	-4.8776270	2.0644150	-1.5799560
H	-6.5414870	0.2545320	-0.0155270
H	-5.7581290	1.7224750	0.5444970
H	3.1844090	-2.6423370	-2.4184810
H	4.2177570	-3.1707580	-1.0951630
H	3.1385380	-0.8981870	1.3049930
H	1.8762840	-1.8788840	2.0078070
H	1.1540560	-4.2601000	-1.2698100
H	2.3549950	-4.6884850	-0.0545070
H	1.3455030	4.0459650	-1.9601380
H	2.6000070	4.5244250	-0.8232340
H	-0.4974320	4.0206560	0.1081970
H	0.8200680	4.6106790	1.1185780
H	3.4171430	1.5149430	0.5361160
H	2.2290590	1.4452180	1.8217050
H	2.7076650	3.8648310	2.4159860
H	4.1766740	2.8898170	2.4858440
H	3.8937560	3.9518310	1.1055590
H	-0.8846960	2.1184590	1.7642440
H	0.3570830	2.8112420	2.8156650
H	-1.1269620	3.7142040	2.4917820
H	2.7503070	2.0765020	-2.6907760
H	3.5954990	3.6112400	-2.9609130
H	4.0418840	2.5880940	-1.5917220
H	-0.4083650	-3.4823050	0.6042090
H	-0.0410740	-5.2130080	0.6822570
H	0.7612330	-4.0914590	1.7860390
H	5.1130340	-1.0796860	-2.2389700
H	3.6364690	-0.2186120	-1.7779560
H	4.7668480	-0.7787320	-0.5339160
H	4.1218690	-2.5855710	2.8702020
H	3.4082570	-3.9095460	1.9481080
H	4.6850570	-2.9159600	1.2312390

II-Pc$_2$

C	1.0202060	-2.7624170	0.4809550
C	1.4493380	-3.0638220	-0.9931080
C	1.8188870	-1.7828050	-1.7466430
C	3.0176040	-1.3107170	-0.9050970
C	2.5859800	-1.0069000	0.5857740
C	1.0821070	-1.2694120	0.8383920
C	2.8143100	-3.6269570	-0.4835740
C	3.8709550	-2.6029200	-0.9337680
C	3.1766710	-2.1090710	1.5026560
C	2.3922170	-3.3286410	0.9857350
C	4.6907510	-2.2766930	1.4146710
C	5.1069340	-2.5712590	-0.0412780
H	0.9990400	-1.0655960	-1.8057050
P	-0.2136050	-0.0926450	0.1825140
N	-1.5069400	-0.6237640	1.1542330
P	-2.9212270	-1.0233700	0.5608760
C	-3.5476590	0.0616370	-0.7769080
C	-4.9490680	-0.2388820	-1.3118050

II-Pd$_2$

C	3.4091720	0.6247250	-1.0465770
C	4.0754880	0.7374090	0.3628920
C	3.3715220	-0.1505900	1.3924050
C	3.6156030	-1.5353410	0.7669010
C	2.9403170	-1.6501480	-0.6602820
C	2.2215290	-0.3463250	-1.0855850
C	5.2809520	-0.1230630	-0.1372140
C	5.1539500	-1.4674380	0.6001180
C	4.0622230	-1.6631340	-1.7303450
C	4.6188520	-0.2393360	-1.5421990
C	5.1120260	-2.7557800	-1.5519700
C	5.7641100	-2.6406550	-0.1587480
P	0.5924010	0.1099530	-0.3003260
N	0.3008630	1.5292710	-1.1851990
P	-0.4312060	2.7948030	-0.5759920
C	-1.9310350	3.2513800	-1.5147820
C	-3.0664240	2.2352070	-1.3682980
C	-4.2850280	2.5928450	-2.2193490

C	-5.3460960	0.7370640	-2.4183450
N	0.3247500	1.3112540	0.9853750
P	0.7587700	2.6230790	0.2104090
C	1.6474050	3.7264000	1.3559660
C	2.9333310	3.1114810	1.9149200
C	3.6008470	4.0152950	2.9490440
C	1.8788640	2.3354590	-1.2102400
C	2.4051250	3.5720860	-1.9411480
C	3.3135320	3.1913840	-3.1090560
C	-0.6066740	3.6568570	-0.4499880
C	-1.3147800	3.0316660	-1.6576790
C	-2.6427270	3.7148570	-1.9742200
C	-3.0173710	-2.7265410	-0.1077060
C	-2.2132720	-2.9123560	-1.3980950
C	-2.0209050	-4.3845070	-1.7519310
C	-4.1706670	-0.9872940	1.8861250
C	-4.4048400	0.4101360	2.4655610
C	-5.3671620	0.3860650	3.6510480
H	0.1106850	-3.2670800	0.8166080
H	2.1487580	-2.0132270	-2.7666510
H	0.9231200	-1.1822100	1.9228810
H	4.1706700	-2.8049780	-1.9690550
H	3.5427110	-0.4555520	-1.3426140
H	3.0640830	-4.6648290	-0.7168690
H	2.3785790	-4.1806510	1.6701690
H	2.8971710	-1.8918610	2.5396740
H	2.8928780	-0.0016260	0.8884280
H	0.7974560	-3.7475560	-1.5414750
H	5.0038810	-3.0869360	2.0828480
H	5.1756190	-1.3657760	1.7831440
H	5.6313450	-3.5307450	-0.1128960
H	5.8015650	-1.8091060	-0.4117160
H	-2.8084390	0.0067640	-1.5844840
H	-3.4963150	1.0858410	-0.3882050
H	-2.6166910	-3.3708290	0.6845970
H	-4.0653170	-3.0121480	-0.2563200
H	-5.1052590	-1.4089220	1.4984510
H	-3.8112740	-1.6692710	2.6655730
H	1.8586960	4.6709470	0.8414190
H	0.9486070	3.9548610	2.1692650
H	1.3315440	1.6795390	-1.8986730
H	2.7099550	1.7353670	-0.8219720
H	-0.2296570	4.6570920	-0.6946360
H	-1.3100750	3.7738360	0.3834470
H	-0.6565430	3.0822260	-2.5329230
H	-1.4889240	1.9674060	-1.4664850
H	1.5669840	4.1736860	-2.3117080
H	2.9592530	4.2083370	-1.2413090
H	2.6963970	2.1395100	2.3600930
H	3.6345510	2.9174230	1.0941540
H	-3.4430090	0.8352930	2.7712780
H	-4.8054800	1.0683610	1.6852760
H	-4.9897380	-1.2645600	-1.6965020
H	-5.6799480	-0.1820740	-0.4967770
H	-2.7205960	-2.3971290	-2.2221660
H	-1.2319790	-2.4350020	-1.2910060
H	-3.1230300	3.2561730	-2.8436480
H	-3.3333940	3.6327000	-1.1277690
H	-2.5006530	4.7796180	-2.1867030
H	3.6917290	4.0798830	-3.6223910
H	4.1732910	2.6101520	-2.7599280
H	2.7728150	2.5818850	-3.8402880
H	4.5177910	3.5608390	3.3348690
H	3.8624870	4.9851740	2.5133080
H	2.9327170	4.1989860	3.7967510
H	-1.4585600	-4.8997250	-0.9655960
H	-2.9833170	-4.8943430	-1.8649660
H	-1.4673790	-4.4953350	-2.6890710
H	-5.5265670	1.3923550	4.0486800
H	-6.3412590	-0.0191070	3.3579050
H	-4.9749450	-0.2385420	4.4601810
H	-6.3479640	0.5189390	-2.7986460
H	-5.3400790	1.7675330	-2.0482490
H	-4.6448020	0.6804050	-3.2573790

C	-5.4168770	1.5777580	-2.0817490
N	-0.3489980	-1.0890450	-1.0700680
P	-1.3599560	-2.0111480	-0.2723740
C	-2.5505390	-2.7616820	-1.4288410
C	-3.4725630	-1.7389620	-2.0963130
C	-4.4619860	-2.3618290	-3.0858890
C	-3.7994100	-3.0104260	-4.3003820
C	-0.5968070	-3.4485120	0.5695730
C	0.2959440	-3.0484020	1.7489170
C	1.2392810	-4.1665420	2.1986510
C	0.5211110	-5.4259170	2.6803590
C	-2.3494390	-1.1614790	1.0179040
C	-3.4074290	-2.0042470	1.7309690
C	-4.1651530	-1.2032130	2.7907190
C	-5.2502150	-2.0224850	3.4848010
C	-0.9791800	2.6464130	1.1661100
C	-1.7430350	3.8437960	1.7358850
C	-2.2129410	3.6128140	3.1767470
C	-3.2053570	2.4590160	3.3196270
C	0.6287870	4.2806460	-0.6519140
C	1.8886500	4.1353950	0.2046550
C	2.8999070	5.2569100	-0.0311940
C	4.1659600	5.0860680	0.8050510
H	2.3190530	0.0979360	1.5339890
H	3.2203510	1.5712740	-1.5561400
H	3.8730340	-0.0901660	2.3656490
H	1.9354400	-0.4707070	-2.1399110
H	5.6125550	-1.3948940	1.5935560
H	3.3058940	-2.3632520	1.4115640
H	6.2801910	0.3179360	-0.1043460
H	5.2045200	0.1310190	-2.3874040
H	3.6040710	-1.7609200	-2.7209400
H	2.2927120	-2.5308220	-0.7172030
H	4.2862460	1.7539060	0.7044030
H	5.8636970	-2.6667360	-2.3444800
H	4.6383940	-3.7352790	-1.6820340
H	6.8470480	-2.4960290	-0.2429170
H	5.6172140	-3.5599920	0.4191220
H	-1.9473780	-3.2942680	-2.1717750
H	-3.1356760	-3.5158200	-0.8897120
H	-2.8601170	-0.9885550	-2.6109110
H	-5.0792450	-3.1020100	-2.5612930
H	-5.1475170	-1.5778770	-3.4290660
H	-3.1821330	-3.8685590	-4.0174230
H	-4.5502880	-3.3672410	-5.0110430
H	-3.1558720	-2.2937130	-4.8218800
H	-1.6280920	-0.7528330	1.7356420
H	-2.8138560	-0.2980340	0.5250910
H	-4.1250470	-2.3992870	1.0005950
H	-2.9372660	-2.8741250	2.2070000
H	-3.4510790	-0.8290150	3.5349340
H	-4.6115670	-0.3180160	2.3200010
H	-4.8214670	-2.8981870	3.9834260
H	-5.7739060	-1.4292460	4.2397190
H	-5.9927180	-2.3801360	2.7636300
H	-1.3808700	-4.1473140	0.8811200
H	-0.0026520	-3.9541080	-0.2023010
H	0.8944420	-2.1719840	1.4724100
H	-0.3333110	-2.7410080	2.5942470
H	1.9102950	-4.4194180	1.3668600
H	1.8770340	-3.7814910	3.0031300
H	1.2335770	-6.1672490	3.0532370
H	-0.1758870	-5.1903730	3.4922230
H	-0.0522150	-5.8946760	1.8745060
H	0.8985540	4.4048540	-1.7075720
H	0.0503990	5.1648690	-0.3609470
H	1.6135260	4.1170120	1.2672630
H	2.3595310	3.1685380	-0.0127300
H	3.1627840	5.2839240	-1.0960690
H	2.4316230	6.2226870	0.1964100
H	3.9306450	5.0634510	1.8744390
H	4.6738700	4.1479180	0.5562680
H	4.8715630	5.9040650	0.6336070
H	-0.0893970	2.4321090	1.7680560
H	-1.5886900	1.7382100	1.2063380
H	-1.1075490	4.7363940	1.7052350
H	-2.6193260	4.0621790	1.1124700
H	-1.3404940	3.4297880	3.8162600
H	-2.6770150	4.5361020	3.5414020
H	-3.5850490	2.3907750	4.3430150
H	-4.0631200	2.5950190	2.6510270
H	-2.7468780	1.4954240	3.0778090
H	-1.6238720	3.3312420	-2.5640700
H	-2.2598890	4.2488480	-1.2005100
H	-2.7016640	1.2382410	-1.6450090
H	-3.3772520	2.1726050	-0.3164860
H	-4.6436210	3.5885650	-1.9298380

				H	-3.9814400	2.6670800	-3.2708800
				H	-6.2939490	1.8769860	-2.6626020
				H	-5.1011300	0.5916640	-2.4362010
				H	-5.7255700	1.4722500	-1.0358730
				H	-4.0376440	-1.2044740	-1.3227700
**II-Pe$_2$**				**II-Pf$_2$**			
C	-4.9065000	-2.1414170	-1.8497810	C	1.1455700	-1.2107100	-0.7062370
C	-3.3814860	-2.0187070	-1.8523550	C	2.6466570	-0.9873430	-0.4056910
C	-3.0359260	-2.1707520	-0.3609800	C	3.2511590	-1.8750280	-1.5234180
C	-4.0425480	-3.2191690	0.1812380	C	2.4812410	-3.1822280	-1.2616070
C	-5.1637650	-3.3096830	-0.8863770	C	1.1038950	-2.7457220	-0.6535050
P	-1.2543710	-2.4244960	0.0027370	C	2.9088940	-3.7670230	0.1172300
C	-1.2355010	-2.6169050	1.8384080	C	3.9527720	-2.8408820	0.7652650
C	-2.0102640	-1.4926750	2.5672940	C	5.1876660	-2.6148910	-0.1001730
C	-1.3325100	-1.3906710	3.9355590	C	4.7671790	-2.0386050	-1.4679770
C	0.1501770	-1.5728880	3.6030520	C	3.0833800	-1.5798070	0.9948610
C	0.1759640	-2.6483990	2.5013130	C	1.8919090	-2.2268620	1.7240040
N	-0.3658140	-1.2986180	-0.6557640	C	1.5377130	-3.3346640	0.7287260
P	0.4864360	-0.0378350	0.0980390	H	1.0615460	-1.5477410	1.9235100
N	-0.0416300	1.3872280	-0.6735980	P	-0.1544870	-0.1907860	0.1722450
P	-0.9265560	2.5018910	0.0223110	N	-1.4812990	-0.7755980	-0.7181940
C	-2.6869920	2.0272520	0.3028300	P	-2.9325140	-0.9904490	-0.1142550
C	-3.4279130	1.6504270	-1.0113720	C	-2.9231240	-1.5363720	1.6599170
C	-4.4872690	2.7421120	-1.2004070	C	-4.2865510	-1.6331950	2.3492760
C	-4.9044670	3.0734340	0.2334070	N	0.2029350	1.3106550	-0.5286510
C	-3.5712070	3.1017850	0.9948150	P	0.7050690	2.6774830	0.0756450
C	2.0692960	-0.2046570	-0.8790970	C	1.0170770	2.7614590	1.9016400
C	3.2371890	0.7787630	-0.6336060	C	2.1092560	1.7666710	2.3181280
C	3.9781540	0.5048330	0.7379670	C	2.3332980	3.1251640	-0.6959690
C	3.3391970	-0.6742850	1.4932710	C	2.1855090	3.3352540	-2.2067040
C	3.5791980	-1.8030040	0.4875900	C	-0.5433780	4.0116560	-0.2475870
C	2.8455240	-1.5283840	-0.8650920	C	-1.1920240	3.8709800	-1.6277050
C	4.1712040	0.3422250	-1.7912780	C	3.1376990	4.2578340	-0.0486190
C	5.5468390	1.0023570	-1.7938870	C	-3.8014480	-2.3047800	-1.0861610
C	6.2646490	0.7340690	-0.4548300	C	-3.3297100	-2.3439500	-2.5435310
C	5.3714150	-0.1080050	0.4498810	C	-3.9907140	0.5299990	-0.1526710
C	4.2181260	-1.1742050	-1.5306480	C	-3.4039060	1.5837050	0.7937000
C	4.9466580	-1.4463300	-0.1809540	C	-5.3320270	-2.2534080	-0.9997400
H	2.2856300	-0.5287530	1.7330930	C	-4.0837820	1.0783300	-1.5809330
C	-0.8086680	-4.0804380	-0.6616440	C	-2.1239750	-2.8337220	1.8278110
C	-0.5898280	-4.0976620	-2.2023100	C	-0.0725280	5.4452050	0.0114120
C	0.7197210	-4.8850470	-2.4373940	C	-0.2577120	2.5324730	2.7237420
C	0.9718850	-5.6384790	-1.1276420	H	0.8934800	-4.1216200	1.1281190
C	0.4980120	-4.6454180	-0.0654070	H	0.9814780	-0.9172260	-1.7534040
C	-0.3658320	3.1057030	1.6760940	H	2.2264890	-2.6510040	2.6781960
C	1.1820610	3.2530650	1.7893180	H	2.9680410	-1.4580120	-2.4963810
C	1.5899970	2.4664190	3.0504170	H	2.9482630	0.0598530	-0.4971220
C	0.3160960	2.4025920	3.8960440	H	2.4751960	-3.8789190	-2.1037580
C	-0.7716200	2.1757140	2.8450850	H	3.1725340	-4.8273060	0.1361690
C	-0.9506980	3.9816370	-1.0637060	H	4.2559720	-3.2421270	1.7397460
C	0.3292350	4.8358680	-0.9808970	H	3.5986630	-0.8223250	1.5931120
C	0.2577930	5.6763950	-2.2565530	H	0.2011640	-3.1806750	-1.0860210
C	-0.1971620	4.6681030	-3.3206500	H	5.7243750	-3.5624650	-0.2218820
C	-1.1039950	3.6568560	-2.5736660	H	5.8726620	-1.9341820	0.4175570
H	3.4597170	-2.8117140	0.8919880	H	5.0897310	-2.6942360	-2.2847340
H	1.7187350	-0.0390960	-1.9083960	H	5.2409690	-1.0665160	-1.6449090
H	3.8841060	-0.8583890	2.4269610	H	-3.4480140	1.2764310	1.8424210
H	3.6700460	0.5457250	-2.7443550	H	-2.3572750	1.7787780	0.5390490
H	2.9339270	1.8265210	-0.7140730	H	-3.9577590	2.5230520	0.6978030
H	4.5440490	-1.7701480	-2.3870670	H	-4.9950970	0.2542370	0.1883080
H	5.7307870	-2.2073250	-0.1950130	H	-4.6375820	0.4115750	-2.2468340
H	5.8814930	-0.2888020	1.4036710	H	-3.0856120	1.2221880	-2.0024730
H	4.0368570	1.4193490	1.3366420	H	-4.5967940	2.0451570	-1.5766620
H	2.2871530	-2.3697500	-1.2779860	H	-2.3434460	-0.7379630	2.1401630
H	7.2140240	0.2112360	-0.6166800	H	-4.8889660	-0.7285540	2.2307680
H	6.5101770	1.6736920	0.0525730	H	-4.8640820	-2.4825660	1.9726350
H	6.1318320	0.6150060	-2.6358290	H	-4.1400470	-1.7929450	3.4223030
H	5.4302600	2.0782200	-1.9660950	H	-1.1500320	-2.7722140	1.3366310
H	1.5446010	-4.1865820	-2.6177880	H	-1.9566950	-3.0273280	2.8921170
H	0.6588650	-5.5463190	-3.3058310	H	-2.6640700	-3.6944180	1.4191310
H	0.3591360	-6.5472700	-1.0870210	H	-3.4644650	-3.2335970	-0.6087500
H	2.0168990	-5.9337730	-0.9971450	H	-2.2404160	-2.3581010	-2.6007190
H	0.3482830	-5.0957210	0.9192890	H	-3.7273590	-3.2394840	-3.0315990
H	1.2427940	-3.8481590	0.0390110	H	-3.6848840	-1.4729560	-3.1025090
H	-0.5097890	-3.0835320	-2.6011020	H	-5.7010100	-2.2198330	0.0272620
H	-1.4367740	-4.5837060	-2.6947490	H	-5.7325290	-1.3836420	-1.5287960
H	-1.6811860	-2.2012540	4.5869710	H	2.8926210	2.1922680	-0.5512130
H	-3.0821480	-1.6972250	2.6299910	H	-2.1010180	4.4795120	-1.6704600
H	-1.8842200	-0.5437410	2.0359450	H	-0.5245180	4.2114960	-2.4241160
H	-6.1623980	-3.2793910	-0.4433120	H	-1.4552430	2.8304410	-1.8243600
H	0.3561990	-3.6395390	2.9276730	H	0.6248340	5.7793850	-0.7624000
H	-5.0800350	-4.2554580	-1.4317540	H	4.1077470	4.3372100	-0.5500060
H	0.7557770	-1.8489590	4.4706750	H	3.3327670	4.0819190	1.0120530
H	-3.5797160	-4.1989300	0.3330430	H	2.6403490	5.2251520	-0.1454250
H	0.9804290	-2.4530070	1.7889860	H	3.1706110	3.3134820	-2.6828640
H	-4.4325700	-2.9066070	1.1533310	H	1.7344000	4.3075490	-2.4288930

H	-5.3258570	-2.3108380	-2.8452480
H	-3.0080440	-1.0816710	-2.2714200
H	-5.3498140	-1.2182910	-1.4552660
H	-2.9523050	-2.8404990	-2.4359590
H	1.8827370	1.4503110	2.7655430
H	2.4352000	2.9236010	3.5725710
H	1.6951180	2.8646760	0.9061720
H	1.2159840	4.1917590	-1.0125690
H	0.3795720	5.4341600	-0.0672500
H	-0.7103440	5.1470400	-4.1585990
H	-0.4939700	6.4651720	-2.1330070
H	1.2076470	6.1586470	-2.5037070
H	0.6760410	4.1495590	-3.7299820
H	-2.1486160	3.7425590	-2.8837090
H	-0.7818020	2.6316350	-2.7712390
H	0.3405770	1.6100690	4.6505700
H	-0.7243690	1.1322740	2.5208340
H	0.1508590	3.3557330	4.4134330
H	-2.7572620	1.5420740	-1.8672880
H	-3.9271980	0.6853960	-0.8784010
H	-5.3184480	2.4165920	-1.8316270
H	-4.0431770	3.6293080	-1.6684740
H	-5.4584520	4.0119780	0.3205040
H	-3.6920830	2.9033700	2.0621180
H	-3.1272780	4.1001500	0.9091660
H	-1.7811680	2.3765440	3.2118480
H	-5.5418990	2.2717520	0.6248700
H	-1.5451680	-0.4420650	4.4383950
H	0.5461840	-0.6364250	3.1958240
H	1.4470280	4.3099970	1.8831110
H	-2.6145450	1.1408840	0.9424590
H	-1.7917010	4.6061910	-0.7388750
H	-0.8359000	4.0852890	1.8242970
H	-3.2495970	-1.2061150	0.1189710
H	-1.6245630	-4.7651250	-0.4034380
H	-1.7422950	-3.5705380	2.0306610

H	1.3770100	3.7789800	2.0979940
H	-0.6843840	1.5489070	2.5060830
H	-0.0135670	2.5705870	3.7903450
H	-1.0206450	3.2924430	2.5357080
H	3.0402190	1.8987040	1.7595160
H	1.7685500	0.7391050	2.1660890
H	2.3385340	1.8954580	3.3806520
H	-0.9331440	6.1209060	-0.0131090
H	0.4121710	5.5636770	0.9846920
H	-1.3201390	3.7752210	0.4896610
H	1.5676680	2.5538490	-2.6562890
H	-5.7517120	-3.1459090	-1.4736470

II-Pg_2

C	-5.2590320	-2.2852980	0.0794580
C	-3.9967900	-2.6162580	0.8686310
C	-3.0309350	-1.4235190	1.0747410
C	-2.6349900	-0.8252450	-0.3356040
C	-3.3585000	-1.6417290	-1.4354470
C	-4.8764940	-1.7029410	-1.2970200
C	-1.1720370	-1.1458670	-0.7251150
C	-1.2311020	-2.6797800	-0.7056300
C	-1.6272890	-3.2723260	0.6850980
C	-1.8478950	-2.1668090	1.7208330
C	-2.6669940	-3.0048340	-1.2454370
C	-3.0571380	-3.5943240	0.1426650
H	-0.9662220	-1.5461280	1.8883620
P	0.2068300	-0.1901760	0.0982150
N	-0.0422980	1.2952060	-0.6873620
P	-0.6285840	2.5948030	-0.0551730
N	0.4431000	3.8663420	-0.3197020
C	1.4405980	3.7691340	-1.3732620
N	1.4911010	-0.8896350	-0.7669740
P	2.9510330	-1.0169790	-0.2101210
N	3.5072570	0.0603410	0.9651700
C	3.4557020	1.4831500	0.6359860
N	3.2114690	-2.5534970	0.4221250
C	2.1369450	-3.2929950	1.0648970
N	4.0509240	-0.7499690	-1.4411830
C	5.4543620	-0.4261060	-1.2473410
C	4.5414940	-3.0242970	0.7696180
N	-2.1651300	2.9049750	-0.6784760
C	-2.9696430	4.0424960	-0.2595120
N	-0.9246710	2.7392700	1.5967310
C	0.2085580	2.8762720	2.5038980
C	-2.5052670	2.4178560	-2.0065760
C	-2.0039620	1.9492080	2.1814090
C	0.0766680	5.2342270	0.0119760
C	3.7271940	-1.1874010	-2.7897230
C	3.2010490	-0.2057430	2.3654010
H	-0.3848620	-3.1616770	-1.1987210
H	-2.1568550	-2.5902090	2.6839460
H	-1.0446680	-0.8434130	-1.7747180
H	-4.2729180	-3.0207420	1.8498110
H	-3.4588910	-0.6490680	1.7181960
H	-3.3920050	-4.6344030	0.1531890
H	-2.7541810	-3.6792960	-2.1010120
H	-3.1015680	-1.2191250	-2.4135320
H	-2.8646900	0.2424690	-0.3832100
H	-1.0178050	-4.1109560	1.0304160
H	-5.2901030	-2.3122190	-2.1086240
H	-5.2891400	-0.6955600	-1.4236780
H	-5.8651460	-3.1912690	-0.0328440

II-Ph_2

C	-0.1554310	4.1656860	-1.3082610
N	-0.1009780	3.0849560	-0.3105160
C	-1.1690760	3.2324330	0.6952110
C	-1.6776900	4.6583570	0.4856090
C	-1.4847310	4.8691590	-1.0168180
P	1.2423160	2.1724140	0.0767260
N	2.1055240	2.2365230	-1.3694850
C	1.5281170	1.7326810	-2.6329570
C	2.5445090	0.7122320	-3.1393850
C	3.8707380	1.3125360	-2.6712650
C	3.5281360	1.8432230	-1.2772920
N	0.9400340	0.7967090	0.7693290
P	0.2894160	-0.5385790	-0.0658660
N	-1.1650380	-0.8617520	0.7382430
P	-2.6715290	-0.8929590	0.3540170
N	-3.1917440	0.3847690	-0.6016140
C	-2.3787890	0.8396780	-1.7447720
C	-3.3239730	1.7428240	-2.5502880
C	-4.3812880	2.1684180	-1.5287810
C	-4.5619100	0.8971580	-0.7042280
C	1.3316580	-1.8279480	0.7927750
C	2.8521510	-1.8682150	0.5176890
C	3.1923180	-2.4758670	-0.9031930
C	1.9152140	-2.8749090	-1.6668930
C	1.3539540	-3.9359770	-0.7182220
C	1.0114500	-3.3264330	0.6800130
C	2.6164490	-4.6279570	-0.1121800
C	3.8189330	-3.8802420	-0.7158230
C	2.2777570	-4.0269610	1.2843500
C	3.2672980	-2.8921720	1.6042870
C	5.0613790	-3.9114760	0.1676640
C	4.7290060	-3.3270800	1.5561470
H	1.2224060	-2.0520090	-1.8477220
N	2.1723740	3.0359450	1.1546110
C	2.5166070	4.4419480	0.9173530
C	3.5191280	4.7648040	2.0291430
C	4.2161220	3.4223830	2.2638030
C	3.0706000	2.4163980	2.1396890
N	-3.1138480	-2.3923330	-0.2889840
C	-2.1938350	-3.1049330	-1.1800470
C	-2.7501630	-2.9135590	-2.6122340
C	-4.1938690	-2.3953440	-2.4090650
C	-4.4352670	-2.5586360	-0.8995830
N	-3.7185330	-0.7764630	1.6537710
C	-3.9602500	-1.9723320	2.4874490
C	-4.3798660	-1.3940760	3.8381790
C	-3.5233620	-0.1315480	3.9380160
C	-3.5759970	0.4198770	2.5123100
H	0.0371930	-3.6040990	1.0871300

H	-5.8664070	-1.5739660	0.6501600
H	-3.5592130	2.1142100	-2.0265530
H	-2.3783760	2.4557830	3.0778840
H	-2.3581640	3.1901960	-2.7759250
H	-2.8245090	1.8457890	1.4709980
H	-1.6600960	0.9442060	2.4639100
H	-2.7226140	4.3280060	0.7633940
H	-2.8299790	4.9145570	-0.9139860
H	-4.0298800	3.7649430	-0.2886410
H	-0.1241050	3.3737420	3.4214950
H	0.6322510	1.8985090	2.7736710
H	0.9901360	3.4799600	2.0426960
H	0.9799560	5.7971370	0.2726060
H	-0.5897630	5.2506990	0.8756390
H	-0.4152080	5.7501710	-0.8252840
H	1.6622200	2.7198200	-1.5641660
H	2.3587680	4.2775640	-1.0550790
H	2.4555150	1.9095630	0.7902090
H	3.7317480	1.6338950	-0.4092340
H	4.1725660	2.0236780	1.2632980
H	3.8910980	0.3654020	2.9953800
H	3.3349710	-1.2634940	2.5939510
H	2.1724050	0.0790410	2.6255890
H	2.1975910	-4.3512460	0.7840470
H	2.1888890	-3.2306040	2.1620510
H	1.1734350	-2.9028700	0.7380330
H	5.2991100	-2.5338250	0.1596650
H	5.7281500	0.4286580	-1.8784850
H	4.6070440	-4.1016860	0.5817000
H	5.6404720	-0.1616450	-0.2064370
H	4.7850740	-2.8508460	1.8282900
H	4.0709530	-0.4370000	-3.5112710
H	2.6470670	-1.2947950	-2.8876360
H	4.2121150	-2.1432380	-3.0337370
H	6.1053920	-1.2677430	-1.5234990
H	1.0975330	4.2384570	-2.3072510
H	-1.8855560	1.5533970	-2.2448340

H	2.1849490	-3.3163560	-2.6337020
H	1.2032670	-1.5452360	1.8472540
H	4.0597470	-4.2940500	-1.7023460
H	3.8426270	-1.8002090	-1.4686830
H	2.6877970	-5.7168100	-0.1698200
H	2.1315210	-4.7419820	2.0979570
H	3.0489760	-2.4667710	2.5902410
H	3.3314190	-0.8945750	0.6548780
H	0.5839770	-4.5786980	-1.1515270
H	4.9123890	-4.0648900	2.3454960
H	5.3686440	-2.4663670	1.7812550
H	5.4211960	-4.9430390	0.2521440
H	5.8629110	-3.3414220	-0.3152940
H	2.9861940	5.0730370	2.9348050
H	4.2032410	5.5681550	1.7477780
H	4.9562750	3.2422880	1.4761850
H	4.7236270	3.3612800	3.2291470
H	3.4048560	1.4301110	1.8095200
H	2.5561800	2.2847130	3.1004140
H	1.6266630	5.0785730	0.9658000
H	2.9713950	4.5779350	-0.0751130
H	2.4904840	0.5644310	-4.2205720
H	1.4254270	2.5581140	-3.3498870
H	0.5415230	1.2930350	-2.4690910
H	-2.2942100	4.3811240	-1.5697700
H	4.1507410	2.6947670	-0.9839780
H	-1.4648840	5.9208990	-1.3100900
H	4.1656450	2.1364860	-3.3299490
H	0.6888270	4.8571150	-1.2017480
H	3.6711370	1.0481230	-0.5325880
H	-0.1299770	3.7650960	-2.3250000
H	-1.0568820	5.3647830	1.0474320
H	-1.9657930	2.4980240	0.5186980
H	-2.7138050	4.7826480	0.8097500
H	-0.7822730	3.0717080	1.7056990
H	-5.3129280	2.5068390	-1.9876450
H	-3.9998960	2.9714040	-0.8880750
H	-5.2307540	0.2035920	-1.2356600
H	-2.2008850	-4.1688950	-0.9109040
H	-1.1741600	-2.7400680	-1.0564630
H	-4.2603730	-1.3367730	-2.6799610
H	-2.1480290	-2.1923670	-3.1720610
H	-2.7282830	-3.8545990	-3.1676070
H	-4.9295550	-2.9384810	-3.0069140
H	-5.1559570	-1.8536390	-0.4810600
H	-4.7966330	-3.5716390	-0.6790080
H	-3.8006510	1.1721160	-3.3545640
H	-2.0145670	-0.0002550	-2.3490310
H	-2.7943980	2.5823560	-3.0076170
H	-2.6759630	0.9849330	2.2537650
H	-4.4412550	1.0826330	2.3824850
H	-3.8843210	0.5861350	4.6780410
H	-2.4923000	-0.4003250	4.1920950
H	-4.2154730	-2.0992770	4.6558600
H	-4.7297210	-2.6063010	2.0392910
H	-3.0498120	-2.5753330	2.5990970
H	-1.5041690	1.3926680	-1.3887100
H	-5.4426700	-1.1289590	3.8230730
H	2.3720310	-0.2493710	-2.6450600
H	4.6904200	0.5907840	-2.6425930
H	-4.9868370	1.0804800	0.2834990

III-Pa2

C	1.2856310	1.0706200	-2.0148430
C	1.9365920	1.8725440	-0.8873570
C	3.4402500	1.6041400	-1.0955950
C	3.5531600	0.2831910	-1.9184030
C	2.1146430	-0.2053390	-2.1796040
C	1.6165920	1.5518430	0.6045990
C	2.9784030	1.2325230	1.3233740
C	4.1213850	1.3477170	0.2634530
C	0.5556120	0.5086350	1.0949560
C	1.3229170	-0.5009440	1.9785620
C	2.7563050	-0.0077890	2.2450070
C	-0.2487210	-0.4630060	0.2019620
C	0.7382670	-1.5791860	-0.1943720
C	1.4409180	-1.8259190	1.1583280
C	2.9419290	-2.0920110	0.9477400
C	3.2424860	-1.7768710	-0.5522080
C	1.8781980	-1.4461630	-1.2619030
C	4.3025660	-0.7352370	-1.0360760
C	3.5783000	-1.2964950	2.0969790
C	5.0596210	0.1757550	-0.0589480
P	-1.6887370	0.0777470	-0.8892110
N	-2.6023530	0.8325490	0.3508950
P	-2.9499480	2.3771750	0.2656230
C	-4.0138230	2.8307760	1.6594330

III-Pb2

C	0.4865160	-0.0192890	0.9538210
C	1.4307750	1.1956680	1.0791430
C	2.3964100	0.6803950	2.1701120
C	2.3780110	-0.8812480	2.0927510
C	1.4006880	-1.2619980	0.9589270
C	3.8086750	-1.3315230	1.7495690
C	4.6479600	-0.1323500	2.2148070
C	3.8387220	1.1277790	1.8740900
C	2.3356720	1.7841750	-0.0585840
C	3.8265390	1.7483770	0.4415010
C	2.2896380	-1.7507380	-0.2368260
C	3.7808140	-1.8036990	0.2613730
C	4.7202200	1.2487450	-0.7395070
C	3.7641930	0.8681380	-1.8873650
C	2.3300480	1.3171640	-1.5469300
C	3.7427160	-0.6891710	-1.9658570
C	2.2967590	-1.1311760	-1.6693040
C	1.4798970	0.1238630	-1.9873770
C	4.6875840	-1.2100020	-0.8647430
C	5.6044680	-0.0015810	-0.6255350
H	0.4627250	0.1182730	-1.6034560
P	-1.1621170	0.0231950	0.0611500
N	-1.8460780	-1.3137960	0.8667350
P	-2.5781470	-2.5124960	0.1378520

H	0.2168900	0.9108250	-1.9070480
N	-2.4227940	-1.4239310	-1.1508260
P	-3.4911210	-2.1406640	-0.2269960
C	-5.1383440	-1.3599580	-0.1904490
C	-3.7829570	-3.8166950	-0.8583830
C	-3.0925310	-2.3715510	1.5407530
C	-3.8465130	2.9267770	-1.2255260
C	-1.5245550	3.5111840	0.3593480
H	0.1421970	-2.4517650	-0.4763480
H	-0.1719260	1.0587910	1.6985390
H	3.1656340	-3.1524310	1.1017950
H	2.8522080	0.3413470	3.2780590
H	-0.8612870	-0.9725520	0.9612040
H	4.6569090	-1.1724760	2.0526330
H	3.3892630	-1.8805800	3.0078570
H	5.5189480	-0.3164130	0.7938880
H	5.8927130	0.6055630	-0.6314470
H	1.4170700	1.6544420	-2.9365460
H	4.7352030	2.2077590	0.5493650
H	1.7156380	2.9344000	-1.0410900
H	3.9219960	2.4262310	-1.6333710
H	4.0880480	0.4671200	-2.8550000
H	5.0391710	-1.2839890	-1.6314150
H	2.0143090	-0.5517690	-3.2132380
H	0.9619380	-2.6463200	1.7021880
H	0.7846460	-0.6868360	2.9131410
H	1.2640500	2.5012490	1.0175440
H	3.1676800	2.0569070	2.0165110
H	3.5885090	-2.7282540	-0.9657610
H	1.6836380	-2.2656890	-1.9594610
H	-2.8527790	-4.3873840	-0.8117680
H	-4.5477890	-4.3233260	-0.2646090
H	-4.1124050	-3.7590510	-1.8979910
H	-2.9688960	-1.3849890	1.9927690
H	-2.1571490	-2.9290710	1.6349890
H	-3.8927380	-2.9154280	2.0507070
H	-5.8427170	-1.9528090	0.3991960
H	-5.5121990	-1.2620190	-1.2124310
H	-5.0273410	-0.3659520	0.2472540
H	-4.0761310	3.9940260	-1.1758150
H	-3.2275910	2.7309780	-2.1045030
H	-4.7748130	2.3575380	-1.3152830
H	-1.8369880	4.5562120	0.2872880
H	-1.0034120	3.3509000	1.3062860
H	-0.8358980	3.2800110	-0.4587770
H	-3.4991410	2.6008660	2.5946900
H	-4.9386770	2.2517860	1.6104050
H	-4.2516520	3.8967110	1.6289100

C	-1.4703550	-3.7759210	-0.5963530
C	-0.6970050	-3.2511790	-1.8060370
N	-1.8049520	1.3576610	0.8975660
P	-2.6156180	2.5308910	0.2108560
C	-1.5814660	3.9398550	-0.3394330
C	-0.6521930	3.5579790	-1.4918910
C	-3.7740630	3.2633070	1.4146520
C	-4.8386440	2.2743810	1.8902250
C	-3.6172020	2.0609140	-1.2531560
C	-4.4206910	3.1887710	-1.9010730
C	-3.5671680	-3.4464100	1.3526900
C	-4.6497220	-2.6026060	2.0249870
C	-3.7264200	-2.0310160	-1.2102450
C	-4.4554720	-3.1752450	-1.9150070
H	0.7812070	-2.1121890	1.2589510
H	0.8303740	2.0254400	1.4618090
H	4.0897340	-2.1996040	2.3543890
H	4.1394660	1.9230170	2.5636570
H	0.0587590	-0.0632980	1.9692030
H	5.6897890	-0.1292610	1.9060700
H	4.6694150	-0.1882670	3.3116360
H	6.2458300	-0.0547240	0.2498500
H	6.2870250	0.0332200	-1.4854910
H	1.3946870	0.1791750	-3.0815780
H	5.3550030	2.0896420	-1.0364320
H	2.0527690	2.1857360	-2.1532840
H	4.0986360	1.2897110	-2.8400450
H	4.0656480	-1.0216400	-2.9570020
H	5.3000970	-2.0326110	-1.2473170
H	1.9969700	-1.9255110	-2.3603970
H	2.0470920	-1.2986100	3.0487440
H	2.0729980	1.0088340	3.1626640
H	2.0474680	2.8386340	-0.1032360
H	4.1318090	2.7876970	0.5917400
H	4.0593980	-2.8603020	0.3054810
H	1.9745810	-2.7871100	-0.3914560
H	-3.9997520	-4.3184760	0.8511640
H	-2.8560920	-3.8252680	2.0943950
H	-3.1310650	-1.4465670	-1.9198300
H	-4.4395790	-1.3299530	-0.7631410
H	-0.7804830	-4.0605630	0.2058000
H	-2.0526320	-4.6671630	-0.8542890
H	-4.2297240	4.1538580	0.9693750
H	-3.1598070	3.6042950	2.2546640
H	-2.9211790	1.6122910	-1.9691690
H	-4.2755200	1.2496310	-0.9246580
H	-1.0002800	4.2389090	0.5397060
H	-2.2219040	4.7868710	-0.6074040
H	-3.7558950	-3.8658160	-2.3947540
H	-5.0725670	-3.7504880	-1.2186880
H	-5.1152320	-2.7809270	-2.6922590
H	-5.1593020	-3.1813850	2.7993840
H	-4.2119430	-1.7155190	2.4896030
H	-5.4044650	-2.2727160	1.3046780
H	-0.2027130	-2.3041050	-1.5720690
H	0.0729700	-3.9645620	-2.1116520
H	-1.3571870	-3.0828160	-2.6622000
H	-5.4393140	2.7133690	2.6906350
H	-5.5174200	1.9972840	1.0779520
H	-4.3730200	1.3608980	2.2704120
H	-5.1353330	3.6310770	-1.2008810
H	-3.7704350	3.9876180	-2.2692100
H	-4.9876360	2.8057720	-2.7535580
H	-1.2108660	3.3839700	-2.4164660
H	0.0740520	4.3520030	-1.6842270
H	-0.0971570	2.6451660	-1.2563370

III-Pc2

C	1.0303830	-0.2164560	-1.0993160
C	1.8742050	-1.4963900	-0.9291320
C	2.9090850	-1.3039800	-2.0592730
C	3.0155610	0.2344130	-2.3167300
C	2.0451890	0.9240230	-1.3314410
C	4.4701490	0.6400550	-2.0187700
C	5.2200490	-0.6904880	-2.1768680
C	4.3009070	-1.7809120	-1.6074160
C	2.6952250	-1.8782300	0.3517170
C	4.1966750	-2.0670520	-0.0758630
C	2.9430750	1.5976640	-0.2376620
C	4.4451090	1.4264400	-0.6702630
C	5.0962460	-1.3913070	1.0089670
C	4.1445980	-0.6955370	2.0024780
C	2.6863690	-1.0945400	1.7019980
C	4.2554770	0.8393570	1.7423210
C	2.8596910	1.3177250	1.2944800
C	1.9352490	0.2311000	1.8453420
C	5.2694880	1.0291290	0.5966350

III-Pd2

C	-6.2216540	-0.6915980	0.8613500
C	-5.5297330	0.6709430	0.7084560
C	-4.4554470	0.6157050	1.8121160
C	-4.1949340	-0.8915650	2.1219100
C	-5.1203140	-1.7074560	1.1975560
C	-2.7197600	-1.1693880	1.7704230
C	-2.0888600	0.2234530	1.8345790
C	-3.1300910	1.1976640	1.2840060
C	-4.7992170	1.0935550	-0.6076830
C	-3.2999130	1.4128990	-0.2523490
C	-4.2100180	-2.3412260	0.0967060
C	-2.7150290	-1.9997700	0.4476100
C	-4.8139840	0.2556300	-1.9250350
C	-3.3439030	-0.0308030	-2.2787560
C	-2.3963810	0.7800980	-1.3671860
C	-3.0869440	-1.5406620	-1.9696650
C	-1.9931890	-1.5925100	-0.8820750
C	-1.2730180	-0.2535920	-1.1382710
C	-4.4074380	-2.1236730	-1.4370060

C	6.0866150	-0.2688590	0.6668740
H	0.9269260	0.2274610	1.4401390
P	-0.6150730	0.0098360	-0.2368640
N	-1.0996560	1.3967060	-1.0820200
P	-1.9763710	2.5549520	-0.4549210
C	-2.4403530	2.3650780	1.3085910
C	-3.2978520	3.4874440	1.8979230
C	-3.6450760	3.2180240	3.3611950
N	-1.4246340	-1.2816970	-1.0093370
P	-2.3322220	-2.2945420	-0.1942980
C	-3.3378490	-1.5384220	1.1406130
C	-4.2562320	-2.4663530	1.9370910
C	-5.0431260	-1.6963720	2.9968510
C	-1.4245990	-3.6729150	0.6028450
C	-0.4957460	-3.2069090	1.7275970
C	0.4121350	-4.3257270	2.2312020
C	-3.5056490	-3.1215280	-1.3182270
C	-4.4912840	-2.1611930	-1.9892770
C	-5.4252030	-2.8758260	-2.9629520
C	-1.1083020	4.1574750	-0.5715200
C	0.1323030	4.1824730	0.3292270
C	1.1069060	5.2989150	-0.0350190
C	-3.5504850	2.7590250	-1.3600020
C	-4.4562750	1.5354840	-1.1817260
C	-5.6133420	1.5004900	-2.1762160
H	1.5022110	1.7316310	-1.8288620
H	1.2217680	-2.3473200	-1.1487540
H	4.8365580	1.3314630	-2.7843160
H	4.5545170	-2.7288990	-2.0925810
H	0.6220810	-0.3521290	-2.1145880
H	6.2492960	-0.7029360	-1.8285480
H	5.2759390	-0.8783510	-3.2577020
H	6.7526170	-0.4594000	-0.1702520
H	6.7408430	-0.1664630	1.5432690
H	1.8320850	0.4199440	2.9232580
H	5.6483380	-2.1930910	1.5095210
H	2.3164700	-1.7866190	2.4665000
H	4.4148210	-0.9229010	3.0380960
H	4.5812690	1.3535020	2.6515730
H	5.9392490	1.8665780	0.8165490
H	2.6080550	2.2676060	1.7789170
H	2.7435920	0.4591810	-3.3526730
H	2.5893130	-1.8149610	-2.9726550
H	2.3234560	-2.8722370	0.6149730
H	4.4161750	-3.1344010	0.0167490
H	4.8117050	2.4235500	-0.9297750
H	2.7171700	2.6638820	-0.3326160
H	-0.8176390	4.2593650	-1.6238690
H	-1.7892170	4.9839160	-0.3403390
H	-3.2754600	2.8780450	-2.4146050
H	-4.0546150	3.6810300	-1.0500700
H	-2.9570410	1.4031840	1.3983640
H	-1.5061870	2.2551030	1.8704770
H	-0.8406430	-4.1441860	-0.1973660
H	-2.1356330	-4.4233850	0.9683110
H	-2.9011440	-3.6371860	-2.0735070
H	-4.0398540	-3.8955110	-0.7548070
H	-3.9217310	-0.7420110	0.6639080
H	-2.6256920	-1.0355900	1.8052370
H	-3.8477700	0.6304080	-1.2967970
H	-4.8526380	1.5212350	-0.1586530
H	-0.1785290	4.2910360	1.3749540
H	0.6448200	3.2160310	0.2524780
H	-2.7685860	4.4441110	1.8181330
H	-4.2232390	3.5938860	1.3201420
H	0.1171550	-2.3720520	1.3688700
H	-1.0923170	-2.8166140	2.5607710
H	-5.0882340	-1.6505960	-1.2235640
H	-3.9281940	-1.3834840	-2.5157510
H	-4.9549970	-2.9724720	1.2609620
H	-3.6644570	-3.2520210	2.4207000
H	-6.0171330	-3.6413880	-2.4507340
H	-6.1179500	-2.1692630	-3.4292160
H	-4.8580560	-3.3682620	-3.7594870
H	-4.3662610	-1.2029590	3.7020280
H	-5.6649790	-0.9229620	2.5339150
H	-5.6984730	-2.3618910	3.5656340
H	-0.1724040	-5.1580100	2.6368550
H	1.0327200	-4.7189500	1.4188040
H	1.0796160	-3.9649810	3.0196580
H	-6.2482730	2.3869880	-2.0781510
H	-5.2402570	1.4664110	-3.2049640
H	-6.2391790	0.6170880	-2.0166110
H	-4.2604330	4.0214180	3.7757450
H	-4.1999170	2.2793690	3.4625210
H	-2.7381310	3.1377110	3.9690190
H	1.4717190	5.1749860	-1.0601600
C	-5.4459660	-1.1422500	-2.0003700
H	-1.1016310	0.3003760	1.3879790
P	0.3727680	0.1716470	-0.3491740
N	0.7362690	1.4786690	-1.3704770
P	1.4993270	2.7795610	-0.8839110
C	3.2348240	2.8590720	-1.4606100
C	4.0848380	1.7232890	-0.8869380
C	5.4847050	1.6455680	-1.4935440
C	6.3138040	0.5183150	-0.8813650
N	1.2908560	-1.1263660	-0.9657480
P	1.9023320	-2.2550730	-0.0364690
C	2.2805670	-1.7895910	1.6963410
C	3.2874730	-0.6451060	1.8033790
C	3.3919310	-0.0704140	3.2147210
C	4.3560770	1.1111360	3.2930820
C	0.8461430	-3.7365080	0.1658240
C	0.5151640	-4.4307830	-1.1598120
C	-0.7154610	-5.3381540	-1.0725850
C	-0.5786610	-6.4643340	-0.0496960
C	3.4608460	-2.8426150	-0.7773300
C	4.1858530	-3.9681150	-0.0386760
C	5.4959980	-4.3560310	-0.7258250
C	6.2326350	-5.4770480	0.0031050
C	1.6024660	3.0318790	0.9280540
C	0.2569660	3.3184600	1.6009900
C	0.3396630	3.1503120	3.1182170
C	-0.9697700	3.4805150	3.8291210
C	0.6809060	4.2642240	-1.5581640
C	1.3032590	5.6158850	-1.2003490
C	0.4721170	6.7892250	-1.7219150
C	1.0876450	8.1434160	-1.3779280
H	-1.2831960	-2.3898480	-1.1106070
H	-1.9542120	1.6114140	-1.9220310
H	-4.5936250	-3.1085150	-1.8772350
H	-5.2760450	0.8807420	-2.6957810
H	-0.8929640	-0.3979080	-2.1633400
H	-6.4540450	-1.2332810	-1.6052010
H	-5.5304660	-1.3765150	-3.0702170
H	-6.9031520	-0.9735390	0.0635640
H	-6.8441730	-0.6131390	1.7629450
H	-1.9612100	0.4641510	2.9002020
H	-6.2632200	1.4525410	0.9304390
H	-2.9509520	2.1855290	1.7208870
H	-4.7857680	1.1351100	2.7167960
H	-4.3998730	-1.1010060	3.1760690
H	-5.5760780	-2.5352330	1.7500200
H	-2.2564260	-1.7959390	2.5397880
H	-2.7582570	-2.0590480	-2.8757420
H	-3.1402970	0.1748980	-3.3341180
H	-3.1685160	2.4902860	-0.3901700
H	-5.2653790	2.0436500	-0.8832140
H	-4.3252860	-3.4199850	0.2356380
H	-2.2434360	-2.9478150	0.7269140
H	-0.3550830	4.2115810	-1.2014430
H	0.6413210	4.1305670	-2.6453250
H	1.4041930	5.7086530	-0.1114420
H	2.3168200	5.6835300	-1.6134920
H	-0.5396800	6.7251090	-1.3025880
H	0.3626680	6.6970620	-2.8095590
H	2.0883020	8.2410580	-1.8120640
H	0.4753940	8.9662770	-1.7576460
H	1.1815700	8.2670930	-0.2937990
H	3.1998310	2.7971260	-2.5550530
H	3.6638250	3.8339350	-1.2019320
H	3.5639490	0.7690680	-1.0408250
H	4.1825510	1.8543800	0.1980440
H	5.4032400	1.5002310	-2.5778040
H	5.9983080	2.6038670	-1.3464420
H	5.8451040	-0.4552800	-1.0622370
H	7.3219570	0.4858780	-1.3041550
H	6.4061170	0.6445830	0.2029680
H	2.3263380	3.8278130	1.1375260
H	2.0176350	2.1028730	1.3338170
H	-0.0718720	4.3373720	1.3595800
H	-0.5083880	2.6384330	1.2080680
H	1.1451780	3.7849380	3.5092210
H	0.6272210	2.1138580	3.3390410
H	-1.7747640	2.8195920	3.4914630
H	-1.2778400	4.5119400	3.6272400
H	-0.8740600	3.3644620	4.9125510
H	4.1016840	-1.9569380	-0.8582550
H	3.2197540	-3.1439060	-1.8031240
H	3.5386130	-4.8519970	0.0265610
H	4.4014820	-3.6627800	0.9930650
H	6.1408580	-3.4706610	-0.7920260
H	5.2832570	-4.6614080	-1.7577720
H	5.6166730	-6.3809890	0.0562190

H	0.6303670	6.2821570	0.0350620
H	1.9735180	5.2945530	0.6329850

H	7.1655150	-5.7360830	-0.5055700
H	6.4797230	-5.1817630	1.0283480
H	1.3246760	-1.4893660	2.1414490
H	2.6243370	-2.6782430	2.2371530
H	2.9843600	0.1505530	1.1166380
H	4.2778740	-0.9803360	1.4678420
H	2.3935740	0.2486330	3.5418760
H	3.7083510	-0.8579750	3.9097110
H	4.0163230	1.9365770	2.6574840
H	4.4395840	1.4921980	4.3148280
H	5.3583810	0.8241710	2.9570570
H	1.3232270	-4.4259590	0.8707740
H	-0.0704810	-3.3732460	0.6482950
H	0.3437940	-3.6714240	-1.9316710
H	1.3820090	-5.0195800	-1.4867900
H	-0.9062740	-5.7659030	-2.0634150
H	-1.5925860	-4.7240540	-0.8272260
H	0.3069890	-7.0750730	-0.2571590
H	-0.4821340	-6.0722520	0.9676440
H	-1.4526590	-7.1215920	-0.0688310

III-Pe$_2$

C	5.3021730	1.7312890	-0.0049070
C	3.8823720	1.9261880	0.5664990
C	3.2008370	2.9312400	-0.3792690
C	3.7600070	2.5259770	-1.7523870
C	5.2388830	2.2030510	-1.4792980
P	1.3645030	2.8444550	-0.2693760
C	1.0443000	3.1936030	1.5088080
C	-0.4364360	3.0651900	1.9758570
C	-0.7370470	4.3562960	2.7593080
C	0.6375650	4.8503350	3.2212010
C	1.5290380	4.5739410	2.0058890
N	0.7574960	1.4995450	-0.8362300
P	0.3545910	0.1455780	0.1018150
N	1.3297230	-1.0978740	-0.5109320
P	2.1951640	-2.1942860	0.2158840
C	1.9940310	-2.4057030	2.0352780
C	0.5196760	-2.4356010	2.5392360
C	0.4592300	-1.4429360	3.7170220
C	1.9116320	-1.2892320	4.1762500
C	2.6851400	-1.2919740	2.8560990
C	-1.1854510	-0.2956410	-0.8766830
C	-1.8715490	-1.6724080	-0.7845950
C	-2.8247510	-1.5957940	-1.9964100
C	-3.1042710	-0.0794900	-2.2551530
C	-2.3109330	0.7106430	-1.1912460
C	-2.7339270	-2.1842460	0.4193570
C	-4.1606970	-2.5514350	-0.1320680
C	-4.1762080	-2.2524000	-1.6646990
C	-2.9334510	-1.4279280	1.7703270
C	-4.4492320	-1.2171050	1.9517100
C	-5.2227040	-2.0088120	0.8785740
C	-2.3639750	-0.0229850	1.9724110
C	-3.3728140	0.9504760	1.3628980
C	-4.7285900	0.2961200	1.6926930
C	-5.6634460	0.3795150	0.4698060
C	-4.7976970	0.9003640	-0.7229290
C	-3.3674500	1.2506870	-0.1683190
C	-4.6169510	0.1364760	-2.0730890
C	-6.3142230	-1.0115370	0.4632570
C	-5.1773340	-1.2758180	-2.2995880
H	-1.3393920	0.1072800	1.6356390
C	0.7482000	4.2531690	-1.2669160
C	0.8786900	3.9996220	-2.8015370
C	-0.5224850	4.2640390	-3.3885270
C	-1.2092430	5.1652750	-2.3601460
C	-0.7466720	4.5627120	-1.0325690
C	1.8956130	-3.8605630	-0.5157020
C	1.6293710	-3.8273890	-2.0479900
C	0.4042900	-4.7398820	-2.2873130
C	0.2802710	-5.5819430	-1.0133410
C	0.6818850	-4.5992870	0.0876330
C	3.9934770	-1.8801240	0.0223600
C	4.9578970	-2.9835710	0.5469120
C	5.7985210	-3.3982670	-0.6723770
C	5.8431450	-2.1313470	-1.5307720
C	4.4042770	-1.6138160	-1.4461250
H	-3.2806090	1.9126250	1.8772950
H	-2.5417460	-2.0772240	2.5604180
H	-6.4487570	1.1225690	0.6415500
H	-5.7100150	-2.8805690	1.3267590
H	-2.3552040	0.1640360	3.0556730
H	-6.8826290	-1.2686700	-0.4263120
H	-7.0441650	-1.0058120	1.2841310
H	-6.2215700	-1.4239740	-2.0380790
H	-5.1209890	-1.4504890	-3.3826360

III-Pf$_2$

C	0.8885630	-0.0357890	0.8814020
C	1.7813220	-1.2920460	0.8478320
C	2.7528150	-0.9767840	2.0060100
C	2.7947510	0.5795820	2.1494810
C	1.8501770	1.1565820	1.0713000
C	2.6741940	-1.7396240	-0.3607210
C	4.1588260	-1.8397140	0.1481730
C	4.1799060	-1.4342910	1.6562880
C	2.7038730	-1.0569200	-1.7646390
C	4.1608500	-0.6289330	-2.0286240
C	5.0861860	-1.2136140	-0.9428570
C	1.9147050	0.2264860	-2.0388990
C	2.7796150	1.3831050	-1.5370160
C	4.2093590	0.9233260	-1.8820380
C	5.1591970	1.2360900	-0.7087850
C	4.2609920	1.6987090	0.4840450
C	2.7763950	1.7814690	-0.0287640
C	4.2473750	1.0160340	1.8882390
C	6.0209710	-0.0332180	-0.6419010
C	5.0332300	-0.2703880	2.1816410
H	0.8920860	0.2226630	-1.6698440
P	-0.7358770	0.0640310	-0.0477410
N	-1.4933390	-1.2286990	0.7558920
P	-2.2639380	-2.4745670	0.1649490
C	-3.7336330	-2.0414930	-0.8890140
C	-4.8222680	-3.1120350	-1.0268920
N	-1.3000670	1.4546960	0.7498460
P	-2.2809600	2.5203250	0.1105680
C	-3.9815060	1.8469880	-0.1856280
C	-4.4932540	1.1229360	1.0638590
C	-1.7608080	3.2618570	-1.5110280
C	-1.9788330	2.3141050	-2.6974630
C	-2.4633850	3.9183020	1.3175960
C	-2.8518260	5.2626850	0.6893510
C	-0.2910510	3.6894190	-1.4291190
C	-1.2186340	4.0683770	2.1989840
C	-5.0095200	2.8492590	-0.7163730
C	-1.1624370	-3.6823210	-0.7164360
C	-1.8386260	-4.9750070	-1.1825800
C	-2.9605080	-3.4182350	1.5900330
C	-1.8487910	-3.9973190	2.4716380
C	-0.3466310	-3.0452390	-1.8462760
C	-3.8865050	-2.5151450	2.4128780
C	-3.3230280	-1.4915980	-2.2607620
H	2.5235920	2.2827970	-2.1063780
H	2.3940110	-1.8152750	-2.4910880
H	5.8112630	2.0781900	-0.9617270
H	5.6884140	-2.0292550	-1.3555960
H	1.8465320	0.3293420	-3.1311010
H	6.6519000	-0.1363450	0.2366460
H	6.7130720	0.0277450	-1.4927340
H	6.0782170	-0.2705930	1.8835200
H	5.0424050	-0.3744550	3.2751670
H	0.4377140	-0.1106650	1.8853730
H	4.4417520	-2.3327550	2.2243520
H	1.1471450	-2.1463930	1.1034470
H	2.4060090	-1.4294500	2.9401920
H	2.4653850	0.8709420	3.1517420
H	4.5529250	1.7755920	2.6150100
H	1.2549480	1.9759820	1.4815380
H	4.5620690	1.3794940	-2.8119940
H	4.4875800	-0.9236140	-3.0304910
H	2.3450580	-2.7626020	-0.5652980
H	4.4190800	-2.9019540	0.1485600
H	4.5821420	2.7250950	0.6827310

H	-0.6792320	-0.3683830	-1.8537370
H	-4.2672380	-3.2157370	-2.1767290
H	-1.0974390	-2.4235460	-0.9615980
H	-2.3665460	-2.0492450	-2.8813790
H	-2.7809530	0.1937580	-3.2644930
H	-5.0059770	0.7878860	-2.8622510
H	-1.8351240	1.5842420	-1.6448030
H	-5.1873100	0.7513640	2.5756780
H	-4.7727520	-1.4933450	2.9598620
H	-2.2583900	-3.1286180	0.7049550
H	-4.2528870	-3.6391530	-0.0652910
H	-5.2661850	1.8472870	-1.0056800
H	-3.2653440	2.3393180	-0.2319760
H	-1.0733470	3.3187980	-3.4624890
H	-0.4811980	4.7013640	-4.3896580
H	-0.8456850	6.1959060	-2.4537100
H	-2.2981820	5.1827180	-2.4606560
H	-0.9071550	5.2176710	-0.1721860
H	-1.2989020	3.6322500	-0.8582130
H	1.2054700	2.9812830	-3.0212820
H	1.6180400	4.6848460	-3.2258540
H	-1.4340120	4.1889810	3.5849900
H	-0.5305160	2.1887180	2.6219030
H	-1.1362380	2.9049670	1.1535850
H	5.8463290	3.1036190	-1.6126760
H	2.5975100	4.5794980	2.2388820
H	5.6252540	1.4516350	-2.1741540
H	0.9821880	4.2562230	4.0762390
H	3.2245750	1.6311700	-2.0924500
H	1.3536360	5.3504220	1.2516940
H	3.6318030	3.2998170	-2.5132520
H	5.6218230	0.6877880	0.0772130
H	3.8922850	2.2570930	1.6084920
H	6.0260960	2.3267240	0.5583120
H	3.3242930	0.9820090	0.5234180
H	-0.4967050	-4.1267080	-2.4052030
H	0.5050630	-5.3462500	-3.1914230
H	1.4266840	-2.8078690	-2.3849740
H	4.4453190	-3.8370480	1.0009640
H	5.6020070	-2.5603610	1.3238190
H	6.5329930	-1.4028390	-1.0856950
H	6.7866140	-3.7742290	-0.3942260
H	5.2852680	-4.1934450	-1.2273780
H	6.1690240	-2.3150450	-2.5582470
H	4.2998680	-0.5589690	-1.7102960
H	3.7754100	-2.1816300	-2.1398220
H	-0.7217840	-5.9957560	-0.8681480
H	-0.1441270	-3.9017960	0.2640490
H	0.9883180	-6.4191740	-1.0404790
H	-0.1911250	-2.1505150	1.7601900
H	0.2570590	-3.4479040	2.8592540
H	-0.2140400	-1.7774380	4.5112290
H	0.0940630	-0.4779150	3.3519830
H	2.0827980	-0.3840830	4.7660010
H	3.7571580	-1.4705240	2.9783290
H	2.5582960	-0.3203950	2.3635200
H	0.9180880	-5.0809470	1.0404170
H	2.2140090	-2.1494120	4.7863220
H	-1.1890430	5.1057000	2.0990230
H	0.6414540	5.9019690	3.5208780
H	2.5079120	-4.1920400	-2.5882330
H	2.4738700	-3.3611820	2.2800920
H	4.1590950	-0.9649580	0.5987590
H	2.7893030	-4.4636230	-0.3178910
H	3.5165120	3.9538360	-0.1330250
H	1.3430160	5.1320880	-0.9952500
H	1.6216910	2.4176120	2.0230400

H	2.5045870	2.8412970	-0.0279580
H	0.0216610	4.1353360	-2.3786550
H	-0.1147050	4.4253650	-0.6392930
H	0.3469290	2.8239130	-1.2286780
H	-2.3815000	4.1524610	-1.6619930
H	-3.0318230	2.0556750	-2.8395450
H	-1.4129630	1.3884290	-2.5587770
H	-1.6309750	2.7975240	-3.6162810
H	-3.2896700	3.5944030	1.9633770
H	-1.4259180	4.7784430	3.0060310
H	-0.9232770	3.1092140	2.6259120
H	-0.3695370	4.4528170	1.6248190
H	-2.0298020	5.6734200	0.0947230
H	-3.7335310	5.1978600	0.0479720
H	-3.0723680	5.9824120	1.4835160
H	-3.8035990	1.0855510	-0.9573250
H	-5.9250050	2.3220810	-1.0027780
H	-5.2812130	3.5781010	0.0537890
H	-4.6534070	3.3958110	-1.5945450
H	-3.7555310	0.4029260	1.4263620
H	-4.7197430	1.8272800	1.8718510
H	-4.1548320	-1.2081650	-0.3132490
H	0.4809560	-3.7097250	-2.1160010
H	-0.9501460	-2.8871020	-2.7433080
H	0.0779670	-2.0837250	-1.5491960
H	-2.4911020	-4.7914830	-2.0413450
H	-5.2356550	-3.4162710	-0.0623230
H	-4.4573070	-4.0067950	-1.5370330
H	-5.6467320	-2.7081230	-1.6235820
H	-2.4826730	-0.7945430	-2.1861250
H	-4.1687000	-0.9621890	-2.7124260
H	-3.5474560	-4.2449150	1.1742730
H	-1.2663940	-4.7651790	1.9552340
H	-1.1674640	-3.2053400	2.7971870
H	-2.2872440	-4.4591830	3.3616370
H	-4.3169980	-3.0832510	3.2431230
H	-4.7124780	-2.1091120	1.8214970
H	-3.3213940	-1.6739320	2.8233820
H	-1.0755100	-5.6916420	-1.5023550
H	-2.4317140	-5.4522070	-0.3974430
H	-0.4507780	-3.9318480	0.0812280
H	-3.0425530	-2.3015190	-2.9409350
H	-5.4205910	0.5890450	0.8288110

III-Pg$_2$

C	1.7640980	-0.2029130	2.0339080
C	2.5720260	-1.4299160	1.6088720
C	4.0207230	-1.0254770	1.9433330
C	4.0519930	0.5328180	1.9997700
C	2.6213920	1.0189280	1.6954110
C	2.5605030	-1.9178300	0.1271360
C	4.0523070	-1.9422590	-0.3720630
C	4.9628790	-1.4555100	0.8017380
C	1.6779210	-1.3169310	-1.0214600
C	2.6621250	-0.8521770	-2.1190540
C	4.0875780	-1.3449150	-1.8139060
C	0.7766470	-0.0685700	-0.9149690
C	1.7348710	1.1398760	-0.9372760
C	2.6980600	0.7097390	-2.0639880
C	4.1430320	1.1156000	-1.7249250
C	4.1291550	1.6112910	-0.2438850
C	2.6365540	1.6151960	0.2534090
C	5.0146730	1.0047340	0.8920160

III-Ph$_2$

C	0.3767270	4.1254510	2.0719230
N	1.5045340	3.8513950	1.1684640
C	2.2799200	5.0645100	0.8925060
C	1.8075390	6.0464060	1.9661270
C	0.3410370	5.6535700	2.1581290
P	1.5725670	2.5303280	0.1517120
N	0.7550630	1.3929860	0.8562620
P	0.1980410	0.0068360	0.0350630
N	0.9638720	-1.2757550	0.8475220
P	1.9049940	-2.3809230	0.2642530
N	2.4667110	-3.4135940	1.4617340
C	1.5652700	-4.4980130	1.8967900
C	2.0804290	-4.8557720	3.2894610
C	2.4990350	-3.4921830	3.8420590
C	3.1549130	-2.8251970	2.6302800
N	1.1071880	3.1330230	-1.3473100
C	1.3250810	2.3728920	-2.5942800
C	-0.0317910	2.3953530	-3.2978030

C	4.9405550	-0.1188480	-2.1714640
C	5.8895120	-0.2377590	0.6710280
P	-0.8554630	-0.0185010	0.0034410
N	-1.5054740	1.3312930	-0.7985940
P	-1.9750410	2.6437690	-0.0903520
N	-1.0823140	3.9545420	-0.6686960
C	-0.6739700	3.9432200	-2.0664920
H	0.7464710	-0.1683430	1.6540030
N	-1.5904460	-1.3175870	-0.8141840
P	-2.3602930	-2.4988150	-0.1374890
N	-3.0665100	-3.4015210	-1.3681110
C	-3.4458350	-2.7416940	-2.6092450
N	-1.5079230	-3.6855270	0.7148140
C	-0.8495040	-3.2388990	1.9418180
N	-3.4718350	-2.0119870	1.0357870
C	-4.1829410	-2.9596340	1.8850480
C	-0.6392070	-4.5652310	-0.0627190
C	-4.2381960	-0.7967460	0.7880360
C	-3.9320670	-4.5207650	-1.0229260
N	-1.8289090	2.8607400	1.5700020
C	-2.7553100	2.1739090	2.4626520
N	-3.6292790	2.8512160	-0.3271360
C	-4.2900630	4.0833070	0.0752570
C	-0.4886430	2.9662490	2.1393910
C	-4.3039040	2.1590090	-1.4140680
C	-1.2412120	5.3005970	-0.1369050
H	1.1508540	2.0087240	-1.2478500
H	1.0506400	-2.1311310	-1.3956890
H	4.4545760	1.9642650	-2.3421930
H	4.3614270	-2.1604640	-2.4907990
H	0.3431110	-0.0259860	-1.9279050
H	5.9814190	-0.1542990	-1.8616700
H	4.9647030	-0.0793830	-3.2689520
H	6.5333510	-0.2186110	-0.2039090
H	6.5695280	-0.2841510	1.5324310
H	1.6808970	-0.2394800	3.1293390
H	5.5685470	-2.3131220	1.1112600
H	2.2680070	-2.2807010	2.2277600
H	4.3401600	-1.4445970	2.9021870
H	4.3856280	0.8680250	2.9864180
H	5.6548410	1.8115430	1.2627490
H	2.3480000	1.8343460	2.3728390
H	2.3816560	1.1253550	-3.0257850
H	2.3281180	-1.1840460	-3.1068780
H	2.2368240	-2.9615220	0.1894860
H	4.3208400	-2.9939120	-0.5054600
H	4.4427270	2.6572560	-0.3038400
H	2.3562450	2.6639920	0.3908580
H	0.2979000	-4.0672500	-0.3532610
H	-1.1485610	-4.9002980	-0.9656960
H	-0.3859580	-5.4397710	0.5448220
H	-0.5966080	-4.1128250	2.5503770
H	0.0759940	-2.6845940	1.7288830
H	-1.5119680	-2.5919420	2.5171190
H	-4.5301470	-0.3546700	1.7469010
H	-5.1549080	-0.9973050	0.2130610
H	-3.6342520	-0.0673340	0.2488430
H	-5.1594330	-3.2360920	1.4628550
H	-3.5928100	-3.8655150	2.0215420
H	-4.3558780	-2.5061950	2.8679900
H	-4.9746640	-4.2076380	-0.8661800
H	-3.9171820	-5.2531990	-1.8372680
H	-3.5737650	-5.0131130	-0.1176640
H	-3.4189900	-3.4707360	-3.4269150
H	-4.4622620	-2.3226550	-2.5605540
H	-2.7415720	-1.9384130	-2.8205990
H	-0.5193210	3.6064740	3.0282940
H	-0.1008700	1.9809430	2.4294390
H	0.1976900	3.4078820	1.4158520
H	-2.8297600	2.7337960	3.4013830
H	-3.7440080	2.1186190	2.0077340
H	-2.4149740	1.1536940	2.6892400
H	-4.4016360	2.7971600	-2.3046650
H	-5.3109050	1.8653190	-1.0940420
H	-3.7399420	1.2658780	-1.6811870
H	-4.3044020	4.8300250	-0.7317160
H	-5.3272640	3.8645870	0.3530840
H	-3.7931660	4.5156160	0.9450450
H	-1.9917290	5.8815860	-0.6917780
H	-1.5325480	5.2641250	0.9130280
H	-0.2843050	5.8302420	-0.2091260
H	-1.4002710	4.4643630	-2.7081460
H	-0.5738270	2.9127570	-2.4069200
H	0.2939960	4.4473450	-2.1701990
C	-0.6210760	3.7354570	-2.8536050
C	-0.1978240	3.8200790	-1.3867220
N	3.1703090	2.1589470	-0.1405750
C	3.9204950	1.4337400	0.9023180
C	5.3783620	1.5621540	0.4559420
C	5.4075830	2.9051000	-0.2763930
C	4.0806990	2.8913070	-1.0340390
C	-1.4419970	-0.0652080	0.9527910
C	-2.3921580	1.1496390	0.9671950
C	-3.3531300	0.7462460	2.1041120
C	-3.3296290	-0.8142790	2.1823970
C	-2.3574320	-1.3050460	1.0835940
C	-4.7960340	1.1598630	1.7654330
C	-4.7840460	1.6308150	0.2758020
C	-3.2941860	1.6100150	-0.2276450
C	-5.6024990	-0.0583390	2.2393320
C	-4.7632030	-1.2977640	1.8980520
C	-4.7478870	-1.9171280	0.4661820
C	-3.2603960	-1.9216850	-0.0418590
C	-3.2872780	0.9835020	-1.6571520
C	-4.7261440	0.5131260	-1.9500650
C	-5.6794370	1.0178000	-0.8480120
C	-3.2743480	-1.4654050	-1.5321400
C	-4.7180960	-1.0446210	-1.8663600
C	-5.6596690	-1.4395660	-0.7111090
C	-2.4489440	-0.2580690	-1.9748840
C	-6.5698240	-0.2077160	-0.5995720
N	1.1355330	-3.3461750	-0.8541560
C	1.8137590	-4.3055300	-1.7275400
C	0.6974760	-4.7872330	-2.6558980
C	-0.5328640	-4.7617410	-1.7453660
C	-0.3240150	-3.5005640	-0.9039980
N	3.3377450	-1.8941220	-0.4527620
C	3.2686270	-1.0182880	-1.6380450
C	4.4664900	-1.4396220	-2.4952880
C	5.4670520	-1.9796500	-1.4711420
C	4.5803160	-2.6830140	-0.4419250
H	-1.4324210	-0.2311440	-1.5909470
H	-1.7949120	2.0148280	1.2633600
H	-1.7355600	-2.1187010	1.4682490
H	-5.0958560	2.0225420	2.3689780
H	-5.0392190	-2.0993680	2.5906120
H	-1.0058440	-0.0140890	1.9641140
H	-6.6453170	-0.0908790	1.9352920
H	-5.6203960	0.0019550	3.3359760
H	-7.2071280	-0.1621610	0.2792250
H	-7.2558950	-0.2619840	-1.4556710
H	-2.3633260	-0.3111030	-3.0695650
H	-6.2782240	-2.2949500	-1.0007680
H	-2.9834260	-2.3312250	-2.1367920
H	-5.0502530	-1.4756310	-2.8156000
H	-5.0575910	0.8361650	-2.9415010
H	-6.3093540	1.8267360	-1.2315070
H	-3.0004950	1.7810520	-2.3509090
H	-3.0290380	1.1736740	3.0580580
H	-2.9904670	-1.1339340	3.1725550
H	-2.9538930	-2.9705570	-0.0803240
H	-5.0297320	-2.9628530	0.6177730
H	-5.0853040	2.6811480	0.3210790
H	-3.0099810	2.6542290	-0.3897980
H	-0.1072220	4.8516210	-1.0297660
H	-0.9333690	3.3047410	-0.7525440
H	-1.7061020	3.7940190	-2.9699440
H	-0.1722960	4.5551400	-3.4250330
H	-0.6497340	1.5736430	-2.9225810
H	0.0559160	2.2976470	-4.3824580
H	2.0896750	2.8720820	-3.2040630
H	1.6667470	1.3567500	-2.3838820
H	0.5552820	3.6766150	3.0570330
H	-0.5563830	3.7081670	1.6861520
H	-0.0838010	6.0082800	3.0998290
H	-0.2625890	6.0587020	1.3382210
H	1.9443430	7.0872220	1.6650300
H	2.3657100	5.8825940	2.8940200
H	4.1902990	2.3499440	-1.9844790
H	6.2673150	3.0195630	-0.9404980
H	2.0681080	5.4538880	-0.1150280
H	3.5881020	0.3933340	0.9654420
H	5.6229840	0.7577220	-0.2453720
H	3.7737340	1.9062000	1.8833470
H	6.0753830	1.5038110	1.2944820
H	5.0341470	-2.6872900	0.5523360
H	4.4052560	-3.7273970	-0.7302870
H	4.8616300	-0.6102810	-3.0866730
H	6.2045600	-2.6576690	-1.9063920
H	4.1751590	-2.2365220	-3.1880090
H	6.0095460	-1.1556220	-0.9975660

H	2.3192320	-1.1536390	-2.1691590
H	3.3320410	0.0338700	-1.3393130
H	2.6309290	-3.8274490	-2.2752240
H	2.2384270	-5.1445090	-1.1549840
H	0.9037910	-5.7719520	-3.0806320
H	0.5714200	-4.0771230	-3.4800410
H	-0.5344600	-5.6458200	-1.0985890
H	-1.4783660	-4.7406660	-2.2926920
H	-0.7538440	-3.5990090	0.0963590
H	-0.7845150	-2.6229940	-1.3716860
H	0.5214750	-4.1585090	1.9660480
H	3.0447410	-1.7366860	2.6471950
H	4.2262620	-3.0596350	2.5905710
H	1.6014870	-5.3379900	1.1967920
H	3.1714760	-3.5557370	4.7004080
H	2.9503870	-5.5168730	3.2122200
H	1.6108320	-2.9255980	4.1418340
H	1.3213570	-5.3570990	3.8938700
H	5.4205000	3.7288620	0.4464620
H	3.7008480	3.8890580	-1.2649180
H	3.3550720	4.8659350	0.9550970

I-Pa$_2$ (dication)

C	1.7076360	-1.6805060	-0.8531350
C	1.9740480	-1.9885330	0.6585910
C	1.5425140	-0.8531390	1.5839940
C	2.4166000	0.2963820	1.0765120
C	2.1468520	0.6282560	-0.4433070
C	1.0849490	-0.2966370	-1.0717340
C	3.4956890	-1.7337610	0.4324960
C	3.8292180	-0.3429740	1.0288860
C	3.4363740	0.1199210	-1.1427190
C	3.2263710	-1.4130630	-1.0681030
C	4.5371180	0.3829090	-0.1153220
P	-0.6025110	-0.1121890	-0.4757780
N	-1.1700040	1.2566750	-0.1140330
P	-1.1583680	2.8698500	0.0506970
C	-0.1015820	3.3363840	1.4307590
N	-1.3874420	-1.4377150	-0.4516580
P	-2.8908230	-1.9074490	0.0181940
C	-2.6708800	-3.2596630	1.1809960
C	-3.8437940	-0.5912090	0.7964310
C	-3.7700590	-2.4971060	-1.4342440
C	-0.5789330	3.6551130	-1.4620660
C	-2.8398560	3.4104240	0.3822690
H	5.4900890	-0.0776390	-0.3919400
H	4.6990520	1.4471270	0.0807620
H	1.6669160	-2.9835750	0.9792860
H	1.0012960	-0.0803600	-2.1456130
H	1.7981830	-1.0880170	2.6217080
H	3.5812270	0.5207700	-2.1469360
H	1.9652600	1.6906440	-0.6144590
H	3.7455070	-1.9926610	-1.8305960
H	4.1996270	-2.5301930	0.6720500
H	4.3356420	-0.3629670	1.9949430
H	2.3695700	1.1929700	1.6972080
H	1.2401950	-2.4777130	-1.4283610
H	-2.1315520	-2.8955410	2.0572010
H	-3.6471460	-3.6443360	1.4832130
H	-2.0942880	-4.0519570	0.6998980
H	-4.7488840	-2.8774490	-1.1325110
H	-3.8986560	-1.6733450	-2.1384470
H	-3.1939900	-3.2963560	-1.9036460
H	-4.8036660	-0.9961990	1.1243050
H	-3.2995760	-0.2001680	1.6584660
H	-4.0121680	0.2117810	0.0766680
H	-0.1802230	4.4129910	1.5971560
H	-0.4243190	2.8028010	2.3264740
H	0.9351990	3.0807260	1.2063100
H	-0.6030370	4.7398030	-1.3379770
H	0.4418120	3.3365240	-1.6806630
H	-1.2344950	3.3664400	-2.2857650
H	-2.8589910	4.4956240	0.5025640
H	-3.4782280	3.1248030	-0.4558850
H	-3.1979110	2.9324150	1.2959460
H	0.4616320	-0.6496340	1.5999560

I-Pa$_2$ (protonated)

C	-1.7793670	-1.7527450	-0.6586970
C	-2.7327560	-0.9572650	-1.6150670
C	-2.5104910	0.5538620	-1.5773580
C	-2.7836550	0.8502140	-0.1003250
C	-1.8175860	0.0608980	0.8648110
C	-0.8203080	-0.8614770	0.1393340
C	-3.9129030	-1.1865150	-0.6214520
C	-4.1468500	0.1460110	0.1361460
C	-2.7702040	-0.9822930	1.5139380
C	-2.9641150	-1.9684080	0.3332060
C	-4.1149600	-0.2626680	1.6085390
P	0.6690600	-0.1937190	-0.6774990
N	1.2252930	1.0471000	0.1761480
P	1.1832310	2.6358500	0.0799620
C	0.5529600	3.2853440	-1.4897560
N	1.7081050	-1.4078800	-0.8796390
P	2.9522270	-1.7853870	0.0555250
C	3.6232420	-3.3603910	-0.5122680
C	4.2984920	-0.5745390	-0.0032920
C	2.5431280	-1.9796470	1.8121160
C	0.1563070	3.3626440	1.3798880
C	2.8448210	3.3099630	0.3036080
H	-4.9373340	-0.9285010	1.8893300
H	-4.0993280	0.5912380	2.2935410
H	-2.8295090	-1.3803240	-2.6158310
H	-0.3544990	-1.4913780	0.9118820
H	-3.2581530	1.0578050	-2.1996890
H	-2.3775000	-1.4283650	2.4299470
H	-1.3370250	0.7150880	1.5948900
H	-3.2287600	-2.9886890	0.6129100
H	-4.8124780	-1.6865030	-0.9819320
H	-5.0087460	0.7246780	-0.2021510
H	-2.8047300	1.9172580	0.1365620
H	-1.3166320	-2.6408590	-1.0910600
H	3.9221550	-3.2665790	-1.5580640
H	4.4902650	-3.6388270	0.0906520
H	2.8552930	-4.1314830	-0.4251490
H	2.1571990	-1.0299910	2.1912620
H	1.7793310	-2.7520990	1.9259040
H	3.4312390	-2.2616970	2.3823220
H	4.6208410	-0.4442290	-1.0383700
H	3.9222640	0.3768280	0.3783040
H	5.1422740	-0.9093380	0.6044700
H	-0.4893030	2.9854450	-1.6219670
H	0.6082560	4.3763620	-1.4839880
H	1.1502400	2.9001210	-2.3190200
H	-0.8835910	3.0641300	1.2306290
H	0.4978070	2.9978050	2.3508880
H	0.2267490	4.4524420	1.3545860
H	2.8209980	4.4017770	0.2893550
H	3.2409960	2.9693980	1.2628380
H	3.4887270	2.9480690	-0.5007520
H	0.3196930	0.1977520	-1.9807600
H	-1.5328210	0.8790640	-1.9371530

I-Pb$_2$ (dication)

C	3.9256670	0.9920100	-1.1104430
C	3.8405410	-0.5335540	-1.3658700
C	4.8929010	-1.1154610	-0.4222110
C	4.3594640	-0.4114450	0.8256260
C	4.2832020	1.0774940	0.4035710
C	2.8526210	-0.7797600	0.8610610
C	2.4927420	-0.8742570	-0.6740620
C	2.4899460	1.5094120	-0.8007170

I-Pb$_2$ (protonated)

C	4.2203220	-2.8065110	0.0408280
C	4.4386740	-1.5460720	-0.7963150
C	3.0653630	-1.2819320	-1.4724750
C	2.0472990	-1.7977010	-0.3798060
C	2.9846560	-2.2705190	0.7632280
C	1.2707070	-0.6585290	0.3102360
C	2.4289950	0.1769670	0.8694140
C	3.4413560	0.6749290	-0.2109840

C	1.6127390	0.2989530	-1.1444310
C	2.8433910	1.5826470	0.7222630
C	2.2224340	0.4454660	1.5289540
P	-0.0631240	0.3600100	-0.4957920
N	-0.6046930	1.7943720	-0.3474210
P	-2.0230970	2.4658440	0.1550710
C	-2.8637000	1.4728850	1.4204810
C	-2.0025550	1.1253920	2.6362800
N	-0.8496320	-0.9111020	-0.1980850
P	-1.1988330	-2.4908510	-0.0727110
C	-1.0986880	-3.2787610	-1.7000390
C	-1.9753140	-2.6132080	-2.7630570
C	-0.0344450	-3.3070450	1.0488750
C	-0.0231800	-2.7154040	2.4598330
C	-2.8777680	-2.6125160	0.5927230
C	-3.3560390	-4.0459720	0.8366510
C	-1.5990420	4.0840190	0.8405020
C	-0.8489810	4.9888860	-0.1398240
C	-3.1162480	2.6552660	-1.2775550
C	-3.5117960	1.3320780	-1.9376220
H	5.9096330	-0.8094020	-0.6853520
H	4.8554260	-2.2068430	-0.3540120
H	2.1677830	2.4411880	-1.2623980
H	2.5244860	0.5028970	2.5787940
H	1.4627470	0.2340300	-2.2308880
H	4.8803850	-0.6002420	1.7652630
H	2.6514020	-1.7191710	1.3802450
H	5.1303260	1.6950150	0.7003550
H	4.5282210	1.5519480	-1.8246720
H	3.8864950	-0.8321300	-2.4140810
H	2.1058410	-1.8537130	-0.9597930
H	2.7359180	2.5715700	1.1667200
H	-2.5903520	3.3013690	-1.9865020
H	-3.9958700	3.2031370	-0.9249790
H	-0.9915120	3.8878170	1.7293140
H	-2.5342620	4.5406110	1.1798010
H	-3.2213090	0.5655390	0.9260560
H	-3.7478320	2.0490220	1.7132960
H	-2.8806070	-2.0307990	1.5202160
H	-3.5289040	-2.0895230	-0.1146650
H	-0.0432150	-3.2603400	-1.9894850
H	-1.3717710	-4.3294960	-1.5556260
H	0.9518500	-3.2399820	0.5791710
H	-0.3035850	-4.3681300	1.0632770
H	-1.1449970	0.5077260	2.3581450
H	-1.6358180	2.0160820	3.1504390
H	-2.6019000	0.5512600	3.3450550
H	-0.5655820	5.9106430	0.3712460
H	0.0609900	4.5059430	-0.5033260
H	-1.4692950	5.2592630	-0.9973710
H	-4.0783410	0.6886080	-1.2605140
H	-4.1414480	1.5402640	-2.8041920
H	-2.6360760	0.7821810	-2.2911410
H	-2.7357710	-4.5609290	1.5745000
H	-4.3764920	-4.0193650	1.2223470
H	-3.3640440	-4.6357770	-0.0831620
H	0.2211730	-1.6498850	2.4489630
H	-0.9863380	-2.8417910	2.9597210
H	0.7344060	-3.2246940	3.0576670
H	-3.0347870	-2.6486780	-2.4984340
H	-1.6901570	-1.5702260	-2.9184490
H	-1.8496640	-3.1397780	-3.7104670
H	1.1215880	0.4541040	1.5530030

C	3.0586250	0.2443340	-1.6233030
C	3.4539240	-0.9056530	1.3298970
C	4.4634720	-0.4067770	0.2545490
P	-0.0712840	0.1571130	-0.6347990
N	0.0867890	1.7515740	-0.6346030
P	-0.6959010	2.8746890	0.1936820
C	-0.1891430	4.4868650	-0.4732710
C	1.3264790	4.6976540	-0.4642580
N	-1.5021560	-0.3392230	-0.0985200
P	-2.3029440	-1.7174430	-0.0900380
C	-1.6154300	-2.9620110	1.0513200
C	-1.5893400	-2.4942590	2.5079310
C	-3.9922670	-1.3299390	0.4513840
C	-4.9360060	-2.5284500	0.5535440
C	-2.3900300	-2.5339840	-1.7164420
C	-2.9854210	-1.6395140	-2.8053930
C	-2.5137680	2.7788840	0.0773310
C	-3.0328450	2.6589000	-1.3554580
C	-0.3066710	2.9165900	1.9771460
C	-0.6896110	1.6527420	2.7494690
H	5.0517800	-3.0217820	0.7196510
H	4.0146530	-3.6966520	-0.5625350
H	3.7322830	1.7217680	-0.1133240
H	0.7379400	-1.0922360	1.1671730
H	3.8385090	0.5427510	-2.3329540
H	2.5009720	-2.9315160	1.4857130
H	1.4096890	-2.5968480	-0.7682010
H	3.7655600	-0.9144360	2.3748920
H	5.4544670	-0.0902540	0.5818210
H	5.2801210	-1.5507120	-1.4921350
H	2.9383850	-1.8180180	-2.4155030
H	2.1286820	0.9250640	1.6044000
H	-2.9173020	3.6649460	0.5794590
H	-2.8130470	1.8994670	0.6542550
H	-0.5750620	4.5335470	-1.4964150
H	-0.7038660	5.2620140	0.1038780
H	0.7698660	3.0997340	2.0562700
H	-0.8129880	3.7943030	2.3935570
H	-4.3706560	-0.5825530	-0.2533100
H	-3.8886140	-0.8210980	1.4152060
H	-1.3680700	-2.8334380	-1.9731320
H	-2.9672990	-3.4570640	-1.6009520
H	-2.1955890	-3.8841890	0.9424610
H	-0.6037300	-3.1838130	0.6934380
H	0.1215880	-0.2657110	-1.9664460
H	1.8326590	3.8942620	-1.0048590
H	1.5717660	5.6459670	-0.9474950
H	1.7237850	4.7302480	0.5537800
H	-2.7561890	3.5223140	-1.9666340
H	-2.6411740	1.7567070	-1.8315940
H	-4.1232850	2.5909840	-1.3481630
H	-0.1684630	0.7738910	2.3622580
H	-0.4136200	1.7660330	3.8000860
H	-1.7627540	1.4529310	2.7017250
H	-2.5999020	-2.3869760	2.9113870
H	-1.0582020	-3.2217670	3.1250380
H	-1.0834860	-1.5297420	2.6089370
H	-2.9636100	-2.1576460	-3.7663440
H	-4.0254920	-1.3813970	-2.5880410
H	-2.4193560	-0.7099920	-2.9087550
H	-5.0668740	-3.0245030	-0.4120180
H	-4.5745940	-3.2693590	1.2719530
H	-5.9197140	-2.1941090	0.8896600
H	2.1236970	0.6809430	-1.9804170

I-Pc2 (dication)

C	-3.1952200	-3.3976010	0.1285660
C	-4.3049680	-2.3393390	0.3527840
C	-5.0106680	-2.2667920	-1.0015580
C	-3.7337340	-2.0250610	-1.8071630
C	-2.7996610	-3.1771170	-1.3617830
C	-3.0706420	-0.8163810	-1.0971450
C	-3.4575600	-1.0410980	0.4185070
C	-1.8468030	-2.7669390	0.5875940
C	-2.2617950	-1.4692380	1.2749550
C	-1.4438190	-2.5536170	-0.9102000
C	-1.5792230	-1.0919210	-1.3612090
P	-0.4450780	0.0769490	-0.6002660
N	-1.0513960	1.4796750	-0.3934840
P	-0.4845910	2.8613460	0.3112460
C	1.2516930	3.1619690	-0.1096670
C	1.5295230	3.2459650	-1.6158540
C	3.0236950	3.3813400	-1.8978370
N	0.9892630	-0.3117030	-0.2564070
P	2.3115660	-1.2431020	-0.1155030
C	1.9609220	-2.6402440	0.9824070
C	1.4157030	-2.2284430	2.3566070

I-Pc2 (protonated)

C	-3.2276420	-3.6618470	-0.9221370
C	-4.3208440	-2.6306580	-0.5407300
C	-4.3870660	-2.7199720	0.9842320
C	-2.8873660	-2.5094670	1.1916460
C	-2.2381820	-3.5804950	0.2780070
C	-2.5670070	-1.2168330	0.3921360
C	-3.5686400	-1.3020000	-0.8211100
C	-2.1941490	-2.9295460	-1.8325670
C	-2.8586200	-1.5898000	-2.1464410
C	-1.1966200	-2.8561770	-0.6275560
C	-1.0977010	-1.4604100	0.0029720
P	-0.1586400	-0.1732190	-0.9047960
N	-1.0138070	1.1668700	-1.0729600
P	-0.9740360	2.5886930	-0.3452370
C	-0.0685740	2.6400250	1.2379020
C	-0.6380720	1.7099270	2.3128820
C	0.2291420	1.7070900	3.5692090
N	1.2641280	0.0545000	-0.1913270
P	2.5558250	-0.8450830	0.0655010
C	3.2357640	-1.5793630	-1.4538590
C	3.5675500	-0.5355310	-2.5266690

C	1.1486840	-3.4461230	3.2374020	C	4.0130360	-1.1874840	-3.8331460
C	2.8192820	-1.8441120	-1.7444610	C	3.8107300	0.2374710	0.8033310
C	3.1963430	-0.7230440	-2.7226900	C	5.1453540	-0.4345870	1.1381520
C	3.5401310	-1.2856500	-4.0996560	C	6.1252720	0.5544480	1.7660590
C	3.6203110	-0.2117430	0.5898460	C	2.2603170	-2.2272950	1.2135980
C	4.9884010	-0.8977880	0.6951070	C	1.7589110	-1.7679970	2.5887370
C	6.0287500	0.0490020	1.2897060	C	1.1537140	-2.9194600	3.3870770
C	-1.5287750	4.2024370	-0.2966140	C	-0.2809950	3.8861140	-1.4314210
C	-3.0122770	4.0352660	0.0605020	C	0.9238520	3.4597590	-2.2802410
C	-3.8587220	5.1325740	-0.5804630	C	2.2004400	3.1876650	-1.4867240
C	-0.6639700	2.6974770	2.1070170	C	-2.6887940	3.0853400	-0.0013830
C	0.3029620	1.6914940	2.7476620	C	-2.8617890	4.4474780	0.6780550
C	-0.0592650	1.4158550	4.2041500	C	-4.3365040	4.7817210	0.8937790
H	-5.5122500	-3.1999870	-1.2743020	H	-4.7364560	-3.6926760	1.3453080
H	-5.7270470	-1.4429890	-1.0729870	H	-4.9987090	-1.9326100	1.4365880
H	-0.5352770	-3.0537380	-1.2454990	H	-1.8184930	-3.4713430	-2.7016480
H	-2.5800050	-1.6626820	2.3037410	H	-0.5350480	-1.5553900	0.9405820
H	-1.3622120	-1.0089480	-2.4346610	H	-3.6002010	-1.7203240	-2.9426130
H	-4.9295940	-2.5046620	1.2317130	H	-2.5213990	-2.4961160	2.2206530
H	-3.9976680	-0.1819970	0.8189460	H	-2.7107990	-0.3038370	0.9739800
H	-3.4337030	-4.4091270	0.4551930	H	-1.9407980	-4.5044470	0.7753090
H	-2.7648630	-4.0385950	-2.0274220	H	-3.5917170	-4.6438150	-1.2262070
H	-3.8374800	-1.9030810	-2.8861870	H	-5.2610970	-2.7324460	-1.0864610
H	-3.4009000	0.1529460	-1.4703100	H	-4.2237240	-0.4287930	-0.8548670
H	-1.1674410	-3.3975270	1.1618120	H	-0.2398840	-3.3582880	-0.7888030
H	1.8200280	2.3410400	0.3398940	H	3.1774550	-2.8193400	1.3032720
H	1.5504270	4.0818530	0.4049810	H	1.5185810	-2.8728540	0.7279520
H	-0.5380660	3.6963400	2.5385030	H	3.9549700	1.0675010	0.1029710
H	-1.7034540	2.3954040	2.2770230	H	3.3513250	0.6661920	1.7012930
H	-1.3948600	4.2319220	-1.3835120	H	4.1199360	-2.1704820	-1.1900810
H	-1.1239890	5.1365780	0.1089510	H	2.4829810	-2.2867360	-1.8234190
H	1.2518820	-3.2888420	0.4564280	H	-3.1272920	2.2870880	0.6084570
H	2.8929720	-3.2074060	1.0847710	H	-3.2086430	3.0615680	-0.9660100
H	3.6591250	-2.5302720	-1.5884820	H	0.9662440	2.3646220	1.0174220
H	1.9867750	-2.4398990	-2.1328740	H	-0.0688640	3.6803460	1.5823540
H	3.2695540	0.1228650	1.5719150	H	-1.1039850	4.1895300	-2.0880940
H	3.6820880	0.6799260	-0.0430420	H	-0.0333340	4.7529890	-0.8080890
H	-3.3593640	3.0526910	-0.2760030	H	0.0246400	-0.7033630	-2.1993210
H	-3.1359410	4.0659640	1.1478420	H	4.9802040	-1.2707260	1.8268270
H	0.2871000	0.7472180	2.1934490	H	5.5867590	-0.8576340	0.2288690
H	1.3266380	2.0729990	2.6854670	H	2.5878020	-1.3193380	3.1473510
H	1.1427440	2.3505290	-2.1147440	H	1.0092200	-0.9778620	2.4638000
H	0.9966720	4.1004950	-2.0441020	H	2.6892410	0.0948150	-2.7055120
H	2.1267910	-1.5646290	2.8591560	H	4.3581540	0.1288260	-2.1594660
H	0.4895320	-1.6584980	2.2288210	H	0.6523380	2.5706320	-2.8601270
H	4.9111360	-1.7950380	1.3179110	H	1.1065690	4.2600770	-3.0045520
H	5.3177100	-1.2246240	-0.2969420	H	-1.6598110	2.0115490	2.5701060
H	2.3662040	-0.0141450	-2.8111940	H	-0.7017690	0.6896690	1.9192350
H	4.0538680	-0.1643940	-2.3322420	H	-2.3442530	4.4491500	1.6439560
H	2.0693320	-4.0092400	3.4150130	H	-2.3953160	5.2302750	0.0697160
H	0.7466860	-3.1357720	4.2046110	H	4.2513920	-0.4298610	-4.5842830
H	0.4254330	-4.1225850	2.7714400	H	4.9038040	-1.8047180	-3.6802010
H	5.7375030	0.3696940	2.2942710	H	3.2241150	-1.8291900	-4.2379700
H	7.0008130	-0.4447670	1.3592770	H	1.8812370	-3.7238420	3.5325220
H	6.1447260	0.9418150	0.6679170	H	0.8215050	-2.5790900	4.3712820
H	4.3842080	-1.9790330	-4.0397970	H	0.2877550	-3.3397530	2.8639440
H	2.6884030	-1.8232580	-4.5268280	H	5.7201540	0.9692790	2.6942730
H	3.8112100	-0.4792330	-4.7851890	H	7.0750710	0.0663680	1.9991890
H	-3.5374090	6.1232020	-0.2449200	H	6.3297050	1.3869560	1.0856750
H	-3.7791470	5.1017500	-1.6712920	H	2.4501260	4.0318540	-0.8352050
H	-4.9112910	5.0095060	-0.3138600	H	2.0974890	2.2889210	-0.8719890
H	-0.0337120	2.3350800	4.7965370	H	3.0431960	3.0243300	-2.1648320
H	-1.0633540	0.9880940	4.2817620	H	-4.8724840	4.8182770	-0.0597350
H	0.6467660	0.7070190	4.6439970	H	-4.8195480	4.0274740	1.5228430
H	3.2067970	3.4447740	-2.9730140	H	-4.4486970	5.7528660	1.3829350
H	3.4327040	4.2804910	-1.4281800	H	1.2480400	1.3804210	3.3367920
H	3.5787390	2.5193600	-1.5143460	H	0.2895520	2.7078670	4.0077410
H	-1.4648470	-0.7150840	1.3686230	H	-0.1783860	1.0287830	4.3236910
				H	-2.1814250	-0.7975060	-2.4721510

**I-Pd$_2$ (dication)**				**I-Pd$_2$ (protonated)**			
C	-3.8463620	-0.1469340	-0.3418780	C	-2.4433700	0.4292780	-0.2984860
C	-3.1318690	-0.2172790	-1.7329700	C	-3.4911290	-0.4248810	0.4332380
C	-2.0173650	0.8274430	-1.8848440	C	-4.6020790	0.6264960	0.7031880
C	-2.7856650	2.1441010	-1.6696820	C	-4.6518480	1.3483920	-0.6681000
C	-3.5025840	2.1926070	-0.2622640	C	-3.3046330	1.0623670	-1.3978670
C	-3.2570300	0.9282260	0.5667890	C	-5.3692810	0.3111160	-1.5822900
C	-4.3537330	0.4906260	-2.3940080	C	-5.6453670	-0.8702050	-0.6170800
C	-3.9695050	1.9680970	-2.6557190	C	-5.8948190	-0.1883890	0.7288940
C	-4.9988530	2.0561090	-0.6500510	C	-4.2205560	-1.4721500	-0.4897840
C	-5.0676120	0.5507020	-1.0107850	C	-3.7062090	-1.4337960	-1.9308720
C	-5.1074510	2.7555620	-2.0053940	C	-4.0227940	0.0125160	-2.3116410
P	-0.5742490	0.6005010	-0.8371590	P	-0.8274430	-0.2660030	-0.7968370
N	0.0928000	1.9270000	-0.4195740	N	-0.5837490	-1.7350940	-0.1929390
P	1.3174280	2.3306240	0.6075030	P	0.1842410	-2.2145400	1.1281030
C	1.9112580	3.9478220	0.0677250	C	-0.6403980	-1.7003320	2.6727670
C	3.1545160	4.4456970	0.8148070	C	-0.6278050	-0.1818190	2.8881700

C	3.5768510	5.8333560	0.3289340	C	-1.6989160	0.2960540	3.8678340
C	4.8193860	6.3446660	1.0531690	C	-1.7522360	1.8197650	3.9504090
N	-0.0784950	-0.7945790	-0.4861860	N	0.2739160	0.8358830	-0.4082350
P	0.0864710	-2.3997710	-0.3402630	P	1.2575410	1.8212770	-1.1789280
C	-1.4520140	-3.1346250	0.2678310	C	2.0369340	1.0647790	-2.6417570
C	-1.9134300	-2.5801350	1.6199290	C	2.7191560	-0.2681950	-2.3198580
C	-3.2429510	-3.1961080	2.0577080	C	3.3439580	-0.9227700	-3.5505210
C	-3.7741250	-2.5711710	3.3443300	C	3.9996320	-2.2589810	-3.2126300
C	0.4986970	-3.1200530	-1.9462280	C	0.4737470	3.3603200	-1.7662710
C	1.8038680	-2.5996100	-2.5599300	C	0.0593240	4.3545970	-0.6716170
C	2.0828950	-3.2720260	-3.9055310	C	-1.0162390	3.8510260	0.2936040
C	3.3611000	-2.7596640	-4.5625390	C	-1.4462080	4.9253770	1.2885210
C	1.4192930	-2.7091930	0.8421720	C	2.5686570	2.2826500	-0.0108900
C	1.7336310	-4.1898310	1.0816290	C	3.6453020	3.2231020	-0.5568060
C	2.8910170	-4.3560170	2.0680660	C	4.6731340	3.5874490	0.5155280
C	3.2218550	-5.8228350	2.3293460	C	5.7674730	4.5102930	-0.0143120
C	0.6189870	2.4284650	2.2747010	C	0.2207280	-4.0316050	1.1687310
C	0.2524880	1.0602160	2.8646180	C	1.2353340	-4.7100370	0.2381080
C	-0.6347710	1.1911480	4.1017690	C	1.0175920	-4.4739830	-1.2573400
C	-0.9879130	-0.1696620	4.6943210	C	1.9411720	-5.3431510	-2.1072840
C	2.6674370	1.1246990	0.5857370	C	1.8966100	-1.6049270	1.2128970
C	3.3120560	0.9400330	-0.7914420	C	2.6922210	-2.0127210	2.4539630
C	4.4168180	-0.1159910	-0.7630710	C	4.1174190	-1.4587750	2.4085710
C	5.0773130	-0.2934620	-2.1264090	C	4.9371030	-1.8460660	3.6364760
H	-6.0706900	2.5906290	-2.4971510	H	-6.7941450	0.4359370	0.7367240
H	-4.9126080	3.8309700	-1.9547920	H	-5.9433590	-0.8916920	1.5666100
H	-2.8764750	-1.2113220	-2.0983820	H	-2.8324950	1.8950280	-1.9235180
H	-3.8045730	0.9792250	1.5128480	H	-4.2928680	-2.1195010	-2.5524750
H	-1.6092530	0.7908310	-2.9038560	H	-2.1413500	1.2313860	0.3859050
H	-5.6892870	2.3754070	0.1318940	H	-6.3990080	-1.5811250	-0.9619250
H	-3.2679860	3.1164370	0.2683860	H	-4.2078800	-2.4696300	-0.0451150
H	-6.0483200	0.0860710	-0.9151620	H	-6.2226490	0.6459070	-2.1730610
H	-4.8509290	-0.0188620	-3.2184940	H	-5.0241250	2.3732290	-0.6463350
H	-3.7227660	2.2066270	-3.6912930	H	-4.4000780	1.2730580	1.5604010
H	-2.1804590	3.0305070	-1.8582340	H	-3.1087940	-0.8859650	1.3450730
H	-4.0197470	-1.1118760	0.1346090	H	-3.9725740	0.2277670	-3.3799130
H	2.2992740	-2.1836450	0.4565740	H	0.4203740	-4.3185710	2.2066530
H	1.1346810	-2.2119740	1.7758970	H	-0.7991260	-4.3573680	0.9366930
H	0.8482330	-4.7036970	1.4735750	H	2.2529270	-4.4041020	0.5117810
H	1.9937440	-4.6784170	0.1353130	H	1.1829760	-5.7863390	0.4387750
H	3.7741620	-3.8394060	1.6735140	H	-0.0286330	-4.6847120	-1.5093390
H	2.6324590	-3.8590350	3.0105450	H	1.1755140	-3.4157770	-1.4966720
H	2.3597530	-6.3506900	2.7496290	H	1.8084740	-5.1398360	-3.1737190
H	3.5079760	-6.3317900	1.4032910	H	2.9925030	-5.1597530	-1.8596750
H	4.0510990	-5.9177930	3.0352980	H	1.7423300	-6.4067430	-1.9391940
H	0.5401820	-4.2053290	-1.7996660	H	-1.6668710	-2.0776740	2.5958680
H	-0.3518380	-2.9186150	-2.6061310	H	-0.1752580	-2.2224880	3.5154770
H	1.7417970	-1.5136620	-2.6979860	H	0.3625960	0.1288460	3.2421710
H	2.6442080	-2.7898050	-1.8809630	H	-0.7742570	0.3414110	1.9378580
H	2.1523080	-4.3556340	-3.7541580	H	-2.6761680	-0.0842600	3.5440710
H	1.2301780	-3.1037600	-4.5738550	H	-1.5114770	-0.1323850	4.8594500
H	3.5484660	-3.2744900	-5.5085390	H	-1.9929750	2.2505740	2.9715890
H	3.2915940	-1.6873990	-4.7735860	H	-2.5131500	2.1552190	4.6602310
H	4.2290090	-2.9193040	-3.9144790	H	-0.7889450	2.2332950	4.2668690
H	-2.2094870	-2.9643390	-0.5049540	H	2.3952280	-1.9493160	0.2992130
H	-1.2910690	-4.2173210	0.3211030	H	1.8203230	-0.5155110	1.1336390
H	-1.1546570	-2.7745270	2.3867970	H	2.1927240	-1.6446640	3.3584490
H	-2.0230760	-1.4907510	1.5546160	H	2.7314320	-3.1057430	2.5410250
H	-3.9820580	-3.0735530	1.2564370	H	4.6138070	-1.8217380	1.5001170
H	-3.1106730	-4.2758840	2.1906130	H	4.0726740	-0.3657180	2.3224350
H	-4.7271690	-3.0209370	3.6342920	H	5.9499840	-1.4379160	3.5806020
H	-3.0705310	-2.7118560	4.1711640	H	4.4722640	-1.4684540	4.5531400
H	-3.9342620	-1.4946940	3.2189840	H	5.0174140	-2.9343430	3.7265160
H	1.3392440	2.9485520	2.9144820	H	1.2491720	0.9240890	-3.3913430
H	-0.2648120	3.0702450	2.1853490	H	2.7493260	1.7822680	-3.0638170
H	-0.2740310	0.4469670	2.1221000	H	3.4963940	-0.1169850	-1.5597260
H	1.1667540	0.5150690	3.1235520	H	1.9864350	-0.9563320	-1.8801900
H	-0.1227360	1.8067420	4.8502560	H	2.5684760	-1.0713320	-4.3116330
H	-1.5518550	1.7260600	3.8273920	H	4.0843930	-0.2419790	-3.9874130
H	-0.0874930	-0.7151650	4.9946270	H	4.4359090	-2.7256210	-4.0999580
H	-1.6285790	-0.0642800	5.5736370	H	4.7966280	-2.1295230	-2.4727300
H	-1.5230710	-0.7833370	3.9632300	H	3.2661830	-2.9552620	-2.7934780
H	1.0699930	4.6385830	0.1896680	H	3.0165730	1.3453200	0.3366530
H	2.1044910	3.8643970	-1.0072720	H	2.0617220	2.7233480	0.8553930
H	2.9594820	4.4861810	1.8927530	H	4.1603610	2.7503640	-1.4017660
H	3.9857110	3.7460290	0.6676630	H	3.1870570	4.1419090	-0.9432470
H	3.7655700	5.7928320	-0.7503560	H	4.1579500	4.0666430	1.3569020
H	2.7461290	6.5335800	0.4756170	H	5.1216400	2.6669130	0.9090510
H	4.6431650	6.4180700	2.1312000	H	5.3427820	5.4474810	-0.3888750
H	5.6691880	5.6722390	0.8974130	H	6.4889560	4.7579480	0.7690800
H	5.1042920	7.3359800	0.6909320	H	6.3137030	4.0384900	-0.8377030
H	2.2510620	0.1811300	0.9523020	H	-0.3951010	3.0518060	-2.3595120
H	3.4012580	1.4575680	1.3284820	H	1.1769470	3.8352050	-2.4593770
H	2.5495530	0.6464570	-1.5229380	H	0.9434010	4.6686780	-0.1025470
H	3.7315390	1.8910440	-1.1398490	H	-0.3085430	5.2565470	-1.1738750
H	5.1674140	0.1659090	-0.0158710	H	-1.8854810	3.5121030	-0.2848110
H	3.9962390	-1.0733120	-0.4308470	H	-0.6485230	2.9730340	0.8368130

H	4.3402670	-0.5782170	-2.8836070	H	-1.8460640	5.8042300	0.7720270
H	5.5529680	0.6359070	-2.4551710	H	-2.2214130	4.5510670	1.9633460
H	5.8433410	-1.0726530	-2.0948510	H	-0.5993960	5.2536100	1.9004470
H	-2.2085780	0.7639490	0.8605020	H	-0.8932460	-0.4309340	-2.1944470
				H	-2.6595090	-1.7267210	-2.0394850

I-Pe$_2$ (dication)				I-Pe$_2$ (protonated)			
C	-4.7798880	0.7623150	3.1338400	C	-0.2219780	-5.1739400	-0.9641490
C	-4.7366150	0.2316700	1.6968830	C	-1.2818020	-4.4084780	-0.1567310
C	-3.2777010	0.5204120	1.2625600	C	-0.9375470	-2.9501690	-0.4832900
C	-2.4295930	0.5677310	2.5714100	C	-0.6789210	-2.9673030	-1.9979820
C	-3.4331690	0.3226220	3.7115470	C	0.0003590	-4.3368060	-2.2491810
P	-2.6459110	-0.5941210	-0.0231720	P	-2.0833440	-1.6622790	0.0939890
C	-2.7407830	-2.3406240	0.4608360	C	-3.7761750	-2.0878680	-0.4505900
C	-1.9562910	-2.7194140	1.7293760	C	-4.6820410	-0.8694860	-0.7416690
C	-1.8624200	-4.2423300	1.6092370	C	-6.1315980	-1.4068570	-0.6119180
C	-1.5776870	-4.4892430	0.1193450	C	-6.0081670	-2.8725320	-0.1435030
C	-2.1942730	-3.2874480	-0.6405950	C	-4.6212690	-2.9510850	0.5014000
N	-1.0633110	-0.2648100	-0.2522080	N	-1.7383950	-0.2153030	-0.4912370
P	0.0917860	0.6348440	-0.6931980	P	-0.3790300	0.5832640	-0.7485790
N	1.5830370	0.2759340	-0.5971990	N	0.9426020	-0.1625870	-0.2328330
P	2.5190730	-0.9392290	-0.0020130	P	2.5017160	0.0786900	-0.0143970
C	4.0989010	-0.8096640	-0.8903100	C	2.8406980	1.5824560	0.9727280
C	5.3364540	-1.4506040	-0.1879370	C	4.3466760	1.9105580	1.1821180
C	6.4508850	-0.3854100	-0.2820970	C	4.4845530	2.2154580	2.6836360
C	6.0082670	0.5521450	-1.4083560	C	3.4035190	1.3538420	3.3413460
C	4.4951570	0.6528190	-1.2092030	C	2.2111480	1.5035540	2.3916080
C	-0.1859310	2.2340650	-1.4708920	C	-0.5361960	2.2503820	-0.0144080
C	0.9809950	3.2279430	-1.3344330	C	-1.9119280	2.7619580	0.4546670
C	0.4029690	4.4748620	-2.0511790	C	-1.5299940	4.1352020	1.0714200
C	-1.0416130	4.4923950	-1.4926430	C	-0.5583420	4.6746390	-0.0088330
C	-1.3698950	3.0650030	-0.9554490	C	-0.0522290	3.4537810	-0.8341360
C	-0.8494120	4.9983750	-0.0321570	C	-1.5140950	5.0690260	-1.1733880
C	0.6836370	5.2009080	0.0662790	C	-2.9137030	4.7144140	-0.6081620
C	1.0771430	5.6494480	-1.3414030	C	-2.7818280	4.9932990	0.8896670
C	1.1651580	3.7278300	0.1526090	C	-2.8805360	3.1672200	-0.7192990
C	0.1505400	3.1028090	1.1163050	C	-2.2535260	2.9413480	-2.0961900
C	-1.1684420	3.5749760	0.5114830	C	-1.0215480	3.8419840	-2.0026400
C	-3.5925310	-0.2975640	-1.5522490	C	-2.0114800	-1.7383970	1.9205620
C	-2.8479180	-0.6711090	-2.8736330	C	-0.5604140	-1.7259330	2.4713020
C	-3.8582590	-1.5027900	-3.6860620	C	-0.6583770	-0.9129130	3.7655800
C	-5.2256400	-1.1095970	-3.1222390	C	-1.6442930	0.1952020	3.3931010
C	-4.9648120	-1.0132730	-1.6161380	C	-2.7378750	-0.5395040	2.6004490
C	2.7166410	-0.6815110	1.7838800	C	3.1223970	-1.3844820	0.8911890
C	1.3776030	-0.8060560	2.5651140	C	4.6553680	-1.5345200	1.0405160
C	1.4274500	0.2986260	3.6484130	C	4.8549000	-3.0462020	1.1843860
C	2.8703040	0.8211750	3.6396520	C	3.8806350	-3.6189180	0.1537730
C	3.2970500	0.6919030	2.1755270	C	2.6278920	-2.7359730	0.2830020
C	1.7406450	-2.5571920	-0.2790700	C	3.4264820	0.2707280	-1.5747370
C	1.5924990	-2.9087720	-1.7885100	C	3.1742930	-0.8580540	-2.5984180
C	2.5604590	-4.0760920	-2.0162360	C	3.4001010	-0.2092110	-3.9899590
C	2.5146440	-4.8390040	-0.6914790	C	3.6892150	1.2820730	-3.7247640
C	2.5379630	-3.7272320	0.3669110	C	3.0789890	1.5558610	-2.3468870
H	0.6462870	6.6158060	-1.6194130	H	-2.6132750	6.0503700	1.1195960
H	2.1592790	5.6829470	-1.4987660	H	-3.6372000	4.6352030	1.4717340
H	-2.3526800	2.6598430	-1.1977130	H	1.0067420	3.4424980	-1.1010430
H	0.2829330	3.5112200	2.1230670	H	-2.9264820	3.3156200	-2.8759010
H	-0.3417070	1.9834180	-2.5293750	H	0.0884390	2.1713010	0.8847400
H	1.0142940	5.8237720	0.8985450	H	-3.7530540	5.1797290	-1.1290180
H	2.2023200	3.6256670	0.4770570	H	-3.8609120	2.7001250	-0.6016290
H	-1.4593180	5.8339550	0.3091820	H	-1.4188980	6.0658690	-1.6052830
H	-1.7841170	4.9804170	-2.1226360	H	0.1728500	5.4053540	0.3385070
H	0.4792790	4.4358350	-3.1386570	H	-1.1113540	4.0672920	2.0781230
H	1.9115400	2.8593640	-1.7657030	H	-2.3962260	2.0879820	1.1619300
H	-2.0392500	3.4715490	1.1603620	H	-0.5272490	4.0445680	-2.9538340
H	-3.6855060	-2.5711330	-3.5167640	H	0.3127090	-0.5360180	4.0998990
H	-3.7676520	-1.3213620	-4.7590260	H	-1.0727320	-1.5285510	4.5723360
H	-5.5310810	-0.1315210	-3.5103910	H	-2.0435820	0.7353340	4.2549420
H	-6.0128830	-1.8282100	-3.3606610	H	-1.1414460	0.9264320	2.7471280
H	-5.7403970	-0.4722600	-1.0712660	H	-3.5085260	-0.9194930	3.2771100
H	-4.9085060	-2.0250700	-1.2018520	H	-3.2336700	0.1212920	1.8844750
H	-1.9147900	-1.2150940	-2.7109150	H	0.1119370	-1.2117680	1.7751420
H	-2.5856720	0.2509150	-3.3976990	H	-0.1746020	-2.7381310	2.6194060
H	-2.8215910	-4.6874890	1.8928910	H	-6.6756310	-1.3341800	-1.5567100
H	-2.4461380	-2.3905590	2.6479950	H	-4.4683100	-0.4462220	-1.7245580
H	-0.9529530	-2.2810540	1.7021250	H	-4.5009110	-0.0744970	-0.0131420
H	-3.1571980	0.8601520	4.6210010	H	1.0680410	-4.2113720	-2.4502110
H	-2.9873090	-3.5865260	-1.3289420	H	-4.2502550	-3.9734630	0.6113080
H	-3.4721980	-0.7442720	3.9581650	H	-0.4332960	-4.8258230	-3.1248330
H	-1.9864540	-5.4404590	-0.2259780	H	-6.0458060	-3.5507040	-1.0029620
H	-1.6096670	-0.1531550	2.5875950	H	-1.6224950	-2.9034460	-2.5512920
H	-1.4248270	-2.7864400	-1.2349890	H	-4.6525140	-2.4937650	1.4973090
H	-1.9761240	1.5582490	2.6607140	H	-0.0603760	-2.1228590	-2.3123840
H	-5.6353080	0.3763450	3.6923900	H	-0.5262820	-6.2012180	-1.1777270
H	-5.4613870	0.7103240	1.0358200	H	-1.2494820	-4.6322350	0.9131710
H	-4.8481340	1.8559750	3.1228670	H	0.7050160	-5.2230260	-0.3830430
H	-4.9479500	-0.8432490	1.7033660	H	-2.2855490	-4.6537810	-0.5239180

H	0.7396120	1.1091540	3.3878190	H	5.8913200	-3.3494070	1.0172500
H	1.1235950	-0.0787980	4.6265340	H	4.5686190	-3.3644470	2.1935890
H	0.5099670	-0.6630370	1.9145760	H	5.1783790	-1.1931420	0.1401260
H	1.7688860	-2.0634440	-2.4602190	H	2.1415720	-1.2108830	-2.5143290
H	0.5657040	-3.2388360	-1.9699870	H	3.8280680	-1.7151530	-2.4199090
H	1.5810010	-5.4084160	-0.6222720	H	4.7694090	1.4569870	-3.6785280
H	2.2755950	-4.6850400	-2.8767190	H	4.2207560	-0.6862770	-4.5304850
H	3.5757760	-3.7031220	-2.1963320	H	2.5021580	-0.3226030	-4.6032790
H	3.3417090	-5.5412220	-0.5676270	H	3.2825040	1.9364030	-4.4994100
H	2.1028640	-4.0249050	1.3227620	H	3.4703850	2.4602560	-1.8734600
H	3.5784330	-3.4467550	0.5612120	H	1.9908620	1.6622600	-2.4391440
H	2.9513010	1.8461690	4.0079020	H	4.3101360	-3.5186780	-0.8496110
H	2.8327260	1.4876310	1.5812950	H	2.1162050	-2.6109020	-0.6732880
H	3.5094100	0.1879980	4.2644080	H	3.6571090	-4.6770510	0.3100350
H	5.1556000	-1.7276940	0.8530870	H	4.9864070	1.0644610	0.9192630
H	5.6148720	-2.3667160	-0.7133440	H	4.6594460	2.7457250	0.5517550
H	7.4306630	-0.8346910	-0.4570300	H	4.2716350	3.2737690	2.8705310
H	6.5096160	0.1740680	0.6575330	H	5.4897970	2.0072410	3.0581750
H	6.4990430	1.5273200	-1.3700570	H	3.7280650	0.3072160	3.3833280
H	3.9562300	1.0337260	-2.0780450	H	1.6881470	2.4423990	2.6010980
H	4.2753280	1.3177840	-0.3675990	H	1.4715040	0.7029860	2.4924810
H	4.3783830	0.7465670	2.0297690	H	1.9082000	-3.1887450	0.9703780
H	6.2252040	0.1034250	-2.3842910	H	3.1656080	1.6645310	4.3614880
H	-1.0940990	-4.6626220	2.2617680	H	-6.6853000	-0.8157330	0.1226610
H	-0.4980780	-4.5239190	-0.0560100	H	-6.8061910	-3.1673580	0.5422930
H	1.2853990	-1.8055570	2.9952660	H	5.0495990	-0.9686730	1.8874570
H	3.9231420	-1.3234720	-1.8418780	H	2.3639380	2.3837410	0.3993910
H	0.7527300	-2.4625600	0.1844030	H	4.4924180	0.3069340	-1.3204560
H	3.3992550	-1.4662750	2.1262370	H	2.6824420	-1.2757920	1.8896080
H	-3.2440630	1.5034260	0.7790150	H	0.0027550	-2.6934520	0.0132680
H	-3.7712860	0.7834510	-1.5439370	H	-2.4937670	-2.6794130	2.2037090
H	-3.8008880	-2.5548450	0.6353580	H	-3.6401660	-2.6493160	-1.3847290
H	0.2340010	2.0124000	1.2419790	H	-0.2526200	0.7938800	-2.1379260
				H	-2.0528000	1.8938310	-2.3291190
**I-Pf$_2$ (dication)**				**I-Pf$_2$ (protonated)**			
C	1.2860250	-2.2417890	1.5575190	C	-5.4953810	-0.2342820	-0.1709350
C	2.6711650	-1.9072620	0.9964890	C	-4.1915630	0.0671680	-0.9089620
C	3.3747230	-3.2897230	0.9758820	C	-3.1302600	0.1707180	0.2184950
C	2.2297710	-4.1882650	0.4450630	C	-3.6429640	-0.8831800	1.2782120
C	0.8888970	-3.4321240	0.6885980	C	-4.9333030	-1.4261430	0.6041640
C	2.1697970	-3.8203070	-1.0678280	C	-2.7597240	-2.1359980	1.3557670
C	3.2885660	-2.7578520	-1.2144570	C	-2.8551420	-2.6444510	-0.0786000
C	4.3443080	-3.2044610	-0.2032290	C	-2.3320710	-1.6082580	-1.1242820
C	2.6206600	-1.5323230	-0.5362160	C	-1.8541090	-0.2838640	-0.5143950
C	1.1949230	-1.6133040	-1.1129920	C	-4.3216630	-2.3259860	-0.5003400
C	0.8226510	-3.0733400	-0.8331080	C	-3.8022610	-1.2927080	-1.5427040
P	0.0244660	-0.3758580	-0.5359510	P	-0.2661320	-0.2310100	0.3890190
N	-1.4160540	-0.8975320	-0.4529010	N	0.5763450	1.0815140	0.0249260
P	-2.9427590	-0.4497770	-0.0546940	P	0.5086060	2.6699760	0.1221560
C	-4.0835780	-1.3458940	-1.1818380	C	-0.7593610	3.3461680	-1.0333920
C	-5.4454930	-1.6118950	-0.5231990	C	-0.6075120	2.8155940	-2.4657410
N	0.5118550	1.0422150	-0.2927880	N	0.5030090	-1.6004910	0.1016000
P	1.2772380	2.4573130	-0.0911400	P	2.0454580	-1.9888680	-0.0564360
C	1.9796420	2.9818530	-1.7005090	C	2.8965620	-0.9460570	-1.3103900
C	0.8748690	3.1298420	-2.7551200	C	4.3474840	-1.3670770	-1.5628260
C	0.0142790	3.6845060	0.4200750	C	2.9944410	-1.8332840	1.5165300
C	-0.5483070	3.3952750	1.8172820	C	2.1958040	-2.4727100	2.6592450
C	2.6399560	2.1858180	1.1061600	C	2.1071780	-3.7431420	-0.6323540
C	2.1871590	1.5384840	2.4201790	C	0.8344030	-4.5106210	-0.2579270
C	-3.2071630	-0.9334610	1.6927850	C	3.3433650	-0.3720710	1.8280310
C	-2.4328930	-0.0184250	2.6486330	C	0.0854050	3.3020860	1.8006130
C	-3.1665060	1.3605050	-0.2389250	C	-1.3770220	3.0431850	2.1808390
C	-2.8501560	1.8334950	-1.6623130	C	2.2178120	3.2480650	-0.2395010
C	0.4665250	5.1428630	0.2759540	C	2.5226350	4.6939910	0.1651640
C	3.4687190	3.4530870	1.3486900	C	1.0340100	2.7021210	2.8462890
C	3.0879620	2.0396760	-2.1824870	C	2.6246730	2.9705450	-1.6920700
C	-2.8414070	-2.4077330	1.9092750	C	-0.8915540	4.8723550	-1.0100780
C	-4.5552100	1.8266930	0.2152570	C	3.3674810	-4.4948820	-0.1846450
C	-3.4562480	-2.6255910	-1.7482100	C	2.0911100	-0.8719380	-2.6109990
H	4.7929850	-4.1685740	-0.4599320	H	-6.3144080	-0.5066520	-0.8443430
H	5.1404640	-2.4682140	-0.0574330	H	-5.8244050	0.5861580	0.4751480
H	0.0423370	-4.0118210	1.0565810	H	-1.6631800	-2.0098040	-1.8855080
H	1.2266930	-1.4379270	-2.1973680	H	-3.2143120	-2.8575960	2.0438910
H	1.3602670	-2.5322680	2.6094790	H	-1.6813070	0.4115940	-1.3459420
H	3.6206680	-2.5705040	-2.2366240	H	-5.6105870	-1.9360740	1.2923000
H	3.1295300	-0.5920270	-0.7528860	H	-3.8259320	-0.4131460	2.2471770
H	2.1884740	-4.6230300	-1.8040320	H	-4.9587860	-3.1392120	-0.8498880
H	2.2914340	-5.2410210	0.7187700	H	-4.0835690	-1.4197130	-2.5887010
H	3.7880340	-3.5881220	1.9402910	H	-4.1895610	0.9170710	-1.5949830
H	3.2122450	-1.1542910	1.5726290	H	-3.0663270	1.1749530	0.6446410
H	-0.0594040	-3.4212730	-1.3682480	H	-2.4974870	-3.6642000	-0.2284590
H	-4.2807660	-0.8014880	1.8677530	H	-1.6825270	2.9142310	-0.6234240
H	-2.4144370	1.7785970	0.4399750	H	0.2438930	4.3857800	1.7598120
H	2.4155970	3.9677000	-1.5007630	H	2.1337050	-3.6539570	-1.7256380
H	3.2628270	1.4554850	0.5747440	H	3.9264200	-2.3921240	1.3791650
H	-4.2326420	-0.6461050	-2.0121100	H	2.8025410	2.5767320	0.4022910

H	2.7044030	1.0307470	-2.3595240	H	3.3616180	-4.6735400	0.8947340
H	3.4725180	2.4139550	-3.1339760	H	3.3943340	-5.4695930	-0.6791730
H	3.9303970	1.9844480	-1.4893460	H	4.2911230	-3.9699440	-0.4394570
H	0.4076080	2.1654200	-2.9730760	H	0.7519480	-4.6463730	0.8245700
H	0.0955700	3.8362640	-2.4596730	H	-0.0596920	-3.9875910	-0.6004920
H	1.3217830	3.5001100	-3.6804000	H	0.8671110	-5.5016110	-0.7198260
H	-0.7798680	3.4963960	-0.3137630	H	2.8803810	0.0503820	-0.8545120
H	1.3002860	5.3805990	0.9399760	H	2.0373640	-1.8425050	-3.1146880
H	0.7538870	5.3956690	-0.7467640	H	1.0735350	-0.5177800	-2.4321040
H	-0.3696870	5.7898340	0.5520270	H	2.5797730	-0.1735210	-3.2970720
H	0.1218440	3.7650530	2.5966430	H	4.3977930	-2.3301920	-2.0800080
H	-1.5034420	3.9145400	1.9278220	H	4.8313840	-0.6251070	-2.2040670
H	-0.7209780	2.3298090	1.9911110	H	4.9328060	-1.4396680	-0.6416280
H	2.9107750	4.1877080	1.9342890	H	4.0603140	0.0417940	1.1142570
H	4.3566260	3.1802820	1.9237950	H	2.4542230	0.2647990	1.8208290
H	3.8078100	3.9253600	0.4235190	H	3.7937810	-0.3130890	2.8227580
H	1.5403240	0.6738510	2.2595620	H	2.7714280	-2.4108370	3.5867240
H	3.0745160	1.1936750	2.9565940	H	1.2460510	-1.9508420	2.8101000
H	1.6647920	2.2479490	3.0634140	H	1.9742220	-3.5269540	2.4725010
H	-1.3545260	-0.0664520	2.4672760	H	-1.5596920	2.9261860	-2.9916200
H	-2.7549740	1.0237330	2.5890160	H	0.1454690	3.3792070	-3.0205960
H	-2.6040550	-0.3568470	3.6729510	H	-0.3245630	1.7603320	-2.4887630
H	-1.7826200	-2.5837290	1.7031910	H	-1.0249210	5.2728950	-0.0018590
H	-3.0334890	-2.6673740	2.9527390	H	-0.0174680	5.3512680	-1.4594830
H	-3.4293750	-3.0836650	1.2851540	H	-1.7642850	5.1650230	-1.6002290
H	-5.3703120	-2.3679660	0.2626170	H	2.3796260	4.8696170	1.2338570
H	-5.8969140	-0.7139130	-0.0954220	H	3.5698590	4.9095210	-0.0646510
H	-6.1288820	-1.9932310	-1.2851640	H	1.9114900	5.4124960	-0.3864710
H	-2.5307950	-2.4163560	-2.2865720	H	2.2918120	1.9868030	-2.0322000
H	-3.2389380	-3.3562020	-0.9649670	H	2.2170370	3.7279440	-2.3668160
H	-4.1673360	-3.0779420	-2.4439500	H	3.7143400	3.0080810	-1.7728930
H	-3.6011220	1.4906290	-2.3788470	H	-1.5620710	3.4443130	3.1809560
H	-2.8582640	2.9267450	-1.6788010	H	-2.0835120	3.5237370	1.4995840
H	-1.8691160	1.5012550	-2.0091080	H	-1.5987870	1.9721350	2.2154530
H	-5.3334530	1.4818020	-0.4708510	H	2.0869200	2.8918730	2.6212140
H	-4.8060810	1.4963050	1.2261930	H	0.8190770	3.1398930	3.8245720
H	-4.5738180	2.9189910	0.2107370	H	0.8919800	1.6200740	2.9248380
H	0.5639990	-1.4106280	1.5428820	H	-0.6151970	-0.1695250	1.7582840
				H	-1.7360830	-1.9744240	1.6998060
**I-Pg2 (dication)**				**I-Pg2 (protonated)**			
C	-0.7414270	-3.0263150	-0.9489360	C	4.5841380	-1.8982080	-0.2306170
C	-0.7188510	-3.5257060	0.5347740	C	4.9506310	-0.8774720	0.8772800
C	-1.0684840	-2.4301500	1.5388950	C	5.5247380	0.3076540	0.1010420
C	-2.4821440	-2.0514400	1.0887740	C	4.3253090	0.4424640	-0.8364180
C	-2.5184550	-1.5307900	-0.4000580	C	4.1446440	-0.9853550	-1.4126670
C	-1.1288850	-1.5495300	-1.0713430	C	3.1027830	0.5223480	0.1158470
C	-2.0706110	-4.2655190	0.2964260	C	3.5350300	-0.4106690	1.3160310
C	-3.1847320	-3.4296130	0.9750010	C	3.1014690	-2.3200060	0.0008880
C	-3.2248140	-2.6881200	-1.1544370	C	2.7549220	-1.7318520	1.3644520
C	-2.0998660	-3.7544720	-1.1750470	C	2.6581260	-1.4045230	-1.1856410
C	-4.2207340	-3.2366860	-0.1327930	C	1.9908880	-0.0928890	-0.7541280
P	0.0788300	-0.3693620	-0.4538650	P	0.3024490	-0.1132370	-0.0700070
N	1.5104020	-0.9216830	-0.3681890	N	-0.5406640	1.1252040	-0.6474820
P	3.0079560	-0.3505960	-0.0268380	P	-0.8162080	2.5772210	-0.0832590
N	3.7810610	0.1078510	-1.4043540	N	-2.4680090	2.8031400	-0.0876190
C	4.6590680	-0.8274710	-2.1091270	C	-3.0170660	4.1216090	0.2136870
N	-0.3345820	1.0535740	-0.0907290	N	-0.3066150	-1.5583810	-0.3955040
P	-1.3853040	2.2835470	0.0158340	P	-1.7208780	-2.1835830	-0.0446720
N	-0.6562420	3.5625910	-0.6975440	N	-2.9022040	-1.5578170	-1.0473270
C	0.7545440	3.8507610	-0.4491360	C	-4.3215030	-1.8383090	-0.8467650
N	-2.7886550	1.9846570	-0.7782990	N	-1.5234210	-3.8280140	-0.1525700
C	-2.7711570	1.6743000	-2.2070930	C	-2.6973820	-4.6905130	-0.0869770
N	-1.6368210	2.5352730	1.6158460	N	-2.3666130	-1.9437940	1.4707490
C	-1.8530010	1.4450040	2.5633220	C	-2.8703020	-0.6238950	1.8468150
C	-4.1156580	2.1181580	-0.1844380	C	-0.3640120	-4.4346480	-0.7941360
N	2.9584250	0.9815240	0.9315710	N	-0.3781430	2.9683110	1.4704500
C	3.9250070	2.0784870	0.8664190	C	1.0114470	3.2417510	1.8228670
N	3.7459120	-1.6057400	0.7183490	N	0.0541330	3.6679110	-0.9966870
C	3.4487690	-3.0084200	0.4269290	C	0.4041500	3.3647230	-2.3799620
C	2.1551570	0.9554420	2.1560130	C	-1.2129700	2.6492450	2.6230500
C	4.9551860	-1.3861590	1.5107650	C	0.0318080	5.0984290	-0.7087390
C	3.1261280	1.0889380	-2.2725720	C	-3.3259030	1.9949280	-0.9471380
C	-1.9450490	3.8688270	2.1320760	C	-1.8277440	-2.6860090	2.6081470
C	-1.3891170	4.6236800	-1.3862050	C	-2.5610630	-1.3332690	-2.4506210
H	-4.6835800	-4.1737240	-0.4560560	H	6.4540540	0.0675500	-0.4255340
H	-5.0070700	-2.5221930	0.1291130	H	5.6874540	1.1942230	0.7226120
H	0.1064630	-3.3201840	-1.5659290	H	2.8536220	-3.3758010	-0.1195240
H	-1.0921350	-2.8333100	2.5556730	H	1.8767680	0.5360430	-1.6468260
H	-1.2348360	-1.2738700	-2.1297460	H	3.1615770	-2.3633480	2.1624190
H	-3.5422030	-3.8217690	1.9281960	H	4.3586760	1.2421890	-1.5791850
H	-2.9946150	-1.3599040	1.7602800	H	2.8891190	1.5404340	0.4477920
H	-2.1151370	-5.3401450	0.4708110	H	4.5877820	-1.1538200	-2.3948340
H	-2.1618360	-4.4828970	-1.9827550	H	5.3290530	-2.6715100	-0.4218590
H	-3.6149410	-2.4050600	-2.1334730	H	5.5563520	-1.2787690	1.6923930
H	-3.0307390	-0.5725340	-0.4855620	H	3.5342590	0.1354510	2.2622890
H	0.1509170	-4.1272330	0.7982390	H	2.1451560	-1.9143500	-2.0010790

H	0.8661760	4.6928100	0.2424290
H	-1.7455500	1.6161180	-2.5762360
H	1.2441380	2.9730770	-0.0252110
H	-3.2666990	0.7151590	-2.3912820
H	-3.2936340	2.4516200	-2.7737010
H	-0.9974860	4.7410200	-2.4007420
H	-2.4506680	4.3848610	-1.4432650
H	-1.2757490	5.5725620	-0.8513990
H	-4.6662710	2.9358660	-0.6606930
H	-4.6773890	1.1883660	-0.3206380
H	-4.0404550	2.3220350	0.8826200
H	-1.4868580	0.5043890	2.1549280
H	-2.9145190	1.3330500	2.8108990
H	-1.2961360	1.6553990	3.4802670
H	-1.6844170	4.6329570	1.3999420
H	-1.3557010	4.0436430	3.0360670
H	4.8676930	-1.9309650	2.4545280
H	5.0832030	-0.3272690	1.7360610
H	5.8445410	-1.7404870	0.9784160
H	4.2582610	-3.4643540	-0.1534690
H	2.5173780	-3.0880450	-0.1312730
H	3.3413020	-3.5509370	1.3703590
H	2.4954720	0.5950740	-3.0212900
H	2.5081560	1.7685850	-1.6831320
H	3.8890160	1.6814170	-2.7829780
H	5.1863650	-1.4647670	-1.3999510
H	2.8066870	1.0218190	3.0327190
H	5.4019750	-0.2508090	-2.6649120
H	1.4518340	1.7937950	2.1638260
H	4.0989100	-1.4554890	-2.8112530
H	3.3941730	3.0348350	0.8627550
H	4.5262300	2.0024920	-0.0377960
H	4.5889340	2.0549060	1.7376220
H	1.5880000	0.0265000	2.2335220
H	-3.0081590	3.9644900	2.3779810
H	1.2440330	4.1011910	-1.3947820
H	-0.3470290	-1.6015060	1.5775050

H	-3.0980580	-4.9103960	-1.0850730
H	-3.1404800	-0.0445490	0.9644970
H	-2.4232750	-5.6352410	0.3925250
H	-3.7573040	-0.7359310	2.4782760
H	-2.1144590	-0.0658000	2.4131220
H	-0.0838010	-5.3399810	-0.2452010
H	-0.5812840	-4.7131510	-1.8336210
H	0.4712840	-3.7361420	-0.7806100
H	-1.5028160	-3.6769510	2.2935950
H	-0.9775750	-2.1607210	3.0636910
H	-2.6118820	-2.7952730	3.3631280
H	-4.6529360	-2.6976690	-1.4437020
H	-4.9024920	-0.9614210	-1.1516520
H	-4.5297590	-2.0395850	0.2037190
H	-1.5237820	-1.0136770	-2.5385400
H	-3.2016060	-0.5399250	-2.8482640
H	-2.8937140	1.0023510	-1.0696400
H	-3.4643050	2.4542150	-1.9351980
H	-4.3084770	1.8975180	-0.4731790
H	-3.1229240	4.7375710	-0.6887770
H	-2.3829030	4.6489550	0.9279170
H	-4.0052690	4.0007210	0.6676790
H	-0.8046980	1.7943080	3.1774350
H	-1.2482340	3.5104240	3.2988310
H	-2.2268010	2.4132220	2.3025530
H	1.4653140	2.3804010	2.3302290
H	-0.7159160	5.6233860	-1.3166840
H	1.0557050	4.1017460	2.4993950
H	-0.1833070	5.2771750	0.3451220
H	1.5923940	3.4738040	0.9305380
H	-0.3027150	3.8262320	-3.0818710
H	0.4042910	2.2874150	-2.5373350
H	1.4052930	3.7543970	-2.5923030
H	1.0162850	5.5228860	-0.9309690
H	-2.7160460	-2.2370120	-3.0550950
H	-3.4809220	-4.2261030	0.5143020
H	0.4386000	0.0744910	1.3270620
H	1.6821890	-1.6451100	1.5486600

I-Ph_2 (dication)

C	3.1403140	-2.6281500	0.8869060
N	2.3459760	-1.3790410	0.7794350
C	1.7092840	-1.0562270	2.0802870
C	1.9933720	-2.2846190	2.9486380
C	3.3133720	-2.8189200	2.3906090
P	2.7530750	-0.1334380	-0.2148840
N	3.8700880	0.8743360	0.4058780
C	5.1665370	0.3691350	0.8989990
C	6.0484540	1.6168220	0.8754390
C	5.0612330	2.7382120	1.2087840
C	3.8000560	2.3515210	0.4329650
N	1.4744090	0.8538200	-0.4829070
P	-0.0575660	0.7419480	-0.5468070
C	-0.8888580	2.2310640	-1.1130400
C	-0.0959370	3.5390050	-1.0554120
C	0.1470010	4.0452280	0.4049180
C	-0.4348940	3.1110550	1.4628530
C	-1.9205260	3.1346710	1.0937570
C	-2.1890290	2.6129550	-0.3722110
C	-0.9564880	5.1283940	0.2047060
C	-2.2187400	4.6514850	0.9653970
C	-2.5844040	3.9073570	-1.1312150
C	-1.2086860	4.6142690	-1.2439350
C	-3.3286600	4.7338510	-0.0828260
N	-0.8798170	-0.4990940	-0.2273840
P	-2.1948840	-1.4043850	0.0384100
N	-1.7398410	-2.8925750	-0.4479800
C	-2.6932320	-4.0237950	-0.4471110
C	-1.8083650	-5.2415140	-0.7472040
C	-0.4259470	-4.8232990	-0.2372680
C	-0.3509660	-3.3545600	-0.6407590
N	-3.4832680	-0.7937830	-0.7633120
C	-4.8959220	-1.0486000	-0.3896700
C	-5.6675590	-0.4964040	-1.5872760
C	-4.7429050	-0.7960720	-2.7679330
C	-3.3572000	-0.4784320	-2.2064450
N	-2.6464710	-1.4633390	1.6122220
C	-3.1590010	-0.2788130	2.3285090
C	-2.1303420	-0.0577520	3.4327020
C	-1.7755400	-1.4903560	3.8495640
C	-1.8821360	-2.3188690	2.5538790
N	3.3299080	-0.8331270	-1.5739870
C	2.5538420	-1.8515760	-2.3148670
C	3.3070730	-1.9729810	-3.6388180
C	3.8261680	-0.5532140	-3.8725780
C	4.2579350	-0.1052310	-2.4764780
H	-3.5302180	5.7562250	-0.4159460

I-Ph_2 (protonated)

C	-0.4283130	-3.5782660	0.7111550
N	-1.6835630	-3.0496750	0.1518670
C	-2.5405190	-4.1379450	-0.3234810
C	-1.5231800	-5.1961850	-0.7455840
C	-0.4434140	-5.0734590	0.3368160
P	-2.0090860	-1.4389790	-0.0018100
N	-2.6889540	-1.3145750	-1.5093080
C	-1.9510430	-1.8118030	-2.6787380
C	-1.6695160	-0.5479950	-3.4892780
C	-2.9464480	0.2860600	-3.2906620
C	-3.5407630	-0.1927410	-1.9416600
N	-0.6397750	-0.7307770	0.3332760
P	-0.0078470	0.7357140	0.4226380
N	1.5231790	0.8481900	-0.0373590
P	2.7379270	-0.1458100	0.2259840
N	3.1043860	-0.6154720	1.7842830
C	3.6466110	0.3801390	2.7192880
C	2.4386460	0.8025140	3.5602780
C	1.6147590	-0.4947530	3.6877610
C	2.1191870	-1.4114480	2.5428450
C	-0.9656940	1.9451820	-0.5454070
C	-0.2722400	3.0979640	-1.2785950
C	0.2474860	4.2357810	-0.3440130
C	-0.0171040	3.9509210	1.1303050
C	-1.5485590	3.8373340	1.1355170
C	-2.0930230	2.6965420	0.1854350
C	-0.9481450	5.1362980	-0.7782790
C	-1.9763320	5.1272350	0.3820650
C	-2.7369280	3.5008200	-0.9752380
C	-1.4685130	4.0036180	-1.7119040
C	-3.2910320	4.7562960	-0.3031680
N	-3.2149520	-0.8471890	0.9756790
C	-4.6080710	-1.3379560	0.8774760
C	-5.0213460	-1.5848240	2.3274530
C	-4.2391260	-0.5086630	3.0821440
C	-2.8816270	-0.5171470	2.3793480
N	2.5194700	-1.6040350	-0.5189870
C	3.2516740	-2.8439220	-0.1994150
C	3.2910640	-3.5953040	-1.5319330
C	2.0076440	-3.1392230	-2.2309220
C	1.9234090	-1.6579390	-1.8644480
N	4.0630360	0.6549750	-0.3293130
C	5.3731230	0.0002040	-0.4508870
C	6.3258850	1.1669960	-0.7221930
C	5.4444340	2.1652310	-1.4778840
C	4.1004670	2.0628050	-0.7549010
H	-3.6304580	5.5102680	-1.0205330

H	-4.2665030	4.2731690	0.2422270	H	-4.1021430	4.5445810	0.4010100
H	1.1622550	4.3896000	0.6016960	H	1.2549860	4.5876160	-0.5703280
H	-1.1306710	1.9928950	-2.1585880	H	-1.4231900	1.3118940	-1.3142920
H	-0.2924900	3.5284390	2.4643270	H	0.2839450	4.8087100	1.7420140
H	-3.0921010	3.7247280	-2.0797870	H	-3.4312840	2.9209400	-1.5874900
H	-2.9551560	1.8350970	-0.4051840	H	-2.8010260	2.0367710	0.6955440
H	-1.1100390	5.3134000	-2.0736920	H	-1.5978080	4.2193980	-2.7731450
H	-0.6909110	6.1762490	0.3420080	H	-0.7418310	6.1138900	-1.2153690
H	-2.3983310	5.1468780	1.9207170	H	-1.9761240	6.0236100	1.0055390
H	-2.5616520	2.6338240	1.8213530	H	-1.9796640	3.7576820	2.1359170
H	0.7657450	3.5703410	-1.7204040	H	0.4226000	2.7723410	-2.0526950
H	2.6613190	-2.3357140	-4.4400130	H	1.8733650	1.5691270	3.0198000
H	4.1433590	-2.6699940	-3.5280390	H	2.7258090	1.2188740	4.5281180
H	4.6492200	-0.5074310	-4.5875060	H	1.7827330	-0.9727020	4.6557300
H	3.0171570	0.0884770	-4.2356110	H	0.5416760	-0.3036820	3.6010150
H	5.2864520	-0.4106750	-2.2580330	H	2.6150400	-2.3010360	2.9444950
H	4.1860810	0.9759330	-2.3415030	H	1.3128220	-1.7418020	1.8857540
H	1.5258200	-1.5050970	-2.4824830	H	4.1264470	1.2008120	2.1830030
H	2.5167020	-2.7929130	-1.7624130	H	4.4052210	-0.1063130	3.3439530
H	6.4592080	1.7591720	-0.1291890	H	3.3471660	-4.6767970	-1.3926860
H	5.5501640	-0.4303240	0.2583660	H	2.7048800	-3.4180590	0.5595070
H	5.0581080	-0.0227500	1.9174060	H	4.2477140	-2.6364370	0.1977960
H	4.1529480	-2.2170480	2.7530980	H	5.8404240	3.1826490	-1.4683190
H	3.8170870	2.7560460	-0.5850950	H	0.8977960	-1.2842080	-1.8357140
H	3.4989430	-3.8617840	2.6526050	H	5.3307410	1.8509770	-2.5206580
H	5.4226860	3.7277320	0.9244660	H	1.1439940	-3.6766910	-1.8231620
H	2.5688810	-3.4586320	0.4556000	H	3.2487140	2.2992690	-1.3946930
H	2.8830100	2.6854690	0.9209300	H	2.4974160	-1.0469280	-2.5742550
H	4.0830210	-2.5462690	0.3435160	H	4.0681740	2.7340210	0.1135120
H	1.2058120	-3.0342800	2.8160600	H	7.2098990	0.8567060	-1.2824280
H	2.1588340	-0.1551130	2.5145960	H	5.6296890	-0.5441040	0.4638960
H	2.0403460	-2.0272680	4.0077960	H	6.6566620	1.6026770	0.2264220
H	0.6392510	-0.8770170	1.9457170	H	5.3705230	-0.7144830	-1.2849140
H	-4.9682820	-0.2030930	-3.6556420	H	-1.9547400	-6.1974090	-0.8057510
H	-4.8032420	-1.8554520	-3.0358480	H	-1.1173240	-4.9368770	-1.7295920
H	-3.1177380	0.5834040	-2.3340670	H	-3.1837880	-4.5151230	0.4835690
H	-3.4698080	-3.8788290	-1.2032060	H	-4.6744960	-2.2351370	0.2568450
H	-3.1762360	-4.1143640	0.5325220	H	-5.2362100	-0.5665720	0.4169220
H	-0.3752640	-4.9134800	0.8530810	H	-4.7212780	0.4662030	2.9541710
H	-2.1905790	-6.1449530	-0.2698510	H	-6.1017540	-1.5166220	2.4688220
H	-1.7698420	-5.4164100	-1.8264950	H	-4.6931730	-2.5804770	2.6445770
H	0.3859950	-5.4103360	-0.6707230	H	-4.1458580	-0.7060590	4.1516310
H	0.3468970	-2.7624710	-0.0451960	H	-2.3729140	0.4490880	2.4428620
H	-0.0705480	-3.2509900	-1.6951680	H	-2.2229880	-1.2768230	2.8197470
H	-5.8016830	0.5844620	-1.4769700	H	-0.7210900	-5.6753100	1.2069280
H	-5.1485410	-0.5391310	0.5412690	H	-0.3836150	-3.4412830	1.7970430
H	-6.6519790	-0.9575070	-1.6809050	H	0.5378230	-5.4145890	-0.0021680
H	-3.2719170	0.5676020	1.6486860	H	-1.0521690	-2.3519640	-2.3735590
H	-4.1433560	-0.5105120	2.7493180	H	-2.5850590	-2.5067430	-3.2436400
H	-2.5254310	0.5383190	4.2571280	H	-1.4612610	-0.7591030	-4.5400330
H	-1.2550910	0.4611320	3.0281300	H	-0.7979250	-0.0323330	-3.0704960
H	-0.7820970	-1.5682490	4.2958250	H	-2.7393790	1.3594370	-3.2869960
H	-2.4072830	-3.2636650	2.7139150	H	-4.5701910	-0.5414860	-2.0717560
H	-0.8969540	-2.5545320	2.1422710	H	-3.5562700	0.5881220	-1.1779340
H	-5.0826520	-2.1201420	-0.2608040	H	0.4154360	-3.0395380	0.2737530
H	-2.4994770	-1.8547780	4.5827680	H	-3.6564340	0.0946900	-4.0988120
H	6.8773990	1.5466160	1.5813960	H	4.1638920	-3.2783290	-2.1124540
H	4.8506010	2.7452030	2.2826830	H	2.0229900	-3.2959460	-3.3110630
H	-2.5664880	-1.0710920	-2.6753520	H	-3.1802460	-3.7983110	-1.1405690
H	0.0271620	2.1157810	1.4944800	H	-0.1269430	1.1360840	1.7715900
				H	0.5246290	3.0846650	1.5150930
**II-Pa$_2$ (dication)**				**II-Pa$_2$ (protonated)**			
C	-2.9940050	-1.5224940	-1.0528890	C	3.7809370	-0.4053150	1.0609870
C	-3.1711050	-0.0125770	-1.2886040	C	3.8950300	0.5776170	-0.1175020
C	-4.5335280	0.5163680	-0.8538700	C	4.5820510	-0.0181700	-1.3412400
C	-4.7845610	0.1802200	0.6306200	C	3.8266240	-1.2816820	-1.8015220
C	-3.5818950	-0.5581670	1.2074580	C	2.6348410	-1.5417810	-0.8858050
C	-3.2364160	-1.8518720	0.4507950	C	3.0210710	-1.6838700	0.5975900
C	-2.2383420	0.2001700	1.0754890	C	1.6276950	-0.3669410	-0.7912640
C	-1.9903960	0.5379530	-0.4479320	C	2.3995770	0.9271780	-0.3218400
C	-1.4630940	-1.7533510	-0.8543990	C	2.5239870	0.0540040	1.8685840
C	-0.8742630	-0.3485710	-1.0571190	C	2.0404890	1.3023490	1.1250890
C	-1.7019020	-2.0660840	0.6579820	C	1.7638600	-1.2319320	1.4119280
C	-1.2850540	-0.9023300	1.5605650	C	0.7190980	-0.9508800	0.3165010
P	0.8045250	-0.1329440	-0.4591020	P	-0.8410660	-0.1189480	0.7893960
N	1.6180140	-1.4450910	-0.4572630	N	-1.1469120	1.0343060	-0.2855770
P	3.1583770	-1.8031570	-0.0001490	P	-1.5741370	2.5675200	-0.2144320
C	4.3599040	-0.7566690	-0.8393050	C	-3.0351500	2.8740650	-1.2325610
N	1.3755040	1.2391250	-0.1000220	N	-1.9864760	-1.2309270	1.0179830
P	1.2743340	2.8527200	0.0697050	P	-2.9220360	-1.9447000	-0.0669690
C	0.1843420	3.2612020	1.4423910	C	-4.0616040	-0.8135710	-0.9065580
C	2.9238690	3.4706060	0.4244560	C	-2.0310590	-2.8077830	-1.3920050
C	0.6750820	3.6155940	-1.4467000	C	-3.9223070	-3.1823760	0.7820960
C	3.3263850	-1.6015990	1.7807520	C	-1.9593510	3.1700910	1.4512680
C	3.4371790	-3.5198100	-0.4433000	C	-0.2658500	3.6359760	-0.8621390
H	-0.9752990	-2.5307170	-1.4398430	H	2.6288580	0.1753470	2.9475710

H	-1.4814600	-1.1343590	2.6108820
H	-0.7789790	-0.1228690	-2.1278470
H	-3.7553490	-0.7766740	2.2660930
H	-2.2059900	1.1037360	1.6880630
H	-3.9008330	-2.6907350	0.6609870
H	-3.5032900	-2.1522790	-1.7828900
H	-3.0030940	0.2298370	-2.3430800
H	-1.8355520	1.6067830	-0.6109540
H	-1.3590640	-3.0464150	0.9873350
H	-5.3027240	0.0725460	-1.4933720
H	-4.5698830	1.5968960	-1.0243660
H	-5.6759790	-0.4445440	0.7449120
H	-4.9626410	1.0901430	1.2122580
H	2.5751210	-2.2151040	2.2815820
H	3.1787850	-0.5521350	2.0439890
H	4.3249340	-1.9178110	2.0893240
H	3.3214730	-3.6360500	-1.5224680
H	2.7065560	-4.1451590	0.0726170
H	4.4465690	-3.8132920	-0.1479320
H	4.2421890	-0.8652670	-1.9191300
H	5.3683120	-1.0602590	-0.5492030
H	4.1950760	0.2841650	-0.5545520
H	3.2931360	2.9988360	1.3368900
H	2.8898220	4.5540490	0.5580640
H	3.5855430	3.2263720	-0.4086200
H	-0.3378290	3.2734930	-1.6660290
H	0.6729340	4.7012670	-1.3280340
H	1.3381570	3.3401090	-2.2690080
H	0.1973240	4.3410780	1.6046720
H	-0.8346370	2.9438720	1.2158390
H	0.5345490	2.7510420	2.3416060
H	-0.2142560	-0.6496160	1.5380640

H	0.3559170	-1.9219500	-0.0401090
H	2.6165290	2.1805950	1.4350620
H	2.1055710	-2.4386260	-1.2264850
H	1.0918230	-0.1896340	-1.7259730
H	3.4748650	-2.6424470	0.8559420
H	4.7118160	-0.5659830	1.6079830
H	4.4270270	1.4817300	0.1988560
H	2.2516390	1.7437510	-1.0329410
H	1.4149540	-1.8884870	2.2106760
H	5.6225980	-0.2525430	-1.0926710
H	4.6128660	0.7314450	-2.1389990
H	4.4835730	-2.1578510	-1.7893900
H	3.4710670	-1.1727920	-2.8315470
H	-2.2623440	4.2185200	1.4045190
H	-1.0800780	3.0855490	2.0940550
H	-2.7740200	2.5788790	1.8754760
H	-0.5942100	4.6775750	-0.8807010
H	-0.0150140	3.3140170	-1.8752150
H	0.6206690	3.5447890	-0.2306300
H	-3.2589220	3.9427360	-1.2628040
H	-3.8881180	2.3361940	-0.8137650
H	-2.8451110	2.5151830	-2.2463920
H	-1.3641200	-3.5564580	-0.9590070
H	-1.4413650	-2.0880470	-1.9656590
H	-2.7398200	-3.3013530	-2.0612120
H	-4.5303540	-2.6909530	1.5440080
H	-4.5730440	-3.6933450	0.0693250
H	-3.2638260	-3.9097330	1.2609520
H	-4.7352750	-1.3679720	-1.5638460
H	-4.6441370	-0.2702650	-0.1594250
H	-3.4770600	-0.1023120	-1.4938190
H	-0.6422490	0.4110310	2.0780380
H	0.9919500	1.5498090	1.2917290

II-Pb$_2$ (dication)

C	-1.9953690	-1.7219490	-0.3090870
C	-2.2825740	-1.4377430	1.2007170
C	-1.9729000	0.0129820	1.5689280
C	-2.9715990	0.7638370	0.6723260
C	-2.6967620	0.4925280	-0.8605540
C	-1.4996700	-0.4675780	-1.0525530
C	-3.8156970	-1.4356840	0.9039280
C	-4.2690120	0.0239850	1.0825410
C	-3.8016100	-0.4364500	-1.4215780
C	-3.5308240	-1.7099830	-0.6030600
C	-5.2152070	0.1073980	-1.2437080
C	-5.4930700	0.3852440	0.2481990
P	0.1145420	0.1572360	-0.5829220
N	1.2502860	-0.7930660	-0.2110700
P	1.9403000	-2.2341670	0.0855470
C	1.7241840	-3.3473100	-1.3280840
C	2.2344510	-2.7676330	-2.6491590
N	0.2498850	1.6886630	-0.7004340
P	1.4323800	2.7675880	-0.3096380
C	2.2030870	2.3188630	1.2706360
C	3.3074730	3.2846630	1.7072570
C	2.6893300	2.8492140	-1.6112460
C	3.5133390	1.5690220	-1.7675230
C	0.6146340	4.3737370	-0.1773900
C	-0.4676610	4.4037050	0.9043180
C	3.6989160	-1.9175340	0.3752290
C	4.5025870	-3.1569740	0.7749410
C	1.1941510	-2.9827690	1.5555330
C	1.3093570	-2.1082260	2.8064330
H	-1.4498380	-2.6368800	-0.5421260
H	-2.1922450	0.2055850	2.6225130
H	-1.3733830	-0.6944620	-2.1196900
H	-4.4697460	0.2272210	2.1394420
H	-3.0129490	1.8363520	0.8721890
H	-4.4345170	-2.1699590	1.4205280
H	-3.9608290	-2.6182000	-1.0259680
H	-3.6103880	-0.6199630	-2.4839620
H	-2.5891540	1.4212840	-1.4219810
H	-1.9013680	-2.1809350	1.9019830
H	-5.9258360	-0.6207520	-1.6470700
H	-5.3267060	1.0177960	-1.8407210
H	-6.3512100	-0.1953460	0.6010300
H	-5.7399550	1.4392640	0.4095700
H	2.2390130	-4.2805080	-1.0763600
H	0.6557270	-3.5777520	-1.3860180
H	3.7460490	-1.1454640	1.1493870
H	4.0879850	-1.4665220	-0.5427420
H	1.6758010	-3.9551160	1.6995800
H	0.1480110	-3.1821480	1.3030400
H	1.3981050	2.2751080	2.0109090
H	2.5864240	1.3001410	1.1582720
H	3.3291510	3.7042000	-1.3681310

II-Pb$_2$ (protonated)

C	-2.8918540	-0.9309770	-1.8514550
C	-2.2084800	-1.5572250	-0.5934560
C	-1.3664330	-0.5363190	0.1915730
C	-2.4596940	0.4991240	0.5556750
C	-3.1588690	1.1198260	-0.7156970
C	-2.5595030	0.5556760	-2.0129760
C	-3.5800340	-1.5471720	0.1573780
C	-3.4519200	-0.4694240	1.2487980
C	-4.5922300	0.5449920	-0.8348530
C	-4.2639190	-0.9345440	-1.1013940
C	-5.4709820	0.7595020	0.3926050
C	-4.7839710	0.1632970	1.6381710
P	0.2388180	-0.0130670	-0.5063080
N	0.3549620	1.5898180	-0.4603760
P	1.5969580	2.4385300	0.0986150
C	1.1638610	4.1863230	-0.1375890
C	2.2327950	5.1869830	0.3028070
N	1.4370610	-0.7523650	0.2686780
P	2.1669510	-2.1677800	0.2549950
C	3.6774640	-2.1425840	-0.7600230
C	3.4122490	-1.7735540	-2.2201660
C	2.6980520	-2.5624790	1.9457650
C	1.5335380	-2.6331900	2.9342530
C	1.1082760	-3.5284710	-0.3429850
C	1.7675930	-4.9089740	-0.3656110
C	1.9522980	2.1606660	1.8646150
C	0.7201510	2.2925570	2.7595370
C	3.1742720	2.1165920	-0.7565380
C	3.0987550	2.3345600	-2.2680100
H	-2.8116620	-1.5210660	-2.7655650
H	-1.0422500	-1.0055370	1.1298070
H	-3.0915950	0.9698410	-2.8759170
H	-2.9760500	-0.8902370	2.1414480
H	-2.0939470	1.2811410	1.2244920
H	-3.9715420	-2.5029590	0.5106150
H	-5.0843790	-1.5096690	-1.5347920
H	-5.0752450	0.9859560	-1.7139090
H	-3.1423080	2.2114180	-0.6699680
H	-1.7301740	-2.5258760	-0.7493210
H	-6.4476050	0.2934930	0.2233180
H	-5.6548450	1.8311550	0.5229820
H	-5.4161180	-0.5969990	2.1090820
H	-4.6070070	0.9342410	2.3955750
H	0.9307390	4.2990120	-1.2009910
H	0.2259100	4.3434240	0.4041510
H	2.7418220	2.8577420	2.1642570
H	2.3640150	1.1483610	1.9240840
H	3.9470570	2.7435600	-0.3000090
H	3.4239260	1.0766260	-0.5217200
H	4.1660460	-3.1186690	-0.6776890
H	4.3445450	-1.4123580	-0.2894020
H	3.4005880	-1.7719210	2.2291950

H	2.1593970	3.0973830	-2.5357690
H	1.3883000	5.1258500	0.0051260
H	0.1923540	4.5771350	-1.1664550
H	0.7718300	-2.5835810	3.6284570
H	2.3501040	-1.9816480	3.1139290
H	0.8757730	-1.1173830	2.6476850
H	5.5491290	-2.8763410	0.9042930
H	4.1523500	-3.5795540	1.7200190
H	4.4596260	-3.9358720	0.0097510
H	3.3097440	-2.5767460	-2.6201920
H	2.0436190	-3.4832370	-3.4505090
H	1.7223910	-1.8359670	-2.9010690
H	3.7049550	2.9597590	2.6701790
H	4.1350970	3.3030020	0.9939060
H	2.9313690	4.3033800	1.8286280
H	2.8852700	0.7162690	-2.0345460
H	4.2431780	1.7093020	-2.5665530
H	4.0613650	1.3249250	-0.8537340
H	-0.0423970	4.2831000	1.9036820
H	-0.9823210	5.3654360	0.8736130
H	-1.2078750	3.6159150	0.7424530
H	-0.9182460	0.3048150	1.4568740

H	3.2625370	-3.4999260	1.9201550
H	0.2232360	-3.5298370	0.3022740
H	0.7649930	-3.2458640	-1.3442990
H	0.2179740	-0.4336680	-1.8553440
H	2.1037430	-5.2123760	0.6293320
H	1.0479790	-5.6529600	-0.7137130
H	2.6260670	-4.9357360	-1.0416400
H	2.7793140	-2.5147380	-2.7161550
H	2.9215410	-0.7988830	-2.2982590
H	4.3544510	-1.7176390	-2.7690530
H	1.9126250	-2.7913110	3.9460720
H	0.9607530	-1.7018480	2.9250840
H	0.8548340	-3.4580800	2.6997490
H	1.8802130	6.2045380	0.1215320
H	2.4562400	5.0992650	1.3693320
H	3.1647630	5.0554870	-0.2537730
H	2.2889070	1.7487420	-2.7126220
H	2.9281720	3.3864020	-2.5142480
H	4.0358920	2.0282870	-2.7379910
H	-0.0549330	1.5800950	2.4639010
H	0.9898450	2.0871600	3.7978220
H	0.2919620	3.2981480	2.7171720
H	-1.5045680	0.7855360	-2.1652310

II-Pc$_2$ (dication)

C	1.8809430	0.2094200	1.3060750
C	2.8838700	1.3131530	0.8881020
C	4.0541220	0.8901660	1.8094560
C	4.1569380	-0.6025840	1.4548140
C	2.7111610	-1.0619940	1.0743190
C	4.5970680	-0.7572540	-0.0319110
C	4.7796780	0.6346600	-0.6624250
C	5.7768770	1.5124510	0.0857980
C	5.3456360	1.6619840	1.5595150
C	3.3182680	1.1454420	-0.6219940
C	2.6247760	-0.0599720	-1.2796030
C	3.1531890	-1.2071470	-0.4177830
P	0.2649650	0.2878790	0.5334070
N	-0.2254680	1.7381620	0.3087990
P	-1.6537730	2.2809360	-0.3239360
C	-1.4865570	4.0754370	-0.4208520
C	-2.7305820	4.8025210	-0.9464150
C	-2.4899960	6.3085700	-1.0254470
N	-0.5538980	-0.9582980	0.2199120
P	-1.0509950	-2.5026230	0.2139970
C	-1.4722280	-2.9665390	1.9123140
C	-2.5127240	-2.0424560	2.5598160
C	-2.7898920	-2.4439430	4.0063780
C	-2.5070880	-2.5981900	-0.8569240
C	-3.0734420	-4.0137170	-1.0294180
C	-4.3109650	-4.0013500	-1.9241870
C	0.2337920	-3.6026340	-0.4331710
C	0.5747010	-3.3557510	-1.9092590
C	1.6652810	-4.3091660	-2.3915630
C	-1.9195070	1.5549030	-1.9617130
C	-0.7785290	1.8186120	-2.9540690
C	-0.8962140	0.9132380	-4.1775260
C	-3.0392920	1.8089350	0.7435860
C	-2.8840710	2.2443630	2.2058430
C	-4.0324270	1.7147190	3.0615620
H	2.3200760	-1.9541300	1.5640770
H	2.9517380	-0.1642970	-2.3178490
H	1.6423820	0.3174600	2.3724460
H	5.0878390	0.5345930	-1.7081690
H	3.1748430	2.0786970	-1.1698290
H	5.4139080	-1.4533550	-0.2247840
H	4.6913160	-1.2052630	2.1895660
H	3.7496160	1.0129380	2.8540840
H	2.5162040	2.3189370	1.0940640
H	3.0318690	-2.2011630	-0.8472510
H	6.1183770	1.2830720	2.2356630
H	5.1906520	2.7137600	1.8191410
H	6.7728220	1.0643420	0.0132750
H	5.8358990	2.4889780	-0.4049470
H	-0.5336920	-2.9522620	2.4774340
H	-1.8236260	-4.0039860	1.8896590
H	-3.2601490	-1.9259430	-0.4329110
H	-2.2120310	-2.1742320	-1.8236180
H	1.1162540	-3.4747630	0.2025780
H	-0.1185490	-4.6296150	-0.2823930
H	-1.2235930	4.4171290	0.5860500
H	-0.6157950	4.2679030	-1.0575620
H	-2.8764660	1.9247820	-2.3445740
H	-2.0420930	0.4799090	-1.7870930
H	-3.1240210	0.7191130	0.6687950
H	-3.9495780	2.2259750	0.2986100
H	-1.9301810	1.8782050	2.6000760

II-Pc$_2$ (protonated)

C	-3.2122530	-0.7164790	-2.2486560
C	-2.5558020	-1.5306150	-1.0882840
C	-1.8052430	-0.6347530	-0.0881750
C	-2.9636410	0.2705690	0.3963410
C	-3.6340360	1.0812820	-0.7796870
C	-2.9467710	0.7867300	-2.1222140
C	-3.9578330	-1.7163160	-0.4223530
C	-3.9350050	-0.8499170	0.8498590
C	-5.0292920	0.4775520	-1.0767260
C	-4.6146810	-0.9142330	-1.5859370
C	-5.9723660	0.4291010	0.1205180
C	-5.3142420	-0.3561120	1.2735800
P	-0.1933870	0.0592830	-0.5938850
N	-0.1483130	1.6387050	-0.3037030
P	1.0823690	2.4330920	0.3573550
C	2.6644610	2.2476010	-0.5270560
C	2.5831660	2.5755160	-2.0206780
C	3.9152980	2.3305560	-2.7260380
N	1.0047090	-0.7370370	0.1320960
P	1.6471070	-2.1851080	-0.0739260
C	0.5283000	-3.5411720	0.3972750
C	0.0912720	-3.4788100	1.8667570
C	-1.1316500	-4.3540100	2.1297520
C	2.1528600	-2.5101510	-1.7928610
C	3.0383230	-1.4025960	-2.3750060
C	3.3795370	-1.6511460	-3.8415580
C	3.1077560	-2.2607740	0.9986680
C	3.8557970	-3.5970200	1.0055840
C	5.0533030	-3.5602190	1.9529330
C	0.6114040	4.1859110	0.3500970
C	1.6616670	5.1602320	0.8913390
C	1.1536340	6.6004980	0.8630640
C	1.4427240	1.9257840	2.0689140
C	0.2037110	1.8574140	2.9653160
C	0.5354860	1.2830590	4.3405200
H	-3.0604550	-1.1273690	-3.2477680
H	-1.5084800	-1.2518400	0.7691720
H	-3.4569430	1.3294780	-2.9249590
H	-3.4787320	-1.4053580	1.6770560
H	-2.6683590	0.9314920	1.2141250
H	-4.3142790	-2.7364290	-0.2673140
H	-5.3842680	-1.4349900	-2.1587820
H	-5.4938910	1.0496570	-1.8874700
H	-3.6750180	2.1462250	-0.5388330
H	-2.0209270	-2.4326920	-1.3924870
H	-6.9161580	-0.0370760	-0.1819740
H	-6.2144060	1.4507540	0.4316810
H	-5.9274620	-1.2159320	1.5634480
H	-5.2125060	0.2695300	2.1665850
H	3.4130080	2.8715590	-0.0253770
H	2.9644840	1.2036110	-0.3780740
H	-0.3178340	4.2533730	0.9270620
H	0.3517110	4.4286140	-0.6862050
H	1.9027940	0.9351290	1.9853020
H	2.1952250	2.6071870	2.4817930
H	1.0064560	-4.4989750	0.1648340
H	-0.3413720	-3.4568260	-0.2651630
H	1.2325320	-2.6146250	-2.3803810
H	2.6586700	-3.4815600	-1.8270220
H	3.7659520	-1.4424760	0.6860240
H	2.7520420	-2.0063790	2.0037770
H	-0.1187090	-0.1634630	-1.9899360

H	-2.8523310	3.3373780	2.2652530	H	3.1769300	-4.4021270	1.3084560
H	-0.7941290	2.8692160	-3.2603780	H	4.1994870	-3.8386950	-0.0061790
H	0.1895180	1.6496420	-2.4694160	H	0.9225190	-3.7946730	2.5067120
H	-2.9967130	4.4229080	-1.9387760	H	-0.1348870	-2.4421770	2.1428740
H	-3.5839410	4.6030860	-0.2897130	H	2.5229240	-0.4405890	-2.2768690
H	-0.3198360	-3.4926600	-2.5254870	H	3.9623410	-1.3216890	-1.7907810
H	0.8967180	-2.3179630	-2.0486280	H	1.7987220	1.9662120	-2.4851760
H	-2.1506900	-1.0087700	2.5301980	H	2.2830480	3.6208380	-2.1569350
H	-3.4458280	-2.0718240	1.9867280	H	-0.2333220	2.8564590	3.0758430
H	-2.3134010	-4.6693990	-1.4674470	H	-0.5559780	1.2345310	2.4802520
H	-3.3328100	-4.4349800	-0.0523950	H	1.9293590	4.8892570	1.9185230
H	-4.9952710	2.0905520	2.7033520	H	2.5787930	5.0867300	0.2964450
H	-4.0674300	0.6207550	3.0376880	H	-0.3560960	1.2402790	4.9718710
H	-3.9114430	2.0262480	4.1017050	H	0.9353050	0.2677390	4.2497340
H	-0.0922100	1.1195180	-4.8879850	H	1.2845590	1.8947090	4.8532030
H	-0.8313500	-0.1404570	-3.8869430	H	0.9052600	6.9072960	-0.1577500
H	-1.8506970	1.0656910	-4.6898520	H	0.2534160	6.7095630	1.4758840
H	-3.3808050	6.8204240	-1.3971930	H	1.9109670	7.2881300	1.2484030
H	-2.2487900	6.7179710	-0.0399780	H	3.8380150	2.5493990	-3.7942880
H	-1.6598160	6.5368320	-1.7004230	H	4.7028730	2.9637130	-2.3058170
H	-4.0714010	-3.6097880	-2.9173000	H	4.2312510	1.2879540	-2.6162270
H	-4.7071980	-5.0123800	-2.0442600	H	4.7333610	-3.3515430	2.9785160
H	-5.0986220	-3.3762870	-1.4932470	H	5.5806060	-4.5176100	1.9506410
H	2.5803150	-4.2019510	-1.8006930	H	5.7625940	-2.7815390	1.6558910
H	1.3358610	-5.3491020	-2.3106450	H	3.9247650	-2.5919110	-3.9646090
H	1.9126100	-4.1104310	-3.4368650	H	2.4712290	-1.7047040	-4.4496970
H	-3.5253050	-1.7713840	4.4550730	H	4.0030690	-0.8440110	-4.2358390
H	-3.1835330	-3.4629980	4.0623270	H	-1.4161130	-4.3207670	3.1846350
H	-1.8769900	-2.3982160	4.6074710	H	-1.9871550	-4.0115910	1.5373210
H	1.5273870	-0.0056090	-1.3496220	H	-0.9343930	-5.3969960	1.8635120
				H	-1.8979260	1.0816320	-2.1713010
**II-Pd$_2$ (dication)**				**II-Pd$_2$ (protonated)**			
C	6.4308320	-0.8086700	-0.3478010	C	-1.3733680	-1.1145760	-0.8850010
C	5.0959030	-1.2994070	-0.8975160	C	-2.7454520	-0.9723640	-0.1843220
C	3.9668780	-0.2394820	-0.8839960	C	-3.0573820	-2.4800250	-0.0132200
C	3.7912000	0.2966580	0.5927620	C	-2.8468160	-2.9609310	-1.4596070
C	4.7925430	-0.4327110	1.5226700	C	-1.7636770	-2.0117070	-2.0719270
C	6.2492980	-0.2915900	1.0943610	C	-3.9271430	-2.3191530	-2.3814450
C	2.4881420	-0.2360640	1.2335850	C	-4.8486650	-1.4181350	-1.5398880
C	2.7231330	-1.7558250	1.1828830	C	-5.5203430	-2.1501380	-0.3826850
C	2.9093290	-2.2703800	-0.2811150	C	-4.4489960	-2.7768550	0.5334250
C	2.7911470	-1.1375140	-1.3015190	C	-3.8386490	-0.3226520	-1.1163480
C	4.2632250	-1.8734760	1.4202410	C	-3.2586610	0.0269280	-2.4963810
C	4.4467440	-2.3968350	-0.0360010	C	-2.8489010	-1.3638140	-2.9887430
P	0.9421110	0.3339850	0.5280580	P	-0.3031950	0.3485160	-1.1652300
N	-0.2960210	-0.5412990	0.6311660	N	1.1512770	0.0620660	-0.5614200
P	-1.3888900	-1.7096150	0.8695430	P	2.3593010	-0.9665530	-0.5846820
C	-1.6093750	-1.9934740	2.6402510	C	1.8690120	-2.7205690	-0.6861910
C	-2.1007150	-0.7525600	3.3980060	C	1.2100550	-3.2485200	0.5955990
C	-2.1440430	-0.9753680	4.9128440	C	0.3501920	-4.4855710	0.3402970
C	-3.1214270	-2.0657180	5.3486710	C	-0.3120910	-5.0086500	1.6116290
N	1.0164960	1.7471370	-0.0882380	N	-0.9626030	1.6959620	-0.5948690
P	-0.0576030	2.6777670	-0.9246940	P	-0.7059530	2.4565790	0.7908470
C	-1.7373130	2.4804460	-0.2788700	C	1.0393450	2.8794520	1.0954390
C	-2.7990720	3.3246090	-0.9916120	C	1.7263500	3.5719150	-0.0829690
C	-4.2036040	2.9655060	-0.5022940	C	3.2305810	3.7225940	0.1427430
C	-5.2848230	3.8063730	-1.1752320	C	3.9357320	4.3854970	-1.0373900
C	0.4949260	4.3867320	-0.7628310	C	-1.6582270	3.9989910	0.7427940
C	0.5176430	4.8812920	0.6880910	C	-3.1613830	3.7742370	0.5358890
C	1.0760750	6.3013140	0.7893030	C	-3.9320170	5.0767910	0.3010410
C	1.1006690	6.8096110	2.2284780	C	-3.8818760	6.0487200	1.4789960
C	-0.0063110	2.1840820	-2.6669350	C	-1.2686250	1.5497550	2.2722240
C	-0.6005220	0.7968530	-2.9382290	C	-0.4338350	0.3105780	2.6172110
C	-0.3808430	0.3546620	-4.3849040	C	-1.1221870	-0.5919760	3.6405550
C	-0.8993580	-1.0585140	-4.6374570	C	-0.2257770	-1.7423210	4.0921720
C	-2.9555430	-1.1676520	0.1418110	C	3.5124480	-0.6754760	-1.9587110
C	-4.1014770	-2.1787370	0.2538320	C	4.0545730	0.7578330	-1.9757250
C	-5.3936360	-1.6136630	-0.3392660	C	4.9175430	1.0639140	-3.2024300
C	-6.5527550	-2.6024910	-0.2502280	C	6.1900950	0.2226430	-3.2868320
C	-0.8275730	-3.2292260	0.0666090	C	3.2584040	-0.7538340	0.9765080
C	-0.5707740	-3.0536950	-1.4352620	C	4.5115470	-1.6186680	1.1399120
C	-0.0858730	-4.3393340	-2.1104110	C	5.1067270	-1.5289370	2.5494100
C	-1.1198070	-5.4636530	-2.1068060	C	4.2055350	-2.1151210	3.6361090
H	2.0656960	-2.3377040	1.8279280	H	-2.6543560	-1.4334950	-4.0598530
H	2.9365320	-1.5083150	-2.3199670	H	-0.7506810	-1.7127600	-0.2141540
H	2.4257570	0.0986420	2.2773040	H	-4.0483230	0.4379630	-3.1341560
H	5.2284300	-1.6577870	-1.9235060	H	-2.3059540	-2.9247070	0.6493890
H	4.1462340	0.5865860	-1.5744940	H	-2.6942390	-4.0375450	-1.5603320
H	4.8982850	-3.3836900	-0.1419230	H	-4.4512850	-2.9978180	-3.0568420
H	4.5928360	-2.5330330	2.2234300	H	-5.6126480	-0.9631720	-2.1799330
H	4.6724580	-0.0558970	2.5437830	H	-4.3148780	0.5258770	-0.6185480
H	3.8735590	1.3831000	0.6403770	H	-0.9226280	-2.4768420	-2.5908420
H	2.3587780	-3.1773650	-0.5327220	H	-6.1910540	-2.9181660	-0.7823060
H	6.8778290	-0.8507240	1.7945510	H	-6.1478570	-1.4467600	0.1746700
H	6.5470310	0.7583510	1.1760930	H	-4.5799370	-3.8615270	0.6078680
H	7.1547810	-1.6289410	-0.3810170	H	-4.5240450	-2.3827580	1.5526540

H	6.8204280	-0.0201280	-0.9992300	H	3.5044390	0.3104340	1.0601450
H	1.4943720	4.4291980	-1.2095520	H	2.5191440	-0.9617110	1.7578590
H	-0.1651520	5.0023950	-1.3840320	H	4.2792950	-2.6689220	0.9212440
H	-0.4965740	4.8618320	1.1044840	H	5.2650720	-1.3058940	0.4085350
H	1.1276940	4.2035330	1.2963580	H	6.0640750	-2.0608880	2.5509530
H	2.0898230	6.3187090	0.3718340	H	5.3333270	-0.4806540	2.7791120
H	0.4704440	6.9719900	0.1683590	H	4.7055770	-2.0952460	4.6082330
H	1.5055420	7.8237900	2.2801620	H	3.2697460	-1.5568430	3.7407750
H	0.0928550	6.8274190	2.6558670	H	3.9500650	-3.1565450	3.4116010
H	1.7214560	6.1666170	2.8608440	H	4.3180750	-1.4133010	-1.8798070
H	-1.9675540	1.4149060	-0.3698620	H	2.9643880	-0.8934800	-2.8832510
H	-1.7032300	2.7036150	0.7927440	H	3.2096550	1.4543820	-1.9410670
H	-2.6091290	4.3896930	-0.8166650	H	4.6481350	0.9364740	-1.0695840
H	-2.7473440	3.1656690	-2.0753900	H	4.3174820	0.9224250	-4.1096770
H	-4.3879130	1.9015530	-0.6975660	H	5.1877920	2.1254770	-3.1714130
H	-4.2509590	3.0973060	0.5851880	H	6.7842000	0.3215110	-2.3717380
H	-6.2785820	3.5283740	-0.8145390	H	5.9666690	-0.8394330	-3.4275420
H	-5.1376190	4.8715580	-0.9703910	H	6.8128100	0.5402220	-4.1276540
H	-5.2697680	3.6682550	-2.2611230	H	1.1809040	-2.7933780	-1.5368120
H	-0.5277830	2.9551180	-3.2442900	H	2.7485870	-3.3179240	-0.9473110
H	1.0494480	2.2221620	-2.9569660	H	1.9825260	-3.4791080	1.3387210
H	-0.1477620	0.0526990	-2.2718920	H	0.5825090	-2.4715440	1.0507750
H	-1.6748740	0.7993270	-2.7202020	H	-0.4197970	-4.2358150	-0.4017220
H	-0.8793120	1.0639660	-5.0554190	H	0.9695340	-5.2698870	-0.1102750
H	0.6896820	0.4019290	-4.6161750	H	0.4371790	-5.2809590	2.3621180
H	-0.3473880	-1.7888540	-4.0367960	H	-0.9652890	-4.2496370	2.0564850
H	-1.9598970	-1.1430940	-4.3781140	H	-0.9209290	-5.8928330	1.4051540
H	-0.7858870	-1.3374660	-5.6880550	H	-1.4532200	4.5388900	1.6732360
H	-2.3105460	-2.8294780	2.7347620	H	-1.2462050	4.5950960	-0.0795040
H	-0.6445870	-2.3361930	3.0289920	H	-3.3043070	3.1088110	-0.3221550
H	-1.4416170	0.0937300	3.1768810	H	-3.5805100	3.2617760	1.4106550
H	-3.1033500	-0.4804010	3.0477730	H	-3.5416340	5.5669590	-0.5994910
H	-1.1352930	-1.2134950	5.2709150	H	-4.9757790	4.8217630	0.0866570
H	-2.4248420	-0.0271170	5.3828680	H	-2.8661820	6.4129890	1.6620460
H	-3.1841720	-2.1155330	6.4388840	H	-4.5138440	6.9213620	1.2920330
H	-4.1267470	-1.8664540	4.9623000	H	-4.2355770	5.5678860	2.3975420
H	-2.8153950	-3.0550070	4.9944780	H	1.5431240	1.9304780	1.3051150
H	-1.5934210	-3.9871460	0.2613030	H	1.0832770	3.4907650	2.0042170
H	0.0807920	-3.5494180	0.5894560	H	1.5564250	2.9829940	-0.9918140
H	-1.4861350	-2.7127970	-1.9337780	H	1.2815990	4.5595030	-0.2557920
H	0.1783140	-2.2680960	-1.5815280	H	3.4078550	4.3023070	1.0566590
H	0.1809160	-4.0944250	-3.1441030	H	3.6635480	2.7294710	0.3178640
H	0.8370540	-4.6807920	-1.6259640	H	5.0139880	4.4541420	-0.8684560
H	-2.0600650	-5.1298280	-2.5582030	H	3.7758240	3.8157000	-1.9592240
H	-0.7581340	-6.3223550	-2.6781650	H	3.5549630	5.3984000	-1.2043130
H	-1.3388710	-5.8152320	-1.0939550	H	-2.3115680	1.2729820	2.0803000
H	-2.7522620	-0.9239570	-0.9070580	H	-1.2776110	2.2531930	3.1127110
H	-3.2185370	-0.2300980	0.6447130	H	-0.2217520	-0.2719230	1.7130000
H	-3.8378090	-3.1054200	-0.2692580	H	0.5408030	0.6263170	3.0077580
H	-4.2714900	-2.4414540	1.3047960	H	-1.4220540	0.0055380	4.5099130
H	-5.6536500	-0.6871720	0.1868580	H	-2.0469540	-0.9891870	3.2022190
H	-5.2173230	-1.3401660	-1.3864740	H	-0.7425540	-2.3955300	4.8005870
H	-6.7625450	-2.8686830	0.7907340	H	0.0892080	-2.3564190	3.2423830
H	-7.4641600	-2.1774470	-0.6788150	H	0.6788470	-1.3643260	4.5802770
H	-6.3239160	-3.5252600	-0.7929670	H	-0.2444070	0.5074260	-2.5631850
H	1.8099120	-0.6411060	-1.3356350	H	-2.4581620	0.7670720	-2.4793280
				H	-2.6818630	-0.4531150	0.7737980
**II-Pe$_2$ (dication)**				**II-Pe$_2$ (protonated)**			
C	3.1611530	0.9067680	3.7747670	C	2.2997650	-1.4238340	4.0308600
C	2.2532050	0.5690300	2.5899110	C	2.7208140	-1.8770880	2.6173420
C	3.2251520	0.5981430	1.3862680	C	1.5612340	-2.7837320	2.0923950
C	4.6406760	0.2638980	1.9563000	C	0.4416400	-2.6541200	3.1490360
C	4.4336000	0.1107940	3.4759880	C	1.2078540	-2.4099600	4.4504540
P	2.7268660	-0.4266410	-0.0289120	P	1.0476580	-2.3530110	0.3875890
C	3.6649290	0.1331130	-1.4830390	C	2.6025980	-2.2768240	-0.5797340
C	3.0933460	-0.3220610	-2.8624330	C	2.4018580	-2.0515650	-2.1075290
C	4.2971170	-0.9081750	-3.6233940	C	2.9204410	-3.3345660	-2.7688710
C	5.5193180	-0.2623210	-2.9665550	C	4.0620690	-3.7691920	-1.8480800
C	5.1596300	-0.2737730	-1.4776200	C	3.4865810	-3.5478690	-0.4431420
N	1.1249690	-0.2312190	-0.2684090	N	0.1842890	-1.0132220	0.3233480
P	-0.1154180	0.5537600	-0.6967370	P	0.3824940	0.5400580	0.6884100
N	-1.5598770	0.0265450	-0.6499110	N	-0.9650190	1.4004090	0.6281170
P	-2.3340310	-1.3043270	-0.0661280	P	-2.3261720	1.1858040	-0.1880900
C	-1.3681280	-2.8069740	-0.4073220	C	-2.1109170	1.4411520	-1.9898950
C	-1.2624880	-3.1121400	-1.9276030	C	-3.4347650	1.4466360	-2.8095090
C	-2.1735190	-4.3251020	-2.1429460	C	-3.1798770	0.5067340	-4.0012270
C	-1.9851660	-5.1395610	-0.8615090	C	-2.1540960	-0.4977440	-3.4712580
C	-1.9628120	-4.0842320	0.2568390	C	-1.1979850	0.3787350	-2.6574600
C	0.0090040	2.2089080	-1.3796990	C	1.6900020	1.2481350	-0.3843680
C	-1.2566600	3.0880220	-1.2454420	C	3.0981870	1.5103750	0.2077270
C	-1.5459530	3.4467490	0.2664820	C	3.8312930	1.8692910	-1.1086540
C	-0.5015600	2.8246600	1.2091770	C	2.8801770	2.9494030	-1.6525480
C	0.7790880	3.4980320	0.7138690	C	1.4568280	2.5798950	-1.1178020
C	1.0830480	3.1321060	-0.7749540	C	3.1142200	2.7666270	1.1673770
C	-0.7185070	4.3433650	-1.9748380	C	3.8697670	3.9204490	0.4606210
C	-1.6523920	5.5468590	-1.9037100	C	5.2884190	3.5777980	0.0187090

C	-1.9504840	5.9012230	-0.4320890
C	-1.2123570	4.9419290	0.4949720
C	0.6049840	4.5526520	-1.2202960
C	0.3090330	4.9177530	0.2652220
C	2.9927920	-2.1983780	0.2738370
C	2.2946950	-2.7629880	1.5242720
C	2.3459080	-4.2721750	1.2725220
C	2.0624090	-4.4249230	-0.2313490
C	2.4943100	-3.0906830	-0.8915750
C	-2.5260630	-1.1164020	1.7297440
C	-3.2363650	0.1780450	2.1705530
C	-2.8337820	0.2842590	3.6432490
C	-1.3489640	-0.1068790	3.6511190
C	-1.1738760	-1.1239580	2.4959900
C	-3.9391000	-1.3363050	-0.9131590
C	-5.0009980	-2.2806070	-0.3006970
C	-6.3192330	-1.6518390	-0.7615110
C	-6.0753290	-0.1528430	-0.5707750
C	-4.6159990	0.0694730	-1.0121450
H	0.2246870	2.0308880	-2.4412230
H	-0.7166900	3.0999830	2.2460010
H	-0.5227720	4.0922970	-3.0224170
H	-2.1303040	2.6489530	-1.7285610
H	1.3295580	5.1770840	-1.7433060
H	0.8424770	5.7794740	0.6672270
H	-1.4297790	5.1952160	1.5375650
H	-2.5755210	3.1961690	0.5311550
H	2.1101390	2.8545210	-1.0114540
H	-1.6408960	6.9263470	-0.2059740
H	-3.0240060	5.8451720	-0.2264720
H	-1.1830850	6.3879730	-2.4233640
H	-2.5740310	5.3175290	-2.4473080
H	1.6330140	-4.8181790	1.8941370
H	3.3479840	-4.6433110	1.5101430
H	2.5903590	-5.2776910	-0.6616310
H	0.9947520	-4.5945480	-0.4001940
H	3.2730810	-3.2254590	-1.6447710
H	1.6381580	-2.6280890	-1.3911030
H	1.2535030	-2.4241940	1.5662360
H	2.7852870	-2.4696400	2.4546730
H	4.2343640	-0.7170080	-4.6966730
H	2.6936780	0.5535760	-3.3786200
H	2.2741200	-1.0390650	-2.7819060
H	5.3006610	0.4572310	4.0420340
H	5.7674800	0.4015840	-0.8725100
H	4.2759180	-0.9433690	3.7283710
H	5.6359830	0.7706270	-3.3130500
H	5.0902270	-0.6327630	1.5225520
H	5.2975630	-1.2883770	-1.0888900
H	5.3159020	1.0920320	1.7317470
H	2.7051840	0.6473190	4.7326580
H	1.4303540	1.2750080	2.4631490
H	3.3775150	1.9810280	3.7820560
H	1.8188690	-0.4236490	2.7357790
H	-3.0096410	1.2819720	4.0511350
H	-3.4192430	-0.4278060	4.2344020
H	-2.8452170	1.0376590	1.6144660
H	-4.9634310	-2.2684570	0.7941940
H	-4.8772980	-3.3152520	-0.6278580
H	-6.7721980	0.4693030	-1.1364000
H	-6.4867950	-1.8790170	-1.8202590
H	-7.1765210	-2.0248790	-0.1968180
H	-6.1923820	0.1041120	0.4878660
H	-4.5600370	0.4116750	-2.0473430
H	-4.1180020	0.8312090	-0.4087160
H	-0.7320710	0.7777910	3.4665440
H	-0.3388980	-0.8278390	1.8542000
H	-1.0319850	-0.5235260	4.6088630
H	-1.5076410	-2.2612180	-2.5698630
H	-0.2285880	-3.3859450	-2.1571410
H	-1.9148880	-4.8796430	-3.0473710
H	-3.2188550	-4.0085950	-2.2392540
H	-2.7678830	-5.8849400	-0.7055750
H	-1.3674820	-4.3879900	1.1202480
H	-2.9815810	-3.9176520	0.6200050
H	-0.9494040	-2.1293960	2.8583400
H	-1.0268070	-5.6689830	-0.8988310
H	4.3379980	-1.9941330	-3.4842710
H	6.4507240	-0.7945580	-3.1717770
H	-4.3170580	0.1377760	2.0167600
H	-0.3792170	-2.5805960	0.0050230
H	-3.7036200	-1.6809540	-1.9259230
H	-3.1230190	-1.9746230	2.0574210
H	3.2375610	1.6149160	0.9767980
H	4.0726600	-2.3324560	0.3951700
H	3.6077810	1.2255650	-1.4236360
H	-0.4677800	1.7244560	1.2240030
C	5.2641970	2.3552900	-0.9215410
C	2.9091380	4.1903410	-0.7113230
C	1.4871090	3.8213780	-0.1765970
C	1.7242110	3.4203250	1.2790460
C	0.0314710	-3.7133530	-0.2925360
C	-1.1006200	-4.1969950	0.6365010
C	-2.0590440	-4.9007360	-0.3251520
C	-2.0914980	-3.9722860	-1.5436960
C	-0.6734610	-3.3617460	-1.6340630
C	-3.0288480	-0.4736740	0.1279450
C	-4.4679050	-0.7692520	-0.3427540
C	-4.8967000	-1.9260900	0.5655360
C	-4.3537430	-1.5148900	1.9367900
C	-2.9983300	-0.8424560	1.6432100
C	-3.4894990	2.4820590	0.3342520
C	-3.8429410	2.4903220	1.8317230
C	-4.3633950	3.9142870	2.0406300
C	-3.3719990	4.7755770	1.2463840
C	-2.9409120	3.9078270	0.0378180
H	0.6423730	2.5518300	-1.8419290
H	1.7927840	4.3081030	1.9164730
H	1.7905630	0.4726370	-1.1535270
H	3.8935450	4.7856870	1.1322050
H	3.5511130	2.5061130	2.1346750
H	3.0441440	5.1609030	-1.1922900
H	2.9847160	3.1442410	-2.7214300
H	3.8288840	0.9933740	-1.7675330
H	3.5414590	0.6401850	0.7008560
H	0.6812940	4.5410520	-0.3295200
H	5.6860260	2.6041590	-1.9009770
H	5.8715970	1.5378250	-0.5189470
H	5.7305560	4.4465610	-0.4803140
H	5.9036760	3.3786140	0.9024610
H	-3.0470000	-5.0664460	0.1129000
H	-1.6490540	-5.8782020	-0.6033860
H	-2.3773130	-4.4891970	-2.4623730
H	-2.8289100	-3.1780160	-1.3811660
H	-0.1123750	-3.7694500	-2.4776460
H	-0.7340810	-2.2816590	-1.7758170
H	-1.6074290	-3.3433810	1.0995040
H	-0.7359440	-4.8483390	1.4336220
H	3.2325420	-3.1731280	-3.8033410
H	3.0195270	-1.2038600	-2.4205490
H	1.3743960	-1.8093020	-2.3879420
H	1.6556200	-3.3479600	4.7988290
H	4.2541180	-3.4235700	0.3237790
H	0.5680110	-2.0268470	5.2493430
H	4.9310730	-3.1197390	-2.0035670
H	-0.1883670	-1.7853830	2.9252080
H	2.8913590	-4.4230830	-0.1592250
H	-0.2035450	-3.5327150	3.1906170
H	1.8794490	-0.4126290	3.9873300
H	3.6523850	-2.4470580	2.6372320
H	3.1444730	-1.3959070	4.7228660
H	2.9047250	-1.0073770	1.9805170
H	-5.9773250	-2.0885550	0.5591690
H	-4.4184070	-2.8554950	0.2326810
H	-5.1276610	0.0891930	-0.1690560
H	-2.9382920	2.3355340	2.4309630
H	-4.5690470	1.7172140	2.0975170
H	-3.8012290	5.7310120	0.9357380
H	-5.3707060	4.0025440	1.6173600
H	-4.4196410	4.1936830	3.0958090
H	-2.4995570	4.9969400	1.8692130
H	-3.3480110	4.2807390	-0.9050900
H	-1.8507760	3.9030100	-0.0555150
H	-5.0381520	-0.7932140	2.3968380
H	-2.8304710	0.0219470	2.2881290
H	-4.2560700	-2.3570410	2.6262530
H	-4.2773040	1.0722660	-2.2222840
H	-3.6992260	2.4592750	-3.1214910
H	-2.7428550	1.0684250	-4.8340980
H	-4.0979590	0.0368560	-4.3626970
H	-2.6472850	-1.2291950	-2.8187280
H	-0.4977990	0.8839360	-3.3304060
H	-0.5972620	-0.1901660	-1.9425420
H	-2.1599670	-1.5174970	1.8226880
H	-1.6437900	-1.0562300	-4.2599770
H	2.1384940	-4.1036720	-2.7741500
H	4.3815790	-4.8014900	-2.0098230
H	-4.5201110	-1.0197050	-1.4053550
H	-1.6342030	2.4255180	-2.0370090
H	-4.4097850	2.3262020	-0.2397950
H	-2.3354160	-1.1449810	-0.3920010
H	1.9047990	-3.8238160	2.0587530
H	0.7181480	-4.5520210	-0.4544100
H	3.1395790	-1.4198640	-0.1628890

H	1.6282230	3.4374270	1.3959000	H	0.8749540	0.6674350	2.0037870
				H	0.9297640	2.8021130	1.6968950
**II-Pf$_2$ (dication)**				**II-Pf$_2$ (protonated)**			
C	2.3870140	-0.3545670	-1.6075240	C	-3.1110330	-0.1570660	1.9136350
C	3.3297400	0.5935710	-0.8482430	C	-3.6727090	0.2043580	0.5293810
C	4.6672740	-0.1485990	-1.0903140	C	-4.9336240	-0.6950690	0.5317700
C	4.2706890	-1.5692290	-0.6525610	C	-4.2984920	-2.0427250	0.9185700
C	2.7472890	-1.6973140	-0.9709460	C	-3.0621890	-1.6844440	1.8069720
C	3.9579630	-1.5746930	0.8740170	C	-3.3482280	-2.5126590	-0.2231940
C	4.1518290	-0.1600570	1.4462610	C	-3.3536580	-1.4728210	-1.3576030
C	5.5451380	0.4075370	1.1973760	C	-4.7431660	-1.1941130	-1.9208940
C	5.8520610	0.4166340	-0.3145830	C	-5.6900630	-0.7391530	-0.7914770
C	3.0234790	0.5959910	0.7018170	C	-2.7063760	-0.2710040	-0.6233350
C	1.8650010	-0.3685930	1.0448860	C	-1.4493320	-0.9974720	-0.0857200
C	2.4331970	-1.7124680	0.5593830	C	-2.1094610	-2.1662600	0.6661130
P	0.2301020	0.0818430	0.4573400	P	-0.1014680	-0.0655480	0.7300260
N	-0.8055980	-1.0124970	0.2649810	N	-0.4041290	1.5008720	0.6296660
P	-1.7452130	-2.3255730	0.1263250	P	0.4426330	2.7639030	0.1337600
C	-0.8952910	-3.4998710	-0.9964770	C	1.3123640	2.4369030	-1.4574800
C	-1.6897110	-4.7950510	-1.2024990	C	0.4383360	1.7336440	-2.5012150
N	0.0287660	1.5989880	0.3323490	N	1.3214570	-0.4971380	0.1316530
P	-1.0863250	2.7346160	-0.0732270	P	2.2710290	-1.7702900	-0.0004900
C	-0.6562650	4.1701690	0.9777900	C	2.5793660	-2.6243700	1.6021360
C	-0.6752860	3.8378830	2.4746480	C	1.3478340	-3.3833390	2.1108190
C	-0.8155170	3.1762540	-1.8316040	C	1.5493810	-3.0261400	-1.1416080
C	0.6138710	3.6868660	-2.0536740	C	1.1227920	-2.4375210	-2.4940760
C	-2.7865960	2.0669070	0.1095240	C	3.8937350	-1.1153540	-0.5702620
C	-3.8664570	2.9765800	-0.4889300	C	3.8306960	-0.5859690	-2.0083420
C	-1.1501690	2.0008950	-2.7581370	C	2.4075130	-4.2826200	-1.3223470
C	-3.3932330	-1.7618680	-0.4459670	C	1.7391180	3.2937120	1.3338740
C	-4.5127170	-2.7911330	-0.2484030	C	2.9433640	2.3446300	1.3553770
C	-1.9276670	-3.0805270	1.7859860	C	-0.7761370	4.1388010	0.0377100
C	-0.5944020	-3.6123930	2.3217220	C	-0.1791280	5.5499520	0.0176780
C	-3.3549250	-1.2251620	-1.8821820	C	1.1161960	3.4286360	2.7290290
C	-2.5638810	-2.0847360	2.7651100	C	-1.7717320	3.9262820	-1.1084970
C	-0.4698140	-2.8706760	-2.3289190	C	2.0214300	3.6625580	-2.0410630
C	-3.1205260	1.6540520	1.5475850	C	5.0864390	-2.0569990	-0.3714430
C	-1.4573510	5.4343800	0.6483430	C	3.0724410	-1.6114480	2.6438520
H	1.9154120	-2.5918080	0.9415240	H	-1.4379830	-2.9878140	0.9245030
H	2.6190310	-0.3482390	-2.6758970	H	-3.8230870	0.1436180	2.6895010
H	1.7254750	-0.3933690	2.1341090	H	-0.9322280	-1.4114480	-0.9586470
H	4.8873580	-0.1345710	-2.1627250	H	-5.6057110	-0.3555440	1.3276770
H	3.3332980	1.6120940	-1.2418520	H	-3.9082330	1.2661660	0.4216940
H	4.9332300	-2.3566100	-1.0134340	H	-5.0047730	-2.7843860	1.2964580
H	4.4167080	-2.3673160	1.4657030	H	-3.4583340	-3.5460170	-0.5572250
H	3.9393220	-0.1526200	2.5204600	H	-2.6908930	-1.7946230	-2.1689320
H	2.8595250	1.6058990	1.0797220	H	-2.4595250	0.5513470	-1.2987940
H	2.4143100	-2.5755240	-1.5243000	H	-2.9509870	-2.2079240	2.7577200
H	6.2749750	-0.1987860	1.7429640	H	-5.1194410	-2.1000240	-2.4078360
H	5.6012180	1.4171370	1.6160960	H	-4.6670960	-0.4293680	-2.7010820
H	6.7425690	-0.1798080	-0.5365400	H	-6.5427110	-1.4201270	-0.6974060
H	6.0599990	1.4325940	-0.6646860	H	-6.1042100	0.2520230	-1.0043080
H	-0.1615440	-4.3886470	1.6869750	H	0.4114610	5.7653010	0.9111150
H	0.1330270	-2.8049150	2.4449170	H	0.4499460	5.7201110	-0.8593570
H	-0.7652800	-4.0530110	3.3066060	H	-0.9956850	6.2766980	-0.0174200
H	-2.6123100	-3.9235850	1.6370360	H	-1.3221090	4.0069200	0.9799820
H	-2.7014140	-2.5797030	3.7289340	H	-1.3186200	4.1630860	-2.0754150
H	-3.5424350	-1.7282080	2.4347760	H	-2.6266720	4.5941870	-0.9725350
H	-1.9120570	-1.2209560	2.9234490	H	-2.1443470	2.8989770	-1.1360550
H	0.0169680	-3.7315160	-0.4329000	H	2.0666130	1.7095690	-1.1355370
H	-2.5764350	-4.6227630	-1.8176700	H	1.0794260	1.3525590	-3.3022640
H	-1.0556800	-5.5095050	-1.7325990	H	-0.1039090	0.8863190	-2.0758470
H	-1.9982600	-5.2613230	-0.2634800	H	-0.2880680	2.4123770	-2.9532570
H	0.0108690	-1.8992340	-2.1972210	H	2.6472970	4.1785950	-1.3077210
H	0.2505360	-3.5366830	-2.8099860	H	2.6701270	3.3443050	-2.8620730
H	-1.3147970	-2.7488140	-3.0079620	H	1.3041290	4.3804300	-2.4491650
H	-3.5811340	-0.9193600	0.2312220	H	2.0813820	4.2800250	1.0004760
H	-4.6220250	-3.0984560	0.7936760	H	2.6353330	1.3143110	1.5551880
H	-5.4561070	-2.3347550	-0.5578210	H	3.4966390	2.3577490	0.4124620
H	-4.3615920	-3.6836230	-0.8591490	H	3.6312160	2.6560220	2.1464270
H	-4.2349750	-0.5994840	-2.0506410	H	0.2900200	4.1448330	2.7516310
H	-2.4663300	-0.6204570	-2.0840160	H	0.7379860	2.4629450	3.0775290
H	-1.5200970	3.9914200	-2.0331560	H	0.6337820	-3.3097360	-0.6048230
H	-3.9684460	3.9074880	0.0726750	H	4.6826410	0.0758170	-2.1852570
H	-3.6808760	3.2208600	-1.5370990	H	3.8911600	-1.4036290	-2.7314490
H	-4.8237440	2.4519860	-0.4379870	H	2.9157230	-0.0206180	-2.2017370
H	-4.0235350	1.0375500	1.5352040	H	4.9839460	-2.9786680	-0.9495730
H	-0.5087020	1.1362880	-2.5627200	H	1.8342910	-5.0313520	-1.8758270
H	-0.9749790	2.3083990	-3.7914120	H	2.7091690	-4.7323060	-0.3727760
H	-2.1946140	1.6894150	-2.6830200	H	3.3078040	-4.0649300	-1.9033780
H	0.7467940	3.9052480	-3.1156790	H	1.9674590	-2.3727330	-3.1830450
H	1.3528390	2.9335830	-1.7670790	H	0.6923440	-1.4378380	-2.3962960
H	0.3900480	4.3318260	0.6906840	H	3.3761420	-3.3519970	1.4107180
H	-1.0196450	6.2719250	1.1968600	H	3.3003260	-2.1318540	3.5777410
H	-1.4317930	5.6887150	-0.4142310	H	3.9780430	-1.0880110	2.3253670
H	-2.4998050	5.3452660	0.9624450	H	2.3037420	-0.8618100	2.8541830

H	-0.1618870	2.8986060	2.6932500	H	1.5887270	-3.8550210	3.0672370
H	-1.6936910	3.7847500	2.8638440	H	1.0275700	-4.1733980	1.4270100
H	-0.1570590	4.6364850	3.0109070	H	0.5021180	-2.7109820	2.2850600
H	-2.3235520	1.0740840	2.0195310	H	5.9931120	-1.5522020	-0.7164380
H	-3.3313290	2.5237710	2.1727190	H	5.2395850	-2.3241030	0.6766300
H	-2.7295070	1.1571810	-0.5013860	H	4.0170120	-0.2530630	0.0967050
H	0.8191930	4.6056300	-1.5000160	H	0.3721770	-3.0896650	-2.9487370
H	-3.3844670	-2.0420510	-2.6071060	H	1.8757250	3.7739890	3.4354970
H	1.3183410	-0.0959330	-1.5612980	H	-0.1624550	-0.4371290	2.0919570
				H	-2.1653770	0.3253500	2.1609210
**II-Pg$_2$ (dication)**				**II-Pg$_2$ (protonated)**			
C	-4.3047510	-1.2346430	0.8376340	C	-3.6454210	-3.0017590	1.7963110
C	-4.6428680	0.2489760	1.0682200	C	-2.4443780	-2.7748380	0.8841040
C	-5.7634310	0.7631070	0.1711680	C	-2.0325330	-1.2893160	0.7233950
C	-5.4007710	0.5303070	-1.3100920	C	-3.2747470	-0.4695370	0.2004260
C	-4.0279140	-0.1248650	-1.4170640	C	-4.4758320	-1.4309710	0.0233890
C	-3.9265690	-1.4582880	-0.6572320	C	-4.8615920	-2.2069800	1.2778510
C	-2.8947860	0.6733550	-0.7249670	C	-3.0827300	-0.0463660	-1.2632310
C	-3.2581810	0.8885020	0.7981960	C	-2.9801210	-1.4139740	-1.9442560
C	-2.8064870	-1.3887760	1.2452970	C	-1.7551930	-2.2355330	-1.4317920
C	-2.3989880	0.0050860	1.7201360	C	-0.9461290	-1.4841100	-0.3607040
C	-2.4240250	-1.6200330	-0.2514370	C	-3.9365760	-2.3250660	-1.1074990
C	-1.7744840	-0.3760590	-0.8896050	C	-2.7134240	-3.1382350	-0.5872850
P	-0.1332830	0.0989520	-0.3403030	P	0.1220060	-0.0994380	-0.8916180
N	0.9524380	-0.9561830	-0.1600510	N	1.6218710	-0.6306630	-1.1408140
P	1.5678140	-2.4497720	-0.0404660	P	2.6326540	-1.2992290	-0.1130810
N	3.0807630	-2.3250160	-0.6543860	N	2.5406520	-0.9571010	1.5149070
C	3.9069590	-1.1277080	-0.5222070	C	2.9973440	0.3386600	2.0142410
N	0.0686740	1.6152950	-0.2029970	N	-0.0405970	1.0913050	0.1707180
P	1.2783260	2.6976610	-0.0241410	P	-0.2267100	2.6591430	0.0762510
N	1.8562760	3.1691560	-1.4918080	N	1.0754150	3.3767960	-0.6888820
C	2.3374910	2.1336530	-2.4095930	C	1.0985870	4.8084590	-0.9838900
N	0.5915700	3.9307820	0.8032500	N	-1.5076970	3.2759430	-0.7844480
C	-0.8151730	4.3086470	0.6639820	C	-2.8410230	3.3876090	-0.2044440
N	2.5653180	2.0579940	0.7749590	N	-0.4937020	3.1714250	1.6311240
C	3.9473850	2.4961690	0.5647970	C	-0.5311840	4.5963830	1.9338140
C	1.4253380	4.9162930	1.4905340	C	-1.5013650	3.3241470	-2.2436760
N	1.5730350	-2.9782610	1.5151320	N	2.4205480	-2.9549330	-0.1369590
C	0.3085440	-3.0449400	2.2482580	C	1.9379410	-3.6211270	-1.3424940
N	0.6605800	-3.5264150	-0.8817250	N	4.1491290	-0.7606990	-0.5316260
C	0.4901240	-4.9305050	-0.5057140	C	4.4295300	-0.2161110	-1.8548470
C	2.7504820	-2.8061680	2.3654070	C	3.1816990	-3.8405130	0.7401460
C	0.2844100	-3.2240830	-2.2628900	C	5.3219190	-1.3092240	0.1418040
C	3.7923950	-3.5172730	-1.1131850	C	1.4508410	-1.5056390	2.3198420
C	2.3480020	1.3798330	2.0537210	C	-0.3676480	2.3077020	2.7950190
C	1.2978210	4.3473490	-2.1573400	C	2.4145040	2.8276550	-0.4911980
H	-1.9261370	-2.5607390	-0.4883010	H	-3.1114650	-1.3974850	-3.0269960
H	-2.6864740	0.1631610	2.7632210	H	-0.2121230	-2.1919080	0.0438420
H	-1.6101060	-0.5655370	-1.9595990	H	-3.9727690	0.4853360	-1.6172440
H	-4.9062710	0.4131540	2.1182000	H	-1.5880830	-3.3435710	1.2642910
H	-3.2206340	1.9486490	1.0569450	H	-1.6383130	-0.8576510	1.6456300
H	-5.0233390	-1.9352570	1.2643460	H	-2.7146590	-4.2098500	-0.7948580
H	-4.3978420	-2.3021050	-1.1620450	H	-4.7024860	-2.8909560	-1.6410180
H	-3.7721700	-0.2673940	-2.4723290	H	-5.3373490	-0.8550930	-0.3324910
H	-2.6699990	1.6163890	-1.2255350	H	-3.4939480	0.3679580	0.8679070
H	-2.5493230	-2.2009070	1.9248460	H	-1.1510770	-2.7207460	-2.2005080
H	-6.1376770	-0.1146600	-1.7990290	H	-5.6965700	-2.8764720	1.0454130
H	-5.3922280	1.4731150	-1.8658180	H	-5.2244820	-1.5075650	2.0383220
H	-6.6949630	0.2504310	0.4309150	H	-3.8654750	-4.0737770	1.8368830
H	-5.9250720	1.8271410	0.3708730	H	-3.3839800	-2.6982180	2.8155180
H	4.2912540	3.1055140	1.4079040	H	3.4880760	-3.3156930	1.6450370
H	3.1137170	2.5580010	-3.0504380	H	5.0737060	-1.6080000	1.1611840
H	4.5956120	1.6186560	0.4857740	H	2.5487910	-4.6849940	1.0315930
H	2.7748390	1.3028710	-1.8534400	H	5.7319540	-2.1750680	-0.3937740
H	1.5242850	1.7516530	-3.0385040	H	6.0960460	-0.5376060	0.1961280
H	3.0140200	0.5151430	2.1164970	H	1.3921120	-2.9163560	-1.9680580
H	2.5589310	2.0513710	2.8928140	H	2.7667480	-4.0438010	-1.9246930
H	1.3205410	1.0261330	2.1432660	H	1.2630450	-4.4354460	-1.0580520
H	2.0522880	4.7486120	-2.8376980	H	3.5235070	0.2159510	-2.2759130
H	0.3978780	4.0989350	-2.7320300	H	5.1878270	0.5681730	-1.7617860
H	1.0553900	5.1192300	-1.4275280	H	4.8109270	-0.9875870	-2.5365840
H	-0.9128310	5.2403410	0.0961250	H	0.5830580	-0.8336250	2.3188880
H	-1.2435530	4.4546140	1.6594730	H	1.7948000	-1.6252570	3.3513390
H	-1.3686640	3.5203630	0.1561610	H	1.1431210	-2.4812860	1.9443640
H	2.4344590	4.5325120	1.6411200	H	3.8222040	0.7097520	1.4070560
H	0.9893150	5.1277250	2.4703400	H	3.3450550	0.2184000	3.0445910
H	3.6616910	-2.8315780	1.7691200	H	0.0881680	5.1924360	-1.1218030
H	2.7874280	-3.6330630	3.0785640	H	1.6565890	4.9720440	-1.9111500
H	2.7095120	-1.8627870	2.9218570	H	1.5862230	5.3773300	-0.1818250
H	-0.5152720	-3.2576020	1.5651410	H	2.3790650	1.7414640	-0.4352670
H	0.1051910	-2.1040290	2.7739140	H	2.8884680	3.2217040	0.4180380
H	0.3617420	-3.8562300	2.9779880	H	3.0357970	3.1021670	-1.3489500
H	-0.7772200	-3.4425080	-2.4123150	H	-3.5061340	2.6073290	-0.5922820
H	0.4602240	-2.1731450	-2.4960430	H	-2.7914260	3.2966770	0.8799800
H	0.8713680	-3.8298360	-2.9610630	H	-3.2684390	4.3639830	-0.4571050
H	-0.5755330	-5.1773610	-0.4919120	H	-2.1545070	2.5494020	-2.6642890

H	4.2903550	-3.2918750	-2.0598490	H	-1.2961920	4.7849700	2.6939390
H	0.9119320	-5.1138900	0.4811780	H	-0.4919330	3.1788610	-2.6279880
H	4.5447710	-3.8355040	-0.3833950	H	0.4333150	4.9558970	2.3136130
H	0.9923380	-5.5803010	-1.2306920	H	-1.8660000	4.2998480	-2.5831720
H	3.2946850	-0.2869220	-0.1953630	H	0.5777990	2.4827440	3.3247970
H	4.3487410	-0.8867070	-1.4935390	H	-0.4125250	1.2656530	2.4843870
H	4.7146970	-1.2849420	0.2017210	H	-1.1932730	2.5095420	3.4863630
H	3.0965330	-4.3399140	-1.2796840	H	-0.7973440	5.1733720	1.0468870
H	1.4874700	5.8501920	0.9213640	H	2.1837520	1.0743710	1.9979800
H	4.0259740	3.0802440	-0.3504060	H	4.0745330	-4.2344450	0.2384430
H	-1.3184610	0.1989380	1.6977810	H	-0.2990830	0.2858070	-2.1761580
				H	-2.2384450	0.6217160	-1.4320190

II-Ph$_2$ (dication)

C	3.7601160	-3.6586020	0.0405880
N	3.1515580	-2.3282880	0.2308080
C	4.1639750	-1.2997690	0.5589340
C	5.4889620	-2.0485790	0.3922550
C	5.1273200	-3.5030560	0.7029390
P	1.5522540	-2.0741950	0.0364410
N	1.4078870	-0.4783620	0.3349890
P	0.4038690	0.6814400	0.3721550
C	1.1296940	2.2184440	0.9448230
C	2.6488980	2.3871980	0.7283690
C	2.9913160	2.5310280	-0.8080200
C	1.7265910	2.4579920	-1.6809680
C	0.9501320	3.6711620	-1.1695990
C	0.5873000	3.5258280	0.3426860
C	1.7032870	4.5588080	0.7079030
C	2.8097350	3.7628580	1.4210730
C	4.1962640	4.3775710	1.2633930
C	4.5494610	4.5115610	-0.2324740
C	3.3958960	3.9994370	-1.0880920
C	2.0592980	4.7067220	-0.8017780
N	0.9216780	-2.3637660	-1.4567110
C	0.7820000	-3.7384260	-1.9990480
C	1.4374410	-3.6550220	-3.3735360
C	1.0676630	-2.2419390	-3.8262870
C	1.2234660	-1.4067670	-2.5526890
N	0.7672920	-3.1017390	1.0339280
C	1.2411240	-3.3171100	2.4214860
C	-0.0385890	-3.2256580	3.2472440
C	-1.0768980	-3.8687180	2.3254340
C	-0.6856450	-3.3741410	0.9269160
N	-1.0849640	0.7143640	0.0654970
P	-2.6673600	0.3809350	0.0739950
N	-3.3316980	1.5046890	-0.8976680
C	-2.7285280	2.7820000	-1.3199120
C	-3.7096500	3.2815840	-2.3799790
C	-5.0616540	2.7737580	-1.8723770
C	-4.7354670	1.3757820	-1.3419940
N	-3.3133970	0.4690560	1.5748680
C	-2.9737440	-0.5102160	2.6342840
C	-2.8250720	0.3471950	3.8911300
C	-3.8068260	1.4936130	3.6399560
C	-3.6020280	1.8019280	2.1577060
N	-2.9623260	-1.1442530	-0.4384170
C	-4.1502720	-1.9440990	-0.0558110
C	-4.5380480	-2.6468710	-1.3546780
C	-3.1859610	-2.8761280	-2.0325300
C	-2.4229280	-1.5813860	-1.7478470
H	0.1378360	4.0061250	-1.8148400
H	0.9129320	2.1949190	2.0216940
H	1.9904770	2.5743340	-2.7357650
H	2.5752810	3.6558810	2.4855240
H	3.2360430	1.5986820	1.2012360
H	1.3925900	5.4650200	1.2290090
H	1.9788170	5.7099810	-1.2219820
H	3.6513520	4.0973550	-2.1483330
H	3.7615140	1.8144080	-1.1020160
H	-0.4397190	3.7576360	0.6221970
H	4.7544820	5.5545170	-0.4940510
H	5.4547820	3.9447580	-0.4721560
H	4.2081670	5.3534640	1.7590960
H	4.9276100	3.7525010	1.7853460
H	-1.8033440	0.7347360	3.9597110
H	-3.0410420	-0.2177800	4.7992730
H	-4.8338550	1.1583540	3.8142450
H	-3.6191900	2.3679450	4.2649880
H	-4.4798950	2.2448480	1.6825560
H	-2.7500530	2.4805150	2.0236670
H	-2.0590840	-1.0583790	2.3914520
H	-3.7886300	-1.2338130	2.7377940
H	-3.4738310	2.8276340	-3.3474870
H	-1.7236600	2.6187720	-1.7121100
H	-2.6692120	3.4814310	-0.4774840
H	-2.6748800	-3.7238350	-1.5639190

II-Ph$_2$ (protonated)

C	4.1438140	2.2268570	0.8250500
N	2.8156940	1.5809300	0.9181150
C	2.5049250	1.2414990	2.3248460
C	3.8198710	1.4924400	3.0667310
C	4.4629500	2.6246110	2.2639590
P	1.5398280	1.9631370	-0.0728920
N	0.8755900	3.4455660	0.2129450
C	-0.5390140	3.6927980	0.5257850
C	-0.7540760	5.1157280	0.0175010
C	0.5830990	5.7896890	0.3377920
C	1.6170120	4.7018520	0.0175870
N	0.3450590	0.9490460	0.0959010
P	0.0662300	-0.6156860	0.2732000
C	1.2831480	-1.6257910	-0.6298100
C	2.5471420	-2.0756180	0.1405140
C	2.2210520	-3.2513620	1.1493480
C	0.7335800	-3.6595450	1.1112160
C	0.6031430	-4.1093400	-0.3422420
C	0.8979810	-2.9400390	-1.3287200
C	2.2880520	-3.5798730	-1.6656630
C	3.3487450	-2.6470810	-1.0555530
C	1.9912050	-4.7443250	-0.6746780
C	2.8387830	-4.5621690	0.5978950
C	4.6403700	-3.3588240	-0.6692740
C	4.3378840	-4.4957410	0.3278970
N	-1.3990850	-1.0869930	-0.1758240
P	-2.7958940	-0.4098050	0.1841380
N	-2.9654420	1.0664500	-0.5383850
C	-3.9842390	2.0707780	-0.1801500
C	-4.3558800	2.7010520	-1.5218930
C	-3.0432000	2.6320960	-2.3058300
C	-2.4692550	1.2692370	-1.9126940
N	-3.1624570	-0.0516310	1.7690210
C	-2.3858570	0.9781720	2.4864690
C	-1.6798110	0.2440330	3.6557740
C	-2.1424450	-1.2226420	3.5538270
C	-3.4243120	-1.1381540	2.7217570
N	-3.9266140	-1.4992790	-0.3062290
C	-3.6535450	-2.8761830	-0.7484360
C	-4.9926700	-3.3166420	-1.3424230
C	-6.0188770	-2.5590340	-0.4956130
C	-5.3633110	-1.1898550	-0.3019220
N	2.2782080	2.1176420	-1.5557940
C	3.2882560	1.1552700	-2.0389740
C	2.7578740	0.6295690	-3.3961900
C	1.3368230	1.2003320	-3.5135500
C	1.4213810	2.4869480	-2.6947380
H	-0.2927710	-4.6908750	-0.5645330
H	1.5971000	-0.9293020	-1.4162230
H	0.5739450	-4.5007740	1.7936840
H	3.5819970	-1.8339730	-1.7514650
H	3.0670280	-1.2502790	0.6375740
H	2.4782530	-3.8586860	-2.7038010
H	2.0120100	-5.7534760	-1.0903470
H	2.6293610	-5.3721800	1.3052260
H	2.5777600	-3.0068030	2.1531080
H	0.1856350	-2.8109620	-2.1436860
H	4.6773410	-5.4604840	-0.0636210
H	4.8681920	-4.3381130	1.2730070
H	5.1176670	-3.7469290	-1.5752590
H	5.3356880	-2.6313970	-0.2371990
H	-5.1211710	-4.4002710	-1.3073790
H	-5.0594600	-2.9969180	-2.3875560
H	-7.0008240	-2.4843010	-0.9665870
H	-6.1404850	-3.0511440	0.4750560
H	-5.6135000	-0.5082340	-1.1259570
H	-5.6625410	-0.7135940	0.6374910
H	-3.3717640	-3.5107410	0.1021520
H	-2.8356460	-2.8989920	-1.4703370
H	-1.9793760	0.6819280	4.6108890
H	-3.0690560	1.7465630	2.8629360
H	-1.6803940	1.4606110	1.8071620
H	-3.1763020	2.7248060	-3.3852890

H	-4.8114390	0.6183680	-2.1310070	H	-4.2783060	-0.8565630	3.3495930
H	-3.2620730	-3.0742100	-3.1027010	H	-2.3704870	3.4324200	-1.9787610
H	-5.8315390	2.7469490	-2.6449980	H	-2.3055700	-1.6836090	4.5302650
H	-2.6253280	-0.8256840	-2.5168780	H	-1.3766280	1.2495810	-1.9240210
H	-5.3888960	1.0815790	-0.5149940	H	-3.6789340	-2.0631440	2.2011500
H	-1.3438820	-1.7360580	-1.6797400	H	-2.8398690	0.4815470	-2.5817840
H	-5.1627060	-1.9876590	-1.9661700	H	-4.7404430	3.7167660	-1.4097160
H	-3.8667990	-2.6727570	0.7123010	H	-4.8363240	1.6173220	0.3301310
H	-5.0897860	-3.5700810	-1.1702700	H	-5.1219980	2.0953410	-2.0179450
H	-4.9418630	-1.3112550	0.3495400	H	-3.5507230	2.8187970	0.4967210
H	0.0524440	-3.7325560	4.2092440	H	3.3848600	1.0022310	-4.2101190
H	-0.2830120	-2.1741350	3.4341680	H	2.7723380	-0.4623440	-3.4484300
H	1.6934200	-4.3116730	2.5017990	H	4.2492650	1.6636630	-2.1630400
H	2.2437440	-1.0164580	-2.4596160	H	-0.7141930	3.6397370	1.6088330
H	0.5256280	-0.5643840	-2.5284050	H	-1.1731920	2.9460430	0.0449780
H	1.0767850	-4.4324160	-4.0491270	H	0.7597280	6.7016430	-0.2354690
H	0.0264970	-2.2170680	-4.1635900	H	-0.9207430	5.1041910	-1.0657110
H	1.6962190	-1.8645670	-4.6340520	H	-1.6083830	5.6032600	0.4917650
H	2.5239580	-3.7536050	-3.2786390	H	0.6246460	6.0437170	1.4017390
H	-0.2828260	-3.9803010	-2.0933760	H	1.9732060	4.7791310	-1.0165310
H	1.2392630	-4.4773310	-1.3379980	H	2.4870380	4.7607070	0.6801560
H	-0.9941030	-4.9582130	2.3736220	H	1.0323060	1.3735820	-4.5476790
H	-0.8798700	-4.1260830	0.1584810	H	1.9046730	3.2870490	-3.2681640
H	-2.1048410	-3.5997850	2.5773740	H	0.6079020	0.5246490	-3.0517610
H	4.0830920	-0.4390760	-0.1073680	H	1.7078170	1.8882600	2.7146000
H	4.0246320	-0.9530590	1.5883530	H	2.1738280	0.2022130	2.4131390
H	6.2636750	-1.6480830	1.0482010	H	4.4501990	0.5981540	3.0228870
H	5.8377970	-1.9629810	-0.6414970	H	3.6538470	1.7408030	4.1165060
H	5.8542110	-4.2203380	0.3185760	H	3.9819760	3.5792020	2.5046150
H	3.1488330	-4.4385510	0.5022410	H	4.8761090	1.5014900	0.4505350
H	3.8634920	-3.8839600	-1.0282870	H	4.1285720	3.0714480	0.1329650
H	-1.2318950	-2.4644690	0.6581380	H	0.4526180	2.8577750	-2.3514830
H	5.0324490	-3.6497950	1.7835170	H	5.5357840	2.7252320	2.4395790
H	-3.6718200	4.3665590	-2.4897990	H	-0.5925170	0.3320520	3.5837890
H	-5.4174580	3.4067840	-1.0534810	H	-1.4022100	-1.8264030	3.0183750
H	1.9915160	-2.5765490	2.7033700	H	3.4366770	0.3608260	-1.3031470
H	1.1715890	1.5104030	-1.6414990	H	0.2663700	-0.8984840	1.6425300
				H	0.0093460	-2.8950350	1.3913420
**III-Pa$_2$ (dication)**				**III-Pa$_2$ (protonated)**			
C	0.0611870	0.0079730	-1.3873690	C	-0.1336410	-0.7655430	0.0927870
C	0.9527540	1.2505030	-1.1722330	C	0.8635320	-1.4213660	-0.8765620
C	2.1564780	0.8611400	-2.0609650	C	1.7089390	-2.2411500	0.1168130
C	2.1663830	-0.6950610	-2.1910290	C	1.5816530	-1.5431060	1.5101890
C	0.9667100	-1.2362940	-1.3893530	C	0.6797380	-0.2976890	1.3258420
C	1.5333220	1.6862730	0.2112910	C	1.8618940	-0.6000610	-1.7545090
C	3.0990200	1.6913200	0.0911200	C	3.3102350	-1.1052080	-1.4062320
C	3.4698970	1.2614410	-1.3623790	C	3.1929960	-2.1856740	-0.2822030
C	1.1648890	1.0403340	1.5811370	C	1.9130220	0.9612460	-1.8630520
C	2.4776300	0.5244350	2.2041180	C	3.3483280	1.3857260	-1.4819660
C	3.6794440	1.0350540	1.3853930	C	4.2461040	0.1415580	-1.3306910
C	0.2836580	-0.2013150	1.6687630	C	1.0642630	1.8976610	-0.9911400
C	1.1507580	-1.4250720	1.3689620	C	1.7880170	2.0469120	0.3394030
C	2.4663840	-1.0330700	2.0755420	C	3.2687840	2.0634820	-0.0777770
C	3.6700230	-1.4190250	1.1926130	C	4.0937050	1.1936480	0.8901220
C	3.0967770	-1.8556830	-0.1926260	C	3.0661690	0.4324190	1.7917270
C	1.5305170	-1.8681360	-0.0801930	C	1.6260380	0.9476680	1.4280980
C	3.4781710	-1.1928220	-1.5558630	C	3.0007750	-1.1194170	1.9293470
C	4.5899220	-0.2000190	1.3430800	C	5.0526780	0.4699150	-0.0667990
C	4.3713400	0.0525450	-1.6466810	C	3.8850070	-2.0447990	1.0818850
P	-1.5424300	-0.1312470	-0.6091200	P	-1.6194470	0.0402610	-0.6042380
N	-2.3914090	1.1075390	-0.3511200	N	-2.0565380	1.1815210	0.4461000
P	-2.8413280	2.5971170	0.0739210	P	-3.0692290	2.4056730	0.2446470
C	-4.4539010	2.9252270	-0.6513320	C	-3.1063420	3.3577760	1.7749000
N	-2.0410650	-1.5922380	-0.5600570	N	-2.7173650	-1.0671690	-1.0161450
P	-3.3792820	-2.3473770	0.0093450	P	-3.4946880	-2.1709360	-0.1576970
C	-3.1471860	-2.7162000	1.7562210	C	-3.7174230	-1.7775310	1.5993800
C	-4.8786470	-1.3673940	-0.1851220	C	-5.1413290	-2.4042630	-0.8592420
C	-3.5383160	-3.8803020	-0.9124150	C	-2.6801250	-3.7891140	-0.2128830
C	-1.6883350	3.8539090	-0.5010840	C	-4.7755990	1.9172680	-0.1254480
C	-2.9554960	2.6744040	1.8680850	C	-2.5682860	3.5354130	-1.0810650
H	0.6943720	-2.2896060	1.8566990	H	1.5025750	3.0007260	0.7931710
H	0.7239630	1.8244320	2.2022580	H	1.7131480	1.2037590	-2.9106770
H	4.1812160	-2.2895020	1.6128910	H	4.6893580	1.8261390	1.5551310
H	4.1995510	1.8256180	1.9332140	H	4.9408630	0.0704340	-2.1728800
H	-0.0529380	-0.2925880	2.7079660	H	1.0849200	2.8779520	-1.4848190
H	5.4288560	-0.1509320	0.6554690	H	5.6257920	-0.3501190	0.3562340
H	5.0364140	-0.2787890	2.3426260	H	5.7960120	1.2197500	-0.3683280
H	5.3007170	0.0143440	-1.0869200	H	4.9424270	-1.7984410	1.0598250
H	4.6676470	0.1354680	-2.7001710	H	3.8257280	-3.0357590	1.5510870
H	-0.2735930	0.1041640	-2.4346100	H	-0.6476840	-1.6422490	0.5182090
H	3.9028470	2.1372070	-1.8530140	H	3.5010170	-3.1378390	-0.7240260
H	0.4348620	2.1117550	-1.5995930	H	0.3086620	-2.0874020	-1.5448660
H	2.0693690	1.3197600	-3.0486260	H	1.3468930	-3.2713500	0.1789330
H	2.0923800	-0.9832960	-3.2422610	H	1.1400070	-2.2345780	2.2332670
H	3.9199550	-1.9768550	-2.1766310	H	3.1685770	-1.3555230	2.9842530
H	0.4533010	-2.0238010	-1.9432160	H	-0.0268170	-0.2179510	2.1551660

H	2.5325250	-1.4921380	3.0648600	H	3.6583330	3.0840800	-0.1283550
H	2.5559180	0.8154270	3.2543560	H	3.7591250	2.0858890	-2.2146580
H	1.2274290	2.7304290	0.3097940	H	1.6512350	-0.9299670	-2.7746160
H	3.4091090	2.7366890	0.1542050	H	3.6625400	-1.6616500	-2.2784310
H	3.4070840	-2.8972780	-0.3019960	H	3.2782690	0.7898940	2.8025920
H	1.2121890	-2.9109800	-0.1329590	H	1.2499820	1.4790510	2.3055930
H	-4.8118090	-0.4768510	0.4423760	H	-5.7030620	-1.4720920	-0.7713380
H	-4.9919360	-1.0734080	-1.2302460	H	-5.0446670	-2.6666340	-1.9147600
H	-5.7396720	-1.9647350	0.1223860	H	-5.6704250	-3.2014710	-0.3326310
H	-3.6731240	-3.6520500	-1.9711370	H	-2.5548250	-4.0871720	-1.2558750
H	-4.4008770	-4.4393220	-0.5445980	H	-3.2827210	-4.5354460	0.3101230
H	-2.6310520	-4.4723450	-0.7784330	H	-1.6963950	-3.7290810	0.2587630
H	-2.2185420	-3.2750590	1.8849390	H	-4.2694030	-0.8413570	1.6988470
H	-3.9885770	-3.3114170	2.1175980	H	-2.7435280	-1.6632210	2.0809210
H	-3.0940240	-1.7815210	2.3182700	H	-4.2697980	-2.5802410	2.0932090
H	-1.5912050	3.7883800	-1.5864330	H	-5.4060730	2.8001300	-0.2536520
H	-2.0664080	4.8419610	-0.2290490	H	-4.7872440	1.3236650	-1.0424540
H	-0.7157660	3.7014680	-0.0298280	H	-5.1668320	1.3123180	0.6953810
H	-3.6601540	1.9153990	2.2127850	H	-2.5837490	3.0103160	-2.0392130
H	-1.9712020	2.4869720	2.3015340	H	-3.2498830	4.3875060	-1.1314980
H	-3.3034730	3.6651000	2.1678120	H	-1.5542720	3.8905450	-0.8844450
H	-4.3786670	2.8549800	-1.7380830	H	-3.4405630	2.7118000	2.5892070
H	-4.7802030	3.9293070	-0.3713150	H	-3.7887540	4.2045760	1.6768390
H	-5.1741510	2.1915180	-0.2860330	H	-2.1004940	3.7215510	1.9938430
H	-0.6796940	-0.1273850	1.1430860	H	-1.2805240	0.5754990	-1.8584650
				H	0.0152470	1.6639130	-0.8804930
**III-Pb_2 (dication)**				**III-Pb_2 (protonated)**			
C	4.9369180	-0.4581770	-1.6311960	C	1.6657720	1.7044230	-1.1444360
C	4.1174590	0.8401620	-1.6471610	C	2.3858710	0.6056910	-1.9443560
C	2.7787530	0.3674400	-2.2435310	C	3.8717340	0.9451560	-1.6901270
C	2.6783110	-1.1683300	-1.9816180	C	3.9329720	1.7825900	-0.3747840
C	3.9679020	-1.5852870	-1.2494610	C	2.4846760	1.9474200	0.1131220
C	1.4570250	-1.4103460	-1.0651950	C	4.7357280	0.9578430	0.6494150
C	0.6364760	-0.1414860	-1.3909080	C	5.5767350	0.0498900	-0.2602860
C	1.6134900	1.0425490	-1.4959810	C	4.6756420	-0.3466000	-1.4374150
C	3.7753910	1.6356140	-0.3456580	C	2.2223960	-0.9261170	-1.6583500
C	2.2123920	1.7442950	-0.2392060	C	3.6426170	-1.5095830	-1.3186780
C	3.5778470	-1.8704860	0.2342160	C	2.2896410	0.9949740	1.3248100
C	2.0149580	-1.7665510	0.3512110	C	3.6997100	0.3998510	1.6819570
C	4.3258460	1.2861970	1.0726560	C	3.5031320	-2.4386100	-0.0676600
C	3.1046400	1.0420660	1.9815690	C	2.0427950	-2.3227310	0.3995830
C	1.8101580	1.4434570	1.2407460	C	1.2140570	-1.5589580	-0.6486710
C	3.0326900	-0.4972400	2.2414800	C	2.0427210	-1.4558400	1.7008270
C	1.6923060	-0.9917710	1.6643770	C	1.2367250	-0.1657930	1.4020550
C	0.8794410	0.2971770	1.6444520	C	0.3239160	-0.7093580	0.2698000
C	4.2003960	-1.1405350	1.4688150	C	3.5118240	-1.1125530	2.0075170
C	5.1773920	0.0350540	1.3254140	C	4.2740960	-2.2009540	1.2397960
P	-0.9536340	0.1819120	-0.6373530	P	-1.1406050	0.0957360	-0.4755440
N	-1.3094190	1.6813850	-0.6558470	N	-2.2706040	-1.0097420	-0.7777670
P	-2.5631520	2.6212450	-0.1729390	P	-2.8912340	-2.2611050	-0.0013720
C	-2.2385190	4.2683240	-0.8434970	C	-1.8979430	-3.7773270	-0.2044370
C	-0.8709190	4.8198030	-0.4306050	C	-1.7115990	-4.1810660	-1.6675500
N	-1.9194530	-0.9451360	-0.2778390	N	-1.5295990	1.3330230	0.4708830
P	-2.4487180	-2.4568790	-0.0343250	P	-2.2915030	2.7158830	0.2118630
C	-1.6191450	-3.1953630	1.3981250	C	-1.9147360	3.4317230	-1.4230800
C	-1.7428680	-2.3706700	2.6807280	C	-2.5884410	4.7750600	-1.7115010
C	-2.1366060	-3.4857150	-1.4916620	C	-1.7678690	3.8942800	1.4889550
C	-2.8058010	-2.9597130	-2.7639780	C	-0.2533840	4.0975180	1.5241950
C	-4.2304370	-2.3472140	0.2711250	C	-4.1027890	2.5941340	0.3535210
C	-4.9151010	-3.6965850	0.5023100	C	-4.7341150	1.6977900	-0.7132260
C	-4.1395930	1.9809170	-0.7972300	C	-3.0943780	-2.0610390	1.8003990
C	-5.3573500	2.8106420	-0.3796950	C	-3.8924950	-0.8174030	2.1944510
C	-2.6115860	2.7056350	1.6391000	C	-4.5293120	-2.5750310	-0.7231620
C	-3.0592280	1.4034470	2.3074850	C	-5.2481970	-3.8198860	-0.2020210
H	1.2148010	-1.6965060	2.3504200	H	2.3094060	2.9686660	0.4629840
H	1.4014430	2.3695120	1.6520490	H	2.1420350	0.7474110	-3.0009800
H	4.6783050	-1.9100720	2.0811140	H	5.4179970	1.6060260	1.2073970
H	4.8850990	2.1591560	1.4201710	H	5.3123930	-0.5715920	-2.2981370
H	0.5518120	0.4993890	2.6713960	H	1.7177140	2.6147850	-1.7556680
H	6.0107400	-0.1162570	0.6460090	H	6.1040170	-0.7650540	0.2272950
H	5.6299870	0.1713020	2.3159690	H	6.3605210	0.6935220	-0.6810770
H	5.8684160	-0.4275060	-1.0746670	H	5.3450260	-2.0483380	1.1445540
H	5.2253260	-0.6453860	-2.6734330	H	4.1611500	-3.1232080	1.8247860
H	0.2944770	-0.3125820	-2.4264490	H	-0.2293890	-1.4764730	0.8349720
H	4.6031950	1.5429420	-2.3292820	H	3.7116350	-3.4578670	-0.4054850
H	1.1468360	1.8105160	-2.1150130	H	0.5791660	-2.2508660	-1.2112310
H	2.7208070	0.5691550	-3.3156290	H	1.6071050	-3.3057200	0.6030630
H	2.5608370	-1.7031600	-2.9268730	H	1.5825630	-2.0152200	2.5201640
H	4.3479130	-2.5222210	-1.6651380	H	3.7132640	-1.2391970	3.0752250
H	0.8940680	-2.2756860	-1.4214650	H	0.5778400	0.0709190	2.2405670
H	3.0994210	-0.7023300	3.3125140	H	4.4036630	2.7512160	-0.5644900
H	3.1968310	1.5789710	2.9286770	H	4.2918870	1.5183860	-2.5211300
H	1.9551790	2.7965870	-0.3760950	H	1.9326460	-1.3459350	-2.6245530
H	4.1424280	2.6455260	-0.5417890	H	3.8999920	-2.1925870	-2.1320940
H	3.8285840	-2.9226050	0.3861470	H	3.9968170	0.8524160	2.6313710
H	1.6453450	-2.7792870	0.5284590	H	2.0057900	1.6474680	2.1548180

H	-1.6041970	2.9860890	1.9616750	H	-3.5652070	-2.9746500	2.1787380
H	-3.2769510	3.5345320	1.9020540	H	-2.0884930	-2.0272570	2.2338900
H	-3.0499370	4.9229440	-0.5114640	H	-4.3716280	-2.6319620	-1.8046830
H	-2.3144750	4.1765050	-1.9314220	H	-5.1214680	-1.6718780	-0.5444550
H	-4.2180110	0.9508590	-0.4353390	H	-2.3738430	-4.5772530	0.3720490
H	-4.0446000	1.9308950	-1.8861930	H	-0.9297870	-3.5835060	0.2704400
H	-4.6559290	-1.8293540	-0.5941860	H	-2.1274470	3.4877480	2.4399850
H	-4.3559500	-1.6811900	1.1304100	H	-2.2991100	4.8364880	1.3218150
H	-2.0495270	-4.1939650	1.5255680	H	-4.3017300	2.2070010	1.3581910
H	-0.5704920	-3.3390050	1.1210340	H	-4.5140790	3.6084420	0.3179060
H	-2.4789860	-4.4967050	-1.2490240	H	-2.2075830	2.6876500	-2.1714790
H	-1.0498290	-3.5366690	-1.6078050	H	-0.8246630	3.5183360	-1.4806660
H	-0.0686740	4.1426090	-0.7337720	H	-0.8067960	0.5307410	-1.7686300
H	-0.7090030	5.7824830	-0.9184520	H	-5.7987460	1.5663500	-0.5078080
H	-0.8066130	4.9762120	0.6487980	H	-4.6384480	2.1352970	-1.7111930
H	-3.0148750	1.5200580	3.3914140	H	-4.2620240	0.7111190	-0.7291690
H	-4.0849410	1.1390610	2.0386790	H	0.1171240	4.5393070	0.5945440
H	-2.4120970	0.5663700	2.0373430	H	0.0159780	4.7672650	2.3435820
H	-5.3006480	3.8331750	-0.7598590	H	0.2556970	3.1426830	1.6755240
H	-6.2584870	2.3520550	-0.7904110	H	-2.2916060	5.5408080	-0.9900610
H	-5.4697280	2.8506210	0.7069160	H	-2.2980360	5.1248110	-2.7044230
H	-1.2201140	-2.8838140	3.4896690	H	-3.6784340	4.6922680	-1.6945170
H	-1.2895160	-1.3833310	2.5659040	H	-5.4248120	-3.7672320	0.8755990
H	-2.7841070	-2.2384680	2.9846480	H	-6.2198200	-3.9134010	-0.6915940
H	-4.5122200	-4.2137380	1.3764630	H	-4.6826280	-4.7317980	-0.4119120
H	-5.9795680	-3.5308780	0.6765230	H	-4.9004270	-0.8306070	1.7698080
H	-4.8191040	-4.3548620	-0.3647800	H	-3.9894470	-0.7683630	3.2811430
H	-2.5156680	-3.5878630	-3.6077380	H	-3.3816680	0.0864730	1.8549050
H	-3.8954480	-2.9855790	-2.6871980	H	-1.0210560	-5.0240380	-1.7374850
H	-2.4985310	-1.9344260	-2.9854110	H	-2.6586780	-4.4848850	-2.1222370
H	-0.0847740	0.2241600	1.1205640	H	-1.3027820	-3.3545210	-2.2552950
				H	0.6149570	1.5621690	-0.9348650
**III-Pc2 (dication)**				**III-Pc2 (protonated)**			
C	-5.6082000	-0.4455660	-1.0350360	C	-2.0340150	1.7266980	-1.4476790
C	-4.5591490	-1.5465520	-1.2485640	C	-2.7792880	2.1782180	-0.1994910
C	-3.4898460	-0.8122760	-2.0795820	C	-4.2497990	2.1528440	-0.6505170
C	-3.6505750	0.7141440	-1.7910270	C	-4.3269230	1.1939810	-1.8797100
C	-4.8350120	0.8609450	-0.8143620	C	-2.9008350	0.6523750	-2.1224690
C	-2.0878740	-1.2173570	-1.5920570	C	-5.2738040	0.0408090	-1.4911050
C	-2.2687870	-2.0187440	-0.2705100	C	-6.0914920	0.6574140	-0.3478400
C	-3.8097280	-2.2433740	-0.0647300	C	-5.1248520	1.5377400	0.4581760
C	-4.1308310	-2.0081360	1.4428220	C	-2.6787850	1.3334330	1.1024430
C	-2.8372280	-1.4980340	2.1055670	C	-4.1439800	0.9545310	1.5288050
C	-1.6540200	-1.6290120	1.1166540	C	-2.9121700	-0.8509950	-1.6842820
C	-3.0459250	0.0212010	2.4005530	C	-4.3844800	-1.2231980	-1.2747940
C	-1.9795860	0.7972230	1.6063800	C	-4.1394900	-0.5337650	1.9949230
C	-0.9189890	-0.3000910	1.4339500	C	-2.7294840	-1.0829330	1.7133190
C	-5.1621600	-0.9651680	1.8926930	C	-1.7796510	0.0642180	1.2915850
C	-4.4469330	0.3937290	1.8821730	C	-2.8562970	-2.0574160	0.4975150
C	-4.2353550	1.2298960	0.5774940	C	-1.9632500	-1.4963080	-0.6255400
C	-2.6930600	1.4554170	0.3879870	C	-0.9625620	-0.6815570	0.2075690
C	-2.3459490	1.1923200	-1.1141810	C	-4.3290590	-2.0418240	0.0564580
C	-1.3590590	0.1175700	-1.5887930	C	-5.0409760	-1.5902600	1.3407190
P	0.6052940	0.1761030	0.6228680	P	0.5783040	-0.0861090	-0.5721800
N	0.7806000	1.7004870	0.5275700	N	0.9962700	1.2378980	0.2337190
P	1.8040260	2.7821310	-0.1544110	P	2.2609060	2.2083300	0.0786610
C	0.7553090	3.9363000	-1.0698930	C	3.7449250	1.4951250	0.8492530
C	-0.2691440	4.6500430	-0.1784580	C	5.0056210	2.3642090	0.8437400
C	-1.3051640	5.4003290	-1.0111440	C	6.2052000	1.5948150	1.3934130
N	1.6889370	-0.8479280	0.2865750	N	1.6583860	-1.2729480	-0.6967720
P	2.3467500	-2.3307720	0.2496130	P	2.2399210	-2.4390870	0.2264700
C	1.3446400	-3.4462850	-0.7626580	C	2.7085160	-1.9661160	1.9259230
C	1.1361150	-2.9562320	-2.2006880	C	1.6000940	-1.3071240	2.7534660
C	0.2319470	-3.9060400	-2.9817750	C	2.0877900	-0.9470180	4.1551680
C	2.4740550	-3.0010590	1.9258050	C	3.7470830	-3.0721310	-0.5639980
C	3.3741220	-2.1776090	2.8559590	C	4.8168330	-1.9985740	-0.7920380
C	3.3673900	-2.7450930	4.2734190	C	6.0221400	-2.5477940	-1.5513220
C	3.9939250	-2.1500630	-0.4730370	C	1.0866440	-3.8437130	0.3873430
C	4.8143900	-3.4432040	-0.5467270	C	0.6343540	-4.4187540	-0.9604600
C	6.1840150	-3.1820210	-1.1699290	C	-0.5721370	-5.3415210	-0.8070250
C	2.6619250	3.6343180	1.1907740	C	2.6891080	2.6283550	-1.6441410
C	3.4921470	4.8470780	0.7502720	C	3.4070610	1.5108740	-2.4138070
C	4.1605510	5.5160270	1.9495450	C	3.5912750	1.8726820	-3.8853800
C	3.0161490	2.0360240	-1.2774470	C	1.8594460	3.7556380	0.9336740
C	2.4172380	1.2649250	-2.4614620	C	0.5578790	4.3881190	0.4272110
C	3.5129820	0.7278130	-3.3794100	C	0.1708660	5.6209400	1.2398190
H	-1.5474600	1.5958600	2.2116690	H	-2.6716210	0.6587840	-3.1919130
H	-1.0058880	-2.4523830	1.4272630	H	-2.4724950	3.1989440	0.0486630
H	-4.9485940	1.0489300	2.5991800	H	-5.9545330	-0.1858870	-2.3170560
H	-4.4110960	-2.9765710	1.8654250	H	-5.7091290	2.3168690	0.9568030
H	-0.5204190	-0.4695950	2.4494260	H	-2.0137260	2.5796980	-2.1383380
H	-6.1218470	-1.0041640	1.3870410	H	-6.7028130	-0.0343000	0.2246100
H	-5.3776480	-1.1835010	2.9464070	H	-6.8005270	1.3491320	-0.8216580
H	-6.3890450	-0.6604730	-0.3117360	H	-6.0881120	-1.3211890	1.2387460
H	-6.1246510	-0.3348300	-1.9971080	H	-5.0268970	-2.4593110	2.0116860
H	-1.0958560	0.3608820	-2.6249630	H	-0.4938160	-1.4604230	0.8270500

H	-5.0159500	-2.3430640	-1.8421500	H	-4.3359130	-0.5337740	3.0710490
H	-1.6109010	-1.8797750	-2.3176500	H	-1.0851230	0.2949510	2.1028230
H	-3.6073330	-1.0092400	-3.1477850	H	-2.3265440	-1.6176890	2.5782790
H	-3.8300900	1.2564740	-2.7223530	H	-2.5341600	-3.0616360	0.7899890
H	-5.4728980	1.6954120	-1.1182240	H	-4.6628180	-3.0562650	-0.1804490
H	-2.0259100	2.1497000	-1.5341040	H	-1.4240350	-2.3089220	-1.1228000
H	-2.9477150	0.2112770	3.4718140	H	-4.6969980	1.7341780	-2.7555020
H	-2.6213290	-2.0337410	3.0327600	H	-4.5995650	3.1512180	-0.9276200
H	-1.8341870	-3.0000560	-0.4787250	H	-2.2981780	2.0277010	1.8558020
H	-3.9886360	-3.3092720	-0.2217340	H	-4.3623580	1.5262140	2.4343850
H	-4.6631860	2.2071190	0.8122010	H	-4.7407390	-1.9416050	-2.0173300
H	-2.5117180	2.5241170	0.5181800	H	-2.6943310	-1.3977850	-2.6048070
H	3.6479990	2.8573070	-1.6355470	H	1.7454050	2.8897010	-2.1373140
H	3.6500870	1.3833340	-0.6673940	H	3.3012550	3.5373810	-1.6291650
H	0.2489260	3.3403250	-1.8374200	H	2.7045260	4.4441280	0.8233010
H	1.4032230	4.6486680	-1.5916790	H	1.7760180	3.5071140	1.9980430
H	3.2874470	2.8834330	1.6860760	H	3.9240370	0.5535710	0.3164840
H	1.8864340	3.9283410	1.9063630	H	3.4582390	1.2308610	1.8739290
H	4.5078730	-1.3882660	0.1234630	H	3.4381190	-3.5031740	-1.5226610
H	3.8472290	-1.7233680	-1.4709480	H	4.1325600	-3.8914080	0.0534230
H	0.3857600	-3.5620270	-0.2452610	H	1.5648740	-4.6114710	1.0055940
H	1.8333200	-4.4267480	-0.7456610	H	0.2192850	-3.4823230	0.9531280
H	2.8395720	-4.0300290	1.8318910	H	3.5655460	-1.2886870	1.8465230
H	1.4534180	-3.0592090	2.3195720	H	3.0713320	-2.8771330	2.4164890
H	0.6972170	-1.9526570	-2.1862380	H	0.3143840	0.2002210	-1.9226000
H	2.1016830	-2.8695670	-2.7096230	H	0.3861630	-3.5983780	-1.6430960
H	4.9423330	-3.8633610	0.4566090	H	1.4635230	-4.9673550	-1.4193730
H	4.2797050	-4.1925290	-1.1400910	H	4.3727550	-1.1688850	-1.3525300
H	4.3984910	-2.1725980	2.4684370	H	5.1514950	-1.5948970	0.1707160
H	3.0344020	-1.1366450	2.8724920	H	1.2597560	-0.3969300	2.2475160
H	4.2575030	4.5373470	0.0311910	H	0.7362780	-1.9781690	2.8303220
H	2.8493480	5.5721920	0.2394770	H	0.6667730	4.6627800	-0.6282140
H	-0.7708100	3.9130680	0.4575690	H	-0.2396660	3.6399530	0.4785770
H	0.2475220	5.3475350	0.4893900	H	4.3869100	1.3253610	-1.9591620
H	1.8154380	0.4289150	-2.0912250	H	2.8486740	0.5715540	-2.3332560
H	1.7468510	1.9127690	-3.0348910	H	5.2260450	2.7026310	-0.1750260
H	-0.7412270	-4.0178950	-2.4921350	H	4.8361080	3.2641240	1.4441250
H	0.0609910	-3.5295710	-3.9931170	H	2.6238570	2.0240120	-4.3741710
H	0.6828360	-4.8995650	-3.0589000	H	4.1710970	2.7947540	-3.9924620
H	4.0161420	-2.1552340	4.9254120	H	4.1189070	1.0767830	-4.4173910
H	2.3583480	-2.7317620	4.6962250	H	0.9475060	6.3903280	1.1856900
H	3.7256380	-3.7787930	4.2834510	H	-0.7609630	6.0541930	0.8662580
H	6.7524310	-2.4604420	-0.5755220	H	0.0249880	5.3634710	2.2937170
H	6.7634270	-4.1065090	-1.2271860	H	6.4187360	0.7158460	0.7763060
H	6.0816300	-2.7811020	-2.1828830	H	7.0998840	2.2224720	1.4084830
H	3.4136360	5.8635590	2.6695680	H	6.0140320	1.2507900	2.4147910
H	4.8294370	4.8195010	2.4637320	H	-0.3549800	-6.1648700	-0.1196400
H	4.7497960	6.3783670	1.6286590	H	-1.4340100	-4.7926580	-0.4122560
H	-0.8294860	6.1444980	-1.6567480	H	-0.8597920	-5.7703580	-1.7704650
H	-1.8674100	4.7099030	-1.6484340	H	2.9349440	-0.2546530	4.1075740
H	-2.0172400	5.9175180	-0.3635020	H	1.2919320	-0.4672540	4.7306600
H	3.0795190	0.1358830	-4.1889970	H	2.4131690	-1.8379540	4.7009130
H	4.0871680	1.5456670	-3.8242110	H	6.7723800	-1.7670550	-1.7026780
H	4.2131150	0.0912520	-2.8292520	H	6.4931720	-3.3682600	-1.0011970
H	-0.3711130	0.0852400	-1.1110660	H	5.7248810	-2.9271690	-2.5338720
				H	-0.9942870	1.4943520	-1.2648810
**III-Pd$_2$ (dication)**				**III-Pd$_2$ (protonated)**			
C	-1.3737310	0.5253420	1.6606310	C	-5.5496510	-1.7576540	-1.2165240
C	-2.4552530	1.4982380	1.1873090	C	-4.8095860	-2.2590890	0.0327800
C	-3.5967840	1.1661660	2.1724250	C	-3.3450330	-2.2313740	-0.4331860
C	-3.3415610	-0.2801070	2.7070250	C	-3.2513930	-1.1958590	-1.6004580
C	-2.0476720	-0.7889800	2.0409910	C	-4.6748540	-0.6573780	-1.8329560
C	-3.0714060	1.4590800	-0.2478610	C	-2.3101030	-0.0556000	-1.1367120
C	-4.6178440	1.2269420	-0.1011530	C	-1.4644090	-0.8454570	-0.1047360
C	-4.9434850	1.1359140	1.4230280	C	-2.4400810	-1.7164730	0.7006950
C	-2.5760550	0.5531030	-1.4166120	C	-4.8515700	-1.5097640	1.4052870
C	-3.7618940	-0.3334130	-1.8444580	C	-3.3772990	-1.1371390	1.8062670
C	-5.0497050	0.1627270	-1.1612060	C	-4.6968880	0.8046910	-1.2925030
C	-1.4902130	-0.5224860	-1.2297940	C	-3.2305630	1.1858190	-0.8752700
C	-2.1413170	-1.7480470	-0.5562250	C	-5.7530100	-0.2716720	1.7023770
C	-3.4884100	-1.7755250	-1.3116240	C	-4.8144970	0.8759760	2.1274270
C	-4.6250070	-2.1164130	-0.3297310	C	-3.3758170	0.3443170	2.3135720
C	-4.0156780	-2.0685220	1.1065950	C	-4.7780720	1.8932340	0.9450410
C	-2.4675390	-1.8402140	0.9685490	C	-3.3192560	1.9636240	0.4658600
C	-4.5345310	-1.1450650	2.2560980	C	-2.5359630	1.4587440	1.6684280
C	-5.7358870	-1.1552770	-0.7745180	C	-5.6656150	1.3180050	-0.1753610
C	-5.6301210	-0.0925580	2.0350690	C	-6.6025150	0.3845990	0.6058200
P	0.1442050	0.0314600	-0.7696040	P	0.0848470	-0.3148310	0.7123220
N	1.2297720	-0.9438260	-0.3235250	N	0.5956750	1.0132300	-0.0264460
P	1.8940640	-2.2993930	0.2638520	P	1.4288730	2.3134180	0.3786640
C	1.0107190	-3.7696620	-0.3139410	C	0.3430330	3.7604850	0.5573480
C	0.7830680	-3.7827570	-1.8307770	C	-0.5124050	4.0232860	-0.6833850
C	-0.1680870	-4.9014340	-2.2558430	C	-1.5073660	5.1645060	-0.4707220
C	-0.4604330	-4.8646370	-3.7532310	C	-2.4738180	5.3140460	-1.6428730
N	0.4004620	1.4923800	-1.1952520	N	1.0877710	-1.5782680	0.7638780
P	1.6497710	2.5356070	-0.9700520	P	2.0062230	-2.2361780	-0.3725530

C	2.1504780	2.4762910	0.7720710	C	3.0780310	-1.0153570	-1.1940520
C	3.2120080	3.5001440	1.1862290	C	4.1813010	-1.5591940	-2.1049730
C	3.5617770	3.3624550	2.6693620	C	5.0870890	-0.4426970	-2.6393030
C	4.6203010	4.3697920	3.1100610	C	5.9315240	0.2357460	-1.5604530
C	0.9776520	4.1625420	-1.3718950	C	3.0797780	-3.4655360	0.4151020
C	-0.2112190	4.5195370	-0.4699490	C	4.0152520	-2.8403600	1.4555760
C	-0.9536430	5.7661570	-0.9484470	C	4.9491750	-3.8550680	2.1197640
C	-2.1560790	6.0816510	-0.0620390	C	5.9627700	-4.4757100	1.1595190
C	3.0862730	2.1423430	-2.0046250	C	1.0767420	-3.0854520	-1.6902650
C	2.8789530	2.1814670	-3.5277560	C	0.1096390	-4.1545550	-1.1693650
C	1.9532010	1.1076150	-4.1109280	C	-0.8712610	-4.6190040	-2.2457380
C	2.3969900	-0.3247620	-3.8127960	C	-1.7930990	-5.7312820	-1.7526830
C	3.6026050	-2.3453950	-0.3245140	C	2.6079760	2.6667950	-0.9576940
C	4.4503460	-1.1835330	0.2103680	C	3.5079450	3.8870590	-0.7564640
C	5.7870980	-1.0455080	-0.5226300	C	4.4781130	4.0653560	-1.9255930
C	6.6976450	-2.2629550	-0.3752960	C	5.4045770	5.2637590	-1.7388450
C	1.8564620	-2.2056170	2.0683220	C	2.3610070	2.2131460	1.9410970
C	2.5881100	-3.3569210	2.7703190	C	3.4697650	1.1568870	1.9578350
C	2.5500230	-3.2166240	4.2958220	C	4.2849710	1.1972040	3.2505800
C	3.2842880	-1.9835430	4.8203600	C	5.3908490	0.1449230	3.2747900
H	-1.5534020	-2.6285000	-0.8204120	H	-3.0356390	2.9994080	0.2646600
H	-2.2629400	1.2059490	-2.2328550	H	-3.1219590	0.3057200	3.3766480
H	-4.9683650	-3.1407780	-0.4961660	H	-6.2713520	2.1108800	-0.6241100
H	-5.6833890	0.6782670	-1.8876200	H	-6.4129460	-0.5481190	2.5300270
H	-1.2698150	-0.8660610	-2.2562020	H	-2.4944070	2.2759860	2.4002780
H	-6.5850980	-1.0712710	-0.1037320	H	-7.2125390	-0.2906280	0.0126310
H	-6.1399490	-1.5697040	-1.7069330	H	-7.3137430	1.0411390	1.1242000
H	-6.5270350	-0.4363170	1.5289320	H	-6.5987570	-1.5091400	-1.0861210
H	-5.9595210	0.2125590	3.0365250	H	-5.5337770	-2.5936630	-1.9281790
H	-0.9400950	0.9468630	2.5750980	H	-0.9973060	-1.5877700	-0.7679020
H	-5.5340850	2.0210020	1.6746240	H	-5.1244060	-3.2890450	0.2245510
H	-2.0900580	2.5155510	1.3506640	H	-1.8791290	-2.5441120	1.1470710
H	-3.6164540	1.8756940	3.0029740	H	-3.0153990	-3.2148490	-0.7789670
H	-3.2443010	-0.2676710	3.7951260	H	-2.8556200	-1.6799920	-2.4984080
H	-4.8509070	-1.8030870	3.0699440	H	-4.8888570	-0.6072400	-2.9045420
H	-1.4186760	-1.3031700	2.7722620	H	-1.6305990	0.2260370	-1.9445530
H	-3.4473400	-2.4827420	-2.1435140	H	-5.1364420	2.8719230	1.2760270
H	-3.8618750	-0.3486380	-2.9321730	H	-5.1733740	1.3673090	3.0359820
H	-2.9353540	2.4745320	-0.6275950	H	-3.1358880	-1.7227630	2.6965210
H	-5.1007930	2.1495780	-0.4306320	H	-5.1837280	-2.2705380	2.1161010
H	-4.1442250	-3.0821680	1.4923540	H	-4.9418090	1.4179560	-2.1634550
H	-1.9811210	-2.7659280	1.2862500	H	-2.8786970	1.9197000	-1.6058250
H	0.6665010	4.1157110	-2.4214060	H	1.6319880	2.0231310	2.7374620
H	1.7829760	4.9009090	-1.3006000	H	2.7746770	3.2109570	2.1291520
H	-0.9102210	3.6747920	-0.4462840	H	3.0316310	0.1597480	1.8300740
H	0.1357910	4.6762740	0.5583250	H	4.1510930	1.3196420	1.1121600
H	-1.2853250	5.6110450	-1.9819170	H	3.6139640	1.0514250	4.1055320
H	-0.2648100	6.6187870	-0.9641010	H	4.7220100	2.1966840	3.3638740
H	-1.8447510	6.2665140	0.9712120	H	4.9717730	-0.8655060	3.2494600
H	-2.6873190	6.9685610	-0.4173260	H	6.0546320	0.2525620	2.4099850
H	-2.8643870	5.2458940	-0.0529920	H	5.9992550	0.2317050	4.1790190
H	3.8558570	2.8671540	-1.7175750	H	-0.2962330	3.5578930	1.4252370
H	3.4461510	1.1618130	-1.6744050	H	0.9606060	4.6301080	0.8089510
H	3.8736420	2.0706420	-3.9725140	H	-1.0509380	3.1047420	-0.9426410
H	2.5246910	3.1746600	-3.8224480	H	0.1296170	4.2626430	-1.5399520
H	0.9270890	1.2607170	-3.7594360	H	-2.0733500	4.9838570	0.4520310
H	1.9263570	1.2544780	-5.1955290	H	-0.9577390	6.1004470	-0.3147750
H	3.4180880	-0.5020440	-4.1662610	H	-1.9334930	5.4961900	-2.5776000
H	2.3785050	-0.5471720	-2.7410790	H	-3.0701760	4.4040380	-1.7750060
H	1.7387970	-1.0471000	-4.3032950	H	-3.1644800	6.1468380	-1.4849790
H	2.5004740	1.4549830	0.9611310	H	2.0143910	2.7604060	-1.8738370
H	1.2322670	2.6093880	1.3556010	H	3.2135020	1.7634700	-1.0793760
H	4.1204870	3.3650020	0.5884910	H	2.8995990	4.7931280	-0.6481040
H	2.8485240	4.5159550	0.9913240	H	4.0830720	3.7803600	0.1717630
H	3.9166320	2.3423080	2.8601610	H	3.9046730	4.1785030	-2.8535200
H	2.6516500	3.4939460	3.2665830	H	5.0735550	3.1505800	-2.0378990
H	5.5478500	4.2378910	2.5437260	H	6.0874630	5.3706770	-2.5860670
H	4.8548490	4.2529180	4.1713720	H	6.0082370	5.1545670	-0.8318760
H	4.2744500	5.3964830	2.9527650	H	4.8315890	6.1924880	-1.6491800
H	3.5503710	-2.3193470	-1.4192270	H	1.7963740	-3.5204130	-2.3926130
H	4.0200090	-3.3183400	-0.0457170	H	0.5365580	-2.3042050	-2.2401900
H	4.6313690	-1.3232030	1.2828390	H	-0.4543860	-3.7648510	-0.3124700
H	3.8918720	-0.2469430	0.1044970	H	0.6800100	-5.0138840	-0.7979100
H	6.2964040	-0.1584940	-0.1317100	H	-0.3121720	-4.9624820	-3.1243140
H	5.5929660	-0.8511800	-1.5849150	H	-1.4690600	-3.7594670	-2.5765030
H	6.2676050	-3.1520010	-0.8468670	H	-2.5077790	-6.0251900	-2.5261100
H	7.6675500	-2.0803060	-0.8451250	H	-1.2182640	-6.6187360	-1.4688370
H	6.8726840	-2.4940500	0.6808340	H	-2.3626970	-5.4111130	-0.8732400
H	2.2915030	-1.2348110	2.3257120	H	2.4092850	-0.3435340	-1.7436390
H	0.8009790	-2.1728360	2.3601510	H	3.5061490	-0.4296380	-0.3749850
H	3.6340050	-3.3905640	2.4415910	H	3.7333250	-2.0964400	-2.9473150
H	2.1320190	-4.3105150	2.4853740	H	4.7997180	-2.2825990	-1.5592570
H	1.5059190	-3.2006460	4.6302380	H	5.7537330	-0.8729400	-3.3940260
H	3.0007710	-4.1168460	4.7259160	H	4.4742880	0.3058020	-3.1574530
H	3.3151890	-1.9884290	5.9130210	H	5.3189760	0.7767860	-0.8310330
H	4.3164330	-1.9584540	4.4544770	H	6.5260490	-0.5000030	-1.0081560
H	2.7966520	-1.0528460	4.5140170	H	6.6192460	0.9617150	-2.0028480

H	1.5829290	-4.6469950	0.0061450	H	2.4286140	-4.2117920	0.8828030
H	0.0596600	-3.7944140	0.2306950	H	3.6339950	-3.9731500	-0.3813380
H	0.3676390	-2.8208690	-2.1547540	H	4.6256570	-2.0564780	0.9874000
H	1.7405880	-3.8954150	-2.3511800	H	3.4025850	-2.3484710	2.2184750
H	-1.1071720	-4.8088130	-1.6954010	H	4.3513850	-4.6433320	2.5932120
H	0.2644460	-5.8694590	-1.9786360	H	5.4852960	-3.3450930	2.9282790
H	0.4597430	-4.9693990	-4.3368640	H	5.4770190	-5.0835530	0.3898630
H	-1.1363960	-5.6736530	-4.0417950	H	6.5455100	-3.6978990	0.6535530
H	-0.9304700	-3.9162570	-4.0346860	H	6.6615820	-5.1243610	1.6947420
H	-0.4741760	0.4303030	1.0326960	H	-0.1884540	-0.1033990	2.0745290
				H	-1.5028230	1.2351760	1.4380270
**III-Pe$_2$ (dication)**				**III-Pe$_2$ (protonated)**			
C	-5.0149740	-1.7441080	-2.4156970	C	-1.1541390	-5.9126410	-1.4600540
C	-3.7100610	-1.0101450	-2.0873420	C	-1.5519490	-5.1724860	-0.1778690
C	-3.5383020	-1.1814630	-0.5497840	C	-0.9862710	-3.7428800	-0.3815360
C	-4.8970370	-1.7169980	-0.0136050	C	-0.7686760	-3.5751230	-1.9163960
C	-5.8753830	-1.4977660	-1.1751950	C	-1.3448020	-4.8522270	-2.5463170
P	-2.1280750	-2.2343400	-0.0970050	P	-1.9697660	-2.4121810	0.3981510
C	-2.1554270	-2.4819760	1.7003510	C	-3.6812160	-2.4707820	-0.2291710
C	-2.1890330	-1.1440880	2.4855700	C	-3.8324500	-2.0199020	-1.7062140
C	-1.3488270	-1.3916960	3.7632210	C	-5.1984140	-1.2894260	-1.7829060
C	-0.9995550	-2.8883430	3.7539400	C	-5.8503560	-1.4740940	-0.4049800
C	-0.9517390	-3.2544490	2.2686180	C	-4.6599850	-1.5717750	0.5500120
N	-0.7149850	-1.5222560	-0.5325550	N	-1.4070860	-0.9545900	0.0830300
P	-0.0812740	-0.1208490	-0.5640890	P	-0.0940380	-0.0911660	0.4175010
N	-0.7909890	1.1771440	-0.1742700	N	-0.2242540	1.4350260	-0.0324920
P	-0.8898450	2.7815860	0.0831400	P	-1.5440760	2.3230360	-0.2048470
C	0.0225780	3.1796710	1.6036380	C	-0.9539390	4.0013110	-0.5941900
C	-0.3413370	2.3082870	2.8456520	C	0.0208280	4.0408480	-1.8037490
C	-0.6659870	3.3136350	3.9635640	C	1.0992920	5.0925890	-1.4377880
C	0.0975510	4.5789550	3.5688140	C	0.6127180	5.7565510	-0.1418540
C	-0.1114420	4.6568210	2.0529100	C	-0.1620600	4.6417760	0.5629080
C	1.4803090	-0.1225450	-1.4403620	C	1.3755750	-0.7826510	-0.4153950
C	2.5360470	0.9879740	-1.2559420	C	2.1953960	0.0432420	-1.4291470
C	3.5976820	0.5024810	-2.2688350	C	3.0732310	-1.0802220	-2.0227280
C	3.4025270	-1.0352470	-2.4584620	C	3.1831400	-2.2035670	-0.9410070
C	2.2206330	-1.4666670	-1.5688990	C	2.3642990	-1.7372500	0.2819100
C	5.0068030	0.7092810	-1.6827960	C	4.4990670	-0.5616260	-2.2776750
C	4.8232830	1.1110620	-0.1858850	C	4.5855920	0.8510750	-1.6178920
C	3.2857550	1.2868780	0.0827340	C	3.1560700	1.2270880	-1.0792580
C	5.4325610	0.3268610	1.0211540	C	5.6313110	1.2457400	-0.5254500
C	4.2560570	-0.0764750	1.9309800	C	4.8304660	1.7319510	0.6976860
C	2.9672840	0.6219210	1.4558220	C	3.3399080	1.8659970	0.3315980
C	4.6934870	-1.7167980	-1.9689110	C	4.6699080	-2.3261360	-0.5669340
C	5.7222990	-0.5841400	-2.0960010	C	5.3625430	-1.7410500	-1.8066240
C	4.3554520	-2.3946390	-0.6018740	C	4.8264430	-1.6960840	0.8536330
C	2.8157010	-2.2232340	-0.3420640	C	3.3943380	-1.3109500	1.3798440
C	2.6287590	-1.8071710	1.1513570	C	3.4966830	0.1030880	2.0362330
C	4.0400090	-1.6117060	1.7445170	C	4.9303700	0.6125030	1.7826050
C	5.1014530	-2.0966720	0.7372770	C	5.7944220	-0.5159960	1.1876050
C	6.1750910	-1.0052050	0.8448900	C	6.5891590	0.2218910	0.1006150
C	1.9454330	-0.5049490	1.5751690	C	2.6498510	1.2863510	1.5636830
C	-2.1509850	-3.8129790	-0.9945130	C	-2.0069080	-2.8170090	2.1830720
C	-0.7750590	-4.5555060	-0.9541930	C	-0.6145300	-3.1864830	2.7511340
C	-1.1117930	-6.0097350	-0.5745990	C	-0.7174920	-2.8036990	4.2285570
C	-2.5863670	-6.1800630	-0.9473460	C	-1.4639900	-1.4684640	4.1845990
C	-3.2078250	-4.8434750	-0.5307460	C	-2.5253270	-1.6507400	3.0804030
C	-0.1399450	3.7089970	-1.2943380	C	-2.6339770	1.6880900	-1.5237740
C	-0.5601590	5.1947730	-1.3967820	C	-1.8896260	1.2314430	-2.7965120
C	-0.2514280	5.5245210	-2.8598020	C	-2.9858100	1.2644190	-3.8638470
C	-0.7580650	4.2903720	-3.6112550	C	-3.7414620	2.5587240	-3.5441180
C	-0.3950030	3.0945440	-2.7060700	C	-3.7781780	2.6276470	-2.0013940
C	-2.6587920	3.1735460	0.2103340	C	-2.5848300	2.3512300	1.3000240
C	-3.4314690	2.8741290	-1.0882900	C	-1.7774040	2.4106020	2.6324270
C	-4.8882130	2.8999980	-0.6206210	C	-2.4812220	3.4801560	3.4914170
C	-4.8669540	2.2292040	0.7646370	C	-3.8884730	3.5941470	2.8969750
C	-3.4210520	2.3880510	1.3038100	C	-3.6408230	3.4745440	1.3899890
H	2.1006480	1.9277670	-1.6007380	H	1.4953120	0.4175510	-2.1801050
H	1.5657530	-2.1600120	-2.0991310	H	1.8009360	-2.5820210	0.6916340
H	5.4990390	1.5492340	-2.1799000	H	4.6642980	-0.4241240	-3.3503250
H	4.9781550	-2.5166710	-2.6575700	H	4.9528010	-3.3794530	-0.4820130
H	1.0845290	0.0601080	-2.4555450	H	0.8297830	-1.4636020	-1.0866320
H	6.6859280	-0.7612100	-1.6288670	H	6.4260890	-1.5442320	-1.7100340
H	5.9312500	-0.4856070	-3.1688620	H	5.2775510	-2.5078240	-2.5880210
H	6.9486080	-1.0230660	0.0831740	H	7.1414080	-0.4040050	-0.5944580
H	6.6962140	-1.1861150	1.7937000	H	7.3504090	0.8087300	0.6315910
H	1.6942490	-0.6090210	2.6377010	H	2.7226710	2.0497280	2.3499660
H	5.5366100	-3.0412600	1.0747020	H	6.5014710	-0.8875500	1.9353710
H	2.1236030	-2.6374870	1.6506690	H	3.3262680	-0.0360680	3.1078960
H	4.1356000	-2.1240980	2.7047120	H	5.3744950	1.0138840	2.6978310
H	4.4631860	0.1513840	2.9791640	H	5.2301520	2.6719120	1.0886140
H	6.0939840	1.0212420	1.5463090	H	6.2285860	2.0642720	-0.9383470
H	2.6901770	1.4213160	2.1490560	H	3.0573160	2.9197910	0.2426610
H	3.4790400	1.0170080	-3.2252240	H	2.6225150	-1.4840280	-2.9338160
H	3.2000900	-1.2602880	-3.5081330	H	2.7886020	-3.1449310	-1.3351110
H	2.3652610	-3.2163050	-0.4028040	H	3.1758600	-1.9808170	2.2154840

H	4.5223460	-3.4595280	-0.7783830	H	5.1752300	-2.5211700	1.4797760
H	5.2641340	2.1076670	-0.1137560	H	4.7928000	1.5285050	-2.4503110
H	3.1159640	2.3544940	0.2448300	H	2.7885900	2.0475530	-1.7014740
H	-0.9892210	-6.1554540	0.5044130	H	0.2591220	-2.7357620	4.7140840
H	-0.4575100	-6.7254700	-1.0769320	H	-1.3107310	-3.5528510	4.7654380
H	-2.6920720	-6.3152430	-2.0295600	H	-1.9088730	-1.1907200	5.1429210
H	-3.0612470	-7.0306930	-0.4533930	H	-0.7640190	-0.6712580	3.9071640
H	-4.1868730	-4.6644050	-0.9803220	H	-3.4925870	-1.9209410	3.5099150
H	-3.3310990	-4.8306780	0.5578450	H	-2.6733880	-0.7260650	2.5161700
H	-0.0634440	-4.1055670	-0.2583110	H	0.1751100	-2.5830570	2.2855380
H	-0.3167640	-4.4975590	-1.9436210	H	-0.3633230	-4.2367140	2.5862730
H	-1.8876900	-1.0981660	4.6659610	H	-5.8233040	-1.6696300	-2.5940980
H	-3.2181390	-0.8537160	2.7074070	H	-3.7852510	-2.8783600	-2.3797100
H	-1.7471080	-0.3321490	1.9005640	H	-3.0233730	-1.3400220	-1.9863150
H	-6.2329110	-0.4615680	-1.1729310	H	-0.8573250	-5.1055110	-3.4907180
H	-1.0146550	-4.3292320	2.0837480	H	-4.9212400	-1.9768250	1.5301660
H	-6.7457890	-2.1541320	-1.1116690	H	-2.4147330	-4.7252720	-2.7496140
H	-1.7889580	-3.4663840	4.2458660	H	-6.4197640	-2.4097710	-0.3738570
H	-4.8477740	-2.7859210	0.2168260	H	-1.2141950	-2.6642250	-2.3223790
H	-0.0221590	-2.8856640	1.8221800	H	-4.2206470	-0.5776490	0.6977570
H	-5.1920030	-1.2094500	0.9073710	H	0.3058650	-3.5131690	-2.1153690
H	-4.8294740	-2.8184640	-2.5350770	H	-1.7494370	-6.8141230	-1.6246120
H	-3.8234180	0.0544740	-2.3087800	H	-1.1653470	-5.6396600	0.7307070
H	-5.4712930	-1.3823620	-3.3394670	H	-0.1001010	-6.2092330	-1.4083440
H	-2.8547640	-1.3596610	-2.6725330	H	-2.6444760	-5.1634910	-0.0978070
H	-0.7284730	6.4511810	-3.1863330	H	-2.5865520	1.2431120	-4.8810720
H	0.8303380	5.6358300	-2.9935770	H	-3.6480200	0.3978890	-3.7481180
H	-1.6332920	5.3201860	-1.2196730	H	-1.0965390	1.9339210	-3.0694490
H	-3.1677710	1.8744620	-1.4526530	H	-0.7201260	2.6390850	2.4787730
H	-3.2241780	3.5900110	-1.8870390	H	-1.8151270	1.4323590	3.1207390
H	-5.5988920	2.6746700	1.4408140	H	-4.5110980	2.7579370	3.2362170
H	-5.2202270	3.9395110	-0.5352650	H	-2.4840980	3.2218340	4.5530000
H	-5.5555170	2.3960020	-1.3236270	H	-1.9644690	4.4412910	3.3910720
H	-5.1191440	1.1682090	0.6793580	H	-4.3965060	4.5228400	3.1688100
H	-3.3834740	2.9084400	2.2625760	H	-4.5416220	3.2442420	0.8165660
H	-2.9723190	1.4033820	1.4529200	H	-3.2408510	4.4234620	1.0142340
H	-1.8450500	4.3537370	-3.7292600	H	-3.1781180	3.4118910	-3.9377050
H	-1.1826100	2.3374970	-2.7104680	H	-3.6677260	3.6577070	-1.6514800
H	-0.3272530	4.1911970	-4.6096030	H	-4.7409150	2.5938190	-3.9843170
H	-1.1667780	1.6173720	2.6698550	H	0.4899750	3.0648790	-1.9538010
H	0.5260180	1.6984030	3.1114870	H	-0.5170280	4.2976950	-2.7195990
H	-0.3906500	2.9324760	4.9491040	H	1.2607080	5.8149400	-2.2413440
H	-1.7410840	3.5240810	3.9786700	H	2.0551290	4.5892770	-1.2574190
H	-0.2572460	5.4752060	4.0822870	H	1.4307810	6.1504210	0.4666020
H	0.6099010	5.3024260	1.5487930	H	-0.8058380	5.0007480	1.3704500
H	-1.1133180	5.0497540	1.8478680	H	0.5414240	3.9112390	0.9800990
H	0.5162350	2.6018560	-3.0549730	H	-4.7343000	2.2684680	-1.6106410
H	1.1644360	4.4618090	3.7901160	H	-0.0649240	6.5875310	-0.3687850
H	-0.4342170	-0.7924070	3.7246550	H	-5.0408960	-0.2227700	-1.9738380
H	-0.0590700	-3.1054730	4.2652270	H	-6.5324750	-0.6605960	-0.1459700
H	-0.0276640	5.8282160	-0.6853960	H	-1.4278840	0.2504300	-2.6579130
H	1.0676620	2.9908010	1.3328930	H	-1.8277360	4.6257640	-0.8083060
H	-2.7234640	4.2468040	0.4212980	H	-3.1095620	1.3888390	1.2571360
H	0.9350950	3.6806960	-1.0824730	H	-3.0663730	0.7932230	-1.0618020
H	-3.3015010	-0.2156990	-0.0907340	H	-0.0145860	-3.6760160	0.1195300
H	-2.3656340	-3.5324600	-2.0314030	H	-2.6698860	-3.6835310	2.2871910
H	-3.0718410	-3.0441700	1.9115180	H	-4.0199440	-3.5078960	-0.1274340
H	0.9634680	-0.2886150	1.1329370	H	0.1438610	-0.1865480	1.8094090
				H	1.5899650	1.1178970	1.4469240
**III-Pf$_2$ (dication)**				**III-Pf$_2$ (protonated)**			
C	-1.4620930	-0.0415200	-1.7453080	C	-2.1750590	1.2215570	-1.8518270
C	-2.3051290	-1.2945710	-1.4997010	C	-2.9237410	-0.0842950	-2.1456360
C	-3.6683820	-0.8634800	-2.0805580	C	-4.3979630	0.3607620	-2.0365010
C	-3.6966360	0.6974660	-2.0767740	C	-4.4222910	1.6507800	-1.1571080
C	-2.3509650	1.1757520	-1.4981290	C	-2.9628320	1.9547320	-0.7726520
C	-2.5797950	-1.8521330	-0.0681940	C	-2.7746110	-1.3684670	-1.2653690
C	-4.1350730	-1.8734630	0.1495770	C	-4.2022120	-1.7494040	-0.7251870
C	-4.8063220	-1.3341610	-1.1529320	C	-5.2198310	-0.7085480	-1.2897870
C	-1.9293110	-1.3179040	1.2456360	C	-1.7707160	-1.5484480	-0.0823730
C	-3.0816080	-0.8675260	2.1692950	C	-2.6065010	-1.8413520	1.1785190
C	-4.4283630	-1.3326440	1.5851850	C	-4.0687260	-2.1158630	0.7886700
C	-1.0472170	-0.0588110	1.3023800	C	-0.8642870	-0.4235120	0.4482160
C	-1.9636710	1.1781550	1.2455410	C	-1.7692300	0.5179360	1.2774590
C	-3.1013740	0.6955820	2.1710880	C	-2.5979730	-0.5403130	2.0437110
C	-4.4607540	1.1296440	1.5924760	C	-4.0604910	-0.0796190	2.1748990
C	-4.1870310	1.6852110	0.1590430	C	-4.2135670	1.1893150	1.2803020
C	-2.6317190	1.7117250	-0.0615750	C	-2.7913200	1.5661720	0.7250620
C	-4.8456510	1.1274150	-1.1441380	C	-5.2390210	1.3194410	0.1065640
C	-5.3274860	-0.1136620	1.8351110	C	-4.8379870	-1.3728870	1.8913590
C	-5.7427720	-0.1181670	-1.1424210	C	-6.1042210	0.1425970	-0.3679640
P	0.5413610	-0.0376610	0.4836580	P	0.6108090	0.0825690	-0.5098120
N	1.1625130	-1.4209590	0.3122990	N	0.8644030	1.6473690	-0.3361890
P	2.2802800	-2.5345420	-0.0726320	P	2.0658720	2.5795110	0.1507980
C	3.9104780	-1.7625690	-0.4183550	C	1.8759410	4.1741480	-0.7427920
C	4.8638300	-2.6602490	-1.2186800	C	1.6282810	3.9761690	-2.2424090
N	1.1414610	1.3484450	0.3119880	N	1.8512070	-0.8350870	-0.0852780

P	2.1129650	2.6216450	0.0784510	P	2.4349000	-2.3098440	-0.1979060
C	3.6378990	2.0912990	-0.7894940	C	2.2713940	-3.0551800	-1.8745600
C	3.3654080	1.5222280	-2.1877270	C	0.8197600	-3.3966490	-2.2329120
C	2.5666900	3.2626840	1.7339570	C	1.5711760	-3.4371890	0.9774210
C	3.3775810	2.2266140	2.5218470	C	1.5016770	-2.8765030	2.4045320
C	1.0915340	3.8708030	-0.7883700	C	4.2393470	-2.1530310	0.1300710
C	0.6009770	3.3917530	-2.1600280	C	5.0899840	-3.3637250	-0.2675560
C	1.3195730	3.7043400	2.5093680	C	2.0720100	-4.8848090	0.9620050
C	1.7466360	5.2544570	-0.8610450	C	4.5257220	-1.7087040	1.5697300
C	4.7345830	3.1624530	-0.8141280	C	2.8947870	-2.1249930	-2.9239760
C	2.2829400	-3.7265620	1.3185880	C	1.9107440	2.9423990	1.9498480
C	3.3490750	-4.8150040	1.1484930	C	0.6035900	3.6856860	2.2499500
C	1.7012820	-3.3739300	-1.5941000	C	3.7210920	1.8020390	-0.0858530
C	0.4008010	-4.1451730	-1.3427250	C	4.0580940	1.6125890	-1.5685630
C	2.3451250	-3.0544260	2.6954610	C	2.0020420	1.6368480	2.7502070
C	1.5453230	-2.3533840	-2.7293780	C	4.8747320	2.4529670	0.6845810
C	4.5779630	-1.2150810	0.8483960	C	3.0037250	5.1735870	-0.4689430
H	-1.4276470	2.0135010	1.7003570	H	-1.1185140	1.0536580	1.9732590
H	-1.3689000	-2.1402230	1.6955710	H	-1.1482850	-2.4154930	-0.3240400
H	-4.8714270	1.9548110	2.1804990	H	-4.2685230	0.2268140	3.2042130
H	-4.8189390	-2.1718700	2.1669380	H	-4.2896420	-3.1834880	0.8780800
H	-0.6694930	-0.0524780	2.3410890	H	-0.3094700	-0.9330230	1.2506950
H	-6.2913940	-0.1243730	1.3359250	H	-5.9066910	-1.2592970	1.7357280
H	-5.5541150	-0.1202750	2.9089750	H	-4.7384250	-1.9903620	2.7938260
H	-6.5347220	-0.1347900	-0.3996690	H	-6.6443760	-0.3976920	0.4041210
H	-6.2539840	-0.1221640	-2.1136950	H	-6.8778880	0.5808290	-1.0122490
H	-1.1949300	-0.0366600	-2.8090960	H	-2.2482060	1.8264660	-2.7650720
H	-5.3391990	-2.1745130	-1.6063210	H	-5.8687980	-1.2448080	-1.9885720
H	-1.8801840	-2.1124070	-2.0883050	H	-2.6933600	-0.3839910	-3.1721220
H	-3.7990840	-1.2406880	-3.0977010	H	-4.8204860	0.5699640	-3.0231690
H	-3.8434090	1.0739110	-3.0920340	H	-4.8682560	2.4790200	-1.7148340
H	-5.4058500	1.9525630	-1.5924860	H	-5.9035670	2.1514090	0.3581570
H	-1.9659210	2.0131410	-2.0833700	H	-2.7563340	3.0259120	-0.8543110
H	-2.9574940	1.0703850	3.1872560	H	-2.1517050	-0.7458140	3.0206830
H	-2.9318710	-1.2412410	3.1849830	H	-2.1756250	-2.6720220	1.7451070
H	-2.2587940	-2.8951660	-0.1135880	H	-2.4892540	-2.1488270	-1.9760700
H	-4.4257690	-2.9255410	0.1915180	H	-4.4756430	-2.6910470	-1.2078070
H	-4.5107480	2.7275310	0.2037360	H	-4.4964840	1.9832980	1.9760850
H	-2.3307040	2.7611500	-0.0986350	H	-2.4980610	2.4963530	1.2193700
H	1.6291180	4.0800830	3.4873270	H	3.0640810	-4.9659010	1.4143740
H	0.7714050	4.5043600	2.0066690	H	1.3919820	-5.5049560	1.5524060
H	0.6407850	2.8621860	2.6716380	H	2.1153280	-5.3082730	-0.0448720
H	3.1975070	4.1379260	1.5406160	H	0.5492800	-3.4243750	0.5762790
H	4.3120240	1.9536460	2.0252650	H	1.3144570	-1.7998570	2.4171700
H	2.7919650	1.3188690	2.6979390	H	2.4274340	-3.0648960	2.9525510
H	3.6351850	2.6486220	3.4958800	H	0.6891070	-3.3708430	2.9438040
H	0.2209480	3.9258560	-0.1230440	H	4.4979320	-1.3175000	-0.5329300
H	1.0193320	5.9592190	-1.2714580	H	5.5394680	-1.3033220	1.6268660
H	2.0537350	5.6305620	0.1177340	H	3.8304330	-0.9360530	1.9080070
H	2.6151060	5.2550890	-1.5236560	H	4.4667160	-2.5527450	2.2619340
H	0.2692160	2.3509070	-2.1436600	H	6.1408130	-3.1355170	-0.0685830
H	-0.2495700	4.0085290	-2.4607540	H	4.8323920	-4.2542340	0.3106500
H	1.3749790	3.4989880	-2.9221570	H	5.0031960	-3.6065480	-1.3290760
H	3.9743630	1.2708000	-0.1440720	H	2.8448770	-3.9887600	-1.8456510
H	4.4543750	4.0124840	-1.4402080	H	3.9491480	-1.9148590	-2.7262210
H	4.9874620	3.5316170	0.1822940	H	2.8280340	-2.5936730	-3.9092680
H	5.6385210	2.7216640	-1.2416470	H	2.3620700	-1.1706240	-2.9679270
H	2.4922340	0.8640150	-2.2173860	H	0.3758520	-4.1222820	-1.5466830
H	3.2165600	2.3218340	-2.9159100	H	0.1863640	-2.5044430	-2.2522690
H	3.6305910	-0.9184820	-1.0588110	H	0.9475210	4.5699240	-0.3139200
H	1.5780110	-2.2849970	2.8067780	H	1.9089720	1.8551100	3.8171550
H	2.1668060	-3.8181520	3.4565820	H	1.1933800	0.9523630	2.4750330
H	3.3218610	-2.6095610	2.8939790	H	2.9515550	1.1170530	2.5978810
H	3.1781750	-5.5914560	1.8977290	H	0.4611100	3.7489280	3.3321810
H	5.1370950	-3.5643910	-0.6707120	H	2.5311240	3.6507370	-2.7646880
H	5.7816820	-2.0984390	-1.4091900	H	1.3163360	4.9288490	-2.6798560
H	4.4510220	-2.9507260	-2.1868160	H	0.8405130	3.2403460	-2.4182700
H	5.4148700	-0.5737720	0.5587640	H	3.2055110	5.3015550	0.5982880
H	4.9842060	-2.0227830	1.4622480	H	2.7246970	6.1507560	-0.8721500
H	2.4930940	-4.0848500	-1.8562010	H	3.5414440	0.8054260	0.3328020
H	0.0157720	-4.5105370	-2.2977080	H	4.3519760	2.5564070	-2.0369370
H	0.5495610	-5.0104140	-0.6934030	H	4.9027370	0.9230860	-1.6617830
H	-0.3605360	-3.4994050	-0.8970580	H	3.2197170	1.1914530	-2.1300060
H	1.2590520	-2.8801180	-3.6423280	H	5.7778060	1.8552730	0.5286550
H	2.4677470	-1.8054870	-2.9391250	H	4.6909160	2.4900040	1.7608830
H	0.7576760	-1.6306190	-2.4994700	H	5.0875630	3.4669830	0.3387040
H	3.3150330	-5.2927330	0.1655980	H	0.6088420	4.7056910	1.8582960
H	4.3546100	-4.4176560	1.3099200	H	-0.2549670	3.1561790	1.8250980
H	1.2916250	-4.1850710	1.2252280	H	2.7555170	3.5856030	2.2212450
H	3.8956800	-0.6236070	1.4644680	H	3.9311900	4.8683540	-0.9618200
H	4.2363810	0.9446090	-2.5089350	H	0.7935080	-3.8344130	-3.2343970
H	-0.4756680	-0.0162330	-1.2631420	H	0.3146920	-0.1761940	-1.8648000
				H	-1.1151480	1.1554080	-1.6519830
**III-Pg$_2$ (dication)**				**III-Pg$_2$ (protonated)**			
C	0.9236710	0.1108560	-1.2334900	C	0.6494450	-0.1515140	0.5345660
C	1.8524510	1.2834330	-0.8403990	C	1.4044130	-1.4869970	0.5395800

C	2.9811560	1.0634050	-1.8720930	C	2.2578110	-1.3183890	1.8094820
C	2.9548410	-0.4425360	-2.2881380	C	2.4134460	0.2182650	2.0533970
C	1.8016920	-1.1173220	-1.5184310	C	1.6754080	0.9490350	0.9036810
C	2.5372310	1.4641650	0.5538330	C	2.3714500	-1.8987640	-0.6131910
C	4.0904880	1.4825180	0.3196380	C	3.7728200	-2.1848750	0.0404850
C	4.3475110	1.3241690	-1.2112240	C	3.6680430	-1.8832820	1.5722000
C	2.2646860	0.5855830	1.8099210	C	2.6112070	-1.0886660	-1.9337980
C	3.6039350	-0.0507300	2.2236400	C	4.1312290	-0.8213580	-2.0158460
C	4.7523310	0.5918990	1.4224120	C	4.8665510	-1.5886790	-0.8986060
C	1.3536330	-0.6329130	1.7268150	C	2.0319630	0.3108390	-2.2054780
C	2.1699570	-1.7925630	1.1455910	C	2.9526020	1.3129330	-1.5256390
C	3.5426740	-1.5562790	1.8135010	C	4.3465540	0.7000320	-1.7401230
C	4.6729540	-1.7863590	0.7900720	C	5.1696060	0.8119740	-0.4426690
C	3.9955450	-1.9538970	-0.6055620	C	4.1812300	1.2938870	0.6714410
C	2.4407170	-1.9725970	-0.3831160	C	2.7898400	1.5752220	-0.0029580
C	4.2970970	-1.0544500	-1.8480840	C	3.9206820	0.5284270	2.0042370
C	5.6289200	-0.6239450	1.0891520	C	5.8781070	-0.5502330	-0.3951910
C	5.2042720	0.1824470	-1.7748040	C	4.5501220	-0.8438980	2.2807940
P	-0.6534870	-0.1352440	-0.4281130	P	-0.8025330	-0.0190940	-0.5626340
N	-1.4838180	1.0601970	0.0371360	N	-1.9917590	-0.7934780	0.1960080
P	-1.8340340	2.6401420	0.0731660	P	-2.6012430	-2.2320900	-0.0423270
N	-3.3545480	2.7793550	-0.5233370	N	-1.9974440	-3.2755260	1.1125420
C	-4.3878540	1.8169470	-0.1485050	C	-1.5933460	-2.7694560	2.4198660
N	-1.1372130	-1.5943660	-0.4452870	N	-1.1110830	1.4941490	-0.9798140
P	-2.4779760	-2.4437430	-0.0601800	P	-1.7145050	2.7031630	-0.1491280
N	-3.4496270	-1.6345710	0.9886070	N	-1.3459070	2.7842670	1.4701730
C	-2.8736780	-1.0878360	2.2189980	C	-0.5544730	3.8425420	2.0780760
N	-3.3861410	-2.7221210	-1.4047410	N	-1.1448510	4.0727000	-0.8888750
C	-3.2191940	-3.9636740	-2.1618890	C	-1.8178100	5.3548310	-0.7401650
N	-1.9034950	-3.8250330	0.6044500	N	-3.3727350	2.6537520	-0.1856300
C	-2.7678290	-4.6674090	1.4294580	C	-4.2273670	3.3248680	0.7853260
C	-3.7777740	-1.5692000	-2.2190820	C	0.2135160	4.1469350	-1.4127380
C	-0.6501390	-4.4531230	0.1881020	C	-4.0642480	2.2427880	-1.4021100
C	-4.9095720	-1.7336580	0.9978010	C	-1.8402930	1.7876830	2.4122090
N	-0.8790710	3.5765570	-0.8785990	N	-2.2775550	-3.0328840	-1.4617550
C	0.0347650	4.5955400	-0.3720020	C	-2.9635660	-2.6649410	-2.6975820
N	-1.7297280	3.0879240	1.6478780	N	-4.2565710	-2.0560210	-0.0453880
C	-0.7380430	2.5537800	2.5745970	C	-4.8838960	-0.8883420	0.5642070
C	-0.8801310	3.4135540	-2.3317270	C	-0.9942220	-3.6939930	-1.6874600
C	-2.4383240	4.2768640	2.1208350	C	-5.1121200	-3.2368760	-0.0853440
C	-3.8635560	3.9956770	-1.1558720	C	-2.3559970	-4.6906180	1.1305030
H	1.7248650	-2.7257530	1.4993870	H	2.8721560	2.2771440	-2.0373740
H	1.9012710	1.2477280	2.5992360	H	2.2986770	-1.7423510	-2.7535680
H	5.1923910	-2.7239750	1.0062610	H	5.9331950	1.5889840	-0.5436680
H	5.3292470	1.2636850	2.0638470	H	5.4205910	-2.4327970	-1.3198830
H	1.0816650	-0.9147120	2.7507830	H	2.1218680	0.4688590	-3.2877180
H	6.4183520	-0.4607440	0.3617390	H	6.3866600	-0.7964640	0.5325140
H	6.1429830	-0.8866600	2.0226300	H	6.6679340	-0.5017970	-1.1564230
H	6.1697560	0.0384000	-1.3001810	H	5.6185470	-0.9178500	2.1007670
H	5.4272160	0.4542050	-2.8144980	H	4.4217590	-1.0270570	3.3557760
H	0.5459900	0.4088840	-2.2276680	H	0.0667410	-0.2049620	1.4674760
H	4.7597620	2.2725380	-1.5658520	H	3.7843940	-2.8390480	2.0914540
H	1.3176060	2.2091320	-1.0526330	H	0.6800030	-2.2977560	0.6736930
H	2.8294490	1.6974130	-2.7491120	H	1.7726270	-1.7883590	2.6697310
H	2.8047770	-0.5303320	-3.3666940	H	1.9800710	0.4878840	3.0207720
H	4.6815730	-1.7141010	-2.6304960	H	4.1936160	1.2030900	2.8209500
H	1.2417330	-1.7907620	-2.1700710	H	1.0976820	1.7859650	1.3009260
H	3.6649810	-2.1915350	2.6940540	H	4.8633700	1.1673810	-2.5828740
H	3.7669410	0.0406680	3.3001770	H	4.5233500	-1.0835020	-3.0023480
H	2.2438420	2.4735580	0.8536580	H	1.9845130	-2.8605660	-0.9600830
H	4.4255360	2.4959070	0.5514990	H	3.9240090	-3.2661060	-0.0099750
H	4.2730120	-2.9601400	-0.9274370	H	4.5784870	2.2635260	0.9821580
H	2.1049300	-2.9896530	-0.5960980	H	2.6088240	2.6502160	0.0805790
H	-3.9179400	-0.6882340	-1.5904670	H	0.6141260	3.1412180	-1.5400950
H	-4.7271530	-1.7869670	-2.7137330	H	0.8761440	4.7145550	-0.7450450
H	-3.0199680	-1.3441710	-2.9790300	H	0.2008970	4.6458680	-2.3873470
H	-3.0379250	-4.8009430	-1.4884350	H	-1.3382560	5.9891570	0.0168650
H	-4.1450350	-4.1615450	-2.7067020	H	-1.7959660	5.8856890	-1.6970370
H	-2.3937930	-3.8931310	-2.8799860	H	-2.8615260	5.2102750	-0.4603740
H	-0.0603030	-3.7556030	-0.4048700	H	-4.3943320	3.1110480	-1.9865340
H	-0.8435800	-5.3546200	-0.4035920	H	-4.9448660	1.6500090	-1.1348300
H	-0.0781250	-4.7309760	1.0780470	H	-3.4060110	1.6262360	-2.0137490
H	-2.2073790	-4.9989770	2.3075900	H	-3.6350700	3.7271390	1.6072310
H	-3.6396560	-4.1088170	1.7704450	H	-4.7788660	4.1507950	0.3188320
H	-3.1100410	-5.5480280	0.8749130	H	-4.9547270	2.6138880	1.1930850
H	-5.2641550	-2.1946510	0.0776770	H	-0.9995130	1.3245090	2.9443430
H	-5.2487120	-2.3336060	1.8495110	H	-2.5017650	2.2505230	3.1547880
H	-5.3420770	-0.7327890	1.0863040	H	-2.3902870	1.0015330	1.8917250
H	-3.1295530	-0.0284980	2.3150120	H	-0.1145790	4.4867320	1.3186760
H	-3.2561340	-1.6320010	3.0884000	H	-1.1713310	4.4570180	2.7456040
H	-1.7866450	-1.1819210	2.2189240	H	0.2617550	3.4078330	2.6671400
H	0.1161700	3.1237310	-2.6836170	H	-1.1583890	-4.6487010	-2.1975850
H	-1.5960830	2.6475490	-2.6344180	H	-0.3373870	-3.0769420	-2.3149430
H	-1.1586010	4.3547650	-2.8161920	H	-0.4915260	-3.8872690	-0.7400660
H	0.0345290	4.6084800	0.7167240	H	-3.9078640	-2.1710470	-2.4715220
H	1.0548780	4.3892200	-0.7135860	H	-2.3475790	-1.9913880	-3.3072050
H	-0.2634020	5.5834860	-0.7367710	H	-3.1688680	-3.5685500	-3.2802490

H	-2.9131470	4.0488180	3.0787780
H	-3.2178190	4.5668130	1.4161580
H	-1.7556280	5.1228440	2.2564580
H	-0.3725960	1.5911650	2.2229030
H	0.1123610	3.2352380	2.6963940
H	-1.2096940	2.4027100	3.5490840
H	-4.2780160	3.7521120	-2.1385630
H	-4.6535280	4.4406350	-0.5413310
H	-3.0681220	4.7295290	-1.2810070
H	-5.0309330	2.2179250	0.6427030
H	-3.9264790	0.8917830	0.1982870
H	-5.0039080	1.5935320	-1.0243760
H	0.3716650	-0.4455950	1.2742990

H	-4.6294360	-4.0394620	-0.6453050
H	-6.0451660	-2.9843110	-0.5981020
H	-5.3570790	-3.6016410	0.9206600
H	-4.2130820	-0.0316150	0.4992660
H	-5.8076340	-0.6596630	0.0223990
H	-5.1377360	-1.0662680	1.6177970
H	-1.4964610	-5.2723590	1.4790240
H	-3.2013820	-4.8860510	1.8022330
H	-2.6151090	-5.0354600	0.1291290
H	-1.3502870	-1.7098180	2.3533570
H	-0.7050670	-3.3135380	2.7585950
H	-2.3881630	-2.9035350	3.1650240
H	-0.5251950	-0.7042550	-1.7567980
H	0.9858170	0.4787460	-1.9882380

III-Ph$_2$ (dication)

C	3.3398670	2.7706160	-1.9580120
N	2.1149990	2.1576380	-1.3888640
C	1.1397860	1.8669830	-2.4715010
C	1.8833980	2.2397830	-3.7591360
C	2.8783790	3.3101440	-3.3070560
P	1.5404880	2.5410120	0.1066760
N	2.8529620	2.4838700	1.0793910
C	3.8677660	1.4070980	0.9706200
C	4.4712780	1.3133800	2.3786050
C	3.3610540	1.8386490	3.2910580
C	2.7777870	2.9858350	2.4717380
N	0.3101800	1.5304930	0.4521790
P	-0.0776810	0.0440230	0.4227060
N	0.7375570	-1.1701630	0.0005940
P	1.8682460	-2.3219210	0.0129650
N	1.3061150	-3.4780940	-0.9849650
C	-0.0934710	-3.6794680	-1.3972920
C	0.0270860	-4.7479320	-2.4825900
C	1.2014260	-5.6031860	-1.9992180
C	2.1877340	-4.5704660	-1.4475330
C	-1.6388620	-0.2876610	1.2265340
C	-2.6162310	0.8752660	1.4510210
C	-3.7194700	0.1478020	2.2448320
C	-3.6193540	-1.3738350	1.9033740
C	-2.4613150	-1.5524100	0.8950060
C	-5.1019590	0.6281720	1.7664270
C	-4.8607690	1.4903440	0.4848520
C	-3.3104370	1.6283170	0.2767140
C	-5.9057350	-0.6798940	1.7445850
C	-4.9527120	-1.7726730	1.2428430
C	-4.6633180	-1.9785830	-0.2763900
C	-3.1142910	-1.8462330	-0.4942310
C	-3.0065650	1.4042870	-1.2391050
C	-4.3441940	1.0202260	-1.9087450
C	-5.5032420	1.2022730	-0.9082610
C	-2.8892420	-1.0037750	-1.7848730
C	-4.2716290	-0.5033550	-2.2465460
C	-5.3771160	-1.1999860	-1.4290640
C	-2.0879080	0.2927730	-1.7537180
C	-6.3559280	-0.0477170	-1.1626020
N	3.3155560	-1.7348880	-0.4687260
C	3.4096800	-1.0279540	-1.7666710
C	4.9163080	-0.9434450	-2.0178860
C	5.4598760	-2.2016170	-1.3374190
C	4.6297110	-2.2816170	-0.0581990
N	2.1238680	-2.9335070	1.5113950
C	2.6817950	-2.0967570	2.5998150
C	1.7938760	-2.4213240	3.7999330
C	1.4212820	-3.8850250	3.5565550
C	1.1657520	-3.9296520	2.0502400
N	0.8543400	4.0091600	0.2985390
C	1.6087010	5.2596390	0.0771930
C	0.5102790	6.3193010	-0.0293690
C	-0.5955680	5.7662640	0.8719380
C	-0.5748750	4.2684320	0.5729800
H	-2.1179720	1.6190920	2.0759280
H	-1.8541960	-2.4214030	1.1601120
H	-5.5468820	1.2904320	2.5139320
H	-5.2927610	-2.7343040	1.6364530
H	-1.2407100	-0.5032720	2.2345730
H	-6.8736300	-0.6367460	1.2548580
H	-6.1193920	-0.9201760	2.7939890
H	-7.1377360	-0.2437570	-0.4348830
H	-6.8781500	0.1286730	-2.1117280
H	-1.8316130	0.5439030	-2.7897260
H	-5.8866970	-1.9465390	-2.0444340
H	-2.4517980	-1.6663410	-2.5366030
H	-4.4089480	-0.6586560	-3.3193670
H	-4.5079220	1.6001020	-2.8203060
H	-6.0950260	2.0827570	-1.1734160
H	-2.6393560	2.3555850	-1.6334880

III-Ph$_2$ (protonated)

C	0.3212410	-3.7443980	-1.9344460
N	1.6466270	-3.2812820	-1.4702020
C	2.3645260	-2.7494140	-2.6427620
C	1.2632630	-2.0997380	-3.4797100
C	0.0912750	-3.0784770	-3.3138090
P	1.7895690	-2.4071180	-0.0592780
N	0.8762480	-3.2954380	1.0047220
C	0.8585040	-4.7733040	1.0467100
C	0.9815640	-5.1003160	2.5332230
C	0.2285560	-3.9413180	3.1892370
C	0.6548290	-2.7327210	2.3536280
N	1.3680650	-0.8935260	-0.0668220
P	0.1424170	0.1158520	0.0857360
N	0.4241740	1.5765750	-0.5171520
P	1.5968380	2.5376140	-0.0218120
N	3.0625170	2.0365610	-0.5979010
C	3.1304040	1.4527560	-1.9557780
C	4.6235090	1.4832960	-2.2873300
C	5.1264350	2.7127850	-1.5274840
C	4.3619890	2.6167030	-0.2086860
C	-1.3583330	-0.5412320	-0.7066830
C	-2.3410290	0.2330790	-1.6206530
C	-3.2129800	-0.9511470	-2.1055580
C	-3.1325670	-2.0688100	-1.0161330
C	-2.1960820	-1.5514950	0.0912720
C	-4.6895640	-0.5270670	-2.2023840
C	-4.7948570	0.8872620	-1.5556650
C	-3.3398740	1.3693880	-1.2044680
C	-3.4059450	2.0509000	0.1928360
C	-4.8174640	1.8110710	0.7543970
C	-5.7262400	1.2329180	-0.3463660
C	-4.6814540	0.7147490	1.8583850
C	-3.1915760	0.3087750	1.9138290
C	-2.5108710	1.5505370	1.3159370
C	-6.5212770	0.1665920	0.4224900
C	-5.5426760	-0.4827670	1.4105540
C	-4.5587160	-1.6093090	0.9664930
C	-3.0970860	-1.1293260	1.2953450
C	-4.5520420	-2.2588370	-0.4564660
C	-5.4203860	-1.7415530	-1.6133660
N	3.4064020	-2.4043450	0.2619160
C	4.1995670	-1.2137610	0.5984300
C	5.6073520	-1.6249970	0.1779220
C	5.6473600	-3.1085370	0.5570290
C	4.2425250	-3.6132920	0.1943750
N	1.8655280	2.6796280	1.6100870
C	2.5545240	1.6218680	2.3853170
C	2.0060600	1.7803950	3.8084830
C	0.6126760	2.3772710	3.5987560
C	0.8334490	3.3427850	2.4360650
N	1.1488900	4.0333950	-0.5486900
C	1.9594400	5.2344280	-0.2975350
C	0.9386770	6.3716000	-0.3699270
C	-0.0732700	5.8555700	-1.3960120
C	-0.1939840	4.3692990	-1.0547440
H	-3.2250660	3.1198490	0.0435850
H	-2.8632390	0.2158710	2.9533390
H	-6.4294710	1.9941450	-0.6971580
H	-6.1164780	-0.8746960	2.2556100
H	-2.5278860	2.3125400	2.1070620
H	-7.1130950	-0.5185970	-0.1776160
H	-7.2467930	0.7193480	1.0338800
H	-6.4747270	-1.5992740	-1.3959770
H	-5.3843030	-2.5217740	-2.3851090
H	-0.8437800	-1.1754970	-1.4454020
H	-4.7647980	-3.3231330	-0.3201180
H	-1.5385320	-2.3551650	0.4398290
H	-2.7478690	-2.9941180	-1.4559610
H	-2.8470250	-1.3334480	-3.0629310
H	-4.9841140	-0.4241110	-3.2508430
H	-1.7487610	0.6295040	-2.4485830

H	-3.5891430	0.2996820	3.3189360
H	-3.4313570	-1.9504570	2.8121510
H	-2.7477830	-2.8428600	-0.7473260
H	-4.9117890	-3.0258390	-0.4625970
H	-5.2256070	2.4838000	0.7554160
H	-3.0591460	2.6765420	0.4489590
H	2.2141130	5.2051550	-0.8315110
H	2.2744800	5.4552280	0.9254390
H	0.8647890	7.3054290	0.2746200
H	0.1563230	6.3838800	-1.0629170
H	-0.3474540	5.9390320	1.9239350
H	-1.5740830	6.2063460	0.6724140
H	-1.1836790	4.0318510	-0.3072010
H	-0.9237790	3.6606720	1.4091390
H	1.7518160	3.2385740	2.7447110
H	3.3911090	3.8885130	2.5644550
H	2.5951290	1.0717100	3.4521700
H	3.7269660	2.1682590	4.2648330
H	5.3504220	1.9598420	2.4518290
H	4.7845290	0.2952570	2.6188400
H	3.4004700	0.4628600	0.6705950
H	3.7524890	3.5290000	-1.2906010
H	2.3721640	4.2725950	-3.1751790
H	4.6198910	1.6640240	0.2209960
H	0.2330370	2.4709110	-2.3499340
H	1.1970250	2.5833450	-4.5342670
H	0.8567900	0.8101290	-2.4563980
H	2.4224800	1.3684170	-4.1441010
H	0.1307450	-3.6371920	1.8322900
H	1.3472000	-4.9122560	1.6094830
H	0.8952940	-1.7954810	3.7783110
H	2.2616490	-4.5369500	3.8138790
H	2.3075780	-2.2554060	4.7481870
H	0.5480550	-4.2095070	4.1244360
H	3.7233240	-2.3807260	2.7822980
H	2.6598080	-1.0352530	2.3362730
H	2.9098100	-1.6053150	-2.5541230
H	2.9351680	-0.0469680	-1.6949870
H	5.3295450	-0.0516520	-1.5349890
H	5.1465260	-0.8900030	-3.0830090
H	6.5295860	-2.1502310	-1.1281140
H	5.2718720	-3.0816620	-1.9610660
H	5.0586860	-1.6485870	0.7268540
H	4.5331940	-3.2951840	0.3349370
H	-0.6990030	-4.0406970	-0.5564750
H	2.7915320	-4.9699470	-0.6269760
H	2.8632610	-4.2008700	-2.2279010
H	-0.5220910	-2.7444750	-1.7621510
H	1.6469890	-6.2113660	-2.7878360
H	0.2692070	-4.2776580	-3.4406800
H	0.8743550	-6.2689600	-1.1944890
H	-0.8979240	-5.3146540	-2.6002220
H	3.7078290	3.4472950	-4.0027850
H	4.1004620	1.9930140	-2.0951240
H	-1.0966720	0.2150820	-1.2900030

H	-5.2381780	2.7259090	1.1810180
H	-5.0172500	1.1081430	2.8218480
H	-2.7423710	-1.7490640	2.1235030
H	-4.7703010	-2.4429060	1.6408640
H	-5.1501340	1.5337730	-2.3621260
H	-3.0993790	2.1801080	-1.8965940
H	2.8318070	-3.5804510	-3.1842090
H	3.1544350	-2.0598210	-2.3355580
H	1.0169070	-1.1211460	-3.0527490
H	1.5514110	-1.9551120	-4.5229850
H	-0.8800190	-2.5800270	-3.3672380
H	0.1132330	-3.8349450	-4.1024210
H	0.3254790	-4.8352610	-2.0177520
H	-0.4492860	-3.4781710	-1.2072700
H	4.2154880	-4.0381170	-0.8157530
H	3.8965070	-4.3822970	0.8936380
H	6.4280670	-3.6692960	0.0397060
H	5.8143420	-3.2102680	1.6340230
H	6.3808990	-1.0329010	0.6716620
H	5.7210660	-1.5081820	-0.9057730
H	3.8118020	-0.3343360	0.0819220
H	1.5776990	-2.2881550	2.7497470
H	0.4640920	-3.8099100	4.2468420
H	4.1654550	-1.0124480	1.6781320
H	1.6598330	-5.1999940	0.4397890
H	2.0349220	-5.0844750	2.8339320
H	-0.0946300	-5.1388600	0.6453000
H	0.5676320	-6.0802510	2.7788920
H	2.7105460	0.4433980	-1.9505460
H	2.5617550	2.0614480	-2.6702340
H	6.2082900	2.7198550	-1.3802270
H	4.8027280	1.5320650	-3.3629580
H	4.8427610	3.6278720	-2.0586280
H	5.1125810	0.5820070	-1.9019570
H	4.2420260	3.5786950	0.2940040
H	4.8787030	1.9384990	0.4826730
H	2.3314610	0.6283240	1.9811310
H	3.6384640	1.7585140	2.3484480
H	2.6285920	2.4802890	4.3747120
H	1.9930530	0.8295880	4.3450250
H	0.2200290	2.8777690	4.4860710
H	-0.0988720	1.5968790	3.3052220
H	1.2056850	4.3114300	2.7905630
H	-0.0807290	3.5240950	1.8641960
H	2.4538150	5.1791040	0.6776550
H	-0.9569030	4.2035870	-0.2824700
H	-0.4538220	3.7524410	-1.9167430
H	2.7332760	5.3460060	-1.0681090
H	0.3257060	5.9756550	-2.4085960
H	1.3955760	7.3226190	-0.6501120
H	-1.0371610	6.3658560	-1.3461550
H	0.4551200	6.4954780	0.6048790
H	-0.8517520	-4.0955180	3.0959140
H	-0.1149360	-1.9548260	2.3335650
H	-0.1256800	0.1814580	1.4696390
H	-1.4721260	1.4749820	1.0291380

$I(Pa_2)_2$ (dication)

C	1.0735440	1.9932420	0.5414560
C	-0.0707880	2.7828340	1.2031690
C	0.3298430	4.2509240	0.9027990
C	0.7768440	4.1329310	-0.5754260
C	1.1002040	2.6329870	-0.8519020
C	-0.5732860	3.9877100	-1.3379100
C	-1.6242790	4.0398780	-0.2003650
C	-1.0037490	4.9942360	0.8201790
C	-1.4404590	2.6330790	0.4295110
C	-1.3249340	1.7360190	-0.8147800
C	-0.2587300	2.4884510	-1.6196740
H	-0.9012550	6.0162830	0.4426190
H	-1.5433160	5.0189280	1.7720270
H	1.9885380	2.4054500	-1.4415260
H	-2.2737140	1.7602100	-1.3664930
H	2.0063640	2.2201990	1.0746060
H	-2.6456590	4.2489200	-0.5234680
H	-2.2515370	2.3412370	1.0989610
H	-0.7653210	4.6450800	-2.1858330
H	1.4876670	4.8864850	-0.9143580
H	1.0831420	4.6507360	1.5839280
H	-0.1810020	2.5641380	2.2659530
H	-0.1734420	2.1693910	-2.6582220
P	0.9146160	0.1819540	0.6002960
N	0.8058320	-0.1858100	2.1520810
P	0.2376260	-1.4202310	2.9998970
C	-0.0293920	-2.9453900	2.0612170
N	2.0339360	-0.5609930	-0.2935300
P	3.6207510	-0.6774500	-0.0674140

$I(Pa_2)_2$ (protonated)

C	1.3368710	1.5244730	-0.5254270
C	0.6056880	2.6074520	0.2889760
C	0.8449540	3.8565530	-0.6113570
C	0.5333920	3.2577370	-2.0094700
C	0.6631180	1.7096780	-1.8816770
C	-1.0153000	3.1376980	-1.9946970
C	-1.3942200	3.7042900	-0.5977490
C	-0.3411230	4.7773270	-0.3395670
C	-0.9753330	2.5128040	0.3080940
C	-1.5619800	1.3419440	-0.5023750
C	-0.8986170	1.5817420	-1.8635220
H	-0.4038730	5.6177320	-1.0388650
H	-0.3640940	5.1640130	0.6845810
H	1.1484270	1.1765060	-2.7006520
H	-2.6190820	1.6011740	-0.6653860
H	2.3739410	1.8758110	-0.6303230
H	-2.4410640	3.9938710	-0.4821010
H	-1.3735310	2.5859830	1.3233790
H	-1.5809910	3.5450230	-2.8338630
H	1.0086910	3.7605020	-2.8531400
H	1.8432780	4.2861770	-0.5048550
H	1.0049520	2.7220880	1.2991820
H	-1.3245710	0.9842930	-2.6709110
P	1.5403250	-0.2100270	0.1102840
N	2.4573510	0.1350430	1.5075050
P	1.9907760	-0.3122630	2.9557610
C	0.7057170	0.7301570	3.7227550
N	2.6146230	-0.8224180	-1.0417160
P	4.1985330	-0.7788890	-0.9820200

C	4.1021270	-1.2401380	1.5848610
C	4.2712890	-1.8774350	-1.2469200
C	4.5089150	0.8755140	-0.3556100
C	1.4021990	-1.8212530	4.3217320
C	-1.3139010	-0.9742950	3.8088970
H	-1.7298420	-1.8250130	4.3532920
H	-1.1240530	-0.1535380	4.5040770
H	-2.0062420	-0.6420660	3.0342490
H	1.6061560	-0.9207160	4.9044200
H	0.9734780	-2.5863760	4.9730540
H	2.3358490	-2.1925560	3.8957430
H	-0.3823650	-3.7371860	2.7258770
H	-0.7738250	-2.7511090	1.2868100
H	0.9102480	-3.2534260	1.5970210
H	4.2830490	1.2397690	-1.3598890
H	4.1960610	1.6226800	0.3769890
H	5.5856150	0.7161470	-0.2626980
H	3.7042650	-0.5497160	2.3317040
H	3.6924210	-2.2372130	1.7590400
H	5.1908200	-1.2800880	1.6633710
H	4.0578420	-1.5452350	-2.2647200
H	5.3514860	-1.9743820	-1.1183050
H	3.7989380	-2.8467530	-1.0766140
P	-0.9749670	-0.0291830	-0.5450170
N	-0.8797570	-0.6916160	-1.9951400
P	0.0605740	-1.6293370	-2.8776990
C	1.4472360	-0.7215160	-3.6017250
N	-2.0261250	-0.6881250	0.4879130
P	-3.4870310	-1.2667000	0.1465830
C	-4.2873270	-1.7230830	1.6964330
C	-4.5766650	-0.0884980	-0.6948990
C	-3.4531950	-2.7520510	-0.8885640
C	0.7092150	-3.0780070	-2.0084360
C	-0.9118960	-2.2525410	-4.2665960
H	-1.7193900	-2.8885830	-3.8989400
H	-1.3375720	-1.4089410	-4.8131690
H	-0.2728720	-2.8339960	-4.9352390
H	1.2955670	-2.7289130	-1.1568370
H	-0.1286580	-3.6831650	-1.6544920
H	1.3345950	-3.6753850	-2.6761690
H	1.0509310	0.0411620	-4.2758380
H	1.9951480	-0.2395180	-2.7908020
H	2.1035380	-1.3938090	-4.1592440
H	-2.8213870	-3.5076050	-0.4164510
H	-4.4627520	-3.1484680	-1.0176670
H	-3.0339120	-2.4888690	-1.8611750
H	-3.6731030	-2.4612700	2.2162890
H	-4.4012110	-0.8375610	2.3247640
H	-5.2708090	-2.1510660	1.4916420
H	-4.6312110	0.8337070	-0.1122350
H	-4.1762740	0.1313010	-1.6877120
H	-5.5786330	-0.5108890	-0.8019770

C	4.9930540	0.8388670	-0.7002890
C	4.9481000	-1.8576360	0.2803200
C	4.8620750	-1.3717840	-2.5616400
C	1.3345310	-2.0074100	3.1088260
C	3.3962860	-0.2291550	4.0951020
H	4.1692020	-0.9279710	3.7677190
H	3.8056500	0.7831810	4.0866000
H	3.0840720	-0.4848650	5.1103990
H	2.0814700	-2.7147880	2.7405170
H	1.0959240	-2.2377390	4.1498760
H	0.4303600	-2.1063000	2.5030190
H	-0.2094620	0.6787490	3.1267620
H	0.4852890	0.4012350	4.7416850
H	1.0514360	1.7664890	3.7425790
H	4.5309420	-0.7101310	-3.3649220
H	5.9543390	-1.3905340	-2.5349890
H	4.4882680	-2.3797140	-2.7539610
H	4.6950390	1.5343120	-1.4886390
H	4.6491780	1.2191020	0.2643770
H	6.0822150	0.7421820	-0.6946060
H	4.6347190	-1.4943330	1.2607550
H	4.5819440	-2.8777070	0.1421220
H	6.0388980	-1.8492130	0.2102830
P	-1.8496890	-0.2805730	0.2941620
N	-2.0029860	-1.4293630	-0.8157640
P	-1.1251280	-2.5324470	-1.5558680
C	-0.0877050	-3.5405940	-0.4650910
N	-3.1758810	-0.0997060	1.2112920
P	-4.6859630	-0.4166890	0.7920520
C	-5.0412220	-2.1838260	0.5998830
C	-5.7751420	0.1992840	2.0928660
C	-5.2334090	0.3520490	-0.7598540
C	-2.2739920	-3.6731370	-2.3638480
C	-0.0352300	-1.9099240	-2.8598930
H	0.8147710	-1.3832560	-2.4128240
H	0.3388990	-2.7514720	-3.4495020
H	-0.6017130	-1.2394740	-3.5099020
H	-2.8979250	-4.1539260	-1.6074610
H	-2.9105530	-3.1139380	-3.0527320
H	-1.7232390	-4.4373340	-2.9167820
H	0.6952040	-2.9022370	-0.0491160
H	-0.7008030	-3.9510620	0.3401930
H	0.3639560	-4.3576350	-1.0332220
H	-5.1305250	1.4373660	-0.6899280
H	-4.6085490	-0.0185950	-1.5764770
H	-6.2774230	0.1008830	-0.9617610
H	-4.4241450	-2.5688070	-0.2143190
H	-4.7829980	-2.7055560	1.5237340
H	-6.0982700	-2.3408210	0.3726240
H	-6.8169150	-0.0165680	1.8459050
H	-5.5170730	-0.2858900	3.0363250
H	-5.6405510	1.2778300	2.1948200
H	-0.8313390	-0.5277380	1.2168350

I(Pb$_2$)$_2$ (dication)

C	-0.5061880	-1.8935370	5.0101910
C	0.7368780	-1.8930070	4.1199170
C	0.1713200	-1.8069210	2.6773520
C	-1.1254870	-0.9242560	2.8685310
C	-1.1128640	-0.6335530	4.3915940
C	-0.9734360	0.4826950	2.2615430
C	0.2303880	1.0069750	3.0535960
C	1.5177410	0.1323320	2.8672810
C	1.2947140	-1.0627420	1.9323200
C	0.0326810	0.4042870	4.4770470
C	1.3109410	-0.4651870	4.2913820
P	0.9872940	-0.6489430	0.1832490
N	2.1050850	0.3897990	-0.3439360
P	3.5484890	-0.0158580	-0.9385640
C	4.4087760	-1.2800780	0.0550810
C	5.8357010	-1.6080200	-0.3898070
P	-0.8625430	0.5392860	0.4424830
N	-0.8185100	2.0452840	-0.0710840
P	0.1190490	3.2838340	-0.4277010
C	1.4667710	3.5527280	0.7649190
C	2.4778420	4.6335190	0.3776560
N	0.8564690	-1.9913750	-0.6638090
P	-0.1570440	-3.0207930	-1.3338490
C	-1.1072600	-2.3378680	-2.7350080
C	-0.3367750	-1.2728050	-3.5105640
N	-2.0488750	-0.3536310	-0.1863450
P	-3.4863680	0.2005000	-0.6608790
C	-4.2018420	1.3957020	0.5166940
C	-5.5606020	1.9878030	0.1394800
C	-3.3903000	0.9911860	-2.3048080
C	-4.6535870	0.9515380	-3.1681460
C	-4.6180820	-1.2144100	-0.7858690

I(Pb$_2$)$_2$ (protonated)

C	-1.0665450	-0.1045470	2.8313410
C	0.4144690	0.3577180	3.0516380
C	1.4571820	-0.1521330	2.0530240
C	1.2141880	-1.6681580	2.1733170
C	-0.2801980	-2.1392100	1.9300380
C	-1.2961050	-1.0186820	1.6297020
C	0.5447720	-0.6635070	4.2287640
C	1.3738780	-1.8694960	3.7063130
C	-0.7415020	-2.5338110	3.3645760
C	-0.9197510	-1.1275560	3.9998750
C	0.5106670	-3.0819660	4.0410270
P	1.8041510	0.6176870	0.3715000
N	1.3365450	2.1963540	0.7535720
P	0.9003070	3.3523670	-0.2324330
C	2.2655130	4.3567020	-0.9238400
P	-1.5730150	-0.3490660	-0.0362000
N	-1.8863970	-1.6304230	-0.9654900
P	-1.2532180	-2.1917680	-2.3178670
C	-2.5658040	-2.7022670	-3.4665190
N	3.5033710	0.6480300	0.4711860
P	4.5462060	-0.2216580	-0.3376930
C	4.2598190	-2.0280660	-0.2303080
C	4.7394330	0.1357430	-2.1277780
C	6.2165370	0.0776990	0.3310730
C	-0.0862900	2.8082240	-1.6790680
C	-0.1292650	4.5583010	0.6663370
N	-2.7003740	0.8014890	0.0369550
P	-4.2763870	0.8377690	0.2988280
C	-5.2491710	0.8859450	-1.2472270
C	-4.9531340	-0.5058720	1.3379240
C	-4.7230900	2.3812190	1.1513170
C	-0.1706220	-1.0138240	-3.1944500

C	-4.7983850	-1.9681610	0.5324090
C	0.8353620	-4.3854690	-2.0097090
C	1.7890980	-5.0071510	-0.9901630
C	-1.3596610	-3.7116790	-0.1565280
C	-2.4426010	-4.5983660	-0.7733750
C	3.4687440	-0.6580830	-2.6400870
C	2.8727670	0.3272790	-3.6455310
C	4.5785710	1.4788100	-0.9602620
C	4.9642500	1.9739780	0.4350320
C	0.8840060	3.1500960	-2.0718410
C	-0.0976370	2.7491920	-3.1715320
C	-0.9318040	4.7670290	-0.4427330
C	-1.5930100	5.0513690	0.9067530
H	-0.2709500	-1.7776250	6.0725970
H	-1.1306480	-2.7826430	4.8788880
H	2.4357470	0.6890980	2.6763650
H	-1.8600000	1.0817150	2.5090330
H	2.1896120	-1.6982340	1.9247350
H	-2.0737140	-0.2965470	4.7843530
H	-2.0234210	-1.4432500	2.5288150
H	-0.0126730	1.0878810	5.3246390
H	2.1181040	-0.3659870	5.0169390
H	1.4552880	-2.7018540	4.2637000
H	-0.0607540	-2.7826780	2.2487010
H	0.3835700	2.0829990	2.9880310
H	1.3891800	-3.9752340	-2.8608660
H	0.1405430	-5.1302510	-2.4112370
H	-2.0182260	-1.9099690	-2.3126090
H	-1.3976310	-3.1732910	-3.3800260
H	-1.7967320	-2.8356320	0.3335280
H	-0.7868100	-4.2611350	0.5969550
H	4.4811550	-0.9495090	-2.9362430
H	2.8654850	-1.5699910	-2.5780970
H	5.4670020	1.2643780	-1.5630760
H	4.0079820	2.2404550	-1.5008430
H	3.7753560	-2.1736490	0.0224340
H	4.3976060	-0.9166320	1.0881000
H	1.6669320	2.3949830	-1.9610930
H	1.3661100	4.1059610	-2.2997960
H	0.9948090	3.7916450	1.7224770
H	1.9481830	2.5748680	0.8682570
H	-1.6882950	4.5982870	-1.2156780
H	-0.3186110	5.6123600	-0.7714220
H	-3.0520960	2.0175830	-2.1273160
H	-2.5688810	0.4786720	-2.8155820
H	-5.5780540	-0.8437070	-1.1582950
H	-4.2178440	-1.8735040	-1.5633690
H	-3.4516810	2.1873440	0.6183380
H	-4.2531440	0.8768210	1.4797960
H	-3.0481740	-4.0525530	-1.5019130
H	-2.0184240	-5.4734990	-1.2728090
H	-3.1130300	-4.9559870	0.0112510
H	0.5842750	-1.6677090	-3.9481170
H	-0.9512920	-0.8793940	-4.3238770
H	-0.0671690	-0.4435810	-2.8507910
H	2.4664180	-4.2527000	-0.5830290
H	1.2468990	-5.4657350	-0.1586300
H	2.3882250	-5.7853140	-1.4677160
H	1.8682490	0.6346590	-3.3473840
H	3.4914840	1.2224360	-3.7522710
H	2.7984730	-0.1449440	-4.6275560
H	6.2671320	-2.3476670	0.2878060
H	5.8604820	-2.0278020	-1.3980220
H	6.4796140	-0.7247040	-0.3701680
H	5.4729000	2.9370910	0.3591380
H	4.0845430	2.1063250	1.0697990
H	5.6406100	1.2761750	0.9354680
H	-6.3143890	1.2142420	-0.0305110
H	-5.9172470	2.6228030	0.9532720
H	-5.4910620	2.6087920	-0.7566280
H	-5.5129440	1.4092660	-2.6737410
H	-4.4737870	1.4989410	-4.0960170
H	-4.9201440	-0.0741440	-3.4341300
H	-5.2976550	-1.3496670	1.2827110
H	-5.4121940	-2.8562350	0.3688000
H	-3.8375700	-2.2893550	0.9411610
H	3.0173450	4.3753780	-0.5373340
H	1.9994090	5.6054080	0.2283590
H	3.2155360	4.7455720	1.1753210
H	-0.8743160	3.5041550	-3.3205080
H	0.4336310	2.6244980	-4.1179980
H	-0.5870540	1.8035180	-2.9275000
H	-0.8559680	5.3288540	1.6650460
H	-2.2977400	5.8796600	0.8086560
H	-2.1420760	4.1771910	1.2669630
C	-0.2517540	-3.6828710	-2.0120060
H	0.3818100	-3.2383340	5.1172000
H	0.8746350	-4.0098560	3.5875230
H	0.5194110	1.4159470	3.2890340
H	-2.2841030	-1.4848970	1.7763790
H	2.4263270	0.0339440	2.5376260
H	-1.6332070	-3.1647320	3.3776910
H	-0.3392260	-2.9686540	1.2218590
H	-1.6246240	-1.0750630	4.8308650
H	0.8252570	-0.2932210	5.2161260
H	2.4150120	-1.8933140	4.0350680
H	1.9127800	-2.2766540	1.5919020
H	-1.8138140	0.6807590	2.9486650
H	3.2213140	-2.1746090	-0.5395760
C	5.1957580	-2.9321300	-1.0311320
H	4.2935680	-2.2719010	0.8371650
H	6.4239960	1.1396510	0.1619240
C	6.3469150	-0.2673520	1.8141880
H	6.9352700	-0.4879040	-0.2714970
H	5.0613820	1.1802170	-2.1972490
H	5.5639980	-0.4734890	-2.5139810
C	3.4657660	-0.0862580	-2.9430200
H	1.8397850	5.2144430	-1.4561870
H	2.8165870	4.7527820	-0.0643040
C	3.1941410	3.5412130	-1.8234280
H	0.4695970	1.9709750	-2.1158710
H	-1.0129020	2.3876030	-1.2705190
C	-0.3972960	3.8617390	-2.7412050
C	-1.2784750	3.8926780	1.4199890
H	0.5324840	5.0918380	1.3567170
H	-0.4981930	5.2957290	-0.0547060
H	-5.2401130	1.9284130	-1.5826130
C	-4.7202060	-0.0251500	-2.3524800
H	-6.2853610	0.6436380	-0.9873280
C	-4.2456280	2.4711680	2.6000760
H	-4.2998550	3.1926440	0.5501770
H	-5.8132500	2.4720500	1.0919860
H	-4.3345290	-0.5365950	2.2414660
H	-5.9515070	-0.1820250	1.6527670
C	-5.0210510	-1.8840820	0.6743040
H	-2.0808380	-3.0924850	-4.3676090
C	-3.5219550	-3.7313770	-2.8595340
H	-3.1096820	-1.7990850	-3.7563230
H	0.5704270	-0.6745310	-2.4589480
H	0.3721890	-1.5742950	-3.9631880
C	-0.9121780	0.1754060	-3.8086600
C	1.1173660	-3.3653410	-1.4116770
H	-0.8425450	-4.3039050	-1.3314770
H	-0.1503260	-4.2318520	-2.9539260
H	-0.4140190	0.3004770	-0.4769080
H	3.1521210	-1.1348690	-2.9182950
H	2.6427440	0.5172560	-2.5499270
H	3.6249340	0.1885880	-3.9887760
H	5.0987740	-2.7588580	-2.1065900
H	6.2431760	-2.7771480	-0.7561670
H	4.9559370	-3.9818900	-0.8430710
H	6.2197450	-1.3399710	1.9883090
H	7.3353840	0.0147280	2.1852010
H	5.5927290	0.2649440	2.3991920
H	-1.8998780	4.6415010	1.9185660
H	-1.9139250	3.3098660	0.7466600
H	-0.8882910	3.2034520	2.1734710
H	-1.0237740	3.4269900	-3.5252270
H	-0.9402120	4.7132560	-2.3209540
H	0.5111980	4.2414430	-3.2166830
H	4.0400200	4.1473770	-2.1575570
H	3.5814530	2.6770350	-1.2765760
H	2.6746640	3.1743420	-2.7148310
H	-3.1615400	2.3567320	2.6637570
H	-4.5031450	3.4479960	3.0156240
H	-4.7120730	1.7077110	3.2282210
H	-4.7130580	-1.0737660	-2.0482590
H	-5.3418600	0.0696430	-3.2458270
H	-3.6966440	0.2517700	-2.6139500
H	-4.0545520	-2.1867940	0.2604820
H	-5.3287140	-2.6306210	1.4099620
H	-5.7554420	-1.8940430	-0.1355970
H	1.0334270	-2.6591770	-0.5797310
H	1.5929770	-4.2735160	-1.0346580
H	1.7806990	-2.9272260	-2.1625400
H	-0.1945730	0.8766540	-4.2416650
H	-1.5908200	-0.1398990	-4.6050290
H	-1.4940290	0.7187360	-3.0580960
H	-3.9617910	-3.3562890	-1.9315100
H	-4.3320060	-3.9453520	-3.5600480
H	-3.0123860	-4.6730380	-2.6383290

I(Pc2)2 (dication)				I(Pc2)2 (protonated)			
C	0.9160770	0.2866740	3.3156500	C	-0.0456700	-0.6587300	5.5036820
C	-0.3170900	1.2518970	3.2396700	C	-1.1986050	-0.8262480	4.5176040
C	-1.4170020	0.7529350	2.2941120	C	-0.7791520	-0.0066430	3.2633680
C	-1.7638750	-0.5990240	2.9416320	C	0.7908240	-0.1757120	3.2628710
C	-0.5201590	-1.5699490	3.0150560	C	1.0320610	-1.0766530	4.5074910
C	0.7574620	-0.9506650	2.4210520	C	1.3485350	-1.0898040	2.1585510
C	-0.7821250	0.7591170	4.6430400	C	0.5278620	-2.3597040	2.4043510
C	-1.9721290	-0.2092630	4.4286120	C	-1.0303590	-2.1836980	2.4248660
C	-0.1981660	-1.5958020	4.5330440	C	-1.5313610	-0.7628530	2.1550120
C	0.4429810	-0.1994500	4.7172750	C	0.5267790	-2.4423010	3.9663000
C	-1.5643270	-1.4609090	5.2063870	C	-1.0173280	-2.2719500	3.9811530
P	-0.9854800	0.7165410	0.5235060	P	-1.6395790	-0.1748310	0.3690230
N	-2.0914790	-0.1131510	-0.3117630	N	-1.0268230	1.3886090	0.5267620
P	-3.4709350	0.4947850	-0.8854920	P	-1.6914790	2.7296750	0.0295770
C	-4.6011700	-0.8810530	-1.2327080	C	-3.2081560	3.2708410	0.9019760
C	-5.1221700	-1.5991980	0.0157030	C	-3.1108470	3.1543170	2.4226230
C	-6.0738110	-2.7355190	-0.3498890	P	1.8041830	-0.4502950	0.5183820
P	0.7842200	-0.6010850	0.6318600	N	2.6599230	-1.5910440	-0.2356200
N	0.7018580	-1.9338860	-0.2267420	P	2.5097750	-2.7223010	-1.3381770
P	-0.2160170	-3.0734900	-0.8509440	C	1.3225620	-4.0457150	-0.9270680
C	-0.8490800	-2.6670920	-2.5066920	C	-0.1523540	-3.6303280	-0.9734460
C	0.2105750	-2.0846680	-3.4480760	N	-3.3264020	0.0219050	0.2087770
C	-0.2957030	-1.9628950	-4.8826680	P	-4.2272340	-1.0940760	-0.4694250
N	2.0567550	0.3317610	0.3004010	C	-3.6493570	-1.7698870	-2.0776610
P	3.4733450	-0.1713050	-0.2825420	C	-3.0486600	-0.7444630	-3.0412380
C	3.2820950	-0.7600870	-1.9925440	C	-4.4461900	-2.5925900	0.5626670
C	4.5234820	-0.9279990	-2.8687830	C	-5.2411520	-3.7603210	-0.0276800
C	4.1514920	-1.5769890	-4.2014730	C	-5.9086470	-0.4312410	-0.7016840
C	4.5928590	1.2585340	-0.2033550	C	-5.9880310	0.8619000	-1.5177580
C	6.0949990	0.9825240	-0.3339400	N	2.6526120	0.8846090	0.8406240
C	6.8928720	2.2806160	-0.2264680	P	3.9735340	1.4255960	0.1258850
C	4.2078930	-1.5436570	0.6592630	C	5.3956030	0.3027750	0.3257250
C	4.4732240	-1.2203980	2.1348700	C	6.7192550	0.7499400	-0.3014310
C	4.7340190	-2.4851960	2.9488710	C	3.8108970	1.6847550	-1.6744680
N	-0.7196980	2.2082870	0.0336950	C	2.5780500	2.4852800	-2.1034400
P	0.3997650	3.2737270	-0.3525950	C	4.4267010	3.0006840	0.9189110
C	1.3289800	2.8649700	-1.8726340	C	3.2502720	3.9409830	1.2046250
C	0.5301050	1.9745230	-2.8238170	C	-0.4362760	4.0353840	0.2473550
C	1.2261820	1.7639430	-4.1649690	C	-0.7845840	5.4364490	-0.2558320
C	-0.4675030	4.8311520	-0.7015280	C	-2.2094660	2.7816430	-1.7270390
C	-1.3687460	5.3028540	0.4432350	C	-1.0649460	2.5396340	-2.7103210
C	-2.1917380	6.5269810	0.0488790	C	2.0841900	-2.1258810	-3.0126690
C	1.6116200	3.5729510	0.9701690	C	1.0598290	-0.9881800	-3.0714670
C	2.7606960	4.5200390	0.6073900	C	4.1320400	-3.5205370	-1.5187820
C	3.7271930	4.6935940	1.7774260	C	4.6815040	-4.1053130	-0.2142550
C	-4.2970000	1.6337950	0.2733850	H	-0.1146550	-1.3278770	6.3680040
C	-5.6754330	2.1496190	-0.1546910	H	0.0735120	0.3702050	5.8589000
C	-6.2675930	3.0786420	0.9033260	H	-1.5663260	-2.9730940	1.8932150
C	-3.2651880	1.4160240	-2.4410100	H	2.3761730	-1.3420860	2.4700890
C	-2.6212840	0.6023000	-3.5662100	H	-2.5910930	-0.7450630	2.4475370
C	-2.4941090	1.4126090	-4.8542430	H	2.0660960	-1.0718500	4.8596870
C	0.8424130	-4.5412250	-1.0289370	H	1.3023970	0.7864960	3.3155290
C	1.4164680	-5.0340920	0.3046630	H	0.9616510	-3.3249090	4.4373950
C	2.5231190	-6.0654940	0.0989970	H	-1.6209180	-3.0374690	4.4712580
C	-1.6406670	-3.5350000	0.1790640	H	-2.2017210	-0.5904370	4.8798470
C	-2.6414440	-4.4990470	-0.4686080	H	-1.0524990	1.0472860	3.3194830
C	-3.6045980	-5.0732520	0.5684440	H	0.8855210	-3.2403650	1.8683680
H	-1.4939840	-1.2904700	6.2849820	H	0.4620350	3.6595960	-0.2559030
H	-2.2221010	-2.3161950	5.0232510	H	-0.1971740	4.0572120	1.3168840
H	1.8882520	0.7753000	3.2446190	H	-4.0026470	2.6129100	0.5334900
H	-2.2816990	1.4244920	2.3689710	H	-3.4496920	4.2961690	0.5956610
H	1.5927310	-1.6437370	2.5883060	H	-2.9755550	2.0043370	-1.8287170
H	-2.9562750	0.2124550	4.6399860	H	-2.6949290	3.7452340	-1.9242280
H	-2.6318310	-1.0848100	2.4909330	H	-3.4286430	-2.9198500	0.8103670
H	-0.9132950	1.4984810	5.4328820	H	-4.8939660	-2.2547920	1.5051520
H	1.1301570	-0.1034280	5.5576890	H	-6.5326350	-1.2122810	-1.1506920
H	0.4278530	-2.4372150	4.8351860	H	-6.2929800	-0.2574930	0.3107740
H	-0.7514950	-2.5570610	2.6163900	H	-2.8975610	-2.5349030	-1.8462300
H	-0.0750830	2.3089280	3.1320610	H	-4.4939670	-2.2887210	-2.5465160
H	1.6546520	-4.2494930	-1.7048330	H	1.7351650	-2.9933240	-3.5857960
H	0.2750420	-5.3313730	-1.5319220	H	3.0212480	-1.7972700	-3.4773310
H	-1.2794600	-3.5854260	-2.9233580	H	1.5095800	-4.8795930	-1.6140850
H	-1.6663230	-1.9533400	-2.3613840	H	1.5928920	-4.3940200	0.0764230
H	-2.1239110	-2.5928340	0.4602780	H	4.0529230	-4.2881700	-2.2970850
H	-1.2255700	-3.9793370	1.0899200	H	4.8066700	-2.7432620	-1.8979920
H	4.2722250	1.9626910	-0.9793710	H	3.7828320	0.6799810	-2.1170690
H	4.3780670	1.7326000	0.7612570	H	4.7264710	2.1657340	-2.0360760
H	3.4894290	-2.3679540	0.5645850	H	4.9205460	2.7291920	1.8598320
H	5.1254700	-1.8659550	0.1542160	H	5.1810140	3.4910960	0.2933900
H	2.5801990	-0.0596500	-2.4565620	H	5.5086310	0.1542830	1.4062450
H	2.7406520	-1.7070380	-1.8912860	H	5.0658170	-0.6568040	-0.0892360
H	-1.0587250	4.6571970	-1.6085460	H	0.6731880	-0.1296620	-0.2363120
H	0.2835150	5.5888280	-0.9514520	C	-1.5371360	2.5488800	-4.1617210
H	1.6096620	3.8081500	-2.3548240	H	-0.2963030	3.3101090	-2.5743860
H	2.2503200	2.3618030	-1.5726450	H	-0.5856320	1.5800730	-2.4769480

H	1.9908050	2.5803860	1.2388760
H	1.0531160	3.9580130	1.8304940
H	-4.3685310	1.1060380	1.2314940
H	-3.6027790	2.4707510	0.4191530
H	-4.2525920	1.7790130	-2.7471590
H	-2.6567460	2.2939850	-2.1945890
H	-4.0607370	-1.5809090	-1.8800560
H	-5.4325210	-0.4816680	-1.8249050
H	-3.2004590	-3.9737450	-1.2510110
H	-2.1157180	-5.3241730	-0.9625450
H	0.5162600	-1.1034310	-3.0709530
H	1.1105130	-2.7101520	-3.4431270
H	1.8084880	-4.1798130	0.8685270
H	0.6151110	-5.4716830	0.9099220
H	6.4177160	0.2903560	0.4500590
H	6.3126020	0.4963040	-1.2888840
H	4.9790960	0.0504470	-3.0540930
H	5.2748450	-1.5411510	-2.3589260
H	5.3290390	-0.5425780	2.2171530
H	3.6183670	-0.6825460	2.5577790
H	-4.2754760	-1.9852280	0.5934810
H	-5.6427030	-0.8875240	0.6658660
H	-5.5957130	2.6864980	-1.1053520
H	-6.3543290	1.3062150	-0.3232700
H	-1.6349760	0.2590070	-3.2377000
H	-3.2175380	-0.2955950	-3.7658570
H	0.3536500	1.0118280	-2.3294750
H	-0.4578450	2.4121410	-2.9973900
H	-2.0346300	4.4838180	0.7347380
H	-0.7559200	5.5394580	1.3204160
H	3.3074160	4.1316260	-0.2590870
H	2.3648890	5.4984820	0.3138760
H	6.6125160	2.9787990	-1.0214040
H	6.7096760	2.7727330	0.7340320
H	7.9650700	2.0858880	-0.3089400
H	3.3961530	-0.9829780	-4.7269570
H	5.0249470	-1.6668920	-4.8520980
H	3.7398370	-2.5792340	-4.0447310
H	4.9441900	-2.2401830	3.9930720
H	3.8636660	-3.1497420	2.9252710
H	5.5894360	-3.0392460	2.5504710
H	1.3110610	2.7075110	-4.7118050
H	2.2385240	1.3659400	-4.0378600
H	0.6632690	1.0615190	-4.7869000
H	4.1509470	3.7317450	2.0846120
H	4.5541160	5.3543530	1.5050160
H	3.2179040	5.1246820	2.6447300
H	-2.8419270	6.3019530	-0.8025760
H	-2.8234400	6.8542400	0.8789700
H	-1.5446140	7.3631850	-0.2337740
H	-5.6176490	3.9433840	1.0699880
H	-7.2484860	3.4466110	0.5919800
H	-6.3877370	2.5577010	1.8582660
H	-6.9465400	-2.3526090	-0.8875760
H	-5.5814640	-3.4735020	-0.9911620
H	-6.4271430	-3.2514790	0.5465370
H	-2.0036950	0.8276100	-5.6372500
H	-3.4789630	1.7135610	-5.2237260
H	-1.9053610	2.3218510	-4.6966410
H	-3.0647790	-5.6807140	1.3012200
H	-4.1221040	-4.2766150	1.1114480
H	-4.3607580	-5.7036870	0.0936810
H	-0.5292120	-2.9475380	-5.2987650
H	-1.2041860	-1.3561380	-4.9342660
H	0.4604630	-1.4964320	-5.5203020
H	2.1555550	-6.9324280	-0.4587660
H	3.3581410	-5.6333550	-0.4616780
H	2.9077110	-6.4191670	1.0592460

C	0.3764220	6.4079560	-0.0489760
H	-1.0406420	5.3992100	-1.3211170
H	-1.6721750	5.8121010	0.2656370
C	-4.4095640	3.5620550	3.1146210
H	-2.8651060	2.1176380	2.6742750
H	-2.2842140	3.7718530	2.7955330
C	-2.6603320	-1.3902020	-4.3682980
H	-3.7607320	0.0670360	-3.2246480
H	-2.1708940	-0.2869380	-2.5710400
C	-7.3771440	1.4928290	-1.4511730
H	-5.2401530	1.5686600	-1.1431840
H	-5.7333900	0.6546140	-2.5625900
C	-5.3013190	-4.9423300	0.9387710
H	-6.2581960	-3.4353740	-0.2741870
H	-4.7814320	-4.0870000	-0.9672520
C	-1.0602030	-4.7104060	-0.3903280
H	-0.3109000	-2.6893660	-0.4315270
H	-0.4402300	-3.4367040	-2.0135970
C	0.7392350	-0.6129130	-4.5151310
H	0.1384900	-1.2617640	-2.5453230
H	1.4636960	-0.1152840	-2.5465120
C	6.1049210	-4.6307720	-0.3859250
H	4.6603470	-3.3299790	0.5588180
H	4.0305850	-4.9161820	0.1314100
C	7.8212000	-0.2799480	-0.0575240
H	6.5922580	0.8927820	-1.3798500
H	7.0239660	1.7179750	0.1119440
C	3.6853950	5.1645980	2.0065360
H	2.7969850	4.2698700	0.2645540
H	2.4756390	3.3887010	1.7449790
C	2.4663240	2.5682510	-3.6232320
H	1.6770960	2.0249630	-1.6799470
H	2.6304720	3.4985530	-1.6921110
H	-7.6385750	1.7511440	-0.4198080
H	-7.4216320	2.4069610	-2.0497610
H	-8.1408400	0.8047930	-1.8280950
H	-3.5315090	-1.8415170	-4.8540180
H	-2.2316690	-0.6567130	-5.0567350
H	-1.9168700	-2.1799260	-4.2142510
H	-5.7817190	-4.6551580	1.8794860
H	-5.8664770	-5.7742560	0.5096030
H	-4.2942170	-5.3028770	1.1738910
H	6.1463150	-5.4179350	-1.1454200
H	6.7823940	-3.8287690	-0.6970980
H	6.4830550	-5.0459180	0.5521340
H	1.6461070	-0.3290830	-5.0589990
H	0.2829270	-1.4535080	-5.0457770
H	0.0428260	0.2285290	-4.5539780
H	-0.8250230	-4.8917660	0.6638620
H	-2.1122870	-4.4144820	-0.4534530
H	-0.9470310	-5.6571960	-0.9275130
H	7.5486100	-1.2489560	-0.4885630
H	8.7638500	0.0387700	-0.5103490
H	7.9912060	-0.4255280	1.0138250
H	1.5797570	3.1325010	-3.9242250
H	3.3430110	3.0642140	-4.0510700
H	2.3972170	1.5710240	-4.0671760
H	2.8343130	5.8243610	2.1972710
H	4.1082700	4.8708220	2.9726580
H	4.4446420	5.7408400	1.4682310
H	-2.0199750	3.4997340	-4.4089830
H	-0.6998980	2.4092470	-4.8524880
H	-2.2627420	1.7490510	-4.3424440
H	1.2654650	6.0751400	-0.5953490
H	0.1214910	7.4115570	-0.4003200
H	0.6424540	6.4777120	1.0109210
H	-5.2414470	2.9340300	2.7785290
H	-4.3276370	3.4600940	4.2006800
H	-4.6662000	4.6029990	2.8917420

I(Pd$_2$)$_2$ (dication)

C	0.8662000	0.7728970	-2.8487830
C	-0.4716410	0.8169760	-3.6096320
C	-0.0183100	0.6864230	-5.0873860
C	1.0545040	-0.4221830	-4.9629550
C	1.5056240	-0.4706300	-3.4730230
C	0.2048160	-1.7101160	-4.7535950
C	-1.2498930	-1.1776420	-4.7851760
C	-1.1882240	-0.0159370	-5.7775660
C	-1.3363970	-0.4890630	-3.3973320
C	-0.6456450	-1.5163950	-2.4822760
C	0.6510250	-1.7684400	-3.2620790
P	-0.4290210	-1.0498080	-0.7365330
N	0.3173050	-2.2148730	0.0219120
P	1.5072220	-3.0330040	0.6670790
C	0.7950970	-4.5611450	1.3491720
C	0.0080080	-5.3520370	0.2942820

I(Pd$_2$)$_2$ (protonated)

C	1.5979330	0.7717710	-4.2182850
C	1.9759590	-0.7288750	-4.0878150
C	1.0562340	-1.4295900	-5.0829010
C	-0.2221310	-0.8115940	-4.5268540
C	0.0744490	0.7154260	-4.5161410
C	-0.1779710	-1.1112630	-3.0022740
C	1.3721010	-1.0447580	-2.6894420
C	1.3082060	1.2959020	-2.7757580
C	1.8504610	0.1765020	-1.8877320
C	-0.2341060	1.2458940	-3.0768390
C	-1.0134900	0.0708640	-2.4769090
H	1.2517160	-1.1525760	-6.1241960
H	1.0775800	-2.5213920	-4.9939810
H	-0.7446390	2.2070250	-3.0042010
H	2.9439710	0.1950740	-2.0086790
H	-1.9623460	0.0356240	-3.0384480

C	-1.0403450	-6.2788730	0.9089560
C	-1.8375240	-7.0268070	-0.1564840
P	0.7340980	0.8139240	-1.0306080
N	0.0291240	2.1860480	-0.6291460
P	-1.3230020	2.8476050	-0.1054280
C	-1.1123210	4.6472840	-0.1588900
C	-0.7409360	5.1714370	-1.5504440
C	-0.3657230	6.6560800	-1.5456800
C	-1.5002990	7.5803130	-1.1062660
N	2.1607420	0.5357900	-0.3390080
P	3.1693920	1.6359210	0.2599930
C	4.6747040	0.7420260	0.7440220
C	5.6111090	1.4719760	1.7083880
C	6.8574760	0.6387350	2.0110690
C	7.7643530	1.2991130	3.0454310
C	3.6107310	2.9214370	-0.9486170
C	4.1829860	2.3307090	-2.2452870
C	3.9988750	3.2577770	-3.4468210
C	4.4652790	2.6119520	-4.7488240
C	2.5112550	2.4951520	1.7227950
C	1.9441560	1.5305750	2.7695530
C	1.4032520	2.2398210	4.0141970
C	2.4942420	2.8701840	4.8779600
C	-2.7544320	2.4030210	-1.1399940
C	-4.1359690	2.8391510	-0.6508620
C	-5.2414680	2.3891040	-1.6065270
C	-6.6325110	2.7404600	-1.0862990
C	-1.6521040	2.3856490	1.6222440
C	-2.5687340	3.2758560	2.4662080
C	-2.9026250	2.6122460	3.8038600
C	-3.8138810	3.4704690	4.6753650
N	-1.8362850	-0.5898970	-0.0976270
P	-2.8501750	-1.4759120	0.7810290
C	-4.2854480	-0.4110720	1.0923500
C	-5.4264220	-1.0176380	1.9110480
C	-6.6176600	-0.0602440	2.0306280
C	-6.2978710	1.2465140	2.7563480
C	-3.4155620	-2.9889540	-0.0520350
C	-4.1374780	-2.7186690	-1.3774860
C	-4.2608840	-3.9747210	-2.2440130
C	-5.0802240	-5.0899320	-1.5969640
C	-2.1660970	-2.0268950	2.3767160
C	-1.3191770	-0.9695710	3.0893450
C	-0.7301590	-1.4650580	4.4126640
C	-1.7747440	-1.6595540	5.5099630
C	2.7938090	-3.5604500	-0.5057910
C	3.6603850	-2.4276650	-1.0612030
C	4.6668130	-2.8956770	-2.1169770
C	4.0397990	-3.4142970	-3.4106250
C	2.2902980	-2.0937600	2.0080770
C	3.5506520	-2.6911700	2.6366580
C	4.0950800	-1.8028850	3.7577920
C	5.3632530	-2.3669510	4.3913860
H	-0.9530220	-0.3365440	-6.7969630
H	-2.1025990	0.5851020	-5.7953710
H	2.5732720	-0.5744720	-3.2772190
H	-1.2381760	-2.4416700	-2.4600170
H	1.4494570	1.6651790	-3.1124520
H	-2.0177400	-1.9361040	-4.9484970
H	-2.3573400	-0.2498530	-3.0917700
H	0.4048030	-2.5744960	-5.3865220
H	1.8234650	-0.4252930	-5.7355460
H	0.3321580	1.6231380	-5.5243020
H	-1.0471280	1.7227380	-3.4156200
H	1.2081270	-2.6469240	-2.9365150
H	-2.5538180	2.8163040	-2.1347110
H	-2.7079870	1.3111800	-1.2186700
H	-4.3335370	2.4026670	0.3338130
H	-4.1801650	3.9279930	-0.5273950
H	-5.0801450	2.8478710	-2.5891960
H	-5.1653670	1.3042700	-1.7588130
H	-6.8164320	2.2702890	-0.1140330
H	-6.7416590	3.8221290	-0.9577480
H	-7.4107910	2.4025460	-1.7756600
H	-2.0403460	5.0989130	0.2082760
H	-0.3265480	4.8905310	0.5667540
H	0.0998870	4.5854630	-1.9374730
H	-1.5820050	5.0155380	-2.2372040
H	0.5051000	6.8036410	-0.8949370
H	-0.0463180	6.9300020	-2.5571540
H	-2.3938950	7.4206010	-1.7194130
H	-1.7779890	7.4142870	-0.0607020
H	-1.2086860	8.6295660	-1.2045000
H	-2.0175380	1.3582000	1.5625930
H	-0.6641160	2.3216080	2.0883860
H	-2.0783390	4.2389170	2.6484730
H	-3.5013460	3.5014970	1.9385610
H	3.0436980	-0.9488680	-4.1589690
H	1.7321160	-1.9901080	-2.2828230
H	2.2360050	1.3637010	-4.8764840
H	-0.3147130	1.2740610	-5.3686550
H	-1.1631110	-1.0991830	-5.0018310
H	-0.6006980	-2.0806800	-2.7355980
H	1.6808690	2.2897190	-2.5207660
P	-1.7294150	0.0398310	-0.8036380
N	-2.6886110	1.3382750	-0.6441730
P	-2.4726730	2.5886380	0.3364890
C	-0.7361270	2.8566990	0.8219160
N	-2.4106750	-1.4058390	-0.6702170
P	-3.8123100	-2.1171670	-0.4891590
C	-4.6553860	-1.6334800	1.0577430
C	-4.9637840	-1.9294630	-1.9032740
C	-3.5141860	-3.9166260	-0.3316710
C	-3.0722770	4.0974110	-0.4705690
C	-3.4085050	2.4111740	1.8884880
C	-6.0911430	-2.1401200	1.2203010
H	-4.0149610	-2.0211400	1.8589060
H	-4.6081390	-0.5414570	1.1280200
C	-6.5829320	-2.0246280	2.6637360
H	-6.7548640	-1.5691600	0.5600990
H	-6.1662640	-3.1889420	0.9050780
C	-8.0591050	-2.3823340	2.8094260
H	-5.9732710	-2.6763530	3.3011350
H	-6.4186600	-1.0022730	3.0210240
H	-8.6837490	-1.7038940	2.2187040
H	-8.2537980	-3.4029090	2.4633610
H	-8.3816820	-2.3133900	3.8519740
H	-3.4189490	-4.1221110	0.7396440
H	-4.4099910	-4.4429410	-0.6799220
C	-2.2617200	-4.4171420	-1.0618550
C	-2.3168520	-4.3269050	-2.5866080
H	-1.3964640	-3.8514500	-0.6988290
H	-2.1025690	-5.4617230	-0.7698330
C	-1.0603060	-4.8957370	-3.2415730
H	-3.2025020	-4.8596710	-2.9554320
H	-2.4304820	-3.2788870	-2.8884160
H	-1.1050700	-4.8105020	-4.3308770
H	-0.1665810	-4.3597130	-2.9032380
H	-0.9255290	-5.9523220	-2.9881410
C	-5.1545220	-0.5058860	-2.4354250
H	-4.5483730	-2.5689550	-2.6882700
H	-5.9225230	-2.3762570	-1.6143760
C	-6.0119450	0.3921630	-1.5473470
H	-4.1769840	-0.0321520	-2.5782100
H	-5.6113140	-0.5736230	-3.4291920
C	-6.0397120	1.8360140	-2.0372860
H	-7.0303930	-0.0120640	-1.4896480
H	-5.6133010	0.3763760	-0.5292500
H	-6.6386460	2.4688080	-1.3752380
H	-5.0213380	2.2376360	-2.0653230
H	-6.4617970	1.9058670	-3.0452740
C	-4.9223150	2.3505160	1.6734950
H	-3.0475410	1.4766670	2.3351960
H	-3.1357740	3.2205330	2.5741710
C	-5.6712460	1.8085400	2.8892670
H	-5.3042810	3.3465880	1.4218760
H	-5.1377350	1.7122330	0.8097750
C	-7.1619690	1.6299150	2.6125350
H	-5.2296020	0.8453490	3.1771020
H	-5.5230510	2.4802300	3.7430180
H	-7.3226610	0.9429090	1.7733280
H	-7.6840500	1.2223320	3.4829610
H	-7.6305910	2.5849470	2.3540020
H	-2.9484540	4.9162110	0.2464750
C	-2.3633310	4.3840110	-1.7971360
H	-4.1482030	3.9725050	-0.6346770
C	-2.8400610	5.6753510	-2.4674540
H	-1.2784830	4.4437830	-1.6360970
H	-2.5363150	3.5368660	-2.4692050
C	-2.5082300	6.9400110	-1.6763720
H	-2.3751840	5.7374310	-3.4575160
H	-3.9224320	5.6153590	-2.6363080
H	-2.7893670	7.8360650	-2.2365780
H	-3.0377780	6.9740560	-0.7192330
H	-1.4343860	6.9974510	-1.4667730
H	-0.1223400	2.7350860	-0.0774420
C	-0.4131030	4.1884030	1.5052950
H	-0.4624040	2.0168720	1.4718950
C	1.0117920	4.1839750	2.0642220
H	-0.5219630	5.0083440	0.7841580
H	-1.1200660	4.3921070	2.3192840
C	1.4228890	5.5336630	2.6440750
H	1.0836860	3.4088990	2.8381970
H	1.7056690	3.8745980	1.2743510

H	-3.3922090	1.6476810	3.6139980
H	-1.9714820	2.3853310	4.3384480
H	-4.0362810	2.9721440	5.6227470
H	-3.3477150	4.4347450	4.9015760
H	-4.7642390	3.6675810	4.1678900
H	4.3407780	-0.1998930	1.1886980
H	5.1914540	0.4749010	-0.1847540
H	5.9102330	2.4432580	1.2954070
H	5.0855890	1.6785600	2.6492560
H	6.5463660	-0.3499960	2.3720730
H	7.4113740	0.4678440	1.0803970
H	8.1022150	2.2821980	2.7017120
H	7.2364910	1.4394180	3.9945280
H	8.6499120	0.6883380	3.2405610
H	1.7280290	3.1683750	1.3582930
H	3.3121250	3.1197100	2.1313270
H	1.1506170	0.9363220	2.3016640
H	2.7160000	0.8155730	3.0817310
H	0.6813800	3.0108510	3.7144680
H	0.8447660	1.5102590	4.6115750
H	2.0688270	3.3135870	5.7820900
H	3.2285740	2.1173290	5.1841830
H	3.0289850	3.6620780	4.3444500
H	4.2993160	3.6378540	-0.4892580
H	2.6728300	3.4566710	-1.1446980
H	5.2472120	2.1073660	-2.1109440
H	3.6944640	1.3726970	-2.4632450
H	2.9378970	3.5275440	-3.5321610
H	4.5405660	4.1949010	-3.2729700
H	3.8961750	1.6983830	-4.9546530
H	4.3322460	3.2888000	-5.5970270
H	5.5246410	2.3405540	-4.6968260
H	3.4126730	-4.3054130	0.0091890
H	2.2616490	-4.0896860	-1.3038330
H	3.0243530	-1.6357910	-1.4654840
H	4.2129180	-1.9658950	-0.2369180
H	5.3242740	-2.0511800	-2.3526760
H	5.3069250	-3.6730530	-1.6819280
H	4.8144670	-3.7215970	-4.1183890
H	3.3957710	-4.2822560	-3.2371100
H	3.4364130	-2.6411270	-3.8970630
H	1.5920030	-5.1644610	1.7952240
H	0.1364260	-4.2507110	2.1684260
H	-0.4965910	-4.6516480	-0.3832450
H	0.7017450	-5.9347790	-0.3223030
H	-0.5531330	-6.9920310	1.5842910
H	-1.7235540	-5.6826440	1.5277560
H	-2.6078040	-7.6592080	0.2931630
H	-2.3338930	-6.3246220	-0.8359110
H	-1.1853480	-7.6661880	-0.7600870
H	2.4802160	-1.1131530	1.5602340
H	1.5165500	-1.9395990	2.7680940
H	3.3361530	-3.6884860	3.0387220
H	4.3288690	-2.8237930	1.8749140
H	4.3057980	-0.8020240	3.3582170
H	3.3195660	-1.6690640	4.5217200
H	5.1754710	-3.3482710	4.8386170
H	6.1541890	-2.4874970	3.6433190
H	5.7403300	-1.7044830	5.1751120
H	-3.8929750	0.4823860	1.5866830
H	-4.6399090	-0.0925250	0.1048920
H	-5.7647310	-1.9508860	1.4471840
H	-5.0680300	-1.2714580	2.9156800
H	-7.0027290	0.1574770	1.0265750
H	-7.4207560	-0.5794830	2.5637210
H	-5.5791160	1.8615200	2.2034420
H	-7.2004790	1.8493470	2.8864380
H	-5.8759490	1.0533140	3.7487800
H	-1.5597780	-2.9112340	2.1579940
H	-3.0060940	-2.3623330	2.9934680
H	-0.5117050	-0.6600210	2.4168040
H	-1.9207480	-0.0758230	3.2889230
H	-0.1911980	-2.4070230	4.2460370
H	0.0176620	-0.7379920	4.7496600
H	-1.3035960	-1.9605950	6.4493010
H	-2.3230620	-0.7286210	5.6897920
H	-2.5033080	-2.4320950	5.2453910
H	-4.0396480	-3.5562740	0.6466970
H	-2.5093390	-3.5852590	-0.2208260
H	-5.1354650	-2.3117740	-1.1742910
H	-3.5950060	-1.9491270	-1.9389740
H	-4.7182310	-3.6924740	-3.1983290
H	-3.2539520	-4.3436160	-2.4806630
H	-5.1846380	-5.9409120	-2.2753780
H	-4.6117080	-5.4587710	-0.6788720
H	-6.0846660	-4.7366590	-1.3405620
H	1.4035300	6.3140180	1.8754710
H	0.7482680	5.8428650	3.4497990
H	2.4367030	5.4942420	3.0545820
P	1.6830830	0.2849320	-0.0253570
N	2.5190690	-1.1224650	0.4108130
P	1.8973250	-2.4421120	1.0192270
C	2.9486920	-3.0034030	2.3981280
N	2.7124240	1.5784310	0.2663090
P	4.2599170	1.6197520	0.5914750
C	5.4219630	1.1165690	-0.7437550
C	4.6984080	3.3502310	0.9666690
C	4.7857200	0.6563750	2.0590920
C	0.1877920	-2.3566150	1.6665740
C	1.8163150	-3.8078340	-0.1960260
C	5.5238670	-0.4038190	-0.9514670
H	5.0348020	1.6003240	-1.6496110
H	6.4042510	1.5595920	-0.5434130
C	5.9191220	-0.7902250	-2.3781140
H	6.2601440	-0.8115380	-0.2473420
H	4.5661090	-0.8761020	-0.7026020
C	7.2842060	-0.2583650	-2.8103630
H	5.9108180	-1.8842640	-2.4589390
H	5.1471700	-0.4245670	-3.0691250
H	7.3055290	0.8362440	-2.8060240
H	8.0705040	-0.6086750	-2.1325980
H	7.5382200	-0.5909060	-3.8210530
H	4.8884410	-0.3837950	1.7360500
H	5.7771250	1.0143620	2.3640750
C	3.7851830	0.7237300	3.2127480
C	4.2536950	-0.0568270	4.4414430
H	3.5911890	1.7658300	3.4987600
H	2.8314740	0.3155880	2.8585070
C	3.1660100	-0.1724710	5.5068090
H	4.5718590	-1.0609790	4.1324400
H	5.1428120	0.4262270	4.8649470
H	2.2875450	-0.6956390	5.1112870
H	3.5187100	-0.7244630	6.3827570
H	2.8357050	0.8167310	5.8412800
C	4.3737260	4.3165050	-0.1766970
H	4.1310410	3.6260860	1.8641400
H	5.7611110	3.3821420	1.2301730
C	4.5194500	5.7897820	0.2144670
H	5.0275220	4.1030340	-1.0323690
H	3.3469900	4.1312190	-0.5102640
C	5.9370460	6.1870290	0.6233310
H	4.2016860	6.4064430	-0.6342110
H	3.8247010	6.0112770	1.0343100
H	6.0032220	7.2613980	0.8175990
H	6.2553810	5.6681920	1.5328830
H	6.6546380	5.9435530	-0.1680040
C	3.1244500	-3.9875740	-0.9701860
H	1.5068910	-4.7353440	0.2988950
H	1.0055370	-3.5317910	-0.8830130
C	2.9519140	-4.8118960	-2.2453660
H	3.5192880	-2.9982420	-1.2336790
H	3.8749360	-4.4595520	-0.3231690
C	4.2533360	-4.9462200	-3.0316710
H	2.5584560	-5.8046890	-1.9942700
H	2.1942250	-4.3306620	-2.8782390
H	4.1118970	-5.5371390	-3.9410380
H	4.6304030	-3.9606440	-3.3265500
H	5.0283270	-5.4332570	-2.4304560
H	-0.1090990	-3.3620220	1.9888470
C	0.0014550	-1.3421890	2.7982570
H	-0.4581450	-2.1040120	0.8166730
C	-1.4722720	-1.0865530	3.1124260
H	0.5131170	-1.6953530	3.7030780
H	0.4718450	-0.3921870	2.5166060
C	-1.6673380	-0.0661420	4.2302760
H	-1.9688420	-0.7369290	2.1977950
H	-1.9593420	-2.0327280	3.3817960
H	-2.7289630	0.1134570	4.4269600
H	-1.2072860	-0.4115150	5.1618960
H	-1.2061360	0.8933620	3.9697490
H	2.9190410	-2.1825480	3.1241290
C	2.5814320	-4.3241040	3.0730840
H	3.9740440	-3.0326000	2.0114050
C	3.4709180	-4.6080140	4.2848100
H	1.5336520	-4.3029100	3.3988760
H	2.6698640	-5.1503160	2.3567080
C	3.1216060	-5.9227340	4.9774600
H	4.5198910	-4.6242770	3.9639490
H	3.3783030	-3.7773670	4.9962560
H	3.2346280	-6.7690640	4.2917460
H	3.7688500	-6.1014430	5.8407010
H	2.0848870	-5.9161520	5.3303710
H	-0.6723650	0.0876830	0.1205190

I(Pe2)2 (dication)				I(Pe2)2 (protonated)			
C	-0.7416110	0.5889660	4.8741550	C	-0.6299720	-1.3444570	4.4033420
C	-1.4496620	-0.7834250	4.7665130	C	-0.6292770	-2.7595380	3.7661110
C	-0.5431640	-1.7200930	5.5666430	C	0.6692240	-3.3801180	4.2756710
C	0.7353090	-1.3376570	4.8183000	C	1.5506020	-2.2255260	3.8082330
C	0.7682100	0.2065200	4.9049630	C	0.8760950	-0.9618250	4.4094090
C	0.3693840	-1.5344120	3.3259750	C	1.1864700	-2.0447110	2.3128460
C	-1.1557470	-1.1425100	3.2865140	C	-0.3552200	-2.3952880	2.2748980
C	-0.5737750	1.1433830	3.4304430	C	-0.8891660	-0.3359890	3.2515620
C	-1.4042230	0.1954080	2.5659160	C	-1.3357680	-1.2151200	2.0854350
C	0.9399290	0.7556410	3.4549750	C	0.6284240	0.0524970	3.2364520
C	1.2482410	-0.4934850	2.6203000	C	1.4892420	-0.5530970	2.1193780
H	-0.5069380	-1.4725320	6.6319190	H	0.6810950	-3.5211900	5.3615360
H	-0.8073030	-2.7763210	5.4550870	H	0.9176070	-4.3298940	3.7902820
H	1.6316030	1.5856330	3.3132440	H	0.8389950	1.1054680	3.4322740
H	-2.4623810	0.4619970	2.6675240	H	-2.3254830	-1.6109630	2.3484750
H	2.2977960	-0.7771260	2.7589130	H	2.5252310	-0.3902550	2.4556880
H	-2.5125270	-0.7776910	5.0175710	H	-1.5469660	-3.3329790	3.9155160
H	-1.7682560	-1.9625940	2.9097450	H	-0.5423950	-3.2251040	1.5921610
H	-1.1253490	1.2581460	5.6440730	H	-1.1976200	-1.2456520	5.3300380
H	1.3952770	0.6237560	5.6927390	H	1.3177730	-0.5870430	5.3338190
H	1.6579180	-1.8394280	5.1160170	H	2.6250840	-2.3122330	3.9911250
H	0.5390220	-2.5486270	2.9694680	H	1.7593670	-2.6947330	1.6489610
H	-0.7858110	2.1978210	3.2722170	H	-1.5529170	0.4953220	3.4802340
P	1.0543060	-0.3929400	0.8058360	P	1.6517480	-0.0011120	0.3915110
N	1.9540850	0.8193000	0.2292440	N	1.7754630	1.6046900	0.3822790
N	1.3267680	-1.8608600	0.2629980	N	2.8878150	-0.8123370	-0.2555910
P	1.1078840	-3.1138630	-0.6876370	P	4.4626270	-0.8559390	-0.0026410
C	-0.4251230	-4.0318210	-0.3236880	C	5.1788290	-1.4189060	-1.5810890
C	-0.5360880	-5.4208790	-1.0193920	C	5.0311160	-0.3698590	-2.7186320
C	-0.5996980	-4.3034150	1.1958360	C	6.6439150	-1.8865960	-1.6417360
C	-1.1163160	-5.7440880	1.2995680	C	6.9218290	-1.8761350	-3.1479240
H	0.5960820	-6.6632820	0.3512380	H	6.9695170	0.2595820	-3.4848400
C	-0.4536220	-6.4495420	0.1158880	C	6.2701330	-0.5711000	-3.6252530
H	-0.9293630	-7.3970240	-0.1477500	H	6.0061980	-0.5966650	-4.6850160
H	-2.2056580	-5.7719690	1.1899540	H	6.4319810	-2.7388080	-3.6142090
H	-0.8714780	-6.1994330	2.2623060	H	7.9878210	-1.9309150	-3.3822890
H	-1.2694670	-3.5682220	1.6435830	H	6.7910010	-2.8686970	-1.1850860
H	0.3554910	-4.2293620	1.7212580	H	7.3137530	-1.1806650	-1.1412740
H	0.2206120	-5.6014530	-1.7853070	H	5.0089410	0.6533230	-2.3332550
H	-1.5040130	-5.4895850	-1.5264030	H	4.0909640	-0.5249310	-3.2525390
C	0.0551120	-1.5177140	-2.7553760	C	6.6480830	1.0436420	0.2716210
C	0.0657550	-1.4297270	-4.2980420	C	6.8294820	2.3792400	0.9989490
H	0.6361950	-0.5590680	-4.6357780	H	6.4700680	3.1891000	0.3550730
H	0.2702840	-0.5667750	-2.2613780	H	6.9467320	1.0875370	-0.7797130
H	-0.9264680	-1.8376310	-2.3946500	H	7.2461860	0.2681000	0.7609400
C	1.0952560	-2.6002330	-2.4387010	C	5.1447340	0.7818390	0.4531460
C	0.7279480	-2.7413180	-4.7942550	C	5.9416490	2.2627540	2.2514510
C	0.8483520	-3.6322100	-3.5475010	C	4.8388530	1.2276170	1.9043840
H	4.6918390	-5.7492370	1.4528570	H	6.6635900	-3.5055800	3.5250270
H	1.6364560	-4.3854610	-3.6292140	H	3.8320730	1.6475860	1.9662230
H	4.9604880	-4.0138450	1.5117040	H	7.2946200	-4.0786610	1.9874690
H	1.7253540	-2.5337720	-5.1940900	H	5.5154240	3.2283400	2.5351730
C	3.8038370	-3.8761630	-1.1739990	C	4.5340940	-3.5190740	0.9660520
C	2.4974800	-4.2861190	-0.4744020	C	4.8745980	-2.0601240	1.3120260
C	2.9068040	-4.5382430	0.9998200	C	6.3245150	-2.1289440	1.8373080
H	2.6802860	-3.6620570	1.6156290	H	7.0432540	-2.0084690	1.0235450
H	-0.1012760	-4.1475810	-3.3736100	H	4.8863980	0.3861540	2.6023670
H	2.3533030	-5.3815480	1.4190680	H	6.5261060	-1.3361700	2.5620360
H	5.8630280	-4.4397740	-0.6706820	H	5.2634460	-5.3388240	1.9578100
H	3.7604450	-3.9797860	-2.2613940	H	3.4631130	-3.6879330	0.8348910
H	4.7612730	-5.7940750	-0.9418600	H	4.4481680	-4.1826230	3.0127810
H	4.0410390	-2.8346840	-0.9416310	H	5.0374970	-3.8219280	0.0398910
C	4.7088850	-1.2020070	1.2887780	C	3.2938380	3.1068090	-2.2244280
C	5.0087650	-1.4162630	2.7920120	C	4.6927980	3.7630770	-2.0950780
C	4.7277010	0.3372450	1.0649150	C	2.3958320	3.8253780	-1.1882480
P	3.5119690	0.8998250	-0.1884590	P	1.0960590	2.7326810	-0.5211820
H	4.1146970	-1.7826990	3.3080540	H	5.4320190	3.0160990	-1.7841480
H	5.7902590	-2.1633110	2.9493420	H	5.0413340	4.1732870	-3.0453190
H	3.7426810	-1.6419880	1.0251710	H	3.3553080	2.0394430	-1.9919040
C	3.8448220	-0.1293370	-1.6655780	C	0.0840130	2.0935690	-1.9077670
H	3.3549520	1.4658060	-3.0865830	H	0.4409740	3.8387400	-3.1950140
C	3.2012990	0.3879030	-2.9691320	C	-0.3594220	3.1241030	-2.9724920
H	2.1270440	0.2067660	-3.0018920	H	-1.2336320	3.7003320	-2.6593700
H	6.0923410	-0.9859680	-4.0257430	H	0.3250970	0.3652340	-4.7994240
C	3.9839790	-0.3475290	-4.0580490	C	-0.6221590	2.2648190	-4.2144760
H	3.6665450	-1.3969680	-4.1062130	H	-1.5836110	1.7454500	-4.1152820
H	3.8403330	0.0900200	-5.0487930	H	-0.6583330	2.8563220	-5.1328390
C	5.4271430	-0.2582330	-3.5559870	C	0.5241240	1.2511970	-4.1895390
H	5.8256670	0.7392660	-3.7711310	H	1.4362390	1.7182390	-4.5783520
C	5.3233550	-0.4648460	-2.0312510	C	0.6977320	0.9008420	-2.7023800
H	5.5336120	-1.5051060	-1.7686130	H	0.1293860	0.0056930	-2.4393910
H	6.0588280	0.1403750	-1.5002270	H	1.7416800	0.6926950	-2.4561540
C	5.4089400	-0.0382680	3.3379200	C	4.5471650	4.8510750	-1.0162500
C	3.8779680	2.6336510	-0.6172290	C	-0.0616070	3.6956870	0.5087180
H	5.1909600	0.0759860	4.4024760	H	5.4658880	5.0146280	-0.4466590

H	3.5918950	1.0192830	2.8012230
C	4.6322190	0.9572240	2.4711220
H	6.4834120	0.1239130	3.1990040
C	5.2646700	2.8642260	-1.2745520
H	6.0076890	2.1545330	-0.8990580
H	5.2291240	2.7553450	-2.3609620
H	5.1291800	5.0263730	-1.3847630
C	5.6684380	4.2660220	-0.8079060
H	6.7390400	4.4503400	-0.9227580
C	5.1981260	4.2820090	0.6472050
H	5.8771250	3.6753260	1.2582520
C	3.8085400	3.6369110	0.5724640
H	3.0777990	4.4084350	0.3301410
H	3.4862040	3.1868320	1.5133830
H	5.0541820	1.9628880	2.5140600
H	5.1627880	5.2824370	1.0848850
H	-0.9483850	-1.3147990	-4.6881130
H	0.1553330	-3.2263450	-5.5876700
C	4.4342640	-4.8042720	0.9695890
C	4.8389770	-4.7885670	-0.5142110
H	5.4580750	-1.6813940	0.6537940
H	3.0972450	2.8836670	-1.3439440
H	3.3390760	-1.0598440	-1.3929500
H	5.7046410	0.6111870	0.6509980
H	2.1635070	-5.2295060	-0.9213400
H	-1.2134610	-3.3546990	-0.6599520
H	2.0953680	-2.1775800	-2.5954380
P	-1.1015090	0.2618070	0.7650330
N	-1.9716400	-0.8962730	0.0500710
N	-1.3279860	1.7627240	0.3100860
P	-1.1126030	3.1640200	-0.4000080
C	0.4152950	3.9961190	0.1540230
C	0.5937450	5.4617490	-0.3382440
C	0.5223190	4.0587400	1.7031560
C	0.9910550	5.4828690	2.0323580
H	-0.6758030	6.4923060	1.0898670
C	0.3936340	6.3306010	0.9083650
H	0.8613300	7.3128360	0.8086490
H	2.0843470	5.5451480	2.0044140
H	0.6696760	5.8005090	3.0272730
H	1.1953550	3.2839040	2.0715740
H	-0.4483290	3.8869590	2.1712230
H	-0.0665640	5.7491110	-1.1574780
H	1.6141340	5.5883520	-0.7150630
C	0.2575320	2.3001680	-2.6870300
C	1.0570320	3.4527610	-3.3001650
H	1.8470070	3.1056520	-3.9709740
H	0.0333450	1.5609000	-3.4643810
H	0.7853760	1.7763970	-1.8830400
C	-1.0676520	2.9513740	-2.2227780
C	-0.0099530	4.2837300	-4.0186680
C	-1.2548810	4.2165210	-3.1094080
H	-4.7344560	5.4277230	2.0727360
H	-2.1721300	4.1313050	-3.6968650
H	-5.0029340	3.7019310	1.8834910
H	-0.2356480	3.8238100	-4.9861110
C	-3.8093170	3.9615030	-0.7864190
C	-2.5148450	4.2756250	-0.0177210
C	-2.9444770	4.2921290	1.4711760
H	-2.7261910	3.3281030	1.9422180
H	-1.3609110	5.1297730	-2.5203550
H	-2.3982130	5.0566470	2.0281500
H	-5.8799790	4.4219760	-0.2284400
H	-3.7572440	4.2235030	-1.8455390
H	-4.7934000	5.8135700	-0.2879570
H	-4.0353800	2.8944800	-0.7131450
C	-4.8095730	0.9564960	1.2018680
C	-5.1852410	0.9799350	2.7034490
C	-4.7868320	-0.5434820	0.7902370
P	-3.5012060	-0.9248990	-0.4627450
H	-4.3262280	1.2964770	3.3049810
H	-5.9917620	1.6871060	2.9105410
H	-3.8399780	1.4381010	1.0421180
C	-3.7627760	0.3028590	-1.7944150
H	-3.0960170	-1.0701370	-3.3720070
C	-3.0084460	-0.0109390	-3.1037610
H	-1.9442030	0.2174020	-3.0271640
H	-5.8788170	1.3892380	-4.1895420
C	-3.7476200	0.8304900	-4.1456760
H	-3.4685300	1.8871570	-4.0438530
H	-3.5235500	0.5236290	-5.1699970
C	-5.2157290	0.6357760	-3.7588210
H	-5.5578250	-0.3440020	-4.1101230
C	-5.2171890	0.6726910	-2.2183320
H	-5.4484580	1.6802880	-1.8618900
H	-5.9836060	0.0172570	-1.8034620
C	-5.5760210	-0.4615810	3.0581540
H	3.7128420	3.5522100	0.5147250
C	3.3865380	4.3660960	-0.1437270
H	4.2762700	5.8062560	-1.4793430
C	-0.7027590	4.9518820	-0.1317250
H	-0.0424360	5.4028650	-0.8805230
H	-1.6455890	4.7199450	-0.6339920
H	-1.7846660	5.6744350	1.6051810
C	-0.8812330	5.9277520	1.0382270
H	-0.9805660	6.9641690	0.7058640
C	0.3596870	5.6819460	1.8983670
H	1.2235930	6.1757380	1.4377360
C	0.5387090	4.1566150	1.8695300
H	-0.0323970	3.6935720	2.6803470
H	1.5776860	3.8472260	2.0068830
H	2.9616720	5.1562710	0.4793870
H	0.2636690	6.0709520	2.9151080
H	7.8745330	2.5906120	1.2375380
H	6.5314790	1.9084950	3.1014740
C	6.4590360	-3.5488610	2.4527780
C	5.1285890	-4.2733380	2.1585040
H	2.8822500	3.1896530	-3.2332700
H	-0.8348030	2.9452050	0.7034370
H	-0.7980680	1.7117140	-1.3787790
H	1.9001950	4.6773300	-1.6674210
H	4.2195460	-1.7683280	2.1437080
H	4.5431750	-2.2774350	-1.8310520
H	4.6008240	1.4611390	-0.2147680
P	-1.7302880	-0.2666470	0.5020870
N	-1.4169160	-1.3529590	-0.7304470
N	-3.4150740	-0.1817460	0.7257090
P	-4.2631040	1.1555680	0.8129020
C	-6.0539680	0.7586220	0.7113570
C	-6.6156390	0.5391270	-0.7137250
C	-6.4062610	-0.5358960	1.5100640
C	-7.0473570	-1.4831080	0.4842240
H	-7.9277540	-1.0423640	-1.4880960
C	-7.6821960	-0.5465030	-0.5442760
H	-8.6055540	-0.1125140	-0.1421530
H	-7.7582020	-2.1775590	0.9401980
H	-6.2652030	-2.0762790	-0.0018730
H	-7.1190990	-0.2892190	2.3027260
H	-5.5274730	-0.9877630	1.9745300
H	-5.8400440	0.1615860	-1.3816620
H	-7.0015970	1.4641030	-1.1500520
C	-2.7886680	2.7756880	2.6189410
C	-2.6418490	2.9249400	4.1521270
H	-2.6797130	3.9752370	4.4526340
H	-2.7199870	3.7328810	2.0955420
H	-1.9889940	2.1376040	2.2257740
C	-4.1523990	2.1005920	2.3890850
C	-3.8084590	2.1237000	4.7771770
C	-4.3570930	1.2418940	3.6463170
H	-3.6039340	4.6641340	-2.0866000
H	-5.4019490	0.9587230	3.7998330
H	-5.3529060	4.8269410	-2.2833490
H	-4.5928160	2.8089230	5.1151550
C	-3.7194540	1.8675970	-1.8984480
C	-3.8213490	2.4155290	-0.4476930
C	-4.7223170	3.6670310	-0.5204940
H	-5.7724240	3.4096830	-0.3382910
H	-3.7753190	0.3174420	3.5532120
H	-4.4383960	4.4235760	0.2166680
H	-5.5653840	2.4559010	-2.8817400
H	-2.6741420	1.8654210	-2.2113030
H	-4.1225330	2.9490570	-3.7713060
H	-4.0535520	0.8339560	-1.9874740
C	-3.0984020	-5.2771180	-1.0431900
C	-4.0597170	-5.8752000	-0.0109300
C	-2.5286990	-4.0334460	-0.3219470
P	-1.8451880	-2.7063270	-1.4060750
H	-4.7591720	-6.5932820	-0.4480050
H	-3.4898940	-6.3940340	0.7694480
H	-3.6652350	-4.9958220	-1.9376170
C	-3.0479680	-2.4633140	-2.7787180
H	-4.3877650	-1.3666010	-1.4903990
C	-4.4615480	-2.1214350	-2.2834340
H	-4.9864280	-2.9845990	-1.8650100
H	-4.1078630	-1.0750220	-5.3874070
C	-5.1572880	-1.5619880	-3.5302410
H	-5.5569440	-2.3917410	-4.1226110
H	-6.0014950	-0.9113030	-3.2831660
C	-4.0526290	-0.8275320	-4.3240100
H	-4.1614420	0.2584150	-4.2488120
C	-2.7051680	-1.2832030	-3.7131360
H	-1.9823120	-1.5645230	-4.4815420
H	-2.2585440	-0.4774080	-3.1200710
C	-4.7470730	-4.6378680	0.5746330

C	-3.8300320	-2.5869150	-1.1405620
H	-5.4027160	-0.7012710	4.1101190
H	-3.7106900	-1.4066080	2.4819610
C	-4.7370910	-1.3276580	2.1144290
H	-6.6389600	-0.6258520	2.8503420
C	-5.1786620	-2.7217320	-1.8986280
H	-5.9329430	-2.0390110	-1.4973150
H	-5.0740400	-2.5015930	-2.9634230
H	-5.0847600	-4.8634580	-2.2382210
C	-5.6339310	-4.1553310	-1.6070750
H	-6.7015130	-4.2970350	-1.7901330
C	-5.2346490	-4.3426760	-0.1428330
H	-5.9262800	-3.7851990	0.5002000
C	-3.8280680	-3.7355370	-0.0883470
H	-3.1111470	-4.4923740	-0.4084100
H	-3.5240710	-3.4199710	0.9118020
H	-5.1330060	-2.3399950	2.0181320
H	-5.2448170	-5.3851310	0.1841300
H	1.5322160	4.0517340	-2.5147110
H	0.3087350	5.3111730	-4.2090360
C	-4.4719420	4.5602620	1.4633330
C	-4.8619650	4.7557650	-0.0117540
H	-5.5349610	1.5019960	0.5932050
H	-3.0082590	-2.7467350	-1.8473510
H	-3.3011070	1.1896700	-1.3513250
H	-5.7390570	-0.7793810	0.3013960
H	-2.1860770	5.2808070	-0.3076760
H	1.2048060	3.3471700	-0.2324890
H	-1.8986520	2.2639320	-2.3880120

C	-0.3934490	-3.5321240	-2.1691720
H	-5.5223680	-4.2924910	-0.1196500
H	-3.9748010	-2.5654660	0.5267950
C	-3.6250150	-3.5884670	0.6917610
H	-5.2381600	-4.8307410	1.5323060
C	0.7210470	-3.8409830	-1.1464340
H	0.8192120	-3.0017860	-0.4481580
H	0.5253370	-4.7486760	-0.5681060
H	2.0043290	-4.8716980	-2.5564260
C	1.9842790	-3.9235450	-2.0049150
H	2.9000980	-3.8612880	-1.4109950
C	1.8132580	-2.7427080	-2.9628290
H	2.0778740	-1.8251360	-2.4251580
C	0.3096010	-2.7347510	-3.3074670
H	0.1160870	-3.2188000	-4.2690950
H	-0.0601990	-1.7124980	-3.3863180
H	-3.1987720	-3.6025750	1.6994700
H	2.4468810	-2.8107610	-3.8522680
H	-1.6728860	2.5382860	4.4832810
H	-3.4955200	1.5363420	5.6446410
C	-4.5617840	4.1407330	-1.9700130
C	-4.5423950	2.8325230	-2.7681510
H	-2.3129410	-5.9701210	-1.3569350
H	-0.7721320	-4.4769240	-2.5776620
H	-3.0787540	-3.3921410	-3.3625550
H	-1.6527460	-4.3611690	0.2484840
H	-2.8184290	2.7275120	-0.1342050
H	-6.5702610	1.6114820	1.1640140
H	-4.9327150	2.8724350	2.3509260
H	0.5595140	-0.4736990	-0.3364470

I(Pf$_2$)$_2$ (dication)

C	-0.8462870	0.3623580	4.7051090
C	-0.9734640	-1.1776850	4.6321160
C	0.2416080	-1.6824040	5.4119660
C	1.2566250	-0.8497730	4.6280360
C	0.6942100	0.5888970	4.7133110
C	0.9614100	-1.1895060	3.1417850
C	-0.5998520	-1.4257870	3.1509270
C	-0.9223180	0.9130870	3.2467650
C	-1.3573860	-0.3052180	2.4142510
C	0.6256210	1.1466990	3.2642570
C	1.3816460	0.1105690	2.4220040
H	0.2075880	-1.4233180	6.4746080
H	0.3988500	-2.7610620	5.3134960
H	0.9371630	2.1760550	3.0925840
H	-2.4369250	-0.4614720	2.5454620
H	2.4625050	0.2532130	2.5581900
H	-1.9494780	-1.5772230	4.9134610
H	-0.8544000	-2.4227850	2.7903170
H	-1.4482100	0.8516660	5.4707630
H	1.1254360	1.2178070	5.4920600
H	2.3076310	-0.9538400	4.9036690
H	1.4888420	-2.0787570	2.8016300
H	-1.5130950	1.8137970	3.0973160
P	1.0651680	0.3176710	0.6278140
N	1.0901380	1.8968590	0.3573970
P	1.8370180	2.9077080	-0.6326390
N	1.9233100	-0.6136640	-0.3350920
P	3.1811380	-1.5628920	-0.5743190
C	0.7927010	4.4192880	-0.6673120
C	3.5522110	3.2811340	-0.0526440
C	1.9744240	2.1552740	-2.3072670
C	2.8345340	-2.4465890	-2.1509900
C	4.7112210	-0.5455530	-0.7323930
C	3.3667740	-2.7559410	0.8149350
H	1.7995550	-2.7692330	-1.9891690
C	2.8276780	-1.4651470	-3.3298830
C	3.6765570	-3.6924260	-2.4455510
C	5.9161840	-1.2154820	-1.4029120
H	4.3773920	0.2685480	-1.3886570
C	5.0828000	0.0592310	0.6272220
H	2.3696180	1.1685820	-2.0397680
C	0.5795730	1.9311350	-2.8998060
C	2.9454820	2.7951350	-3.3019340
C	4.1740680	4.5625710	-0.6236810
C	3.6536790	3.2717060	1.4776490
H	4.1225530	2.4241800	-0.4350610
H	3.3320070	-2.0908190	1.6876500
C	2.1378030	-3.6713820	0.8626910
C	4.6782640	-3.5456930	0.8909940
C	1.0417240	5.3956870	-1.8207740
C	0.8071160	5.1284260	0.6929570
H	-0.0869010	1.4453570	-2.1806500
H	0.1176440	2.8695600	-3.2226010
H	0.6562820	1.2866270	-3.7804360
H	3.9720110	2.8013180	-2.9282980

I(Pf$_2$)$_2$ (protonated)

C	-1.0320240	0.0832260	4.2838410
C	-1.0900530	-1.4496300	4.0393380
C	0.0484520	-1.9997210	4.8940420
C	1.1068150	-1.0743510	4.3015210
C	0.4901190	0.3432920	4.4583400
C	0.9795810	-1.2646510	2.7632450
C	-0.5670640	-1.5189600	2.5751310
C	-0.9970030	0.7684970	2.8800030
C	-1.3652540	-0.3748110	1.9339680
C	0.5385350	1.0342620	3.0591350
C	1.4782570	0.1096560	2.2790410
H	-0.1019490	-1.8465520	5.9679910
H	0.2526080	-3.0599740	4.7108390
H	0.8322190	2.0833090	3.0249840
H	-2.4150300	-0.6102700	2.1511410
H	2.4782060	0.2525070	2.7190940
H	-2.0754980	-1.9025480	4.1707420
H	-0.7539420	-2.4786180	2.0904490
H	-1.7172050	0.4668550	5.0417720
H	0.8296850	0.9069970	5.3285630
H	2.1286950	-1.1802160	4.6736360
H	1.5640680	-2.1078890	2.3937800
H	-1.5938070	1.6760590	2.7582540
P	1.9005470	0.4253360	0.5390780
N	1.7850290	2.0124030	0.3755360
P	2.2663330	3.1403660	-0.6374520
N	3.3759290	-0.1650800	0.2524050
P	4.0603790	-1.4593760	-0.3718500
C	4.0162460	3.6489200	-0.3579080
C	2.1087840	2.6156090	-2.3954660
C	1.1875380	4.6198480	-0.3900040
C	3.4280080	-3.0112540	0.4055050
C	3.7910320	-1.6522220	-2.1867690
C	5.8726850	-1.2440790	-0.1434660
H	2.3470200	-2.8204020	0.4289810
C	3.6534960	-4.3029630	-0.3873060
C	3.9159480	-3.1472660	1.8535600
C	4.5822270	-0.6335030	-3.0130570
H	4.1514080	-2.6549240	-2.4402840
C	2.2929940	-1.5716760	-2.5034830
H	0.3745420	4.4598660	-1.1072530
C	0.5704640	4.6547340	1.0124100
C	1.8675430	5.9514060	-0.7335070
C	0.6912370	2.1120610	-2.6955080
C	2.5550960	3.6728110	-3.4097080
H	2.7928430	1.7596410	-2.4555690
H	6.0922640	-0.4270950	-0.8415790
C	6.2513110	-0.7441560	1.2544180
C	6.6903330	-2.4687760	-0.5661630
C	5.0132810	2.6245270	-0.9095140
C	4.2484400	3.8873100	1.1390800
H	0.0846650	3.7095550	1.2595180
H	1.3261300	4.8562970	1.7773590
H	-0.1731750	5.4562070	1.0543690
H	1.1195020	6.7486320	-0.7098680

H	2.9393280	2.2032850	-4.2220710
H	2.6666890	3.8178490	-3.5665060
H	-0.2027890	3.9814140	-0.8032230
H	0.9028010	4.9296860	-2.7981390
H	0.3197640	6.2136370	-1.7438840
H	2.0405920	5.8348420	-1.7862500
H	-0.0169010	5.8460010	0.7333530
H	0.6861150	4.4261410	1.5213120
H	1.7356400	5.6860680	0.8415710
H	3.7085670	5.4528920	-0.1933980
H	5.2341210	4.5876220	-0.3568670
H	4.1053030	4.6323770	-1.7101250
H	3.1916370	4.1615640	1.9122640
H	3.1704130	2.3983910	1.9184010
H	4.7082790	3.2652430	1.7667850
H	3.5325840	-4.4744440	-1.6979630
H	3.3609400	-4.1027720	-3.4087530
H	4.7442140	-3.4749780	-2.5147590
H	2.1762620	-0.6101670	-3.1383990
H	3.8298030	-1.0943790	-3.5623970
H	2.4494050	-1.9765660	-4.2190470
H	5.5435410	-2.8990510	1.0460810
H	4.6226040	-4.2242080	1.7468070
H	4.8578820	-4.1525260	0.0016440
H	2.1671840	-4.4170360	0.0622680
H	2.1207720	-4.2146430	1.8118830
H	1.2051230	-3.1058950	0.7742380
H	5.4965270	-0.6919970	1.3049540
H	5.8430200	0.8329120	0.4885460
H	4.2255310	0.5247570	1.1194910
H	6.7477000	-0.5053400	-1.4129600
H	6.2514250	-2.1070570	-0.8695070
H	5.7122950	-1.4912670	-2.4388490
P	-1.0583110	-0.3299150	0.6047530
N	-1.0834100	-1.8731400	0.1714910
P	-1.8542830	-2.7724820	-0.9044920
N	-1.9269890	0.6941830	-0.2492560
P	-3.1895530	1.6603420	-0.3697770
C	-2.0153440	-1.8469220	-2.4876780
C	-0.8190130	-4.2754730	-1.1184450
C	-3.5606400	-3.2000600	-0.3358350
C	-2.8637110	2.7075910	-1.8470830
C	-4.7209660	0.6626850	-0.6193330
C	-3.3638830	2.6949320	1.1423860
H	-1.8255510	3.0105580	-1.6679560
C	-2.8768330	1.8567260	-3.1235120
C	-3.7091560	3.9771260	-1.9923880
C	-5.9336740	1.3966030	-1.2038310
H	-4.3934740	-0.0768990	-1.3618210
C	-5.0789860	-0.0818820	0.6729820
H	-4.1348990	-2.3073690	-0.6167800
C	-4.1978330	-4.4130110	-1.0270060
C	-3.6350510	-3.3513160	1.1887100
C	-0.8116530	-5.1247950	0.1591590
C	-1.0944560	-5.1241910	-2.3635280
H	0.1758530	-3.8282790	-1.2263510
H	-3.3220000	1.9374910	1.9358270
C	-2.1361550	3.6023320	1.2832570
C	-4.6754010	3.4693670	1.3141100
C	-3.0123200	-2.3709190	-3.5232690
C	-0.6292270	-1.5724360	-3.0795000
H	-4.1495350	-4.3667810	-2.1158210
H	-3.7279540	-5.3449720	-0.7027000
H	-5.2528010	-4.4640400	-0.7442740
H	-3.1662350	-4.2815600	1.5191800
H	-3.1438760	-2.5289270	1.7113570
H	-4.6844970	-3.3762180	1.4950600
H	-2.3945530	-0.8901990	-2.1107840
H	-2.7536470	-3.3652720	-3.8945450
H	-4.0316290	-2.4010520	-3.1313180
H	-3.0141040	-1.6894930	-4.3791070
H	0.0511320	-1.1622130	-2.3271560
H	-0.1774170	-2.4761880	-3.5003050
H	-0.7168720	-0.8438100	-3.8907370
H	-1.7403260	-5.6916450	0.2657920
H	0.0091010	-5.8455020	0.1074070
H	-0.6714910	-4.5148270	1.0548450
H	-0.9722480	-4.5585900	-3.2891920
H	-0.3748540	-5.9474780	-2.3871740
H	-2.0944020	-5.5622230	-2.3563900
H	-4.7783660	3.7678170	-2.0625350
H	-3.5487710	4.6760140	-1.1695220
H	-3.4124060	4.4869430	-2.9131040
H	-2.2183770	0.9902240	-3.0363030
H	-3.8815940	1.5059120	-3.3744530
H	-2.5183940	2.4616860	-3.9606410
H	-4.8575130	4.1744680	0.5010450

H	2.6409130	6.2043080	-0.0021440
H	2.3228000	5.9553700	-1.7266240
H	4.1491440	4.5950140	-0.8942250
H	6.0295080	2.9330620	-0.6472730
H	4.8285350	1.6372240	-0.4757680
H	4.9660730	2.5471250	-1.9992820
H	5.2804420	4.2111020	1.3002160
H	3.5892620	4.6582950	1.5469620
H	4.0830300	2.9658710	1.7046260
H	-0.0173680	2.9441070	-2.7592920
H	0.6844180	1.6026360	-3.6639490
H	0.3160110	1.4167110	-1.9370520
H	2.5656160	3.2325800	-4.4106720
H	1.8573510	4.5154360	-3.4299970
H	3.5574550	4.0595780	-3.2061050
H	3.3089460	-3.8941030	2.3733380
H	4.9555610	-3.4850610	1.8875120
H	3.8411080	-2.2068620	2.4064880
H	4.7144310	-4.5084980	-0.5503730
H	3.2371060	-5.1409520	0.1793290
H	3.1521130	-4.2860800	-1.3575240
H	7.7523430	-2.2089890	-0.5689790
H	6.5579070	-3.2983770	0.1340670
H	6.4341790	-2.8218070	-1.5693140
H	7.2919430	-0.4076530	1.2395120
H	5.6197820	0.0943870	1.5559250
H	6.1677380	-1.5316270	2.0066560
H	2.1166490	-1.8870070	-3.5352150
H	1.6795190	-2.1998320	-1.8517030
H	1.9290310	-0.5440440	-2.4068680
H	5.6575430	-0.8252320	-2.9915880
H	4.2575240	-0.6830490	-4.0560620
H	4.4108430	0.3868750	-2.6587660
P	-1.4996490	-0.0961020	0.0720690
N	-1.2801190	-1.6901560	-0.4273400
P	-2.1747730	-2.7939860	-1.1025540
N	-3.1653140	0.1892860	-0.0139090
P	-4.0540360	1.4793040	-0.1738970
C	-1.3894770	-4.4595120	-0.8363890
C	-3.9019440	-2.9433010	-0.4498590
C	-2.3788690	-2.5668970	-2.9339950
C	-5.6437380	0.9212550	-0.9411630
C	-4.3493670	2.3471970	1.4400760
C	-3.4231990	2.8197180	-1.2912450
H	-5.2664870	0.2656540	-1.7358120
C	-6.4657950	0.0532220	0.0190350
C	-6.5112600	2.0058820	-1.5888000
C	-4.7014020	1.3919030	2.5856820
H	-3.3476710	2.7421390	1.6558720
C	-5.3259320	3.5256730	1.3803980
H	-2.7493220	-3.5089960	-3.3521420
C	-3.3897610	-1.4560240	-3.2375880
C	-1.0176300	-2.2473560	-3.5589620
C	-4.7456540	-4.0325880	-1.1187100
C	-3.9106250	-3.0988530	1.0725640
H	-4.3208900	-1.9565770	-0.6745510
H	-4.2488800	3.5284180	-1.4238140
C	-2.2326980	3.5718180	-0.6891600
C	-3.0508520	2.2360980	-2.6583440
C	0.1017150	-4.3450830	-0.5161900
C	-1.6291930	-5.4743040	-1.9612620
H	-3.1574670	-0.5565540	-2.6611810
H	-3.3589010	-1.2011540	-4.3017990
H	-4.4141620	-1.7536690	-2.9964870
H	-0.2783480	-3.0308030	-3.3664700
H	-1.1180250	-2.1386860	-4.6433730
H	-0.6225050	-1.3114840	-3.1516640
H	-1.8907240	-4.8323490	0.0658970
H	0.2765950	-3.5921490	0.2546770
H	0.4836650	-5.3106740	-0.1698060
H	0.6730060	-4.0626170	-1.4063390
H	-1.1008570	-5.1859750	-2.8753690
H	-1.2449550	-6.4521340	-1.6548580
H	-2.6865670	-5.5968760	-2.2060730
H	-4.7428470	-3.9642980	-2.2103250
H	-4.3986860	-5.0326630	-0.8387130
H	-5.7848920	-3.9449210	-0.7856750
H	-4.9410000	-3.0458540	1.4401990
H	-3.5009520	-4.0647910	1.3884900
H	-3.3367730	-2.3056670	1.5528720
H	-5.9912530	2.5313940	-2.3933730
H	-7.4000080	1.5387290	-2.0246080
H	-6.8541810	2.7484310	-0.8638590
H	-5.8388960	-0.6647870	0.5539230
H	-6.9930840	0.6693750	0.7538490
H	-7.2203350	-0.5039510	-0.5446980
H	-2.7347110	3.0397400	-3.3310080

H	-5.5402420	2.8090220	1.3964820
H	-4.6166340	4.0449040	2.2421190
H	-1.2029830	3.0528040	1.1267730
H	-2.1739390	4.4330620	0.5717270
H	-2.1137480	4.0362430	2.2870460
H	-5.8381610	-0.8397680	0.4607110
H	-4.2166420	-0.5930990	1.1076690
H	-5.4888120	0.5931840	1.4287140
H	-5.7410400	1.7801620	-2.2070930
H	-6.7639710	0.6890340	-1.2799940
H	-6.2653760	2.2263380	-0.5767720

H	-2.2203250	1.5311370	-2.5573710
H	-3.8854440	1.7124390	-3.1336840
H	-1.8760520	4.3163690	-1.4094030
H	-2.4896350	4.1071740	0.2282550
H	-1.4100040	2.8832890	-0.4718040
H	-5.0920910	4.2317780	0.5788330
H	-6.3539960	3.1778520	1.2415160
H	-5.2940590	4.0763570	2.3256050
H	-4.5619290	1.9055200	3.5422390
H	-5.7431890	1.0661520	2.5336520
H	-4.0659290	0.5045870	2.5771810
H	0.9633430	-0.2370850	-0.2701330

I(Pg$_2$)$_2$ (dication)

C	-1.2972050	-1.0342380	1.8019620
C	-0.2397430	-1.9624620	2.4309960
C	-0.8491580	-2.2506490	3.8285340
C	-1.3396020	-0.8357470	4.2250980
C	-1.4665160	0.0003400	2.9183100
C	-0.0181950	-0.0901840	4.5763940
C	1.0628910	-1.1735910	4.3427160
C	0.3649690	-2.4779600	4.7296540
C	1.1026470	-1.2123190	2.7941210
C	1.0561570	0.2792360	2.4235920
C	-0.1369480	0.7478740	3.2653940
P	1.0124750	0.6402080	0.6403090
N	2.1775120	-0.0817350	-0.1547990
P	3.6127270	-0.7240770	-0.2071530
N	3.6116270	-2.2473150	0.4508070
C	2.5171490	-3.1628620	0.1693780
P	-0.8987070	-0.3793190	0.1449150
N	-0.5272450	-1.5521210	-0.8642160
P	-1.0191290	-2.9266230	-1.4556270
N	-2.5336130	-2.7599690	-2.1477630
C	-3.3033260	-3.9112900	-2.6168110
N	-1.9706450	0.6931390	-0.3659850
P	-3.4753410	1.1029400	-0.0874560
N	-4.3270720	0.0939100	0.9185530
C	-4.9694610	0.5106420	2.1554810
N	-3.4781250	2.6322800	0.5497700
C	-2.4166410	3.1336140	1.4108560
N	-4.3332200	1.0914340	-1.5086100
C	-5.7806530	0.9246490	-1.5721650
C	-4.6444120	3.5008040	0.4634410
N	0.8771150	2.2284640	0.5248170
P	1.0568730	3.3459810	-0.5821350
N	0.0649330	3.3391270	-1.9156880
C	-1.2532980	3.9694260	-1.8545720
N	0.7505790	4.7888360	0.1782520
C	1.0982470	6.0243370	-0.5164040
N	2.5794910	3.2670460	-1.2725800
C	3.7182890	3.1565750	-0.3656840
C	0.6921880	4.9163590	1.6282640
N	4.0474510	-0.7955620	-1.8008320
C	3.7386340	0.2895960	-2.7206790
N	4.8013080	0.0806490	0.6258330
C	4.7034180	0.2517750	2.0684280
C	4.9447690	-1.8122500	-2.3257120
C	6.0401550	0.5651170	0.0387340
C	4.7937110	-2.8883080	1.0131730
C	2.9192520	3.9771910	-2.5042160
C	0.1176800	2.1574410	-2.7786550
N	0.1760890	-3.4106930	-2.5025450
C	1.1876020	-2.4851640	-3.0022380
N	-1.2324430	-4.2262430	-0.4436070
C	-0.1621430	-5.1519590	-0.0955550
C	-0.0707400	-4.5512810	-3.3787170
C	-2.3684180	-4.2959850	0.4682250
C	-2.7794290	-1.5658660	-2.9536580
C	-3.7583760	1.6397520	-2.7293290
C	-4.6325750	-1.2678770	0.5007490
H	0.1055970	-2.5272740	5.7914770
H	0.9400840	-3.3696900	4.4613530
H	-0.2238370	1.8304390	3.3599720
H	-2.2312850	-1.5958240	1.6850170
H	1.9619600	0.7806480	2.7870050
H	-1.6205590	-3.0233890	3.8260670
H	-0.0484940	-2.8591460	1.8386000
H	-2.1620340	-0.8057310	4.9396580
H	0.0429710	0.4410000	5.5259990
H	2.0313460	-0.9692860	4.8030070
H	1.9739430	-1.7347860	2.3953760
H	-2.3411300	0.6407090	2.8185380
H	6.8951180	-0.0402110	0.3662720
H	4.5625770	-3.2751030	2.0117300
H	5.9895610	0.5351520	-1.0491700
H	5.1223920	-3.7270270	0.3869250

I(Pg$_2$)$_2$ (protonated)

C	-0.2169940	1.1744890	5.2285820
C	-1.2354670	0.5654370	4.2695040
C	-0.7509190	0.9744420	2.8472980
C	0.8175400	0.9928980	3.0298240
C	0.9942610	0.5785280	4.5178060
C	1.5633080	-0.1565630	2.3400900
C	0.8338920	-1.3652530	2.9319210
C	-0.7285210	-1.3721870	2.7714680
C	-1.3365860	-0.1846760	2.0147580
C	0.6662330	-0.9372080	4.4271570
C	-0.8782900	-0.9464810	4.2637710
H	-0.3282260	0.8216000	6.2593950
H	-0.2211440	2.2696310	5.2244810
H	-1.1300970	-2.3493970	2.4980160
H	2.5773220	-0.1693450	2.7735530
H	-2.4050000	-0.2007080	2.2655080
H	1.9796110	0.8160990	4.9251370
H	1.2404610	1.9718870	2.7994740
H	1.1164850	-1.5630500	5.1989940
H	-1.4698220	-1.5730740	4.9331810
H	-2.2885420	0.7915380	4.4524640
H	-1.1123470	1.9538780	2.5328960
H	1.3206190	-2.3234400	2.7389250
P	-1.4649180	-0.2726490	0.1298920
N	-1.3571690	1.3248380	-0.3805440
P	-2.1772430	2.6503950	-0.2497720
N	-1.4763160	3.7400040	0.8144540
C	-2.1427510	4.8845500	1.4143660
N	-3.1172700	-0.7021870	0.0899920
P	-3.7764530	-1.7578640	-0.8618500
N	-5.4368500	-1.6967750	-0.6025360
C	-5.9632230	-1.2496420	0.6785500
N	-3.6800330	-1.6049090	-2.5396460
C	-4.4663570	-0.5534510	-3.1777460
N	-3.1748480	-3.3230510	-0.6452460
C	-3.4377450	-4.4240990	-1.5640580
C	-2.3551580	-1.7376040	-3.1385770
N	-2.2042350	3.3660650	-1.7698260
C	-2.6858460	4.7249110	-1.9475560
N	-3.7731810	2.6599630	0.2916750
C	-4.0384690	2.2239840	1.6600840
C	-2.1969410	2.5828110	-2.9913030
C	-4.8110260	2.2218720	-0.6362690
C	-0.0261870	3.8228710	0.8616980
C	-3.0609630	-3.7743840	0.7358910
C	-6.2677720	-2.7122090	-1.2371960
H	0.3575250	4.6296630	0.2198370
H	-1.8459610	1.5748120	-2.7705110
H	0.2933450	4.0269560	1.8907130
H	-3.1960980	2.5266020	-3.4491980
H	-1.5201780	3.0397280	-3.7232490
H	-3.2166790	4.8376150	1.2362600
H	-1.9663210	4.8914000	2.4976700
H	-1.7580690	5.8299960	1.0065970
H	-2.1372650	5.1996690	-2.7683570
H	-3.7581720	4.7602410	-2.1907440
H	-2.5180420	5.3180050	-1.0495430
H	-4.8682100	1.1278650	-0.6952340
H	-4.6247880	2.6217730	-1.6343980
H	-5.7766910	2.6094680	-0.2945720
H	-4.0845360	1.1305300	1.7269380
H	-4.9944530	2.6442850	1.9903700
H	-2.5617180	-5.0826090	-1.5997240
H	-3.6329560	-4.0495930	-2.5679900
H	-4.3007570	-5.0245130	-1.2436100
H	-2.8673830	-2.9241220	1.3893220
H	-2.2311510	-4.4834610	0.8298770
H	-3.9782070	-4.2783480	1.0754170
H	-6.9479200	-0.7937820	0.5256660
H	-6.0780900	-2.0827460	1.3879260
H	-5.2901870	-0.5106720	1.1082960
H	-5.8682550	-2.9733750	-2.2185600
H	-3.9949570	0.4326120	-3.0757700

H	5.6156910	-2.1781320	1.0963610	H	-7.2789220	-2.3155800	-1.3757770
H	4.5351880	1.3028710	2.3341250	H	-4.5582820	-0.7808580	-4.2445010
H	3.8891560	-0.3515370	2.4736570	H	-6.3399200	-3.6267950	-0.6303580
H	5.6288890	-0.0827330	2.5502350	H	-1.8010850	-2.5420250	-2.6559570
H	2.3054250	-3.7578270	1.0646110	H	-1.7655970	-0.8131500	-3.0487380
H	1.6197970	-2.6037320	-0.0986280	H	-2.4633140	-1.9776680	-4.2011310
H	2.7677420	-3.8497140	-0.6502430	H	-5.4630930	-0.5069560	-2.7406500
H	5.0134920	-2.6568580	-1.6403130	H	-3.2581610	2.5817430	2.3336340
H	5.9545900	-1.4181020	-2.4978930	H	0.4206330	2.8791090	0.5476740
H	4.5522380	-2.1813690	-3.2792700	P	2.0432820	-0.0561990	0.5993930
H	3.1135140	1.0328300	-2.2232410	N	3.0628790	-1.2592290	0.2776160
H	3.2000120	-0.1000340	-3.5925190	P	2.9090080	-2.7409280	-0.2390760
H	2.1118900	6.3608990	-0.2625250	N	1.4667020	-3.2962180	-0.8456050
H	0.3905360	6.8093740	-0.2331160	C	0.3673510	-3.6975530	0.0298630
H	1.0339850	5.8907540	-1.5980380	N	2.7046760	1.4085240	0.4639160
H	1.6518020	5.2569530	2.0385340	P	3.2849640	2.0862600	-0.8460160
H	0.4396190	3.9561420	2.0743700	N	2.4452810	1.9486150	-2.2772000
H	-0.0780650	5.6480800	1.8940830	C	2.3553560	0.6350960	-2.9166000
H	4.0493530	4.1409370	-0.0079300	N	3.3200420	3.7219200	-0.5436030
H	3.4680640	2.5293720	0.4891440	C	3.9642240	4.6014360	-1.5123860
H	4.5488340	2.6887260	-0.9014520	N	4.7797430	1.4364050	-1.2334190
H	3.6585590	3.3897080	-3.0590350	C	5.7056390	1.1876210	-0.1302640
H	-1.9808790	3.3561520	-1.3148110	C	3.3162690	4.2433400	0.8170710
H	3.3521190	4.9639240	-2.2946680	N	3.9402930	-2.9168200	-1.5401580
H	-1.1901960	4.9418040	-1.3683440	C	4.0972370	-4.2130800	-2.1889690
H	2.0359540	4.1059390	-3.1279630	N	3.1958620	-3.7792490	1.0377820
H	1.1389100	1.7811840	-2.8478170	C	3.1873860	-5.2258870	0.8502050
H	-0.2123610	2.4335720	-3.7842220	C	5.0825050	-2.0292610	-1.7129190
H	-0.5249910	1.3538260	-2.3987940	C	3.9964520	-3.3494710	2.1776120
H	-1.6075550	4.1181250	-2.8785730	C	1.0004140	-2.8208740	-2.1456970
H	4.6565190	0.7783480	-3.0733540	C	5.4605030	1.7517780	-2.4858580
H	6.2170930	1.6020260	0.3448430	C	1.2261440	2.7405230	-2.4599990
H	-2.0661090	-4.0555830	1.4957010	H	0.3267750	-1.9637850	-2.0213410
H	-3.8485450	-1.3332540	-2.9260220	H	5.9863770	-2.4220640	-1.2270600
H	-3.1513680	-3.6057410	0.1597340	H	1.8454850	-2.5318710	-2.7703590
H	-2.2388030	-0.7157470	-2.5411830	H	5.2892600	-1.9232590	-2.7836400
H	-2.4808310	-1.7174040	-3.9995140	H	4.8482940	-1.0476000	-1.3025640
H	0.1905680	-4.9795850	0.9292830	H	-0.3558370	-2.8806730	0.1391270
H	-0.5355690	-6.1794750	-0.1592610	H	-0.1441540	-4.5593990	-0.4144030
H	0.6760640	-5.0409050	-0.7808650	H	0.7434370	-3.9818170	1.0121700
H	-3.0527810	-4.8056670	-2.0475780	H	3.1772400	-4.7935920	-2.1093780
H	-4.3693660	-3.7021450	-2.4824750	H	4.3071330	-4.0571610	-3.2520690
H	-3.1205740	-4.1096940	-3.6805720	H	4.9232620	-4.7928810	-1.7563630
H	-0.7706860	-5.2500040	-2.9169270	H	3.9473240	-2.2662540	2.2778500
H	0.8697020	-5.0836500	-3.5508710	H	5.0471390	-3.6506790	2.0703570
H	-0.4755870	-4.2359460	-4.3490420	H	3.5979430	-3.8072930	3.0893840
H	2.1338850	-3.0245450	-3.1206760	H	2.5303970	-5.5028630	0.0251730
H	1.3352130	-1.6708250	-2.2939130	H	2.8083130	-5.7030390	1.7599600
H	-2.1839600	4.1656700	1.1258780	H	6.4285990	0.4261460	-0.4386110
H	-1.5100750	2.5442890	1.2766760	H	5.1619350	0.8133470	0.7363140
H	-2.7109940	3.1242880	2.4688370	H	6.2604630	2.0940260	0.1490710
H	-4.3359990	4.4867030	0.0995470	H	4.7370700	1.9826370	-3.2673380
H	-5.1258390	3.6267890	1.4408530	H	6.0444030	0.8801610	-2.8011140
H	-5.3801330	3.0986410	-0.2319960	H	6.1446840	2.6032560	-2.3752200
H	-6.0325480	0.1051830	-2.2548610	H	2.3694920	0.7555510	-4.0044620
H	-6.1934390	0.6977230	-0.5896520	H	3.1936780	0.0013230	-2.6242750
H	-6.2607540	1.8389440	-1.9419700	H	1.4208760	0.1288710	-2.6396120
H	-4.1096700	1.0570300	-3.5867470	H	1.0295040	2.8283860	-3.5330170
H	-4.6845860	-0.1677660	2.9677290	H	3.8694540	4.1943750	-2.5205430
H	-2.6722760	1.5760860	-2.6954390	H	1.3587900	3.7399440	-2.0468690
H	-4.6595980	1.5187850	2.4291360	H	3.4696550	5.5776890	-1.4948050
H	-4.0509810	2.6872190	-2.8774130	H	0.3645920	2.2689280	-1.9711420
H	-4.0469430	-1.5428510	-0.3809510	H	4.3364050	4.4107230	1.1882160
H	-5.6960050	-1.3831770	0.2578960	H	2.8075460	3.5432350	1.4777010
H	-4.3956200	-1.9666700	1.3113760	H	2.7821130	5.1992580	0.8320980
H	-6.0620240	0.4958370	2.0570920	H	5.0291400	4.7482030	-1.2872880
H	0.9010920	-2.0690450	-3.9771120	H	4.1932790	-5.6168000	0.6489160
H	-2.7831910	-5.3093110	0.4629800	H	0.4534740	-3.6263410	-2.6480390
				H	0.8994400	-0.1808660	-0.2113380
**I(Ph$_2$)$_2$ (dication)**				**I(Ph$_2$)$_2$ (protonated)**			
C	5.051253	-0.187523	1.898392	C	3.5191360	3.8674650	1.5559560
N	4.665695	-0.102992	0.474852	N	2.7051810	3.9277100	0.3364260
C	5.059211	-1.325446	-0.259352	C	2.9668120	5.2640120	-0.2080370
C	5.948421	-2.077091	0.734486	C	4.4762950	5.4939360	-0.0043060
C	5.401996	-1.659640	2.101375	C	4.8269170	4.6207580	1.2256290
P	3.501789	0.909325	-0.107015	P	2.6824150	2.5847110	-0.6919900
N	3.628538	2.232800	0.892967	N	1.3447770	2.9330840	-1.6352400
C	4.853208	3.026864	1.024101	C	0.9595000	1.9841570	-2.6869920
C	4.526774	3.949772	2.205865	C	-0.2597680	2.6584530	-3.3036570
C	2.985828	4.102650	2.165885	C	-0.9884040	3.2242430	-2.0772190
C	2.523724	3.184891	1.022654	C	0.1264010	3.5292280	-1.0530840
N	1.979371	0.487705	-0.154529	N	2.8036910	1.2849400	0.1519340
P	0.935685	-0.501761	0.528489	P	1.7095880	0.0585230	0.5395300
N	0.591652	-1.863219	-0.210754	C	1.3741930	0.5697650	2.3084080
P	1.128325	-3.174447	-0.892112	C	0.6722100	-0.3519190	3.3167230
N	1.691765	-4.408539	0.056473	C	0.9781190	0.3932460	4.6482490

C	0.752323	-5.162501	0.920030
C	1.523398	-5.384755	2.224397
C	2.985105	-5.373151	1.776998
C	3.002028	-4.273076	0.721457
P	-0.989640	0.562194	0.815399
C	-1.005724	0.602106	2.637560
C	0.192452	1.254261	3.349461
C	-0.132502	0.993978	4.844935
C	-0.613818	-0.476749	4.784544
C	-1.023433	-0.771856	3.312874
C	0.306340	-1.573369	3.103334
C	1.347705	-0.806212	2.279221
C	1.535306	0.449102	3.150840
C	1.782212	-0.158867	4.555058
C	0.708232	-1.274723	4.580767
C	1.237774	0.898031	5.516535
N	-2.133905	-0.382297	0.225354
P	-3.621407	-0.789533	0.581350
N	-3.654695	-2.406418	0.885069
C	-4.848882	-3.239688	0.777300
C	-4.319566	-4.510599	0.105249
C	-2.854525	-4.622423	0.580225
C	-2.511963	-3.255433	1.218456
N	-4.601778	-0.462205	-0.708226
C	-6.030345	-0.107585	-0.643488
C	-6.586987	-0.632229	-1.967219
C	-5.403901	-0.459516	-2.923163
C	-4.210550	-0.876837	-2.064852
N	-4.319805	-0.008734	1.856691
C	-4.461885	1.454238	1.914088
C	-4.772129	1.754425	3.397010
C	-4.471695	0.447113	4.144723
C	-4.771754	-0.630651	3.105636
N	-0.925137	2.074039	0.323880
P	-1.058614	3.035215	-0.926023
N	-0.556990	2.336618	-2.345392
C	-0.797457	2.979593	-3.651790
C	-0.247589	1.961152	-4.645930
C	-0.619745	0.626780	-3.999547
C	-0.397069	0.873009	-2.505186
N	3.956314	1.167382	-1.685964
C	3.072871	1.930418	-2.579994
C	3.800201	3.263076	-2.765221
C	5.288795	2.872288	-2.766148
C	5.345458	1.498520	-2.051514
N	-0.165144	4.382761	-0.598163
C	0.520022	5.223654	-1.587133
C	1.214657	6.279931	-0.726393
C	0.247320	6.462197	0.445812
C	-0.266019	5.045777	0.715885
N	-2.595816	3.572651	-1.233696
C	-3.156327	4.890247	-0.914966
C	-4.476550	4.879919	-1.682753
C	-4.941657	3.430508	-1.515999
C	-3.644046	2.621789	-1.635111
N	-0.190204	-3.817302	-1.658745
C	-1.219413	-2.919617	-2.225153
C	-2.038281	-3.839156	-3.129444
C	-1.007786	-4.859629	-3.618417
C	-0.162457	-5.109436	-2.369227
N	2.456503	-2.840720	-1.848893
C	2.406448	-1.712229	-2.789889
C	2.146076	-2.358310	-4.155677
C	2.817156	-3.747339	-4.053364
C	3.199650	-3.892995	-2.562108
H	1.183669	0.549484	6.552328
H	1.799879	1.836796	5.484855
H	-1.936655	-1.343582	3.152779
H	2.287230	-1.376921	2.262887
H	-1.910928	1.158321	2.905849
H	2.807641	-0.498992	4.714766
H	2.342145	1.101181	2.812744
H	0.916488	-2.116120	5.241481
H	-1.290355	-0.784803	5.581988
H	-0.850330	1.698134	5.269739
H	0.311420	2.312181	3.107618
H	0.174559	-2.613953	2.804933
H	1.228107	-6.310306	2.721731
H	1.341840	-4.555412	2.916352
H	3.686536	-5.170470	2.588744
H	3.253371	-6.331280	1.320765
H	3.096343	-3.287952	1.197460
H	3.819729	-4.382293	0.006116
H	0.495520	-6.112685	0.440058
H	-0.173230	-4.607233	1.082050
H	-2.523257	-3.289479	-3.938714
H	-0.761562	-2.113928	-2.815168

C	0.6700030	1.8564350	4.2245750
C	0.7149910	1.9052310	2.6623120
C	-0.8537410	1.9452940	2.6336830
C	-1.5342430	0.6303530	2.2498880
C	-0.9083240	-0.3258340	3.2778360
C	-1.2646670	0.4321180	4.5910300
C	-0.8830950	1.8848840	4.1920070
C	-0.1748550	0.0316360	5.5803340
P	-1.7551380	0.2246670	0.4942960
N	-1.8890450	-1.3716040	0.4084770
P	-2.1366060	-2.4841290	-0.6839530
N	-2.2471340	-1.8855080	-2.2283860
C	-1.3180330	-0.8170440	-2.6483880
C	-1.2507810	-0.9425470	-4.1750000
C	-1.5881380	-2.4122040	-4.4334530
C	-2.6747960	-2.6779650	-3.3960510
N	2.6389750	-1.2906700	0.9270470
P	3.8411270	-1.9734100	0.2109330
N	5.2329880	-1.0535670	0.3160170
C	6.4718430	-1.2565560	-0.4485620
C	7.4526780	-0.2503050	0.1632940
C	6.9783840	-0.1352770	1.6138960
C	5.4574250	-0.1773850	1.4788630
N	3.8448030	-2.3468410	-1.4305710
C	3.6480580	-1.2239960	-2.3733090
C	2.4586130	-1.6264640	-3.2785860
C	1.8714330	-2.8830480	-2.6230650
C	3.0877410	-3.4981370	-1.9346070
N	3.9839900	-3.5107040	0.8947740
C	3.7495190	-3.6732090	2.3323570
C	5.1511300	-3.6709400	2.9763800
C	6.0996760	-4.1315630	1.8425230
C	5.1744300	-4.3227060	0.6219150
N	3.9127740	2.6013610	-1.8260300
C	4.0225350	3.4848680	-2.9971250
C	5.4720660	3.2969770	-3.4762750
C	5.8795040	1.9391370	-2.8970250
C	5.1946170	1.9546480	-1.5362660
N	-2.9518560	1.1214330	-0.0884870
P	-4.4604730	1.4827040	0.1769000
N	-5.0578740	0.9857640	1.6452260
C	-6.2504600	1.5736880	2.2841550
C	-7.1758200	0.3773910	2.6214560
C	-6.4308430	-0.8694810	2.1114490
C	-4.9715860	-0.4166800	2.0701820
N	-4.7523770	3.1205430	0.2279100
C	-4.1223760	3.9036180	1.3140490
C	-3.2192500	4.8952390	0.5863260
C	-4.0373050	5.2137700	-0.6666270
C	-4.6287500	3.8538280	-1.0556530
N	-5.3361390	0.8843300	-1.0895470
C	-4.7431950	0.4987690	-2.3859790
C	-5.9624520	0.2323380	-3.2838710
C	-7.1167650	-0.0039040	-2.3037490
C	-6.7956190	0.9710500	-1.1740330
N	-0.9330430	-3.6028480	-0.5133590
C	-0.8923100	-4.8212040	-1.3361500
C	0.2066800	-5.6760030	-0.6921430
C	0.2505210	-5.1866880	0.7584930
C	-0.0068180	-3.6864830	0.6317350
N	-3.5470840	-3.3480800	-0.5370000
C	-3.6801670	-4.2784430	0.6064870
C	-5.1457750	-4.1531140	1.0287970
C	-5.8541010	-3.7427040	-0.2632100
C	-4.8563430	-2.7858460	-0.9143030
H	-0.1918640	0.6255750	6.5004000
H	-0.2004420	-1.0312940	5.8424530
H	1.1854140	2.7726330	2.1988660
H	-2.5882830	0.7405620	2.5440480
H	2.4160160	0.6572150	2.6535810
H	-2.3016860	0.2975500	4.9084800
H	-1.3302760	-1.3314200	3.2266200
H	-1.4069770	2.6731500	4.7347400
H	1.1934450	2.6304330	4.7885280
H	1.9907280	0.2228460	5.0203010
H	1.0521560	-1.3757460	3.2961160
H	-1.2800490	2.8333980	2.1607770
H	-5.7949610	-0.6112230	-3.9572560
H	-6.1759310	1.1129720	-3.8975370
H	-8.0988480	0.1751520	-2.7456920
H	-7.0929500	-1.0296050	-1.9202840
H	-7.1276500	1.9887590	-1.4253620
H	-7.2586130	0.6885180	-0.2253110
H	-4.1188090	-0.3882670	-2.2568980
H	-4.1083800	1.2923640	-2.7946310
H	-8.1581340	0.4833560	2.1555720
H	-6.7157530	2.3024840	1.6193120

H	-1.797206	-2.449331	-1.428088
H	2.130761	-4.539392	-4.364279
H	0.855489	-5.425505	-2.605989
H	3.704450	-3.820478	-4.685649
H	-0.388084	-4.419613	-4.407718
H	4.269630	-3.696746	-2.422752
H	-0.612486	-5.889385	-1.742331
H	2.985874	-4.877907	-2.145528
H	2.540956	-1.754128	-4.975273
H	1.656051	-0.985800	-2.486483
H	1.067918	-2.467153	-4.314678
H	3.376131	-1.201242	-2.768839
H	5.920280	-3.156305	0.569994
H	6.986975	-1.747081	0.633047
H	4.174427	-1.912886	-0.529424
H	2.074659	2.040775	-2.154638
H	2.981673	1.394453	-3.534130
H	5.665193	2.778621	-3.787577
H	3.494759	3.784744	-3.675200
H	3.578061	3.917336	-1.913933
H	5.904932	3.617256	-2.257493
H	5.726517	0.727790	-2.730386
H	5.985912	1.501790	-1.167433
H	4.501989	-2.232258	2.348382
H	4.249728	0.154282	2.554561
H	6.119150	-1.800607	2.912111
H	5.725238	2.394216	1.195811
H	5.031911	3.617768	0.112318
H	5.048393	4.904958	2.121474
H	4.839454	3.477728	3.140272
H	2.540529	3.784147	3.112829
H	2.395234	3.757257	0.093082
H	1.581888	2.668802	1.207075
H	5.927901	0.442730	2.088566
H	2.677241	5.135854	1.988758
H	-2.817172	-4.345541	-2.549368
H	-1.454140	-5.776657	-4.007419
H	5.594321	-1.072654	-1.178179
H	1.396639	7.202048	-1.281067
H	2.179536	5.903409	-0.370961
H	0.716783	6.902404	1.327914
H	-0.583785	7.108568	0.145356
H	0.350697	4.522326	1.452645
H	-1.291580	5.049529	1.095877
H	-0.190164	5.701119	-2.276657
H	1.226903	4.630420	-2.174667
H	0.840615	2.060194	-4.715992
H	-0.285306	3.939991	-3.722296
H	-1.868261	3.156836	-3.816201
H	-5.374753	3.299161	-0.518701
H	0.606897	0.583362	-2.191766
H	-5.688415	3.123766	-2.251475
H	-0.021128	-0.210413	-4.367100
H	-3.478097	2.286624	-2.668224
H	-1.114991	0.312297	-1.900204
H	-3.651462	1.732797	-0.997880
H	-4.293009	5.102262	-2.738964
H	-3.346663	5.002802	0.161763
H	-5.189665	5.608690	-1.292798
H	-2.480662	5.688792	-1.230885
H	-4.184340	2.595275	3.770565
H	-5.828377	2.013525	3.506545
H	-3.543438	1.945179	1.570614
H	-4.060035	-1.964183	-2.107988
H	-3.275767	-0.392881	-2.363138
H	-7.481154	-0.090192	-2.280542
H	-5.306326	0.592140	-3.214393
H	-5.486723	-1.058674	-3.831647
H	-6.842853	-1.693456	-1.874963
H	-6.148587	0.981777	-0.588019
H	-6.518499	-0.542727	0.231915
H	-3.417127	0.395690	4.433573
H	-4.243355	-1.568361	3.288430
H	-5.074230	0.332203	5.047798
H	-5.258887	-3.456380	1.773086
H	-5.623175	-2.740472	0.191190
H	-4.362304	-4.383864	-0.980266
H	-4.915335	-5.388879	0.361520
H	-2.730493	-5.424991	1.310848
H	-1.593844	-2.809220	0.828231
H	-2.412065	-3.360208	2.307575
H	-5.847568	-0.847921	3.064631
H	-2.183266	-4.832747	-0.255128
H	-0.669328	2.091712	-5.644156
H	-1.673414	0.398489	-4.193652
H	-5.276271	1.800118	1.268336

H	-5.9482840	2.1021180	3.1946750
H	-4.8373170	5.9194760	-0.4209210
H	-4.3371240	-1.0018160	1.4005520
H	-3.4440230	5.6447720	-1.4760630
H	-6.7563210	-1.1312500	1.0987870
H	-3.9481500	3.3351500	-1.7405800
H	-4.5234580	-0.4644920	3.0717760
H	-5.6026070	3.9414780	-1.5451570
H	-2.2807770	4.4013450	0.3099230
H	-4.8986900	4.4232770	1.8879990
H	-2.9851430	5.7756720	1.1882250
H	-3.5801990	3.2507100	2.0034020
H	-5.5320270	-5.0797280	1.4575970
H	-5.2481480	-3.3648720	1.7800930
H	-3.4438030	-5.2996960	0.2855750
H	-0.3308410	-0.9625820	-2.1921180
H	-1.6970210	0.1625180	-2.3400940
H	-1.9267730	-2.6043530	-5.4534110
H	-2.0100650	-0.3008980	-4.6347910
H	-0.2763320	-0.6443150	-4.5678640
H	-0.7155470	-3.0430200	-4.2327590
H	-3.6424060	-2.3062600	-3.7598640
H	-2.7967680	-3.7316280	-3.1393800
H	-6.0062580	-4.6158990	-0.9056230
H	-4.9671380	-2.7504350	-2.0010130
H	-6.8253570	-3.2725820	-0.0931700
H	-1.8613250	-5.3351590	-1.3179230
H	-0.6584380	-4.5843110	-2.3797850
H	1.1632430	-5.4833030	-1.1851490
H	-0.0047540	-6.7433930	-0.7813000
H	-0.5500010	-5.6580400	1.3389850
H	0.9199870	-3.1371700	0.4295220
H	-0.4596670	-3.2490240	1.5240730
H	-4.9857420	-1.7635120	-0.5332460
H	1.2032880	-5.3987830	1.2494360
H	-7.3311420	0.3166450	3.7012900
H	-6.5860920	-1.7405610	2.7516710
H	-2.9995630	-4.0169600	1.4223850
H	7.3617290	0.7190050	-0.3371780
H	8.4893590	-0.5811790	0.0690120
H	7.3287760	-0.9975860	2.1932780
H	7.3282330	0.7725140	2.1111310
H	4.9643570	-0.5717290	2.3718380
H	5.0295420	0.8123510	1.2813820
H	6.3119740	-1.0795530	-1.5164760
H	6.8596600	-2.2764400	-0.3368520
H	5.1944810	-4.3238510	3.8515680
H	3.2622370	-4.6438960	2.4925170
H	3.0866930	-2.8898320	2.6987280
H	1.1211380	-2.6215350	-1.8692540
H	4.8606250	-5.3715130	0.5465930
H	1.4087620	-3.5619050	-3.3450250
H	6.6298270	-5.0549400	2.0877230
H	3.7034980	-4.0485040	-2.6587840
H	5.6298930	-4.0497000	-0.3312700
H	2.8417520	-4.1731650	-1.1152720
H	2.8108970	-1.8552380	-4.2885360
H	3.4451910	-0.2961820	-1.8313840
H	1.7297000	-0.8166660	-3.3610660
H	4.5550340	-1.0679400	-2.9691780
H	0.0676430	3.4634880	-3.9689610
H	-0.8761720	1.9660860	-3.8825610
H	1.7784240	1.8291430	-3.3933310
H	2.3904320	5.9911770	0.3778060
H	2.6347850	5.3422980	-1.2447480
H	5.6264420	3.9134010	0.9864120
H	5.0307280	5.1534670	-0.8840970
H	4.7097480	6.5506800	0.1450870
H	5.1647790	5.2189190	2.0754670
H	3.6646440	2.8302050	1.8586250
H	2.9753650	4.3866770	2.3555880
H	-1.5726720	4.1168320	-2.3155570
H	0.2745870	4.6028470	-0.9073750
H	-1.6765830	2.4813640	-1.6626660
H	3.3068300	3.1837730	-3.7707590
H	3.8203470	4.5335750	-2.7614490
H	6.1084680	4.0810490	-3.0518610
H	5.5547390	3.3514540	-4.5638530
H	5.4733510	1.1243030	-3.5064410
H	5.7922980	2.5441850	-0.8210620
H	5.0284310	0.9686060	-1.1018390
H	-0.1145040	3.0967250	-0.0728730
H	6.9619450	1.8091410	-2.8237590
H	5.4125580	-2.6612030	3.3072270
H	6.8526520	-3.3655750	1.6362730
H	0.6939270	1.0073510	-2.2566070
H	-0.6319060	0.6642550	-0.2124070

II(Pa2)2 (dication)			
C	4.6152820	-2.6446540	0.2401800
C	3.1034040	-2.7681170	0.0843860
C	2.3052710	-1.5428540	0.5960200
C	2.8221720	-0.2467380	-0.1484860
C	3.9478370	-0.6499640	-1.1335770
C	5.1192690	-1.3810730	-0.4868100
C	1.7931900	0.2729180	-1.1780400
C	1.6967400	-0.9414140	-2.1157770
C	1.1852710	-2.2228790	-1.3797090
C	0.9000740	-1.9662950	0.1076070
C	3.1472310	-1.5230100	-2.1155200
C	2.6383460	-2.7995900	-1.3819720
P	-0.4881070	-0.8634870	0.5088250
P	0.2208830	0.9397640	-0.5562950
H	0.4064950	-2.7852510	-1.8955840
H	2.2358740	1.1147030	-1.7263970
H	0.6353690	-2.9117020	0.5985420
H	4.3120830	0.2504200	-1.6399030
H	3.1329950	0.5188250	0.5658560
H	3.6310590	-1.6765080	-3.0812590
H	2.8050400	-3.7514760	-1.8883830
H	2.7526220	-3.6620630	0.6110060
H	2.3593130	-1.4216760	1.6800580
H	1.2294580	-0.7296690	-3.0777100
H	5.0900680	-3.5448700	-0.1633140
H	4.8654100	-2.6099600	1.3054470
H	5.8498700	-1.6388130	-1.2603750
H	5.6258790	-0.7044250	0.2088950
N	-0.6242110	1.4345890	-1.8164400
N	0.4929750	2.0442980	0.5862980
P	-1.9493350	1.1350740	-2.6505840
P	0.7132570	3.6220960	0.3697400
C	1.8821180	4.0384710	-0.9501020
H	2.8418130	3.5569140	-0.7498420
H	2.0229030	5.1203910	-1.0054950
H	1.4853600	3.6784630	-1.9027640
H	-1.5419740	4.3407040	0.7936190
C	-0.8207250	4.5047740	-0.0099130
H	-1.2266900	4.1105970	-0.9434470
H	-0.6291210	5.5750300	-0.1139470
C	1.3569230	4.3052720	1.9095260
H	1.4814140	5.3862960	1.8176790
H	2.3206990	3.8447710	2.1352230
H	0.6559210	4.0898890	2.7186790
C	-1.9408390	-0.4736190	-3.4765450
H	-2.8837350	-0.6346900	-4.0043850
H	-1.7995480	-1.2449560	-2.7174090
H	-1.1152190	-0.5007850	-4.1911430
C	-3.4671140	1.2584040	-1.6733020
H	-4.3417410	1.0526440	-2.2950480
H	-3.4048700	0.5357140	-0.8582670
H	-3.5414270	2.2673560	-1.2616570
C	-2.0730890	2.3801520	-3.9527950
H	-2.1363250	3.3737710	-3.5049830
H	-1.1861080	2.3269530	-4.5869100
H	-2.9644590	2.1973680	-4.5573380
N	-0.5865280	-0.7892970	2.0998350
N	-1.8185460	-1.3280360	-0.2744170
P	-0.5418440	0.2394410	3.3178060
P	-2.9015360	-2.4170720	0.1981470
C	-2.1890870	-3.9733280	0.7907940
H	-2.9818790	-4.6843650	1.0342060
H	-1.5970110	-3.7761410	1.6881200
H	-1.5465130	-4.3962670	0.0154050
H	-4.4589500	-0.8870650	1.2058360
C	-3.9780910	-1.8149800	1.5225910
H	-3.3660380	-1.6189810	2.4049950
H	-4.7410200	-2.5589020	1.7628560
C	-3.9500400	-2.8013950	-1.2179300
H	-4.7296380	-3.5079030	-0.9258190
H	-3.3401620	-3.2389880	-2.0108080
H	-4.4129120	-1.8829030	-1.5850480
C	-1.6786830	1.6374360	3.1448720
H	-1.5900650	2.3090970	4.0018500
H	-2.7025480	1.2628150	3.0793510
H	-1.4246920	2.1686760	2.2262240
C	-1.0350720	-0.6539550	4.8080820
H	-0.3562990	-1.4948920	4.9630730
H	-0.9931110	0.0116410	5.6731170
H	-2.0527960	-1.0316220	4.6908200
C	1.1118520	0.8992350	3.6349690
H	1.7802420	0.0734110	3.8890810
H	1.4598030	1.3892160	2.7236010
H	1.0861110	1.6143030	4.4608860

II(Pa2)2 (protonated)			
C	0.4718260	4.9469050	0.3744300
C	1.0489200	3.6800780	-0.2439700
C	0.6660010	2.3537090	0.4670160
C	-0.9113240	2.2346730	0.5203690
C	-1.5143490	3.5065730	-0.1462250
C	-1.0611370	4.8303630	0.4561780
C	-1.5317460	1.2125120	-0.4696830
C	-0.9616340	1.7375630	-1.7917150
C	0.6001370	1.8205480	-1.8232620
C	1.3107620	1.3783950	-0.5374670
C	-1.0419060	3.2897290	-1.5989700
C	0.5095790	3.3791600	-1.6552110
P	1.5144630	-0.4057380	-0.0532770
P	-1.8047040	-0.5801180	-0.2186310
H	1.0606190	1.4378970	-2.7356050
H	-2.5988820	1.4731220	-0.4909280
H	2.3567120	1.6913000	-0.6563140
H	-2.6071860	3.4513760	-0.0904260
H	-1.2653960	2.0928730	1.5442000
H	-1.5951560	3.8676860	-2.3420390
H	0.9304390	4.0050870	-2.4451390
H	2.1413220	3.7568670	-0.2642250
H	1.0921740	2.2757570	1.4702610
H	-1.4292560	1.3177720	-2.6846470
H	0.7693070	5.8114600	-0.2292490
H	0.9078750	5.0952660	1.3681050
H	-1.5488270	5.6514990	-0.0802420
H	-1.4062920	4.8878740	1.4941470
N	-3.1383620	-0.9354170	-1.0749130
N	-1.9640610	-0.9184360	1.3407950
P	-4.6416160	-0.9612620	-0.5301400
P	-1.1150050	-1.3695990	2.6084740
C	-2.3000770	-1.8478390	3.8902310
H	-1.7784100	-2.1271240	4.8082090
H	-2.8906310	-2.6963030	3.5379960
H	-2.9661090	-1.0064370	4.0926300
H	0.3684560	-3.1466630	3.2934330
C	-0.0439690	-2.8062760	2.3402960
H	0.7670210	-2.5110170	1.6710330
H	-0.6262980	-3.6111440	1.8866100
C	-0.0692910	-0.0889570	3.3448800
H	0.3002810	-0.4388470	4.3127590
H	-0.6609380	0.8170550	3.4935920
H	0.7852230	0.1111470	2.6896140
C	-5.7541700	-1.1122500	-1.9438910
H	-6.7919510	-1.1518420	-1.6056690
H	-5.5158180	-2.0255750	-2.4927840
H	-5.6180180	-0.2521880	-2.6023410
C	-4.9992070	-2.3553350	0.5721770
H	-4.3643660	-2.2618080	1.4554620
H	-6.0509850	-2.3528880	0.8679900
H	-4.7645000	-3.2898840	0.0584610
C	-5.1576860	0.5180050	0.3895080
H	-6.1944750	0.4184280	0.7196040
H	-4.5095000	0.6323270	1.2621270
H	-5.0653450	1.4005510	-0.2477880
N	2.5771490	-0.2350340	1.2490530
N	2.4520250	-0.9131500	-1.3859100
P	4.1614390	-0.1950400	1.1930400
P	2.0039180	-2.0922720	-2.3463580
C	1.3226700	-3.5813240	-1.5421680
H	2.0494320	-3.9612820	-0.8200890
H	1.1054990	-4.3550800	-2.2823860
H	0.4029320	-3.3253370	-1.0105230
H	1.1234790	-0.8090340	-4.1890040
C	0.7531670	-1.6500240	-3.5979030
H	-0.1711590	-1.3454520	-3.0999310
H	0.5394760	-2.4932350	-4.2598900
C	3.4307080	-2.6673000	-3.3025280
H	3.8593160	-1.8251880	-3.8499230
H	3.1316250	-3.4439780	-4.0102730
H	4.1842290	-3.0692790	-2.6217150
C	4.9434430	1.0034850	0.0613930
H	4.6268660	0.7642210	-0.9563660
H	6.0333190	0.9506330	0.1322990
H	4.6123910	2.0140800	0.3132930
C	4.7957330	0.2195140	2.8400960
H	4.4403840	1.2119650	3.1256880
H	4.4274030	-0.5117040	3.5627830
H	5.8884460	0.2115450	2.8426470
C	4.9529330	-1.7782990	0.7633110
H	4.6525430	-2.0372030	-0.2537500
H	6.0417600	-1.7048690	0.8243780
H	4.6007760	-2.5522310	1.4494270
H	-0.8007830	-1.3091690	-0.8566600

II(Pb$_2$)$_2$ (dication)				II(Pb$_2$)$_2$ (protonated)			
C	-1.3036200	0.1344160	2.0079050	C	-1.4956290	-1.2390060	1.2910670
C	-1.8120840	-1.3192240	2.1473550	C	-0.8702560	-2.6572390	1.2872720
C	-2.1497350	-1.3035910	3.6588680	C	-1.6306400	-3.1884650	2.5363850
C	-0.8258180	-0.7613780	4.2250110	C	-1.2846040	-2.0725790	3.5428750
C	-0.2370730	0.1621640	3.1118250	C	-1.0908740	-0.7686560	2.6918090
C	0.3136410	-1.7933160	3.9735990	C	0.2409270	-2.1084840	3.8399710
C	-0.2600380	-3.0188000	3.2441680	C	0.8911830	-3.2470700	3.0281270
C	-1.4150150	-3.6838440	3.9850110	C	0.2817640	-4.6174270	3.2951030
C	-2.5442830	-2.6616390	4.2288810	C	-1.2284130	-4.5816880	3.0009530
C	-0.6581580	-2.3699810	1.8953460	C	0.6892830	-2.6865850	1.5921270
C	0.6850030	-1.6796260	1.5641810	C	1.3236460	-1.2920130	1.8306140
C	0.9051530	-0.8736190	2.8564040	C	0.4441730	-0.8090480	2.9855870
P	0.8087630	-0.7738380	-0.0116660	P	1.8511820	-0.1923710	0.4201970
N	0.5760120	-1.7851770	-1.2184140	N	3.1959840	-1.1293780	-0.0165640
P	-0.4838360	-2.3854320	-2.2455170	P	4.3306850	-0.6524220	-1.0096910
C	0.2876810	-3.8335230	-3.0276830	C	5.1869730	-2.1122450	-1.6962810
C	0.7686130	-4.8760620	-2.0178750	C	4.2558050	-3.1514410	-2.3210580
P	-0.7817830	0.7115480	0.3615560	P	-1.5573630	-0.1606630	-0.1702000
N	-0.3278940	2.2327740	0.4822770	N	-2.2052410	1.2563380	0.2576420
P	0.9151600	3.2222210	0.6041750	P	-3.7188110	1.7277530	0.4542840
C	1.8674930	3.3790400	-0.9401030	C	-4.6270230	1.8445020	-1.1228560
C	0.9845090	3.3797890	-2.1859510	C	-6.0226800	2.4625760	-1.0206280
N	-1.9393280	0.4106670	-0.7217620	N	-2.3712080	-1.0358310	-1.2670760
P	-3.2026610	1.3642360	-1.0342840	P	-2.1731470	-1.1617820	-2.8479560
C	-2.7797700	2.7655400	-2.1150100	C	-3.4670690	-2.2744360	-3.4672100
C	-2.2340870	2.3455430	-3.4795560	C	-3.3887890	-3.6795500	-2.8674210
C	-4.4562900	0.3661260	-1.8880300	C	-2.3577930	0.3959180	-3.7839230
C	-5.1183470	-0.6810410	-0.9900250	C	-1.4290550	1.5192730	-3.3132030
C	-3.9701540	2.0710520	0.4614350	C	-0.5555340	-1.8491670	-3.3791990
C	-5.2454230	2.8826750	0.2230690	C	0.1717480	-2.6806970	-2.3227690
N	2.1951130	0.0497420	-0.0606630	N	2.4005950	1.1451940	1.3145110
P	3.6147820	-0.4903420	-0.5997150	P	2.0861840	2.6511060	0.9214380
C	3.6902760	-0.6285190	-2.4136620	C	0.9845090	2.8936570	-0.5276460
C	3.5217170	0.7074770	-3.1357280	C	0.7494050	4.3420230	-0.9589320
C	4.8697280	0.7069030	-0.0644590	C	1.3043000	3.5724150	2.2941010
C	6.2957970	0.3653170	-0.5001930	C	-0.0891150	3.0543570	2.6466860
C	4.1008790	-2.1318670	0.0295090	C	3.6054220	3.6052760	0.5532790
C	4.3913660	-2.1647660	1.5312380	C	4.6208700	3.5907530	1.6979940
C	-2.0378600	-2.9243410	-1.4668320	C	3.8305700	0.4250320	-2.4179770
C	-3.1406770	-3.3484970	-2.4383700	C	2.5357520	0.0115780	-3.1175500
C	-0.9352010	-1.2363250	-3.5834340	C	5.6474030	0.3189300	-0.1849990
C	0.2527940	-0.4383690	-4.1152130	C	6.7441320	0.9075480	-1.0734280
C	0.2508880	4.8707670	0.9848830	C	-4.7289050	0.6844600	1.5658960
C	-0.6197920	4.8910560	2.2415840	C	-5.2725060	-0.5911730	0.9115130
C	2.0948020	2.7715220	1.9158740	C	-3.6742210	3.3877500	1.1896300
C	3.3450840	3.6507650	1.9893250	C	-2.9483920	4.4025510	0.3042330
H	1.9183160	-0.4918180	2.9878200	H	0.8391900	0.0692680	3.4974500
H	-2.1148100	0.8204620	2.2820090	H	-2.5814380	-1.4104350	1.3466520
H	1.4540540	-2.4581850	1.4785940	H	2.3101600	-1.4858630	2.2731140
H	-2.9567350	-0.5836020	3.8319500	H	-2.7045140	-3.1866120	2.3188450
H	-2.6824140	-1.5289090	1.5207580	H	-1.1075470	-3.2205730	0.3819180
H	-0.8995790	-0.3528670	5.2337710	H	-1.9520290	-2.0216510	4.4057430
H	0.9533310	-2.0309380	4.8246260	H	0.5318440	-2.0807360	4.8924170
H	0.5304090	-3.7542770	3.0606230	H	1.9654350	-3.2917440	3.2337570
H	-0.9518980	-3.1108640	1.1506190	H	1.2149830	-3.2722050	0.8332270
H	0.0833260	1.1623290	3.4015270	H	-1.6085300	0.1328050	3.0263130
H	-1.0439640	-4.0915210	4.9308100	H	0.4736040	-4.8998920	4.3361320
H	-1.7762470	-4.5343130	3.3979520	H	0.7860900	-5.3635990	2.6716540
H	-2.7507680	-2.5532050	5.2984480	H	-1.8111040	-4.8429550	3.8910930
H	-3.4781420	-2.9903040	3.7615790	H	-1.4975480	-5.3092780	2.2277320
H	-4.1673680	1.2280610	1.1321510	H	-0.7505630	-2.4395030	-4.2810890
H	-3.1981430	2.6872210	0.9359320	H	0.0706740	-1.0085270	-3.6910680
H	-2.0375470	3.3467090	-1.5580360	H	-3.3873180	-2.3038090	-4.5587250
H	-3.6719770	3.3890120	-2.2278870	H	-4.4217740	-1.7985110	-3.2204920
H	-3.9521110	-0.1079520	-2.7360420	H	-2.1809440	0.1583780	-4.8387370
H	-5.1995450	1.0514690	-2.3080450	H	-3.4057760	0.7000240	-3.6955680
H	2.3549150	1.7264360	1.7188050	H	-3.1695420	3.2766380	2.1548670
H	1.5446720	2.8026770	2.8612030	H	-4.7009240	3.7040920	1.3968390
H	2.5464220	2.5230200	-0.9590630	H	-4.6695470	0.8254530	-1.5216250
H	2.4722250	4.2890120	-0.8752440	H	-3.9912440	2.4227000	-1.8010770
H	1.0978120	5.5584990	1.0752670	H	-4.0821310	0.4480380	2.4177320
H	-0.3244150	5.1858760	0.1084400	H	-5.5507630	1.2957400	1.9528670
H	3.2830460	-2.8111360	-0.2365140	H	3.3270550	4.6338860	0.3022730
H	4.9750790	-2.4594750	-0.5421170	H	4.0376050	3.1618070	-0.3514330
H	2.8903910	-1.3258910	-2.6825940	H	1.4093570	2.3076200	-1.3499550
H	4.6407290	-1.1022550	-2.6782690	H	0.0317500	2.4156980	-0.2726460
H	4.7900850	0.7724670	1.0253580	H	1.9769430	3.4763920	3.1526690
H	4.5666190	1.6812410	-0.4601950	H	1.2775660	4.6346770	2.0275030
H	-1.4148590	-1.8174980	-4.3773830	H	5.1122910	1.1049380	0.3575290
H	-1.6838950	-0.5630470	-3.1577760	H	6.0736450	-0.3431080	0.5766340
H	1.1249840	-3.4585950	-3.6251970	H	5.7323340	-2.5529920	-0.8545870
H	-0.4384170	-4.2602380	-3.7272520	H	5.9364560	-1.7680150	-2.4164330
H	-1.7866810	-3.7433550	-0.7864690	H	4.6652030	0.4685310	-3.1257120
H	-2.3528010	-2.0702930	-0.8586670	H	3.7218050	1.4352620	-2.0048120
H	6.9836550	1.1286380	-0.1315210	H	-0.2538160	0.1380720	-0.5922500

H	6.6233430	-0.5982100	-0.1006220	H	-5.6823220	-1.2536220	1.6770200
H	6.3884650	0.3318220	-1.5890180	H	-6.0750070	-0.3608810	0.2058370
H	3.4759070	0.5454640	-4.2146970	H	-4.5012200	-1.1395390	0.3624440
H	2.5968260	1.2045070	-2.8343440	H	-1.9602780	4.0342600	0.0160490
H	4.3559590	1.3852240	-2.9336720	H	-3.5118710	4.6105670	-0.6097920
H	5.2890990	-1.5914360	1.7764790	H	-2.8194460	5.3451510	0.8405520
H	3.5633760	-1.7562030	2.1152970	H	-6.5081950	2.4457510	-1.9987970
H	4.5538950	-3.1947230	1.8548850	H	-6.6620350	1.9142530	-0.3231180
H	1.0276140	-1.0856240	-4.5359090	H	-5.9771410	3.5037320	-0.6900730
H	-0.0755420	0.2433350	-4.9030880	H	0.4427640	-2.0730240	-1.4545160
H	0.7022090	0.1539930	-3.3143300	H	-0.4334930	-3.5215040	-1.9749780
H	1.2873530	-5.6849830	-2.5365000	H	1.0985600	-3.0791490	-2.7432640
H	1.4596890	-4.4296900	-1.2985070	H	-1.6383790	1.8061470	-2.2782080
H	-0.0654610	-5.3156520	-1.4641310	H	-0.3744980	1.2340070	-3.3783980
H	-4.0168520	-3.6767070	-1.8748170	H	-1.5703820	2.4027090	-3.9398460
H	-3.4525890	-2.5229140	-3.0834940	H	-2.4755980	-4.1958610	-3.1765850
H	-2.8263120	-4.1790990	-3.0759790	H	-3.4095210	-3.6383660	-1.7754740
H	-1.9214330	3.2278400	-4.0420980	H	-4.2387180	-4.2767890	-3.2048200
H	-1.3642220	1.6940190	-3.3715450	H	2.2784280	0.7356000	-3.8962330
H	-2.9870170	1.8194180	-4.0725890	H	2.6222100	-0.9699760	-3.5901870
H	-5.7627890	-1.3281890	-1.5882840	H	1.7173510	-0.0313390	-2.3927990
H	-4.3759860	-1.3107500	-0.4932070	H	3.8110200	-2.7869900	-3.2507300
H	-5.7358990	-0.2144900	-0.2181280	H	4.8094720	-4.0642670	-2.5549990
H	-6.0237820	2.2844370	-0.2581060	H	3.4470310	-3.4042040	-1.6305050
H	-5.6391470	3.2300010	1.1805220	H	6.3407350	1.6319790	-1.7862580
H	-5.0572250	3.7625680	-0.3963360	H	7.4849180	1.4271610	-0.4599560
H	-1.0525840	5.8841150	2.3793130	H	7.2696430	0.1328910	-1.6394360
H	-1.4358400	4.1685160	2.1606500	H	-0.5083970	3.6211390	3.4820870
H	-0.0387980	4.6529130	3.1368910	H	-0.7759870	3.1332310	1.8004310
H	3.9867490	3.3038420	2.8023360	H	-0.0488750	2.0008130	2.9328600
H	3.9291230	3.6065000	1.0662890	H	4.8222420	2.5703100	2.0350040
H	3.0978530	4.6974120	2.1861990	H	5.5645410	4.0376460	1.3753850
H	1.6014290	3.4555190	-3.0842880	H	4.2575940	4.1619710	2.5568260
H	0.4068290	2.4533740	-2.2423210	H	1.6664460	4.8168530	-1.3170660
H	0.2822960	4.2183600	-2.1912200	H	0.0226520	4.3698160	-1.7758310
				H	0.3487660	4.9511430	-0.1428820

**II(Pc$_2$)$_2$ (dication)**				**II(Pc$_2$)$_2$ (protonated)**			
C	-0.9705530	-0.7292370	4.3608750	C	1.3978680	-0.1828160	2.1164400
C	-2.0170560	-1.6951380	3.7862830	C	0.6990210	0.7296540	3.1597800
C	-2.0460110	-3.0509360	4.4835330	C	1.2686990	0.0250780	4.4238570
C	-0.6557720	-3.7144430	4.4021380	C	0.8616450	-1.4289390	4.1137020
C	0.3213690	-2.7946600	3.6780580	C	0.8482200	-1.5438460	2.5505580
C	0.4368820	-1.3941490	4.3064170	C	-0.6886260	-1.5422130	4.1358610
C	-0.1163000	-2.4110960	2.2430110	C	-1.2943390	-0.1634610	4.4677470
C	-1.5499370	-1.7491150	2.3140270	C	-0.7973660	0.4263050	5.7813510
C	-0.5721700	0.2194040	3.1822050	C	0.7367100	0.5438080	5.7528400
C	-1.4795600	-0.2344840	2.0225120	C	-0.8874320	0.6102410	3.1820460
C	0.8437140	-0.4410960	3.1379130	C	-1.4725550	-0.3919220	2.1523940
C	0.9993020	-1.3800960	1.9332220	C	-0.7083450	-1.6496120	2.5706080
P	1.0251940	-0.5678970	0.3016670	P	-1.8590850	0.0795820	0.3745660
N	1.9613230	0.7489240	0.3559190	N	-3.3009250	0.8853600	0.7478360
P	3.5556990	0.7489130	0.1166120	P	-4.4335490	1.2157270	-0.3080840
C	4.0212840	0.6464360	-1.6399410	C	-5.5003740	-0.1854010	-0.8070820
C	3.4313320	1.7601770	-2.5088990	C	-6.2059610	-0.8723510	0.3630470
C	3.8606710	1.6231580	-3.9679230	P	1.6259840	0.3215150	0.3870010
P	-1.0324870	0.2027740	0.3054570	N	2.2736030	-0.8677740	-0.4792980
N	-1.8387390	-0.7396850	-0.7214180	P	3.5261300	-1.8391230	-0.5911730
P	-3.3738320	-0.6659600	-1.1989640	C	5.1296890	-1.0101040	-0.8177830
C	-3.6234030	0.3631370	-2.6811590	C	5.5332720	-0.1073710	0.3523500
C	-3.5109820	1.8704420	-2.4324130	N	2.4575710	1.7122500	0.5078180
C	-3.5385550	2.6680790	-3.7331590	P	2.2817670	2.9989620	-0.4323850
N	-1.1687950	1.7569710	0.0368270	C	2.6535730	2.6771710	-2.1879490
P	-0.6295960	3.2317590	0.2897920	C	4.1139560	2.2907910	-2.4313950
C	-2.1086870	4.2900920	0.3481310	C	0.5865020	3.6864380	-0.4650060
C	-3.0695120	3.9091710	1.4806050	C	-0.1964670	3.6076520	0.8495730
C	-4.4514440	4.5274290	1.2848220	C	3.4689760	4.2448400	0.1477130
C	0.3330200	3.4059010	1.8280400	C	3.5016920	5.5534990	-0.6471500
C	1.2343650	4.6377720	1.9795780	N	-2.3309640	-1.4317410	-0.2413120
C	0.5093140	5.9811750	2.0209510	P	-1.5479970	-2.3154470	-1.2944350
C	0.4193590	3.8166740	-1.0732780	C	-0.3846650	-3.5336930	-0.5674360
C	-0.2064060	3.6297420	-2.4587030	C	-0.9988160	-4.6214990	0.3164280
C	0.6815740	4.1882690	-3.5669800	C	-2.7493370	-3.2388700	-2.3150290
N	1.4202810	-1.6395600	-0.8083770	C	-3.9138260	-3.8590070	-1.5376990
P	0.8800800	-2.5150910	-2.0237710	C	-0.4400350	-1.4962320	-2.5053090
C	0.0220090	-1.5760980	-3.3299310	C	-0.9913070	-0.2590540	-3.2131380
C	0.5972320	-0.1704810	-3.5042130	C	-5.5297910	2.4736100	0.4299720
C	0.0206990	0.5589970	-4.7123680	C	-6.7998230	2.8190180	-0.3517260
C	2.3270000	-3.2655200	-2.8390530	C	-3.8798220	1.8807970	-1.9255210
C	3.4666850	-3.6683800	-1.8989910	C	-2.8724370	3.0222350	-1.7870600
C	4.6682500	-4.2077410	-2.6706240	C	3.7013600	-2.9644290	0.8335260
C	-0.2713940	-3.8182760	-1.4995980	C	4.9400490	-3.8662030	0.8411420
C	0.3455450	-4.9065550	-0.6148360	C	3.2668310	-2.8861700	-2.0530170
C	-0.7269400	-5.7546410	0.0654410	C	3.1434760	-2.0950680	-3.3594650
C	-3.8937750	-2.3383560	-1.6774330	H	-1.1069970	-2.5683350	2.1422050
C	-3.9264520	-3.3455210	-0.5244820	H	2.4647370	-0.1772040	2.3942870
C	-4.2077700	-4.7613120	-1.0197770	H	-2.5064280	-0.5742630	2.4726210

C	-4.5021800	-0.0449150	0.0886290
C	-5.9845500	0.0022560	-0.2962180
C	-6.8236560	0.6186220	0.8211830
C	4.2283740	2.3053990	0.7595240
C	4.1353830	2.4457020	2.2822680
C	4.7300710	3.7675680	2.7619700
C	4.4049980	-0.6284960	0.9571000
C	5.9334730	-0.6488500	0.8529460
C	6.5193060	-1.8488840	1.5943970
H	1.6838270	0.2360370	3.2951420
H	-2.4745450	0.2065880	2.1633390
H	1.9640010	-1.8997280	1.9891230
H	-3.0132290	-1.2407070	3.8221770
H	-2.2549560	-2.2761810	1.6702490
H	-1.2519450	-0.2637280	5.3064860
H	1.0301780	-1.3571620	5.2210390
H	1.3068120	-3.2700970	3.6321270
H	-0.1151250	-3.2620780	1.5606330
H	-0.5896860	1.2895430	3.3787800
H	-0.2669510	-3.9346110	5.4014050
H	-0.7084080	-4.6694300	3.8688220
H	-2.3535380	-2.9105440	5.5247720
H	-2.8084260	-3.6807180	4.0138170
H	-4.3552050	-0.6746020	0.9737320
H	-4.1427230	0.9588700	0.3478980
H	-4.6001700	0.1077050	-3.1074230
H	-2.8683050	0.0331530	-3.4036810
H	-4.8763580	-2.2632220	-2.1556660
H	-3.1900820	-2.6642110	-2.4529830
H	1.3582420	3.2571970	-0.9935610
H	0.6477130	4.8733910	-0.8922570
H	-2.6033550	4.1526130	-0.6202070
H	-1.8117230	5.3405060	0.4134340
H	-0.3832980	3.3540720	2.6548800
H	0.9409640	2.4944690	1.8555860
H	3.9806350	-1.5472600	0.5343380
H	4.0936920	-0.5913780	2.0075500
H	5.1151420	0.6513590	-1.6998620
H	3.6768290	-0.3351890	-1.9863950
H	5.2693730	2.3779430	0.4241390
H	3.6803920	3.1153070	0.2649970
H	-1.0551250	-3.2714020	-0.9621200
H	-0.7295020	-4.2537010	-2.3954700
H	-1.0347870	-1.5257860	-3.0605340
H	0.1094160	-2.1591920	-4.2540260
H	2.6906280	-2.5287280	-3.5659030
H	1.9652210	-4.1232950	-3.4181530
H	1.7875400	4.5044530	2.9149750
H	1.9868640	4.6454010	1.1831270
H	-0.3949240	2.5641000	-2.6272210
H	-1.1814300	4.1275660	-2.4986330
H	-3.1621490	2.8179660	1.5241190
H	-2.6522860	4.2289750	2.4415950
H	-6.1157840	0.5856540	-1.2140920
H	-6.3428720	-1.0102180	-0.5123770
H	-2.9667520	-3.3290060	0.0017640
H	-4.6884080	-3.0495140	0.2052130
H	-4.3334840	2.1974600	-1.7852660
H	-2.5852450	2.0724750	-1.8872440
H	3.7638420	-2.7935390	-1.3128390
H	3.1185160	-4.4182830	-1.1818500
H	0.4007680	0.4047640	-2.5917060
H	1.6863340	-0.2273420	-3.6056130
H	0.9920320	-5.5480620	-1.2228830
H	0.9886370	-4.4470310	0.1447060
H	3.0872550	2.3697690	2.5905350
H	4.6636970	1.6164810	2.7656740
H	6.2360550	-0.6896710	-0.1984470
H	6.3480900	0.2771950	1.2667040
H	2.3395610	1.7287370	-2.4337570
H	3.7470780	2.7395130	-2.1314710
H	-4.2153610	-5.4693350	-0.1870960
H	-5.1773620	-4.8163710	-1.5238580
H	-3.4397970	-5.0841210	-1.7301230
H	-3.5054120	3.7422010	-3.5294870
H	-2.6782840	2.4207610	-4.3638300
H	-4.4477570	2.4622130	-4.3065490
H	-7.8805350	0.6450010	0.5442550
H	-6.7289730	0.0408360	1.7457290
H	-6.5013280	1.6440440	1.0291760
H	5.0523160	-3.4575090	-3.3693390
H	5.4776290	-4.4783590	-1.9872490
H	4.4003220	-5.0983760	-3.2475170
H	0.4598310	1.5560610	-4.8083190
H	0.2230520	0.0098890	-5.6366390
H	-1.0638090	0.6772290	-4.6238360
H	-0.2749180	-6.5368750	0.6806760
H	2.3595070	0.1245640	4.4181510
H	1.0150040	1.7720860	3.0846870
H	1.4184640	-2.1834400	4.6732360
H	-1.1076620	-2.3709280	4.7107870
H	-2.3864830	-0.2276720	4.5003100
H	-1.3448300	1.6023380	3.1279720
H	1.3619980	-2.4001710	2.1151600
H	-1.1282130	-0.2105980	6.6091090
H	-1.2637080	1.4053100	5.9355690
H	1.1927410	-0.0286810	6.5678560
H	1.0557160	1.5831960	5.8853350
H	3.2496940	4.4358870	1.2022500
H	4.4459650	3.7478150	0.1173750
H	2.3604710	3.5454450	-2.7885240
H	1.9886060	1.8532970	-2.4762900
H	0.6530910	4.7194790	-0.8253400
H	0.0596550	3.1216560	-1.2434230
H	2.3440010	-3.4433240	-1.8557950
H	4.0799640	-3.6172550	-2.1134210
H	5.0543030	-0.4321220	-1.7453820
H	5.8817880	-1.7878740	-0.9956120
H	3.6799610	-2.3412990	1.7350810
H	2.7888290	-3.5730930	0.8473070
H	-0.1731710	-2.2672640	-3.2408320
H	0.4815150	-1.2444460	-1.9661440
H	0.1868780	-3.9914080	-1.3841360
H	0.3201230	-2.9197410	0.0091930
H	-3.1391850	-2.5112990	-3.0386780
H	-2.2063940	-3.9986010	-2.8897130
H	-3.4257860	1.0402730	-2.4633240
H	-4.7570170	2.1886260	-2.5064420
H	-4.8366100	-0.8946900	-1.3146160
H	-6.2273100	0.1778250	-1.5442020
H	-4.9134850	3.3680130	0.5775550
H	-5.7831930	2.1063190	1.4306390
H	0.3696620	0.5373320	-0.2060220
C	4.9105640	-4.8317760	2.0245380
H	4.9985040	-4.4360070	-0.0924930
H	5.8451700	-3.2514750	0.8934010
C	2.6575560	-2.9694320	-4.5120200
H	2.4469320	-1.2633880	-3.2107130
H	4.1141220	-1.6563510	-3.6157240
C	6.8351140	0.6377260	0.0656600
H	4.7313780	0.6136750	0.5488630
H	5.6474160	-0.7115060	1.2603190
C	4.5895340	6.4918700	-0.1283970
H	3.6769070	5.3454830	-1.7088240
H	2.5293680	6.0532600	-0.5815010
C	0.4214860	4.3760180	2.0135530
H	-1.2037000	3.9891350	0.6521760
H	-0.3279050	2.5558150	1.1246360
C	4.3271720	1.6824080	-3.8143720
H	4.7544360	3.1715930	-2.3114290
H	4.4221340	1.5763320	-1.6615820
C	-7.6104740	3.9121510	0.3425010
H	-6.5399340	3.1485810	-1.3641860
H	-7.4214730	1.9235400	-0.4646630
C	-7.1355670	-1.9916150	-0.1014270
H	-5.4477430	-1.2696710	1.0459970
H	-6.7864130	-0.1372250	0.9344640
C	-2.3147710	3.4812920	-3.1315460
H	-3.3392320	3.8709230	-1.2728780
H	-2.0576090	2.6759550	-1.1432910
C	-5.0280570	-4.3368710	-2.4659070
H	-3.5532600	-4.6977010	-0.9339320
H	-4.3012190	-3.1158580	-0.8333860
C	-0.0367110	0.2298160	-4.3011600
H	-1.9691080	-0.4765640	-3.6604080
H	-1.1501260	0.5301020	-2.4693740
C	0.0511710	-5.2840460	1.2064380
H	-1.7941730	-4.1947720	0.9376390
H	-1.4727300	-5.3784710	-0.3176610
H	0.6034110	5.4230910	1.7495880
H	-0.2454030	4.3570170	2.8798610
H	1.3724820	3.9334890	2.3257110
H	5.3754410	1.4087560	-3.9638550
H	3.7207810	0.7792330	-3.9394760
H	4.0446790	2.3869570	-4.6026120
H	5.5778230	6.0297090	-0.2154720
H	4.6011230	7.4259660	-0.6959370
H	4.4239110	6.7372010	0.9252480
H	-8.5173400	4.1473480	-0.2214340
H	-7.9092150	3.5976740	1.3476590
H	-7.0243020	4.8316030	0.4396470
H	-7.9231440	-1.6000320	-0.7537740
H	-6.5858470	-2.7518690	-0.6649330
H	-7.6158390	-2.4844080	0.7487810

H	-1.3614720	-5.1409170	0.7148260
H	-1.3749220	-6.2356500	-0.6736880
H	3.5756050	0.6477300	-4.3754550
H	3.3965680	2.3950560	-4.5880500
H	4.9462790	1.7184410	-4.0646980
H	4.6246630	3.8699720	3.8451210
H	5.7949920	3.8268270	2.5172760
H	4.2302030	4.6196130	2.2912010
H	7.6093320	-1.8601570	1.5155960
H	6.2557020	-1.8197610	2.6561620
H	6.1389590	-2.7869830	1.1777940
H	0.0926110	6.2566870	1.0475620
H	-0.3093910	5.9661920	2.7475990
H	1.2006710	6.7766260	2.3095220
H	-4.9071480	4.1691320	0.3557260
H	-5.1164660	4.2628850	2.1113080
H	-4.3930580	5.6189890	1.2324800
H	1.6612680	3.7013360	-3.5731940
H	0.2204490	4.0338880	-4.5464100
H	0.8437770	5.2623580	-3.4352620

H	-1.8118650	2.6555360	-3.6457150
H	-3.1115000	3.8469060	-3.7872310
H	-1.5878470	4.2887190	-3.0011200
H	0.4798390	-4.5587310	1.9070140
H	-0.3804610	-6.0995550	1.7931940
H	0.8702940	-5.6955170	0.6066360
H	-5.8393340	-4.8040010	-1.8995800
H	-5.4510680	-3.4984930	-3.0301240
H	-4.6555260	-5.0717740	-3.1870330
H	0.1216280	-0.5430410	-5.0606250
H	-0.4217030	1.1221720	-4.8031730
H	0.9423380	0.4804850	-3.8788790
H	5.7997610	-5.4674240	2.0318460
H	4.8756650	-4.2859340	2.9725870
H	4.0298070	-5.4800110	1.9769890
H	7.1126930	1.2751020	0.9094210
H	7.6582690	-0.0599170	-0.1182520
H	6.7327230	1.2758680	-0.8186780
H	3.3480790	-3.7977410	-4.6988680
H	1.6743800	-3.3974870	-4.2892940
H	2.5701820	-2.3854740	-5.4322130

II(Pd$_2$)$_2$ (dication)

C	-0.7504590	-3.7055860	-3.4912840
C	0.0632590	-4.5865650	-2.5287340
C	1.1556720	-5.3979060	-3.2175460
C	2.1111270	-4.4579130	-3.9821170
C	1.6605220	-3.0114730	-3.8147180
C	0.2119970	-2.7574920	-4.2675440
C	1.5703140	-2.5422200	-2.3426060
C	0.5944130	-3.5054860	-1.5562640
C	-1.2552490	-2.4970640	-2.6371740
C	-0.7491230	-2.8156330	-1.2200350
C	-0.2907240	-1.5475710	-3.4160560
C	0.9356300	-1.1554880	-2.5810350
P	0.6295540	-0.0957410	-1.1381680
P	-0.7035060	-1.4544330	-0.0118560
N	-0.1350930	-2.0759220	1.3470120
P	0.8839670	-1.6694500	2.5146190
C	2.5867690	-2.1247790	2.0858480
C	2.7395480	-3.6222770	1.7898680
C	3.9154810	-3.8965590	0.8515070
C	4.0184410	-5.3665570	0.4552420
N	1.9769830	0.0497400	-0.2626040
P	3.1264350	1.1645780	-0.4506090
C	2.6199970	2.7366540	0.3214910
C	3.2948110	4.0337400	-0.1296070
C	2.7860370	5.2240870	0.6861630
C	3.3542800	6.5544830	0.2014240
N	-0.0143450	1.2553900	-1.6760370
P	-1.2858860	2.2117680	-1.6973280
C	-2.7343010	1.4437610	-2.4837060
C	-4.0246110	2.2688070	-2.4529070
C	-5.1850010	1.5410110	-3.1322130
C	-6.4882790	2.3313970	-3.0580960
C	4.6108390	0.5406590	0.3860030
C	5.8038260	1.4992230	0.4189910
C	7.0472170	0.8294450	1.0056250
C	8.2405800	1.7786510	1.0733150
C	3.5788780	1.4662170	-2.1894290
C	4.2860390	0.2618940	-2.8278100
C	4.2636820	0.3125850	-4.3547810
C	5.0151190	-0.8590240	-4.9813890
C	-1.7900840	2.7886110	-0.0367310
C	-2.3949530	4.1991170	0.0422440
C	-3.1290350	4.4409830	1.3661110
C	-4.5200960	3.8095810	1.4026920
C	-0.8367240	3.6709890	-2.6801380
C	0.4947960	4.2817150	-2.2332200
C	0.8038170	5.6278110	-2.8917510
C	-0.0475200	6.7747020	-2.3503520
N	-2.1260380	-0.6955120	0.0626270
P	-3.3909090	-1.0847350	0.9821340
C	-3.1732820	-0.6803800	2.7415580
C	-2.8086490	0.7799770	3.0057550
C	-2.5779530	1.0675960	4.4889210
C	-2.1674020	2.5163580	4.7358670
C	-4.7826610	-0.1288860	0.3231650
C	-6.0422280	-0.0642630	1.1861560
C	-7.1289010	0.7765580	0.5139450
C	-8.4026100	0.8646840	1.3493670
C	-3.8224130	-2.8527770	0.9509130
C	-4.2207340	-3.3571710	-0.4412600
C	-4.0496570	-4.8690790	-0.5914320
C	-4.3014890	-5.3376090	-2.0218590
C	0.8084630	0.1034610	2.9512360
C	2.0652500	0.7370010	3.5493260

II(Pd$_2$)$_2$ (protonated)

C	-0.1349190	-1.4877610	-4.2069450
C	-0.6864250	-2.8029690	-3.6243980
C	-0.0360680	-4.0537510	-4.2019230
C	1.4853960	-4.0042550	-3.9719640
C	1.8560380	-2.7174840	-3.2467450
C	1.4023320	-1.4375080	-3.9764880
C	1.1485360	-2.5003860	-1.8807480
C	-0.4217800	-2.5474490	-2.1147970
C	-0.3440440	-0.4074140	-3.0868550
C	-1.1243680	-1.1706810	-2.0149370
C	1.1995410	-0.3543370	-2.8623950
C	1.6971990	-1.0766000	-1.6077500
P	1.7788780	-0.3460490	0.0641430
P	-1.6452070	-0.4447480	-0.3671130
N	-2.9162400	-1.5359410	-0.0754930
P	-3.0959710	-2.6042430	1.0759950
C	-4.6140940	-3.5361790	0.6893140
C	-5.0948240	-4.5558970	1.7255600
C	-6.3030600	-5.3660230	1.2418220
C	-7.5397150	-4.5187520	0.9447630
N	2.7899270	-1.2927740	0.8982900
P	4.3231140	-0.9802910	1.2280290
C	5.2801590	-0.4590240	-0.2335700
C	6.7638060	-0.1569080	-0.0085000
C	7.5203760	-0.0429240	-1.3333490
C	8.9644570	0.4132470	-1.1473640
N	2.2770760	1.1866870	-0.0677630
P	1.5376420	2.5983710	-0.1592770
C	0.9519500	2.9972880	-1.8413840
C	0.6513690	4.4687860	-2.1404770
C	-0.0037450	4.6363300	-3.5129560
C	-0.1510240	6.0989130	-3.9238450
C	4.5262080	0.3368340	2.4711470
C	3.6092610	0.1626190	3.6839450
C	3.6544880	1.3635810	4.6271410
C	2.7400040	1.1783300	5.8349650
C	5.0308400	-2.5023480	1.9276140
C	4.9465010	-3.7248990	0.9990850
C	6.1522760	-3.9005590	0.0736170
C	5.9852400	-5.0931330	-0.8646220
C	0.0625440	2.7604110	0.8900980
C	0.3321670	2.5377980	2.3917360
C	-0.0137320	3.7782690	3.2200640
C	0.4258630	3.6879430	4.6773580
C	2.7107900	3.8795340	0.3947110
C	4.1698880	3.6638250	-0.0194320
C	4.4231470	3.5450480	-1.5218610
C	5.9165890	3.5232980	-1.8391020
N	-2.3498240	0.9812260	-0.9411660
P	-3.8618910	1.4196480	-0.7860580
C	-4.0411480	3.1190610	-1.4283840
C	-2.8733610	4.0396080	-1.0646110
C	-3.0141970	5.4547770	-1.6281750
C	-4.1907520	6.2403770	-1.0508700
C	-5.0995480	0.4068980	-1.6809090
C	-4.7701550	0.1964610	-3.1607240
C	-5.5958620	-0.9152870	-3.8178800
C	-5.2990360	-2.3115250	-3.2696120
C	-4.5296610	1.3932630	0.9266350
C	-3.7822800	2.2689890	1.9461060
C	-4.4037340	3.6528870	2.1713930
C	-5.6918580	3.6026880	2.9916900
C	-1.7882990	-3.8838590	1.2342140
C	-0.3652990	-3.3488670	1.4218560

C	1.8186870	2.1895320	3.9613650	C	0.7156820	-4.4206220	1.2652860
C	3.1057160	2.9105830	4.3532830	C	0.6746660	-5.5062850	2.3387050
C	0.4086100	-2.6322940	3.9785430	C	-3.3816040	-1.9543690	2.7694140
C	1.2357440	-2.3623830	5.2382190	C	-2.4068670	-0.8734600	3.2400730
C	0.8332140	-3.2853950	6.3893550	C	-2.8096170	-0.3011630	4.5993430
C	1.6367140	-3.0154610	7.6587970	C	-1.8884350	0.8245710	5.0581170
H	-2.3110180	-2.2328300	-2.7003030	H	-0.8032280	0.5393570	-3.3758920
H	1.6099120	-0.5565830	-3.2061470	H	2.7876370	-1.1534780	-1.7386620
H	-1.4047220	-3.5616640	-0.7535690	H	-2.1303300	-1.3235290	-2.4281720
H	2.3437020	-2.3470360	-4.3557800	H	2.9399420	-2.7015960	-3.0860550
H	2.5442560	-2.4890670	-1.8503570	H	1.4605050	-3.2280430	-1.1283190
H	0.0788880	-2.7062400	-5.3489410	H	2.0177390	-1.1676950	-4.8373050
H	-1.4867320	-4.2466960	-4.0878490	H	-0.4836420	-1.2573810	-5.2161340
H	-0.6034270	-5.2598040	-1.9786140	H	-1.7688500	-2.8621220	-3.7799880
H	1.0862810	-3.9297320	-0.6796610	H	-0.8809920	-3.3119780	-1.4823920
H	-0.7645450	-0.7146990	-3.9355460	H	1.6713200	0.6119810	-3.0334220
H	0.6902800	-6.1201440	-3.8958470	H	-0.2709400	-4.1219370	-5.2698520
H	1.6982670	-5.9797210	-2.4651490	H	-0.4759300	-4.9383860	-3.7287950
H	2.1352980	-4.7049820	-5.0481930	H	2.0300930	-4.0493490	-4.9212960
H	3.1373540	-4.5579670	-3.6133500	H	1.8229260	-4.8605890	-3.3779380
H	-2.4785650	2.0249760	0.3375610	H	-0.3369110	3.7666580	0.7122790
H	-0.9005990	2.7285000	0.5998280	H	-0.6815700	2.0659900	0.4802060
H	-1.5908550	4.9325610	-0.0769700	H	-0.2505420	1.6787640	2.7410550
H	-3.0965190	4.3750360	-0.7811160	H	1.3820650	2.2657720	2.5692880
H	-2.5189550	4.0598020	2.1938990	H	0.4581500	4.6544330	2.7567150
H	-3.2264310	5.5196730	1.5249860	H	-1.0968180	3.9503890	3.1614240
H	-4.4853440	2.7342440	1.1990300	H	0.1286580	4.5812930	5.2337590
H	-4.9913800	3.9371710	2.3810570	H	-0.0133460	2.8198980	5.1768450
H	-5.1732150	4.2610890	0.6487650	H	1.5143670	3.5950820	4.7485430
H	-2.4409820	1.2202400	-3.5157490	H	1.7145430	2.6344580	-2.5357230
H	-2.8659540	0.4873460	-1.9648590	H	0.0570920	2.3757680	-1.9857410
H	-4.3061320	2.4900810	-1.4157940	H	0.0002300	4.9017870	-1.3708030
H	-3.8715340	3.2374500	-2.9443940	H	1.5841120	5.0443810	-2.1180110
H	-4.9240820	1.3453080	-4.1790000	H	0.5971200	4.1043680	-4.2608340
H	-5.3204410	0.5600880	-2.6604010	H	-0.9865030	4.1482600	-3.5042920
H	-6.7771240	2.5120880	-2.0173700	H	-0.6436440	6.1874650	-4.8961430
H	-6.3861970	3.3039260	-3.5504580	H	-0.7468560	6.6572340	-3.1951730
H	-7.3057640	1.7918880	-3.5439500	H	0.8278190	6.5841980	-3.9967090
H	-0.7785950	3.3506230	-3.7256460	H	2.3373250	4.8469540	0.0428290
H	-1.6578730	4.3907210	-2.6095850	H	2.6437040	3.8981400	1.4881880
H	0.4989870	4.4172840	-1.1436150	H	4.7525470	4.5012830	0.3814690
H	1.2872300	3.5626270	-2.4590740	H	4.5487090	2.7601400	0.4698080
H	1.8622860	5.8583200	-2.7246160	H	3.9602050	2.6233910	-1.8946800
H	0.6752000	5.5402150	-3.9771850	H	3.9443800	4.3791370	-2.0505190
H	0.2091500	7.7186780	-2.8384760	H	6.4201960	2.7274770	-1.2790380
H	-1.1166150	6.6018190	-2.5112170	H	6.0987650	3.3520190	-2.9035410
H	0.1130960	6.8992570	-1.2735810	H	6.3905160	4.4704760	-1.5620770
H	1.5401370	2.7935820	0.1491070	H	5.1508290	-1.2541460	-0.9774950
H	2.7552170	2.5916760	1.3989510	H	4.7573340	0.4222770	-0.6254020
H	4.3831200	3.9647970	-0.0287660	H	6.8653560	0.7776340	0.5566830
H	3.0926200	4.2085620	-1.1922750	H	7.2329670	-0.9406610	0.5994680
H	1.6895160	5.2532950	0.6389010	H	7.4977380	-1.0166380	-1.8387790
H	3.0422690	5.0687780	1.7416160	H	6.9925200	0.6562080	-1.9932960
H	2.9875750	7.3849730	0.8104960	H	9.0036430	1.4077680	-0.6903140
H	4.4479120	6.5566750	0.2510740	H	9.5131560	-0.2752300	-0.4961660
H	3.0662200	6.7481560	-0.8373240	H	9.4908060	0.4623490	-2.1045160
H	4.2048420	2.3621020	-2.2515050	H	4.4505000	-2.6762670	2.8395710
H	2.6422570	1.6964810	-2.7121480	H	6.0618750	-2.3064850	2.2416810
H	5.3245600	0.2242610	-2.4792050	H	4.8527750	-4.6221800	1.6206120
H	3.8182960	-0.6742750	-2.4972970	H	4.0242420	-3.6699470	0.4073070
H	3.2211180	0.3126910	-4.6995260	H	6.3138420	-2.9954500	-0.5222380
H	4.6986320	1.2602610	-4.6933100	H	7.0552260	-4.0275720	0.6833270
H	4.9617680	-0.8252520	-6.0726870	H	6.8613120	-5.2197050	-1.5065340
H	6.0713080	-0.8450550	-4.6939750	H	5.8446190	-6.0204530	-0.2993920
H	4.5955350	-1.8165740	-4.6533520	H	5.1104040	-4.9606640	-1.5103350
H	4.8724320	-0.3953590	-0.1206600	H	4.2792230	1.2691760	1.9506700
H	4.3153760	0.2677900	1.4036380	H	5.5795500	0.3893730	2.7685400
H	6.0338110	1.8614650	-0.5909370	H	2.5793580	0.0139720	3.3369470
H	5.5538000	2.3794330	1.0225170	H	3.8859430	-0.7426520	4.2383640
H	6.8126030	0.4547600	2.0093430	H	4.6856880	1.5298290	4.9615670
H	7.3023660	-0.0467110	0.3973220	H	3.3617120	2.2645210	4.0714900
H	9.1178850	1.2798290	1.4938640	H	1.7048100	1.0142930	5.5168980
H	8.5073070	2.1448600	0.0765180	H	3.0486250	0.3123350	6.4296420
H	8.0151460	2.6479730	1.6996420	H	2.7531440	2.0572820	6.4856000
H	0.5456350	0.6284440	2.0271370	H	-2.0681240	-4.5510340	2.0567310
H	-0.0384910	0.2121740	3.6375850	H	-1.8438820	-4.4759860	0.3115190
H	2.4233880	0.1699720	4.4152180	H	-0.1817540	-2.5567470	0.6898240
H	2.8708590	0.7028590	2.8081660	H	-0.2667220	-2.8829090	2.4090670
H	1.3310990	2.7240070	3.1349390	H	0.6199900	-4.8814580	0.2728010
H	1.1088080	2.2089490	4.7967560	H	1.6894760	-3.9175310	1.2838790
H	2.9058190	3.9443150	4.6472670	H	-0.2554440	-6.0820900	2.2976560
H	3.5970450	2.4085670	5.1927130	H	1.5033530	-6.2097110	2.2164240
H	3.8172980	2.9306360	3.5192570	H	0.7516830	-5.0671940	3.3394220
H	2.7917690	-1.5380440	1.1856290	H	-5.3744720	-2.7675650	0.5114830
H	3.2734300	-1.7945070	2.8718230	H	-4.4304160	-4.0228430	-0.2761640
H	2.8674850	-4.1807950	2.7243150	H	-4.2837880	-5.2508960	1.9703770
H	1.8222220	-4.0022210	1.3241470	H	-5.3593980	-4.0401540	2.6566690

H	3.7952660	-3.2826240	-0.0505030
H	4.8471310	-3.5660520	1.3263130
H	4.8650880	-5.5379870	-0.2150950
H	4.1500660	-6.0048830	1.3349570
H	3.1092490	-5.6939490	-0.0614640
H	-0.6511360	-2.4269960	4.1641980
H	0.4773210	-3.6855350	3.6838040
H	2.3042790	-2.4969200	5.0281860
H	1.1022710	-1.3208120	5.5544740
H	-0.2370080	-3.1585990	6.5928780
H	0.9697700	-4.3278130	6.0772630
H	1.3341050	-3.6858680	8.4677850
H	2.7075660	-3.1617490	7.4833990
H	1.4920910	-1.9861130	8.0030310
H	-4.0950800	-0.9586490	3.2646830
H	-2.3869900	-1.3515870	3.1043050
H	-1.9098490	1.0404930	2.4351880
H	-3.6089580	1.4349880	2.6421060
H	-3.4927350	0.8375150	5.0472650
H	-1.8074280	0.3901130	4.8798640
H	-1.9706900	2.6990680	5.7955630
H	-1.2588300	2.7670570	4.1768760
H	-2.9564250	3.2055650	4.4167970
H	-4.6125650	-3.0354160	1.6862750
H	-2.9275640	-3.3726290	1.3170630
H	-5.2601170	-3.0807460	-0.6503890
H	-3.6128840	-2.8582650	-1.2039380
H	-3.0309780	-5.1483060	-0.2913240
H	-4.7244960	-5.3839030	0.1022430
H	-4.1834690	-6.4207930	-2.1105260
H	-5.3138150	-5.0801410	-2.3495510
H	-3.5953810	-4.8645400	-2.7135500
H	-5.0042110	-0.5578280	-0.6607400
H	-4.3934630	0.8768640	0.1350330
H	-6.4239640	-1.0747050	1.3757480
H	-5.8062650	0.3726220	2.1642980
H	-6.7356040	1.7852690	0.3331280
H	-7.3561590	0.3511640	-0.4713390
H	-9.1602830	1.4732900	0.8485960
H	-8.8283240	-0.1291610	1.5214560
H	-8.1995710	1.3159980	2.3260130

H	-6.0199970	-5.9302540	0.3444300
H	-6.5492700	-6.1082490	2.0093950
H	-7.8137480	-3.9088560	1.8126410
H	-7.3752390	-3.8428800	0.0997860
H	-8.3957230	-5.1521300	0.6957130
H	-4.4012610	-1.5466980	2.7612350
H	-3.3875870	-2.7996800	3.4679070
H	-1.3900930	-1.2768470	3.3069140
H	-2.3639930	-0.0696000	2.4958380
H	-3.8423320	0.0680420	4.5432920
H	-2.8129310	-1.1070950	5.3433520
H	-2.1610430	1.1887940	6.0526310
H	-0.8464350	0.4870720	5.0965860
H	-1.9372740	1.6731850	4.3671430
H	-4.1219170	3.0479040	-2.5194370
H	-5.0003850	3.5038940	-1.0646090
H	-2.7598090	4.0993020	0.0236030
H	-1.9556580	3.5754820	-1.4363140
H	-2.0875440	6.0012590	-1.4169810
H	-3.0998620	5.4054870	-2.7214570
H	-5.1509310	5.7773400	-1.2986990
H	-4.1201640	6.2995090	0.0408720
H	-4.2090500	7.2619960	-1.4408170
H	-4.4722290	0.3361980	1.2053130
H	-5.5943630	1.6504860	0.8833110
H	-3.7655620	1.7500910	2.9105740
H	-2.7326110	2.3666920	1.6420330
H	-3.6739200	4.2882830	2.6870630
H	-4.6060890	4.1378630	1.2098600
H	-6.4584790	2.9921540	2.5033710
H	-5.5050530	3.1715420	3.9810320
H	-6.1087670	4.6038760	3.1325920
H	-6.0920930	0.8570870	-1.5573070
H	-5.0882690	-0.5484720	-1.1462580
H	-4.9296960	1.1325270	-3.7086450
H	-3.7048390	-0.0448230	-3.2600220
H	-6.6647210	-0.6938780	-3.7044840
H	-5.3917840	-0.9048580	-4.8947910
H	-4.2277370	-2.5347160	-3.3322450
H	-5.5920100	-2.4101610	-2.2198630
H	-5.8375920	-3.0773070	-3.8352220
H	0.5279640	-0.4213060	0.6861110

II(Pe_2)$_2$ (dication)

C	0.3469860	-2.2637760	5.6661480
C	1.0985780	-1.4690910	4.6038200
C	0.4771020	-1.5708730	3.1896350
C	-1.0278380	-1.1110040	3.2714430
C	-1.3650940	-0.6876990	4.7174950
C	-1.1125790	-1.7721210	5.7599740
C	-1.2193350	0.2441320	2.5590590
C	-0.2980410	1.1320770	3.4209620
C	1.1886660	0.6694970	3.3877050
C	1.3776130	-0.5377320	2.4636130
C	-0.4399100	0.5286170	4.8608520
C	1.0439160	0.0545260	4.8110430
H	1.9171420	1.4642320	3.2476080
H	-2.2570000	0.5697310	2.7019720
H	2.4123420	-0.8883100	2.5322110
H	-2.4145090	-0.3748680	4.7563480
H	-1.6902620	-1.8910330	2.8932160
H	-0.7054600	1.2116540	5.6690360
H	1.7035030	0.4291110	5.5949620
H	2.1400930	-1.8068730	4.5592190
H	0.5497630	-2.5749750	2.7779020
H	-0.4501840	2.2049470	3.3093780
H	0.8602850	-2.1550210	6.6267180
H	0.3851410	-3.3281850	5.4097190
H	-1.3270690	-1.3685600	6.7548260
H	-1.8139950	-2.5969320	5.5984180
P	1.1754810	-0.1445620	0.6837280
N	1.8088290	1.3164530	0.4226910
N	1.7604400	-1.2983440	-0.2323920
P	1.8076970	-2.7101510	-0.9591500
C	3.4092070	-2.8449450	-1.8312130
C	4.6589740	-2.8330700	-0.9013900
C	3.5549330	-4.1096720	-2.7214080
C	5.0198860	-4.5334590	-2.5546040
H	4.8382360	-4.9518690	-0.4430850
C	5.3145070	-4.2055170	-1.0904100
H	6.3805680	-4.1918050	-0.8519610
H	5.6615700	-3.9300860	-3.2066340
H	5.1794270	-5.5846380	-2.8056610
H	3.2850490	-3.9036280	-3.7592790
H	2.9104170	-4.9244910	-2.3767110
H	4.4307190	-2.6035100	0.1418600
H	5.3452340	-2.0565680	-1.2541550

II(Pe_2)$_2$ (protonated)

C	0.4225630	-2.5060130	5.4253210
C	1.0978980	-1.5628170	4.4385020
C	0.5919520	-1.6733760	2.9705940
C	-0.9718300	-1.4383770	2.9635800
C	-1.4435520	-1.1713050	4.4179790
C	-1.0973160	-2.2573680	5.4290150
C	-1.3784320	-0.0828080	2.3521400
C	-0.5974030	0.8623490	3.2728060
C	0.9484730	0.6395620	3.3179800
C	1.4232910	-0.4961510	2.4088350
C	-0.7270880	0.1679970	4.6763230
C	0.8096960	-0.0715660	4.7051380
H	1.5608500	1.5421600	3.2684610
H	-2.4341380	0.0653710	2.6135190
H	2.4532480	-0.7154660	2.7163130
H	-2.5291320	-1.0258900	4.4064320
H	-1.4954670	-2.2794590	2.5024180
H	-1.1413470	0.7534420	5.5003340
H	1.3575790	0.3478840	5.5516370
H	2.1784970	-1.7478460	4.4565960
H	0.8569730	-2.6264340	2.5083530
H	-0.9098340	1.9052200	3.2057070
H	0.8510100	-2.3488610	6.4211710
H	0.6524750	-3.5401600	5.1467160
H	-1.4432250	-1.9507240	6.4224330
H	-1.6449000	-3.1719370	5.1766070
P	1.8344280	-0.1470970	0.6720930
N	3.1276110	0.8042970	0.7517940
N	1.9921950	-1.5441780	-0.1148070
P	1.2694920	-2.2107470	-1.3762190
C	-0.2201350	-3.1954740	-0.9541310
C	-0.7100680	-4.1291950	-2.0980930
C	-0.0225910	-4.1166470	0.2836500
C	-0.5381980	-5.5039450	-0.1367420
H	0.7062080	-5.7643540	-1.8952950
C	-0.3402220	-5.5470800	-1.6529520
H	-0.9519350	-6.3082680	-2.1442270
H	-1.6062820	-5.5889000	0.0914620
H	-0.0248700	-6.3142310	0.3876890
H	-0.5708740	-3.7231120	1.1417650
H	1.0282900	-4.1698400	0.5827120
H	-0.3132230	-3.8815740	-3.0850830
H	-1.7973080	-4.0444160	-2.1737360

C	0.2217230	-4.3033550	0.7129580
C	0.3623400	-5.0940670	2.0306380
H	0.0852720	-6.1403660	1.8783720
H	-0.4100590	-4.8231700	-0.0114910
H	-0.2342100	-3.3217160	0.8862440
C	1.6587950	-4.1308320	0.1870100
C	1.8460910	-4.9830330	2.4589880
C	2.5218710	-4.0288670	1.4579840
H	-0.5073300	-4.8780270	-4.7286620
H	3.5639640	-4.2996100	1.2802120
H	1.2024170	-4.4869090	-4.5460630
H	2.3252820	-5.9646690	2.4134860
C	0.6851220	-1.9802490	-3.4582960
C	0.4526510	-2.8406800	-2.1873570
C	0.0749530	-4.2636910	-2.7030100
H	0.6818980	-5.0676530	-2.2815850
H	2.5123060	-3.0014380	1.8330830
H	-0.9594240	-4.4785700	-2.4159950
H	0.3035720	-2.4492690	-5.5652410
H	0.3430120	-0.9505840	-3.3266490
H	-1.1271420	-2.5105200	-4.5333710
H	1.7465060	-1.9339440	-3.7214190
C	3.2668690	0.7736360	-2.7713090
C	3.3781010	1.3480500	-4.1840340
C	3.2248710	2.0418280	-1.9089870
P	3.2670140	1.7593200	-0.1023580
H	3.7255390	0.6111290	-4.9117470
H	2.3918760	1.6925300	-4.5122670
H	4.1652460	0.1877830	-2.5420960
C	3.7169400	3.3673960	0.6257220
H	1.6790280	4.0131370	0.0794120
C	2.6252780	4.4534050	0.4003320
H	2.9405090	5.1469570	-0.3829300
H	4.5682120	5.4503500	2.2077960
C	2.4629740	5.1648640	1.7613080
H	2.3027910	6.2400970	1.6515840
H	1.5950820	4.7556270	2.2925070
C	3.7351000	4.8203140	2.5398040
H	3.6243510	4.9488630	3.6191180
C	3.9917300	3.3659700	2.1416060
H	4.9971890	3.0140010	2.3839520
H	3.2721240	2.7252690	2.6596380
C	4.3426130	2.5435360	-4.0356610
C	4.5907780	0.5435890	0.2181140
H	5.3611190	2.2445740	-4.2969440
H	5.2636090	2.8388580	-2.0770400
C	4.2841640	2.9702220	-2.5427490
H	4.0682850	3.3654220	-4.7010570
C	4.7576660	0.0826930	1.6979470
H	4.1583950	0.6564140	2.4076850
H	4.4342340	-0.9591720	1.7793840
H	6.6024470	-0.5773400	2.6916830
C	6.2630200	0.1954680	1.9984460
H	6.4850730	1.1668560	2.4543650
C	6.9313470	0.1135440	0.6250170
H	7.9601370	0.4811430	0.6221160
C	6.0045940	0.9552630	-0.2566410
H	6.1502990	0.7838330	-1.3263030
H	6.1972560	2.0163560	-0.0609440
H	4.0084880	4.0203500	-2.4184330
H	6.9435070	-0.9242720	0.2713850
H	-0.3107590	-4.7013430	2.7987990
H	1.9553040	-4.6205010	3.4847740
C	0.1925580	-4.2003640	-4.2343150
C	-0.0554500	-2.7290810	-4.5718960
H	2.3964560	0.1326860	-2.6159600
H	4.2609260	-0.3192020	-0.3664290
H	4.6328510	3.6739790	0.1094840
H	2.2451760	2.5045830	-2.0618810
H	-0.3876230	-2.4232660	-1.6195930
H	3.4351330	-1.9533840	-2.4614660
H	1.9524920	-5.0287290	-0.3693270
P	-1.0351500	0.1446470	0.7343910
N	-1.6522190	-1.2660680	0.2599330
N	-1.6829230	1.4000940	0.0106990
P	-1.6093280	2.9231120	-0.4441230
C	-0.3679250	3.0900970	-1.7637850
C	-0.4506550	1.9493450	-2.8230920
C	-0.2773290	4.4122330	-2.5543820
C	0.3616190	3.9846820	-3.8816740
H	-1.3126220	2.8330720	-4.6120200
C	-0.3193590	2.6471430	-4.1888190
H	0.2266860	2.0389740	-4.9142340
H	1.4413280	3.8428910	-3.7469370
H	0.2253460	4.7274400	-4.6710350
H	0.3007910	5.1723660	-2.0244610
H	-1.2693580	4.8301680	-2.7520780

C	0.3826120	-1.4763730	-4.0251930
C	0.6404690	-0.2564840	-4.9149360
H	-0.2090740	0.4321940	-4.8522290
H	-0.6334220	-1.8651740	-4.1235050
H	1.0717000	-2.2789360	-4.3112970
C	0.6812360	-0.9646080	-2.5940420
C	1.8790620	0.3810760	-4.2832210
C	1.6274240	0.2603120	-2.7728180
H	4.3887320	-5.3283730	-2.5977910
H	1.1231530	1.1569530	-2.4012470
H	5.2537550	-4.9826560	-1.0923710
H	2.0432560	1.4155650	-4.5947270
C	3.6223850	-2.6342230	-2.9351650
C	2.4729300	-3.3468780	-2.1592770
C	3.1748640	-4.2793230	-1.1422840
H	3.3367420	-3.7492830	-0.1981830
H	2.5514280	0.1765250	-2.1977790
H	2.5890430	-5.1752910	-0.9250950
H	5.3299390	-2.6045840	-1.5952930
H	3.5134560	-2.8275250	-4.0056710
H	5.7020020	-3.3190380	-3.1650550
H	3.6110920	-1.5496470	-2.8139470
C	4.4990710	3.5808790	0.6453780
C	5.5559200	4.6612220	0.3332510
C	5.2950710	2.3824530	1.2395240
P	4.7126310	0.7564590	0.6633320
H	5.8010410	4.6473660	-0.7348790
H	5.2021590	5.6673130	0.5715450
H	3.9275040	3.3021060	-0.2426260
C	5.5438090	-0.4874520	1.7134210
H	4.2193150	0.0987180	3.3585070
C	5.2159340	-0.3368920	3.2179490
H	5.9273930	0.3186690	3.7259270
H	4.8883280	-3.6623130	2.6764040
C	5.2369010	-1.7744170	3.7520120
H	6.2722700	-2.0868150	3.9293660
H	4.6928400	-1.8787420	4.6950840
C	4.6320570	-2.6018690	2.6130250
H	3.5383310	-2.5354820	2.6341500
C	5.1694710	-1.9500930	1.3258920
H	6.0614400	-2.4720930	0.9684950
H	4.4212810	-1.9961020	0.5293600
C	6.7926490	4.2524730	1.1430140
C	5.2517890	0.3827940	-1.0424540
H	7.7180080	4.6975840	0.7687540
H	7.1247290	2.4393890	0.0360490
C	6.7844670	2.7241810	1.0367320
H	6.6758780	4.5506100	2.1915350
C	6.6916330	-0.1082330	-1.2797400
H	7.4101680	0.5291350	-0.7570060
H	6.8477240	-1.1263890	-0.9155180
H	6.8676510	-0.9750820	-3.2834300
C	6.8842330	0.0110670	-2.8122890
H	7.8532630	0.4601960	-3.0425210
C	5.7164460	0.8870180	-3.3310060
H	6.0421370	1.6439640	-4.0480200
C	5.0863210	1.5065260	-2.0770500
H	4.0482910	1.8162350	-2.2216960
H	5.6557760	2.3898680	-1.7664990
H	7.4321820	2.2277400	1.7639020
H	4.9728660	0.2615580	-3.8369410
H	0.7779440	-0.5243590	-5.9654060
H	2.7673020	-0.1950650	-4.5674830
C	4.5213760	-4.5871650	-1.8010840
C	4.9316040	-3.2382880	-2.3942660
H	3.7711680	3.9295770	1.3806680
H	4.5651100	-0.4311720	-1.3143970
H	6.6176960	-0.3310230	1.5604670
H	5.1010560	2.3456200	2.3184820
H	1.9053160	-3.9623270	-2.8639010
H	-0.9825560	-2.4402540	-0.7265060
H	-0.2452520	-0.6113730	-2.1241890
P	-1.5572920	0.0882390	0.4945980
N	-2.7745980	-1.0678140	0.3543080
N	-2.2858640	1.6147080	0.4947870
P	-1.9901910	2.9072500	-0.3620120
C	-0.2114690	3.3511960	-0.5320560
C	0.0994210	4.6149920	-1.3751060
C	0.5239740	3.5578320	0.8271780
C	1.0186730	5.0094430	0.7938600
H	1.4421920	6.3016870	-0.9451770
C	1.3149760	5.2468960	-0.6876940
H	2.2310820	4.7172640	-0.9745580
H	1.8813050	5.1762680	1.4443290
H	0.2225390	5.6901640	1.1209430
H	1.3817870	2.8787680	0.8760800
H	-0.1013130	3.3417320	1.6949950

H	-1.3693800	1.3630930	-2.7486570	H	-0.7262530	5.3320940	-1.3288690
H	0.3712240	1.2482060	-2.6500310	H	0.2631290	4.3792100	-2.4303040
C	-3.6837970	2.8798940	-2.4099860	C	-1.8477120	1.9206740	-3.0286400
C	-5.0827210	3.4814120	-2.6050170	C	-2.4201000	2.2850570	-4.3992210
H	-5.8338320	2.7927820	-2.2052640	H	-3.4216000	1.8500230	-4.5088890
H	-3.6739260	1.7953490	-2.5142060	H	-1.9126240	0.8573320	-2.7808920
H	-2.9912230	3.2888730	-3.1527200	H	-0.7873300	2.1984460	-3.0052240
C	-3.2923690	3.3736110	-1.0067900	C	-2.6677760	2.8104050	-2.0726300
C	-5.0783370	4.8046480	-1.7963180	C	-2.5061270	3.8130760	-4.3252650
C	-3.7199900	4.8529740	-1.0617810	C	-2.9545640	4.1132290	-2.8776260
H	-0.7072300	4.7163850	3.5487250	H	-4.0369880	5.3060980	3.4164360
H	-3.7952870	5.3195850	-0.0776700	H	-4.0240790	4.3342760	-2.8424400
H	-2.2959590	5.4627250	3.7404630	H	-4.4359120	3.6415320	2.9888000
H	-5.8984040	4.8151490	-1.0738360	H	-3.1835040	4.2411650	-5.0680380
C	-0.8346020	5.4854440	0.6736170	C	-4.3978570	4.2263320	0.2495490
C	-1.1226680	3.9910870	0.9476140	C	-2.8671910	4.3064140	0.4354810
C	-2.1402760	4.0054980	2.1030360	C	-2.6535960	4.3634380	1.9744650
H	-3.1330740	4.2932650	1.7400630	H	-2.2316470	3.4247570	2.3388600
H	-3.0031870	5.4354030	-1.6480820	H	-2.4417860	4.9902920	-2.4797510
H	-2.2429130	3.0386790	2.5995470	H	-1.9604590	5.1689040	2.2320350
H	-1.9446210	6.9287520	1.8754750	H	-6.0046480	4.8658370	1.6063620
H	0.1952220	5.6365580	0.3478650	H	-4.7264990	4.6002720	-0.7224660
H	-0.2676450	6.7582690	2.3815580	H	-4.8024840	6.1088310	1.2315790
H	-1.4727080	5.8789210	-0.1194780	H	-4.7296870	3.1860520	0.3455200
C	-5.7973950	-1.7534940	1.1532950	C	-5.3903340	-2.8260820	1.6056030
C	-6.7957660	-0.6803940	1.6610730	C	-5.3922200	-4.3428620	1.8008820
C	-4.3984760	-1.0764200	1.1958180	C	-5.0092130	-2.6575070	0.1189290
P	-3.1909830	-1.6286220	-0.0709210	P	-4.2316420	-1.0356170	-0.2371550
H	-7.4581590	-0.3770790	0.8446610	H	-5.3840760	-4.6363180	2.8542000
H	-7.4275420	-1.0600780	2.4668860	H	-6.2858980	-4.7777540	1.3373160
H	-6.0675140	-2.0619560	0.1415760	H	-4.6140010	-2.3847800	2.2418890
C	-3.6754040	-0.8621670	-1.6489860	C	-5.3481460	0.2995590	0.3708330
H	-2.7863710	-2.3908350	-2.9062230	H	-7.1539900	-0.9262860	-0.0927810
C	-2.8015530	-1.2967420	-2.8309640	C	-6.8819320	0.0964200	0.1819800
H	-1.7710720	-0.9555620	-2.7211910	H	-7.2427270	0.7407340	-0.6243520
H	-5.3049930	0.5228510	-3.7227150	H	-6.4855480	2.3918880	1.7739200
C	-3.5110350	-0.6859580	-4.0525240	C	-7.5202890	0.5012640	1.5286270
H	-3.0717240	0.2887520	-4.2886200	H	-8.4849700	0.9990230	1.3986510
H	-3.3874840	-1.3110980	-4.9399610	H	-7.6939140	-0.3895750	2.1433020
C	-5.0000170	-0.5250500	-3.6477170	C	-6.4670790	1.3830690	2.2069750
H	-5.6662540	-1.0967050	-4.2973690	H	-6.6202790	1.4830600	3.2852960
C	-5.1087430	-1.0062330	-2.1834850	C	-5.1510610	0.6892750	1.8502090
H	-5.8368590	-0.4277280	-1.6092710	H	-4.2639260	1.3165000	1.9606710
H	-5.4246430	-2.0529350	-2.1563500	H	-5.0188830	-0.1960110	2.4814980
C	-5.9367580	0.5129890	2.1026850	C	-4.1341620	-4.7801300	1.0449420
C	-3.1805850	-3.4481020	-0.1795070	C	-4.2563980	-0.7758260	-2.0560420
H	-6.4613540	1.4679930	2.0217310	H	-3.2545110	-4.6014870	1.6749640
H	-4.9956200	0.7593310	0.1774670	H	-3.0511910	-3.5265430	-0.3743460
C	-4.7166550	0.4339520	1.1864630	C	-4.0745380	-3.8620260	-0.1961110
H	-5.6176300	0.3938990	3.1443740	H	-4.1380470	-5.8411700	0.7813010
C	-4.2807720	-4.2146790	-0.9268510	C	-5.5739010	-0.8670530	-2.8380470
H	-5.2693600	-3.9342980	-0.5527170	H	-6.0965400	-1.7965830	-2.5842280
H	-4.2673490	-4.0423820	-2.0057330	H	-6.2545300	-0.0394890	-2.6260600
H	-3.2914930	-6.1064050	-1.3041090	H	-5.2122600	0.0982120	-4.7621790
C	-3.9734450	-5.6802350	-0.5624800	C	-5.1225420	-0.8978180	-4.3181670
H	-4.8787480	-6.2910400	-0.5688010	H	-5.7536140	-1.5663640	-4.9091410
C	-3.2922320	-5.6433210	0.8313970	C	-3.6387420	-1.3558700	-4.3093050
H	-3.8769500	-6.1681230	1.5899920	H	-3.4566800	-2.2005090	-4.9786920
C	-3.1370640	-4.1517900	1.1881500	C	-3.3296610	-1.7051860	-2.8458680
H	-2.2259070	-3.9360030	1.7476700	H	-2.2788840	-1.5783130	-2.5739280
H	-3.9823320	-3.8245700	1.8010780	H	-3.5983730	-2.7481990	-2.6456920
H	-3.8841410	1.0599370	1.5085520	H	-4.4196780	-4.3818090	-1.0944760
H	-2.3121680	-6.1274090	0.7911710	H	-2.9874800	-0.5394310	-4.6394930
H	-5.3114430	3.6342450	-3.6619390	H	-1.8093620	1.9314750	-5.2344230
H	-5.2032850	5.6791020	-2.4385040	H	-1.5120460	4.2378020	-4.5043780
C	-1.5743930	5.1065940	3.0018090	C	-4.0538170	4.5888030	2.5918630
C	-1.1363320	6.2057130	2.0157190	C	-4.9394190	5.0384090	1.4262690
H	-5.8117370	-2.6522020	1.7731600	H	-6.3423100	-2.3484650	1.8527810
H	-2.2284120	-3.6381970	-0.6918240	H	-3.8712020	0.2475340	-2.1591460
H	-3.4684430	0.1958500	-1.4473930	H	-5.0323410	1.1633100	-0.2261020
H	-3.9211300	-1.3178750	2.1545340	H	-5.9182410	-2.7372730	-0.4864850
H	-0.2022510	3.5084450	1.3107090	H	-2.5096790	5.2358400	-0.0214050
H	0.5541960	2.9478870	-1.1928540	H	0.2186670	2.4731920	-1.0268260
H	-3.9281620	2.8616060	-0.2722360	H	-3.6296660	2.3105820	-1.9005880
				H	0.8475770	0.6539380	0.0948980
**II(Pf$_2$)$_2$ (dication)**				**II(Pf$_2$)$_2$ (protonated)**			
C	0.8079320	-0.0429060	4.6368980	C	0.7110810	0.0481070	4.3117450
C	1.1073870	-1.5280060	4.3841520	C	1.5437250	-1.1942760	3.9365920
C	0.4438590	-2.4622510	5.3905710	C	1.3021220	-2.3949240	4.8411240
C	-1.0841360	-2.2505790	5.3779500	C	-0.1925330	-2.7639620	4.8275880
C	-1.4505190	-1.1501360	4.3880630	C	-0.9520910	-1.8064950	3.9171600
C	-0.7330120	0.1834040	4.6522210	C	-0.7989930	-0.3230090	4.3083380
C	-0.9861500	-1.4255160	2.9358670	C	-0.4507490	-1.7421400	2.4479770
C	0.5756190	-1.6666050	2.9397870	C	1.0888250	-1.3750210	2.4612950
C	0.8984090	0.6559040	3.2393960	C	0.5196870	0.8564550	2.9804530
C	1.3244720	-0.4805660	2.2873390	C	1.3915740	0.0691050	1.9953340

C	-0.6455220	0.8904260	3.2651090
C	-1.3797540	-0.0620470	2.3068040
P	-1.0564800	0.3118340	0.5436300
P	1.0527560	-0.3460790	0.4801530
N	1.9474890	0.7374850	-0.2657770
P	3.2067290	1.7141750	-0.2833590
C	3.3572440	2.6102160	1.3167340
C	2.1215400	3.4937480	1.5223260
N	-1.0708690	1.9100450	0.4215090
P	-1.8148040	3.0036010	-0.4803040
C	-0.7709370	4.5126550	-0.3817420
C	-1.0163430	5.5840420	-1.4485610
N	-1.9236060	-0.5188530	-0.4994050
P	-3.1780340	-1.4461960	-0.8247920
C	-3.3704500	-2.7571330	0.4527630
C	-2.1438080	-3.6766270	0.4248100
C	-3.5327950	3.3239920	0.1233780
C	-4.1525910	4.6516060	-0.3323620
C	-1.9452120	2.3973560	-2.2139100
C	-0.5480180	2.2236720	-2.8182350
C	-3.6407970	3.1755510	1.6460460
C	-2.9135740	3.1191450	-3.1535800
C	-0.7886480	5.1027520	1.0342770
C	-4.7077460	-0.4190630	-0.8982630
C	-5.9079800	-1.0264370	-1.6338790
C	-2.8231920	-2.1884790	-2.4708410
C	-3.6667020	-3.4013460	-2.8772890
C	-5.0877950	0.0604130	0.5083150
C	-2.8079240	-1.1075430	-3.5592340
C	-4.6831870	-3.5483940	0.4512260
N	1.0716730	-1.8461850	-0.0852390
P	1.8584780	-2.6505730	-1.2238750
C	2.0415840	-1.5888250	-2.7169850
C	3.0529360	-2.0188780	-3.7814870
C	0.8280710	-4.1289310	-1.5829550
C	0.8024150	-5.0854500	-0.3837280
C	3.5565740	-3.1254960	-0.6682740
C	4.2068950	-4.2745720	-1.4501290
C	1.1267100	-4.8662130	-2.8920520
C	3.6083300	-3.4051430	0.8389790
C	0.6640410	-1.2602590	-3.3022120
C	4.7432140	0.7440320	-0.5977270
C	5.9599990	1.5291380	-1.1017370
C	2.8966520	2.8865610	-1.6672300
C	3.7429910	4.1635440	-1.6929560
C	5.0871590	-0.1077830	0.6307280
C	2.9221600	2.1492250	-3.0123710
C	4.6611350	3.3742060	1.5730900
H	1.4999760	1.5600940	3.1809990
H	-2.4635830	0.0696680	2.4267420
H	2.4041990	-0.6490030	2.3977020
H	-2.5358820	-1.0016530	4.3900770
H	-1.5005140	-2.2789180	2.4966990
H	-1.1353170	0.7557740	5.4892810
H	1.3729050	0.4018180	5.4574660
H	2.1889860	-1.7019920	4.3787160
H	0.8211240	-2.6248520	2.4795250
H	-0.9616950	1.9301170	3.1905150
H	0.8583440	-2.2676250	6.3850230
H	0.6979050	-3.4977850	5.1426830
H	-1.4499930	-1.9733430	6.3717330
H	-1.6050740	-3.1722560	5.0985170
H	-4.6966670	3.1448050	1.9290070
H	-3.1608240	2.2646210	2.0072620
H	-3.1790790	4.0211400	2.1618770
H	-4.1012310	2.5054150	-0.3378940
H	-4.0817070	4.8173910	-1.4082220
H	-3.6876450	5.4998480	0.1763270
H	-5.2131540	4.6536050	-0.0664460
H	-2.3413530	1.3915300	-2.0331170
H	-3.9410940	3.0912560	-2.7837120
H	-2.6365760	4.1612740	-3.3289340
H	-2.9035430	2.6079260	-4.1208130
H	-0.0830220	3.1857140	-3.0556220
H	0.1149800	1.6751440	-2.1421980
H	-0.6218730	1.6585840	-3.7520450
H	0.2251460	4.0881910	-0.5518760
H	0.0339910	5.8158460	1.1371000
H	-1.7186090	5.6435330	1.2288840
H	-0.6671900	4.3320550	1.7993010
H	-0.8776860	5.2019610	-2.4617500
H	-2.0138590	6.0219460	-1.3777850
H	-3.3391660	-2.1718070	1.3811050
H	-5.5080830	-0.7480810	1.1120540
H	-4.2335570	0.4770480	1.0475520
H	-5.8452810	0.8456920	0.4345600
H	-6.2426810	-1.9652780	-1.1883630

C	-0.9979530	0.4804580	2.9769210
C	-1.3804030	-0.6048080	1.9645410
P	-1.6722230	-0.1772040	0.1525230
N	-1.0340150	-1.5747950	-0.5177130
P	-1.4221100	-2.6679500	-1.5687380
C	-1.8544670	-1.9666230	-3.2267240
C	-2.0535700	-2.9966200	-4.3405630
P	1.6666180	0.5064430	0.2511740
N	2.0480200	2.0758150	0.2475530
P	1.5100530	3.3395070	-0.5648040
C	0.3969420	2.8775730	-1.9635340
C	1.1142620	1.9986480	-2.9961350
N	-3.3642410	-0.3446290	0.1106600
P	-4.4302790	0.8156080	-0.0638410
C	-6.0850730	0.0750750	0.2838770
C	-6.0954990	-0.8046540	1.5378450
C	-4.5943270	1.5214620	-1.7734620
C	-5.1579140	0.4895440	-2.7567450
C	-4.0891710	2.2738700	1.0309230
C	-4.3576540	1.9455480	2.5043220
C	-3.2428710	2.0546830	-2.2610290
C	-4.7268110	3.6088930	0.6346890
C	-7.2359090	1.0858080	0.2794520
C	0.0657560	-3.7497740	-1.8370080
C	0.9895590	-3.7357860	-0.6161760
C	-2.8342100	-3.7894390	-1.1187430
C	-2.6985720	-4.1815550	0.3567040
C	-0.2482380	-5.1881670	-2.2660210
C	-4.2099330	-3.1719080	-1.3912910
C	-0.8360880	-0.8928470	-3.6145630
N	2.7720490	-0.4948670	-0.3099280
P	4.2731020	-0.9285220	-0.5233340
C	4.5531530	-1.2055260	-2.3238310
C	3.4380840	-2.1066570	-2.8695710
C	4.5360690	-2.5535000	0.3124760
C	4.6641950	-2.4014360	1.8324550
C	5.5146100	0.3308400	0.0021430
C	6.9594710	-0.1662320	-0.1233900
C	5.6546010	-3.4299180	-0.2618730
C	5.2386420	0.9450790	1.3790110
C	4.6151800	0.1233990	-3.0861000
C	0.5505800	4.4490780	0.5487910
C	-0.7925090	3.8066050	0.9102320
C	2.9334330	4.2973420	-1.2526430
C	2.6355350	5.7882120	-1.4605340
C	1.3695600	4.7780790	1.8024490
C	4.2089930	4.0940020	-0.4291490
C	-0.2758800	4.0706200	-2.6519910
H	0.7389770	1.9245500	3.0125610
H	-2.3975340	-0.9282300	2.2236320
H	2.4280390	0.2716990	2.3120530
H	-2.0105260	-2.0878450	3.9111860
H	-0.6111830	-2.6682190	1.8950370
H	-1.3763670	-0.0213940	5.1853920
H	1.0803610	0.5907460	5.1846300
H	2.6123740	-0.9503460	3.9537790
H	1.6704210	-2.1281070	1.9243080
H	-1.6714980	1.3397380	3.0099850
H	1.6407510	-2.1541270	5.8548020
H	1.9195370	-3.2319620	4.4968140
H	-0.6177980	-2.7188120	5.8364140
H	-0.3401650	-3.7897640	4.4725950
H	-2.5175670	1.2418840	-2.3626140
H	-3.3631240	2.5325630	-3.2381810
H	-2.8148680	2.7941970	-1.5773690
H	-5.2975620	2.3594690	-1.7083670
H	-6.2187840	0.2909840	-2.5847360
H	-4.6189040	-0.4602830	-2.6849160
H	-5.0533560	0.8581590	-3.7816300
H	-3.0020670	2.3660190	0.9102710
H	-4.4123800	3.9437150	-0.3567330
H	-5.8192870	3.5618180	0.6470380
H	-4.4217650	4.3783720	1.3513020
H	-5.4289690	1.9638870	2.7260530
H	-3.9650410	0.9639540	2.7824200
H	-3.8770400	2.6956040	3.1404450
H	-6.2156920	-0.5985830	-0.5717810
H	-7.1829610	1.7537730	1.1443720
H	-7.2542310	1.7005320	-0.6251230
H	-8.1901050	0.5536690	0.3361660
H	-7.0026720	-1.4170060	1.5421480
H	-5.2251950	-1.4639650	1.5514010
H	-2.8086110	-1.4599060	-3.0390800
H	-0.7042280	-5.7534300	-1.4468970
H	-0.9158620	-5.2428600	-3.1290680
H	0.6829800	-5.6958290	-2.5358470
H	1.2762190	-2.7164320	-0.3513630

H	-1.2091670	-3.1087450	0.3859930	H	-1.1058310	-3.4748830	-4.6084350
H	-2.1743990	-4.3522880	-0.4355000	H	-2.4361310	-2.5035000	-5.2396690
H	-2.1300410	-4.2980720	1.3248440	H	-2.7649720	-3.7804340	-4.0636220
H	-4.8571590	-4.0738380	-0.4895540	H	0.1428250	-1.3269100	-3.8483440
H	-5.5491410	-2.9172460	0.6572500	H	-0.6995010	-0.1769090	-2.8002030
H	-1.7892680	-2.5257300	-2.3313940	H	-2.7277460	-4.6874850	-1.7371860
H	-3.5279380	-4.2462670	-2.2005140	H	-2.7723740	-3.2917180	0.9892360
H	-4.7334500	-3.1752850	-2.9312500	H	-3.4977560	-4.8762180	0.6342220
H	-3.3481700	-3.7267520	-3.8714520	H	-1.7412570	-4.6668280	0.5712060
H	-3.8072630	-0.7114110	-3.7601520	H	-4.3898790	-3.0182100	-2.4595980
H	-2.4302670	-1.5413380	-4.4890230	H	-4.2981380	-2.2120400	-0.8722330
H	-2.1521170	-0.2765160	-3.2920730	H	-4.9899770	-3.8465940	-1.0222740
H	-6.7418490	-0.3210050	-1.5812270	H	0.5090490	-4.1908100	0.2558240
H	-5.6982210	-1.2029380	-2.6901540	H	1.8901600	-4.3190200	-0.8412960
H	-4.3705540	0.4508450	-1.4766310	H	0.6004270	-3.2598570	-2.6616920
H	-4.6331300	-4.3000890	1.2439110	H	-1.1779300	-0.3507250	-4.5023100
H	-0.2918890	6.3901670	-1.3020890	H	-6.0935200	-0.2082260	2.4534980
H	1.0225260	-4.2210750	-3.7664970	H	6.6375580	-2.9616620	-0.1697300
H	2.1259500	-5.3057820	-2.9043890	H	5.6852770	-4.3712570	0.2946560
H	0.4068600	-5.6823360	-3.0012160	H	5.4896190	-3.6774070	-1.3130840
H	-0.1663170	-3.6758190	-1.6694840	H	3.5775810	-3.0443450	0.1087050
H	0.6473740	-4.5555130	0.5591400	H	5.6559880	-2.0369660	2.1152570
H	1.7307040	-5.6584380	-0.3121750	H	4.5215920	-3.3757980	2.3081940
H	-0.0158030	-5.7997380	-0.5102740	H	3.9145470	-1.7197580	2.2416430
H	4.1330120	-2.2116100	-0.8631730	H	5.3628630	1.1254930	-0.7381600
H	4.6531290	-3.4556080	1.1577330	H	5.3472110	0.2188510	2.1871440
H	3.1081660	-2.6295070	1.4212190	H	5.9629860	1.7464620	1.5553500
H	3.1356490	-4.3598210	1.0828140	H	4.2415980	1.3890840	1.4269300
H	4.1781000	-4.1344280	-2.5315820	H	7.1955480	-0.9028050	0.6498110
H	5.2566330	-4.3507670	-1.1539560	H	7.1697160	-0.6123150	-1.0996520
H	3.7317970	-5.2305900	-1.2159680	H	7.6426310	0.6773870	0.0080440
H	2.4162460	-0.6700090	-2.2510660	H	5.5151680	-1.7182390	-2.4305850
H	-0.0287020	-0.9209090	-2.5260910	H	5.5368020	0.6738520	-2.8815440
H	0.7636600	-0.4593850	-4.0407940	H	4.5787370	-0.0702330	-4.1617570
H	0.2199500	-2.1205250	-3.8130450	H	3.7655490	0.7658860	-2.8339380
H	3.0677520	-1.2619580	-4.5713200	H	2.4657240	-1.6190710	-2.7497450
H	4.0664930	-2.0864900	-3.3792180	H	3.6055650	-2.2967260	-3.9331670
H	3.3116600	1.7841110	2.0382020	H	-0.3758780	2.2659340	-1.4748560
H	5.8439310	-0.8497840	0.3615290	H	2.2719790	5.3488860	1.5697340
H	5.4946640	0.4998770	1.4428240	H	0.7638500	5.3786640	2.4866220
H	4.2186970	-0.6480900	1.0155540	H	1.6716190	3.8636080	2.3197580
H	6.2763640	2.3070090	-0.4041140	H	-0.6587370	2.7943800	1.3040970
H	2.0838840	3.8372190	2.5600770	H	1.5372280	1.0909320	-2.5579110
H	1.1938100	2.9553010	1.3059500	H	1.9170950	2.5415350	-3.5043890
H	2.1622770	4.3841420	0.8875620	H	0.3965440	1.6910350	-3.7613280
H	4.5867990	3.8648590	2.5475970	H	-0.7782760	4.7437400	-1.9525600
H	4.8482540	4.1497790	0.8282230	H	-1.0311590	3.6982160	-3.3499610
H	1.8567780	3.1738320	-1.4729780	H	3.1025330	3.8410080	-2.2354930
H	4.8131030	3.9607420	-1.7682760	H	3.4637210	6.2438750	-2.0102030
H	3.4565600	4.7510030	-2.5696060	H	1.7220430	5.9675970	-2.0313550
H	3.5719200	4.7886830	-0.8147670	H	2.5475660	6.3134930	-0.5050750
H	2.5747060	2.8254890	-3.7979860	H	4.4255170	3.0343960	-0.2947070
H	2.2605400	1.2807890	-3.0071190	H	4.1286480	4.5474830	0.5627860
H	3.9286890	1.8173900	-3.2818980	H	5.0501290	4.5635500	-0.9471550
H	6.7962610	0.8349830	-1.2225570	H	-1.2977530	4.4054090	1.6738090
H	5.7790100	1.9923490	-2.0731970	H	-1.4578390	3.7446100	0.0449250
H	4.4267600	0.0702820	-1.4046070	H	0.3633040	5.3752820	-0.0053810
H	5.5298370	2.7147380	1.6079830	H	0.4456300	4.6519000	-3.2331470
H	2.7990140	-2.9745830	-4.2459260	H	3.3928710	-3.0726850	-2.3578910
				H	0.5299180	0.2807120	-0.5217760
**II(Pg$_2$)$_2$ (dication)**				**II(Pg$_2$)$_2$ (protonated)**			
C	-0.3683510	-1.7865400	2.4612720	C	0.7443070	1.2274740	2.7680390
C	0.9246810	-1.0073150	2.8661050	C	-0.8125740	1.2149050	2.9218050
C	0.9041520	0.4406830	2.3560000	C	-1.5228540	0.0447530	2.2302180
C	-0.3447200	0.9774350	3.0955470	C	-0.8065540	-1.1478140	2.8947570
C	-1.6528490	0.1849960	2.7043370	C	0.7641950	-1.1219210	2.7118620
C	-1.3621120	-0.9209710	1.6666780	C	1.3213010	0.0886850	1.9160560
C	0.4008090	-0.8986670	4.3323770	C	-0.6537380	0.6787350	4.3893750
C	0.0568540	0.5865600	4.5401180	C	-1.1537890	-0.7786750	4.3641460
C	-2.0784230	-0.7155440	3.8894990	C	1.4003520	-0.7603500	4.0837790
C	-0.8845200	-1.6829980	3.9339360	C	0.8932190	0.6892700	4.2313230
C	-2.3071330	0.0380720	5.1944250	C	1.0145950	-1.6715350	5.2421270
C	-1.0342830	0.8198270	5.5797730	C	-0.5138770	-1.6653100	5.4241680
P	1.0206570	0.6453720	0.5525710	P	-2.0138270	0.0398950	0.4899990
N	0.9150170	2.2185260	0.2893720	N	-2.6967910	-1.4088990	0.2928870
P	1.1976470	3.2246190	-0.9004640	P	-3.2881740	-2.0041990	-1.0518450
N	2.7684830	3.0699200	-1.4568420	N	-3.3129360	-3.6565730	-0.8537110
C	3.8317360	3.0346710	-0.4568990	C	-3.3176410	-4.2573700	0.4740310
P	-0.8442340	-0.4083610	-0.0039780	P	1.4374950	0.2956390	0.0384910
N	-0.4115690	-1.6650270	-0.8802900	N	3.0891980	0.7290110	0.0210010
P	-0.8783630	-3.0962340	-1.3477550	P	3.7486650	1.8369970	-0.8688580
N	0.3864840	-3.7044980	-2.2354000	N	5.4085800	1.7658780	-0.6090090
C	1.4589960	-2.8549680	-2.7416420	C	6.2380420	2.8166910	-1.1855170
N	-1.8736140	0.6153050	-0.6756130	N	1.3364670	-1.2715240	-0.5596450
P	-3.3878220	1.0612870	-0.5477700	P	2.1623200	-2.5983600	-0.4980120
N	-4.3243610	0.1660500	0.4911140	N	2.1912950	-3.2323810	-2.0538190

C	-4.6269500	-1.2262020	0.1873130
N	-3.4145020	2.6468870	-0.0672690
C	-4.5616710	3.5081140	-0.3218600
N	-4.1416750	0.9148800	-2.0194190
C	-3.4719780	1.3374940	-3.2419610
C	-2.4144880	3.2207780	0.8215520
N	2.2462580	-0.1439380	-0.0652830
P	3.6686950	-0.8041120	0.0428820
N	4.8093950	0.0747310	0.8674540
C	6.1033740	0.4626230	0.3303670
N	3.5978190	-2.2527180	0.8477680
C	2.5097370	-3.1820480	0.5865220
N	4.2096740	-1.0414590	-1.5018890
C	3.9996290	-0.0402430	-2.5377550
C	4.7294240	-2.8426510	1.5517970
N	0.3133660	3.0973880	-2.3020880
C	0.4188110	1.8407770	-3.0460090
N	0.8500070	4.7347610	-0.3062160
C	1.2593160	5.8974970	-1.0875670
C	-0.9991910	3.7343400	-2.4032320
C	0.6876860	5.0006650	1.1169940
C	3.2145440	3.6590160	-2.7179990
N	-1.1907110	-4.2729900	-0.2180460
C	-2.4076800	-4.2578130	0.5852440
N	-2.3328350	-2.9932910	-2.1690940
C	-3.0711120	-4.1844330	-2.5862810
C	-0.1743270	-5.1807060	0.2983870
C	-2.5106570	-1.8798950	-3.0988600
C	0.1939930	-4.9355290	-2.9946770
C	-5.5835620	0.7569760	-2.1711460
C	-5.0482170	0.7156500	1.6274500
C	5.1159170	-2.1204190	-1.8608490
C	4.6090340	0.4078380	2.2702410
H	-0.2026340	-2.7778160	2.0392220
H	1.7775990	0.9872940	2.7325370
H	-2.2818720	-1.4946290	1.5060670
H	0.9561830	1.1452020	4.8202630
H	-0.4837600	2.0533500	2.9694180
H	1.0133630	-1.3352220	5.1223730
H	-1.0748540	-2.6142000	4.4693380
H	-2.9904630	-1.2562810	3.6128590
H	-2.4440400	0.8698920	2.3975200
H	1.8621120	-1.5377860	2.6958850
H	-2.5819420	-0.6760700	5.9772180
H	-3.1604990	0.7132320	5.0723460
H	-0.6643460	0.5063780	6.5611950
H	-1.2399640	1.8927280	5.6506680
H	-5.6735620	-1.3518180	-0.1159150
H	-5.2411780	3.0506850	-1.0400110
H	-3.9896700	-1.5918730	-0.6225490
H	-5.1214600	3.7182520	0.5976940
H	-4.2142340	4.4583160	-0.7409760
H	-6.1298440	0.7221110	1.4434970
H	-4.8522100	0.1106360	2.5198670
H	-4.7248170	1.7357160	1.8325240
H	-1.5017020	2.6259560	0.7977970
H	-2.1608640	4.2265620	0.4692330
H	-2.7799150	3.2982380	1.8546030
H	-2.3921640	1.2689810	-3.1225400
H	-3.7373730	2.3680950	-3.5105670
H	-3.7691720	0.6772320	-4.0627910
H	-5.8008940	-0.1271430	-2.7810920
H	-6.0190150	1.6325640	-2.6679580
H	-2.1376450	-2.1288090	-4.1012440
H	-1.9948580	-0.9954890	-2.7293730
H	-3.5772790	-1.6453710	-3.1717220
H	-2.8793780	-5.0185690	-1.9122040
H	-2.8007030	-4.4878850	-3.6056950
H	-4.1425780	-3.9618100	-2.5648560
H	1.1342810	-5.4949180	-3.0192730
H	-0.5590190	-5.5687750	-2.5221690
H	-0.1163810	-4.7294940	-4.0270560
H	1.5641110	-1.9693060	-2.1163850
H	0.7254790	-5.1335960	-0.3118450
H	1.2682980	-2.5459630	-3.7777850
H	0.0858690	-4.9315600	1.3348100
H	2.3987430	-3.4176970	-2.7157410
H	-3.1471090	-3.5898960	0.1475840
H	-2.1992630	-3.9342680	1.6132200
H	-2.8364380	-5.2647320	0.6249200
H	-0.5584520	-6.2063240	0.2806200
H	-6.0696980	0.6396890	-1.2030900
H	-4.4581110	-1.8467150	1.0749200
H	6.9130960	-0.1149620	0.7945680
H	2.8056560	-3.9536520	-0.1370860
H	6.1373940	0.3055960	-0.7471350
H	2.2251120	-3.6772140	1.5213210
C	2.1861030	-2.3855830	-3.2321460
N	3.7585250	-2.6297950	0.0423340
C	4.0265070	-2.2707240	1.4321430
N	1.4683940	-3.7481910	0.5050300
C	2.1443500	-4.9134780	1.0515090
C	4.7965500	-2.1441370	-0.8609280
N	-3.0307370	1.2648540	0.2532160
P	-2.8919330	2.7814020	-0.1512250
N	-3.9425490	3.0458070	-1.4219450
C	-5.0826820	2.1662040	-1.6418980
N	-3.1681970	3.7222850	1.2018410
C	-3.9693370	3.2108520	2.3071610
N	-1.4610630	3.3897310	-0.7334520
C	-1.0086610	3.0031290	-2.0673390
C	-3.1587040	5.1791130	1.1237060
N	-4.7887100	-1.3357280	-1.3817040
C	-5.4882370	-1.5893470	-2.6378310
N	-2.4634740	-1.7739350	-2.4800250
C	-1.2308670	-2.5329410	-2.7096880
C	-5.6981680	-1.1400190	-0.2545720
C	-2.4005270	-0.4278530	-3.0515260
C	-3.9467800	-4.4798760	-1.8770700
N	3.6555640	1.7757820	-2.5526630
C	4.4478350	0.7648910	-3.2466800
N	3.1453550	3.3875950	-0.5656140
C	3.0534390	3.7627140	0.8402080
C	2.3309450	1.9352250	-3.1454330
C	3.4049000	4.5369450	-1.4245430
C	5.9329340	1.2518660	0.6476030
C	0.0191290	-3.8447950	0.5457800
C	2.6729670	-4.5800850	-2.3019310
C	-0.3527090	3.7327360	0.1555560
C	-4.1171350	4.3861570	-1.9682610
H	1.1591220	2.2156040	2.5606010
H	-2.5496670	0.0402190	2.6309390
H	2.3963130	0.0884600	2.1255710
H	-2.2421260	-0.8102630	4.4857710
H	-1.2242940	-2.1096370	2.5913530
H	-1.0679880	1.2849270	5.1977770
H	1.4522090	1.2945220	4.9484830
H	2.4901800	-0.7707700	3.9754070
H	1.1116000	-2.0802870	2.3235860
H	-1.3058520	2.1799690	2.7887590
H	1.5213990	-1.3340990	6.1529250
H	1.3816100	-2.6835120	5.0373780
H	-0.7922210	-1.2940700	6.4166400
H	-0.9251600	-2.6771900	5.3431700
H	3.1855850	-2.3071090	-3.6861530
H	4.9817880	-2.7110350	1.7375480
H	1.5087140	-2.8015870	-3.9875350
H	4.0759150	-1.1825690	1.5590160
H	3.2464510	-2.6623280	2.0869240
H	2.1291440	-5.0087720	-3.1507450
H	3.7468240	-4.6037120	-2.5397170
H	2.4982040	-5.2206090	-1.4383680
H	4.8588320	-1.0488480	-0.8568760
H	5.7612010	-2.5551280	-0.5444750
H	4.6061990	-2.4847640	-1.8800060
H	1.7641560	-5.8421920	0.6032490
H	1.9723840	-4.9696430	2.1343100
H	3.2171390	-4.8510710	0.8711710
H	-0.4354120	-2.8899990	0.2799140
H	-0.3000880	-4.1038660	1.5624650
H	3.5910690	4.2181330	-2.4491810
H	4.2725350	5.1163100	-1.0787080
H	2.5308260	5.1988830	-1.4165690
H	2.2346940	4.4761410	0.9834630
H	3.9812700	4.2351170	1.1962270
H	2.8554820	2.8800360	1.4479350
H	5.2608760	0.4891500	1.0352220
H	6.9191940	0.8072650	0.4732290
H	6.0436690	2.0458650	1.4011090
H	5.8388760	3.1314690	-2.1511450
H	1.7429340	1.0063840	-3.1024760
H	7.2501510	2.4304710	-1.3448970
H	1.7748630	2.7136960	-2.6243030
H	6.3076570	3.6962830	-0.5287440
H	3.9796240	-0.2270300	-3.2035760
H	5.4429610	0.6967190	-2.8087480
H	4.5431420	1.0533350	-4.2982520
H	2.4397390	2.2282580	-4.1944990
H	-0.3571260	-4.6207130	-0.1372180
H	1.8370020	-1.3899490	-2.9584680
H	-4.8385220	1.1598770	-1.3034750
H	-3.5711140	3.6013690	3.2498720
H	-5.9818400	2.5188730	-1.1178330
H	-5.0199770	3.5193170	2.2217960

H	1.6430750	-2.6457940	0.1979530	H	-3.9208800	2.1232510	2.3299290
H	5.4728000	0.0850270	2.8625100	H	-3.1967410	4.9624020	-1.8655570
H	4.4799000	1.4887770	2.4064150	H	-4.3499500	4.3092200	-3.0352210
H	3.7314600	-0.1053710	2.6664260	H	-4.9348500	4.9289800	-1.4758200
H	4.4261090	-3.1233790	2.5663210	H	-2.7687790	5.5857350	2.0626980
H	5.0870020	-3.7436840	1.0380100	H	-2.5106630	5.5173500	0.3146920
H	5.5545460	-2.1342440	1.6182630	H	-4.1660690	5.5851680	0.9641540
H	3.3517930	0.7534120	-2.1618810	H	-0.4566420	3.8368000	-2.5149830
H	3.5245690	-0.5006150	-3.4119630	H	-1.8611830	2.7679810	-2.7044490
H	4.9526610	0.4024410	-2.8567710	H	-0.3433400	2.1323760	-2.0099070
H	5.1057030	-2.9012870	-1.1005270	H	-0.7157600	3.9262030	1.1645440
H	6.1474870	-1.7665730	-1.9856280	H	0.1392730	4.6370570	-0.2214200
H	-0.0922350	5.7570050	1.2547390	H	-2.4089200	-0.4945210	-4.1440390
H	1.6184840	5.3761260	1.5618340	H	-1.4784970	0.0850580	-2.7462150
H	0.3938830	4.0885160	1.6333550	H	-3.2532950	0.1715390	-2.7310110
H	0.5357520	6.7051270	-0.9400830	H	-0.3756880	-2.0740390	-2.1978170
H	1.2817030	5.6588140	-2.1526140	H	-1.0373570	-2.5587880	-3.7865510
H	2.2511140	6.2592270	-0.7866610	H	-1.3437480	-3.5557850	-2.3515070
H	3.9880900	3.0175400	-3.1534940	H	-3.8465430	-4.0135430	-2.8586050
H	2.3858220	3.7331410	-3.4206020	H	-3.4478000	-5.4533920	-1.9131570
H	3.6425610	4.6585520	-2.5672020	H	-5.0124900	-4.6445210	-1.6687270
H	4.1552160	4.0451950	-0.1728200	H	-2.7832550	-5.2124740	0.4358490
H	-1.7739200	3.1707890	-1.8750590	H	-6.4205620	-0.3595470	-0.5130020
H	4.6907860	2.5040160	-0.8766900	H	-4.3398550	-4.4466070	0.8287450
H	-0.9665890	4.7440400	-1.9966530	H	-6.2548580	-2.0567490	-0.0156250
H	3.5029360	2.4992800	0.4328180	H	-2.8129080	-3.5969150	1.1773450
H	0.1750650	2.0249610	-4.0962510	H	-6.1682820	-2.4477860	-2.5601960
H	-0.2645810	1.0804060	-2.6484210	H	-4.7769370	-1.7771450	-3.4417290
H	1.4365480	1.4523220	-2.9964850	H	-6.0793230	-0.7048420	-2.8991460
H	-1.2671460	3.7965630	-3.4618000	H	-5.1405000	-0.8148580	0.6228650
H	4.7885280	-2.5648700	-2.8067500	H	0.3831660	2.9207380	0.1835540
H	6.2847460	1.5249980	0.5273890	H	-5.3035700	2.1337090	-2.7145540
				H	-0.8949870	0.1928070	-0.3462120
**II(Ph$_2$)$_2$ (dication)**				**II(Ph$_2$)$_2$ (protonated)**			
C	3.0617010	-4.0353780	-2.5921980	C	2.3476400	-4.2496100	1.5600980
N	2.3529090	-2.9496440	-1.8941020	N	1.5525360	-3.7988290	0.4090470
C	2.2869220	-1.8540160	-2.8725120	C	0.1181590	-3.8048810	0.7542590
C	1.9749600	-2.5445510	-4.2051890	C	0.0434930	-4.5088610	2.1221390
C	2.6371140	-3.9355100	-4.0760390	C	1.3975120	-5.2100300	2.2654690
P	1.0505840	-3.2340810	-0.8873040	P	2.1712610	-2.5940620	-0.5789540
N	-0.2969200	-3.8913510	-1.5886630	N	1.0222780	-2.6172870	-1.8230040
C	-1.3354650	-3.0055200	-2.1568010	C	0.7284080	-3.8873100	-2.5164130
C	-2.1871650	-3.9492620	-3.0043230	C	1.3131950	-3.7565620	-3.9481960
C	-1.1801900	-4.9975500	-3.4832550	C	1.9227890	-2.3438400	-3.9976350
C	-0.3037380	-5.2100800	-2.2488840	C	1.1737340	-1.6107820	-2.8837280
N	0.5470520	-1.8927850	-0.2403810	N	2.4590850	-1.1825790	0.0088410
P	0.9177340	-0.5036960	0.4304540	P	1.5488670	0.2430690	0.1356360
C	1.3700180	-0.7404410	2.1786840	N	2.6419320	1.5016520	-0.0546880
C	0.3614650	-1.4749530	3.0766290	P	4.1751790	1.7734630	-0.0235410
C	-0.9498780	-0.6531430	3.2967850	N	5.0433160	1.3225830	1.3407090
C	-0.9299730	0.6879530	2.5511270	C	5.0400270	-0.0763470	1.7837460
C	0.2857260	1.3605850	3.2302310	C	6.4864480	-0.5295090	1.5876030
C	1.6147980	0.5327610	3.0222220	C	7.3014100	0.7219460	1.9554520
C	-0.4826570	-0.2904650	4.7414000	C	6.3362610	1.9140820	1.7255380
C	-0.1618190	1.2140780	4.7056440	C	1.4441430	0.2951940	2.0102130
C	2.0077680	-0.1463330	4.3561780	C	0.8077190	-0.7886650	2.9159530
C	0.8233580	-1.1122710	4.5266440	C	-0.7696580	-0.7173230	3.0488980
C	2.1835560	0.8228970	5.5194980	C	-1.3929730	0.4258800	2.2160400
P	-0.9799980	0.5856470	0.7339030	C	-0.6326350	1.6262040	2.7902250
N	-0.9238090	2.0814290	0.1945300	C	0.9218450	1.5487850	2.7051300
P	-1.0756830	3.0008320	-1.0845170	C	-0.5621150	1.2627630	4.3174960
N	-2.6154020	3.5389360	-1.3814270	C	-1.1421510	-0.1595330	4.4486020
C	-3.1724510	4.8517050	-1.0376970	C	0.9886810	1.1752520	4.2249640
C	-4.5189740	4.8343090	-1.7576230	C	1.4189200	-0.3064570	4.2624950
C	-4.9715600	3.3838010	-1.5684290	C	-0.5841230	-0.9453310	5.6275930
C	-3.6756750	2.5779630	-1.7263640	C	0.9457560	-1.0599890	5.4991320
N	1.9559020	0.4454610	-0.3152880	P	-1.7885020	0.1113530	0.4700990
P	3.4840640	0.8494900	-0.3103260	N	-3.2128190	-0.6405210	0.5813280
N	3.9119790	1.0382430	-1.9069560	P	-4.0614390	-1.6583660	-0.2738540
C	3.0189050	1.7759760	-2.8129330	N	-3.8318290	-3.1686460	0.3550380
C	3.7443110	3.1027240	-3.0434200	C	-4.3203410	-4.3902750	-0.2958330
C	5.2326720	2.7106450	-3.0520310	C	-3.7620290	-5.5005100	0.5946650
C	5.2962400	1.3553880	-2.3030970	C	-3.8207970	-4.8711720	1.9889200
N	4.6453190	-0.1552630	0.2894770	C	-3.3968420	-3.4223580	1.7397950
C	5.0690920	-0.1843070	1.7036520	N	-1.7283030	1.4261840	-0.4390800
C	5.3956830	-1.6528900	1.9625410	P	-2.5961190	2.7358450	-0.5867470
C	5.9063240	-2.1367140	0.6035110	N	-1.5716650	3.9071200	-1.1295900
C	5.0138500	-1.4081010	-0.4054720	C	-2.0251270	5.0712660	-1.9024010
N	3.6445680	2.2075300	0.6352010	C	-0.7559350	5.9186300	-2.0575320
C	4.8790420	2.9900420	0.7325270	C	0.3761630	4.8919110	-1.9607660
C	4.5828640	3.9269180	1.9106740	C	-0.1243140	3.9377910	-0.8785010
C	3.0449850	4.1099890	1.8827080	N	-3.8378640	2.5040430	-1.6574510
C	2.5515980	3.1717220	0.7684030	C	-5.0100800	3.3877190	-1.7837950
N	1.6290260	-4.4385450	0.0898160	C	-5.2798650	3.4211670	-3.2873570
C	0.7071450	-5.1461150	1.0098290	C	-4.8638780	2.0176520	-3.7343770
C	1.5162950	-5.3318790	2.2966900	C	-3.6160370	1.7305440	-2.8947300

C	2.9627770	-5.3600550	1.8029660
C	2.9614020	-4.2990110	0.7080320
N	-2.1531610	-0.3655760	0.2162970
P	-3.6307060	-0.7454730	0.6364590
N	-4.2735740	0.0906950	1.9055510
C	-4.4015780	1.5544750	1.9161820
C	-4.5625640	1.9211300	3.4104820
C	-4.3311900	0.6114170	4.1828040
C	-4.7195770	-0.4746730	3.1811190
N	-3.6691490	-2.3497500	1.0006800
C	-4.8723690	-3.1759010	0.9575290
C	-4.3711370	-4.4776100	0.3241340
C	-2.8946070	-4.5831460	0.7634920
C	-2.5236870	-3.1945600	1.3360680
N	-4.6504300	-0.4565750	-0.6315600
C	-6.0738230	-0.0890150	-0.5293840
C	-6.6784560	-0.6477760	-1.8178740
C	-5.5255830	-0.5195840	-2.8167640
C	-4.3097670	-0.9242970	-1.9845830
N	-0.6099650	2.2512750	-2.4903460
C	-0.8669970	2.8560720	-3.8120770
C	-0.3695390	1.7904540	-4.7842360
C	-0.7597610	0.4893180	-4.0834670
C	-0.4874570	0.7794480	-2.6056480
N	-0.1657660	4.3507060	-0.8186210
C	0.5038950	5.1551750	-1.8476290
C	1.2299750	6.2284890	-1.0350630
C	0.2964050	6.4525540	0.1570160
C	-0.2241300	5.0497250	0.4792650
H	2.4386190	0.2583250	6.4219650
H	3.0332990	1.4812180	5.3104630
H	-1.8777310	-1.2145840	3.1926580
H	2.3035910	-1.3187240	2.1363160
H	-1.8229470	1.2670080	2.8102580
H	2.9353490	-0.7109040	4.2052940
H	2.4070460	1.1697200	2.6244430
H	1.0033640	-1.9336040	5.2216760
H	-1.1209910	-0.5974740	5.5714830
H	-1.0760840	1.7987810	4.8573320
H	0.4161990	2.4027210	2.9295510
H	0.2181930	-2.5261890	2.8238360
H	1.2235160	-6.2333840	2.8377470
H	1.3692810	-4.4743200	2.9620700
H	3.6930410	-5.1392220	2.5840080
H	3.2018360	-6.3382660	1.3739370
H	3.0842900	-3.2996880	1.1460060
H	3.7541250	-4.4458670	-0.0283020
H	0.4215440	-6.1092040	0.5738000
H	-0.2046240	-4.5710410	1.1800180
H	-2.6892400	-3.4245000	-3.8196690
H	-0.8887650	-2.2247840	-2.7874730
H	-1.8870660	-2.5027890	-1.3608580
H	1.9358990	-4.7312550	-4.3410650
H	0.7035670	-5.5479600	-2.5004540
H	3.5053090	-4.0371260	-4.7305280
H	-0.5778910	-4.5930720	-4.3044050
H	4.1376100	-3.8488550	-2.4906470
H	-0.7454530	-5.9596160	-1.5801540
H	2.8482810	-5.0027680	-2.1362270
H	2.3469600	-1.9734950	-5.0585540
H	1.5559720	-1.1069430	-2.5716710
H	0.8910830	-2.6484500	-4.3237190
H	3.2633890	-1.3563440	-2.9001270
H	5.8542620	-3.2209610	0.4844920
H	6.9491320	-1.8326270	0.4700140
H	4.1180860	-1.9919500	-0.6449290
H	2.0252820	1.8983490	-2.3804280
H	2.9184990	1.2128080	-3.7502800
H	5.5970890	2.5905060	-4.0750290
H	3.4278610	3.6000940	-3.9631770
H	3.5328890	3.7794330	-2.2070860
H	5.8553700	3.4676970	-2.5698310
H	5.6634840	0.5656830	-2.9674880
H	5.9512100	1.3787990	-1.4299700
H	4.4904350	-2.1972220	2.2518350
H	4.2940570	0.2051800	2.3651480
H	6.1269020	-1.7738700	2.7638900
H	5.7486480	2.3515630	0.8938950
H	5.0443320	3.5692080	-0.1892080
H	5.1232900	4.8707690	1.8177150
H	4.8932120	3.4518070	2.8445430
H	2.6036490	3.8280760	2.8431090
H	2.4086190	3.7256920	-0.1699350
H	1.6095840	2.6674270	0.9823470
H	5.9642030	0.4336090	1.8415920
H	2.7561420	5.1441430	1.6796060
H	-2.9542990	-4.4263260	-2.3851040
N	-3.3619080	3.3592210	0.7421910
C	-4.3960380	2.6213030	1.4822290
C	-4.1778910	3.0639110	2.9280950
C	-3.7340420	4.5212890	2.7677300
C	-2.8485190	4.5035680	1.5143450
N	3.6437480	-3.2511650	-1.0021910
C	4.8317160	-2.4735090	-1.3253330
C	5.9636520	-3.4153930	-0.9072720
C	5.4025830	-4.8290450	-1.1904710
C	3.8902580	-4.6189380	-1.4463430
N	-5.6947330	-1.3449150	-0.2536000
C	-6.1354430	-0.0274140	-0.7701160
C	-7.2290180	0.4442090	0.2048820
C	-6.9503530	-0.3469340	1.4854410
C	-6.4961610	-1.6960320	0.9335550
N	-3.7274180	-1.6660890	-1.8957020
C	-2.3292160	-1.7619030	-2.3515840
C	-2.4494790	-2.1016190	-3.8411820
C	-3.7736430	-2.8611360	-3.9310620
C	-4.6633970	-2.1016520	-2.9495490
N	4.5262730	3.4138630	-0.0815420
C	4.2011230	4.1086960	-1.3403030
C	4.1156650	5.5757490	-0.9260230
C	3.4766850	5.4863280	0.4612350
C	4.1596410	4.2603370	1.0741120
N	4.9219080	1.1307900	-1.3723430
C	6.3514830	1.2788030	-1.6485100
C	6.5523040	0.4334630	-2.9042110
C	5.2506010	0.6704860	-3.6762210
C	4.1791740	0.7438480	-2.5805000
H	1.4125140	2.4695150	2.3852970
H	-2.4264130	0.5321840	2.5728380
H	2.5146340	0.2544090	2.2357910
H	-2.2340550	-0.1220720	4.5298020
H	-1.2292540	-1.6907500	2.8555040
H	-0.9709230	1.9837700	5.0289350
H	1.5544610	1.8270140	4.8943640
H	2.5092020	-0.3841390	4.1957660
H	1.1277430	-1.7984710	2.6485430
H	-1.0692760	2.5891870	2.5263030
H	1.4468110	-0.6545980	6.3848830
H	1.2549140	-2.1081650	5.4178580
H	-0.8661000	-0.4413690	6.5585220
H	-1.0529050	-1.9349800	5.6541520
H	0.5185780	-3.8614470	-4.6925280
H	2.0540410	-4.5320150	-4.1569000
H	2.9920040	-2.3758050	-3.7588390
H	1.8092290	-1.8647490	-4.9728930
H	1.6760160	-0.7204180	-2.5085260
H	0.1802020	-1.3052310	-3.2305520
H	-0.3577910	-4.0299020	-2.5580780
H	1.1411870	-4.7304010	-1.9602220
H	6.1600070	-3.2903480	0.1615920
H	4.8214770	-1.5137450	-0.8079070
H	4.8870870	-2.2758250	-2.4094600
H	-0.7995410	-5.2008860	2.1819500
H	3.2568650	-5.3241420	-0.9037070
H	-0.0777090	-3.7695820	2.9214840
H	5.5678300	-5.4951980	-0.3404080
H	-0.2928180	-2.7885890	0.8072920
H	3.6722080	-4.7281910	-2.5190860
H	-0.4500700	-4.3419770	-0.0132080
H	1.6780050	-5.3883510	3.3056500
H	3.2732810	-4.7257280	1.2293300
H	1.3888000	-6.1704700	1.7402430
H	2.6188290	-3.4129420	2.2218280
H	2.4008980	5.3011940	0.3653460
H	3.6138230	6.3868810	1.0636920
H	3.5036310	3.7271830	1.7690630
H	4.3089010	-0.6673270	1.2248360
H	4.7712080	-0.1211050	2.8498130
H	7.6072100	0.6840790	3.0045970
H	6.7435560	-1.3984430	2.1988760
H	6.6415210	-0.8006480	0.5378450
H	8.2101930	0.8145460	1.3550830
H	6.2174660	2.4939880	2.6487150
H	6.6859190	2.6010420	0.9526460
H	3.5403040	6.1753220	-1.6357840
H	3.2332230	3.7753670	-1.7443190
H	5.1200690	6.0062390	-0.8535640
H	6.6232270	2.3291610	-1.8368250
H	6.9537470	0.9266470	-0.8075680
H	6.6472890	-0.6217490	-2.6270020
H	7.4447940	0.7214770	-3.4640460
H	5.3078860	1.6248660	-4.2102620
H	3.7018580	-0.2271850	-2.4210940
H	3.3911220	1.4639390	-2.8222200

H	-1.6457030	-5.9233990	-3.8261900
H	5.5408900	-1.1951900	-1.3391060
H	1.4050570	7.1331610	-1.6198920
H	2.2008190	5.8530980	-0.6950980
H	0.7939420	6.9111230	1.0140310
H	-0.5357600	7.0997370	-0.1386350
H	0.4060950	4.5391120	1.2135330
H	-1.2389070	5.0754560	0.8860780
H	-0.2179560	5.6187640	-2.5343450
H	1.1915200	4.5375140	-2.4329420
H	0.7187960	1.8573040	-4.8842680
H	-0.3318630	3.7994450	-3.9276930
H	-1.9367090	3.0561670	-3.9557210
H	-5.3697780	3.2564560	-0.5563380
H	0.5191980	0.4776950	-2.3126130
H	-5.7420530	3.0706400	-2.2762050
H	-0.1952250	-0.3765980	-4.4381930
H	-3.5475720	2.2266250	-2.7593170
H	-1.1993030	0.2551250	-1.9621710
H	-3.6564720	1.6999740	-1.0737820
H	-4.3741920	5.0539650	-2.8203510
H	-3.3257940	4.9597990	0.0455610
H	-5.2206300	5.5614560	-1.3445340
H	-2.5128240	5.6548600	-1.3751060
H	-3.8641290	2.7053380	3.7105680
H	-5.5733080	2.2946530	3.5914290
H	-3.5212290	2.0214580	1.4594180
H	-4.1747590	-2.0145570	-1.9947320
H	-3.3791900	-0.4643050	-2.3304010
H	-7.5764630	-0.1052630	-2.1191200
H	-5.4253770	0.5211340	-3.1442730
H	-5.6451070	-1.1460080	-3.7024830
H	-6.9427890	-1.7024690	-1.6841280
H	-6.1826550	1.0019890	-0.5004270
H	-6.5359400	-0.4959730	0.3734550
H	-3.2765120	0.4991330	4.4517600
H	-4.2346050	-1.4338600	3.3725560
H	-4.9190940	0.5575860	5.1010560
H	-5.2586050	-3.3487860	1.9712540
H	-5.6574770	-2.6936830	0.3715180
H	-4.4424410	-4.3948830	-0.7640610
H	-4.9665140	-5.3394050	0.6320680
H	-2.7568590	-5.3565320	1.5226200
H	-1.6137300	-2.7724330	0.9020400
H	-2.3931300	-3.2559080	2.4251600
H	-5.8061370	-0.6356580	3.1763590
H	-2.2485200	-4.8333740	-0.0807110
H	-0.8130790	1.8991790	-5.7756000
H	-1.8246530	0.2851260	-4.2394540
H	-5.2775750	1.8784580	1.3430260
C	0.8905350	1.6392580	5.7239260
H	0.4908990	1.4946000	6.7326910
H	1.0832400	2.7114230	5.6139060

H	4.9683920	3.9216310	-2.0976700
H	5.0349260	-0.1120020	-4.4078030
H	6.8924910	-3.2119430	-1.4461150
H	5.8769120	-5.2865650	-2.0619230
H	5.0652460	4.5465240	1.6227710
H	-0.6813290	6.6288260	-1.2275680
H	-0.7525560	6.4855310	-2.9904480
H	0.4873380	4.3533600	-2.9078150
H	1.3374960	5.3440990	-1.7090930
H	0.3035770	2.9354870	-0.9363670
H	0.0940960	4.3408580	0.1223320
H	-2.8196190	5.6243290	-1.3875740
H	-2.4160760	4.7516570	-2.8766060
H	-5.6537270	1.3002220	-3.4878410
H	-3.5058080	0.6687470	-2.6632810
H	-2.7086270	2.0731560	-3.4090660
H	-4.6079490	5.1578320	2.5968550
H	-5.8656360	2.9546910	-1.2484760
H	-3.1993140	4.9074260	3.6376760
H	-6.3208610	3.6578740	-3.5166270
H	-1.7933990	4.3669240	1.7781920
H	-4.8198670	4.3753100	-1.3587320
H	-2.9275630	5.4344940	0.9447400
H	-3.3779350	2.4715240	3.3858640
H	-5.3971250	2.9118960	1.1347340
H	-5.0766300	2.9527660	3.5381690
H	-4.2901770	1.5409170	1.3405310
H	-7.2027740	1.5261360	0.3538430
H	-8.2187990	0.1856750	-0.1837930
H	-5.2921060	0.6722650	-0.7898430
H	-1.7881860	-2.5422540	-1.8001850
H	-1.8012320	-0.8147980	-2.1979680
H	-4.1911830	-2.8806080	-4.9396320
H	-2.5047590	-1.1787710	-4.4283750
H	-1.5954140	-2.6783830	-4.2018710
H	-3.6388130	-3.8956270	-3.5965280
H	-5.1119440	-1.2248130	-3.4346930
H	-5.4718570	-2.7072680	-2.5364130
H	-6.1326560	0.1114350	2.0521680
H	-5.9180480	-2.2870990	1.6454550
H	-7.8225070	-0.4279830	2.1377890
H	-5.4187580	-4.4200280	-0.3185290
H	-3.9547870	-4.4517130	-1.3251470
H	-2.7232340	-5.7112510	0.3173540
H	-4.3338400	-6.4265590	0.5121360
H	-4.8481300	-4.9003850	2.3669940
H	-2.3121010	-3.3072480	1.8187310
H	-3.8510590	-2.7228560	2.4470560
H	-7.3588270	-2.2989520	0.6248100
H	-3.1779120	-5.3684000	2.7186010
H	-4.6634710	1.9433770	-4.8049550
H	-4.6436720	4.1741600	-3.7649440
H	-6.5179840	-0.1264600	-1.7908670
H	-0.8300900	-0.7608810	-0.0554600

III(Pa$_2$)$_2$ (dication)

C	2.2814320	-0.7016440	1.6293910
C	1.8161220	0.7862920	1.6887790
C	3.0828070	1.6588960	1.5649380
C	4.3406250	0.7797440	1.6747250
C	3.8546550	-0.7042020	1.6334580
C	3.0793310	2.2772540	0.1297890
C	1.8108690	1.7637660	-0.5858930
C	0.9224120	1.4491090	0.6286500
C	4.3378280	1.7565430	-0.5864550
C	3.8546550	0.7042020	-1.6334590
C	2.2814310	0.7016440	-1.6293920
C	5.2484260	1.3787890	0.5905670
C	4.3406250	-0.7797440	-1.6747260
C	3.0828060	-1.6588960	-1.5649390
C	1.8161220	-0.7862920	-1.6887790
C	3.0793310	-2.2772540	-0.1297900
C	4.3378280	-1.7565430	0.5864540
C	5.2484250	-1.3787890	-0.5905680
C	1.8108680	-1.7637660	0.5858930
C	0.9224120	-1.4491090	-0.6286510
H	1.3430370	0.9412910	2.6622320
H	1.3324330	2.5719860	-1.1454480
H	4.8223110	0.9293610	2.6451010
H	4.8169820	2.5655470	-1.1450110
H	0.7479430	2.4471180	1.0650020
H	6.1361600	0.8056530	0.3409380
H	5.6197790	2.3270960	1.0004000
H	6.1361600	-0.8056540	-0.3409400
H	5.6197780	-2.3270960	-1.0004010
H	0.7479430	-2.4471180	-1.0650030
H	4.8223100	-0.9293620	-2.6451020

III(Pa$_2$)$_2$ (protonated)

C	0.8452740	-2.0083690	-1.6623280
C	-0.4919300	-2.3804830	-0.9513050
C	-0.3312280	-3.8066760	-0.3990100
C	0.9270540	-4.4659190	-0.9815820
C	1.7014390	-3.3327540	-1.7329350
C	-0.1066100	-3.6624520	1.1422430
C	-0.1102930	-2.1448530	1.4555220
C	-0.9969320	-1.6511270	0.2894770
C	1.2641890	-4.2846230	1.4528250
C	2.2287710	-3.1030630	1.7645890
C	1.3853930	-1.7680450	1.7747900
C	1.4907950	-5.2036030	0.2442260
C	3.5618520	-2.8476530	0.9872010
C	3.4811660	-1.4062780	0.4698220
C	2.2767250	-0.6806890	1.0947150
C	3.2098770	-1.4811110	-1.0700960
C	3.1871860	-2.9696810	-1.4459300
C	3.9591060	-3.6138300	-0.2856020
C	1.8253850	-0.8248080	-1.3252020
C	1.8276510	0.1463800	-0.1154430
H	-1.2786800	-2.3489540	-1.7090020
H	-0.6702710	-1.9512460	2.3743860
H	0.6547130	-5.2148650	-1.7315150
H	1.2085250	-4.9052150	2.3524490
H	-1.9372470	-2.1766720	0.5176450
H	2.4892040	-5.6193810	0.1450690
H	0.8306160	-6.0683490	0.3948570
H	3.9154250	-4.6974320	-0.2331990
H	5.0172270	-3.3734800	-0.4552960
H	2.7588680	0.6891410	-0.3501120
H	4.3768510	-2.9472160	1.7103300

H	1.3430370	-0.9412910	-2.6622330
H	3.0803990	-2.4536230	-2.3154730
H	3.0734570	-3.3687240	-0.1918570
H	4.8169820	-2.5655470	1.1450100
H	1.3324320	-2.5719860	1.1454470
H	3.0803990	2.4536230	2.3154720
H	3.0734570	3.3687240	0.1918560
H	1.9652960	1.1186140	-2.5883380
H	4.1597030	1.1176020	-2.5976590
H	4.1597030	-1.1176020	2.5976580
H	1.9652960	-1.1186140	2.5883370
P	-0.8266370	0.9970570	0.4648290
N	-1.4608440	1.0838370	1.9252160
P	-1.9816720	0.2497190	3.1753730
C	-0.8683450	-1.0640880	3.7324900
H	-0.6762800	-1.7327910	2.8910400
H	0.0675250	-0.6175180	4.0762860
H	-1.3255220	-1.6192350	4.5550170
C	-2.1579480	1.3927420	4.5631550
H	-2.8559470	2.1870810	4.2913650
H	-1.1846040	1.8323930	4.7904210
H	-2.5332110	0.8630790	5.4417050
C	-3.6139560	-0.4812000	2.9031010
H	-4.3316340	0.3174290	2.7031920
H	-3.9346130	-1.0487840	3.7797100
H	-3.5499840	-1.1335160	2.0319660
N	-1.5134170	1.9587990	-0.6336540
P	-2.1588330	3.4080290	-0.3759530
C	-3.7730020	3.3286030	0.4395310
H	-4.2000270	4.3286160	0.5436650
H	-4.4480190	2.7035880	-0.1488490
H	-3.6377890	2.8796370	1.4258220
C	-2.3915300	4.2009110	-1.9794060
H	-1.4219610	4.3049010	-2.4708490
H	-3.0460640	3.5870810	-2.6013770
H	-2.8408140	5.1873560	-1.8468980
C	-1.1429360	4.5225740	0.6291400
H	-1.0291170	4.0978240	1.6296270
H	-1.6227180	5.5008260	0.7076180
H	-0.1604500	4.6392020	0.1659420
P	-0.8266370	-0.9970570	-0.4648290
N	-1.4608450	-1.0838380	-1.9252160
P	-1.9816740	-0.2497190	-3.1753730
C	-3.6139580	0.4811990	-2.9031000
H	-3.5499850	1.1335150	-2.0319650
H	-3.9346160	1.0487820	-3.7797090
H	-4.3316350	-0.3174300	-2.7031900
C	-0.8683470	1.0640880	-3.7324910
H	-1.3255250	1.6192340	-4.5550180
H	0.0675220	0.6175180	-4.0762890
H	-0.6762810	1.7327900	-2.8910410
C	-2.1579510	-1.3927440	-4.5631540
H	-2.5332150	-0.8630810	-5.4417040
H	-2.8559490	-2.1870820	-4.2913630
H	-1.1846060	-1.8323940	-4.7904210
N	-1.5134170	-1.9587980	0.6336540
P	-2.1588330	-3.4080280	0.3759540
C	-2.3915280	-4.2009110	1.9794070
H	-2.8408120	-5.1873560	1.8469000
H	-1.4219570	-4.3049020	2.4708490
H	-3.0460590	-3.5870800	2.6013800
C	-1.1429380	-4.5225730	-0.6291410
H	-1.6227200	-5.5008250	-0.7076180
H	-1.0291200	-4.0978220	-1.6296280
H	-0.1604510	-4.6392010	-0.1659450
C	-3.7730030	-3.3286020	-0.4395280
H	-4.2000290	-4.3286160	-0.5436610
H	-3.6377920	-2.8796350	-1.4258180
H	-4.4480190	-2.7035880	0.1488540

H	2.6132500	0.0055290	1.8761940
H	4.4057090	-0.8528390	0.6601070
H	3.9915510	-0.9405000	-1.6121780
H	3.7490460	-3.1376780	-2.3696260
H	1.8792810	-0.2037160	-2.2230380
H	-1.2274470	-4.4036070	-0.5940550
H	-0.9096520	-4.1688190	1.6859690
H	1.3376120	-1.4444300	2.8176820
H	2.5496590	-3.2696210	2.7962760
H	1.6892250	-3.6489510	-2.7793360
H	0.5459030	-1.7834150	-2.6893340
P	-1.6994710	0.0523150	0.0694410
N	-2.8599990	-0.2689270	-1.1151660
P	-4.3497200	-0.7729920	-0.9249910
C	-4.6343860	-2.2189200	0.1503590
H	-4.2764860	-1.9703400	1.1521910
H	-5.6975840	-2.4716070	0.1877260
H	-4.0710880	-3.0744340	-0.2306970
C	-5.4957240	0.4846730	-0.2746020
H	-5.4378460	1.3786770	-0.9000180
H	-5.1797550	0.7371000	0.7389170
H	-6.5242980	0.1155340	-0.2581120
C	-5.0284210	-1.2363440	-2.5413790
H	-4.4452910	-2.0628770	-2.9533640
H	-4.9616950	-0.3819730	-3.2183100
H	-6.0733790	-1.5415900	-2.4464330
N	-2.5222120	0.1466610	1.5608690
P	-2.5488270	1.3655300	2.5680490
C	-1.1821120	1.4201400	3.7772260
H	-1.3495840	2.2055480	4.5188380
H	-1.1233650	0.4530700	4.2827140
H	-0.2324850	1.6091460	3.2708840
C	-4.0371270	1.2722730	3.6005240
H	-4.0408860	2.0729070	4.3440250
H	-4.9268170	1.3608880	2.9736150
H	-4.0578280	0.3063500	4.1097620
C	-2.5781200	3.0356130	1.8327010
H	-3.4447570	3.1200130	1.1722840
H	-1.6736150	3.1977090	1.2410590
H	-2.6380670	3.8005480	2.6106730
P	0.9776460	1.7355590	0.2175970
N	2.0566610	2.5814240	1.0996080
P	3.1403980	3.6191710	0.5508730
C	2.4345410	5.1870010	-0.0247650
H	1.7664880	4.9713960	-0.8608590
H	1.8597440	5.6397890	0.7859300
H	3.2229670	5.8730910	-0.3426340
C	4.2709730	4.0222000	1.9002270
H	3.7009640	4.4394550	2.7327460
H	5.0141880	4.7501280	1.5676410
H	4.7745110	3.1117560	2.2312870
C	4.1686210	3.0231490	-0.8247970
H	3.5204560	2.7860170	-1.6724060
H	4.7079310	2.1232130	-0.5186640
H	4.8881690	3.7896640	-1.1229420
N	0.5989550	2.5305060	-1.1219180
P	-0.4168990	2.5363940	-2.3479670
C	0.2055760	3.7796410	-3.5083250
H	-0.4342350	3.8327210	-4.3918970
H	0.2241800	4.7545400	-3.0165280
H	1.2207430	3.5114560	-3.8086110
C	-2.1098890	3.0390560	-1.9422850
H	-2.6434180	3.2901410	-2.8630670
H	-2.6164640	2.2079450	-1.4452660
H	-2.0796770	3.9152610	-1.2914240
C	-0.5718830	1.0072960	-3.3075220
H	0.4156310	0.6762120	-3.6342500
H	-1.1917760	1.2059990	-4.1861520
H	-1.0568500	0.2412560	-2.6977270
H	-0.0503600	1.5628530	1.1359590

III(Pb_2)$_2$ (dication)

C	1.3298950	-1.3179610	-1.0181920
C	2.2004030	-0.4150970	-1.9054150
C	3.4439150	-1.3112540	-2.0788790
C	3.4813390	-2.2894330	-0.8613090
C	2.2602530	-1.9599450	0.0253330
C	2.7104620	0.9937950	-1.4737260
C	4.2807990	0.9714170	-1.5536190
C	4.7212120	-0.4559090	-2.0115030
C	2.3131820	1.7456080	-0.1631190
C	3.6251060	2.0311670	0.5990850
C	4.8369190	1.7008490	-0.2906130
C	1.4664370	1.1325030	0.9670020
C	2.3895330	0.1980740	1.7646380
C	3.6734910	1.0526270	1.8155300
C	4.9096660	0.1562420	1.6253870

III(Pb_2)$_2$ (protonated)

C	-1.5711350	5.6458980	-0.5670190
C	-1.4913160	4.6019210	-1.6921890
C	-2.2735530	3.4252520	-1.0902370
C	-2.2234140	3.5891830	0.4647030
C	-1.4378810	4.8773040	0.7557860
C	-1.5734910	2.0905410	-1.4113630
C	-0.1644080	2.4460240	-1.9844960
C	-0.1382990	4.0094410	-2.2019940
C	1.2409190	2.0262850	-1.4241530
C	2.0141720	3.3465180	-1.1703730
C	1.2574680	4.5433640	-1.7623420
C	2.0669120	3.5605980	0.3800770
C	1.3111570	4.8607440	0.6809980
C	1.4117140	5.6028400	-0.6612900
C	1.5867770	1.3183700	-0.0925000

C	4.3798400	-1.2546800	1.2144970
C	2.8097810	-1.2248530	1.2882860
C	4.7850750	-2.0005530	-0.0952200
C	5.7865810	1.0089500	0.6970830
C	5.6560180	-1.3382080	-1.1717680
P	-0.3060730	0.7856150	0.7562080
P	-0.3985650	-0.8925070	-0.6646220
N	-1.0667150	-2.1152600	0.1522710
P	-1.9854480	-3.2725190	-0.4887280
C	-1.2178270	-4.1689530	-1.8780910
N	-0.9392880	0.4793760	2.1836510
P	-1.2263110	-0.5966350	3.3209280
C	-2.9094830	-1.2758670	3.2172800
N	-0.9793880	2.0347540	-0.0138940
P	-1.5524960	3.3788890	0.6643330
C	-3.1702980	3.1502150	1.4672960
C	-0.0890500	-2.0195250	3.3435020
C	-1.0903280	0.2542310	4.9232450
C	-1.7568420	4.6143960	-0.6515290
C	-0.4558680	4.0938560	1.9355200
N	-1.1086690	-0.5635210	-2.0486850
P	-1.5522640	0.5269320	-3.1171130
C	-3.1890430	1.2189230	-2.7101690
C	-0.3574310	1.8899100	-3.2952850
C	-1.6861960	-0.3100100	-4.7257850
C	-3.5716510	-2.6536360	-1.1426730
C	-2.3144350	-4.4731060	0.8349740
H	1.9716340	0.0977140	2.7695660
H	1.8342110	2.6809960	-0.4647830
H	5.4362910	0.0330100	2.5758130
H	5.3131510	2.6220390	-0.6377620
H	1.3422580	1.9800640	1.6614150
H	6.6456840	0.5090860	0.2598560
H	6.2023770	1.8069880	1.3257730
H	6.5689210	-0.8670490	-0.8203330
H	5.9828940	-2.1480960	-1.8367350
H	1.1040870	-2.1614210	-1.6937400
H	5.1564150	-0.3486830	-3.0089780
H	1.6872780	-0.2990400	-2.8635030
H	3.3859220	-1.8762710	-3.0128310
H	3.4393260	-3.3249670	-1.2099150
H	5.2625660	-2.9373490	0.2052690
H	1.7796600	-2.8764980	0.3783270
H	3.7245750	1.6172350	2.7501770
H	3.6515510	3.0666320	0.9487050
H	2.3692380	1.6596210	-2.2693150
H	4.5555920	1.6210560	-2.3878150
H	4.7127480	-1.9145190	2.0189930
H	2.5280130	-1.8809500	2.1141430
H	-0.0434930	-2.3730530	2.3081990
H	0.8975290	-1.6271040	3.6061910
C	-0.4842320	-3.1516800	4.2942920
H	-1.3488730	-0.4594230	5.7119810
H	-1.8610820	1.0315340	4.9244850
C	0.2897940	0.8696770	5.1585320
H	-3.0777640	-1.8976510	4.1020140
H	-2.8966980	-1.9341980	2.3445430
C	-4.0000810	-0.2182250	3.0700380
H	-2.3414380	5.4449260	-0.2437270
C	-0.4311950	5.1096490	-1.2334740
H	-2.3740040	4.1477240	-1.4258740
H	0.5349350	4.1886850	1.4784070
C	-0.9139720	5.4349700	2.5139350
H	-0.3772620	3.3375670	2.7247120
C	-4.2197940	2.5065470	0.5619390
H	-3.5096380	4.1274350	1.8243030
H	-2.9759510	2.5265050	2.3464320
H	-0.1432780	2.2022800	-2.2681700
H	0.5560860	1.4459270	-3.7026250
C	-0.8041270	3.0764670	-4.1495670
H	-1.9930320	0.4287790	-5.4730050
H	-2.5034620	-1.0331010	-4.6334430
C	-0.3899300	-1.0084560	-5.1397860
C	-4.0422410	1.7304750	-3.8724510
H	-2.9997590	2.0000700	-1.9695170
H	-3.7103800	0.4146450	-2.1812700
H	-4.2044980	-3.5146350	-1.3790150
C	-4.2777830	-1.6814560	-0.1995110
H	-3.3237440	-2.1622350	-2.0881710
C	-3.0933120	-5.7155030	0.3995400
H	-1.3366870	-4.7409590	1.2486130
H	-2.8524350	-3.9404620	1.6248020
H	-1.0013480	-3.4073050	-2.6355080
H	-1.9753800	-4.8327410	-2.3077150
C	0.0417050	-4.9480330	-1.4954310
H	-0.0566420	-1.7057580	-4.3662620
H	-0.5486920	-1.5712320	-6.0619480
C	1.3466840	2.3550950	1.0172540
C	-0.0617280	2.8629200	1.4746950
C	-0.0516460	4.4340470	1.3137830
C	-1.4766740	2.3538520	1.0197280
C	-1.7907610	1.3403820	-0.0974010
P	1.7132950	-0.4667750	0.3265020
N	3.0127540	-0.5463740	1.3086560
P	4.5098280	-0.9048340	0.8781190
C	4.7244660	-2.6179490	0.2684730
C	3.8647280	-3.6518390	0.9953290
P	-1.4459980	-0.4826540	0.0026560
N	-2.4786910	-1.0409000	-1.2097070
P	-3.9789450	-1.5252920	-1.0282800
C	-4.2267480	-3.0027010	0.0432580
C	-3.0473640	-3.9731200	0.0505690
N	1.9196990	-1.3774040	-0.9757670
P	1.1971220	-2.0221650	-2.2394010
C	0.2928580	-0.8529260	-3.3066790
C	-0.1626710	-1.4129740	-4.6560710
C	2.5120010	-2.7727580	-3.2521430
C	3.5323950	-1.7604610	-3.7715700
C	0.0198880	-3.3590650	-1.8691780
C	0.5984980	-4.4958090	-1.0292110
N	-2.2649810	-0.7872600	1.4586130
P	-1.8605970	-1.1684500	2.9297490
C	-0.6023980	-0.0866030	3.7200940
C	-0.1658360	-0.4787970	5.1320760
C	-3.3473790	-1.0528740	3.9820660
C	-3.9671560	0.3442660	3.9909090
C	-1.2700920	-2.8839840	3.1975120
C	0.0339630	-3.1933090	2.4636250
C	-4.6254420	-1.9618650	-2.6786860
C	-3.8310510	-3.0640630	-3.3805150
C	-5.1600720	-0.2939980	-0.3519330
C	-6.6091070	-0.7570190	-0.1871000
C	5.5822450	-0.6475950	2.3241320
C	5.1939610	-1.4653570	3.5571200
C	5.1862030	0.1685570	-0.4398920
C	6.5613580	-0.2263890	-0.9823040
H	-1.9774780	1.9927670	1.9224080
H	-2.1127370	1.5538180	-2.1971000
H	-1.9316520	5.4576030	1.5412460
H	-2.0140520	5.0028210	-2.5659990
H	-2.8889040	1.2886960	-0.0297440
H	-0.9391650	6.5225220	-0.6751080
H	-2.6011340	6.0265160	-0.5899440
H	0.7977890	6.4919600	-0.7678320
H	2.4493750	5.9565220	-0.7271010
H	2.6853630	1.3716620	-0.1660170
H	1.7642330	4.9065440	-2.6614300
H	1.7415200	1.4649680	-2.2176860
H	3.0300970	3.2735480	-1.5700570
H	3.1073080	3.6076850	0.7155340
H	1.8459650	5.4464420	1.4346510
H	1.8822100	2.0268370	1.9129460
H	-3.2389900	3.6375230	0.8688150
H	-3.3124870	3.4127700	-1.4334090
H	-0.1705710	2.0031290	-2.9834530
H	-0.1653480	4.1673360	-3.2833670
H	-0.0178480	4.8440360	2.3265890
H	-0.0595750	2.6698380	2.5504870
H	0.2630350	-0.0519140	3.0506460
H	-1.0303550	0.9210060	3.7127420
H	-3.0915670	-1.3803960	4.9950370
H	-4.0543300	-1.7854170	3.5775420
H	-1.1698820	-3.0610620	4.2736000
H	-2.0748090	-3.5349840	2.8391270
H	-5.1478420	-3.5021550	-0.2771710
H	-4.3918270	-2.6193000	1.0547130
H	-5.6771450	-2.2480880	-2.5763470
H	-4.5968030	-1.0358810	-3.2629040
H	-5.1000580	0.5831330	-1.0061090
H	-4.7296840	-0.0048240	0.6130380
H	-0.5685600	-0.5176830	-2.7191720
H	0.9541270	0.0045220	-3.4563840
H	2.0373070	-3.3153230	-4.0761580
H	2.9986620	-3.5200670	-2.6168350
H	-0.3417280	-3.7405020	-2.8306490
H	-0.8328580	-2.8724370	-1.3841320
H	5.7887270	-2.8691730	0.3244890
H	4.4468590	-2.5867050	-0.7898010
H	6.6128130	-0.8596910	2.0220240
H	5.5228540	0.4244770	2.5400670
H	5.2012980	1.1857450	-0.0326480
H	4.4399840	0.1605390	-1.2424810
H	0.6941660	-0.8630810	1.1784850
H	-4.9144670	0.3385470	4.5356110

H	0.4138290	-0.2901350	-5.3238260	H	-3.3086830	1.0711120	4.4759900
H	0.0158920	3.7935480	-4.2307610	H	-4.1563340	0.6863770	2.9698020
H	-1.0818380	2.7754950	-5.1633900	H	0.5333810	0.2629400	5.5262390
H	-1.6531110	3.5991920	-3.7010180	H	-1.0146120	-0.5312450	5.8199750
H	-4.3057240	0.9217880	-4.5582160	H	0.3401200	-1.4481590	5.1426800
H	-4.9717050	2.1490050	-3.4795610	H	0.3025060	-4.2466840	2.5766770
H	-3.5417920	2.5132170	-4.4458370	H	-0.0684990	-2.9868780	1.3935580
H	-1.8749340	5.3489190	3.0264350	H	0.8674400	-2.5928500	2.8432280
H	-0.1802270	5.7857880	3.2425910	H	5.7758440	-1.1339830	4.4203170
H	-1.0048740	6.2015010	1.7396790	H	4.1334220	-1.3425190	3.7904440
H	0.1929410	4.2792200	-1.5733840	H	5.3954410	-2.5291090	3.4104210
H	-0.6215000	5.7603410	-2.0892890	H	3.9343610	-4.6166420	0.4867860
H	0.1392460	5.6826700	-0.4977880	H	4.1777940	-3.7932030	2.0319950
H	-4.4636520	3.1439100	-0.2928700	H	2.8176900	-3.3413900	0.9973830
H	-3.8711220	1.5428460	0.1815320	H	6.8829730	0.4967530	-1.7353410
H	-5.1414710	2.3362530	1.1227680	H	7.3199700	-0.2445180	-0.1952500
H	-4.0435120	0.4478640	3.9358580	H	6.5380080	-1.2110530	-1.4565860
H	-3.8294150	0.3863720	2.1778010	H	4.3216490	-2.2704470	-4.3283470
H	-4.9751140	-0.7005020	2.9704320	H	3.0701630	-1.0281130	-4.4399400
H	0.5612920	1.5435750	4.3408820	H	3.9968210	-1.2172220	-2.9446700
H	0.2888760	1.4457100	6.0861700	H	-0.7232670	-0.6461420	-5.1960790
H	1.0654250	0.1034030	5.2446280	H	0.6826980	-1.7089540	-5.2834540
H	-0.5990870	-2.8042740	5.3248100	H	-0.8202420	-2.2785900	-4.5417080
H	-1.4181170	-3.6286470	3.9867230	H	-0.1977040	-5.1856830	-0.7376480
H	0.2951130	-3.9169050	4.2885680	H	1.3505140	-5.0656800	-1.5819900
H	-3.2436200	-6.3709400	1.2596560	H	1.0662370	-4.1194200	-0.1173470
H	-4.0787270	-5.4578890	0.0031230	H	-3.2493070	-4.8100300	0.7251970
H	-2.5568430	-6.2862610	-0.3629270	H	-2.8402820	-4.3820800	-0.9417980
H	-3.6118400	-0.8591510	0.0792060	H	-2.1452560	-3.4638870	0.3977500
H	-5.1598570	-1.2586280	-0.6858760	H	-4.1516950	-3.1630150	-4.4206790
H	-4.6111310	-2.1761160	0.7171470	H	-2.7624970	-2.8347920	-3.3679840
H	0.5024770	-5.3721660	-2.3897420	H	-3.9782050	-4.0321330	-2.8938180
H	0.7810730	-4.3066600	-1.0083460	H	-7.2122850	0.0468630	0.2432080
H	-0.1862940	-5.7714610	-0.8133680	H	-7.0616820	-1.0314060	-1.1439960
				H	-6.6828540	-1.6186550	0.4822090
**III(Pc_2)$_2$ (dication)**				**III(Pc_2)$_2$ (protonated)**			
C	-4.8891070	-3.3277600	2.1214350	C	-0.3016670	5.7731200	-1.8839050
C	-4.0336860	-3.6446700	0.8867710	C	-0.5404070	4.5071790	-2.7212530
C	-2.6067490	-3.3889310	1.4040910	C	-1.5066810	3.7128920	-1.8310090
C	-2.7339070	-2.4258630	2.6264030	C	-1.3055530	4.2249720	-0.3664290
C	-4.2311690	-2.1261810	2.8154170	C	-0.2336720	5.3235750	-0.4162500
C	-1.7767750	-2.6521330	0.3317480	C	-1.1352830	2.2159690	-1.8236920
C	-2.7752420	-2.2187310	-0.7883190	C	0.2682300	2.1007710	-2.5062100
C	-4.1763430	-2.8455490	-0.4467070	C	0.6116620	3.5245780	-3.0990450
C	-3.0235670	-0.7589170	-1.2789020	C	1.5987220	1.5159260	-1.9174890
C	-4.5254300	-0.4652190	-1.0793410	C	2.6463090	2.6501530	-1.9949770
C	-5.2691520	-1.7608610	-0.7101810	C	2.1184530	3.8229810	-2.8303570
C	-4.6474470	0.4994570	0.1427200	C	2.8537470	3.1817870	-0.5370070
C	-5.4635670	-0.2412030	1.2173870	C	2.4284600	4.6544770	-0.5306660
C	-6.2303910	-1.2787780	0.3858850	C	2.5845790	5.0364580	-2.0107360
C	-2.4075140	0.4864290	-0.6221430	C	1.8836720	1.0533010	-0.4710370
C	-3.2164110	0.7676690	0.6546860	C	1.9364820	2.3386920	0.3787250
C	-3.0565930	-0.0371620	1.9820210	C	0.7133160	3.2583130	0.7368460
C	-4.4578450	-0.6561200	2.3371190	C	1.0562600	4.7067840	0.2074160
C	-1.9808170	-1.1299190	2.2637510	C	-0.8146480	3.0169040	0.4618350
C	-0.9909410	-1.6690950	1.2200120	C	-1.4327760	1.8738530	-0.3610420
P	-0.6316440	0.8441530	-0.7183020	P	1.6296680	-0.6637890	0.1543880
N	-0.3850140	2.2361140	0.0454630	N	2.9717650	-1.1264840	0.9381640
P	0.1623210	3.6295990	-0.5271750	P	4.5423830	-1.1647240	0.6785760
C	1.7906030	3.5152390	-1.3350890	C	5.2565380	-2.3354380	1.8724870
C	2.8810050	2.9760620	-0.4076770	C	6.7860770	-2.3956370	1.9298160
C	4.2146290	2.7791570	-1.1210000	C	7.2658540	-3.4263960	2.9500390
P	0.3658440	-0.6772400	0.5230810	P	-1.5138710	0.1198660	0.2425570
N	1.2930260	-0.0506120	1.6644000	N	-2.6482620	-0.5183240	-0.8293450
P	1.3611810	0.7894760	3.0176740	P	-4.2250040	-0.5994950	-0.6771720
C	-0.1333350	1.7660660	3.3807250	C	-4.7794840	-1.6284300	0.7287510
C	0.0313250	2.9253540	4.3692900	C	-6.2688080	-1.9585600	0.8308050
C	-1.3038360	3.6308620	4.5989120	C	-6.5492800	-2.8652900	2.0286700
N	-0.1365920	0.8246180	-2.2299690	N	1.2669570	-1.5219710	-1.1380540
P	-0.0692530	0.0357530	-3.6106070	P	0.3754820	-2.7149470	-1.6767020
C	-0.1566280	-1.7777970	-3.4820820	C	1.4910350	-4.0097410	-2.3032510
C	0.3307280	-2.5519430	-4.7118860	C	2.3693670	-4.5889800	-1.1890820
C	0.1197210	-4.0543670	-4.5339270	C	3.5139690	-5.4394470	-1.7331460
C	-1.4080880	0.5247300	-4.7504800	C	-0.7503270	-3.4744800	-0.4616070
C	-1.7964460	2.0018080	-4.6583240	C	-1.3929790	-4.7883710	-0.9240420
C	-2.9005170	2.3636520	-5.6480510	C	-2.4505520	-5.2785250	0.0609240
C	1.5004440	0.4747600	-4.4106220	C	-0.6853180	-2.1584620	-3.0405670
C	2.7120510	0.0722620	-3.5673760	C	0.0592420	-1.4328050	-4.1630010
C	4.0290050	0.5230680	-4.1914840	C	-0.9142770	-0.7378070	-5.1125420
N	1.1680140	-1.6298210	-0.4968570	N	-2.3550940	0.4280430	1.7032170
P	2.5846920	-2.3472510	-0.2674670	P	-1.6826360	0.1491730	3.1134590
C	2.7456640	-3.5781240	-1.5909510	C	-2.8896690	0.5242830	4.4246620
C	4.0687600	-4.3464400	-1.6294740	C	-3.4764960	1.9357300	4.3381320
C	4.0736060	-5.3749050	-2.7585730	C	-4.5485470	2.1737910	5.3992600
C	2.7114600	-3.2299930	1.3234650	C	-1.1223340	-1.5639090	3.4625510
C	1.7346840	-4.4094990	1.4278280	C	-2.1371070	-2.6395170	3.0702840

C	1.6062650	-4.9197720	2.8604130	C	-1.6846590	-4.0327440	3.4983130
C	3.9861030	-1.1823290	-0.4134090	C	-0.2025340	1.1782670	3.4487330
C	5.2404840	-1.4883640	0.4104450	C	0.6423200	0.8658040	4.6845350
C	6.3630610	-0.5042720	0.0917170	C	1.7794770	1.8750420	4.8409090
C	1.7194220	-0.2958720	4.4378330	C	-4.8662960	-1.3943200	-2.1865880
C	0.7000580	-1.4047840	4.7003450	C	-4.3828350	-2.8420710	-2.3284630
C	1.1760610	-2.3680950	5.7848760	C	-4.5843630	-3.3932420	-3.7369040
C	2.7543540	1.9582450	2.9936530	C	-5.1195530	0.9874700	-0.4914840
C	4.0901630	1.3660470	2.5411250	C	-4.9690370	1.9203480	-1.6978720
C	5.2033890	2.4101950	2.5721280	C	-5.3239980	3.3647870	-1.3540230
C	0.2932330	4.7289680	0.9104300	C	5.3256540	0.4566830	0.9846980
C	0.8715960	6.1205890	0.6470560	C	5.0300380	0.9831560	2.3942460
C	0.9565840	6.9313870	1.9386440	C	5.3837260	2.4592920	2.5520720
C	-0.9332680	4.4060480	-1.7527380	C	5.0976030	-1.7133330	-0.9722810
C	-2.3805230	4.5620100	-1.2752820	C	4.5437420	-0.9054980	-2.1504320
C	-3.2990570	5.0380820	-2.3980030	C	5.0495500	-1.4395410	-3.4884350
H	-1.4067850	-0.7952880	3.1273880	H	-1.3061650	3.0111390	1.4384380
H	-1.0540660	-3.3270580	-0.1331770	H	-1.8291310	1.6434420	-2.4462800
H	-4.4918110	-2.1586870	3.8768820	H	-0.5225270	6.1699690	0.2142450
H	-4.1630930	-4.7023020	0.6409190	H	-1.0315840	4.8017030	-3.6537180
H	-0.3563560	-2.3463940	1.8177730	H	-2.5061630	2.1115940	-0.2764250
H	-5.9590560	-3.2540750	1.9522130	H	0.4931230	6.4314870	-2.2218920
H	-4.7595850	-4.1772980	2.8043950	H	-1.2234220	6.3639780	-1.9708720
H	-6.7415440	-2.0548360	0.9473950	H	2.1742000	5.9979750	-2.3031790
H	-7.0231050	-0.7238920	-0.1324560	H	3.6659400	5.1169310	-2.1849450
H	-2.7267250	1.2988150	-1.2985590	H	2.9630600	0.8648630	-0.5808780
H	-5.8644170	-2.1093140	-1.5587080	H	2.6207320	3.8538450	-3.8017690
H	-2.7858690	-0.7308720	-2.3454780	H	1.8936070	0.6956530	-2.5750740
H	-4.9487330	0.0027460	-1.9718700	H	3.5931340	2.2690230	-2.3904810
H	-5.1313630	1.4314770	-0.1610910	H	3.9030450	3.0704730	-0.2467390
H	-6.1891700	0.4367980	1.6752330	H	3.1344740	5.2518460	0.0541270
H	-3.0833210	1.8237450	0.9017390	H	2.4514920	2.1114680	1.3192280
H	-2.2978140	-2.8878250	3.5167040	H	-2.2522000	4.6038110	0.0312360
H	-2.1132880	-4.3169910	1.7057730	H	-2.5471790	3.8556480	-2.1381880
H	-2.4113420	-2.7430290	-1.6747620	H	0.0791930	1.4538370	-3.3664010
H	-4.3636040	-3.6098710	-1.2044010	H	0.5417240	3.4272650	-4.1856630
H	-4.8091110	-0.1311200	3.2284240	H	1.2490670	5.3151530	1.0948630
H	-2.8579760	0.7338220	2.7302700	H	0.7662050	3.3206880	1.8262880
H	-0.4853530	2.1242680	2.4051970	H	-3.6894530	-0.2207780	4.3383720
H	-0.8873330	1.0687330	3.7587010	H	-2.4049370	0.3616820	5.3945090
H	1.8328950	0.3446510	5.3202040	H	-0.8918960	-1.6244950	4.5333920
H	2.7034550	-0.7348740	4.2354320	H	-0.1771560	-1.7256030	2.9294340
H	2.8411440	2.3662540	4.0071770	H	0.4160380	1.1003240	2.5465360
H	2.4721600	2.7900960	2.3418060	H	-0.5550650	2.2160360	3.4798580
H	2.5918600	-3.0387810	-2.5326470	H	-4.4211530	-1.1078030	1.6227500
H	1.8973510	-4.2629360	-1.4795790	H	-4.1928680	-2.5519160	0.6572550
H	2.5113620	-2.4793520	2.0976400	H	-5.9604460	-1.3355030	-2.2007850
H	3.7411130	-3.5722410	1.4683240	H	-4.4969940	-0.7885570	-3.0221880
H	3.5751550	-0.2055070	-0.1388000	H	-4.6862410	1.4422090	0.4078720
H	4.2324090	-1.1307140	-1.4803870	H	-6.1779300	0.7938230	-0.2829520
H	0.4017560	-2.0460640	-2.5793480	H	0.9101510	-4.7899930	-2.8069430
H	-1.2059000	-2.0205200	-3.2822000	H	2.1134120	-3.5235040	-3.0639550
H	-2.2762800	-0.1051230	-4.5248270	H	-1.5142260	-2.7090760	-0.2728900
H	-1.0846670	0.2654760	-5.7654750	H	-0.1893240	-3.6220740	0.4686240
H	1.4804990	1.5598870	-4.5639010	H	-1.4159160	-1.4928590	-2.5571270
H	1.5318500	0.0089580	-5.4012940	H	-1.2344510	-3.0223540	-3.4340480
H	2.0536670	4.5055600	-1.7233150	H	4.8370350	-3.3168490	1.6223820
H	1.6466800	2.8506540	-2.1945390	H	4.8430830	-2.0583670	2.8486000
H	-0.7141970	4.7968290	1.3368310	H	6.4032780	0.3770430	0.8052020
H	0.9016740	4.1941350	1.6476670	H	4.9336820	1.1485700	0.2306910
H	-0.8839230	3.7635400	-2.6389070	H	4.8044530	-2.7657670	-1.0648630
H	-0.5043380	5.3736130	-2.0367440	H	6.1938010	-1.6870590	-0.9702260
H	-2.1341740	2.2085700	-3.6370950	H	0.6865180	-0.7414930	1.1686640
H	-0.9183340	2.6337250	-4.8362280	H	-3.1121450	-2.4197900	3.5213680
H	-0.1979790	-2.2124290	-5.6096370	H	-2.2871810	-2.6089250	1.9865940
H	1.3950560	-2.3526920	-4.8812070	H	-2.6761490	2.6756900	4.4558360
H	2.5979210	0.4936530	-2.5623630	H	-3.8954320	2.0834890	3.3379590
H	2.7227280	-1.0170620	-3.4438930	H	0.0166400	0.8756640	5.5846880
H	0.7462670	-4.1162560	1.0556250	H	1.0597460	-0.1434170	4.6015930
H	2.0779100	-5.2213050	0.7776210	H	3.9670410	0.8335720	2.6149330
H	4.8995160	-3.6462910	-1.7706220	H	5.5856930	0.3887720	3.1285570
H	4.2382820	-4.8517910	-0.6717100	H	7.1906210	-2.6458410	0.9430490
H	4.9969020	-1.4376730	1.4770790	H	7.1869440	-1.4106180	2.1938870
H	5.5864090	-2.5090260	0.2143210	H	3.4505710	-0.9550150	-2.1365920
H	4.3654870	0.5208380	3.1811360	H	4.8281250	0.1488230	-2.0476340
H	3.9793610	0.9669880	1.5288790	H	-1.8548570	-4.6556430	-1.9090250
H	-0.2596040	-0.9684700	4.9975290	H	-0.6231740	-5.5586090	-1.0446200
H	0.5236640	-1.9613550	3.7726640	H	2.7714940	-3.7625500	-0.5910650
H	0.4235340	2.5579370	5.3241570	H	1.7531930	-5.1899900	-0.5101210
H	0.7598410	3.6486010	3.9871760	H	0.7353520	-0.6927030	-3.7231340
H	1.8702000	6.0320740	0.2046630	H	0.6847530	-2.1391110	-4.7207720
H	0.2492970	6.6519970	-0.0812080	H	-6.8537040	-1.0373000	0.9275380
H	3.0236830	3.6753400	0.4249710	H	-6.6115180	-2.4506510	-0.0870440
H	2.5416740	2.0278550	0.0276350	H	-3.3199480	-2.8815120	-2.0704990
H	-2.7419710	3.6042570	-0.8837560	H	-4.9053800	-3.4772150	-1.6032560
H	-2.4186390	5.2721960	-0.4418000	H	-3.9377810	1.8823700	-2.0651500
H	-0.9403610	-4.2861570	-4.3915160	H	-5.6038760	1.5634850	-2.5165860

H	0.4735450	-4.6043960	-5.4096500
H	0.6643160	-4.4250180	-3.6598250
H	4.8783920	0.2355740	-3.5640150
H	4.1702150	0.0708970	-5.1775950
H	4.0527980	1.6108000	-4.3127660
H	-2.5801640	2.1833230	-6.6786670
H	-3.7995010	1.7668660	-5.4646270
H	-3.1716350	3.4194460	-5.5597480
H	2.5806760	-5.2126660	3.2631310
H	1.1917170	-4.1476240	3.5160650
H	0.9442550	-5.7882710	2.9052580
H	6.0549520	0.5239530	0.3049050
H	7.2526790	-0.7191120	0.6890420
H	6.6404660	-0.5558840	-0.9658700
H	3.2690050	-6.1045370	-2.6243310
H	3.9288120	-4.8884730	-3.7284300
H	5.0221320	-5.9168510	-2.7889430
H	6.1500230	1.9816680	2.2317890
H	4.9643240	3.2604220	1.9249500
H	5.3521670	2.7937710	3.5859540
H	2.1180760	-2.8454410	5.4963410
H	1.3393870	-1.8438020	6.7311770
H	0.4363400	-3.1547950	5.9557990
H	-1.1900690	4.4652970	5.2955260
H	-1.7018760	4.0258940	3.6584610
H	-2.0456530	2.9403960	5.0124570
H	-4.3246130	5.1555040	-2.0385530
H	-2.9664200	6.0013030	-2.7967990
H	-3.3115940	4.3192870	-3.2237310
H	-0.0337210	7.0541110	2.3880800
H	1.5985220	6.4305380	2.6704560
H	1.3694270	7.9254730	1.7496810
H	4.5612670	3.7137550	-1.5718520
H	4.9829490	2.4410910	-0.4199350
H	4.1334030	2.0324340	-1.9149550

H	3.1358580	-6.2810800	-2.3217580
H	4.1668470	-4.8460220	-2.3826010
H	4.1238470	-5.8417560	-0.9194110
H	-2.0101320	-5.4667120	1.0439530
H	-3.2442950	-4.5342790	0.1849080
H	-2.9116680	-6.2062520	-0.2882160
H	-0.3822280	-0.2120270	-5.9103850
H	-1.5953600	-1.4587710	-5.5767430
H	-1.5230950	-0.0055960	-4.5703960
H	-4.2436020	-4.4306990	-3.8042680
H	-5.6393120	-3.3642730	-4.0280840
H	-4.0183350	-2.8040580	-4.4664140
H	-5.2390700	4.0126910	-2.2314690
H	-6.3473760	3.4423340	-0.9725830
H	-4.6483190	3.7518520	-0.5828620
H	-5.9985640	-3.8077470	1.9396900
H	-6.2370310	-2.3837780	2.9613350
H	-7.6140890	-3.1013600	2.1086380
H	-0.7418290	-4.3066130	3.0122690
H	-1.5269030	-4.0786570	4.5807500
H	-2.4324710	-4.7862560	3.2357610
H	1.3873590	2.8874770	4.9810380
H	2.4115070	1.6331410	5.6997340
H	2.4141520	1.8866750	3.9478100
H	-4.9563960	3.1857600	5.3246720
H	-5.3766950	1.4669320	5.2833520
H	-4.1400800	2.0479850	6.4072840
H	5.1926960	2.7968470	3.5744900
H	6.4390950	2.6402570	2.3260520
H	4.7836700	3.0788880	1.8771360
H	4.7462130	-2.4823460	-3.6299700
H	4.6405230	-0.8556150	-4.3174490
H	6.1416000	-1.3962550	-3.5480410
H	6.9057920	-4.4275860	2.6933490
H	8.3578840	-3.4587190	2.9877130
H	6.8992060	-3.1836710	3.9523520

III(Pd$_2$)$_2$ (dication)

C	0.0278010	3.5864800	-1.1186020
C	0.7604650	3.2678830	0.2231660
C	0.6011190	4.5135190	1.1212400
C	-0.0036180	5.6751290	0.3114650
C	-0.4481730	5.0826230	-1.0623060
C	-0.4312490	4.1491700	2.2356270
C	-0.8681850	2.6887700	1.9941540
C	0.3436960	2.1632450	1.2123860
C	-1.6347320	5.0923820	2.0609070
C	-2.7949290	4.2302210	1.4669880
C	-2.3092570	2.7368770	1.4105370
C	-1.0124610	6.2729750	1.3014060
C	-3.5315520	4.5447550	0.1276830
C	-3.3616650	3.3078190	-0.7726240
C	-2.7447030	2.1440140	0.0342780
C	-2.3382870	3.6924550	-1.8876420
C	-1.9064520	5.1455790	-1.6212830
C	-3.0837160	5.6853590	-0.7962590
C	-1.1284900	2.7486830	-1.7385920
C	-1.7838140	1.5610200	-1.0196090
H	1.8112750	3.1044280	-0.0309790
H	-0.9142800	2.1483050	2.9434060
H	0.7614400	6.4278390	0.1022180
H	-1.9753460	5.4536460	3.0351410
H	1.1393460	2.2131820	1.9725570
H	-1.7098870	6.9957820	0.8889170
H	-0.4198980	6.8274400	2.0405090
H	-2.9312250	6.6491520	-0.3200080
H	-3.9046380	5.8383320	-1.5086920
H	-2.4791270	1.1795310	-1.7863410
H	-4.5875040	4.6922760	0.3706830
H	-3.5007520	1.3852140	0.2510070
H	-4.3103390	3.0066330	-1.2241230
H	-2.7969730	3.5821970	-2.8741430
H	-1.8619410	5.7042110	-2.5602350
H	-0.7964880	2.4019930	-2.7208270
H	1.5577650	4.7922300	1.5726240
H	0.0259180	4.2591900	3.2228850
H	-2.9155560	2.1880140	2.1338150
H	-3.5787070	4.2704130	2.2268440
H	0.1488530	5.6233320	-1.8004390
H	0.8239640	3.5425290	-1.8645640
P	0.5534230	0.3980050	0.8350000
N	0.2969380	-0.4364530	2.1629870
P	-0.7730130	-1.2078650	3.0529790
C	-0.8641570	-2.9779350	2.6531940
H	-1.4601200	-3.0544740	1.7387040
H	-1.4249170	-3.4628760	3.4593330
C	0.4996410	-3.6389750	2.4352810

III(Pd$_2$)$_2$ (protonated)

C	-1.3168280	-0.3388680	-2.0451220
C	-0.8590000	0.5426200	-3.2151400
C	-1.4342920	-0.2782690	-4.3849090
C	-1.5218920	-1.7601060	-3.8891350
C	-0.9702950	-1.7915340	-2.4406780
C	0.6392040	0.8327710	-3.5570760
C	0.8534630	0.3862250	-5.0570660
C	-0.4709440	-0.2592960	-5.5785300
C	1.9254530	0.4169270	-2.7541080
C	2.8072450	-0.3852760	-3.7403290
C	2.2469090	-0.3026120	-5.1679800
C	2.0255000	-0.5066240	-1.5232370
C	1.8173490	-1.9234120	-2.0568780
C	2.7220170	-1.8839310	-3.3021480
C	2.0808890	-2.6588340	-4.4612160
C	0.6238070	-2.9925480	-4.0049820
C	0.4353040	-2.4839860	-2.5217590
C	-0.6417770	-2.6007190	-4.8235970
C	2.3967160	-1.7496740	-5.6603780
C	-0.5552410	-1.7125870	-6.0737990
P	1.5316250	-0.0752530	0.2203010
N	2.3017870	-1.3369870	1.0236460
P	3.7132860	-1.4935470	1.7146220
C	4.1599530	-3.2610730	1.7859110
C	3.1084470	-4.1376240	2.4739070
C	3.3222820	-5.6355790	2.2415920
C	4.6492730	-6.1654880	2.7820640
P	-1.5852420	0.4492790	-0.4124060
N	-2.8299310	1.4668310	-0.6635480
P	-4.3981490	1.2063180	-0.4928640
C	-4.8433730	0.8927910	1.2401410
C	-6.3214700	0.6463410	1.5596260
C	-6.5009680	-0.0843070	2.8958450
C	-5.8746250	0.6365330	4.0882240
N	2.5183940	1.2983340	0.4103070
P	2.0550260	2.7976420	0.6034370
C	0.5066830	3.0444770	1.5823000
C	-0.5670880	3.9569580	0.9831980
C	-1.8854900	3.8956380	1.7522650
C	-2.9303700	4.8466970	1.1767920
C	1.8441710	3.6984250	-0.9795410
C	3.1647610	3.8229400	-1.7517090
C	2.9701290	4.1552930	-3.2338040
C	2.2087400	5.4567810	-3.4778640
C	3.3389700	3.7186890	1.5116840
C	3.0751330	5.2177500	1.6710630
C	4.1393570	5.9024810	2.5296810
C	3.8891210	7.3999300	2.6898120
C	5.1230270	-0.6524700	0.9054120

H	0.9583190	-3.1912600	1.5472100
H	1.1700320	-3.4255060	3.2778450
C	0.3952480	-5.1570890	2.2598200
H	1.3589700	-5.5320680	1.8984570
H	-0.3327010	-5.3802780	1.4709020
C	0.0138200	-5.9017720	3.5377980
H	0.0036190	-6.9818990	3.3687200
H	0.7307740	-5.6928680	4.3386980
H	-0.9804360	-5.6181330	3.8965900
C	-2.4588480	-0.5311660	2.9409950
H	-2.4567760	0.4228770	3.4794240
H	-2.6070970	-0.3204470	1.8766970
C	-3.5886700	-1.4307070	3.4473980
H	-3.4664390	-1.6448300	4.5159490
H	-3.5600680	-2.3959720	2.9287220
C	-4.9541670	-0.7852220	3.2045120
H	-5.0111900	0.1606070	3.7566190
H	-5.0407320	-0.5233090	2.1410880
C	-6.1100510	-1.6971380	3.6056620
H	-7.0753570	-1.2196880	3.4170840
H	-6.0814260	-2.6346140	3.0396050
H	-6.0605640	-1.9490480	4.6699310
C	-0.2162710	-1.0760640	4.7780610
H	0.7148110	-1.6505730	4.8489440
H	-0.9559390	-1.5771670	5.4111770
C	0.0225520	0.3742980	5.2166930
H	0.5917620	0.8919360	4.4363690
H	-0.9388040	0.8952370	5.3072020
C	0.7840280	0.4834700	6.5403330
H	0.9720070	1.5443800	6.7394570
H	1.7676710	0.0111910	6.4246600
C	0.0507100	-0.1354060	7.7288050
H	0.6009220	0.0335320	8.6584830
H	-0.0706540	-1.2166750	7.6102670
H	-0.9465980	0.3028400	7.8432740
N	2.0244180	0.1948880	0.2030500
P	3.3929710	-0.0163660	1.0279800
C	3.6763470	-1.7196170	1.5941930
H	2.8937800	-1.9331380	2.3308680
H	4.6307710	-1.7102520	2.1313450
C	3.6796660	-2.7665600	0.4802430
H	4.3806850	-2.4702340	-0.3107520
H	2.6860800	-2.7947860	0.0204210
C	4.0649580	-4.1633680	0.9746900
H	3.9055810	-4.8737700	0.1560970
H	3.3856530	-4.4610970	1.7830090
C	5.5133170	-4.2712600	1.4489450
H	5.7580730	-5.3030520	1.7146210
H	6.2045820	-3.9538800	0.6606460
H	5.7046250	-3.6535730	2.3317150
C	4.7333170	0.4455580	-0.1020070
H	4.5490290	-0.0806660	-1.0450060
H	4.5915250	1.5123830	-0.3101860
C	6.1525080	0.1713590	0.3969690
H	6.2901300	-0.9044660	0.5606010
H	6.3153450	0.6603620	1.3656020
C	7.1993180	0.6666600	-0.6018990
H	7.0214380	0.1870120	-1.5724090
H	7.0630720	1.7437150	-0.7583610
C	8.6264320	0.3859050	-0.1395220
H	8.7907810	-0.6882440	-0.0051900
H	9.3559140	0.7489820	-0.8685030
H	8.8327280	0.8779160	0.8166110
C	3.5548120	0.9783110	2.5490460
H	2.6336360	0.7870400	3.1143300
H	4.3676060	0.5457000	3.1406480
C	3.7809780	2.4806870	2.3283600
H	4.8412720	2.6549610	2.1125300
H	3.2312090	2.8283090	1.4472420
C	3.3566370	3.3216030	3.5363850
H	2.2719040	3.2233840	3.6803470
H	3.5407220	4.3764110	3.3049730
C	4.0734370	2.9459730	4.8316310
H	5.1600240	3.0091200	4.7102860
H	3.7848700	3.6176570	5.6444270
H	3.8293140	1.9259880	5.1453320
P	-0.8672580	0.0060870	-0.7971970
N	-0.1933850	-0.3470290	-2.1908130
P	1.1210470	-0.3591940	-3.0845670
C	2.0810270	1.1815780	-2.9803950
H	2.2235500	1.3545260	-1.9075360
H	1.4260620	1.9718190	-3.3598010
C	3.4262130	1.1874460	-3.7125320
H	4.0429360	0.3496570	-3.3669670
H	3.2712960	1.0417610	-4.7886880
C	4.2044310	2.4885640	-3.4859100
H	5.1740240	2.3970790	-3.9868870
C	5.5404670	-1.2938160	-0.4265680
C	6.1352810	-0.2723240	-1.3962480
C	6.6112140	-0.8983970	-2.7040230
C	3.7495980	-0.9284240	3.4572070
C	3.4656230	0.5671390	3.6162080
C	3.3164810	1.0148710	5.0714860
C	4.5893450	0.8643730	5.9024830
N	-1.8925370	-0.6348540	0.7142170
P	-1.4785770	-1.5017410	1.9721380
C	-0.4494670	-2.9556800	1.6415560
C	-1.0575760	-3.9656890	0.6727950
C	-0.0601820	-5.0579080	0.2856590
C	-0.6111910	-5.9907710	-0.7893400
C	-3.0390640	-2.1096110	2.6874660
C	-2.9278310	-3.1068150	3.8411620
C	-4.3006070	-3.4702650	4.4183820
C	-5.2297960	-4.1538940	3.4153710
C	-0.5891880	-0.5999950	3.2783590
C	-1.3374960	0.6277870	3.8003500
C	-0.5014060	1.4583850	4.7781750
C	-0.2718590	0.7680070	6.1211610
C	-5.2831360	2.6968120	-1.0236140
C	-4.9199000	3.1526550	-2.4403470
C	-5.6022060	4.4649000	-2.8398280
C	-5.1502390	5.6710000	-2.0168200
C	-5.0763080	-0.1901080	-1.4680200
C	-4.7276210	-1.5736680	-0.9034120
C	-5.0625220	-2.7151320	-1.8607930
C	-4.6179930	-4.0664460	-1.3050190
H	2.2149900	-2.6232730	-1.3164940
H	2.4279490	1.3481770	-2.4882510
H	2.5938900	-3.6131620	-4.6146100
H	2.8771070	0.3398740	-5.7905340
H	3.1111770	-0.4766130	-1.3457180
H	1.8993230	-1.9895240	-6.5955360
H	3.4682390	-1.8881010	-5.8585280
H	0.1749930	-2.0118390	-6.8192730
H	-1.5304560	-1.7982840	-6.5715480
H	-2.4017340	-0.3721340	-2.2330690
H	-0.8560040	0.3920220	-6.3687820
H	-1.3712470	1.5064670	-3.1416040
H	-2.4295120	0.0853360	-4.6581780
H	-2.5627980	-2.0971430	-3.9070260
H	-1.1538390	-3.5285700	-5.0954730
H	-1.6051280	-2.4419590	-1.8338800
H	3.7199820	-2.2674930	-3.0679620
H	3.8444650	-0.0384180	-3.6936750
H	0.6882290	1.9256990	-3.5574660
H	0.9520130	1.3070000	-5.6383910
H	0.5976130	-4.0847570	-3.9587640
H	0.2983440	-3.3781600	-1.9097540
H	1.4021340	4.6829190	-0.7983100
H	1.1134910	3.1212820	-1.5612130
H	3.7826250	4.6014680	-1.2867170
H	3.7283800	2.8864470	-1.6659870
H	2.4432400	3.3253600	-3.7225580
H	3.9561520	4.2139070	-3.7083850
H	2.6973540	6.2963520	-2.9711010
H	2.1612390	5.6895020	-4.5453720
H	1.1807480	5.3960720	-3.1068850
H	3.4153640	3.2361870	2.4928290
H	4.2898730	3.5410980	0.9975330
H	2.0899660	5.3815970	2.1271200
H	3.0437520	5.7009280	0.6865760
H	4.1665850	5.4231710	3.5161420
H	5.1251370	5.7372250	2.0777330
H	3.8862810	7.9030660	1.7171260
H	2.9199130	7.5867250	3.1642540
H	4.6608990	7.8678130	3.3074400
H	0.8016180	3.4047820	2.5748670
H	0.1076580	2.0355160	1.7331750
H	-0.2032040	4.9919270	0.9586050
H	-0.7704740	3.6715210	-0.0559570
H	-1.7105260	4.1175040	2.8126740
H	-2.2696160	2.8705470	1.6955120
H	-3.0964750	4.6300830	0.1160790
H	-2.6077920	5.8900380	1.2572990
H	-3.8890110	4.7501230	1.6962040
H	5.9677910	-0.6031500	1.6014430
H	4.7572590	0.3670290	0.7383220
H	4.6758830	-1.7671410	-0.9097620
H	6.2629350	-2.0975990	-0.2414130
H	6.9681120	0.2496210	-0.9086360
H	5.3732340	0.4905640	-1.6059790
H	7.3900370	-1.6463900	-2.5218010
H	5.7857900	-1.3980260	-3.2227550
H	7.0215150	-0.1432640	-3.3806830

H	4.4190670	2.5961180	-2.4153420
C	3.4881030	3.7397940	-3.9913890
H	3.2074830	3.6324310	-5.0446290
H	2.5764840	3.9476420	-3.4218110
H	4.1331280	4.6179260	-3.9024670
C	0.5668570	-0.5635750	-4.8027180
H	1.4402070	-0.5347460	-5.4631410
H	0.1394500	-1.5703710	-4.8762310
C	-0.4729380	0.4868310	-5.2051410
H	-0.0159860	1.4845370	-5.2039600
H	-1.2710830	0.5047920	-4.4534230
C	-1.0815700	0.2148270	-6.5802270
H	-0.2884830	0.2140020	-7.3375410
H	-1.5188500	-0.7911400	-6.5857400
C	-2.1501100	1.2421370	-6.9465300
H	-2.5804340	1.0340210	-7.9299070
H	-2.9646850	1.2357190	-6.2141120
H	-1.7309380	2.2534630	-6.9698960
C	2.2263350	-1.7441810	-2.6572460
H	1.5913150	-2.4676020	-2.1364180
H	2.9174210	-1.3524360	-1.9062970
C	2.9779450	-2.4281830	-3.8012270
H	2.2607660	-2.8980810	-4.4846620
H	3.5379110	-1.6949540	-4.3934080
C	3.9428880	-3.4897850	-3.2707850
H	3.3912000	-4.1895380	-2.6302170
H	4.6903880	-3.0056130	-2.6296660
C	4.6427290	-4.2578570	-4.3881720
H	5.3333420	-5.0020330	-3.9824190
H	5.2158890	-3.5810960	-5.0302440
H	3.9156110	-4.7809160	-5.0177860
N	-1.8857730	-1.1158650	-0.2382020
P	-2.7752870	-2.1008320	-1.1535840
C	-3.7746830	-1.2579100	-2.4221170
H	-4.1823570	-2.0167020	-3.0987440
H	-3.0611540	-0.6710510	-3.0145850
C	-4.8881930	-0.3731860	-1.8524140
H	-4.5368930	0.1504040	-0.9553680
H	-5.7245420	-1.0035340	-1.5283100
C	-5.3848580	0.6585740	-2.8650030
H	-5.6717740	0.1489470	-3.7924920
H	-4.5551430	1.3295070	-3.1267290
C	-6.5625090	1.4716420	-2.3349470
H	-7.4241280	0.8263980	-2.1360470
H	-6.3037440	1.9750710	-1.3969130
H	-6.8714180	2.2362670	-3.0525460
C	-1.7948670	-3.3178310	-2.0834870
H	-2.4887780	-4.0267890	-2.5484350
H	-1.3186890	-2.7508410	-2.8909620
C	-0.7390770	-4.0394410	-1.2485910
H	-1.2245880	-4.6467870	-0.4749170
H	-0.1253420	-3.2963010	-0.7226400
C	0.1536590	-4.9345520	-2.1086050
H	-0.4657870	-5.6812760	-2.6186440
H	0.6125850	-4.3296550	-2.9019840
C	1.2398740	-5.6296480	-1.2944410
H	1.8421640	-4.8979180	-0.7448830
H	0.8061640	-6.3138500	-0.5590380
H	1.9128560	-6.2053110	-1.9360140
C	-3.9111310	-2.9568770	-0.0290310
H	-3.2992390	-3.4787800	0.7140610
H	-4.4474410	-2.1639180	0.5058480
C	-4.8916210	-3.9301770	-0.6864770
H	-5.4454030	-3.4303740	-1.4904870
H	-4.3411650	-4.7572220	-1.1500660
C	-5.8867930	-4.4902210	0.3310280
H	-6.4505710	-3.6582490	0.7709880
H	-5.3340210	-4.9612010	1.1532630
C	-6.8521020	-5.4970290	-0.2884880
H	-7.4312230	-5.0384110	-1.0966230
H	-6.3114510	-6.3517960	-0.7077670
H	-7.5567620	-5.8771360	0.4560390

H	2.9839100	-1.5114640	3.9840670
H	4.7208720	-1.2027290	3.8848250
H	2.5522090	0.8114300	3.0633870
H	4.2675240	1.1432770	3.1375960
H	3.0015440	2.0659620	5.0796340
H	2.5018380	0.4482160	5.5389940
H	4.4484960	1.2590290	6.9127900
H	4.8874960	-0.1844100	5.9972580
H	5.4218400	1.4075100	5.4418650
H	4.2867500	-3.5808540	0.7444480
H	5.1388970	-3.3459070	2.2712730
H	3.1061330	-3.9334960	3.5521320
H	2.1202150	-3.8606810	2.0966290
H	3.2529150	-5.8413640	1.1655260
H	2.4949940	-6.1798280	2.7122610
H	4.7225330	-7.2490640	2.6525590
H	5.5028650	-5.7134340	2.2672780
H	4.7504290	-5.9467250	3.8508320
H	-3.5936120	-2.5355950	1.8442500
H	-3.5978610	-1.2264620	3.0162840
H	-2.4270020	-4.0208580	3.4995890
H	-2.3065290	-2.6844010	4.6393540
H	-4.7790600	-2.5607360	4.8042480
H	-4.1498250	-4.1302820	5.2794200
H	-4.7483470	-5.0330560	2.9729360
H	-6.1522920	-4.4835280	3.9008960
H	-5.5142120	-3.4840200	2.5971050
H	-0.3817960	-1.3169780	4.0805140
H	0.3769950	-0.3230680	2.8391730
H	-2.2661220	0.3168780	4.2967550
H	-1.6356350	1.2519330	2.9519830
H	0.4653080	1.6923070	4.3141090
H	-1.0054530	2.4170960	4.9446480
H	0.3262710	1.3956510	6.7877570
H	-1.2246470	0.5571990	6.6183270
H	0.2584840	-0.1824710	6.0027040
H	-0.2422200	-3.4218400	2.6133090
H	0.5072520	-2.5627850	1.2707320
H	-1.9567280	-4.4259190	1.1033280
H	-1.3832460	-3.4423340	-0.2316070
H	0.2164460	-5.6325900	1.1786170
H	0.8643520	-4.5846030	-0.0709960
H	-1.5315420	-6.4791170	-0.4518710
H	0.1093820	-6.7711020	-1.0493880
H	-0.8478540	-5.4337050	-1.7035490
H	-5.0331580	3.4656410	-0.2854950
H	-6.3582460	2.5015460	-0.9365140
H	-5.1986910	2.3704650	-3.1559390
H	-3.8322590	3.2702770	-2.5083510
H	-6.6900870	4.3482410	-2.7597770
H	-5.3886140	4.6530410	-3.8978500
H	-5.4242170	5.5735180	-0.9615380
H	-4.0626410	5.7922860	-2.0685730
H	-5.6086680	6.5913910	-2.3891770
H	-6.1623370	-0.0646880	-1.5450610
H	-4.6764130	-0.0755060	-2.4831420
H	-5.2642980	-1.7275770	0.0419000
H	-3.6631090	-1.6227630	-0.6511740
H	-6.1404090	-2.7255860	-2.0625420
H	-4.5681540	-2.5336300	-2.8236020
H	-4.8827230	-4.8843980	-1.9806070
H	-3.5321800	-4.0894560	-1.1596440
H	-5.0856600	-4.2652290	-0.3342960
H	-4.2352090	0.0245040	1.5070200
H	-4.4418320	1.7365600	1.8112760
H	-6.8574410	1.6021560	1.5814130
H	-6.7897770	0.0442490	0.7726260
H	-6.0663520	-1.0890510	2.8082350
H	-7.5716060	-0.2277630	3.0767130
H	-6.2740700	1.6514430	4.1881400
H	-6.0781150	0.0998900	5.0190430
H	-4.7868750	0.7190860	3.9875430
H	-0.5438740	1.3319950	-0.1501870

III(Pe_2)$_2$ (dication)

C	0.5667870	1.4168800	3.5579450
C	1.4689620	0.2133280	3.1441860
C	1.7601840	-0.5854370	4.4338000
C	1.2491610	0.1895050	5.6621000
C	0.4185400	1.3946160	5.1224110
C	0.9380010	-1.9102260	4.3530150
C	0.1756110	-1.8932540	3.0118650
C	1.0465850	-0.9307720	2.2005240
C	-0.0611780	-1.8912600	5.5237640
C	-1.4693940	-1.6193120	4.9024690
C	-1.3122580	-1.6019280	3.3402650
C	0.6111150	-0.9216270	6.5069220

III(Pe_2)$_2$ (protonated)

C	0.2586270	-2.8476190	-2.1680950
C	1.6205770	-2.0718770	-2.1193110
C	2.2120690	-2.1223010	-3.5450110
C	1.4342280	-3.1170570	-4.4162610
C	0.1751450	-3.5287950	-3.5918670
C	1.9999330	-0.7011650	-4.1697430
C	1.2867820	0.1559900	-3.0997930
C	1.7951490	-0.5710350	-1.8449640
C	1.1029600	-0.8814070	-5.4014230
C	-0.2990970	-0.3207840	-5.0069510
C	-0.2067930	0.2926170	-3.5531540
C	1.3317330	-2.3606450	-5.7498330

C	-2.3946420	-0.4281300	5.3008570
C	-2.6626940	0.3754980	4.0143360
C	-2.1222880	-0.3931990	2.7862250
C	-1.8413070	1.6990190	4.1285520
C	-1.0852800	1.6514980	5.4677350
C	-1.9330170	0.6604820	6.2793220
C	-0.8341280	1.7104660	2.9633880
C	-1.5252500	0.7722560	1.9756960
H	2.3858820	0.6468330	2.7400000
H	0.2258350	-2.8720430	2.5309200
H	2.0912880	0.6068530	6.2211310
H	-0.1087160	-2.8769080	5.9950330
H	1.9821310	-1.5038490	2.1030320
H	0.0022000	-0.5904080	7.3427370
H	1.4443210	-1.4783010	6.9550260
H	-1.4972730	0.3064980	7.2088370
H	-2.8374390	1.2097000	6.5714050
H	-2.4213760	1.3523350	1.7283580
H	-3.3261850	-0.8588010	5.6785190
H	-2.9453460	-0.8153330	2.2052080
H	-3.7267100	0.6011020	3.8959020
H	-2.5095530	2.5628480	4.0744170
H	-1.1329180	2.6257400	5.9621480
H	-0.8077030	2.6976320	2.5005540
H	2.8264920	-0.8126880	4.5240590
H	1.6081320	-2.7725170	4.4120950
H	-1.8540260	-2.4714980	2.9656800
H	-2.0497580	-2.5124300	5.1455470
H	0.9385190	2.2794800	5.4968840
H	1.1678140	2.2954290	3.3063710
P	0.8350590	-0.6944610	0.4070230
N	2.1092570	0.1766180	-0.0721080
N	0.6792510	-2.1535380	-0.2189730
P	-0.0851480	-3.2229510	-1.1152290
C	-1.6048360	-3.8305390	-0.3036360
C	-2.4757940	-4.7711540	-1.1777280
C	-1.4172350	-4.5806260	1.0492350
C	-2.0404230	-5.9682790	0.8452970
H	-3.5207180	-6.6265480	-0.6515550
C	-3.1478510	-5.7157930	-0.1773730
H	-3.9973170	-5.2179750	0.3044170
H	-2.4019840	-6.3993570	1.7815890
H	-1.3056220	-6.6635380	0.4223950
H	-1.9760560	-4.0473720	1.8220100
H	-0.3825890	-4.6284300	1.3887970
H	-1.8538460	-5.3678670	-1.8533440
H	-3.1878340	-4.2258280	-1.8018640
C	-0.9649820	-3.4750830	-3.8785370
C	-1.0421830	-2.5360740	-5.0941640
H	-2.0603260	-2.1487960	-5.1940720
H	-1.8923110	-4.0290170	-3.7300020
H	-0.1610590	-4.2051270	-4.0191050
C	-0.6289380	-2.5219560	-2.7177580
C	-0.0597400	-1.3774140	-4.8022440
C	0.3967460	-1.5574520	-3.3392970
H	2.8662250	-7.1207990	-0.5032610
H	0.4640240	-0.6204680	-2.7804240
H	3.7139000	-5.6499360	-0.0571300
H	-0.5501010	-0.4117600	-4.9451460
C	2.2317900	-4.3986740	-2.3200550
C	0.9904640	-4.6664850	-1.4507110
C	1.5531630	-5.3829550	-0.1970810
H	1.7357380	-4.6668870	0.6091220
H	1.3913910	-2.0091410	-3.3239870
H	0.8460370	-6.1229420	0.1806670
H	4.1064650	-5.5268480	-2.4142950
H	1.9932180	-4.1967050	-3.3670900
H	2.6534290	-6.4631700	-2.7743850
H	2.7855140	-3.5427360	-1.9261840
C	5.0545310	-1.6345620	-2.4966160
C	4.7951780	-1.8963030	-3.9872780
C	3.7734510	-0.9034160	-2.0658550
P	3.6485280	-0.1941350	-0.3855190
H	5.7222220	-2.0434970	-4.5454990
H	4.2042950	-2.8123260	-4.0936600
H	5.9281300	-0.9873730	-2.3693060
C	4.5717550	1.3717130	-0.3190310
H	4.2885140	1.3597090	1.8934130
C	4.6404220	2.0171030	1.0989760
H	3.9983860	2.8940760	1.1294570
H	6.3505400	3.4587440	-0.5908230
C	6.1124840	2.4223860	1.2992750
H	6.2084870	3.3496260	1.8694680
H	6.6496970	1.6418770	1.8499250
C	6.6765860	2.5237830	-0.1195270
H	7.7680220	2.4921800	-0.1543030
C	6.0292210	1.3321380	-0.8299170

C	-1.6152150	-1.1513200	-5.1099600
C	-2.2473880	-1.1247540	-3.7127720
C	-1.5141070	-0.1266080	-2.7900370
C	-2.0370890	-2.5438560	-3.0851290
C	-1.2845380	-3.3859580	-4.1235810
C	-1.6166980	-2.6535080	-5.4333010
C	-1.1851520	-2.3387190	-1.8121790
C	-1.6159280	-0.9009170	-1.4630280
H	2.2634240	-2.6184000	-1.4255010
H	1.7051450	1.1653290	-3.0798630
H	2.0246860	-4.0234790	-4.5810190
H	1.4714250	-0.2740660	-6.2336430
H	2.8865280	-0.4562890	-1.9858010
H	0.6646660	-2.7873780	-6.4924500
H	2.3335660	-2.4092640	-6.1974350
H	-1.0538390	-2.9571640	-6.3110500
H	-2.6615540	-2.9074120	-5.6576620
H	-2.7063640	-1.0507140	-1.4086000
H	-2.2616660	-0.6444870	-5.8333030
H	-2.1022680	0.7853890	-2.6817410
H	-3.3171740	-0.8936790	-3.7620800
H	-3.0047710	-2.9865980	-2.8289200
H	-1.7072780	-4.3938110	-4.1797660
H	-1.5517250	-2.9833820	-1.0105390
H	3.2802140	-2.3617790	-3.5110820
H	2.9666280	-0.2607220	-4.4321810
H	-0.3273520	1.3724430	-3.6720590
H	-0.4548870	0.5233040	-5.6841110
H	0.3001910	-4.6016460	-3.4227800
H	0.4185680	-3.6605180	-1.4552690
P	1.8464610	0.1602950	-0.1837840
N	2.8937570	-0.6762970	0.7139540
N	2.2045830	1.7099540	-0.4726290
P	1.8444470	3.0720510	0.2752820
C	2.1646120	2.9641940	2.0712290
C	1.9155880	4.1941020	2.9556420
C	1.3791120	1.8308600	2.7751970
C	1.2967760	2.2606800	4.2634620
H	1.6615330	4.2524200	5.1406290
C	2.0548190	3.5974290	4.3593480
H	3.1149660	3.4205360	4.5751380
H	1.7153800	1.5022340	4.9311630
H	0.2515200	2.4018410	4.5532990
H	1.8785480	0.8679510	2.6404620
H	0.3749820	1.7235340	2.3512380
H	0.8926720	4.5621620	2.8197190
H	2.6058120	5.0184250	2.7555890
C	3.0845370	4.2930060	-1.9546150
C	4.5532140	4.6790060	-2.2671060
H	4.6207490	5.4736980	-3.0138170
H	2.3704180	4.9631400	-2.4382280
H	2.8747050	3.2800250	-2.3077520
C	2.9508800	4.3572240	-0.4094450
C	5.1724970	5.0925790	-0.9233660
C	4.3980300	4.2672350	0.1053600
H	-2.0352320	4.7055490	-1.7196540
H	4.5108230	4.6308160	1.1300500
H	-0.8358070	4.8528150	-3.0022370
H	5.0019690	6.1588310	-0.7366210
C	-0.1515670	5.1169950	0.2994510
C	0.1108890	3.6119670	0.0149380
C	-0.3380820	3.3707900	-1.4562120
H	0.5100920	3.1001560	-2.0918290
H	4.7406240	3.2271350	0.0730570
H	-1.0418420	2.5351200	-1.4875980
H	0.7142660	6.0024550	-1.4830900
H	-1.0961700	5.2098980	0.8424800
H	-0.8390270	6.7095890	-1.0433170
H	0.6107660	5.5972780	0.9175760
C	4.2528240	-3.7086350	0.2537860
C	4.9106270	-4.7514690	-0.6575130
C	4.9112190	-2.4037140	-0.2181910
P	4.4667190	-0.9215330	0.7512600
H	4.8649820	-5.7578960	-0.2350920
H	4.3806400	-4.7747650	-1.6158420
H	4.5164520	-3.9005870	1.3004420
C	5.4658160	0.4828750	0.1395270
H	7.3242210	-0.4972610	0.8996050
C	6.9874970	0.4539360	0.4811120
H	7.1982960	1.2182180	1.2334850
H	6.5722940	2.5468900	-1.3379730
C	7.7135050	0.7778680	-0.8392890
H	8.6218300	1.3632410	-0.6776620
H	8.0079370	-0.1476620	-1.3466580
C	6.6665950	1.5097610	-1.6818360
H	6.9065530	1.5326660	-2.7479110
C	5.3747380	0.7442010	-1.3813390

H	6.0911650	1.3898430	-1.9192450
H	6.5366320	0.4127450	-0.5163100
C	3.9840530	-0.6770910	-4.4878240
C	4.5417650	-1.4236670	0.6521750
H	4.5891710	-0.0235350	-5.1200560
H	4.1074740	0.9906090	-3.0900530
C	3.5180470	0.0774640	-3.2205650
H	3.1291110	-0.9987270	-5.0891760
C	3.9243560	-2.8516750	0.5918040
H	2.8962940	-2.8418970	0.2263200
H	4.5041910	-3.4735700	-0.0945980
H	4.2599550	-4.4653340	2.0501640
C	3.9933950	-3.4057950	2.0296020
H	3.0156550	-3.3150410	2.5175370
C	5.0173540	-2.5209490	2.7462110
H	4.9052420	-2.5311320	3.8334900
C	4.7774030	-1.1316260	2.1481720
H	5.6155370	-0.4503720	2.3087710
H	3.8959290	-0.6851250	2.6127740
H	2.4709310	0.3694390	-3.2725220
H	6.0342800	-2.8538720	2.5103200
H	-0.8065410	-3.0603590	-6.0228360
H	0.7986840	-1.3940630	-5.4785590
C	2.8824930	-6.0387980	-0.6507380
C	3.0596090	-5.6719480	-2.1354470
H	5.2355740	-2.5571650	-1.9405050
H	5.5266010	-1.4587500	0.1706360
H	3.9911170	2.0266700	-0.9794260
H	2.9533920	-1.6302610	-2.0555040
H	0.3474770	-5.3697610	-1.9951820
H	-2.1271410	-2.8871220	-0.1151550
H	-1.5234850	-1.9473610	-2.4507170
P	-0.9601820	0.6348330	0.2520340
N	-2.0939850	-0.1862860	-0.5535550
N	-0.6583540	2.1176110	-0.2317570
P	0.2393020	3.2510050	-0.9003460
C	-0.8739200	4.6396810	-1.3400450
C	-1.8767870	5.0628080	-0.2208390
C	-0.2190350	5.9516370	-1.8313900
C	-1.2965090	7.0033420	-1.5521390
H	-1.1014430	6.9298480	0.5968560
C	-1.8127120	6.5998760	-0.1689320
H	-2.7815920	7.0414850	0.0756380
H	-2.0989330	6.9220220	-2.2946870
H	-0.9056340	8.0230310	-1.5883510
H	0.0761090	5.9004450	-2.8822170
H	0.6676380	6.2011900	-1.2424310
H	-1.6706600	4.5978130	0.7461290
H	-2.8798590	4.7455120	-0.5130540
C	1.0674940	4.6314180	1.4558490
C	2.3817890	5.0357930	2.1249070
H	2.7832270	4.1812890	2.6826840
H	0.3954640	4.0897330	2.1210540
H	0.5441460	5.5276530	1.1063610
C	1.5531120	3.7973780	0.2559990
C	3.3035510	5.3960760	0.9459070
C	2.7479960	4.6259090	-0.2827810
H	2.5538800	3.2735450	-5.3362760
H	3.5021160	3.9948090	-0.7556410
H	1.0888930	4.2144320	-5.0611580
H	4.3476790	5.1432680	1.1482730
C	-0.0160490	2.1631390	-3.4776640
C	1.0211290	2.6229810	-2.4316040
C	2.0111540	3.5301940	-3.2162080
H	1.9389740	4.5816190	-2.9379970
H	2.4316740	5.3384160	-1.0435560
H	3.0392910	3.2231180	-3.0072150
H	0.1317460	2.0849620	-5.6621000
H	-0.5027930	1.2275990	-3.1968390
H	1.3925380	1.1921400	-4.8153000
H	-0.8008850	2.9161040	-3.5974730
C	-5.6902110	-1.8095080	-1.7893930
C	-6.2082920	-3.1721900	-1.3238010
C	-4.4287930	-1.6260220	-0.9199850
P	-3.6658010	0.0377820	-0.8479110
H	-7.2343470	-3.3655720	-1.6450030
H	-5.5753550	-3.9682830	-1.7339030
H	-6.4417700	-1.0432310	-1.5782190
C	-4.6214360	1.0156580	0.3862020
H	-3.3296300	2.7860800	-0.0127900
C	-4.1792920	2.4959210	0.6048620
H	-3.8630610	2.6246710	1.6440700
H	-6.6551030	2.2890440	1.8108610
C	-5.4351180	3.3479000	0.3675600
H	-5.4249570	4.2630920	0.9639450
H	-5.5172760	3.6431560	-0.6846090
C	-6.5830240	2.4015790	0.7230510

H	4.4661210	1.2960370	-1.6360200
H	5.3747050	-0.1944970	-1.9493860
C	6.3630150	-4.2525970	-0.8609720
C	4.9230950	-1.1572270	2.5051930
H	7.0792950	-4.8699010	-0.3133830
H	6.8706440	-2.7623800	0.6478860
C	6.3937480	-2.7938800	-0.3378290
H	6.6452050	-4.2938750	-1.9158900
C	3.9085960	-2.0530840	3.2779070
H	3.0675740	-2.3514880	2.6488560
H	4.4090420	-2.9625760	3.6210780
H	3.2202930	-1.8092180	5.3570380
C	3.4233590	-1.2044040	4.4694400
H	2.4919180	-0.6912630	4.2004930
C	4.5288600	-0.1702400	4.6881680
H	4.1972960	0.6996720	5.2624180
C	4.9441270	0.1943470	3.2622910
H	5.9155890	0.6896930	3.2013760
H	4.1937720	0.8745960	2.8422910
H	6.9518920	-2.1232970	-0.9947740
H	5.3725950	-0.6239660	5.2207580
H	5.0845110	3.8114810	-2.6710100
H	6.2514040	4.9186290	-0.8793530
C	-0.9607900	4.6965470	-1.9272090
C	-0.2780970	5.7720070	-1.0799480
H	3.1635170	-3.6926570	0.1807270
H	5.9207540	-1.6067710	2.5363680
H	5.0064220	1.3349870	0.6516850
H	4.5240170	-2.1812780	-1.2233800
H	-0.4920590	2.9808140	0.6784620
H	3.2366190	2.7279560	2.1219270
H	2.5449100	5.3305380	-0.1128080
P	-1.5834660	-0.1272760	0.2630970
N	-2.7003320	1.0997800	0.0549120
N	-2.3594940	-1.4249610	1.0403020
P	-1.9383400	-2.0879140	2.4164060
C	-2.8198760	-3.6876360	2.5685730
C	-4.3697440	-3.5313720	2.5068330
C	-2.4941660	-4.7080280	1.4583740
C	-3.7017640	-5.6443230	1.4779920
H	-5.1089380	-4.2729450	0.6008720
C	-4.8773890	-4.6715650	1.5943810
H	-5.7878950	-5.1386570	1.9786000
H	-3.6521280	-6.2965180	2.3583730
H	-3.7594690	-6.2822130	0.5917100
H	-1.5453950	-5.2256860	1.6105780
H	-2.4487650	-4.1918370	0.4925820
H	-4.6547000	-2.5586450	2.0975190
H	-4.7953810	-3.6029480	3.5107790
C	0.5046240	-3.2054550	1.4740020
C	1.1167760	-4.4754410	2.1079490
H	2.2000250	-4.3585710	2.2118740
H	1.2678890	-2.6040990	0.9726790
H	-0.2397410	-3.4577780	0.7167430
C	-0.1270370	-2.3905660	2.6366720
C	0.4901250	-4.5955160	3.5025820
C	0.2633280	-3.1433990	3.9319890
H	-3.7830760	0.0553230	6.0938850
H	1.1987230	-2.7237650	4.3139460
H	-4.5947190	1.1681340	4.9939990
H	1.1278160	-5.1397280	4.2045410
C	-1.5534790	0.1932490	3.9892400
C	-2.3587840	-1.1220330	3.9321590
C	-3.8159270	-0.6561860	4.0545680
H	-4.1555750	-0.2723560	3.0880650
H	-0.4695730	-3.0558190	4.7385310
H	-4.4982970	-1.4542710	4.3537200
H	-2.5181100	2.1224480	4.3171670
H	-0.5471200	0.0374110	4.3870150
H	-1.8933010	1.4030330	5.7922490
H	-1.4423960	0.5939080	2.9747070
C	-3.9845340	2.7939130	-2.6308500
C	-4.6106680	2.8126820	-4.0307490
C	-4.6572430	1.5779420	-1.9804950
P	-4.2476820	1.2874820	-0.2125090
H	-4.5610820	3.8004780	-4.4957790
H	-4.0615150	2.1200610	-4.6781070
H	-4.2552190	3.7045660	-2.0834000
C	-5.3569380	-0.0759320	0.3393870
H	-7.1408280	1.2562430	0.1542740
C	-6.8605350	0.2791970	0.5544140
H	-7.0726830	0.3094190	1.6267820
H	-6.6024680	-2.5106250	0.7699910
C	-7.6608270	-0.8513260	-0.1232100
H	-8.5744790	-1.0980740	0.4240430
H	-7.9603310	-0.5506830	-1.1335680
C	-6.6762340	-2.0196770	-0.2077480

H	-7.5540100	2.7384190	0.3527410
C	-6.1429580	1.0800310	0.0871400
H	-6.6763890	0.2093100	0.4760590
H	-6.3355090	1.1338040	-0.9893460
C	-6.0611010	-3.1044820	0.1999210
C	-3.9557820	0.7737870	-2.4965450
H	-6.9278020	-2.5914350	0.6283840
H	-4.9717530	-1.5229030	1.2286060
C	-4.7836710	-2.2675470	0.4525990
H	-6.0085080	-4.0915450	0.6655440
C	-3.1758720	0.0578530	-3.6118690
H	-2.1555250	-0.1201610	-3.2609020
H	-3.6062170	-0.9111200	-3.8768980
H	-3.8960450	0.7672500	-5.5476080
C	-3.1643130	1.0551800	-4.7893200
H	-2.1868740	1.0654230	-5.2797900
C	-3.5160270	2.4360030	-4.1836310
H	-2.8199280	3.2209030	-4.4917590
C	-3.5253630	2.2372670	-2.6607100
H	-4.1909330	2.9406430	-2.1585350
H	-2.5231320	2.3580430	-2.2375030
H	-3.9597040	-2.8850320	0.8160290
H	-4.5114650	2.7484930	-4.5120760
H	2.2569770	5.8589350	2.8319110
H	3.2683330	6.4715740	0.7536870
C	1.6611490	3.3488450	-4.7114660
C	0.7793200	2.0981170	-4.7812440
H	-5.4679790	-1.7761910	-2.8583140
H	-5.0306800	0.6891400	-2.6943130
H	-4.4837610	0.4735980	1.3264970
H	-3.6358530	-2.2167680	-1.3902460
H	1.5519090	1.7412960	-2.0533460
H	-1.4616670	4.2376260	-2.1704910
H	1.9222360	2.8326620	0.6337250

H	-6.9669810	-2.7790100	-0.9391900
C	-5.3460050	-1.3408160	-0.5475510
H	-4.4671940	-1.9526880	-0.3308840
H	-5.3321100	-1.0881210	-1.6151120
C	-6.0650210	2.3171740	-3.8295740
C	-4.7751610	2.7768330	0.7266310
H	-6.7886840	3.1249890	-3.9658990
H	-6.6104070	2.4943430	-1.7265500
C	-6.1297560	1.7620730	-2.3847690
H	-6.3146410	1.5392130	-4.5559750
C	-3.7515800	3.9448670	0.6074870
H	-2.8717700	3.6433170	0.0359810
H	-4.2132110	4.7950500	0.0966100
H	-3.1654850	5.3832660	2.1765210
C	-3.3626660	4.3147650	2.0532620
H	-2.4501720	3.7740480	2.3369460
C	-4.5292830	3.8123850	2.9057340
H	-4.2768000	3.7036380	3.9650620
C	-4.8710970	2.4800630	2.2400060
H	-5.8429540	2.0763460	2.5335270
H	-4.1026430	1.7488120	2.5151280
H	-6.7045680	0.8359890	-2.3184040
H	-5.3791250	4.5013690	2.8321760
H	0.9519590	-5.3650430	1.4926910
H	-0.4641670	-5.1300180	3.4558680
C	-3.7497610	0.4790590	5.0846360
C	-2.3903550	1.1726190	4.8459870
H	-2.8955610	2.7273570	-2.6480570
H	-5.7532920	3.0989180	0.3551690
H	-4.9175420	-0.3645210	1.2983160
H	-4.2720330	0.6799560	-2.4828620
H	-2.1179940	-1.7392650	4.8059370
H	-2.5384530	-4.1142360	3.5392590
H	0.2931060	-1.3777210	2.6445860
H	0.6150670	0.0258010	0.4733700

III(Pf$_2$)$_2$ (dication)

C	-0.6991250	3.1803140	1.6294770
C	-1.7482360	2.7012130	0.5774570
C	-2.2737820	3.9697910	-0.1317920
C	-1.7620260	5.2300310	0.5890410
C	-0.7058210	4.7531380	1.6330330
C	-1.6575620	3.9841320	-1.5653690
C	-0.7850920	2.7222190	-1.6973190
C	-1.4336590	1.8139970	-0.6421590
C	-0.7785820	5.2425350	-1.6689810
C	0.7059980	4.7531220	-1.6330020
C	0.6992430	3.1802980	-1.6294550
C	-1.3826650	6.1455580	-0.5829700
C	1.7622210	5.2299690	-0.5890070
C	2.2739290	3.9697050	0.1318180
C	1.7483360	2.7011520	-0.5774380
C	1.6577090	3.9840610	1.5653960
C	0.7787760	5.2424960	1.6690160
C	1.3828940	6.1455030	0.5830110
C	0.7851930	2.7221790	1.6973380
C	1.4337260	1.8139400	0.6421720
H	-2.5570620	2.2301830	1.1400110
H	-0.9415760	2.2534240	-2.6693630
H	-2.5744700	5.7032570	1.1476330
H	-0.9274830	5.7281330	-2.6374860
H	-2.4297130	1.6527180	-1.0808580
H	-0.8127020	7.0331000	-0.3253290
H	-2.3295620	6.5169870	-0.9959490
H	0.8129660	7.0330690	0.3253760
H	2.3298060	6.5168910	0.9959930
H	2.4297750	1.6526220	1.0808710
H	2.5746820	5.7031680	-1.1475960
H	2.5571440	2.2300960	-1.1399950
H	3.3659070	3.9550790	0.1913920
H	2.4528090	3.9853050	2.3157550
H	0.9276950	5.7280810	2.6375240
H	0.9416590	2.2533720	2.6693790
H	-3.3657610	3.9552060	-0.1913660
H	-2.4526620	3.9854110	-2.3157280
H	1.1275360	2.8670030	-2.5853330
H	1.1154680	5.0585010	-2.5987820
H	-1.1152800	5.0585260	2.5988160
H	-1.1274300	2.8670290	2.5853520
P	0.9547780	0.0523060	0.5632250
N	0.7226160	-0.3158300	2.0966160
P	0.5790160	-1.4271950	3.2268630
C	2.2560910	-1.8053380	3.8954270
C	2.8460720	-0.5780340	4.6048530
H	2.4305700	-0.4653340	5.6095100
H	3.9275970	-0.7026810	4.7065620
H	2.6617630	0.3476620	4.0537590

III(Pf$_2$)$_2$ (protonated)

C	-0.6034630	3.1038030	1.4230050
C	-1.6560300	2.6087860	0.3875660
C	-2.0530450	3.8124810	-0.4797620
C	-1.5471020	5.1121730	0.1560240
C	-0.6007720	4.6788790	1.3262890
C	-1.3181080	3.6384610	-1.8488750
C	-0.4600130	2.3463590	-1.7435270
C	-1.3413890	1.5885410	-0.7078340
C	-0.4309190	4.8804990	-2.0285410
C	1.0348590	4.4164650	-1.8050130
C	1.0331950	2.8479460	-1.6404010
C	-1.0823190	5.8978610	-1.0827760
C	1.9860150	5.0318120	-0.7216700
C	2.4877710	3.8496960	0.1196230
C	2.0758430	2.5255250	-0.5327190
C	1.7508730	3.9065850	1.4956490
C	0.8684320	5.1662130	1.4697730
C	1.5234680	6.0121450	0.3702330
C	0.8870240	2.6209970	1.5945200
C	1.7173640	1.6830800	0.6811350
H	-2.5261550	2.2647290	0.9473650
H	-0.5200010	1.7790410	-2.6764620
H	-2.3782860	5.6606980	0.6092150
H	-0.5058940	5.2587970	-3.0525250
H	-2.2962940	1.5834360	-1.2644260
H	-0.5087150	6.8016950	-0.9007280
H	-2.0031820	6.2321960	-1.5791730
H	0.9559670	6.8785380	0.0427640
H	2.4460970	6.4177720	0.8067760
H	2.6640030	1.6224680	1.2444910
H	2.8166590	5.4971450	-1.2611520
H	2.9393160	2.0782280	-1.0241880
H	3.5713180	3.8919130	0.2690100
H	2.4799050	3.9435140	2.3106040
H	0.9436800	5.7083390	2.4174910
H	0.9490450	2.2028130	2.6022590
H	-3.1349180	3.8381680	-0.6368670
H	-2.0504430	3.5455550	-2.6569600
H	1.4768050	2.4456900	-2.5545490
H	1.5428620	4.6237340	-2.7506680
H	-1.1078510	5.0419060	2.2242310
H	-1.0514430	2.8585660	2.3900870
P	1.5429990	-0.1435350	0.3390610
N	1.2445710	-0.6282400	1.9285830
P	1.7484410	-1.7930950	2.8568280
C	0.8504100	-1.6234680	4.4647750
C	1.3555590	-2.5376920	5.5843040
H	1.4636000	-3.5787500	5.2674500
H	2.3214300	-2.1946350	5.9666290
H	0.6486200	-2.5173370	6.4192590

H	2.8263840	-1.9651990	2.9717420
C	2.3958130	-3.0677670	4.7530030
H	2.0512750	-3.9669740	4.2392220
H	1.8582050	-2.9854950	5.6991230
H	3.4542490	-3.2114230	4.9883360
C	-0.2122620	-2.9423870	2.5486380
H	-0.9860080	-2.4726680	1.9266540
C	0.7426510	-3.6725600	1.6021700
H	0.2109010	-4.4924500	1.1123070
H	1.1129350	-2.9980970	0.8269560
H	1.5988300	-4.1089400	2.1245930
C	-0.8899360	-3.8913030	3.5402130
H	-0.1805450	-4.3490410	4.2339390
H	-1.6726440	-3.3975860	4.1219260
H	-1.3657730	-4.7008700	2.9786690
C	-0.5329400	-0.7879920	4.5537850
C	-0.4295450	0.7295970	4.7200630
C	-0.4028380	-1.4935600	5.9101470
H	-1.5283580	-1.0109980	4.1529930
H	-1.2166400	1.0688750	5.3996090
H	0.5330250	1.0247320	5.1457140
H	-0.5525930	1.2393950	3.7654450
H	-1.2334070	-1.1764470	6.5465990
H	-0.4384090	-2.5815150	5.8412850
N	1.9892540	-0.8025130	-0.3192880
P	3.5517640	-1.0597290	-0.5746050
H	3.5385440	-2.6764950	1.1099750
C	3.7744190	-1.0832520	-2.4014750
C	4.6156330	0.2525060	0.1724920
C	4.0029890	-2.7114780	0.1168440
C	3.3278700	-3.8409520	-0.6732560
C	5.4988420	-2.9923230	0.3049740
C	5.0832570	-1.6679260	-2.9435430
C	3.4825090	0.3072370	-2.9768500
H	5.0424770	-1.6454840	-4.0360760
H	5.9603140	-1.0967920	-2.6359210
H	5.2304100	-2.7075010	-2.6471680
H	4.2434020	1.0368090	-2.6841920
H	5.6117610	-4.0024850	0.7081130
H	5.9688220	-2.3072130	1.0116990
H	6.0534980	-2.9507180	-0.6347960
H	3.4233100	-4.7750810	-0.1136870
H	3.7970890	-3.9898010	-1.6493100
H	4.1020430	1.1694950	-0.1391090
C	6.0568870	0.3551680	-0.3413140
C	4.5594350	0.1774310	1.7033820
H	4.9740810	1.0954910	2.1279450
H	3.5372130	0.0756520	2.0730930
H	5.1423460	-0.6613500	2.0927010
H	6.6224790	-0.5688950	-0.2113480
H	6.5677640	1.1376930	0.2266210
H	6.0976400	0.6410810	-1.3936670
H	3.4806790	0.2600340	-4.0696410
H	2.5050580	0.6713090	-2.6515730
H	2.9625200	-1.7549250	-2.7085580
H	2.2639930	-3.6582500	-0.8288990
H	0.5224630	-1.2115880	6.4182220
P	-0.9547760	0.0523450	-0.5632220
N	-0.7226210	-0.3157850	-2.0966160
P	-0.5790680	-1.4271490	-3.2268710
C	-2.2561580	-1.8052240	-3.8954350
C	-2.8460940	-0.5778920	-4.6048490
H	-2.4305880	-0.4651970	-5.6095050
H	-3.9276240	-0.7024970	-4.7065580
H	-2.6617500	0.3477920	-4.0537470
H	-2.8264540	-1.9650710	-2.9717500
C	-2.3959300	-3.0676400	-4.7530220
H	-1.8583260	-2.9853780	-5.6991450
H	-3.4543740	-3.2112560	-4.9883480
H	-2.0514200	-3.9668640	-4.2392520
C	0.2121550	-2.9423760	-2.5486620
H	0.9859170	-2.4726920	-1.9266720
C	-0.7427850	-3.6725260	-1.6022030
H	-0.2110650	-4.4924420	-1.1123510
H	-1.1130430	-2.9980590	-0.8269800
H	-1.5989800	-4.1088670	-2.1246310
C	0.8897950	-3.8913050	-3.5402480
H	1.3656040	-4.7008960	-2.9787130
H	0.1803880	-4.3490110	-4.2339780
H	1.6725200	-3.3976100	-4.1219560
C	0.5329070	-0.7879780	-4.5537920
C	0.4295690	0.7296170	-4.7200590
C	0.4027710	-1.4935320	-5.9101580
H	1.5283180	-1.0110250	-4.1530060
H	1.2166800	1.0688700	-5.3995980
H	-0.5329870	1.0247900	-5.1457140
H	0.5526300	1.2394030	-3.7654370
H	-0.1677480	-1.9356730	4.2014970
C	0.7665480	-0.1645630	4.9233620
H	1.7305540	0.1964370	5.2925850
H	0.0420280	-0.0783540	5.7397690
H	0.4513780	0.4792140	4.1004420
C	3.5854400	-1.7939070	3.0932340
H	3.9109390	-1.7499200	2.0451580
C	4.0530280	-0.4982050	3.7638720
H	3.8182140	-0.4938880	4.8333730
H	5.1397900	-0.4032710	3.6685260
H	3.5906980	0.3782330	3.3020220
C	4.2107140	-3.0207260	3.7657750
H	5.2995810	-2.9687830	3.6608490
H	3.9882810	-3.0584950	4.8348530
H	3.8847370	-3.9650040	3.3233530
C	1.3390000	-3.5067510	2.2623380
C	2.1671840	-3.8521840	1.0213290
C	-0.1545010	-3.6083580	1.9396790
H	1.5786080	-4.2117650	3.0673210
H	3.2344130	-3.9418530	1.2378870
H	1.8306420	-4.8023770	0.5947830
H	2.0488130	-3.0770430	0.2603620
H	-0.7828680	-3.4722260	2.8235190
H	-0.4343450	-2.8523680	1.1972720
N	3.1735890	-0.4710840	0.0316310
P	4.1398100	-0.7091400	-1.1846260
H	4.4700420	-2.8836520	-0.4290550
C	3.6565940	0.0787220	-2.7992530
C	5.7917920	0.0162260	-0.7734790
C	4.4715850	-2.5145040	-1.4618880
C	3.3062770	-3.1843800	-2.1982540
C	5.8134160	-2.8937870	-2.0995010
C	2.2037570	-0.1922680	-3.2113520
C	4.6211210	-0.1944990	-3.9583180
H	1.9231660	0.5103500	-4.0036740
H	2.0801450	-1.2006300	-3.6145140
H	1.5009210	-0.0713520	-2.3807320
H	4.3625860	0.4520830	-4.8027220
H	5.9173330	-2.4941390	-3.1110260
H	5.8787970	-3.9844770	-2.1697750
H	6.6683210	-2.5526210	-1.5115790
H	2.3372370	-2.8081600	-1.8620840
H	3.3245340	-4.2646430	-2.0235380
H	6.4813400	-0.2603000	-1.5790930
C	6.2996770	-0.5709940	0.5489890
C	5.7245040	1.5430310	-0.6738580
H	4.9692350	1.8413250	0.0612370
H	6.6916910	1.9345060	-0.3439270
H	5.4851580	2.0184320	-1.6290940
H	7.3007180	-0.1865170	0.7666170
H	6.3550430	-1.6634450	0.5379990
H	5.6317370	-0.2774950	1.3629670
H	5.6642420	0.0048420	-3.6976570
H	4.5453980	-1.2286090	-4.3063360
H	3.7219260	1.1467080	-2.5635590
H	3.3765290	-3.0219570	-3.2781500
H	-0.3831830	-4.5931780	1.5200810
P	-1.5734250	-0.1881440	-0.3038090
N	-1.2433180	-1.0962490	-1.5777910
P	-1.9717070	-1.9741230	-2.6771280
C	-0.7682170	-3.2692670	-3.1922420
C	-0.1679000	-4.0320210	-2.0059240
H	0.7189590	-4.5762310	-2.3428310
H	0.1325760	-3.3497130	-1.2073310
H	-0.8646830	-4.7630710	-1.5917090
H	0.0398390	-2.6618650	-3.6193650
C	-1.2952360	-4.2139540	-4.2762090
H	-0.4755660	-4.8397870	-4.6400600
H	-1.7104860	-3.6807500	-5.1360360
H	-2.0660760	-4.8821870	-3.8811450
C	-2.3541670	-0.9904900	-4.1888680
H	-2.8859750	-1.6602750	-4.8736550
C	-3.2699190	0.1864690	-3.8315280
H	-3.5852830	0.6973750	-4.7451050
H	-4.1701650	-0.1214120	-3.2924960
H	-2.7357400	0.9115260	-3.2109930
C	-1.0767840	-0.4926350	-4.8725890
H	-1.3403890	0.1889890	-5.6862930
H	-0.4461430	0.0529670	-4.1645120
H	-0.4880140	-1.3073140	-5.3018070
C	-3.5768830	-2.6983000	-2.1140210
C	-3.3669050	-3.8393270	-1.1127710
C	-4.5405690	-3.1115290	-3.2321300
H	-4.0116560	-1.8535140	-1.5666490
H	-2.6461780	-3.5774660	-0.3333860
H	-4.3182010	-4.0782400	-0.6274290
H	-3.0250620	-4.7497700	-1.6123880

H	-0.5225240	-1.2115250	-6.4182250
H	1.2333460	-1.1764430	-6.5466130
N	-1.9892840	-0.8024380	0.3192880
P	-3.5518030	-1.0596080	0.5745980
H	-3.5386370	-2.6763560	-1.1100000
C	-3.7744600	-1.0831400	2.4014690
C	-4.6156280	0.2526700	-0.1724850
C	-4.0030810	-2.7113350	-0.1168680
C	-3.3280000	-3.8408410	0.6732170
C	-5.4989440	-2.9921290	-0.3050000
C	-5.0833150	-1.6677800	2.9435330
C	-3.4825100	0.3073360	2.9768570
H	-5.0425320	-1.6453500	4.0360670
H	-5.9603560	-1.0966170	2.6359180
H	-5.2304990	-2.7073470	2.6471490
H	-3.4806820	0.2601220	4.0696480
H	-6.0535970	-2.9505210	0.6347710
H	-5.6118970	-4.0022800	-0.7081560
H	-5.9689030	-2.3069920	-1.0117140
H	-3.4234690	-4.7749600	0.1136350
H	-3.7972240	-3.9896890	1.6492680
H	-4.1020080	1.1696390	0.1391270
C	-6.0568800	0.3553730	0.3413210
C	-4.5594330	0.1776110	-1.7033760
H	-3.5372140	0.0758080	-2.0730880
H	-5.1423680	-0.6611490	-2.0927040
H	-4.9740530	1.0956880	-2.1279270
H	-6.5677280	1.1379260	-0.2266000
H	-6.0976240	0.6412670	1.3936790
H	-6.6225030	-0.5686690	0.2113360
H	-2.5050490	0.6713840	2.6515840
H	-4.2433830	1.0369330	2.6842060
H	-2.9625800	-1.7548380	2.7085470
H	-2.2641160	-3.6581770	0.8288640
H	0.4383050	-2.5814890	-5.8413030

H	-5.4528070	-3.5114010	-2.7790840
H	-4.8331870	-2.2708000	-3.8649360
N	-3.1277400	-0.2644130	0.1327320
P	-4.2213400	-0.2355280	1.2717380
H	-3.7164260	1.6936180	2.5254650
C	-5.7025010	0.6530130	0.6297750
C	-4.7750520	-1.9583020	1.6307020
C	-3.7192940	0.6389050	2.8237830
C	-4.7241390	0.4861660	3.9717600
C	-2.3000000	0.2917560	3.2851480
C	-6.2822350	-0.0803860	-0.5867680
C	-5.3548550	2.0954440	0.2497880
H	-6.5878200	-1.1063490	-0.3672350
H	-7.1617470	0.4571270	-0.9513210
H	-5.5454360	-0.1067910	-1.3952270
H	-4.5852080	2.1031910	-0.5283500
H	-2.2164150	-0.7497420	3.6056970
H	-2.0436200	0.9212850	4.1422830
H	-1.5433210	0.4648300	2.5175790
H	-5.7560540	0.6742840	3.6640510
H	-4.4776080	1.2054580	4.7576290
H	-4.8834490	-2.3468130	0.6114620
C	-6.1223530	-2.1271900	2.3411950
C	-3.6656060	-2.7626530	2.3124840
H	-3.5965930	-2.5241430	3.3780590
H	-3.8747210	-3.8326400	2.2245430
H	-2.6927620	-2.5733250	1.8525650
H	-6.0803260	-1.8099070	3.3844460
H	-6.3923180	-3.1873590	2.3309950
H	-6.9304070	-1.5789860	1.8512590
H	-6.2436750	2.5922480	-0.1487960
H	-4.9980080	2.6875150	1.0964830
H	-6.4443330	0.6602010	1.4366590
H	-4.6708770	-0.5122440	4.4141700
H	-4.1221690	-3.8922430	-3.8727550
H	-0.8036340	-0.5912710	0.7883840

III(Pg$_2$)$_2$ (dication)

C	-0.0171450	-1.7671320	2.7804930
C	-1.4020290	-1.2274150	2.3118690
C	-2.1511510	-0.7631230	3.5789290
C	-1.3928120	-1.2194450	4.8377310
C	-0.0168630	-1.7756620	4.3525540
C	-2.1340690	0.7984190	3.5775960
C	-1.3735570	1.2406280	2.3112080
C	-1.5843780	0.0084220	1.4154440
C	-1.3687350	1.2426380	4.8366000
C	0.0168540	1.7756730	4.3525500
C	0.0171400	1.7671390	2.7804890
C	-1.5015800	0.0131060	5.7464110
C	1.3928030	1.2194570	4.8377310
C	2.1511440	0.7631320	3.5789310
C	1.4020250	1.2274210	2.3118690
C	2.1340620	-0.7984100	3.5776020
C	1.3687250	-1.2426270	4.8366060
C	1.5015680	-0.0130920	5.7464140
C	1.3735520	-1.2406230	2.3112140
C	1.5843750	-0.0084180	1.4154480
H	-1.9323230	-2.0622300	1.8494900
H	-1.8773280	2.0793890	1.8309020
H	-1.9206980	-2.0480840	5.3178210
H	-1.8830660	2.0807480	5.3149520
H	-2.6743470	0.0223530	1.2477940
H	-0.8755170	0.0071360	6.6335170
H	-2.5343560	0.0234760	6.1182150
H	0.8755030	-0.0071200	6.6335190
H	2.5343430	-0.0234620	6.1182200
H	2.6743450	-0.0223490	1.2477990
H	1.9206880	2.0480970	5.3178200
H	1.9323190	2.0622350	1.8494890
H	3.1827950	1.1262830	3.5750000
H	3.1580040	-1.1832220	3.5742280
H	1.8830560	-2.0807350	5.3149610
H	1.8773250	-2.0793850	1.8309110
H	-3.1828020	-1.1262740	3.5749960
H	-3.1580110	1.1832310	3.5742180
H	0.0189970	2.8130830	2.4669390
H	0.0256490	2.8256160	4.6542880
H	-0.0256580	-2.8256050	4.6542950
H	-0.0190020	-2.8130770	2.4669460
P	-1.0977880	0.0485320	-0.3375890
N	-1.4690660	1.4766850	-0.9334530
P	-2.5756170	2.5388150	-1.2755880
N	-1.8145260	3.7482990	-2.1286280
C	-2.6149140	4.8283820	-2.6975690
H	-3.4948210	5.0230510	-2.0824650
H	-2.9419050	4.5958430	-3.7195340

III(Pg$_2$)$_2$ (protonated)

C	-1.1724910	3.2071450	0.5892850
C	-2.1965010	2.5294010	-0.3683940
C	-2.6396730	3.5842620	-1.3920030
C	-2.1744270	4.9791740	-0.9492840
C	-1.2116630	4.7506620	0.2640440
C	-1.8899490	3.2453430	-2.7220350
C	-0.9947350	2.0134700	-2.4334740
C	-1.7996110	1.3778890	-1.2783110
C	-1.0345520	4.4735160	-3.0754210
C	0.4463860	4.0809160	-2.7994180
C	0.4851640	2.5513400	-2.4111310
C	-1.7221900	5.5948800	-2.2839630
C	1.3816550	4.8687910	-1.8233380
C	1.9090240	3.8376020	-0.8182500
C	1.5420400	2.4143600	-1.2711770
C	1.1612060	4.0830370	0.5338430
C	0.2440760	5.2968510	0.3212820
C	0.8862050	5.9868170	-0.8905310
C	0.3305990	2.8071580	0.8396350
C	1.2323150	1.7872290	0.0929700
H	-3.0452600	2.2060330	0.2381320
H	-1.0417880	1.3061610	-3.2648550
H	-3.0206310	5.5643040	-0.5762570
H	-1.1168630	4.7036400	-4.1421550
H	-2.7397710	1.1074150	-1.7865470
H	-1.1810840	6.5348820	-2.2278430
H	-2.6478470	5.8251520	-2.8284230
H	0.3002300	6.7778840	-1.3486110
H	1.7934840	6.4780830	-0.5140290
H	2.1796420	1.9798300	0.6232780
H	2.2036200	5.2694960	-2.4241690
H	2.4220610	1.9189890	-1.6899520
H	2.9894410	3.9279840	-0.6708400
H	1.8871930	4.2634150	1.3327380
H	0.3055760	5.9751400	1.1776680
H	0.4111830	2.5705410	1.9018340
H	-3.7222920	3.5495470	-1.5488370
H	-2.6127620	3.0193600	-3.5115190
H	0.9464630	2.0313890	-3.2555610
H	0.9434920	4.1637710	-3.7695970
H	-1.7231000	5.2279640	1.1042680
H	-1.6169730	3.0897710	1.5818520
P	-1.4632690	-0.2210340	-0.4169830
N	-1.1140050	-1.1379750	-1.7930980
P	-1.4458680	-2.5374280	-2.3754710
N	-0.3139060	-3.0162630	-3.5407400
C	0.9468130	-3.5337070	-3.0188850
H	0.7726920	-4.4334970	-2.4249920
H	1.4904140	-2.8041860	-2.3989870

H	-2.0130250	5.7419780	-2.7266630
C	-0.5870300	3.4763470	-2.8673440
H	-0.7968990	3.1850530	-3.9057290
H	0.0295390	4.3817710	-2.8819500
H	-0.0298140	2.6833970	-2.3712760
N	-3.8511050	1.8256250	-2.0983140
C	-5.0772630	2.5662430	-2.3981270
H	-5.0208150	3.0559660	-3.3786140
H	-5.2721690	3.3259940	-1.6424350
H	-5.9179510	1.8652750	-2.4108250
C	-3.4978140	0.9040380	-3.1784010
H	-4.3297370	0.2115370	-3.3378610
H	-3.3053220	1.4402470	-4.1177560
H	-2.6206740	0.3194980	-2.9065310
N	-3.3424060	3.3470330	-0.0419890
C	-4.3818030	2.7052380	0.7568360
H	-4.8113970	1.8595870	0.2236190
H	-3.9878740	2.3513260	1.7179550
H	-5.1799600	3.4260820	0.9609660
C	-2.7176040	4.4797380	0.6334390
H	-3.4887820	5.2144810	0.8849310
H	-2.2192820	4.1727780	1.5626410
H	-1.9845980	4.9524940	-0.0171980
N	-1.6374130	-1.2361430	-1.1143230
P	-2.7675340	-2.3394450	-1.0853040
N	-2.0674980	-3.8140410	-0.8066530
C	-2.7084790	-5.0569420	-1.2128450
H	-3.1016940	-5.6112120	-0.3516180
H	-3.5310550	-4.8639670	-1.9006510
H	-1.9779930	-5.6915540	-1.7264280
C	-0.9116020	-3.9852470	0.0577790
H	-0.2586140	-4.7482340	-0.3770000
H	-0.3435930	-3.0558500	0.1129240
H	-1.1967100	-4.3103710	1.0680260
N	-3.5252070	-2.3801590	-2.5601310
C	-4.8992880	-2.8200330	-2.7576210
H	-4.9330050	-3.7567280	-3.3279990
H	-5.4592350	-2.0604980	-3.3154090
H	-5.3966340	-2.9854370	-1.8021980
C	-2.7260260	-2.3553620	-3.7784900
H	-2.5334030	-3.3691560	-4.1532360
H	-3.2614510	-1.7951690	-4.5518530
H	-1.7746360	-1.8562970	-3.5930350
N	-3.9718280	-2.1008880	0.0318890
C	-4.3594240	-3.0716020	1.0439990
H	-3.6203840	-3.8684250	1.1182240
H	-4.4251050	-2.5772470	2.0197460
H	-5.3352730	-3.5178780	0.8151910
C	-4.8354630	-0.9315700	-0.0539640
H	-4.8882100	-0.4370500	0.9236920
H	-5.8551160	-1.2092870	-0.3483980
H	-4.4496290	-0.2164840	-0.7856210
P	1.0977890	-0.0485330	-0.3375860
N	1.4690700	-1.4766870	-0.9334450
P	2.5756200	-2.5388180	-1.2755790
N	3.8511080	-1.8256280	-2.0983060
C	3.4978160	-0.9040470	-3.1783970
H	3.3053210	-1.4402610	-4.1177480
H	4.3297380	-0.2115470	-3.3378620
H	2.6206770	-0.3195050	-2.9065280
C	5.0772680	-2.5662460	-2.3981150
H	5.0208200	-3.0559730	-3.3786000
H	5.9179540	-1.8652770	-2.4108160
H	5.2721750	-3.3259930	-1.6424200
N	3.3424090	-3.3470330	-0.0419780
C	4.3818040	-2.7052350	0.7568470
H	3.9878730	-2.3513200	1.7179640
H	4.8113990	-1.8595850	0.2236280
H	5.1799610	-3.4260780	0.9609810
C	2.7176060	-4.4797360	0.6334540
H	3.4887850	-5.2144770	0.8849500
H	2.2192830	-4.1727730	1.5626530
H	1.9846020	-4.9524950	-0.0171830
N	1.8145300	-3.7483030	-2.1286180
C	2.6149180	-4.8283870	-2.6975560
H	3.4948260	-5.0230530	-2.0824530
H	2.0130300	-5.7419830	-2.7266480
H	2.9419080	-4.5958500	-3.7195220
C	0.5870350	-3.4763520	-2.8673360
H	0.0298200	-2.6834000	-2.3712700
H	0.7969060	-3.1850600	-3.9057210
H	-0.0295350	-4.3817740	-2.8819400
N	1.6374150	1.2361400	-1.1143220
P	2.7675360	2.3394420	-1.0853050
N	2.0675010	3.8140380	-0.8066580
C	0.9116020	3.9852470	0.0577700
H	1.1967080	4.3103750	1.0680170
H	1.5895180	-3.8112020	-3.8594910
C	-0.1264360	-2.0636150	-4.6327510
H	0.4283220	-1.1687530	-4.3175570
H	0.4209060	-2.5491810	-5.4465690
H	-1.0972050	-1.7426400	-5.0165970
N	-1.4749680	-3.7467090	-1.2080940
C	-2.0614360	-5.0499660	-1.4657240
H	-2.4579740	-5.4569780	-0.5288170
H	-1.3318720	-5.7680460	-1.8686290
H	-2.8911290	-4.9599870	-2.1669100
C	-0.5439580	-3.7300820	-0.0920940
H	0.2960440	-4.4237530	-0.2436730
H	-0.1433150	-2.7255320	0.0576510
H	-1.0637820	-4.0305830	0.8243310
N	-2.8719980	-2.6510010	-3.2373480
C	-3.1040340	-3.5610580	-4.3459150
H	-3.2986770	-2.9993350	-5.2699670
H	-2.2321470	-4.1946710	-4.5055680
H	-3.9773940	-4.1974200	-4.1496510
C	-3.9798530	-1.7495870	-2.9668570
H	-4.8976680	-2.3228570	-2.7826870
H	-3.7766080	-1.1461200	-2.0800580
H	-4.1577750	-1.0887720	-3.8269110
N	-3.0386980	-0.6052090	0.0246590
P	-3.9550330	-0.7279140	1.2689040
N	-5.1581860	-1.8462520	0.8855000
C	-4.7441000	-3.0796840	0.2278570
H	-4.5439110	-3.8794880	0.9575740
H	-5.5412700	-3.4262740	-0.4403240
H	-3.8424720	-2.9098980	-0.3611540
C	-6.3542770	-2.0163200	1.6979090
H	-7.1889630	-2.3136730	1.0524600
H	-6.2279040	-2.7943940	2.4645040
H	-6.6172380	-1.0809750	2.1918200
N	-4.8708860	0.6031460	1.7350920
C	-4.3354190	1.6187090	2.6329940
H	-3.9097620	2.4685660	2.0811610
H	-3.5560270	1.1961370	3.2655340
H	-5.1395620	2.0015050	3.2715730
C	-5.7972520	1.1527530	0.7520330
H	-6.6373920	1.6277370	1.2698860
H	-5.3143260	1.9071050	0.1137040
H	-6.1884550	0.3585070	0.1150960
N	-3.1784670	-1.1212300	2.7171040
C	-1.9917310	-1.9630140	2.6530980
H	-2.2448200	-3.0276660	2.5331810
H	-1.3347660	-1.6449880	1.8428660
H	-1.4321110	-1.8553960	3.5888170
C	-3.9822410	-1.3977020	3.9033910
H	-4.8821370	-0.7817700	3.9140550
H	-3.3953080	-1.1630820	4.7987270
H	-4.2812190	-2.4543300	3.9587520
P	1.5036600	-0.0188120	0.2701360
N	1.1205060	-0.6213360	1.7115240
P	1.6259560	-0.3234050	3.1713280
N	0.7226290	0.8964000	3.8723670
C	1.0485500	1.4702700	5.1735550
H	2.1201830	1.4115170	5.3634900
H	0.5146600	0.9606040	5.9869800
H	0.7554140	2.5253120	5.1798530
C	-0.7025730	0.9630310	3.5654450
H	-0.8939070	0.6006780	2.5541360
H	-1.2998290	0.3724900	4.2736030
H	-1.0313380	2.0061700	3.6319930
N	1.5159780	-1.7622400	3.9949640
C	1.1800900	-3.0326400	3.3747320
H	0.3536270	-3.5086440	3.9156620
H	2.0409460	-3.7148780	3.3924610
H	0.8742350	-2.8676610	2.3428820
C	1.8129550	-1.8404650	5.4171900
H	2.2180250	-0.8970330	5.7836930
H	0.9132590	-2.0854860	5.9941640
H	2.5649580	-2.6170730	5.6022660
N	3.1863950	0.2133040	3.4346330
C	3.5674260	1.5646840	3.0277510
H	3.9664510	1.5644610	2.0054000
H	2.7073780	2.2348610	3.0726550
H	4.3376210	1.9439840	3.7072500
C	4.2849900	-0.7392810	3.2891580
H	5.1182100	-0.4213470	3.9239430
H	3.9714680	-1.7341670	3.6087740
H	4.6311630	-0.7913360	2.2483940
N	3.1010750	-0.0714940	-0.0254380
P	4.1182980	-0.9555040	-0.8220420
N	5.6262200	-0.7333420	-0.1615560
C	6.1142090	0.6153650	0.0868950
H	5.3057630	1.3356750	-0.0436930

H	0.2586160	4.7482330	-0.3770120	H	6.4814540	0.6954430	1.1169700
H	0.3435930	3.0558500	0.1129170	H	6.9338810	0.8735810	-0.5963210
C	2.7084840	5.0569380	-1.2128510	C	6.6131620	-1.7827980	0.0185070
H	3.1016930	5.6112120	-0.3516250	H	7.0309330	-1.7220700	1.0299450
H	3.5310630	4.8639610	-1.9006520	H	6.1601540	-2.7661680	-0.0990620
H	1.9780000	5.6915480	-1.7264410	H	7.4395530	-1.6938840	-0.6995340
N	3.5252100	2.3801510	-2.5601330	N	4.2327950	-0.5097110	-2.4237370
C	4.8992910	2.8200230	-2.7576230	C	5.4704790	-0.5443740	-3.1872520
H	5.4592360	2.0604900	-3.3154140	H	5.5686550	0.3784590	-3.7692560
H	5.3966390	2.9854240	-1.8022000	H	5.4952590	-1.3922710	-3.8851780
H	4.9330090	3.7567200	-3.3279980	H	6.3288860	-0.6220690	-2.5206810
C	2.7260290	2.3553530	-3.7784910	C	3.0335080	-0.3901250	-3.2381660
H	3.2614530	1.7951580	-4.5518530	H	2.9167110	-1.2496540	-3.9121370
H	1.7746380	1.8562910	-3.5930360	H	2.1407340	-0.3268950	-2.6126580
H	2.5334070	3.3691470	-4.1532380	H	3.0837710	0.5200870	-3.8467630
N	3.9718300	2.1008880	0.0318890	N	3.7425670	-2.5812910	-0.7859710
C	4.3594230	3.0716020	1.0439980	C	4.1696170	-3.5172080	-1.8167060
H	4.4250990	2.5772500	2.0197470	H	3.4019920	-4.2879860	-1.9371010
H	5.3352740	3.5178760	0.8151940	H	4.2891150	-3.0069520	-2.7723630
H	3.6203850	3.8684270	1.1182190	H	5.1138640	-4.0184090	-1.5653110
C	4.8354640	0.9315680	-0.0539590	C	3.3795890	-3.2082420	0.4797820
H	5.8551170	1.2092830	-0.3483910	H	2.5822180	-3.9388200	0.3070150
H	4.4496300	0.2164810	-0.7856160	H	3.0053210	-2.4571350	1.1753920
H	4.8882070	0.4370500	0.9236980	H	4.2310600	-3.7276720	0.9399970
				H	0.8881840	-0.7994300	-0.7093550
**III(Ph$_2$)$_2$ (dication)**				**III(Ph$_2$)$_2$ (protonated)**			
C	-4.711345	0.452543	1.451573	C	5.5634470	-2.9963520	0.1452030
N	-4.323491	-0.928758	1.136029	N	4.8382830	-2.6507320	-1.0890570
C	-4.837066	-1.884551	2.133338	C	4.0537760	-3.8387900	-1.4368570
C	-5.666584	-1.005383	3.078932	C	3.5551810	-4.3858800	-0.0945920
C	-5.041268	0.385867	2.940436	C	4.7165520	-4.0762730	0.8698060
P	-3.448733	-1.325685	-0.209195	P	4.0893280	-1.1387560	-1.1468100
N	-3.260075	-2.963773	-0.228422	N	3.2257570	-0.8022280	0.0993450
C	-4.238811	-3.876995	-0.842183	P	1.8469330	0.0655430	0.5303790
C	-3.616079	-5.268144	-0.653165	C	1.8603610	-0.5241520	2.3015080
C	-2.117842	-4.993546	-0.494020	C	1.1962280	0.1101340	3.5454680
C	-2.092452	-3.685394	0.290452	C	1.9161620	-0.7035280	4.6509810
N	-2.035964	-0.629678	-0.346624	C	2.3085900	-2.0780640	4.0160680
P	-1.062897	0.317198	0.497768	C	1.8320170	-2.0295020	2.5553190
N	-1.258821	1.890823	0.375608	C	0.9666640	-1.0068770	5.8201950
P	-1.647658	3.086075	-0.577047	C	-0.4556540	-0.5722040	5.3595090
N	-1.097028	4.447977	0.181833	C	-0.3279210	0.1205590	3.9458170
C	-1.164147	4.560426	1.655288	C	1.2966350	-2.4724680	6.1384550
C	-1.437903	6.044118	1.895244	C	1.5560420	-3.1677150	4.7918850
C	-0.747406	6.720967	0.711100	C	0.4087840	-3.6559140	3.8484740
C	-1.034768	5.772235	-0.455148	C	0.5406430	-2.9004050	2.4679240
P	1.007129	-0.253236	-0.019529	C	-1.5599000	-0.3539120	3.1075790
C	1.774696	-0.715646	1.566420	C	-2.2396600	-1.4833260	3.9079640
C	2.046763	0.296951	2.698389	C	-1.6879590	-1.5338150	5.3377220
C	2.875582	-0.583302	3.658690	C	-1.8380610	-2.8293150	3.2134610
C	2.495005	-2.069634	3.368454	C	-1.0931460	-3.6694010	4.2595320
C	1.449073	-2.055619	2.236459	C	-1.5749980	-3.0482510	5.5786270
C	2.481942	-0.292495	5.117523	C	-0.9048810	-2.4650060	2.0329220
C	1.220991	0.625832	5.066887	C	-1.4603890	-1.0511100	1.7485770
C	0.947623	0.990978	3.562597	P	-1.4234460	-0.1283560	0.1791270
C	2.446939	-1.699832	5.730726	N	-1.1878950	-1.1719090	-1.0218890
C	1.870144	-2.633298	4.656818	P	-2.1092890	-2.2698730	-1.6840630
C	0.335279	-2.752664	4.392504	N	-1.1226850	-3.5349540	-2.0910700
C	0.072054	-2.376061	2.889159	C	-0.0086890	-4.0089220	-1.2569750
C	-0.586524	0.892041	3.324953	C	0.6419580	-5.0705740	-2.1390190
C	-1.202321	0.308454	4.613805	C	-0.5643090	-5.7134980	-2.8274440
C	-0.147520	0.276694	5.734048	C	-1.4777330	-4.5199450	-3.1266980
C	-1.185651	-1.456587	2.846228	N	3.2910830	-1.0845250	-2.6112640
C	-1.585954	-1.174583	4.309337	C	4.0206120	-1.0058610	-3.8833000
C	-0.762642	-2.060659	5.261139	C	2.9355260	-1.1922090	-4.9541890
C	-1.214736	-0.030731	2.273687	C	1.6366710	-0.7981000	-4.2433360
C	-0.415284	-1.083758	6.393509	C	1.8588620	-1.3115930	-2.8228520
N	0.836383	-1.553484	-0.922918	N	5.4236680	-0.1577180	-1.3124200
P	1.524553	-2.636640	-1.832171	C	6.7553520	-0.5353200	-1.8043230
N	2.699229	-1.971262	-2.800214	C	7.5047250	0.8039210	-1.8939680
C	2.317124	-0.769366	-3.575959	C	6.7755440	1.6992680	-0.8864200
C	3.458568	-0.606835	-4.572732	C	5.3233570	1.2835800	-1.0943760
C	3.844937	-2.055823	-4.879530	N	2.3699380	1.6588610	0.8376350
C	3.746966	-2.734379	-3.511833	P	1.9409130	3.0551870	0.2910960
N	2.288242	-3.910764	-1.103274	N	0.3465070	3.4421050	0.6638810
C	3.580506	-3.733521	-0.409494	C	-0.3061510	3.0354010	1.9103930
C	3.583663	-4.807410	0.687175	C	-0.7779810	4.3417980	2.5937690
C	2.585940	-5.850270	0.177460	C	-0.5383280	5.4545560	1.5529150
C	1.507407	-4.995124	-0.478965	C	-0.2836610	4.6958070	0.2453950
N	0.311077	-3.358207	-2.703718	N	2.8807800	4.3222280	0.8978180
C	-0.951707	-2.671230	-3.016007	C	2.8403130	4.5626630	2.3472300
C	-1.689308	-3.645882	-3.963758	C	4.0332190	3.7871400	2.9337450
C	-0.727921	-4.830443	-4.182612	C	5.0474120	3.7043140	1.7710410
C	0.639448	-4.266955	-3.805539	C	4.3148430	4.3319890	0.5657830
N	1.793856	0.994700	-0.640066	N	2.1067120	3.2801460	-1.3519940
P	3.229974	1.666951	-0.610142	C	2.1090740	2.1467130	-2.2837000

N	3.222746	2.855360	0.546197
C	4.316572	3.804641	0.760433
C	3.983673	4.358740	2.144681
C	2.441533	4.414826	2.155558
C	1.986836	3.516044	0.982511
N	-4.414154	-0.904696	-1.483374
C	-3.915818	-0.853272	-2.865460
C	-5.142198	-0.425466	-3.668210
C	-6.289331	-1.094442	-2.907069
C	-5.888962	-0.906396	-1.442772
N	4.583675	0.791222	-0.251808
C	5.085223	-0.246871	-1.176210
C	6.113815	-1.033414	-0.348475
C	5.721157	-0.757179	1.105121
C	5.263837	0.696733	1.048470
N	3.529446	2.192303	-2.160364
C	2.447825	2.815405	-2.954972
C	3.145591	3.878645	-3.819088
C	4.446732	4.174316	-3.070476
C	4.822870	2.799309	-2.526865
N	-3.271908	3.347317	-0.825239
C	-4.133175	4.034382	0.154877
C	-5.568747	3.715678	-0.302490
C	-5.413711	3.220269	-1.744491
C	-4.074552	2.491703	-1.706179
N	-1.013659	2.932128	-2.105584
C	-1.289409	3.914002	-3.175053
C	-0.823049	3.213120	-4.451165
C	-1.101619	1.737029	-4.166704
C	-0.773773	1.583681	-2.681342
H	2.660427	1.100192	2.288092
H	1.650027	-2.844846	1.509530
H	3.271884	0.278030	5.613850
H	2.240883	-3.644830	4.844986
H	2.786821	-0.887103	1.159463
H	2.001921	-1.777454	6.718048
H	3.495239	-1.999442	5.858472
H	0.331773	-1.424746	7.103885
H	-1.336793	-0.958906	6.976835
H	-2.281814	0.224535	2.349443
H	-1.389805	-2.855760	5.673830
H	-1.973091	-2.023862	2.349182
H	-2.660782	-1.322872	4.452913
H	-2.088901	0.874776	4.913130
H	-0.352528	1.060210	6.468898
H	-0.955854	1.903091	3.141145
H	3.947540	-0.435446	3.501334
H	3.386406	-2.633214	3.075756
H	-0.250513	-3.292872	2.389911
H	0.125039	-3.820755	4.484913
H	1.535427	1.550412	5.556742
H	1.165054	2.056087	3.456479
H	-4.012273	-2.371040	2.666600
H	-5.443934	-2.667304	1.669541
H	-6.707455	-0.977112	2.742441
H	-5.651119	-1.383650	4.102728
H	-5.708323	1.191227	3.252990
H	-4.123347	0.453887	3.535119
H	-3.905065	1.156508	1.216173
H	-5.596901	0.752333	0.876148
H	-6.257493	0.054923	-1.063221
H	-6.281988	-1.694762	-0.795695
H	-7.266617	-0.659118	-3.123393
H	-6.327402	-2.161444	-3.150680
H	-5.076642	-0.733613	-4.713148
H	-5.251877	0.664045	-3.639243
H	-3.079709	-0.155756	-2.947941
H	-1.183311	-3.097209	0.130039
H	-1.636208	-4.847903	-1.464181
H	-3.568389	-1.839425	-3.197122
H	-4.371001	-3.632656	-1.902659
H	-3.845599	-5.931303	-1.489466
H	-5.221374	-3.810947	-0.361894
H	-4.007714	-5.727382	0.259547
H	-1.940600	3.917896	2.076438
H	-0.214162	4.254017	2.103367
H	-1.110089	7.731510	0.515086
H	-1.059149	6.378637	2.863092
H	0.332381	6.775216	0.885032
H	-2.515287	6.237227	1.864323
H	-0.236254	5.804537	-1.200973
H	-1.982046	6.024878	-0.951162
H	-3.963653	3.678090	1.177773
H	-3.927323	5.109606	0.136407
H	-6.223989	4.584464	-0.218718
H	-5.991087	2.922008	0.320426
H	-5.352965	4.064961	-2.437789

C	2.6353150	2.7329230	-3.6097270
C	3.2323150	4.0918020	-3.2259870
C	2.3341440	4.5319260	-2.0729760
N	-2.8390670	0.6525840	0.1903850
P	-3.5301820	1.9592390	-0.3440710
N	-3.7356260	3.0072540	0.9307050
C	-4.2741940	4.3618330	0.7159400
C	-4.6897190	4.8240350	2.1157820
C	-5.0568010	3.5178250	2.8245760
C	-4.0082350	2.5370070	2.3014430
N	-5.0497880	1.7069530	-0.9748330
C	-5.1958130	0.9811020	-2.2495750
C	-6.6951300	0.6996840	-2.3297430
C	-7.0901880	0.4849190	-0.8670430
C	-6.2209910	1.4901620	-0.1025030
N	-2.7381990	2.7091210	-1.5843970
C	-3.3019570	3.7623540	-2.4376690
C	-2.5925220	3.5330950	-3.7690880
C	-1.1792620	3.1475030	-3.3241200
C	-1.3912320	2.3245620	-2.0444220
N	-3.3726510	-2.9227210	-0.8308350
C	-3.3507560	-4.2470030	-0.1848720
C	-4.7431990	-4.3625750	0.4427700
C	-5.1169860	-2.9103990	0.7490210
C	-4.5800290	-2.1617910	-0.4680820
N	-2.8661510	-1.6362250	-3.0186740
C	-2.1767630	-0.7385490	-3.9472950
C	-2.1809190	-1.4973510	-5.2797180
C	-3.4542720	-2.3719200	-5.2273210
C	-3.9494990	-2.2839530	-3.7649180
H	1.5302140	1.1512040	3.5858540
H	2.5717010	-2.4844520	1.8958100
H	1.2255400	-0.3987740	6.6924410
H	2.2073460	-4.0281620	4.9737520
H	2.9180130	-0.2754650	2.4893220
H	0.6191350	-2.9749850	6.8225980
H	2.2673500	-2.4566550	6.6520920
H	-1.0401450	-3.3569860	6.4714940
H	-2.6051810	-3.4019240	5.7197840
H	-2.5211610	-1.3112260	1.5732010
H	-1.4323560	-4.7090360	4.2241070
H	-1.1484360	-3.0747310	1.1625070
H	-2.7332490	-3.3444180	2.8492150
H	-3.3275580	-1.3636120	3.8939710
H	-2.4208820	-1.1317300	6.0435630
H	-2.2360320	0.4978570	3.0087230
H	2.8136640	-0.1768900	4.9888960
H	3.3925980	-2.2205590	4.0669660
H	0.7906290	-3.6646150	1.7253220
H	0.6514310	-4.7030380	3.6486990
H	-0.7450860	0.2164510	6.0590780
H	-0.5328380	1.1811610	4.1140780
H	-1.1769570	-0.4977820	-3.5833420
H	-2.7443920	0.1987880	-4.0271450
H	-2.1668660	-0.8200370	-6.1361680
H	-1.2895200	-2.1291790	-5.3430470
H	-4.2233690	-2.0066710	-5.9113490
H	-3.2329140	-3.4047650	-5.5061790
H	-4.2003480	-3.2518080	-3.3279460
H	-4.8468990	-1.6548900	-3.7063590
H	-1.2785070	-4.1070200	-4.1222770
H	-2.5361500	-4.8011400	-3.0869010
H	-0.3056750	-6.2693790	-3.7304650
H	-1.0627480	-6.4001580	-2.1352630
H	1.2403960	-5.7788250	-1.5629730
H	1.2903420	-4.5974260	-2.8848040
H	0.6500830	-3.1795450	-0.9905640
H	-4.3361470	-1.1192660	-0.2484770
H	-4.6032850	-2.5664820	1.6545510
H	-0.3678680	-4.4705950	-0.3254580
H	-2.5783440	-4.3018520	0.5911510
H	-4.7389710	-5.0059440	1.3249130
H	-3.1549950	-5.0433710	-0.9082390
H	-5.4472990	-4.7819270	-0.2830720
H	-4.3892460	3.6773230	-2.4924110
H	-3.0587330	4.7585160	-2.0430450
H	-0.6302460	2.5826800	-4.0813460
H	-2.6142230	4.4142390	-4.4134250
H	-0.6021250	4.0491290	-3.0948410
H	-3.0659590	2.7022520	-4.3039820
H	-0.6304160	2.5520670	-1.2946510
H	-1.3604870	1.2518960	-2.2559470
H	-3.5139120	5.0145260	0.2752890
H	-5.1380190	4.3400260	0.0380410
H	-5.5107090	5.5425750	2.0808630
H	-3.8442930	5.3041420	2.6195580
H	-6.0570100	3.1927760	2.5191220

H	-6.233982	2.572128	-2.061676
H	-3.608796	2.407588	-2.692227
H	-4.186663	1.478576	-1.299071
H	-2.358545	4.158741	-3.222711
H	-1.401163	0.824402	-2.208800
H	0.265984	1.297071	-2.518454
H	-0.740818	4.841763	-3.005568
H	-0.505735	1.060202	-4.783774
H	0.248278	3.378310	-4.599096
H	-2.158584	1.513817	-4.352220
H	-1.345440	3.588158	-5.333158
H	-1.592715	-5.796652	0.027481
H	-2.201113	-3.888001	1.365155
H	4.295606	4.605020	0.006287
H	5.290513	3.315975	0.713975
H	4.345464	3.661697	2.905923
H	4.446893	5.330750	2.324432
H	2.041550	4.063115	3.110254
H	2.080595	5.435830	2.009925
H	1.553837	4.122973	0.177290
H	1.236445	2.772972	1.255217
H	4.604717	0.987438	1.865586
H	6.138005	1.360677	1.069102
H	4.892455	-1.403736	1.415905
H	6.543170	-0.906375	1.807814
H	7.120105	-0.648016	-0.536838
H	6.112022	-2.095906	-0.601011
H	4.258942	-0.881892	-1.509412
H	5.502841	2.833410	-1.673708
H	4.262785	4.866796	-2.242013
H	5.544422	0.202274	-2.062121
H	1.684328	3.254870	-2.304449
H	2.518189	4.761289	-3.960437
H	1.955402	2.065493	-3.579851
H	3.372363	3.466145	-4.807185
H	0.808630	-4.605003	0.275491
H	0.925216	-5.533207	-1.229190
H	3.229514	-4.387067	1.633144
H	3.054895	-6.493943	-0.573382
H	4.583382	-5.211304	0.855451
H	2.183574	-6.482060	0.971393
H	4.404534	-3.885567	-1.113306
H	3.679412	-2.725022	0.008455
H	1.368735	-0.930967	-4.107484
H	2.205132	0.083423	-2.908569
H	4.291003	-0.076885	-4.098091
H	3.153683	-0.041704	-5.455724
H	4.839904	-2.158723	-5.316824
H	3.119674	-2.496914	-5.571959
H	4.697829	-2.639393	-2.972118
H	3.503929	-3.797489	-3.575133
H	-1.489825	-2.460771	-2.093703
H	1.347480	-5.033237	-3.479016
H	1.086393	-3.724071	-4.653010
H	-0.779165	-1.708512	-3.515281
H	-0.750878	-5.210263	-5.205784
H	-1.917502	-3.149119	-4.909666
H	-0.976149	-5.657571	-3.511004
H	-2.637547	-3.982468	-3.536447
H	5.223396	4.600363	-3.708904
H	5.300213	2.196759	-3.310503

H	-5.0409440	3.5990570	3.9133140
H	-4.3464650	1.4983810	2.3124580
H	-3.0935990	2.5827770	2.9059590
H	-4.8447970	1.5914360	-3.0863500
H	-6.7483640	2.4384590	0.0555390
H	-5.9379240	1.0957560	0.8778630
H	-4.6115060	0.0520510	-2.2433970
H	-6.8407120	-0.5341140	-0.5565650
H	-6.9195200	-0.1603300	-2.9649460
H	-8.1549250	0.6396000	-0.6819320
H	-7.2160240	1.5711320	-2.7392670
H	-6.1896140	-2.7608780	0.8902800
H	-5.3070320	-2.1854330	-1.2921110
H	1.1096730	1.7162700	-2.4128590
H	2.7501350	1.3478420	-1.9107130
H	3.3613140	2.0684400	-4.0847820
H	1.8091460	2.8769730	-4.3134530
H	4.2593180	3.9736250	-2.8630080
H	3.2395650	4.8040720	-4.0540780
H	1.3933800	4.9479620	-2.4644160
H	2.7862610	5.2804940	-1.4213580
H	-1.2340960	4.4660100	-0.2557460
H	0.3450860	5.2476830	-0.4540170
H	-1.3829750	6.1439340	1.4710890
H	0.3481540	6.0399910	1.8131630
H	-0.2299810	4.5353400	3.5195820
H	-1.8345630	4.2700670	2.8582360
H	-1.1745350	2.4074310	1.6782620
H	4.4975840	3.8069470	-0.3734340
H	5.3042440	2.6634240	1.5631880
H	0.3804110	2.4458990	2.5158140
H	1.8851160	4.2613260	2.7769590
H	3.7102390	2.7840040	3.2211330
H	2.9553500	5.6398940	2.5207660
H	4.4403780	4.2837620	3.8179030
H	3.2582090	-3.5908040	-2.1401760
H	4.7138260	-4.5671710	-1.9259170
H	4.3517430	-3.7171340	1.8352170
H	3.3107610	-5.4503070	-0.1365580
H	5.3202110	-4.9679870	1.0586850
H	2.6556150	-3.8391520	0.2046600
H	6.5483050	-3.3979160	-0.1213020
H	5.7209990	-2.1056460	0.7568240
H	7.2474770	-1.2146170	-1.0987730
H	6.7117530	-1.0427590	-2.7733580
H	7.3980670	1.2242670	-2.8998090
H	8.5713940	0.6916850	-1.6875600
H	6.9385010	2.7661040	-1.0587800
H	7.0894190	1.4623900	0.1361650
H	4.9150350	1.7971700	-1.9801360
H	4.6589510	1.4931340	-0.2511320
H	4.5198550	-0.0320140	-3.9696840
H	1.2557880	-0.8048820	-2.0651280
H	1.6036310	-2.3803590	-2.7827990
H	4.7932820	-1.7806300	-3.9576290
H	0.7531850	-1.2412530	-4.7085180
H	2.8854340	-2.2447010	-5.2515920
H	1.5112130	0.2891020	-4.2420170
H	3.1349960	-0.5998790	-5.8498020
H	5.9751240	4.2404100	1.9862700
H	4.6258820	5.3751800	0.4303370
H	-0.3984670	0.8032440	0.1117950

Naphtalene

C	-0.7118960	0.0011520	0.0000000
C	-1.4020010	-1.2407850	0.0000000
C	-0.7118960	-2.4257790	0.0000000
C	0.7042820	-2.4280450	0.0000000
C	1.3981000	-1.2452420	-0.0000000
C	0.7118960	-0.0011520	0.0000000
H	-2.4884570	-1.2359780	0.0000000
H	-1.2490710	-3.3687970	0.0000000
H	1.2384630	-3.3727770	0.0000000
H	2.4845750	-1.2436650	-0.0000000
C	1.4020010	1.2407850	0.0000000
C	0.7118960	2.4257790	-0.0000000
C	-0.7042820	2.4280450	-0.0000000
C	-1.3981000	1.2452420	-0.0000000
H	2.4884570	1.2359780	0.0000000
H	1.2490710	3.3687970	-0.0000000
H	-1.2384630	3.3727770	0.0000000
H	-2.4845750	1.2436650	-0.0000000

I

C	-0.2276950	0.7794880	1.2174100
C	-1.0970060	1.1270200	-0.0169640
C	-2.1306310	-0.0001210	-0.0598660
C	-1.0968000	-1.1271750	-0.0170490
C	-0.2275940	-0.7794120	1.2174380
C	-0.1089590	-0.7900050	-1.1651480
C	-0.1090040	0.7900440	-1.1651430
C	1.2608300	0.7832440	0.7549000
C	1.2222280	1.3600330	-0.6603500
C	1.2609310	-0.7830960	0.7550460
C	1.2223920	-1.3599470	-0.6602610
H	2.0862030	1.0844420	-1.2730140
H	2.0864270	-1.0846920	-1.2729760
H	-2.8032470	-0.0002640	0.8045470
H	-2.7314180	-0.0001590	-0.9757350
H	1.9911560	-1.2477010	1.4205100
H	1.1755260	2.4546700	-0.6287000
H	1.1756430	-2.4545910	-0.6285120
H	-1.4775110	2.1508420	-0.0405050
H	-0.4204640	1.1922280	-2.1326770
H	-0.4940790	1.2980830	2.1399380
H	-0.4939740	-1.2980460	2.1399480
H	-1.4771270	-2.1510630	-0.0405610

H	-0.4202370	-1.1922310	-2.1327130
H	1.9909410	1.2480430	1.4203730

II

C	-2.2185430	-0.7720260	-0.0372230
C	-0.7851520	-1.2933590	0.0037480
C	0.1151250	-0.7904510	-1.1504970
C	0.1149850	0.7908560	-1.1502700
C	-0.7852400	1.2933810	0.0040020
C	-2.2186160	0.7718700	-0.0373370
C	1.4745120	1.3380690	-0.6759860
C	1.5108740	0.7816650	0.7519570
C	1.5108900	-0.7819110	0.7518430
C	1.4746210	-1.3378220	-0.6762670
C	0.0174850	0.7794700	1.2114180
C	0.0174510	-0.7796940	1.2112220
H	2.3275840	-1.0346100	-1.2886190
H	2.3273380	1.0347320	-1.2885080
H	2.2422030	-1.2553330	1.4099970
H	1.4529070	2.4333030	-0.6667890
H	1.4528390	-2.4330540	-0.6674480
H	-0.7923570	2.3896670	-0.0028190
H	-0.1946420	1.1823980	-2.1235320
H	-0.2308840	1.2651800	2.1578350
H	-0.2310100	-1.2656800	2.1574830
H	-0.7921790	-2.3896530	-0.0033810
H	-0.1944680	-1.1817970	-2.1238420
H	2.2421330	1.2550170	1.4102390
H	-2.7699730	-1.1630020	0.8249720
H	-2.7198320	-1.1623770	-0.9296830
H	-2.7705020	1.1628810	0.8245270
H	-2.7195030	1.1620420	-0.9300920

III

C	-1.5045920	2.1732420	0.0001760
C	-1.3834450	1.2632890	-1.2302670
C	-2.1442690	-0.0002990	-0.7800940
C	-2.1440480	-0.0003330	0.7804580
C	-1.3830830	1.2632430	1.2306230
C	-0.0003280	0.7852960	-1.7791020
C	-0.0000890	-0.7851180	-1.7791220
C	-1.3830510	-1.2635210	-1.2303770
C	1.3826760	1.2636650	-1.2305190
C	2.1440470	0.0003250	-0.7804590
C	1.3830780	-1.2632480	-1.2306210
C	1.5038890	2.1735850	-0.0000570
C	1.3830470	1.2635240	1.2303690
C	2.1442670	0.0002980	0.7800940
C	1.3834470	-1.2632860	1.2302750
C	0.0003280	-0.7852940	1.7791120
C	0.0000870	0.7851210	1.7791120
C	-1.3826730	-1.2636690	1.2305170
C	1.5045890	-2.1732410	-0.0001670
C	-1.5038820	-2.1735850	0.0000480
H	0.8766330	3.0607450	0.0001800
H	-0.8775260	3.0605500	0.0000390
H	1.9058710	1.7438140	2.0632730
H	1.9052620	1.7439760	-2.0635600
H	1.9064440	-1.7433790	2.0631860
H	1.9057990	-1.7433380	-2.0637050
H	2.5369950	2.5462620	-0.0002180
H	0.8775300	-3.0605530	-0.0000220
H	2.5377890	-2.5456270	-0.0003750
H	-0.8766210	-3.0607400	-0.0001980
H	-2.5369850	-2.5462680	0.0002140
H	-2.5377910	2.5456340	0.0003790
H	-1.9058830	-1.7438080	-2.0632780
H	-1.9064340	1.7433850	-2.0631810
H	-3.1715090	-0.0004140	-1.1561440
H	-3.1711550	-0.0005440	1.1568500
H	-1.9052640	-1.7439860	2.0635500
H	-1.9057990	1.7433280	2.0637130
H	3.1715080	0.0004280	1.1561440
H	3.1711540	0.0005400	-1.1568500
H	-0.0005340	1.0997410	-2.8262580
H	-0.0001840	-1.0994710	-2.8263010
H	0.0005290	-1.0997200	2.8262740
H	0.0001880	1.0994910	2.8262850

$HCOO^-$

C	-0.011486	-0.000000	-0.005234
O	0.007448	-0.000000	1.245870
O	0.945071	0.000000	-0.811842
H	-1.043197	-0.000000	-0.475346

CO2

C	0.000929	-0.000000	0.000001
O	-0.000464	0.000000	1.162232
O	0.002578	-0.000000	-1.162229

I(Pa_2)$_2$

C	-0.7741300	1.8968850	-1.6103140
C	0.7932730	1.8935230	-1.6620110
C	1.4688560	1.3713980	-0.3976270
C	0.8369030	2.3131580	0.6483390
C	-0.7428750	2.3627070	0.6905290
C	-1.4268130	1.4199870	-0.3074290
C	0.8039830	3.4387280	-1.4619720
C	1.1856760	3.6986130	0.0214390
C	-1.0569830	3.7496470	0.0672580
C	-0.7492090	3.4528850	-1.4259850
C	0.0949710	4.6476020	0.5111990
P	1.6734860	-0.4914750	-0.2296800
N	3.0697540	-0.6582350	-1.1979590
P	4.5711690	-0.5446300	-0.7097630
C	5.6752510	-0.5688610	-2.1514060
P	-1.6417160	-0.3703110	0.2098800
N	-2.9421860	-0.1489110	1.2906920
P	-4.4842830	-0.1822900	0.9379150
C	-5.0969030	0.9091560	-0.3943420
N	2.2508320	-0.5492300	1.3790380
P	1.5090610	-1.2240880	2.6004290
C	2.7328810	-1.6412620	3.8774530
C	0.2910630	-0.1882280	3.4791170
C	0.6110330	-2.7732070	2.2643690
C	5.0725450	0.9392470	0.2333510
C	5.1582840	-1.9179720	0.3380720
N	-2.3569900	-0.9749910	-1.2226600
P	-1.6645200	-2.0252720	-2.1811760
C	-0.4923150	-1.3431510	-3.4005780
C	-0.7294040	-3.3693320	-1.3828250
C	-2.9359070	-2.8416140	-3.1883940
C	-5.1444940	-1.8172940	0.4695160

I(Pa_2)$_2$ (diprotonated)

C	-1.0886220	1.6867470	-1.6053060
C	0.4687880	1.8463820	-1.6282230
C	1.1632160	1.6808110	-0.2781620
C	0.3979780	2.7166420	0.5712420
C	-1.1802620	2.5807850	0.5822360
C	-1.7403130	1.4103010	-0.2469600
C	0.3077560	3.3943380	-1.7231000
C	0.6088330	3.9808020	-0.3160410
C	-1.6260900	3.7764330	-0.3043070
C	-1.2372560	3.2413300	-1.7107340
C	-0.5975950	4.8684410	-0.0305170
P	1.6872080	0.0479400	0.3139520
N	2.4267120	-0.7341390	-0.8804330
P	3.8853630	-0.6357680	-1.5284750
C	4.0275430	-1.9579480	-2.7464670
P	-1.9403830	-0.2325100	0.5379180
N	-3.3325580	-0.2087100	1.3480040
P	-4.7767350	-0.6598010	0.8200020
C	-5.2238960	-0.0147370	-0.8161990
N	2.4769980	0.3288740	1.6832190
P	2.6494450	-0.5739610	2.9959890
C	4.3607500	-0.5025530	3.5645540
C	1.6193210	0.0414050	4.3485460
C	2.2327480	-2.3243680	2.7741000
C	4.2166160	0.9211350	-2.3957880
C	5.2276680	-0.8405440	-0.3312310
N	-1.7832400	-1.4018060	-0.5202050
P	-1.0873630	-2.4028670	-1.5229260
C	-0.3063320	-1.6062960	-2.9474840
C	0.2046740	-3.4203380	-0.7652900
C	-2.3265450	-3.5414190	-2.1845830
C	-4.9638610	-2.4570190	0.7076380

C	-5.4434390	0.2986600	2.4027470	C	-6.0150300	-0.0659260	1.9888120
H	0.1096960	5.6193260	0.0045980	H	-0.6769030	5.7155840	-0.7188290
H	0.1185750	4.8120830	1.5941680	H	-0.6272950	5.2402080	0.9982630
H	1.2188600	1.5104380	-2.5930500	H	0.9653940	1.3456720	-2.4598610
H	-2.4728900	1.7509090	-0.3811580	H	-2.8039570	1.6416610	-0.3985560
H	2.5193990	1.6878940	-0.4584290	H	2.1714260	2.1094140	-0.4017680
H	-2.0712380	4.1025430	0.2707690	H	-2.6783590	4.0392590	-0.1818470
H	-1.1187070	2.2593320	1.7124230	H	-1.5755190	2.6270380	1.5992830
H	-1.2813260	4.0750850	-2.1487500	H	-1.8096660	3.6490070	-2.5439620
H	1.3161100	4.0664560	-2.1943170	H	0.7766310	3.9141960	-2.5585230
H	2.2219570	4.0061460	0.1836420	H	1.5969320	4.4310070	-0.2041140
H	1.2597820	2.1732490	1.6460750	H	0.7988450	2.8206450	1.5804150
H	-1.2606470	1.5123220	-2.5089240	H	-1.4985010	1.0951500	-2.4237470
H	3.4388140	-2.3725740	3.4770560	H	5.0097670	-0.9687280	2.8206570
H	3.2801880	-0.7391460	4.1602380	H	4.6506950	0.5425320	3.6902120
H	2.2451290	-2.0583600	4.7620020	H	4.4647850	-1.0266080	4.5171490
H	1.2913770	-3.4983890	1.8111830	H	2.8264690	-2.7384330	1.9558070
H	0.1954820	-3.1881420	3.1863730	H	2.4459850	-2.8770730	3.6916000
H	-0.1958890	-2.5393010	1.5642680	H	1.1708900	-2.4278740	2.5381650
H	-0.5606620	-0.0015510	2.8198150	H	0.5684530	-0.0243960	4.0570520
H	-0.0574780	-0.6860980	4.3884830	H	1.7836800	-0.5445420	5.2556990
H	0.7586920	0.7631400	3.7449510	H	1.8713170	1.0869350	4.5385360
H	5.4622420	0.2937230	-2.7866400	H	3.2486430	-1.8306450	-3.5010980
H	6.7205820	-0.5339970	-1.8339790	H	5.0082610	-1.9315270	-3.2257870
H	5.5033650	-1.4826330	-2.7244220	H	3.8894440	-2.9186370	-2.2463880
H	4.9109210	1.8348030	-0.3718030	H	3.4371550	1.0953200	-3.1406520
H	4.4467050	0.9911790	1.1271590	H	4.2254570	1.7475890	-1.6814680
H	6.1269810	0.8813410	0.5180340	H	5.1883330	0.8736740	-2.8931300
H	4.5724250	-1.9049790	1.2590230	H	5.1689940	-0.0360960	0.4054140
H	4.9891620	-2.8643630	-0.1812600	H	5.1128160	-1.8020250	0.1741390
H	6.2220450	-1.8148950	0.5686450	H	6.1960060	-0.8060630	-0.8356590
H	0.3499280	-0.9013440	-2.8615760	H	0.5117850	-0.9748110	-2.5919330
H	-0.1251500	-2.1241680	-4.0721510	H	0.0993580	-2.3701530	-3.6159470
H	-0.9922430	-0.5668780	-3.9849820	H	-1.0426250	-1.0070830	-3.4864280
H	-3.6159040	-3.3922280	-2.5343150	H	-2.7211210	-4.1599300	-1.3755740
H	-3.5040480	-2.0855020	-3.7348340	H	-3.1398660	-2.9662840	-2.6321080
H	-2.4798850	-3.5339830	-3.9003800	H	-1.8774550	-4.1868780	-2.9427320
H	0.1089570	-2.9123390	-0.8483830	H	1.0867210	-2.8015560	-0.5784150
H	-1.3756880	-3.8924370	-0.6737400	H	-0.1670840	-3.8460660	0.1687930
H	-0.3553350	-4.0766820	-2.1275300	H	0.4805660	-4.2274070	-1.4485670
H	-4.8818410	1.9512450	-0.1452390	H	-5.2287270	1.0773850	-0.7933480
H	-4.5694420	0.6433690	-1.3130000	H	-4.4864040	-0.3576300	-1.5467600
H	-6.1745350	0.7859350	-0.5351670	H	-6.2136770	-0.3730630	-1.1084170
H	-4.6446700	-2.1216580	-0.4522170	H	-4.2366900	-2.8348830	-0.0132220
H	-4.9135770	-2.5379720	1.2577550	H	-4.7640180	-2.9017770	1.6845620
H	-6.2264060	-1.7800060	0.3164580	H	-5.9734020	-2.7192050	0.3835200
H	-6.5149980	0.2434940	2.1948720	H	-7.0111440	-0.3873190	1.6774180
H	-5.2024480	-0.3721890	3.2301580	H	-5.7950930	-0.4705980	2.9787280
H	-5.1836620	1.3207100	2.6872350	H	-5.9789940	1.0243850	2.0298190
				H	-0.9867570	-0.3058480	1.5669580
				H	0.5795120	-0.7749870	0.5352460
**I(Pb$_2$)$_2$**				**I(Pb$_2$)$_2$ (diprotonated)**			
C	0.4015100	-2.8076870	4.1724500	C	-0.5363240	-3.7421810	-3.3619170
C	1.3073700	-1.6396090	3.7933770	C	-1.4678540	-2.5438590	-3.2187790
C	1.1888510	-1.5180960	2.2481570	C	-1.2762390	-2.0653280	-1.7503530
C	-0.3145490	-1.9537850	1.9914220	C	0.2501920	-2.3904070	-1.4850230
C	-0.8169580	-2.2624510	3.4329930	C	0.6948210	-3.0071380	-2.8442360
C	-1.2844780	-0.8260710	1.5884020	C	1.1995990	-1.1783420	-1.4312660
C	-1.0588710	0.1337320	2.7528120	C	0.8697600	-0.5025090	-2.7634580
C	0.4278210	0.5725560	2.9905860	C	-0.6405660	-0.1801580	-3.0233330
C	1.4833880	-0.0186120	2.0516240	C	-1.6224900	-0.5704020	-1.9162530
C	-0.9713890	-0.8195330	3.9852140	C	0.7638920	-1.7294610	-3.7252640
C	0.5001100	-0.3839120	4.2267080	C	-0.7331310	-1.4077830	-3.9828020
P	1.8994160	0.6299580	0.3341950	P	-1.9360730	0.5294840	-0.4922620
N	3.6167650	0.6099870	0.5184740	N	-3.5164090	0.6739830	-0.2629710
P	4.5799010	-0.3498730	-0.2880220	P	-4.6793040	-0.1570400	0.4466800
C	6.2830240	-0.1637130	0.3440140	C	-6.2357340	0.1581580	-0.4319420
C	6.4290100	-0.5544050	1.8144760	C	-6.2098000	-0.3293400	-1.8813720
P	-1.3771930	-0.1921680	-0.1672950	P	1.6038920	-0.2760710	0.0984910
N	-2.6393390	0.9282610	-0.0202880	N	2.7459760	0.8066860	-0.2161100
P	-4.1810230	0.9285370	0.3137640	P	4.3006030	0.7745530	-0.6015140
C	-4.6663830	2.5210830	1.0757460	C	4.6825820	2.1727370	-1.6989840
C	-4.1588330	2.7288210	2.5015110	C	4.1107100	2.0503340	-3.1108480
N	1.5096320	2.2590340	0.6010520	N	-1.1778360	1.8926740	-0.8056510
P	1.0395150	3.3247250	-0.4647220	P	-0.8715170	3.3129770	-0.1446890
C	0.0219630	4.6055490	0.3478950	C	-0.0144720	4.3222530	-1.3862140
C	-1.1214070	4.0186960	1.1740500	C	1.1454140	3.5788000	-2.0465470
C	2.3999960	4.2871630	-1.2319460	C	-2.3271990	4.2783640	0.3677150
C	3.3317610	3.4022420	-2.0595040	C	-3.0824810	3.6889440	1.5591310
C	0.0464150	2.6923700	-1.8691400	C	0.2245400	3.1520010	1.3025510
C	-0.2881480	3.6904190	-2.9768580	C	0.5536680	4.4509380	2.0391290
N	-2.0368190	-1.6183030	-0.8475870	N	1.9089840	-1.4183790	1.1841130
P	-1.4799820	-2.3224550	-2.1496050	P	1.4561940	-1.7521820	2.6777870
C	-0.4175920	-3.7800970	-1.8041730	C	0.3353330	-3.1879100	2.7192350
C	0.9718910	-3.3947990	-1.2951930	C	-1.0363820	-2.9168130	2.1031650
C	-2.8371470	-2.9982800	-3.1703640	C	2.8885940	-2.2109830	3.6969530

C	-3.7720430	-3.9290240	-2.3973070
C	-0.4696980	-1.3101330	-3.3020060
C	-1.2007180	-0.0975630	-3.8794010
C	4.1774760	-2.1341950	-0.1432650
C	5.0650330	-3.1153810	-0.9075880
C	4.7776580	-0.0643220	-2.0944250
C	3.5020170	-0.2853240	-2.9089050
C	-5.2795920	0.8742510	-1.1614740
C	-4.8390910	-0.1124660	-2.2396090
C	-4.8656380	-0.3260490	1.4756950
C	-4.9861450	-1.7417560	0.9074460
H	0.2451060	-2.9022830	5.2530590
H	0.7508010	-3.7689960	3.7793160
H	0.5599550	1.6391690	3.1758550
H	-2.2881130	-1.2536430	1.7262680
H	2.4447580	0.1661690	2.5538820
H	-1.7268500	-2.8675670	3.4587470
H	-0.3727730	-2.8218610	1.3295500
H	-1.6921050	-0.7023420	4.7975990
H	0.7685960	0.0323350	5.2002250
H	2.3393140	-1.6732730	4.1519110
H	1.8833320	-2.1765280	1.7171820
H	-1.7856870	0.9455130	2.8066120
H	3.1348790	-2.2225710	-0.4628860
H	4.1821890	-2.3544840	0.9298690
H	6.5470470	0.8887570	0.1956210
H	6.9555660	-0.7539200	-0.2875440
H	5.1273570	0.9680320	-2.2027750
H	5.5861470	-0.7092560	-2.4570070
H	1.9761770	5.0992870	-1.8330560
H	2.9491940	4.7511500	-0.4055410
H	0.6031080	1.8391130	-2.2708620
H	-0.8599540	2.2674940	-1.4248300
H	0.6967070	5.1934320	0.9798020
H	-0.3535590	5.2824770	-0.4275190
H	-5.2886260	1.8919040	-1.5687740
H	-6.2986000	0.6627270	-0.8165560
H	-4.2749280	3.3008470	0.4135260
H	-5.7599370	2.5864480	1.0398790
H	-4.2071700	-0.3155270	2.3508950
H	-5.8437740	0.0421980	1.8081600
H	-2.3901580	-3.5126920	-4.0284060
H	-3.3963040	-2.1425170	-3.5604210
H	0.4077210	-0.9826630	-2.7356530
H	-0.1125230	-1.9739840	-4.0978310
H	-0.9535470	-4.3631440	-1.0478020
H	-0.3473960	-4.3994900	-2.7050900
H	3.1656140	-1.3256250	-2.8492150
H	2.6887040	0.3455320	-2.5399630
H	3.6740260	-0.0508800	-3.9629120
H	5.0051350	-2.9550500	-1.9878830
H	6.1146800	-3.0281760	-0.6111340
H	4.7495120	-4.1432910	-0.7091280
H	6.2613200	-1.6257010	1.9608440
H	7.4348390	-0.3232700	2.1744210
H	5.7080870	-0.0106080	2.4301540
H	-1.7381310	4.8154530	1.6002290
H	-1.7621730	3.3654600	0.5731050
H	-0.7243680	3.4113780	1.9919840
H	-0.9192530	3.2100150	-3.7304480
H	-0.8365270	4.5570210	-2.5951680
H	0.6088900	4.0573990	-3.4843770
H	4.1890590	3.9721890	-2.4275890
H	3.7022080	2.5749690	-1.4468500
H	2.8170590	2.9754110	-2.9267020
H	-3.0699130	2.6551480	2.5407580
H	-4.4422800	3.7199750	2.8659140
H	-4.5765560	1.9889910	3.1905570
H	-4.8554330	-1.1416990	-1.8761900
H	-5.4921900	-0.0408910	-3.1142570
H	-3.8132340	0.0991530	-2.5486320
H	-4.0507160	-2.0540950	0.4305170
H	-5.2343570	-2.4493580	1.7037060
H	-5.7827270	-1.7947840	0.1587730
H	0.9083980	-2.6234390	-0.5206610
H	1.4865400	-4.2596630	-0.8677140
H	1.5945660	-3.0056730	-2.1064030
H	-0.5248320	0.4866890	-4.5105190
H	-2.0570130	-0.3928000	-4.4928050
H	-1.5592270	0.5542780	-3.0780940
H	-4.1953460	-3.4153450	-1.5302090
H	-4.5943270	-4.2607570	-3.0367300
H	-3.2468640	-4.8196890	-2.0395890

I(Pc$_2$)$_2$

C	3.7262920	-3.3376270	3.0890630
C	0.5935090	-0.3903110	3.5357730
C	1.4446430	0.8646820	3.7418860
C	-4.3925080	-1.9592890	0.4517350
C	-5.5100120	-2.7990940	1.0727730
C	-4.9473510	0.3634020	2.1712610
C	-3.7044400	0.2063380	3.0485910
C	5.3853180	1.0265850	0.8443730
C	4.9617030	0.2625450	2.0962600
C	4.8800750	-0.7169160	-1.4837420
C	4.9421290	-2.0018650	-0.6534560
H	-0.4298990	-4.0796180	-4.3974430
H	-0.8259730	-4.5924790	-2.7368480
H	-0.8230310	0.8063530	-3.4475100
H	2.2004810	-1.6183260	-1.5712280
H	-2.6182170	-0.5424800	-2.3814480
H	1.6243740	-3.5755860	-2.7767930
H	0.3806040	-3.0734530	-0.6439150
H	1.4433300	-1.7816980	-4.5760830
H	-1.0646600	-1.2419690	-5.0078010
H	-2.5141930	-2.6878960	-3.4946000
H	-1.9191640	-2.6007690	-1.0480630
H	1.5540380	0.2959470	-3.0486600
H	-3.4492610	-2.1293450	0.9807740
H	-4.2239620	-2.2438740	-0.5924230
H	-6.3898130	1.2410760	-0.3873580
H	-7.0495510	-0.3065770	0.1334280
H	-5.2642800	1.4106570	2.1291410
H	-5.7906660	-0.2062450	2.5745870
H	-1.9778430	5.2945390	0.5806650
H	-2.9759280	4.3404500	-0.5117430
H	-0.2642550	2.4374010	1.9748530
H	1.1393430	2.6723910	0.9341720
H	-0.7657170	4.6131070	-2.1274900
H	0.3232390	5.2402270	-0.8944290
H	5.3828620	2.1037310	1.0417640
H	6.4018190	0.7604800	0.5350510
H	4.2956900	3.0646250	-1.1952270
H	5.7735920	2.2674950	-1.7262770
H	4.2208170	-0.8418320	-2.3491880
H	5.8706790	-0.4701910	-1.8814500
H	2.5070820	-2.4869400	4.6856400
H	3.4951560	-1.3105320	3.8254460
H	-0.3014230	-0.1561790	2.9481870
H	0.2416270	-0.7845140	4.4949280
H	0.8492750	-3.9859420	2.1740210
H	0.2410410	-3.5122130	3.7605020
H	0.5301830	0.5110080	0.5176770
H	-3.4313940	-0.8455380	3.1695120
H	-2.8481360	0.7404750	2.6245590
H	-3.8928750	0.6166050	4.0425690
H	-5.6571900	-2.5581270	2.1286520
H	-6.4603540	-2.6572580	0.5515990
H	-5.2502180	-3.8577300	1.0075970
H	-6.1348120	-1.4188560	-1.9389610
H	-7.1288100	-0.0317420	-2.3903950
H	-5.3643040	0.1037850	-2.4225360
H	1.6540380	4.2314080	-2.7597200
H	1.8775190	3.2367950	-1.3097450
H	0.7780120	2.6980650	-2.5793400
H	1.2269980	4.2368240	2.8724800
H	1.0549880	5.1694490	1.3855410
H	-0.3413360	4.9266250	2.4484830
H	-3.9431360	4.3163940	1.8008660
H	-3.4473310	2.6888420	1.3178380
H	-2.4542320	3.6266250	2.4523160
H	3.0288180	1.9043370	-3.0866950
H	4.3107480	2.9657140	-3.6719610
H	4.5597840	1.2180940	-3.6586330
H	4.9759480	-0.8180810	1.9406620
H	5.6359290	0.4967760	2.9230630
H	3.9490960	0.5470780	2.3881330
H	3.9939510	-2.2135180	-0.1501380
H	5.1775130	-2.8463870	-1.3047280
H	5.7242620	-1.9416940	0.1075480
H	-0.9384250	-2.5217100	1.0882090
H	-1.6139430	-3.8419150	2.0451830
H	-1.6079850	-2.2016660	2.7008110
H	0.8381350	1.6523410	4.1949270
H	2.2899340	0.6739660	4.4074370
H	1.8368470	1.2493630	2.7958430
H	4.0596750	-3.0773330	2.0812670
H	4.6093950	-3.5152140	3.7062830
H	3.1642740	-4.2733660	3.0315000
H	-1.3854710	-0.1210220	0.6392230

I(Pc$_2$)$_2$ (diprotonated)

C	-0.4478610	-1.7359460	4.0330850	C	-1.0428940	-2.3804110	3.9099220
C	-0.7941410	-0.2935430	4.4940580	C	-1.2088860	-0.9705060	4.5389760
C	0.3860950	0.0957810	5.3803980	C	-0.0210600	-0.8547410	5.4887990
C	1.4429220	-0.2411870	4.3319110	C	1.0209010	-1.2088090	4.4328180
C	1.1004360	-1.6969270	3.9113180	C	0.4991570	-2.5440010	3.8338340
C	0.9994050	0.5253930	3.0533220	C	0.7368980	-0.2387920	3.2489200
C	-0.5772350	0.4848530	3.1623690	C	-0.8321220	-0.0729170	3.3262470
C	-0.5763200	-1.7585090	2.4791840	C	-1.1075540	-2.2111470	2.3598110
C	-1.3001390	-0.4463100	2.1707990	C	-1.6326140	-0.7776670	2.2167250
C	0.9863950	-1.7125890	2.3506600	C	0.4497360	-2.3757770	2.2809870
C	1.6002270	-0.3670950	1.9576090	C	1.2608390	-1.0937130	2.0806480
P	1.7078170	0.2330410	0.1844390	P	1.7493420	-0.4130520	0.4630130
N	2.7575960	1.5530340	0.4419370	N	2.6165200	0.8925980	0.8178780
P	4.3319610	1.5126210	0.2785350	P	4.0075600	1.3683480	0.1837390
C	5.0074330	3.1546000	0.7046890	C	4.5066420	2.8957440	1.0324960
C	4.5251790	4.2942190	-0.1956640	C	3.3988550	3.9428210	1.1871210
C	4.9904100	5.6598340	0.3057820	C	3.8538740	5.1202300	2.0452720
P	-1.7020550	-0.0191790	0.3801300	P	-1.8445620	-0.0116670	0.5760210
N	-3.3858550	-0.3850440	0.4032230	N	-3.3994470	0.0286640	0.1636150
P	-4.0400750	-1.7136550	-0.1426770	P	-4.3616970	-1.0807680	-0.4758830
C	-5.8433910	-1.4773130	-0.3069620	C	-5.9802240	-0.3076520	-0.7475170
C	-6.2807590	-0.3574220	-1.2542080	C	-5.9532710	0.9768530	-1.5821940
C	-7.7693760	-0.0442040	-1.1184160	C	-7.3121760	1.6731250	-1.5829490
N	-1.6791890	1.6741560	0.4958430	N	-1.1233510	1.4166110	0.5763850
P	-2.7616800	2.6436190	-0.1165840	P	-1.6479140	2.8173080	-0.0030970
C	-3.2534970	2.3449410	-1.8583180	C	-2.1038320	2.7984030	-1.7660790
C	-2.0887820	2.3703460	-2.8494430	C	-0.9457090	2.4932200	-2.7184260
C	-2.5540250	2.1096680	-4.2806150	C	-1.4049020	2.4797340	-4.1739580
C	-4.3777980	2.7015580	0.7468960	C	-3.1282890	3.4364820	0.8538150
C	-4.2682510	3.0931810	2.2209530	C	-2.9701460	3.5385450	2.3731210
C	-5.5978850	2.9339310	2.9554710	C	-4.2749500	3.9556410	3.0474840
C	-2.0878010	4.3391270	-0.0528430	C	-0.2883310	3.9950440	0.2275000
C	-2.9989330	5.4506770	-0.5785140	C	-0.5529650	5.4141470	-0.2822350
C	-2.3397000	6.8245370	-0.4693360	C	0.6520380	6.3236280	-0.0533770
N	2.7371520	-1.0083710	-0.3774050	N	2.5426490	-1.5628380	-0.3172520
P	2.6396290	-2.1392890	-1.4617460	P	2.4932460	-2.7151180	-1.4036490
C	4.3234630	-2.7542170	-1.8281160	C	4.1358250	-3.4857880	-1.4830680
C	5.1268590	-3.1530770	-0.5886270	C	4.6382000	-4.0175680	-0.1370100
C	6.5948510	-3.4206150	-0.9118940	C	6.0528650	-4.5813810	-0.2467920
C	1.7232090	-3.6424480	-0.9459300	C	1.3139210	-4.0563020	-1.0282480
C	0.2047070	-3.4770130	-0.8384460	C	-0.1611900	-3.6437140	-1.0246960
C	-0.4358180	-4.6387520	-0.0823160	C	-1.0581160	-4.7383890	-0.4527660
C	2.0126200	-1.7062630	-3.1362230	C	2.1416950	-2.1377680	-3.0993160
C	0.7500830	-0.8414400	-3.1815470	C	1.0930400	-1.0271350	-3.2122370
C	0.4228230	-0.3937740	-4.6027320	C	0.8462530	-0.6505070	-4.6698070
C	-3.4192730	-2.3855280	-1.7384900	C	-3.7333710	-1.7895090	-2.0393660
C	-3.1378610	-1.3501300	-2.8294090	C	-3.0316480	-0.8046780	-2.9798070
C	-2.6768330	-2.0124770	-4.1254750	C	-2.5523170	-1.5012160	-4.2500380
C	-3.8865080	-3.1411800	0.9999040	C	-4.6323370	-2.5065390	0.6251070
C	-4.4257690	-4.4996030	0.5440830	C	-5.5587920	-3.6067460	0.0941250
C	-4.1661740	-5.5850320	1.5879000	C	-5.6421880	-4.7775660	1.0716400
C	5.2460140	0.3486920	1.3687960	C	5.3480260	0.1600900	0.4309830
C	6.7335550	0.1240230	1.0832300	C	6.7226440	0.5432240	-0.1259150
C	7.3395810	-0.9059200	2.0356990	C	7.7370200	-0.5770440	0.0968840
C	4.9643000	1.1351160	-1.4111100	C	3.9394050	1.6571650	-1.6187320
C	4.0067800	1.5161510	-2.5407070	C	2.6967090	2.4066060	-2.1073060
C	4.5644600	1.1408500	-3.9117380	C	2.6470970	2.4776290	-3.6308450
H	0.4647560	-0.5135710	6.2880140	H	-0.0590730	-1.5743530	6.3125010
H	0.3828620	1.1542840	5.6631940	H	0.1060640	0.1516520	5.8993950
H	-1.0269180	-2.6522180	2.0406830	H	-1.6643850	-2.9689620	1.8058500
H	2.6698320	-0.4602590	2.1979040	H	2.2902030	-1.3681660	2.3668500
H	-2.3198200	-0.5536570	2.5697900	H	-2.6769360	-0.7922720	2.5593620
H	2.4877040	-0.0718850	4.6049390	H	2.0661270	-1.2193890	4.7471300
H	1.3711940	1.5522050	3.0182350	H	1.2469320	0.7210930	3.3361210
H	1.6827250	-2.4785890	4.4039070	H	0.9494310	-3.4503300	4.2384340
H	-0.9071590	-2.5387130	4.6138740	H	-1.6321520	-3.1746470	4.3678650
H	-1.7946450	-0.1718970	4.9169530	H	-2.1985510	-0.7599100	4.9484180
H	-1.0013130	1.4906440	3.1733760	H	-1.1099410	0.9734160	3.4565070
H	1.4247380	-2.5684840	1.8322800	H	0.7889410	-3.2257940	1.6887830
H	-1.1434320	4.3199010	-0.6080260	H	0.5845550	3.5522870	-0.2655980
H	-1.8242600	4.5225140	0.9950660	H	-0.0638970	3.9986800	1.2999630
H	-4.7621500	1.6794430	0.6619340	H	-3.9269650	2.7287230	0.6023880
H	-5.0591170	3.3731320	0.2101350	H	-3.3983380	4.4048240	0.4174260
H	-3.7326020	1.3597560	-1.8620370	H	-2.8988130	2.0498910	-1.8639970
H	-4.0192100	3.0780290	-2.1400270	H	-2.5507860	3.7703480	-2.0047050
H	-2.8148240	-3.2180050	1.2170370	H	-3.6381610	-2.9217290	0.8318620
H	-4.3628320	-2.8318880	1.9383390	H	-5.0071460	-2.1043840	1.5731980
H	-6.2936420	-2.4307380	-0.6066600	H	-6.6406380	-1.0545860	-1.2009990
H	-6.1964200	-1.2638830	0.7095620	H	-6.3710270	-0.0979790	0.2554310
H	-2.4958510	-2.9301360	-1.5079780	H	-3.0425290	-2.5957850	-1.7639450
H	-4.1435040	-3.1289750	-2.0931560	H	-4.5791920	-2.2657590	-2.5478500
H	1.8681760	-2.6388620	-3.6968140	H	1.8479490	-3.0137530	-3.6891390
H	2.8336580	-1.1690070	-3.6272400	H	3.0926710	-1.7824560	-3.5128620
H	1.9710650	-4.4595350	-1.6342530	H	1.4887370	-4.8594990	-1.7530870
H	2.1414520	-3.9102640	0.0322020	H	1.6010930	-4.4476100	-0.0455370
H	4.2464530	-3.5891110	-2.5347670	H	4.0998210	-4.2817890	-2.2358050
H	4.8352570	-1.9402910	-2.3562170	H	4.8149800	-2.7124850	-1.8623660

H	5.1435420	0.0561320	-1.4410510	H	3.9796380	0.6610530	-2.0786310
H	5.9352110	1.6307130	-1.5358710	H	4.8519480	2.1836160	-1.9186380
H	4.7127740	3.3428500	1.7439840	H	4.8625880	2.5804220	2.0206610
H	6.1021780	3.0903140	0.6872560	H	5.3685230	3.3117880	0.4991160
H	5.0979350	0.7032170	2.3966860	H	5.4007010	-0.0201590	1.5111230
H	4.6966720	-0.5933190	1.2730940	H	4.9863710	-0.7681250	-0.0256210
H	-1.5830390	3.3424270	-2.8048170	H	0.6467830	-0.0058650	-0.2947310
H	-1.3460590	1.6197990	-2.5528480	H	-0.1604190	3.2472980	-2.5924420
H	-3.2585530	5.2553710	-1.6255160	H	-0.4946270	1.5269620	-2.4592400
H	-3.9418160	5.4550910	-0.0193550	H	-0.7928350	5.3900340	-1.3513120
H	-3.5047350	2.4679690	2.6980990	H	-1.4276550	5.8338680	0.2267400
H	-3.9223300	4.1306220	2.3087580	H	-2.6447910	2.5695250	2.7664400
H	-4.0350950	-0.7541010	-3.0266700	H	-2.1819810	4.2592910	2.6187670
H	-2.3746350	-0.6522240	-2.4677140	H	-3.7101270	0.0122240	-3.2449050
H	-5.6949800	0.5430700	-1.0442490	H	-2.1795960	-0.3444390	-2.4662840
H	-6.0628170	-0.6435240	-2.2887590	H	-5.1916330	1.6528570	-1.1800010
H	-5.5013250	-4.4308310	0.3462650	H	-5.6639850	0.7438080	-2.6119720
H	-3.9544590	-4.7910790	-0.4015290	H	-6.5603380	-3.1984480	-0.0768250
H	-0.0453630	-2.5292620	-0.3440070	H	-5.1943420	-3.9688290	-0.8732690
H	-0.2196250	-3.4207020	-1.8481900	H	-0.2869510	-2.7324360	-0.4291500
H	-0.0975030	-1.3920240	-2.7630700	H	-0.4803900	-3.4002930	-2.0441250
H	0.8866810	0.0323060	-2.5365890	H	0.1480930	-1.3345670	-2.7491530
H	5.0534550	-2.3480410	0.1488000	H	1.4412490	-0.1497470	-2.6560290
H	4.6796810	-4.0416460	-0.1280860	H	4.6138720	-3.2067880	0.5988320
H	6.8642350	-0.2258900	0.0530890	H	3.9633240	-4.7983990	0.2306590
H	7.2849800	1.0680820	1.1640570	H	6.6468670	0.7523020	-1.1983970
H	4.8966220	4.1361970	-1.2149010	H	7.0788400	1.4623900	0.3519240
H	3.4319810	4.2665490	-0.2503250	H	3.0962340	4.3104650	0.2016350
H	3.0523860	1.0077310	-2.3716180	H	2.5183110	3.4688880	1.6318280
H	3.7907730	2.5895030	-2.5127700	H	1.7980480	1.9084670	-1.7245050
H	-8.0045570	0.2927050	-0.1033820	H	2.6844220	3.4211880	-1.6966850
H	-8.0702380	0.7444830	-1.8138520	H	-7.6079640	1.9522340	-0.5668260
H	-8.3793290	-0.9292560	-1.3271160	H	-7.2809480	2.5826650	-2.1884440
H	-3.4395420	-2.6981520	-4.5088980	H	-8.0887110	1.0199480	-1.9929480
H	-2.4706940	-1.2678200	-4.8995530	H	-3.3893810	-1.9630590	-4.7818420
H	-1.7589980	-2.5896360	-3.9668230	H	-2.0714300	-0.7914980	-4.9274590
H	-4.6464860	-5.3360470	2.5395690	H	-1.8274680	-2.2888890	-4.0169460
H	-4.5520060	-6.5531020	1.2563650	H	-6.0229460	-4.4501470	2.0439080
H	-3.0923770	-5.6945000	1.7743420	H	-6.3090010	-5.5551540	0.6904730
H	6.7011030	-4.2274740	-1.6444310	H	-4.6551060	-5.2241650	1.2284870
H	7.0682510	-2.5252910	-1.3298290	H	6.0908910	-5.4137940	-0.9563180
H	7.1500940	-3.7046110	-0.0131100	H	6.7549320	-3.8154750	-0.5916030
H	1.2378700	0.2080900	-5.0194270	H	6.4021300	-4.9463530	0.7226060
H	0.2663790	-1.2526590	-5.2640350	H	1.7774950	-0.3530970	-5.1620250
H	-0.4871320	0.2122890	-4.6200300	H	0.4291950	-1.4955260	-5.2255400
H	-0.0961610	-4.6581060	0.9590380	H	0.1438730	0.1827630	-4.7435580
H	-1.5271860	-4.5597200	-0.0775090	H	-0.7864830	-4.9646390	0.5836400
H	-0.1753070	-5.5993680	-0.5386160	H	-2.1092840	-4.4343060	-0.4664960
H	6.8235160	-1.8677780	1.9433080	H	-0.9701450	-5.6617840	-1.0322980
H	8.3992800	-1.0700660	1.8198810	H	7.4151340	-1.4950940	-0.4056830
H	7.2518110	-0.5788890	3.0768600	H	8.7186350	-0.3017320	-0.2975030
H	3.8483610	1.3699670	-4.7067660	H	7.8487400	-0.7993110	1.1626280
H	5.4914270	1.6833770	-4.1250200	H	1.7478900	2.9969150	-3.9730430
H	4.7929210	0.0699450	-3.9645450	H	3.5158160	3.0135230	-4.0247050
H	4.6444720	6.4610450	-0.3536450	H	2.6455530	1.4753570	-4.0696450
H	4.6036560	5.8593730	1.3105620	H	3.0566870	5.8625270	2.1399180
H	6.0833660	5.7100100	0.3527150	H	4.1274970	4.7881380	3.0517270
H	-3.2999740	2.8492790	-4.5899650	H	4.7251620	5.6154110	1.6053340
H	-1.7193330	2.1614140	-4.9859340	H	-1.8752480	3.4304490	-4.4423610
H	-3.0116350	1.1185710	-4.3691650	H	-0.5606530	2.3220040	-4.8506390
H	-1.4085740	6.8574880	-1.0442220	H	-2.1350150	1.6829820	-4.3490120
H	-2.9992220	7.6101550	-0.8487840	H	1.5286930	5.9578550	-0.5972640
H	-2.0978870	7.0596660	0.5722360	H	0.4443180	7.3403480	-0.3961990
H	-5.9411130	1.8947570	2.9128890	H	0.9087870	6.3698260	1.0096120
H	-5.5072130	3.2170450	4.0083150	H	-5.0686680	3.2304230	2.8418340
H	-6.3744430	3.5599820	2.5033460	H	-4.1492400	4.0217610	4.1313820
				H	-4.6096430	4.9326800	2.6851460
				H	-1.1639750	-0.8744950	-0.3155890
**I(Pd$_2$)$_2$**				**I(Pd$_2$)$_2$ (diprotonated)**			
C	1.4371260	1.4819080	-2.7393840	C	0.3117400	-0.0935790	-5.4763710
C	-0.0771280	1.5007570	-3.1524780	C	-0.8809630	0.3146750	-4.6170040
C	-0.9418780	0.3302680	-2.6769800	C	-0.6607300	-0.4262620	-3.2746310
C	-0.1223560	-0.8410950	-3.2352840	C	0.9152390	-0.4330480	-3.1299460
C	1.3963630	-0.8616800	-2.7935480	C	1.3568070	0.2945910	-4.4357620
C	1.8628400	0.2897780	-1.8825860	C	1.5205600	0.5299300	-2.0839240
C	0.3230880	1.0575190	-4.5993440	C	0.8999050	1.8543810	-2.5276490
C	-0.0276770	-0.4505330	-4.7356660	C	-0.6640090	1.8448700	-2.6263340
C	2.1271490	-0.4894370	-4.1171410	C	-1.3722670	0.5254250	-2.2958330
C	1.8196290	1.0310200	-4.1847110	C	0.9950800	1.7626310	-4.0807380
C	1.2683490	-1.0888640	-5.2267300	C	-0.5547700	1.7695380	-4.1865760
P	-1.7305210	0.1932650	-0.9859800	P	-1.6739000	-0.2416650	-0.6676950
N	-2.4827950	1.7321420	-0.9463150	N	-2.8889350	-1.2367280	-0.9113010
P	-2.0577800	2.8821140	0.0565210	P	-4.3299620	-1.5900260	-0.3439490
C	-3.1131360	4.3396620	-0.2291250	C	-5.0219050	-0.3015220	0.7426760
C	-2.9327500	4.9534610	-1.6199170	C	-6.3425240	-0.6403580	1.4372250
C	-3.9521250	6.0536050	-1.9289430	C	-6.8082610	0.5090080	2.3334400

C	-3.8563630	7.2627570	-0.9993410
P	1.6168120	0.3423830	-0.0162900
N	2.1646190	-1.2293470	0.3622640
P	1.2612030	-2.4109170	0.8937550
C	-0.1743200	-1.9561530	1.9215050
C	0.1520760	-1.1443960	3.1735050
C	-1.1107260	-0.8523180	3.9825190
C	-0.8844040	0.1166700	5.1377970
N	2.9136300	1.3659740	0.3725720
P	4.4265780	1.0181400	0.6818320
C	5.2833330	2.5639840	1.1400420
C	5.3215910	3.6045930	0.0172310
C	5.9007660	4.9505830	0.4633950
C	7.3742800	4.8906220	0.8641100
C	4.7031710	-0.1146110	2.0960590
C	3.9116970	0.2858930	3.3404730
C	4.0526000	-0.7151450	4.4863540
C	3.2336140	-0.3152660	5.7109570
C	5.4758190	0.3201170	-0.6631520
C	5.2550030	-1.1706460	-0.9537570
C	5.7735440	-1.5936680	-2.3299440
C	7.2788320	-1.4070870	-2.5134370
N	-2.9635580	-0.8488800	-1.5186690
P	-3.9178790	-1.7439750	-0.6393540
C	-5.6603940	-1.5908740	-1.2039680
C	-6.0843450	-0.1668890	-1.5729620
C	-5.9676600	0.8379020	-0.4282600
C	-6.4816890	2.2264380	-0.7968040
C	-3.5717070	-3.5535130	-0.7699130
C	-2.7871300	-3.9374180	-2.0276010
C	-3.5124570	-3.7144520	-3.3530630
C	-2.6332810	-4.0902930	-4.5446320
C	-3.8808060	-1.4491870	1.1762700
C	-5.0014970	-2.0892320	1.9981890
C	-4.6914600	-2.0789210	3.4957980
C	-5.8535350	-2.5874580	4.3439100
C	-2.2502350	2.4959880	1.8443960
C	-3.7172320	2.3978020	2.2726320
C	-3.9109580	1.8410410	3.6812670
C	-5.3844510	1.6324140	4.0221500
C	-0.3354210	3.4591460	-0.1368510
C	0.1480690	4.6455850	0.6954630
C	1.6172210	4.9569670	0.3992590
C	2.1653610	6.1086560	1.2370640
C	0.4900830	-3.4366690	-0.4120870
C	1.4963920	-4.2032490	-1.2733520
C	0.8992950	-4.6679920	-2.6019700
C	1.9235910	-5.3540040	-3.5010190
C	2.3028470	-3.5428560	1.8717230
C	1.5944220	-4.7549340	2.4773880
C	2.5555310	-5.6588380	3.2502380
C	1.8567940	-6.8659350	3.8708420
H	1.5636250	-0.7602270	-6.2295990
H	1.2420100	-2.1847610	-5.2054510
H	-0.5652330	2.4723080	-3.0575620
H	2.9607160	0.2580110	-1.9358760
H	-1.8546270	0.3826710	-3.2901470
H	3.1887150	-0.7490180	-4.1180460
H	1.6926950	-1.8394500	-2.4151630
H	2.5318490	1.6300640	-4.7562340
H	0.0260210	1.6868220	-5.4409630
H	-0.9363760	-0.6677310	-5.3026410
H	-0.6029050	-1.8114910	-3.0817420
H	1.8269320	2.4451120	-2.4006910
H	-2.9096550	-1.8328670	1.5064710
H	-3.8538270	-0.3675680	1.3413660
H	-5.9405000	-1.5510800	1.8192350
H	-5.1721780	-3.1257200	1.6781690
H	-3.7979570	-2.6887750	3.6785710
H	-4.4327470	-1.0587860	3.8032330
H	-6.7381010	-1.9553960	4.2110250
H	-6.1286900	-3.6096710	4.0630650
H	-5.5984700	-2.5881550	5.4074530
H	-2.9886220	-3.8292900	0.1166890
H	-4.5187770	-4.1033110	-0.7115110
H	-1.8591710	-3.3585650	-2.0442280
H	-2.4968870	-4.9927640	-1.9462430
H	-4.4429900	-4.2967920	-3.3717230
H	-3.7910930	-2.6578280	-3.4348110
H	-3.1576570	-3.9445430	-5.4934350
H	-1.7271600	-3.4735360	-4.5639840
H	-2.3189770	-5.1384530	-4.4898400
H	-5.7511730	-2.2435880	-2.0790370
H	-6.3133420	-2.0142610	-0.4319610
H	-5.4589410	0.1756150	-2.4054130
H	-7.1180570	-0.1973430	-1.9394050
H	-6.5031690	0.4627090	0.4545500

C	-8.1305000	0.2078040	3.0334930
P	1.7918460	0.2123180	-0.3021930
N	2.7778150	1.3644950	0.2256230
P	4.3798590	1.4512510	0.2144920
C	5.1511930	1.2346060	-1.4254360
C	5.2161410	-0.2219620	-1.9147900
C	5.4400520	-0.3236940	-3.4256670
C	6.7533040	0.2960370	-3.8990850
N	-1.7318800	0.7089600	0.6025760
P	-1.7631420	2.1473200	1.2720010
C	-0.3439170	3.1821320	0.7993420
C	-0.3637830	4.6270230	1.3060040
C	0.9456930	5.3409920	0.9676300
C	0.9664850	6.7935900	1.4330180
C	-3.2883030	3.0646590	0.9007610
C	-3.4935150	3.3305010	-0.5936020
C	-4.9153380	3.7886820	-0.9275020
C	-5.3246880	5.0845720	-0.2310380
C	-1.6910550	1.9436810	3.0743560
C	-2.7451430	0.9768970	3.6174270
C	-2.6646230	0.8137680	5.1343600
C	-3.5981760	-0.2820940	5.6410790
N	2.2946930	-1.2968020	-0.1228020
P	1.7854530	-2.6554490	0.5341850
C	3.2400810	-3.5360110	1.1629530
C	2.9612140	-4.8503130	1.8946730
C	4.2508410	-5.5009860	2.3974120
C	3.9923390	-6.8102550	3.1382480
C	0.6011360	-2.4690370	1.9063600
C	1.1512440	-1.7289130	3.1280410
C	0.1242620	-1.6682510	4.2589490
C	0.6687220	-0.9866620	5.5104350
C	0.9605370	-3.7193830	-0.6907090
C	1.8171060	-3.9544310	-1.9411960
C	0.9783280	-4.3338320	-3.1626160
C	1.8116860	-4.4014810	-4.4393550
C	4.8261370	3.1120670	0.7874570
C	4.3368070	4.2176470	-0.1567730
C	4.5240240	5.6227450	0.4222640
C	5.9848320	6.0142180	0.6384710
C	5.1707800	0.2357330	1.3138430
C	4.6426290	0.2705380	2.7491220
C	5.2830950	-0.8028490	3.6289480
C	4.7256810	-0.7942660	5.0493560
C	-5.4714140	-1.8437560	-1.7495890
C	-5.1549860	-0.9734540	-2.9716570
C	-5.3430180	0.5295660	-2.7635040
C	-5.0609910	1.3301310	-4.0320260
C	-4.3032160	-3.1217300	0.6513760
C	-3.2249590	-4.1274730	0.2302170
C	-3.3694440	-4.6818150	-1.1874750
C	-2.3553510	-5.7851270	-1.4782390
H	0.3948440	0.4846160	-6.4015530
H	0.3209800	-1.1609110	-5.7188400
H	-1.1550270	2.7314810	-2.2249880
H	2.5824330	0.5972930	-2.3649510
H	-2.4048390	0.6373680	-2.6548370
H	2.4026850	0.1235650	-4.6973370
H	1.3048870	-1.4479150	-3.0525200
H	1.5603660	2.5274350	-4.6128970
H	-1.0351350	2.5544960	-4.7702870
H	-1.8759470	0.1630380	-5.0398070
H	-1.0685680	-1.4399410	-3.2694250
H	1.3279040	2.7367780	-2.0492770
H	-4.2395490	-0.0957370	1.4804760
H	-5.1348260	0.6010760	0.1332550
H	-7.1189760	-0.8539980	0.6932070
H	-6.2295210	-1.5458060	2.0446680
H	-6.0336890	0.7136230	3.0834590
H	-6.9055700	1.4198600	1.7293020
H	-8.9268770	0.0251570	2.3047400
H	-8.0447190	-0.6820310	3.6656610
H	-8.4401100	1.0425580	3.6680910
H	-4.1313100	-2.8125830	1.6884390
H	-5.3026190	-3.5701660	0.6111220
H	-2.2413510	-3.6544320	0.3336940
H	-3.2502640	-4.9562390	0.9469740
H	-4.3862680	-5.0707340	-1.3274030
H	-3.2347400	-3.8709170	-1.9134180
H	-2.4510650	-6.1535850	-2.5032630
H	-1.3327740	-5.4163300	-1.3510650
H	-2.4904140	-6.6331320	-0.7990960
H	-5.4120790	-2.9014240	-2.0211220
H	-6.4910960	-1.6688630	-1.3884000
H	-4.1232970	-1.1702020	-3.2866170
H	-5.8031380	-1.3031660	-3.7912370
H	-6.3629200	0.7292810	-2.4122090

H	-4.9093900	0.9293960	-0.1637790
H	-6.3921780	2.9156470	0.0494220
H	-5.9022560	2.6426120	-1.6286770
H	-7.5343220	2.2008170	-1.0988730
H	-1.7372900	1.5362670	1.9916340
H	-1.7218610	3.2420800	2.4478890
H	-4.1872240	3.3871320	2.2134470
H	-4.2605720	1.7624400	1.5642360
H	-3.3810800	0.8858380	3.7647110
H	-3.4439770	2.5128970	4.4118260
H	-5.8499940	0.9352790	3.3152500
H	-5.5080940	1.2202860	5.0279480
H	-5.9393570	2.5751640	3.9721160
H	-2.9020240	5.0687870	0.5616690
H	-4.1497370	4.0093510	-0.0976080
H	-1.9194010	5.3660080	-1.7117270
H	-3.0188380	4.1569060	-2.3666780
H	-3.8059190	6.3816410	-2.9644700
H	-4.9631040	5.6291150	-1.8782790
H	-4.5442520	8.0539880	-1.3110150
H	-4.1043680	6.9996100	0.0336020
H	-2.8422470	7.6778160	-1.0032260
H	-0.2217410	3.6765850	-1.2052100
H	0.2854000	2.5745470	0.0561310
H	-0.4652870	5.5340000	0.4925000
H	0.0403750	4.4300750	1.7657970
H	2.2062150	4.0471360	0.5701510
H	1.7213510	5.1948060	-0.6677550
H	1.5838190	7.0239310	1.0806140
H	2.1282180	5.8696600	2.3054710
H	3.2060340	6.3261110	0.9788460
H	5.2453650	0.9179250	-1.5541760
H	6.5238980	0.5214980	-0.4105110
H	5.7571720	-1.7646420	-0.1790450
H	4.1880450	-1.4059300	-0.8739610
H	5.5142680	-2.6466960	-2.4938340
H	5.2385090	-1.0217750	-3.0997990
H	7.5635150	-0.3525780	-2.4403160
H	7.8356230	-1.9557020	-1.7455720
H	7.6074520	-1.7710220	-3.4913730
H	4.3768470	-1.1015060	1.7545820
H	5.7799710	-0.1594870	2.3020240
H	4.2304970	1.2763090	3.6911050
H	2.8545870	0.3772830	3.0663440
H	3.7343400	-1.7071090	4.1405540
H	5.1104800	-0.8133250	4.7606780
H	2.1731110	-0.2229920	5.4533870
H	3.3231190	-1.0534320	6.5131890
H	3.5634550	0.6520640	6.1045470
H	4.7495480	2.9717230	2.0064040
H	6.2930590	2.2999520	1.4740340
H	5.9143330	3.2204160	-0.8230240
H	4.3033850	3.7540260	-0.3565890
H	5.7780030	5.6708230	-0.3539430
H	5.3082200	5.3330650	1.3043570
H	7.7561150	5.8863270	1.1074460
H	7.5293870	4.2556760	1.7419070
H	7.9839490	4.4867720	0.0483370
H	-0.2461910	-4.1174540	0.0310190
H	-0.0703620	-2.7161510	-1.0199710
H	2.3679930	-3.5667540	-1.4745430
H	1.8739820	-5.0710630	-0.7173060
H	0.0558820	-5.3418820	-2.4068230
H	0.4796480	-3.7999620	-3.1257650
H	1.4723060	-5.6775790	-4.4434540
H	2.7473920	-4.6720440	-3.7393950
H	2.3517840	-6.2357180	-3.0123590
H	-0.7204700	-2.8728190	2.1779520
H	-0.8093990	-1.3621720	1.2496090
H	0.8812660	-1.6718820	3.8035840
H	0.6212840	-0.2007080	2.8702800
H	-1.8678650	-0.4429980	3.3022870
H	-1.5256300	-1.7953980	4.3600790
H	-1.8147910	0.3139210	5.6788440
H	-0.1597590	-0.2853520	5.8538110
H	-0.4999360	1.0750940	4.7721380
H	2.7604240	-2.9308050	2.6566140
H	3.1191950	-3.8584860	1.2115130
H	0.7967530	-4.4211100	3.1529740
H	1.1076050	-5.3408360	1.6870730
H	3.3508290	-5.9975160	2.5744550
H	3.0469520	-5.0704490	4.0353880
H	1.3813220	-7.4822140	3.1004190
H	2.5632670	-7.4974540	4.4169430
H	1.0772820	-6.5496350	4.5720590
H	-4.6712570	0.8807630	-1.9710290
H	-5.2099000	2.4006030	-3.8672850
H	-4.0280450	1.1845070	-4.3678450
H	-5.7210730	1.0188400	-4.8477970
H	-0.6828330	1.5794970	3.3048480
H	-1.7922730	2.9334460	3.5327700
H	-3.7507420	1.3269750	3.3532780
H	-2.6133900	0.0007940	3.1344360
H	-1.6322340	0.5802300	5.4188130
H	-2.9085440	1.7688650	5.6139180
H	-3.3287260	-1.2519970	5.2089730
H	-3.5497260	-0.3734100	6.7294700
H	-4.6377290	-0.0718590	5.3668700
H	-3.2678630	3.9957480	1.4761700
H	-4.1144700	2.4653330	1.2992430
H	-2.7751010	4.0869900	-0.9332500
H	-3.2790320	2.4177070	-1.1621850
H	-4.9866460	3.9184340	-2.0126530
H	-5.6202700	2.9889690	-0.6642770
H	-6.3121120	5.4112310	-0.5675710
H	-5.3712150	4.9620250	0.8553980
H	-4.6113760	5.8867350	-0.4485850
H	-0.2884700	3.1671660	-0.2937230
H	0.5528560	2.6630960	1.1594670
H	-1.2035750	5.1712150	0.8573770
H	-0.5138050	4.6517550	2.3918600
H	1.7746020	4.7907760	1.4297260
H	1.1106190	5.2952060	-0.1165470
H	0.1646510	7.3694370	0.9596900
H	0.8316490	6.8599340	2.5175480
H	1.9175210	7.2716640	1.1822610
H	4.5651160	1.8522930	-2.1174230
H	6.1521600	1.6770470	-1.3793800
H	6.0274140	-0.7392360	-1.3897920
H	4.2965020	-0.7590070	-1.6552680
H	5.4105120	-1.3817040	-3.7083630
H	4.6003840	0.1583020	-3.9436020
H	6.7803570	1.3744510	-3.7141810
H	7.6060990	-0.1541550	-3.3797780
H	6.8933440	0.1424610	-4.9725010
H	4.9825120	-0.7470500	0.8669300
H	6.2527370	0.4100960	1.2812150
H	4.8257780	1.2552210	3.1959030
H	3.5556990	0.1306490	2.7341400
H	5.1225490	-1.7895850	3.1758140
H	6.3690560	-0.6524560	3.6522850
H	3.6425500	-0.9539160	5.0429620
H	5.1786460	-1.5802380	5.6596320
H	4.9159640	0.1660480	5.5395740
H	4.3754380	3.2319800	1.7792690
H	5.9133780	3.1371540	0.9146210
H	4.8706290	4.1492990	-1.1124420
H	3.2753950	4.0537970	-0.3715420
H	4.0521330	6.3389870	-0.2599920
H	3.9790530	5.6972150	1.3717470
H	6.0643040	7.0491440	0.9823340
H	6.4672410	5.3811730	1.3897340
H	6.5554470	5.9236500	-0.2920420
H	0.6744580	-4.6605290	-0.2091910
H	0.0282900	-3.2052380	-0.9574000
H	2.3854060	-3.0440110	-2.1684980
H	2.5569860	-4.7378910	-1.7416450
H	0.4824510	-5.2951920	-2.9846420
H	0.1766860	-3.5940090	-3.2892230
H	1.1972230	-4.6826450	-5.2988470
H	2.2690220	-3.4295050	-4.6561280
H	2.6178850	-5.1362700	-4.3460240
H	0.2723640	-3.4787420	2.1799800
H	-0.2830650	-1.9505090	1.5184250
H	2.0592000	-2.2260790	3.4924040
H	1.4420220	-0.7097170	2.8449870
H	-0.7678090	-1.1389130	3.9019050
H	-0.1989700	-2.6867350	4.5050080
H	-0.0884540	-0.9445710	6.2985380
H	1.5307480	-1.5316700	5.9087640
H	0.9900400	0.0383620	5.2950140
H	3.7542660	-2.8256280	1.8195770
H	3.8980530	-3.6941250	0.3008730
H	2.2959830	-4.6711850	2.7484130
H	2.4392010	-5.5491260	1.2298280
H	4.9179440	-5.6806890	1.5458020
H	4.7719980	-4.7973000	3.0582490
H	3.4956450	-7.5375340	2.4876450
H	4.9269080	-7.2564750	3.4888640
H	3.3495890	-6.6475310	4.0096540
H	-0.5528100	-1.0734520	-0.4985230
H	0.6102870	0.4488500	0.4097080

I(Pe_2)$_2$				I(Pe_2)$_2$ (diprotonated)			
C	-4.2419380	3.1034670	-2.7498020	C	-4.8009060	2.7183680	-2.7780690
C	-3.5979050	2.0174100	-1.8647620	C	-3.8478980	1.8861850	-1.9041570
C	-3.6251240	2.5817420	-0.4183320	C	-3.9831840	2.4683690	-0.4677720
C	-4.4316160	3.8936020	-0.4833880	C	-5.0191410	3.6142530	-0.5561560
C	-4.1836670	4.3880650	-1.9121760	C	-4.9412940	4.0454510	-2.0253910
P	-4.1316720	1.3716250	0.8702180	P	-4.2962450	1.2247270	0.8366620
C	-3.8784490	2.3280550	2.4270430	C	-4.0982820	2.1425500	2.4039250
C	-2.4518390	2.8932140	2.5825320	C	-2.7191530	2.8086670	2.5705140
C	-2.1889140	2.9807660	4.1061100	C	-2.5011140	2.9393340	4.0969740
C	-3.4120390	2.3339980	4.7938760	C	-3.6426110	2.1429340	4.7724290
C	-4.0995190	1.5023310	3.7034340	C	-4.2674700	1.2808800	3.6659540
N	-3.4275660	-0.0422180	0.7853910	N	-3.3660230	-0.0639900	0.7072850
P	-1.7679480	-0.2680560	0.3818240	P	-1.8380790	-0.5061200	0.5427960
N	-1.7506920	-1.4375150	-0.8057470	N	-1.4582220	-1.3931180	-0.7268820
P	-2.2586710	-2.7767020	-1.4118390	P	-1.8380550	-2.7571150	-1.4661090
C	-0.9187210	-3.6550770	-2.3155300	C	-0.3483840	-3.5018500	-2.2109360
C	0.2040530	-4.1830530	-1.3936660	C	0.7633080	-3.7949490	-1.1790190
C	1.4447090	-4.1961440	-2.2919000	C	2.0289460	-3.8438690	-2.0368410
C	1.3080040	-2.8939810	-3.0858940	C	1.8410550	-2.6601900	-2.9875200
C	-0.2015020	-2.7659800	-3.3748200	C	0.3387830	-2.6735190	-3.3368860
C	-1.3273850	-1.2135550	1.9524150	C	-1.3528520	-1.2701620	2.1302540
C	-0.3837810	-2.4257450	2.1069460	C	-0.3422220	-2.4228950	2.3241570
C	-0.6268110	-2.7888560	3.6040030	C	-0.5831160	-2.7286750	3.8336030
C	-0.5569510	-1.3772430	4.2457530	C	-0.5922060	-1.2888530	4.4144280
C	-0.8125930	-0.3549710	3.1039230	C	-0.8868250	-0.3206490	3.2354430
C	0.6634450	-3.4590970	4.0703100	C	0.7354010	-3.3087090	4.3351990
C	1.5701180	-2.3334360	3.5791570	C	1.5877980	-2.1603780	3.8064570
C	0.9616520	-1.0502670	4.2069840	C	0.9071710	-0.8856790	4.3770740
C	1.1693400	-2.1223150	2.0962220	C	1.1927400	-2.0373180	2.3111550
C	1.5165240	-0.6436660	1.8835740	C	1.4765390	-0.5478140	2.0681860
C	0.7187910	-0.0269960	3.0410640	C	0.6250320	0.0833360	3.1761610
P	1.5250850	-0.0980750	0.0897620	P	1.6593130	0.0141290	0.3423510
N	2.9000480	-0.9290660	-0.4600530	N	2.9326390	-0.7185430	-0.3005910
P	4.4270070	-1.0296180	-0.0536270	P	4.5018960	-0.8043280	0.0084540
C	4.7389770	-2.2936440	1.2489290	C	4.8415140	-2.0210670	1.3300660
C	4.3891560	-3.7292160	0.8273840	C	4.4985790	-3.4743620	0.9614470
C	4.8407060	-4.5406030	2.0451510	C	5.0488930	-4.2416160	2.1658070
C	6.1532780	-3.8639050	2.4986800	C	6.3854040	-3.5429480	2.4861540
C	6.1313750	-2.4310690	1.8974300	C	6.2714390	-2.1046700	1.9100850
N	1.9740760	1.5331790	0.3054710	N	1.6151750	1.6195290	0.3771630
P	1.3918750	2.7079090	-0.5835220	P	1.0908830	2.7658470	-0.6068150
C	0.3521910	3.8651560	0.4032390	C	0.0110480	3.8853250	0.3467810
C	0.1762190	5.3163470	-0.0766770	C	-0.2281050	5.3013780	-0.2014980
C	-0.4764010	5.9917940	1.1325490	C	-0.9279390	5.9859000	0.9769280
C	0.2517990	5.3836110	2.3463100	C	-0.1911380	5.4569920	2.2245310
C	0.7947810	4.0083540	1.8786230	C	0.4559160	4.1098750	1.8096810
C	5.3092500	-1.5893420	-1.5624400	C	5.2545750	-1.3811280	-1.5455600
C	5.3264250	-0.5147610	-2.6847200	C	5.1673080	-0.3407440	-2.6900050
C	6.6448280	-0.7421290	-3.4636260	C	6.3409240	-0.6847150	-3.6437250
C	7.1903160	-2.0849040	-2.9589220	C	7.0052550	-1.9436210	-3.0598670
C	6.7542280	-2.1168810	-1.4910040	C	6.7151840	-1.8616380	-1.5575670
C	5.1833050	0.5517680	0.5120840	C	5.1790640	0.8235600	0.4941540
C	6.7036180	0.7571660	0.4298890	C	6.6927530	1.0715660	0.4048780
C	6.8938240	2.0657870	1.2032690	C	6.8356300	2.4181830	1.1215740
C	5.9377190	1.9406220	2.4031430	C	5.8522820	2.3425990	2.3057580
C	4.8104250	0.9750050	1.9527900	C	4.7911810	1.2771530	1.9218930
C	2.7531690	3.7192180	-1.2871980	C	2.4599750	3.7430790	-1.3039500
C	3.6723220	2.9186550	-2.2377390	C	3.3894360	2.9356950	-2.2363970
C	5.0960010	3.5064830	-2.0561620	C	4.7759440	3.6237510	-2.1329270
C	4.9450290	4.6625660	-1.0519430	C	4.6072910	4.7686950	-1.1156340
C	3.7168960	4.2769660	-0.2241200	C	3.4095280	4.3515380	-0.2577480
C	0.2716680	2.2001620	-1.9559870	C	0.0586140	2.1332170	-1.9821690
C	-0.3297340	3.3532830	-2.7950090	C	-0.3829380	3.1631670	-3.0469460
C	-0.5714020	2.7309030	-4.1739570	C	-0.6741720	2.2959670	-4.2767660
C	0.6618930	1.8466850	-4.3669510	C	0.4622490	1.2708320	-4.2617330
C	0.8579670	1.1919910	-2.9914050	C	0.6636880	0.9354470	-2.7735820
C	-5.9585840	1.1650370	0.8199620	C	-6.0436100	0.6849590	0.8266250
C	-6.5657090	0.9292630	-0.5820730	C	-6.6514870	0.4214180	-0.5724480
C	-7.7806560	0.0350880	-0.3212180	C	-7.6453550	-0.7198220	-0.3421110
C	-7.2635450	-0.9293630	0.7478750	C	-6.9107580	-1.6138140	0.6563710
C	-6.4331480	-0.0409650	1.6908090	C	-6.2579670	-0.6304120	1.6411930
C	-2.8919850	-4.0568660	-0.2439530	C	-2.4818440	-4.0713940	-0.3638970
C	-3.5865080	-5.2860980	-0.8723650	C	-3.0275170	-5.3227100	-1.0928550
C	-4.4939700	-5.7970320	0.2521800	C	-3.9712510	-5.9423730	-0.0577090
C	-5.0575590	-4.5050590	0.8539900	C	-4.6852710	-4.7233750	0.5322130
C	-3.8699820	-3.5236870	0.8460930	C	-3.5856780	-3.6508600	0.6532720
C	-3.5725280	-2.5250690	-2.6801430	C	-3.0440400	-2.4584790	-2.8073520
C	-4.9148350	-2.0913590	-2.0759840	C	-4.4513070	-2.1245430	-2.2862410
C	-5.6890870	-1.5510110	-3.2818760	C	-5.1572740	-1.5321730	-3.5130180
C	-4.6274260	-0.8427390	-4.1496810	C	-4.0536110	-0.8161890	-4.3263780
C	-3.2553060	-1.3974900	-3.6873660	C	-2.7043090	-1.2531050	-3.7099190
H	0.7031380	-3.6098830	5.1550510	H	0.7707820	-3.4057710	5.4244770
H	0.8609830	-4.4147700	3.5716900	H	0.9863900	-4.2726360	3.8817710
H	0.9699610	1.0169000	3.2420010	H	0.8224460	1.1443030	3.3314530
H	-2.3199880	-1.5756600	2.2512780	H	-2.3267700	-1.6657220	2.4500890

H	2.5646320	-0.5130910	2.1858560
H	-1.5602140	-3.3301450	3.7801970
H	-0.6280750	-3.2460010	1.4293210
H	-1.0938890	-1.2628630	5.1900180
H	1.4429950	-0.6988500	5.1221840
H	2.6452680	-2.4610180	3.7346630
H	1.6964940	-2.7886660	1.4096280
H	-1.4436130	0.4958000	3.3522130
H	7.3582800	0.0541480	-3.2245740
H	6.4969910	-0.7277960	-4.5464290
H	6.7166000	-2.9137330	-3.4983720
H	8.2721730	-2.1813430	-3.0850900
H	6.8170770	-3.1146050	-1.0484460
H	7.3977530	-1.4528230	-0.9064210
H	5.2999300	0.4983460	-2.2752630
H	4.4437330	-0.6169220	-3.3197280
H	6.5903210	2.9046590	0.5666640
H	7.0713990	0.8153670	-0.5992290
H	7.2413190	-0.0533280	0.9340040
H	6.2363120	-3.8436470	3.5883940
H	3.8217240	1.4384800	1.9419340
H	7.0197650	-4.4159520	2.1232080
H	5.5441300	2.9135640	2.7105020
H	6.9242170	-2.3294070	1.1532760
H	4.7643680	0.1143410	2.6279750
H	6.3017880	-1.6580990	2.6516890
H	4.9684840	-5.6038290	1.8253120
H	3.3304420	-3.8534530	0.5890740
H	4.0797370	-4.4568590	2.8298690
H	4.9675530	-4.0229250	-0.0573160
H	5.7704110	2.7429580	-1.6513990
H	5.5289530	3.8390010	-3.0028360
H	3.6589270	1.8613730	-1.9572230
H	0.3777630	4.1841120	-2.9025310
H	-1.2394840	3.7586340	-2.3438420
H	0.5468420	1.1100080	-5.1669770
H	-1.4745490	2.1074360	-4.1514470
H	-0.7022790	3.4803410	-4.9595700
H	1.5238930	2.4762600	-4.6201270
H	0.2828840	0.2643760	-2.9320900
H	1.8956260	0.9166260	-2.7962760
H	5.8398980	4.8179600	-0.4421300
H	3.9705260	3.4830080	0.4894480
H	4.7446430	5.6001040	-1.5832940
H	1.1513300	5.7769400	-0.2737390
H	-0.4222600	5.4046290	-0.9855280
H	-1.5423510	5.7352590	1.1558580
H	-0.4070250	7.0825310	1.0984580
H	1.0799830	6.0310440	2.6503670
H	0.4112470	3.1809540	2.4791920
H	1.8842230	3.9736520	1.9645070
H	3.2971590	5.1153430	0.3395910
H	-0.4115990	5.2877470	3.2101190
H	7.9316670	2.2388990	1.5007780
H	6.4695960	1.5236130	3.2640690
H	3.3257870	2.9826610	-3.2720570
H	-0.6359740	3.3865360	0.3939320
H	-0.5283780	1.6908880	-1.4023030
H	2.3103470	4.5615700	-1.8318980
H	4.0274970	-2.0173950	2.0360640
H	4.6757430	-2.4194730	-1.9001000
H	4.7071670	1.2787960	-0.1559570
H	-8.1349920	-0.4702480	-1.2246970
H	-8.6109200	0.6313600	0.0765940
H	-8.0603470	-1.4656440	1.2705040
H	-6.6127180	-1.6763040	0.2783820
H	-7.0494460	0.3257020	2.5171780
H	-5.5931110	-0.5934840	2.1163710
H	-5.8644930	0.3852270	-1.2175100
H	-6.8117820	1.8651920	-1.0912160
H	-2.0573470	4.0165500	4.4308570
H	-2.3530140	3.8637630	2.0895160
H	-1.7241930	2.2223940	2.1094710
H	-3.1856590	4.8391370	-1.9759690
H	-5.1578770	1.3230690	3.9124550
H	-4.9064930	5.1425900	-2.2344770
H	-4.0992770	3.1128370	5.1420280
H	-5.5029700	3.7072590	-0.3430960
H	-3.6145890	0.5257430	3.5919400
H	-4.1205690	4.6102640	0.2823250
H	-5.2887670	2.8474560	-2.9509820
H	-2.5647690	1.8182350	-2.1583150
H	-3.7402150	3.2045690	-3.7165490
H	-4.1157840	1.0617200	-1.9516990
H	-5.2679410	-6.4832470	-0.1035750
H	-3.8935840	-6.3282740	1.0008290
H	-4.2084540	-4.9993880	-1.7277000

H	2.5088010	-0.3696450	2.4052260
H	-1.4881980	-3.3088640	4.0230800
H	-0.5323590	-3.2811010	1.6784090
H	-1.1484800	-1.1593360	5.3427080
H	1.3602700	-0.4718580	5.2777180
H	2.6659910	-2.2245450	3.9706270
H	1.7584320	-2.7057280	1.6604830
H	-1.5604780	0.5056550	3.4461570
H	7.0567610	0.1422370	-3.6612350
H	6.0009690	-0.8396030	-4.6700990
H	6.5347640	-2.8453060	-3.4675760
H	8.0740240	-1.9997930	-3.2795260
H	6.8532200	-2.8171270	-1.0453060
H	7.3801080	-1.1291930	-1.0889110
H	5.2877430	0.6776230	-2.3100240
H	4.1916030	-0.3857790	-3.1787210
H	6.5410870	3.2201230	0.4362730
H	7.0575390	1.1000740	-0.6256510
H	7.2520100	0.2986070	0.9408770
H	6.5893260	-3.5333880	3.5591220
H	3.7724400	1.6732810	1.9398010
H	7.2137790	-4.0699880	2.0051020
H	5.3927150	3.3133280	2.5075280
H	7.0241480	-1.9550710	1.1329870
H	4.8321380	0.4421120	2.6281590
H	6.4374030	-1.3355250	2.6682730
H	5.1672560	-5.3090280	1.9660340
H	3.4311380	-3.6335800	0.7947040
H	4.3516800	-4.1378780	3.0050010
H	5.0283950	-3.7765810	0.0503110
H	5.5312120	2.9106910	-1.7844210
H	5.1180630	3.9907410	-3.1026150
H	3.4517750	1.8969360	-1.8968110
H	0.4265590	3.8623800	-3.2833870
H	-1.2447160	3.7525770	-2.7236960
H	0.2439710	0.3802160	-4.8573460
H	-1.6403620	1.7893350	-4.1596760
H	-0.7168180	2.8800780	-5.1989200
H	1.3712890	1.7233230	-4.6727530
H	0.1082630	0.0378760	-2.4946980
H	1.7129780	0.7369120	-2.5428140
H	5.5073480	4.9496790	-0.5227900
H	3.7013110	3.5867860	0.4724190
H	4.3676120	5.7017830	-1.6362300
H	0.7265540	5.7996120	-0.4046060
H	-0.8189100	5.3188620	-1.1198370
H	-1.9820980	5.6848540	0.9939880
H	-0.9044130	7.0755170	0.9022180
H	0.5833820	6.1631840	2.5352790
H	0.1447010	3.2761890	2.4420400
H	1.5451420	4.1643470	1.8837100
H	2.9661050	5.1872320	0.2897420
H	-0.8705330	5.3343480	3.0719160
H	7.8623720	2.6188040	1.4356830
H	6.3735320	2.0375460	3.2168080
H	3.0075220	2.9124380	-3.2597480
H	-0.9551590	3.3646510	0.3631250
H	-0.8403000	1.7719960	-1.4697540
H	2.0019360	4.5623400	-1.8706280
H	4.1619010	-1.7268110	2.1397770
H	4.6185770	-2.2342300	-1.8146390
H	4.6885730	1.5062370	-0.2094710
H	-7.9093760	-1.2344710	-1.2699750
H	-8.5684710	-0.3311430	0.1029660
H	-7.5602500	-2.3355840	1.1575270
H	-6.1344040	-2.1777080	0.1289400
H	-6.9261590	-0.4257030	2.4817710
H	-5.3262890	-1.0311550	2.0473000
H	-5.8850550	0.0842180	-1.2733530
H	-7.1118300	1.3165270	-0.9963040
H	-2.5218500	3.9873440	4.4052570
H	-2.6743040	3.7740880	2.0615120
H	-1.9351670	2.1799070	2.1311150
H	-4.0484810	4.6623250	-2.1828700
H	-5.3122490	1.0311440	3.8655890
H	-5.8106780	4.6284560	-2.3384270
H	-4.3974700	2.8320740	5.1628840
H	-6.0304900	3.2568600	-0.3353910
H	-3.7221700	0.3374030	3.5509920
H	-4.7957320	4.4217750	0.1452860
H	-5.7766340	2.2249090	-2.8455720
H	-2.8174910	2.0087410	-2.2448250
H	-4.4238330	2.8420760	-3.7964920
H	-4.0553110	0.8161710	-1.9540570
H	-4.6562530	-6.6713480	-0.4973920
H	-3.3883950	-6.4521180	0.7181480
H	-3.6036680	-5.0469550	-1.9828880

H	-4.7279020	-1.3036780	-1.3355290	H	-4.3683030	-1.3828440	-1.4819030
H	-5.4400530	-2.9052080	-1.5685910	H	-4.9752880	-2.9941030	-1.8812490
H	-4.7992810	-1.0255360	-5.2138520	H	-4.1076030	-1.0975890	-5.3806610
H	-6.1275690	-2.3904850	-3.8328390	H	-5.5945370	-2.3434040	-4.1026290
H	-6.5117610	-0.8886200	-2.9964680	H	-5.9764740	-0.8627950	-3.2359400
H	-4.6671460	0.2417090	-4.0065940	H	-4.1614850	0.2709750	-4.2845210
H	-2.6501590	-1.7586250	-4.5221240	H	-1.9670710	-1.5039490	-4.4740970
H	-2.6807310	-0.6154210	-3.1804510	H	-2.2857310	-0.4495400	-3.0935650
H	-5.8677290	-4.1302630	0.2167300	H	-5.4672690	-4.3906990	-0.1598620
H	-4.1707150	-2.4847230	0.6805380	H	-3.9726320	-2.6418390	0.4877960
H	-5.4761590	-4.6453140	1.8544630	H	-5.1683840	-4.9266660	1.4907150
H	0.3699430	-3.4883760	-0.5625400	H	0.8436550	-2.9623120	-0.4702720
H	-0.0337340	-5.1626530	-0.9683940	H	0.5842110	-4.7127640	-0.6127480
H	1.4107620	-5.0633950	-2.9633710	H	2.0672510	-4.7877620	-2.5930810
H	2.3789790	-4.2497610	-1.7232470	H	2.9401590	-3.7697240	-1.4388570
H	1.6422870	-2.0613670	-2.4573020	H	2.0945560	-1.7407450	-2.4481210
H	-0.4472090	-3.1185480	-4.3811220	H	0.1533400	-3.1498000	-4.3028480
H	-0.5178010	-1.7224860	-3.3093340	H	-0.0503930	-1.6584470	-3.4068180
H	-3.3603490	-3.5389730	1.8142180	H	-3.1604350	-3.6596810	1.6607960
H	1.9093060	-2.8865540	-4.0003190	H	2.4746250	-2.7160070	-3.8767270
H	-1.2662070	2.4515600	4.3644270	H	-1.5187950	2.5498030	4.3800120
H	-3.1347640	1.7330970	5.6645590	H	-3.2896630	1.5379790	5.6111560
H	-2.8683460	-6.0315360	-1.2258200	H	-2.2281750	-5.9962620	-1.4109880
H	-1.3946340	-4.5071590	-2.8166050	H	-0.6975160	-4.4522240	-2.6318880
H	-3.7058360	-3.4694310	-3.2229870	H	-3.0820590	-3.3712340	-3.4143270
H	-1.9847040	-4.4095250	0.2588440	H	-1.5961730	-4.3805110	0.2013410
H	-2.5938300	2.8288820	-0.1405610	H	-3.0227380	2.9140520	-0.1849210
H	-6.3787370	2.0898820	1.2299040	H	-6.5960330	1.5000900	1.3053130
H	-4.5941040	3.1608720	2.4122170	H	-4.8733680	2.9198170	2.3946910
				H	0.6085710	-0.5156410	-0.4123680
				H	-1.0186450	0.6349180	0.4412570
**I(Pf₂)₂**				**I(Pf₂)₂ (diprotonated)**			
C	1.0243800	0.8788920	-2.8141890	C	1.4307110	0.1716670	2.2275220
C	-0.5073150	1.1892170	-2.9609480	C	0.9476230	-1.1682760	2.8175170
C	-1.4684890	0.2438170	-2.2329910	C	1.0763090	-0.8581380	4.3358950
C	-1.0121120	-1.0954390	-2.8317430	C	0.4465210	0.5614560	4.3847950
C	0.5259010	-1.4071890	-2.6584100	C	0.4840280	1.1414510	2.9360980
C	1.3608230	-0.3309980	-1.9456780	C	-1.0725890	0.2729140	4.2373030
C	-0.4752210	0.6035400	-4.4108130	C	-1.1185940	-1.2742130	4.1167130
C	-1.1283540	-0.8044620	-4.3532230	C	0.0296890	-1.7438130	5.0040500
C	1.0561570	-1.2570710	-4.1158770	C	-0.5982900	-1.4582890	2.6631530
C	1.0393210	0.2896910	-4.2592450	C	-1.4247390	-0.3715750	1.9476510
C	-0.0943090	-1.7169570	-5.0077530	C	-1.0484130	0.8545720	2.7874290
P	-1.8273200	0.3559160	-0.3994390	P	-1.6096530	-0.3575800	0.1234920
N	-1.9446850	2.0451130	-0.3721030	N	-3.1222530	0.0062790	-0.2567850
P	-2.3752240	3.0859190	0.7224300	P	-4.1734190	1.1989240	-0.3682830
C	-2.2115500	2.4957520	2.4727740	C	-3.9396720	2.2331360	-1.8763510
C	-0.7905230	1.9899620	2.7443240	C	-4.1226930	1.4385960	-3.1719450
P	1.5136730	-0.1312080	-0.0800900	P	1.8407450	0.3292680	0.4569050
N	1.3809730	-1.7703270	0.3152360	N	3.3992060	-0.0372070	0.3109710
P	2.2583920	-2.7985300	1.1092120	P	4.2441660	-1.1696970	-0.4378880
C	4.0203630	-2.9081510	0.5500800	C	5.9700040	-0.9982030	0.1645450
C	4.8747820	-3.9454890	1.2827250	C	6.9088190	-2.0835780	-0.3721130
N	3.1921710	0.1849210	-0.0298870	N	1.3662480	1.7647170	-0.0452040
P	4.0635150	1.4824560	0.1219790	P	1.8495110	3.1816730	-0.5764010
C	4.4411310	2.2939870	-1.5073610	C	0.4609840	4.3820400	-0.3446030
C	4.8031130	1.2923900	-2.6086940	C	0.9133560	5.8456020	-0.2585780
C	3.3813200	2.8706920	1.1498750	C	3.3449120	3.8495300	0.2672610
C	2.2143510	3.5886450	0.4641880	C	3.1789450	3.7583040	1.7888280
C	5.6290000	0.9797050	0.9792720	C	2.2060460	3.0906750	-2.3797010
C	6.5051920	0.0963080	0.0832970	C	2.6443040	4.4153040	-3.0114570
C	2.9563160	2.3446000	2.5251970	C	3.5347330	-2.8483060	-0.1317110
N	-3.4568630	-0.1948680	-0.4447740	C	3.7694920	-3.3037810	1.3139050
P	-4.0445110	-1.5075240	0.2183840	C	4.3008500	-0.9567530	-2.2695060
C	-3.8736040	-1.7302430	2.0584270	C	2.8752450	-0.8572780	-2.8232440
C	-4.7137320	-0.7307770	2.8580840	C	4.6374930	3.1602240	-0.1834480
C	-5.8736890	-1.4569580	-0.0501410	C	1.0277970	2.4675560	-3.1381530
C	-6.2647490	-1.0577630	-1.4767640	C	-0.4295490	4.0108380	0.8438990
C	-3.3145090	-3.0731880	-0.4761260	C	3.9482860	-3.9330120	-1.1319940
C	-3.4700770	-4.3524020	0.3535860	C	5.1416930	0.2547460	-2.6851920
C	-6.6219530	-2.7114620	0.4109050	C	6.0668050	-0.8425630	1.6860490
C	-1.3185690	4.6076750	0.5829590	N	-1.1126900	-1.7820120	-0.4146830
C	-0.7374360	4.7700500	-0.8250390	P	-1.8230290	-3.0099550	-1.1491980
C	-4.1358590	3.6452740	0.5484560	C	-2.2473880	-2.6472690	-2.9202890
C	-5.1122870	2.5560740	1.0029370	C	-1.3047400	-1.5655500	-3.4677430
C	-1.9832270	5.9071950	1.0538770	C	-0.6191750	-4.4023870	-1.1615860
C	-4.4008830	4.0313180	-0.9104120	C	0.5183880	-4.1666900	-2.1591730
C	-2.6646770	3.4851710	3.5496870	C	-3.3849540	-3.5856550	-0.3471790
C	-3.7703070	-3.3085860	-1.9215980	C	-3.3651480	-3.5356810	1.1822430
C	-2.4037140	-1.6674750	2.4901370	C	-5.8202070	0.3935280	-0.5132820
C	2.3570070	-2.4975890	2.9406590	C	-6.9933630	1.3739380	-0.6024500
C	3.3089040	-1.3392770	3.2579560	C	-4.0724670	2.3700290	1.0573890
C	1.5358270	-4.4955600	0.8709670	C	-4.7618590	3.7245840	0.8493430
C	0.0523380	-4.4324160	0.5002180	C	-0.0566070	-4.6536060	0.2419630
C	0.9513270	-2.2111740	3.4786250	C	-3.8132000	-4.9669870	-0.8591780
C	1.7690630	-5.4661480	2.0352600	C	-3.7103430	-2.2309040	-3.1185580

C	4.0960080	-3.0936290	-0.9673290
C	6.4535160	2.0952320	1.6307590
C	5.4468070	3.4476990	-1.4496800
H	0.0669470	-1.4992670	-6.0698040
H	-0.3279610	-2.7817010	-4.8955780
H	-0.7699090	2.2417010	-2.8526460
H	2.4032350	-0.5932680	-2.1735170
H	-2.4657840	0.4551200	-2.6505630
H	2.0304910	-1.7254050	-4.2788990
H	0.6894030	-2.4014810	-2.2376220
H	1.7360530	0.7013910	-4.9929670
H	-0.7959540	1.2364810	-5.2412290
H	-2.1516880	-0.8571260	-4.7342180
H	-1.6168160	-1.9479540	-2.5195750
H	1.6471120	1.7600730	-2.6356890
H	-2.2466500	-2.8145660	-0.4969560
H	-4.2533930	-2.7358940	2.2713090
H	-0.4822340	4.3920380	1.2592860
H	-2.8807620	1.6267610	2.4949670
H	-6.1673510	-0.6231290	0.5996520
H	-0.2976490	3.8361300	-1.1789460
H	-1.5086630	5.0703630	-1.5415240
H	0.0334970	5.5477520	-0.8155590
H	-1.2389820	6.7095910	1.0712350
H	-2.7806800	6.2157300	0.3707590
H	-2.4097790	5.8305170	2.0564980
H	-4.2668100	4.5302180	1.1814010
H	-6.1370730	2.8458610	0.7477580
H	-4.8834920	1.6057780	0.5059850
H	-5.0770200	2.4029670	2.0855930
H	-5.4457970	4.3327990	-1.0344010
H	-3.7710780	4.8616320	-1.2427170
H	-4.2020440	3.1776840	-1.5641400
H	-0.0723960	2.8168800	2.7734110
H	-0.7557160	1.4947210	3.7210190
H	-0.4593000	1.2780510	1.9823520
H	-2.6793540	2.9859510	4.5238840
H	-1.9697260	4.3272400	3.6270850
H	-3.6665280	3.8839930	3.3667100
H	-3.1008840	-4.0244170	-2.4094560
H	-4.7798320	-3.7304620	-1.9496330
H	-3.7678790	-2.3878360	-2.5100920
H	-4.5195830	-4.6209920	0.5028080
H	-2.9890960	-5.1823890	-0.1745230
H	-2.9950270	-4.2745540	1.3339430
H	-7.7003850	-2.5306560	0.3681490
H	-6.4088160	-3.5639200	-0.2410700
H	-6.3761450	-2.9985640	1.4372570
H	-7.3221630	-0.7748000	-1.4969100
H	-5.6665350	-0.2110520	-1.8187710
H	-6.1302110	-1.8836540	-2.1798220
H	-2.3070400	-1.9921250	3.5314400
H	-1.7516770	-2.2951970	1.8767870
H	-2.0179410	-0.6478570	2.4084590
H	-5.7869800	-0.9118010	2.7564430
H	-4.4653170	-0.8082060	3.9213180
H	-4.5132760	0.2956570	2.5418090
H	5.2271640	0.3425960	1.7768710
H	3.4614040	2.7103570	-1.7737770
H	2.7391420	-3.4093750	3.4130410
H	4.3896250	-1.8997750	0.7663980
H	4.1972530	3.5904140	1.2856460
H	3.0714980	-0.4642630	2.6469050
H	3.2199090	-1.0582750	4.3125170
H	4.3538150	-1.6037250	3.0714360
H	0.2613760	-3.0408140	3.2971420
H	0.9892710	-2.0365590	4.5586910
H	0.5307300	-1.3222080	2.9983960
H	2.0780680	-4.8805470	-0.0023110
H	-0.1206920	-3.7033580	-0.2943030
H	-0.2924090	-5.4181030	0.1710420
H	-0.5554710	-4.1400620	1.3628240
H	1.2003440	-5.1665530	2.9212520
H	1.4278480	-6.4661620	1.7496400
H	2.8208950	-5.5444560	2.3198900
H	4.8209610	-3.8479900	2.3711170
H	4.5769780	-4.9653980	1.0173850
H	5.9242970	-3.8282160	0.9928840
H	5.1343500	-2.9893240	-1.3008130
H	3.7529360	-4.0882890	-1.2744010
H	3.4918200	-2.3429310	-1.4801370
H	5.8919690	2.6378030	2.3950250
H	7.3284130	1.6545990	2.1196900
H	6.8184480	2.8204710	0.8991820
H	5.9106740	-0.6457240	-0.4554520
H	7.0540030	0.6979290	-0.6481350
H	7.2436180	-0.4325900	0.6941150

C	-6.0493990	-0.6801880	0.5572320
C	-4.5210590	1.7227500	2.3735640
C	-2.5645400	2.9105500	-1.8466440
H	-0.1206820	-1.5062060	6.0617240
H	0.2416700	-2.8129000	4.9046890
H	0.7579270	2.1894320	2.8246650
H	-2.4700720	-0.5934340	2.2067670
H	2.4274570	0.3521440	2.6568740
H	-2.0985340	-1.7243380	4.2882800
H	-0.7732750	-2.4564560	2.2616550
H	-1.7630190	0.7115570	4.9579160
H	0.7812710	1.1958860	5.2054700
H	2.0998810	-0.9242770	4.7094860
H	1.5369270	-2.0319180	2.5052680
H	-1.6534880	1.7450160	2.6136220
H	2.4575100	-2.6679370	-0.2535650
H	4.7731890	-1.8604010	-2.6695460
H	-0.1328560	4.2605700	-1.2590140
H	3.0454450	2.3859940	-2.4249870
H	6.2642050	-0.0362420	-0.2729360
H	-0.7100690	2.9566520	0.8103460
H	0.0722720	4.1945880	1.7979940
H	-1.3341630	4.6268680	0.8265040
H	0.0314500	6.4912280	-0.2822950
H	1.4409370	6.0428640	0.6792170
H	1.5625610	6.1431900	-1.0845190
H	3.3940220	4.9060310	-0.0193440
H	5.4787540	3.5836170	0.3725480
H	4.5962720	2.0864970	0.0231100
H	4.8394420	3.3131140	-1.2470190
H	4.0396140	4.2249250	2.2750950
H	2.2794190	4.2657930	2.1467220
H	3.1362850	2.7145590	2.1143970
H	0.1931490	3.1701260	-3.2246510
H	1.3454410	2.2073010	-4.1514260
H	0.6611950	1.5593890	-2.6512940
H	2.9810330	4.2315700	-4.0353120
H	1.8117080	5.1228240	-3.0634280
H	3.4685080	4.8897740	-2.4719140
H	3.0885430	-4.1265450	1.5495580
H	4.7911460	-3.6696930	1.4484720
H	3.5960920	-2.5020410	2.0360060
H	5.0294300	-4.0919680	-1.1403180
H	3.4791310	-4.8772310	-0.8407480
H	3.6252790	-3.7074670	-2.1508830
H	7.9401600	-1.8224450	-0.1210800
H	6.6963280	-3.0546030	0.0840690
H	6.8513470	-2.1932990	-1.4588610
H	7.0695530	-0.4882140	1.9405690
H	5.3392400	-0.1164270	2.0552940
H	5.9090380	-1.7911830	2.2035180
H	2.9056450	-0.7945550	-3.9138380
H	2.2609560	-1.7220800	-2.5599210
H	2.3731940	0.0414000	-2.4531890
H	6.2081590	0.1002840	-2.5060390
H	5.0096720	0.4374200	-3.7550190
H	4.8337400	1.1571800	-2.1506920
H	-5.7287340	-0.1261200	-1.4751580
H	-2.9915150	2.5534500	1.1181340
H	-2.0733110	-3.5820890	-3.4666340
H	-4.1187510	-2.8437950	-0.6796540
H	-4.7145440	3.0067400	-1.8298010
H	-3.9702820	-1.3968100	-2.4611190
H	-3.8538950	-1.9021840	-4.1517690
H	-4.4087980	-3.0496400	-2.9330450
H	-0.2492900	-1.8179050	-3.3503090
H	-1.5033280	-1.4197670	-4.5330140
H	-1.4733370	-0.6129450	-2.9570650
H	-1.1870440	-5.2802650	-1.4893900
H	-0.8241840	-4.9715850	0.9501320
H	0.6976480	-5.4442570	0.1951670
H	0.4157590	-3.7469230	0.6327070
H	1.0609710	-3.2512380	-1.9088990
H	1.2233500	-5.0003660	-2.1058310
H	0.1716420	-4.0932250	-3.1919520
H	-3.7776380	-5.0480810	-1.9496650
H	-3.1813700	-5.7552050	-0.4389820
H	-4.8417440	-5.1654230	-0.5464780
H	-4.3618610	-3.7892430	1.5550480
H	-2.6597100	-4.2477170	1.6163110
H	-3.1190360	-2.5399090	1.5532480
H	-6.8489280	2.1406790	-1.3682480
H	-7.9016570	0.8211650	-0.8565620
H	-7.1703530	1.8695500	0.3561860
H	-5.1606920	-1.2989700	0.6985500
H	-6.3201610	-0.2399700	1.5194370
H	-6.8739770	-1.3258120	0.2431450

H	2.6119280	3.1744510	3.1510160
H	2.1328840	1.6324030	2.4200460
H	3.7733890	1.8447320	3.0541130
H	1.8031920	4.3444870	1.1422290
H	2.5162110	4.1056970	-0.4503320
H	1.4134000	2.8858010	0.2162000
H	5.2047440	4.1826890	-0.6767420
H	6.4606940	3.0793510	-1.2658230
H	5.4609530	3.9716560	-2.4106580
H	4.7344660	1.7837690	-3.5847600
H	5.8242780	0.9189810	-2.4966220
H	4.1243890	0.4375210	-2.6046490

H	-3.9838150	2.1096890	-4.0238200
H	-3.3800760	0.6412360	-3.2512080
H	-5.1181050	0.9966460	-3.2604890
H	-2.4437310	3.5302900	-2.7393860
H	-2.4352520	3.5556050	-0.9749960
H	-1.7603220	2.1677180	-1.8456390
H	-4.3545910	4.2801630	0.0021270
H	-5.8389930	3.6183020	0.7027800
H	-4.6094590	4.3340800	1.7444730
H	-4.1591370	2.3221160	3.2134360
H	-5.6119840	1.6879310	2.4380430
H	-4.1401920	0.7063350	2.4969420
H	1.0676140	-0.6339900	-0.2200200
H	-0.7378970	0.6260820	-0.3758470

I(Pg$_2$)$_2$

C	-0.8565020	-0.5981760	4.3486840
C	-1.2050760	0.9115380	4.2369340
C	-0.1507880	1.5910080	5.1061290
C	1.0312270	0.9270190	4.4048610
C	0.6935720	-0.5890230	4.4525730
C	0.8080160	1.2100260	2.8924700
C	-0.7651860	1.1931820	2.7688780
C	-0.7644530	-1.1483520	2.8938990
C	-1.3876700	-0.0255450	2.0595680
C	0.8044750	-1.1335250	2.9886820
C	1.5206340	0.0079000	2.2639070
P	1.8873280	-0.0340030	0.4390490
N	2.8427710	1.3757150	0.4614270
P	3.4370490	2.0921900	-0.7900110
N	5.0410970	1.6261540	-1.0499820
C	5.8317920	2.1106370	-2.1710220
P	-1.5379350	-0.3229070	0.2068030
N	-3.1751940	-0.8663600	0.2988580
P	-3.7680050	-1.8741860	-0.7437100
N	-3.1284470	-3.4344490	-0.6143730
C	-2.8646010	-3.9383240	0.7248780
N	-1.6180740	1.2120490	-0.4901940
P	-2.4925100	2.4953280	-0.3963890
N	-4.0292610	2.5338290	0.2943370
C	-5.1484270	1.9500420	-0.4390200
N	-1.7185150	3.6986090	0.5024570
C	-0.2654220	3.7640560	0.4822840
N	-2.8321390	3.0219800	-1.9651790
C	-2.0238700	2.5799610	-3.0869610
C	-2.3400380	4.9672660	0.8460030
N	-5.4430480	-1.8702130	-0.5452440
C	-6.2405030	-2.8325650	-1.2919310
N	-3.6187840	-1.6185270	-2.4076950
C	-2.2858230	-1.7512730	-2.9922790
C	-6.0039440	-1.5603500	0.7618330
C	-4.3508130	-0.4926910	-2.9833610
C	-3.4006230	-4.4991190	-1.5692810
C	-3.4332050	4.3298070	-2.1729910
C	-4.1412640	2.2876050	1.7281220
N	3.0122980	-1.3107310	0.4811010
P	2.9595040	-2.7341130	-0.1439140
N	3.1229170	-3.8814540	1.0841620
C	3.7920580	-3.5174670	2.3224210
N	1.6308110	-3.3481680	-0.9692400
C	1.3382210	-2.8876790	-2.3190900
N	4.1606140	-2.8805010	-1.3223910
C	5.2812170	-1.9554640	-1.3269860
C	0.4289170	-3.7475180	-0.2436250
N	2.8023380	1.8953850	-2.3380010
C	1.5030420	2.4918200	-2.6308630
N	3.2875080	3.7549210	-0.5680580
C	3.1153430	4.2987630	0.7684830
C	2.9895160	0.6197680	-3.0221080
C	3.9076230	4.6836840	-1.4985280
C	5.8497870	1.2820560	0.1105840
C	4.4634160	-4.1637610	-1.9347020
C	3.1364810	-5.3116460	0.8233520
H	-0.2306830	1.3292640	6.1674630
H	-0.1496750	2.6825170	5.0090680
H	-1.1887760	-2.1401860	2.7185020
H	2.5477980	0.0154750	2.6625150
H	-2.4502750	-0.0036120	2.3341650
H	2.0315030	1.1902620	4.7580450
H	1.2214650	2.1667000	2.5668390
H	1.1658090	-1.1513860	5.2610910
H	-1.4278200	-1.1583560	5.0920630
H	-2.2508810	1.1605950	4.4364440
H	-1.1383700	2.1437230	2.3864860
H	1.2758620	-2.1098090	2.8532770
H	0.1059870	4.4556430	-0.2899570
H	-1.5953920	1.6049860	-2.8598230

I(Pg$_2$)$_2$ (diprotonated)

C	-0.8582400	-0.9568100	4.3272970
C	-1.1714350	0.5638450	4.4008540
C	-0.0727580	1.1146840	5.3027620
C	1.0693000	0.5085220	4.4946150
C	0.6928350	-0.9946940	4.3826080
C	0.8081440	0.9738930	3.0337790
C	-0.7683630	1.0083470	2.9629690
C	-0.8212800	-1.3503880	2.8198990
C	-1.4481930	-0.1177200	2.1577320
C	0.7465070	-1.3828640	2.8675440
C	1.4756420	-0.1769860	2.2684030
P	1.9800580	-0.0262980	0.5336100
N	2.7376030	1.3911110	0.4846000
P	3.3694880	2.0543710	-0.8154820
N	4.8961080	1.4326410	-1.0906350
C	5.6818630	1.8662720	-2.2442170
P	-1.7337130	-0.1156880	0.3541620
N	-3.2225790	-0.7149080	0.2039640
P	-3.8525930	-1.7205240	-0.8495590
N	-3.2148270	-3.2532930	-0.6263140
C	-3.1481940	-3.7522770	0.7467340
N	-1.4140340	1.2848250	-0.3640220
P	-2.1576720	2.6929110	-0.2917250
N	-3.7485650	2.7231910	0.2053340
C	-4.7976430	2.3717850	-0.7477870
N	-1.4189820	3.7073600	0.7929000
C	0.0380820	3.7543860	0.8169650
N	-2.0667280	3.3403090	-1.8163400
C	-2.0651000	2.5421330	-3.0319770
C	-2.0488340	4.8785490	1.3956690
N	-5.4996230	-1.6348180	-0.6587710
C	-6.3231110	-2.6128010	-1.3670870
N	-3.6124300	-1.4674180	-2.4782810
C	-2.2536830	-1.5953690	-3.0011230
C	-6.1100290	-1.1368480	0.5686160
C	-4.4111200	-0.4714940	-3.1901630
C	-3.4013320	-4.3319300	-1.5966300
C	-2.3965690	4.7443180	-2.0304670
C	-4.0896580	2.3692710	1.5824760
N	2.8903160	-1.2772270	0.1396700
P	2.8103310	-2.7871310	-0.2964250
N	2.9435640	-3.7558350	1.0597250
C	3.7612770	-3.3042030	2.1824900
N	1.4503580	-3.3847910	-1.0387380
C	1.1752620	-3.0632890	-2.4350910
N	3.9975770	-3.0095200	-1.4429670
C	5.1275310	-2.0956310	-1.5518190
C	0.2655260	-3.7906690	-0.2902220
N	2.6048540	1.8469620	-2.2790290
C	1.3115730	2.5010300	-2.4856500
N	3.3108210	3.6991400	-0.5665060
C	3.3738590	4.2413790	0.7869480
C	2.7063890	0.5733960	-2.9918160
C	3.8404760	4.6045930	-1.5820400
C	5.7225040	1.0784130	0.0623280
C	4.2742170	-4.3514010	-1.9449220
C	2.8841280	-5.2128760	0.9619000
H	-0.1270390	0.7310320	6.3262530
H	-0.0458060	2.2083890	5.3318720
H	-1.2751780	-2.3030690	2.5462800
H	2.5010390	-0.2330700	2.6711250
H	-2.4980380	-0.1133310	2.4808040
H	2.0856620	0.7083980	4.8387880
H	1.2435480	1.9469170	2.8055410
H	1.1753940	-1.6569870	5.1013770
H	-1.4239420	-1.5866730	5.0136960
H	-2.2023970	0.8121890	4.6604910
H	-1.1261260	2.0047060	2.6999320
H	1.1852510	-2.3486610	2.6142590
H	0.4249310	4.5455360	0.1609250
H	-1.8042500	1.5120130	-2.7954860

H	0.0954500	4.1235390	1.4537680	H	0.3710520	3.9610370	1.8390990
H	-2.6527710	2.4982290	-3.9821490	H	-3.0430500	2.5642550	-3.5314700
H	-1.2074910	3.2842460	-3.3096340	H	-1.3214710	2.9406560	-3.7295520
H	-3.4250640	4.8659480	0.8675200	H	-3.1181590	4.8921530	1.1885490
H	-2.0034550	5.2770080	1.8431850	H	-1.8982530	4.8571330	2.4807500
H	-2.0737360	5.7654920	0.1379400	H	-1.6031900	5.8024390	1.0068370
H	-2.6800000	5.1278600	-2.2531430	H	-1.7506370	5.1454750	-2.8169580
H	-4.0133900	4.3185350	-3.1026170	H	-3.4421080	4.8767960	-2.3375380
H	-4.1139000	4.5715830	-1.3554430	H	-2.2231760	5.3267770	-1.1265300
H	-5.1553800	0.8565060	-0.3572120	H	-4.9662810	1.2879680	-0.7814580
H	-5.0928220	2.2229360	-1.4924770	H	-4.5412950	2.7229590	-1.7479770
H	-6.0823570	2.3455930	-0.0242980	H	-5.7274930	2.8623050	-0.4461320
H	-4.2090200	1.2123490	1.9399230	H	-4.3034610	1.2980490	1.6751710
H	-5.0427840	2.7805550	2.1092390	H	-4.9765020	2.9321360	1.8882840
H	-2.5049420	-5.1222810	-1.6838170	H	-2.5065560	-4.9630350	-1.6027740
H	-3.6585970	-4.0834750	-2.5428930	H	-3.5514420	-3.9309590	-2.5979940
H	-4.2237030	-5.1476050	-1.2366360	H	-4.2644350	-4.9575510	-1.3366960
H	-2.6076250	-3.1100230	1.3841420	H	-3.0519390	-2.9227510	1.4462790
H	-2.0215430	-4.6378110	0.6967030	H	-2.2775030	-4.4064700	0.8571970
H	-3.7330180	-4.4708330	1.1418350	H	-4.0467870	-4.3270870	1.0057010
H	-7.0013020	-1.1240770	0.6332180	H	-7.0449280	-0.6248600	0.3202310
H	-6.1033380	-2.4580230	1.3911890	H	-6.3338250	-1.9553370	1.2649610
H	-5.3608350	-0.8418740	1.2683670	H	-5.4392440	-0.4325390	1.0555750
H	-5.8166860	-2.9876520	-2.2852860	H	-5.8526630	-2.9028620	-2.3081110
H	-3.8538080	0.4676550	-2.7875760	H	-3.9670650	0.5277390	-3.1226540
H	-7.2567900	-2.4419910	-1.4121050	H	-7.2925800	-2.1623350	-1.5987500
H	-4.4183150	-0.6344450	-4.0671660	H	-4.4675830	-0.7555810	-4.2446200
H	-6.3088470	-3.8046950	-0.7810150	H	-6.4923380	-3.5129370	-0.7621570
H	-1.7437980	-2.5734380	-2.5245840	H	-1.7381240	-2.4282090	-2.5231200
H	-1.6926060	-0.8347970	-2.8643490	H	-1.6637120	-0.6809420	-2.8445550
H	-2.3834830	-1.9581990	-4.0635830	H	-2.2998200	-1.7948380	-4.0748340
H	-5.3593190	-0.4507440	-2.5722210	H	-5.4198890	-0.4316380	-2.7822310
H	-3.2773670	2.6939290	2.2552730	H	-3.2748770	2.6241190	2.2613550
H	0.1607180	2.7767070	0.3055080	H	0.4635210	2.7998520	0.5070120
H	0.6901770	-2.0006830	-2.3143790	H	0.5531740	-2.1630680	-2.5225340
H	6.1324630	-2.3287900	-0.7366840	H	5.9814730	-2.4296480	-0.9470350
H	2.2622930	-2.6447370	-2.8437370	H	2.1050810	-2.8961190	-2.9775510
H	5.6271140	-1.8096470	-2.3580490	H	5.4463200	-2.0489950	-2.5987170
H	4.9621800	-0.9952830	-0.9237510	H	4.8290190	-1.0989320	-1.2295790
H	-0.3330560	-2.9580060	-0.2618810	H	-0.5680480	-3.1009060	-0.4687460
H	0.0064100	-4.6485300	-0.7086080	H	-0.0520500	-4.7904410	-0.6091190
H	0.6739560	-3.9772700	0.7941130	H	0.4722190	-3.8158720	0.7796290
H	3.5626680	-4.7757140	-2.0001800	H	3.3704980	-4.9622720	-1.9256500
H	4.8348840	-4.0009670	-2.9530340	H	4.6116340	-4.2818450	-2.9836530
H	5.2313920	-4.7229460	-1.3802020	H	5.0555660	-4.8538510	-1.3601120
H	3.7188120	-2.4399950	2.4676650	H	3.7774850	-2.2162430	2.2228320
H	4.8532410	-3.8094260	2.3162880	H	4.7930210	-3.6707750	2.1018400
H	3.3071910	-4.0259050	3.1649670	H	3.3311130	-3.6841240	3.1148540
H	2.5671440	-5.5419630	-0.0776250	H	2.2774940	-5.5218090	0.1109180
H	2.6676350	-5.8379000	1.6635220	H	2.4248020	-5.6121620	1.8715830
H	6.6332140	0.5774230	-0.1920130	H	6.4667830	0.3411750	-0.2533600
H	5.2202800	0.8054480	0.8613860	H	5.1052410	0.6344380	0.8420030
H	6.3400590	2.1638250	0.5508740	H	6.2526530	1.9501570	0.4683870
H	5.1871320	2.3532530	-3.0160360	H	5.0283920	2.1451990	-3.0711400
H	6.5287280	1.3248420	-2.4870560	H	6.3159620	1.0364270	-2.5728980
H	6.4214430	3.0014930	-1.9110410	H	6.3268620	2.7196790	-1.9993370
H	2.9909930	0.7863680	-4.1055220	H	2.6663080	0.7616110	-4.0688080
H	3.9405940	0.1704660	-2.7368970	H	3.6433110	0.0716140	-2.7567420
H	2.1860660	-0.0885610	-2.7801070	H	1.8770680	-0.0969060	-2.7287300
H	1.4122410	2.6355380	-3.7137100	H	1.1597930	2.6452610	-3.5593950
H	3.9187220	4.2629500	-2.5050510	H	3.7289620	4.1703530	-2.5763950
H	1.4168740	3.4618630	-2.1407360	H	1.2922290	3.4742920	-1.9962810
H	3.3232380	5.6104240	-1.5268410	H	3.2731460	5.5400340	-1.5534110
H	0.6717310	1.8599880	-2.2922950	H	0.4880840	1.8949990	-2.0864730
H	4.0754530	4.5885600	1.2219060	H	4.4081260	4.4475860	1.0924870
H	2.6347480	3.5534050	1.4006910	H	2.9279130	3.5376700	1.4883110
H	2.4800110	5.1913730	0.7199510	H	2.8112280	5.1799240	0.8195830
H	4.9376900	4.9412880	-1.2109070	H	4.8996640	4.8364990	-1.4110240
H	4.1566620	-5.7048260	0.7060340	H	3.8845710	-5.6516590	0.8578180
H	0.8224960	-3.6841570	-2.8689920	H	0.6429960	-3.8997770	-2.8987110
				H	0.8692170	0.0278310	-0.3244420
				H	-0.8144940	-1.0167780	-0.2074340
**$I(Ph_2)_2$**				**$I(Ph_2)_2$ (diprotonated)**			
C	-4.8713320	-2.8998850	-0.9788570	C	4.7999010	-3.7078810	-1.1102430
N	-3.5528840	-3.4882080	-0.6782680	N	4.8682960	-2.9684080	0.1732920
C	-3.6581020	-4.4060430	0.4763400	C	4.2849060	-3.7846450	1.2607960
C	-5.0977210	-4.2488550	0.9724700	C	3.4689110	-4.8492510	0.5332970
C	-5.8568590	-3.8426670	-0.2913720	C	4.3130310	-5.1069680	-0.7165280
P	-2.2094120	-2.4794150	-0.7403830	P	4.5366290	-1.3431660	0.1245470
N	-0.9200700	-3.5244260	-0.5544030	N	5.0805980	-0.8391720	1.6077120
C	-0.7831460	-4.7528760	-1.3447820	C	6.2779520	-1.3913260	2.2706110
C	0.3262330	-5.5461850	-0.6385240	C	7.1569550	-0.1672680	2.6285400
C	0.3146700	-5.0030610	0.7945450	C	6.3643760	1.0612340	2.1444390
C	-0.0138160	-3.5235590	0.6001050	C	4.9262740	0.5484800	2.0605770
N	-2.2064460	-1.3594980	0.3388120	N	3.0234810	-0.9981930	-0.1631960
P	-1.6289310	0.2414800	0.3933900	P	1.7503070	-0.2785210	0.4678130

N	-2.9395000	1.2585050	0.1144430	C	1.6019550	-0.6931310	2.2279090
P	-4.4595500	1.4932190	0.2811880	C	0.9332440	-2.0100460	2.6173800
N	-5.2903800	0.8626960	-1.0241010	C	-0.6311570	-1.9689660	2.6838570
C	-4.6273880	0.4984970	-2.2878080	C	-1.3048950	-0.6250640	2.3826620
C	-5.7899460	0.1891170	-3.2481970	C	-0.5683450	0.2958420	3.3696970
C	-6.9947490	-0.0457950	-2.3299500	C	1.0093100	0.2579320	3.2802060
C	-6.7375460	0.9409450	-1.1938630	C	1.3981150	-0.5155150	4.5754920
C	-1.5503490	0.4078000	2.2533630	C	1.0017820	-1.9635240	4.1739650
C	-0.9335890	1.6924820	2.7971740	C	-0.8423000	-0.4698120	4.6963610
C	0.6352520	1.7012630	2.8561530	C	-0.5493440	-1.9289940	4.2457400
C	1.3626410	0.4284640	2.4066390	C	0.3374420	-0.1223500	5.5981300
C	0.6650770	-0.6026750	3.3066380	P	-1.8756680	-0.0818450	0.7431250
C	-0.9146020	-0.6150350	3.2120170	N	-2.6352990	-1.2901450	0.0368630
C	0.5607620	1.5082860	4.4089630	P	-2.5474490	-2.6537540	-0.7363750
C	0.9095530	0.0234100	4.7084540	N	-1.2056220	-3.0095670	-1.6346580
C	-1.3295320	-0.0057170	4.5862820	C	-0.8152410	-2.1037050	-2.7260620
C	-0.9912560	1.4905970	4.3410730	C	0.4270270	-2.7789600	-3.2928800
C	-0.2541900	-0.4628630	5.5677490	C	1.1282240	-3.3114420	-2.0387110
P	1.7951740	-0.0001210	0.6341850	C	-0.0150750	-3.6901920	-1.0790230
N	2.9363570	-1.1828820	1.0717610	N	1.5845270	1.3025140	0.3302060
P	4.0693280	-1.8375090	0.2293500	P	1.9556160	2.4627950	-0.6827910
N	4.3359650	-3.3766480	0.8753420	N	2.1353760	1.9105810	-2.2346960
C	4.2302740	-3.5727190	2.3234070	C	1.2695630	0.8122110	-2.7061930
C	5.6822030	-3.5471390	2.8458110	C	1.2705720	0.9509620	-4.2323260
C	6.5430420	-3.9449650	1.6215300	C	1.5339590	2.4417640	-4.4548210
C	5.5204820	-4.1448390	0.4831260	C	2.5657990	2.7457690	-3.3732830
N	2.7125750	1.3808010	0.2678870	N	3.3556210	3.3040240	-0.4209080
P	2.5218650	2.6477410	-0.6073230	C	4.6954600	2.8783310	-0.8579760
N	3.7952440	2.7556310	-1.6957700	C	5.6136110	3.9219990	-0.2237520
C	3.8880390	3.6674900	-2.8456350	C	4.9217590	4.2394480	1.1036130
C	5.3475840	3.5421640	-3.3102290	C	3.4303200	4.1940370	0.7591380
C	5.7805550	2.1731230	-2.7803250	N	0.7538950	3.5831860	-0.5125780
C	5.0857280	2.1224090	-1.4243100	C	0.7515550	4.8337220	-1.2882280
N	2.4022490	4.0077420	0.3991900	C	-0.3382240	5.6913320	-0.6294010
C	3.1969680	4.0303230	1.6310520	C	-0.4590890	5.1226170	0.7883820
C	4.4607030	4.8478540	1.2936820	C	-0.2111760	3.6274800	0.5991870
C	4.0396660	5.7339410	0.0957930	N	5.3984410	-0.7125190	-1.1301620
C	2.5642230	5.3555340	-0.1546150	C	4.8048540	-0.3343590	-2.4285150
N	1.2168810	2.9035490	-1.6230220	C	6.0223490	0.0181880	-3.2995390
C	0.9692140	1.9593770	-2.7200300	C	7.1505160	0.2859630	-2.2968710
C	-0.2576590	2.5566280	-3.4010940	C	6.8619280	-0.7283160	-1.1931960
C	-1.0934650	3.0642400	-2.2194180	N	-2.7736360	1.2063550	1.0537300
C	-0.0631110	3.4646630	-1.1456890	P	-3.9398970	1.9070060	0.2381260
N	-2.2703900	-1.9793440	-2.3392730	N	-3.7902340	2.2588790	-1.3754030
C	-1.3213900	-0.9413300	-2.7761600	C	-3.5913380	1.1616030	-2.3503330
C	-1.3033320	-1.0610520	-4.3014660	C	-2.5604720	1.6990480	-3.3656770
C	-1.6042470	-2.5420990	-4.5376330	C	-1.9500350	2.9304470	-2.6883070
C	-2.6635370	-2.8281630	-3.4748020	C	-3.1170800	3.4701950	-1.8684610
N	5.4451700	-0.8799050	0.2187420	N	-4.0919360	3.4173900	0.9300120
C	6.6194350	-1.0393420	-0.6489740	C	-3.9457470	3.5838530	2.3826080
C	7.6165170	-0.0067000	-0.1135920	C	-5.3868310	3.6334170	2.9188120
C	7.2761840	0.0660050	1.3762820	C	-6.2223400	4.1803530	1.7356110
C	5.7513450	-0.0325510	1.3835640	C	-5.2217230	4.2857410	0.5640950
N	3.9294000	-2.1951970	-1.4090200	N	-5.2800140	0.9299490	0.2792060
C	3.6365930	-1.0767110	-2.3337610	C	-6.5101860	1.1615110	-0.5013390
C	2.5494770	-1.6035660	-3.2949970	C	-7.4870220	0.1190500	0.0466790
C	1.9856760	-2.8456520	-2.5980630	C	-7.0645770	-0.0155530	1.5107290
C	3.2069630	-3.3908820	-1.8632000	C	-5.5402510	0.0476100	1.4355340
N	-5.1822470	0.9700860	1.7045550	N	-3.7673160	-2.6337840	-1.8600370
C	-6.4596380	1.4743680	2.2282670	C	-3.8864480	-3.5573270	-3.0069310
C	-7.3523860	0.2244250	2.4490130	C	-5.3639530	-3.4588580	-3.4137950
C	-6.5015740	-0.9630550	1.9647560	C	-5.7997410	-2.0914010	-2.8817450
C	-5.0685020	-0.4409370	2.0833730	C	-5.0647260	-2.0158890	-1.5498060
N	-4.8869970	3.1239070	0.3047130	N	-2.6083390	-3.9075970	0.3705320
C	-4.3244490	3.9404340	1.4005800	C	-3.4657560	-3.7924150	1.5625360
C	-3.4262540	4.9595840	0.7022960	C	-4.7263460	-4.6344370	1.2635840
C	-4.1954710	5.2290230	-0.5917470	C	-4.3337410	-5.5231870	0.0598930
C	-4.7106280	3.8386640	-0.9802500	C	-2.8258300	-5.2821750	-0.1096570
H	-0.3156880	0.0382830	6.5406030	H	0.3753640	-0.7279670	6.5089830
H	-0.2468490	-1.5469210	5.7272730	H	0.3726180	0.9367160	5.8716370
H	1.0922450	2.6214230	2.4910490	H	-1.1169290	-2.8293430	2.2263900
H	-2.6136960	0.4360230	2.5252100	H	2.6629950	-0.8058010	2.4898900
H	2.3847970	0.5354840	2.8025370	H	-2.3231510	-0.7311390	2.7945390
H	-2.3695340	-0.2050490	4.8584620	H	2.4432910	-0.3862830	4.8643940
H	-1.3000480	-1.6260570	3.0598000	H	1.4304230	1.2629430	3.2260310
H	-1.5506640	2.2036670	4.9507740	H	1.5340610	-2.7590700	4.6954630
H	1.0471010	2.2440620	5.0535290	H	-1.0652570	-2.7074230	4.8082470
H	1.9178470	-0.1474880	5.0941730	H	-1.8435120	-0.3005320	5.0970760
H	1.0797160	-1.6095300	3.2093130	H	-0.9474100	1.3193150	3.3709650
H	-1.3770570	2.6088070	2.3985800	H	1.3407380	-2.8961600	2.1256870
H	-5.5705750	-0.6671770	-3.8911010	H	5.8220340	0.8704260	-3.9525140
H	-5.9857960	1.0498830	-3.8960340	H	6.2878130	-0.8334130	-3.9331370
H	-7.9537890	0.1198380	-2.8263610	H	8.1454990	0.1628820	-2.7286470
H	-6.9832330	-1.0672760	-1.9342320	H	7.0738840	1.3003470	-1.8912140
H	-7.0668590	1.9540860	-1.4754690	H	7.2451590	-1.7232320	-1.4604780
H	-7.2575120	0.6664010	-0.2731310	H	7.2954200	-0.4442720	-0.2310170
H	-3.9812860	-0.3692080	-2.1376750	H	4.1274950	0.5116760	-2.2909840

H	-4.0001280	1.3130470	-2.6701110
H	-8.2994010	0.2995430	1.9079570
H	-6.8946000	2.1971210	1.5360170
H	-6.2890290	1.9954160	3.1784790
H	-5.0335800	5.9063740	-0.3951380
H	-4.3392480	-0.9603780	1.4537560
H	-3.5813730	5.6714200	-1.3799900
H	-6.7129240	-1.1888600	0.9139250
H	-3.9690190	3.3342620	-1.6108060
H	-4.7203120	-0.5153510	3.1244300
H	-5.6537350	3.8813920	-1.5346120
H	-2.4591480	4.4976300	0.4745670
H	-5.1364030	4.4417260	1.9435200
H	-3.2523570	5.8566400	1.3013820
H	-3.7823790	3.3149700	2.1148420
H	-5.4807950	-5.1596130	1.4381970
H	-5.1468740	-3.4414310	1.7098000
H	-3.4635910	-5.4363220	0.1513860
H	-0.3227080	-1.1279370	-2.3589570
H	-1.6389680	0.0479210	-2.4344440
H	-1.9530030	-2.7606690	-5.5495280
H	-2.0992650	-0.4447900	-4.7353150
H	-0.3522850	-0.7339160	-4.7291140
H	-0.7072730	-3.1418900	-4.3439610
H	-3.6557430	-2.5270430	-3.8414120
H	-2.7235260	-3.8795820	-3.1880890
H	-6.0433880	-4.7197050	-0.9205660
H	-5.0333170	-2.8382340	-2.0593980
H	-6.8161770	-3.3616380	-0.0850810
H	-1.7271930	-5.3121470	-1.3583350
H	-0.5163410	-4.5300780	-2.3847310
H	1.2900100	-5.3392890	-1.1116070
H	0.1555970	-6.6237000	-0.6927500
H	-0.4765440	-5.4899700	1.3755110
H	0.8953120	-2.9440040	0.3904770
H	-0.4975560	-3.0647770	1.4651080
H	-4.9589630	-1.8838020	-0.5685900
H	1.2649640	-5.1526210	1.3138540
H	-7.5929070	0.1161730	3.5102400
H	-6.6772840	-1.8713760	2.5469270
H	-2.9310770	-4.1637510	1.2575480
H	7.4417200	0.9662760	-0.5833070
H	8.6513620	-0.2953210	-0.3111440
H	7.7097260	-0.7922730	1.9029470
H	7.6382870	0.9772110	1.8581800
H	5.3644680	-0.4726650	2.3065790
H	5.2747160	0.9481320	1.2665140
H	6.3640790	-0.8595030	-1.6975920
H	7.0493000	-2.0463630	-0.5809960
H	5.8180980	-4.2250580	3.6921390
H	3.7821750	-4.5585760	2.5052930
H	3.5828940	-2.8134770	2.7614120
H	1.2113380	-2.5750250	-1.8725060
H	5.2346490	-5.2023370	0.4145810
H	1.5575440	-3.5682510	-3.2988390
H	7.1283510	-4.8511180	1.7956290
H	3.8438410	-3.9680940	-2.5479440
H	5.8811500	-3.8409500	-0.5008950
H	2.9637970	-4.0286260	-1.0133390
H	2.9963730	-1.8836130	-4.2540360
H	3.2889620	-0.1976470	-1.7825870
H	1.7880820	-0.8452250	-3.4947300
H	4.5384350	-0.7844970	-2.8844710
H	0.0520390	3.3870820	-4.0446650
H	-0.7893640	1.8269330	-4.0174430
H	1.8388020	1.8907560	-3.3785530
H	1.9034140	6.0345280	0.3994320
H	2.2635960	5.3920520	-1.2029560
H	5.2778080	4.1776980	1.0096280
H	4.6493020	5.5036240	-0.7828280
H	4.1495570	6.8014960	0.3016470
H	4.8013280	5.4350300	2.1502310
H	3.3988280	3.0129400	1.9655950
H	2.6124140	4.5414950	2.4074520
H	-1.7366320	3.9051370	-2.4944820
H	0.0275520	4.5511740	-1.0451900
H	-1.7326560	2.2716570	-1.8178280
H	3.1940460	3.3618120	-3.6375100
H	3.6467550	4.7030240	-2.5908010
H	5.9556810	4.3239520	-2.8417540
H	5.4449400	3.6454630	-4.3932360
H	5.3952090	1.3744010	-3.4241290
H	5.6704770	2.6926030	-0.6824580
H	4.9386600	1.1162940	-1.0316020
H	-0.3522450	3.0575210	-0.1706210
H	6.8654120	2.0626530	-2.7071910
H	5.9439330	-2.5409010	3.1867620
H	4.2236310	-1.1550090	-2.8626740
H	8.1398360	-0.2254510	2.1558320
H	6.7792740	-2.1041480	1.6148770
H	5.9708340	-1.9290140	3.1736970
H	5.1646890	-5.7471150	-0.4658360
H	4.2836620	1.1226580	1.3888350
H	3.7576770	-5.5864080	-1.5255160
H	6.6968200	1.3728260	1.1483940
H	4.0829550	-3.2401110	-1.7952670
H	4.4553500	0.5546050	3.0525170
H	5.7774520	-3.7231390	-1.5992790
H	2.4925530	-4.4376850	0.2538240
H	5.0903580	-4.2376730	1.8503010
H	3.3066850	-5.7428330	1.1390730
H	3.6859750	-3.1686790	1.9372370
H	5.2155390	5.2043860	1.5203930
H	5.1553670	3.4704120	1.8439180
H	3.0477510	5.1885550	0.5037220
H	0.2535470	0.9233650	-2.3050060
H	1.6693360	-0.1545580	-2.3834670
H	1.9001980	2.6700540	-5.4573120
H	2.0882640	0.3605760	-4.6587090
H	0.3364260	0.6021920	-4.6774060
H	0.6211560	3.0208390	-4.2780210
H	3.5628680	2.4294580	-3.7067250
H	2.6239570	3.7998400	-3.0968820
H	5.6499780	4.8148650	-0.8555940
H	4.7690730	2.8762510	-1.9473280
H	6.6334590	3.5509880	-0.0994180
H	1.7325380	5.3215170	-1.2401880
H	0.5256520	4.6414750	-2.3426890
H	-1.2812350	5.5749210	-1.1699640
H	-0.0804580	6.7518520	-0.6432190
H	0.3146550	5.5503910	1.4341590
H	-1.1346450	3.1009860	0.3369890
H	0.1982270	3.1422370	1.4871770
H	4.9403010	1.8676340	-0.5060080
H	-1.4335680	5.3198130	1.2415610
H	7.3157270	-0.1229950	3.7083850
H	6.4689630	1.9148090	2.8172620
H	2.8348100	3.8074190	1.5926960
H	-7.3544680	-0.8352740	-0.4726710
H	-8.5257590	0.4300430	-0.0789460
H	-7.4460370	0.8322200	2.0907130
H	-7.4168760	-0.9364130	1.9797110
H	-5.0879840	0.4408740	2.3494090
H	-5.1040220	-0.9402130	1.2467630
H	-6.3238940	1.0396560	-1.5714860
H	-6.9065900	2.1704560	-0.3409160
H	-5.4645400	4.2558260	3.8128250
H	-3.4368310	4.5387340	2.5609440
H	-3.3350450	2.7865380	2.8041630
H	-1.1318970	2.6497580	-2.0158570
H	-4.8414240	5.3096050	0.4746290
H	-1.5689370	3.6632370	-3.4041700
H	-6.6677330	5.1529080	1.9555540
H	-3.8094130	4.0390200	-2.5016210
H	-5.6396390	4.0072380	-0.4048460
H	-2.8202910	4.1053450	-1.0349390
H	-3.0619570	1.9916970	-4.2920930
H	-3.2288740	0.2597280	-1.8502970
H	-1.8174570	0.9390450	-3.6196010
H	-4.5361270	0.9088270	-2.8427090
H	0.1265200	-3.6009880	-3.9493610
H	1.0490030	-2.0901570	-3.8693530
H	-1.6201950	-1.9934770	-3.4546810
H	-2.2501430	-5.9586070	0.5322590
H	-2.4653200	-5.4101000	-1.1317660
H	-5.5760960	-3.9919350	1.0178690
H	-4.8654290	-5.2016410	-0.8408540
H	-4.5613230	-6.5790120	0.2191020
H	-5.0094150	-5.2277940	2.1356780
H	-3.6757200	-2.7465150	1.7869770
H	-2.9188570	-4.2160920	2.4124990
H	1.7718630	-4.1680360	-2.2520070
H	-0.1866580	-4.7699230	-1.0558410
H	1.7535180	-2.5321470	-1.5916890
H	-3.2312160	-3.2310650	-3.8215490
H	-3.6080340	-4.5830470	-2.7531580
H	-5.9418850	-4.2465370	-2.9189000
H	-5.4974540	-3.5707160	-4.4913180
H	-5.4510780	-1.2921670	-3.5446950
H	-5.6086920	-2.5920050	-0.7853290
H	-4.9228810	-1.0033070	-1.1721630
H	0.1949150	-3.3687930	-0.0525730
H	-6.8819460	-1.9984720	-2.7681090
H	-5.7197410	2.6264690	3.1888690

H	7.2442130	-3.1444240	1.3688540	H	-7.0388650	3.4957610	1.4898350
H	0.7576570	0.9492340	-2.3440780	H	-0.5770530	-1.1037940	-2.3365380
				H	0.6892750	-0.9258550	-0.1930630
				H	-0.8113710	0.3460290	-0.0651870
**II(Pa_2)$_2$**				**II(Pa_2)$_2$ (diprotonated)**			
C	0.6892220	3.2836950	-1.6249640	C	0.2694560	3.3507690	-1.9010360
C	1.1786890	3.5993210	-0.1987820	C	0.8319190	3.6569290	-0.4990850
C	0.6143060	4.8971780	0.3662590	C	0.2475690	4.9097480	0.1380910
C	-0.9236300	4.8231910	0.3968970	C	-1.2813800	4.7721100	0.2462080
C	-1.3909160	3.5029500	-0.2050110	C	-1.7253780	3.4445800	-0.3530880
C	-0.8660640	3.2507750	-1.6340630	C	-1.2788890	3.2392930	-1.8153840
C	-0.8577460	2.2212750	0.4993540	C	-1.0920490	2.1795870	0.2979820
C	0.7235000	2.3005660	0.5186690	C	0.4817230	2.3239090	0.2189510
C	0.7279060	1.7172510	-1.7587700	C	0.3767170	1.7940680	-2.0867710
C	1.3769300	1.2826740	-0.4359420	C	1.1314040	1.3789580	-0.8148290
C	-0.8340480	1.6937430	-1.7910720	C	-1.1812490	1.6898810	-2.0152380
C	-1.4596740	1.1891340	-0.4920130	C	-1.7129110	1.1514930	-0.6828010
P	-1.6320690	-0.6740770	-0.2526980	P	-1.8948060	-0.6546440	-0.4429520
N	-2.1570900	-0.6753960	1.3741270	N	-1.7519190	-1.0557410	1.0841440
P	-1.3957770	-1.2769160	2.6196440	P	-1.0880550	-1.3282390	2.4899360
C	-0.1882090	-0.1856250	3.4442030	C	-0.3359250	0.1278070	3.2581680
P	1.5874700	-0.4763470	0.1738140	P	1.6526160	-0.3007970	-0.3720160
N	2.3514550	-1.1361590	-1.2092420	N	2.4073330	-0.8543470	-1.6773060
P	1.6942740	-2.2368950	-2.1356550	P	2.6838490	-2.3657220	-2.1386030
C	2.9964450	-3.0597170	-3.0969200	C	4.3791670	-2.5170630	-2.7386380
N	2.8543590	-0.1831950	1.2782430	N	2.4275560	-0.2553580	1.0377560
P	4.4071430	-0.2311680	0.9799880	P	3.8610940	0.2942700	1.4888850
C	5.0814620	-1.8861530	0.6136240	C	5.2361130	-0.3903750	0.5300610
N	-3.0657340	-0.8800990	-1.1592870	N	-3.2801550	-1.0802760	-1.1461970
P	-4.5494670	-0.7509120	-0.6238700	P	-4.7264350	-1.1948080	-0.4654100
C	-5.0233340	0.7627720	0.2863530	C	-5.1931110	0.2125720	0.5812420
C	-5.7032080	-0.8229770	-2.0249760	C	-5.9599130	-1.3180790	-1.7750370
C	-5.0993890	-2.0905230	0.4863000	C	-4.9060420	-2.6645710	0.5763640
C	-2.6073890	-1.6423890	3.9253010	C	-2.3472820	-1.9260520	3.6407680
C	-0.4878770	-2.8362710	2.3637200	C	0.2185820	-2.5821630	2.4548370
C	5.0642910	0.7981490	-0.3802130	C	4.0331850	2.0980570	1.4024690
C	5.3171920	0.3200020	2.4513510	C	4.1029170	-0.1700140	3.2141070
C	0.7819030	-3.5723910	-1.2973190	C	2.4565180	-3.6090090	-0.8385760
C	0.5218560	-1.6264930	-3.3922880	C	1.5995070	-2.8388050	-3.5055750
H	1.2191420	1.3030050	-2.6417630	H	0.8274290	1.4228630	-3.0068920
H	-2.5232640	1.4496560	-0.5428690	H	-2.7853970	1.3839050	-0.6809550
H	2.4304510	1.5765890	-0.5319770	H	2.1534530	1.7600260	-0.9739840
H	-2.4865230	3.4791790	-0.1965520	H	-2.8148240	3.3697380	-0.2752970
H	-1.2550200	2.1008110	1.5107870	H	-1.4203040	2.0322500	1.3282330
H	-1.3634400	3.8418620	-2.4076240	H	-1.8522810	3.8113470	-2.5462250
H	1.1664620	3.8777010	-2.4090290	H	0.6700400	3.9815980	-2.6959270
H	2.2735030	3.6445530	-0.1729000	H	1.9219490	3.7555950	-0.5377480
H	1.1058830	2.2315200	1.5412920	H	0.9281760	2.2534260	1.2130740
H	-1.2742260	1.2733470	-2.6991950	H	-1.6569480	1.2644710	-2.8999010
H	0.9558540	5.7381870	-0.2484360	H	0.5244820	5.7788930	-0.4674130
H	1.0199160	5.0620430	1.3707350	H	0.7014870	5.0567950	1.1234120
H	-1.3689960	5.6539810	-0.1623780	H	-1.7889570	5.5870590	-0.2798600
H	-1.3002600	4.9028080	1.4230700	H	-1.6088700	4.8211970	1.2897980
H	-2.1100500	-2.0125340	4.8253080	H	-1.9154160	-2.0520430	4.6360160
H	-3.3102660	-2.3978240	3.5665280	H	-2.7303710	-2.8876140	3.2926530
H	-3.1602230	-0.7321970	4.1688220	H	-3.1649710	-1.2038610	3.6864000
H	-0.0618930	-3.1961700	3.3039820	H	0.4735440	-2.8701290	3.4777700
H	0.3109770	-2.6373040	1.6444250	H	1.1088280	-2.1647110	1.9757240
H	-1.1670150	-3.5904160	1.9584160	H	-0.1326290	-3.4596740	1.9082610
H	0.1509680	-0.6298100	4.3846060	H	0.0489670	-0.1401270	4.2455260
H	-0.6651890	0.7756310	3.6520490	H	-1.0796420	0.9199440	3.3638760
H	0.6717380	-0.0299890	2.7873060	H	0.4954420	0.4650860	2.6336180
H	-6.7371380	-0.7759910	-1.6735480	H	-6.9562670	-1.4299890	-1.3420210
H	-5.5513490	-1.7558860	-2.5722270	H	-5.7343660	-2.1855300	-2.3984300
H	-5.5120460	0.0171570	-2.6962560	H	-5.9271980	-0.4156480	-2.3884920
H	-4.4822260	-2.0500850	1.3855950	H	-4.1750030	-2.6065900	1.3846340
H	-6.1544760	-1.9796310	0.7505180	H	-5.9136370	-2.7200130	0.9945320
H	-4.9482010	-3.0526930	-0.0090570	H	-4.7070130	-3.5554740	-0.0226460
H	-6.0653080	0.7090870	0.6146560	H	-6.1897290	0.0564280	1.0003560
H	-4.3628840	0.8499100	1.1519810	H	-4.4704170	0.3076340	1.3957630
H	-4.8922460	1.6374930	-0.3557080	H	-5.1926810	1.1304180	-0.0111810
H	1.4339260	-4.0548750	-0.5650190	H	3.0910420	-3.3661590	0.0166870
H	0.4296320	-4.3145790	-2.0184360	H	2.7206310	-4.5986820	-1.2173800
H	-0.0705820	-3.1153190	-0.7857750	H	1.4116300	-3.6212630	-0.5194550
H	1.0158260	-0.8702900	-4.0073030	H	1.7237040	-2.1228340	-4.3206950
H	-0.3287770	-1.1719430	-2.8774880	H	0.5618250	-2.8161130	-3.1652220
H	0.1692350	-2.4411050	-4.0308710	H	1.8442340	-3.8422990	-3.8606870
H	3.5508110	-2.3118010	-3.6682170	H	4.5473180	-1.7708250	-3.5178080
H	2.5654270	-3.7915520	-3.7846000	H	4.5483170	-3.5154720	-3.1476070
H	3.6834130	-3.5659040	-2.4148530	H	5.0739860	-2.3394270	-1.9155460
H	4.5773950	0.4823740	-1.3053790	H	3.9903030	2.4277270	0.3616580
H	6.1479030	0.6801880	-0.4710520	H	4.9916210	2.4032570	1.8292080
H	4.8295260	1.8488250	-0.1917450	H	3.2231770	2.5680740	1.9645300
H	5.0507340	1.3549370	2.6770450	H	3.3073180	0.2748030	3.8155620
H	5.0468850	-0.3098080	3.3016490	H	4.0526330	-1.2571370	3.3000380
H	6.3949750	0.2536770	2.2824100	H	5.0725520	0.1825940	3.5716670

H	4.6066210	-2.2388060	-0.3040590	H	5.1030540	-0.1200010	-0.5200770
H	6.1670690	-1.8547760	0.4880810	H	6.1886110	0.0032690	0.8916770
H	4.8294800	-2.5654110	1.4316360	H	5.2332480	-1.4784410	0.6257210
				H	-0.9348530	-1.2799030	-1.2557410
				H	0.5652490	-1.1110730	-0.0447470
**II(Pb_2)$_2$**				**II(Pb_2)$_2$ (diprotonated)**			
C	-0.8621260	0.3740370	-2.8984310	C	0.9999230	2.2333470	-1.8783030
C	0.6191520	0.8030300	-2.6522240	C	-0.4326570	1.7602210	-2.2809150
C	1.4120750	-0.0431520	-1.6401650	C	-1.4001760	1.4339410	-1.1276640
C	1.2662180	-1.4265460	-2.3043190	C	-1.3688520	2.7699910	-0.3574280
C	-0.2505770	-1.8531760	-2.4776330	C	0.0949470	3.1755210	0.0926670
C	-1.2942160	-0.7949740	-2.0190940	C	1.2323310	2.2185270	-0.3681270
C	1.0167400	0.1549420	-4.0310950	C	-0.8131000	3.2552680	-2.5988780
C	1.8691400	-1.0794100	-3.6894040	C	-1.8272860	3.6842870	-1.5239030
C	-0.5776440	-1.8124320	-3.9985450	C	0.5315320	4.3918420	-0.7773180
C	-0.4564360	-0.2950920	-4.2496730	C	0.6066190	3.7156820	-2.1623060
C	0.3321140	-2.6560330	-4.8844510	C	-0.4088760	5.5890270	-0.7285370
C	1.7909990	-2.1849750	-4.7355140	C	-1.8064470	5.1774250	-1.2250580
P	1.3481160	-0.0348910	0.2398800	P	-1.5735040	-0.0939130	-0.1458340
N	3.0007600	-0.4934520	0.4117850	N	-3.1061060	-0.0357240	0.3176190
P	3.5443090	-1.4896520	1.5121150	P	-4.0461960	-0.3467050	1.5622180
C	2.5558810	-3.0003520	1.8800290	C	-3.5551110	0.4465090	3.1402120
C	1.9174240	-3.6698400	0.6630760	C	-2.7696740	1.7504830	3.0039310
P	-1.8052150	-0.3605560	-0.2759650	P	1.7666690	0.6283830	0.3252370
N	-3.3182300	0.3453240	-0.6990030	N	3.3209410	0.4735630	-0.0554260
P	-3.7143380	1.8198270	-0.2780520	P	4.2194470	-0.4083460	-1.0340330
C	-5.5297730	2.0022220	-0.3354340	C	5.9270610	0.1932440	-0.8985720
C	-6.1283450	1.6539010	-1.6988990	C	6.0699160	1.6773800	-1.2414880
N	-2.1933330	-1.9507920	0.2095140	N	1.4290200	0.6207430	1.8976680
P	-2.9145210	-2.2550760	1.5841760	P	2.1253270	-0.2616180	3.0484720
C	-4.7224340	-1.9337060	1.5652350	C	3.8554450	0.2220040	3.3515910
C	-5.5098130	-2.2948870	2.8255700	C	4.6218420	-0.6616630	4.3381060
C	-2.7735700	-4.0368470	1.9790550	C	1.2079330	0.0533040	4.5828950
C	-1.4053530	-4.6392610	1.6631210	C	-0.2388290	-0.4410080	4.5515140
C	-2.3285930	-1.3227420	3.0559410	C	2.1454010	-2.0616390	2.7210350
C	-0.8315460	-1.4527040	3.3377120	C	0.8983200	-2.6104040	2.0231410
N	1.2741820	1.6197450	0.5819480	N	-1.0712330	-1.3815610	-0.9628560
P	2.3383420	2.7817240	0.5031750	P	-1.7205030	-2.3139890	-2.0882150
C	1.4659620	4.3834820	0.6330900	C	-0.3706430	-3.2381050	-2.8751690
C	0.5547720	4.4854480	1.8547140	C	0.5650470	-3.9024720	-1.8654330
C	3.5664400	2.7644080	1.8662190	C	-2.9016060	-3.5077790	-1.3832300
C	4.4777540	3.9872970	1.9769010	C	-3.5026990	-4.5024620	-2.3777590
C	3.3495450	2.9922260	-1.0252840	C	-2.5902920	-1.4426880	-3.4353030
C	4.5753740	2.0817000	-1.1325860	C	-3.9674330	-0.8900280	-3.0546910
C	5.2179350	-2.0448720	1.0283910	C	-5.7122700	0.2371670	1.1373580
C	5.2993900	-2.7049870	-0.3478540	C	-5.7746770	1.7409100	0.8628290
C	3.8350140	-0.7732690	3.1791510	C	-4.2124950	-2.1204430	1.9482000
C	2.5722010	-0.1980500	3.8210100	C	-2.8727350	-2.8088480	2.2128530
C	-3.2223890	2.3207730	1.4184570	C	4.2365220	-2.1768600	-0.5867970
C	-3.5808110	3.7441950	1.8472210	C	5.0266990	-3.0910650	-1.5258030
C	-3.0760070	3.1601060	-1.3613900	C	3.7582970	-0.3068160	-2.7958930
C	-1.5905790	3.4555200	-1.1392580	C	2.4283540	-0.9843750	-3.1325490
H	-1.5823360	1.1929460	-2.9482560	H	1.8092450	1.8140990	-2.4765580
H	2.4586280	0.2564190	-1.7810700	H	-2.3893620	1.3810660	-1.6044420
H	-2.2600650	-1.1526640	-2.4027680	H	2.1651500	2.7673580	-0.1773850
H	2.9177480	-0.7939720	-3.5473700	H	-2.8422850	3.3919220	-1.8121110
H	1.8346140	-2.2077990	-1.7937050	H	-2.0603800	2.7997970	0.4857250
H	1.4446960	0.8052590	-4.7989090	H	-1.1074200	3.4908500	-3.6227380
H	-0.9428330	0.0493720	-5.1660740	H	1.1931510	4.2649510	-2.9000520
H	-1.6146120	-2.1339420	-4.1461510	H	1.5297380	4.7117610	-0.4619000
H	-0.4329420	-2.8364390	-2.0344970	H	0.1308410	3.3826520	1.1639600
H	0.7713780	1.8796860	-2.5468530	H	-0.4645960	1.0484210	-3.1069500
H	-0.0020860	-2.5779260	-5.9253300	H	0.0067630	6.3936690	-1.3435400
H	0.2295160	-3.7106110	-4.6040480	H	-0.4510790	5.9691350	0.2974250
H	2.1831940	-1.8107000	-5.6884370	H	-2.0803490	5.7257920	-2.1325300
H	2.4424640	-3.0135400	-4.4357780	H	-2.5731380	5.4056380	-0.4778130
H	3.2190510	-3.6910180	2.4136960	H	-4.4796740	0.6029290	3.7062630
H	1.7720060	-2.7059660	2.5856910	H	-2.9715430	-0.2872120	3.7037200
H	5.5985570	-2.7103480	1.8108300	H	-6.3872220	-0.0481710	1.9508400
H	5.8370230	-1.1408630	1.0487980	H	-6.0088910	-0.3316270	0.2499700
H	4.2887700	-1.5396180	3.8180560	H	-4.8782530	-2.2079190	2.8134230
H	4.5852710	0.0132460	3.0421380	H	-4.7291480	-2.5823590	1.1007020
H	0.8864260	4.4851560	-0.2906420	H	0.1706350	-2.5153760	-3.4938630
H	2.2101840	5.1866860	0.6307630	H	-0.8119130	-3.9710860	-3.5573160
H	4.1429210	1.8465800	1.7129760	H	-3.6818640	-2.9024290	-0.9100990
H	2.9960150	2.6213300	2.7895340	H	-2.3742320	-4.0280610	-0.5777320
H	2.6599880	2.8115620	-1.8562220	H	-1.9184060	-0.6408820	-3.7584910
H	3.6468990	4.0456700	-1.0852130	H	-2.6723260	-2.1408420	-4.2753730
H	-5.7855860	3.0279180	-0.0496750	H	6.5583100	-0.4241580	-1.5450490
H	-5.9304990	1.3397780	0.4398900	H	6.2340330	0.0023130	0.1352270
H	-3.6847550	1.5904140	2.0920280	H	4.6440390	-2.2204610	0.4289540
H	-2.1414540	2.1479490	1.4704800	H	3.1912310	-2.4981580	-0.5239630
H	-3.2550880	2.8202590	-2.3869560	H	3.7339460	0.7580400	-3.0479490
H	-3.6795970	4.0627640	-1.2137730	H	4.5742710	-0.7483530	-3.3778580
H	-4.8293550	-0.8729400	1.3150220	H	4.3339120	0.2270670	2.3669030
H	-5.1026430	-2.4863420	0.6988600	H	3.8231420	1.2621360	3.6923680

H	-3.5509060	-4.5438660	1.3964000	H	1.2439050	1.1391080	4.7169670
H	-3.0312910	-4.1719710	3.0351170	H	1.7610180	-0.3964360	5.4128540
H	-2.9185910	-1.6396420	3.9222510	H	2.3125720	-2.5670660	3.6780730
H	-2.5815680	-0.2744020	2.8627660	H	3.0332220	-2.2512650	2.1091030
H	5.0014560	2.1393970	-2.1382260	H	-0.7324410	-0.0176350	0.9753660
H	5.3547470	2.3842210	-0.4261870	H	-4.3529520	-0.2732820	-3.8691640
H	4.3137770	1.0421820	-0.9126140	H	-4.6844390	-1.6968230	-2.8801940
H	-0.1581650	3.6572890	1.8728820	H	-3.9322040	-0.2783150	-2.1488320
H	1.1307470	4.4537170	2.7850330	H	0.9596420	-3.1610110	-1.1660160
H	-0.0055930	5.4243880	1.8399520	H	0.0508270	-4.6740750	-1.2857700
H	5.2245090	3.8345120	2.7608240	H	1.4027840	-4.3751100	-2.3838900
H	5.0143880	4.1844610	1.0439520	H	-4.2302440	-5.1370880	-1.8673490
H	3.9110730	4.8871510	2.2328020	H	-4.0200990	-3.9986840	-3.1987380
H	1.1463390	-3.0234380	0.2366710	H	-2.7374580	-5.1546180	-2.8063350
H	2.6465560	-3.8869000	-0.1213010	H	-1.8180320	1.5938110	2.4880680
H	1.4487980	-4.6134740	0.9548580	H	-3.3307300	2.5107120	2.4554540
H	2.0388260	0.4545600	3.1223700	H	-2.5444360	2.1475360	3.9964360
H	1.8853020	-0.9904030	4.1299840	H	-2.1746570	-2.6602270	1.3833820
H	2.8254330	0.3863180	4.7098190	H	-2.4012760	-2.4337850	3.1258400
H	4.8280090	-3.6911730	-0.3484090	H	-3.0212540	-3.8837160	2.3361530
H	4.8030780	-2.0867570	-1.1009270	H	-5.5906980	2.3212160	1.7709390
H	6.3431820	-2.8377320	-0.6444850	H	-5.0368920	2.0316680	0.1098590
H	-0.5369590	-0.7513380	4.1236330	H	-6.7660490	2.0102410	0.4923270
H	-0.5783790	-2.4592170	3.6855670	H	1.0322320	-3.6753300	1.8192570
H	-0.2372630	-1.2304430	2.4442540	H	0.0054450	-2.4986480	2.6398390
H	-0.6194340	-4.2086300	2.2886640	H	0.7034380	-2.1132720	1.0692200
H	-1.4170960	-5.7193460	1.8328880	H	-0.2919910	-1.5277300	4.6525190
H	-1.1380820	-4.4500600	0.6213800	H	-0.8052780	-0.0005780	5.3750290
H	-5.1987120	-1.6943540	3.6846770	H	-0.7221550	-0.1530110	3.6140850
H	-6.5765130	-2.1132900	2.6679920	H	4.7207790	-1.6855760	3.9685710
H	-5.3914320	-3.3498630	3.0898720	H	5.6285900	-0.2623430	4.4794900
H	-1.1924400	4.0615830	-1.9581990	H	4.1400860	-0.6971690	5.3188860
H	-1.4371460	4.0111320	-0.2100050	H	2.1767570	-0.8094120	-4.1809280
H	-0.9894290	2.5434010	-1.0707140	H	2.4851240	-2.0651560	-2.9773120
H	-5.8464370	0.6404000	-1.9934980	H	1.5994930	-0.6043950	-2.5267150
H	-7.2191810	1.7144270	-1.6637040	H	5.4064730	2.2843570	-0.6208100
H	-5.7806630	2.3426590	-2.4746750	H	7.0979490	2.0003130	-1.0641800
H	-4.6624040	3.9052250	1.8506970	H	5.8355360	1.8722610	-2.2913920
H	-3.2152040	3.9348180	2.8598290	H	6.0768320	-2.7954270	-1.5902660
H	-3.1324010	4.4938360	1.1892880	H	4.9931560	-4.1163600	-1.1514380
				H	4.6103110	-3.0930400	-2.5365020
				H	1.0418370	-0.3800250	-0.3415860
**$\mathrm{II(Pc_2)_2}$**				**$\mathrm{II(Pc_2)_2}$ (diprotonated)**			
C	-0.4686960	-1.9949250	2.4632870	C	-0.7182330	-1.8535970	2.5532540
C	1.0709310	-1.7699340	2.3389330	C	0.8291260	-1.6987040	2.4454900
C	1.4381950	-0.3560530	1.8985820	C	1.3054420	-0.3120940	2.0124410
C	0.7745430	0.4531800	3.0411640	C	0.6493270	0.5621540	3.1124490
C	-0.7827790	0.2095680	3.2120050	C	-0.9249960	0.4018670	3.2389450
C	-1.3580050	-0.7781320	2.1715020	C	-1.5410140	-0.6022420	2.2299960
C	1.1959140	-1.7141670	3.8973860	C	0.9304860	-1.6131470	4.0071970
C	1.5075070	-0.2400270	4.2262450	C	1.3133560	-0.1536960	4.3245090
C	-1.0179020	-0.6508380	4.4821070	C	-1.2356090	-0.4215060	4.5211350
C	-0.3352940	-1.9564860	4.0298120	C	-0.6117200	-1.7728510	4.1171190
C	-0.4526270	-0.0850550	5.7796030	C	-0.6751560	0.1503630	5.8159580
C	1.0536610	0.1901300	5.6157830	C	0.8487890	0.3220320	5.6938340
P	1.3404050	0.0649040	0.0861600	P	1.5365760	0.1990200	0.2846760
N	2.0482140	1.6107260	0.2418010	N	2.1667180	1.6663900	0.4149160
P	1.7042520	2.8182140	-0.7204510	P	2.0668310	3.0230530	-0.4241690
C	3.0949020	3.9993920	-0.6501190	C	3.3897390	4.1112980	0.1808780
C	3.0239700	5.2183660	-1.5712920	C	3.5364000	5.4518360	-0.5449300
C	4.2674580	6.0960470	-1.4370880	C	4.6692060	6.2839220	0.0529250
P	-1.9022940	-0.1038570	0.4976980	P	-2.0896030	-0.0972890	0.5681170
N	-2.0742440	-1.5480110	-0.3872480	N	-2.0500910	-1.2902960	-0.4814400
P	-1.3964740	-2.0653260	-1.7105830	P	-1.3167530	-2.2271920	-1.5286510
C	-0.8498450	-0.8530250	-2.9842310	C	-0.2712670	-1.3534070	-2.7396020
C	-1.8112120	0.3225360	-3.1681280	C	-0.8296940	-0.0143750	-3.2246470
C	-1.5505740	1.1240190	-4.4415120	C	0.0039880	0.5478370	-4.3728650
N	-3.4906680	0.2291070	1.0589410	N	-3.5380710	0.5453550	0.8407300
P	-4.6976530	0.7553140	0.1919580	P	-4.6322890	1.0148670	-0.2360030
C	-4.3573950	2.0609450	-1.0536840	C	-3.9464500	1.8438290	-1.7067370
C	-3.5802140	3.2519350	-0.4934310	C	-3.1567570	3.1177350	-1.3916230
C	-3.1655870	4.2349370	-1.5856440	C	-2.4359340	3.6581870	-2.6242030
C	-5.5767930	-0.5047840	-0.8106710	C	-5.6298830	-0.3567790	-0.8926190
C	-6.2326110	-1.6114420	0.0178180	C	-6.2925900	-1.2049290	0.1969580
C	-7.0020900	-2.6029850	-0.8518010	C	-7.1893700	-2.2899870	-0.3932460
C	-5.9455100	1.4596660	1.3259840	C	-5.7401040	2.1737350	0.6135020
C	-7.2619380	1.9261900	0.6994550	C	-6.9157450	2.6950940	-0.2183940
C	-8.2264660	2.4739400	1.7501860	C	-7.7594020	3.6926240	0.5727920
N	2.5401570	-0.9651230	-0.5170420	N	2.3354740	-0.9254300	-0.5169090
P	4.0690670	-1.2830060	-0.3098560	P	3.7218500	-1.7058800	-0.6008630
C	4.6186090	-2.3071950	-1.7223120	C	3.8066770	-2.4385570	-2.2615210
C	4.2808920	-1.6868790	-3.0799960	C	3.6709600	-1.3979610	-3.3792240
C	4.6514480	-2.6003680	-4.2455360	C	3.4581010	-2.0435960	-4.7449970
C	5.2493440	0.1239090	-0.2637030	C	5.2084710	-0.6802600	-0.3727760
C	5.2025780	0.9600040	1.0181050	C	5.2902490	0.0285890	0.9833960
C	6.2089180	2.1085940	0.9842380	C	6.5297060	0.9161970	1.0739180

C	4.4807110	-2.2950610	1.1694950
C	5.9505100	-2.6755010	1.3694160
C	6.1499410	-3.5069420	2.6358690
C	1.4860280	2.3959430	-2.4897500
C	2.7116920	1.7145840	-3.1030010
C	2.4378120	1.1606720	-4.4989320
C	0.1573010	3.7314160	-0.3324010
C	-0.2192020	3.7501950	1.1521250
C	0.8103510	4.4152120	2.0628680
C	0.1087820	-3.0964360	-1.5433150
C	-0.0346680	-4.3671000	-0.7048380
C	1.3220290	-5.0125710	-0.4257220
C	-2.5966310	-3.1061160	-2.6306960
C	-3.4991040	-3.9768080	-1.7557890
C	-4.4562990	-4.8247340	-2.5906000
H	-0.8408500	-2.9207770	2.0211320
H	2.5180010	-0.2270830	2.0608990
H	-2.3478810	-1.0686580	2.5436790
H	2.5833060	-0.0527540	4.1303670
H	1.0001660	1.5208560	2.9752090
H	1.8517970	-2.4355520	4.3923070
H	-0.6398020	-2.8433160	4.5922480
H	-2.0956250	-0.8065100	4.6019640
H	-1.3229460	1.1614480	3.2465560
H	1.6135240	-2.5613890	1.8179140
H	-0.6363690	-0.7964370	6.5932560
H	-0.9933750	0.8314810	6.0409690
H	1.6372050	-0.3481590	6.3714950
H	1.2756170	1.2547170	5.7523270
H	3.1962430	4.3105060	0.3947720
H	3.9835030	3.3996160	-0.8795660
H	1.1946040	3.2920410	-3.0509780
H	0.6278820	1.7139390	-2.5110400
H	0.2265630	4.7477450	-0.7381950
H	-0.6306810	3.2204900	-0.8975990
H	4.1116400	-3.2744920	-1.6157960
H	5.6951450	-2.4936810	-1.6367180
H	5.0116120	0.7536080	-1.1291470
H	6.2564540	-0.2800510	-0.4284520
H	4.1095130	-1.7450210	2.0419770
H	3.8617270	-3.1974800	1.0936710
H	-0.7291640	-1.4097170	-3.9231670
H	0.1419950	-0.5009710	-2.6802210
H	0.4388280	-3.3496540	-2.5601120
H	0.8809250	-2.4428450	-1.1173880
H	-3.2181990	-2.4273910	-3.2291650
H	-2.0301410	-3.7210490	-3.3413600
H	-3.7838090	1.5828750	-1.8542090
H	-5.3065230	2.3816470	-1.4992700
H	-4.8189160	-0.9343700	-1.4778930
H	-6.3211430	0.0006460	-1.4384860
H	-5.4522930	2.2901150	1.8441790
H	-6.1332790	0.6927530	2.0857950
H	6.3124450	-3.2399570	0.5029290
H	6.5648680	-1.7698720	1.4305960
H	3.2097000	-1.4625120	-3.1039040
H	4.8011090	-0.7269180	-3.1871230
H	4.1863500	1.3535290	1.1308800
H	5.4118040	0.3233050	1.8870270
H	2.9202210	4.8934980	-2.6130330
H	2.1333200	5.8131390	-1.3395810
H	-1.1810670	4.2678280	1.2463930
H	-0.3840220	2.7177730	1.4796090
H	3.5470230	2.4246370	-3.1453360
H	3.0175760	0.8958990	-2.4435660
H	-7.0650750	2.7012100	-0.0499080
H	-7.7391440	1.0941190	0.1687880
H	-5.4587490	-2.1322820	0.5921550
H	-6.9193430	-1.1726660	0.7519510
H	-4.1867460	3.7717700	0.2579840
H	-2.6936280	2.8740940	0.0263530
H	-2.8926770	-4.6303070	-1.1197440
H	-4.0551120	-3.3244510	-1.0753520
H	-2.8466930	-0.0421700	-3.1859540
H	-1.7302970	0.9700420	-2.2872530
H	-0.5269690	-4.1257510	0.2434520
H	-0.6811350	-5.0875880	-1.2197330
H	1.0717640	5.4183300	1.7071140
H	0.4219240	4.5101140	3.0811410
H	1.7260320	3.8180270	2.1131530
H	3.3357260	0.6990050	-4.9208930
H	1.6544930	0.3956100	-4.4656940
H	2.1074120	1.9487260	-5.1842610
H	5.1713070	5.5323210	-1.6905300
H	4.2124730	6.9631610	-2.1011170
H	4.3783310	6.4616470	-0.4111310
H	-9.1619560	2.8065380	1.2916280

C	3.7931000	-3.0645000	0.6085860
C	5.0564650	-3.9307190	0.5878800
C	4.9603210	-5.0668740	1.6043850
C	2.2964580	2.8380260	-2.2217380
C	3.6494830	2.2334790	-2.6043630
C	3.8215220	2.0946660	-4.1142020
C	0.4414330	3.8411640	-0.2630870
C	-0.2366530	3.7227980	1.1060390
C	0.5208530	4.3676990	2.2632090
C	-0.1771730	-3.4410240	-0.7877600
C	-0.8066130	-4.5876070	0.0088240
C	0.2330310	-5.3113940	0.8620050
C	-2.5745310	-3.1032080	-2.5077210
C	-3.7169340	-3.7419320	-1.7121620
C	-4.8008530	-4.2824610	-2.6422450
H	-1.1224180	-2.7731360	2.1320700
H	2.3820020	-0.2854480	2.2492860
H	-2.5469470	-0.8219170	2.6113460
H	2.3977680	-0.0206420	4.2547680
H	0.9330190	1.6130430	3.0381140
H	1.5387180	-2.3623680	4.5160420
H	-0.9745020	-2.6270630	4.6905110
H	-2.3214970	-0.5165120	4.6181540
H	-1.4175890	1.3775790	3.2362900
H	1.3445870	-2.5273580	1.9603350
H	-0.9320850	-0.5222390	6.6408220
H	-1.1643710	1.1065320	6.0274630
H	1.3742330	-0.2490230	6.4661280
H	1.1397850	1.3691510	5.8257940
H	3.2109990	4.2665110	1.2487720
H	4.3113740	3.5229380	0.1034600
H	2.1542560	3.8219810	-2.6838840
H	1.4743450	2.2056150	-2.5771310
H	0.5615680	4.8908000	-0.5538390
H	-0.1884920	3.3770590	-1.0312960
H	2.9824380	-3.1599490	-2.3107160
H	4.7399130	-3.0024450	-2.3603750
H	5.2177910	0.0551290	-1.1839580
H	6.0781040	-1.3293550	-0.5292140
H	3.6591320	-2.6130350	1.5986900
H	2.9030400	-3.6772160	0.4189550
H	-0.1340130	-2.0424180	-3.5821980
H	0.7134540	-1.2168040	-2.2761160
H	0.4338340	-3.8397080	-1.6067850
H	0.4994660	-2.8453410	-0.1638950
H	-2.9783250	-2.3589230	-3.2054190
H	-2.0497720	-3.8524390	-3.1119980
H	-3.3036930	1.1013470	-2.1948520
H	-4.7664110	2.0517710	-2.4030130
H	-4.9531410	-0.9655210	-1.5038140
H	-6.3789270	0.0672400	-1.5714380
H	-5.1082930	2.9997000	0.9587760
H	-6.0946520	1.6592200	1.5133390
H	0.3278830	0.2320350	-0.4159120
H	5.2088250	-4.3482800	-0.4129520
H	5.9328940	-3.3127560	0.8110750
H	2.8264230	-0.7380200	-3.1529020
H	4.5663840	-0.7671940	-3.4053360
H	4.3967350	0.6460510	1.1246290
H	5.3093570	-0.7128490	1.7906500
H	3.7334490	5.2819120	-1.6092410
H	2.5993270	6.0158820	-0.4833850
H	-1.2273000	4.1807790	1.0187060
H	-0.4068730	2.6641750	1.3314140
H	4.4619970	2.8499990	-2.2035680
H	3.7347910	1.2521000	-2.1283370
H	-6.5449350	3.1750460	-1.1312350
H	-7.5464500	1.8578670	-0.5366040
H	-5.5139920	-1.6549610	0.8220710
H	-6.8898150	-0.5632220	0.8550290
H	-3.8338630	3.8828610	-0.9968770
H	-2.4316330	2.9096550	-0.5965860
H	-3.3330500	-4.5545790	-1.0900170
H	-4.1389050	-3.0008150	-1.0249560
H	-1.8691570	-0.1270100	-3.5541300
H	-0.8409140	0.6966610	-2.3882580
H	-1.6125050	-4.2109280	0.6481600
H	-1.2661520	-5.2983960	-0.6852030
H	0.7800420	5.4076450	2.0398280
H	-0.0897530	4.3580710	3.1698090
H	1.4448560	3.8265250	2.4882310
H	4.7985030	1.6664220	-4.3540980
H	3.0560130	1.4416070	-4.5445810
H	3.7475870	3.0682590	-4.6080370
H	5.6234670	5.7529870	-0.0195870
H	4.7707460	7.2366570	-0.4729680
H	4.4815540	6.4978030	1.1096460

H	-8.4681620	1.7084150	2.4946290	H	-8.5974940	4.0554220	-0.0277820
H	-7.7865830	3.3264160	2.2778130	H	-8.1658080	3.2291380	1.4770990
H	-7.8202990	-2.1033820	-1.3811000	H	-7.1605780	4.5567640	0.8767110
H	-6.3485470	-3.0562430	-1.6030020	H	-8.0003500	-1.8495640	-0.9816670
H	-7.4317580	-3.4085730	-0.2492780	H	-6.6227180	-2.9548320	-1.0510460
H	-2.5416490	3.7394750	-2.3385330	H	-7.6363710	-2.8981900	0.3974960
H	-4.0403630	4.6480580	-2.0985090	H	-1.7135010	2.9293090	-3.0079890
H	-2.5904450	5.0682640	-1.1705220	H	-3.1452500	3.8784160	-3.4275110
H	1.9652920	-4.3274820	0.1365000	H	-1.8951300	4.5790200	-2.3892210
H	1.2162540	-5.9322470	0.1573590	H	0.6536240	-4.6436400	1.6212220
H	1.8384030	-5.2613200	-1.3593940	H	-0.2104190	-6.1656660	1.3795640
H	-5.1300460	-5.4053680	-1.9533810	H	1.0580560	-5.6816290	0.2443240
H	-5.0709540	-4.1962760	-3.2449110	H	-5.5890940	-4.7842290	-2.0742990
H	-3.9069980	-5.5253540	-3.2283580	H	-5.2609310	-3.4748020	-3.2213860
H	-1.6726590	0.4959320	-5.3301690	H	-4.3851130	-5.0076530	-3.3487590
H	-2.2470840	1.9643340	-4.5234970	H	-0.0637360	-0.0959250	-5.2546280
H	-0.5342200	1.5288320	-4.4594260	H	-0.3296370	1.5499300	-4.6560090
H	7.2019270	-3.7730670	2.7727050	H	1.0599500	0.6079160	-4.0917690
H	5.8233280	-2.9516400	3.5212530	H	5.8638470	-5.6815820	1.5877910
H	5.5693830	-4.4340340	2.5900810	H	4.8354230	-4.6742480	2.6183170
H	6.1566000	2.7050910	1.8999790	H	4.1048670	-5.7139070	1.3858610
H	7.2346490	1.7379900	0.8808240	H	6.5767460	1.4192510	2.0432150
H	6.0121030	2.7777790	0.1392840	H	7.4457910	0.3303810	0.9501080
H	5.7243750	-2.8190660	-4.2532900	H	6.5139380	1.6849590	0.2942730
H	4.1167190	-3.5538230	-4.1787440	H	4.2912460	-2.7030870	-5.0064250
H	4.3949450	-2.1371380	-5.2030130	H	2.5395800	-2.6391690	-4.7541290
				H	3.3730680	-1.2793680	-5.5224670
				H	-1.2636600	0.9610530	0.1360330
**II(Pd_2)$_2$**				**II(Pd_2)$_2$ (diprotonated)**			
C	1.7024370	-1.3631940	-1.1971550	C	1.7109420	-0.5574580	-1.6103720
C	0.9620830	-2.6995700	-1.4504770	C	1.2816500	-1.9796090	-2.0398140
C	1.6904480	-3.0916580	-2.7656910	C	1.9461520	-1.9742250	-3.4414810
C	1.4704400	-1.7947140	-3.5725270	C	1.3646760	-0.6591430	-3.9959390
C	1.3853480	-0.6487730	-2.5081850	C	1.1085880	0.2700590	-2.7551310
C	-0.0451440	-1.6219950	-3.8783080	C	-0.1688790	-0.8186760	-4.1935690
C	-0.8113880	-2.8183600	-3.2836630	C	-0.5949920	-2.2270290	-3.7366530
C	-0.3253930	-4.1731280	-3.7844110	C	0.1245590	-3.3582810	-4.4581180
C	1.1682750	-4.3461750	-3.4538800	C	1.6467970	-3.1953110	-4.2996780
C	-0.5835560	-2.5391020	-1.7736630	C	-0.2866680	-2.1186550	-2.2149580
C	-1.0805200	-1.0729110	-1.7559630	C	-1.0948980	-0.8192140	-1.9609950
C	-0.1356650	-0.4759000	-2.8036920	C	-0.4296360	0.1208180	-2.9683870
P	-1.6521280	-0.2926370	-0.1381740	P	-1.7252590	-0.1925390	-0.3708790
N	-1.9706070	1.2815490	-0.7081360	N	-2.7449010	0.9950080	-0.7333420
P	-3.3908430	1.9576920	-0.8518790	P	-4.3434720	1.0225380	-0.6141260
C	-4.4492480	1.9622180	0.6544350	C	-4.9401940	0.9937450	1.1148040
C	-3.8525430	2.6553860	1.8892160	C	-4.0213150	1.7185290	2.1059550
C	-4.2921200	4.1120170	2.0782710	C	-3.8464350	3.2233600	1.8762380
C	-5.7380390	4.2450510	2.5543200	C	-5.1224140	4.0391980	2.0773840
P	1.7105120	-0.4833930	0.4671180	P	1.7607520	-0.1836370	0.1786080
N	2.6858480	0.8491070	0.0084560	N	1.7571820	1.3766900	0.4678830
P	2.2008450	2.3508210	-0.0865680	P	1.0710590	2.8042550	0.5112050
C	3.6158300	3.4563350	0.2722070	C	2.3384470	4.0373380	0.9569060
C	4.9553650	3.0243390	-0.3315850	C	3.7328490	3.7702390	0.3871140
C	5.0350060	3.0651340	-1.8568920	C	3.8309110	3.7326910	-1.1379620
C	6.4443470	2.7586110	-2.3582420	C	5.2834360	3.7420780	-1.6083600
N	2.7728530	-1.5348680	1.2704350	N	2.9946630	-1.0053690	0.7825590
P	4.3534910	-1.4594540	1.2784650	P	4.5755140	-0.7685100	0.8004920
C	5.0023290	-3.0149730	2.0028890	C	5.3344180	-2.3816180	1.1709780
C	4.1482400	-4.2474180	1.6798180	C	4.6903950	-3.5743330	0.4512320
C	4.0194260	-4.5767190	0.1918030	C	4.7487900	-3.5137200	-1.0763380
C	3.1723940	-5.8221370	-0.0571790	C	4.3088950	-4.8136100	-1.7438730
N	-3.1789360	-1.0889040	-0.1489730	N	-2.3002700	-1.3948380	0.4737000
P	-3.7379740	-2.1786620	0.8455740	P	-2.4565720	-2.6628540	1.3953810
C	-4.2659020	-1.5876980	2.5033680	C	-1.9343470	-2.3539770	3.1114990
C	-3.2527670	-0.6989940	3.2259810	C	-0.6014920	-1.6096470	3.2446760
C	-3.8205690	-0.1251740	4.5242720	C	-0.1254150	-1.5382700	4.6961650
C	-2.8534750	0.8293140	5.2177430	C	1.3162320	-1.0507040	4.8039810
C	-5.2678810	-2.8424570	0.1030710	C	-4.2121060	-3.1291910	1.4434430
C	-6.0955280	-3.8171290	0.9452910	C	-4.6069490	-4.2154570	2.4490760
C	-7.2862140	-4.4033840	0.1778710	C	-6.0869680	-4.5988880	2.3358980
C	-8.3027930	-3.3592430	-0.2823830	C	-7.0501680	-3.4447390	2.6114510
C	-2.6968370	-3.6621990	1.1501790	C	-1.5690240	-4.1281250	0.7747700
C	-1.2905700	-3.3866680	1.6982580	C	-0.0388250	-4.0500220	0.8336240
C	-0.3466730	-4.5832470	1.5528500	C	0.6519040	-5.1159290	-0.0197580
C	-0.7741020	-5.8202520	2.3395970	C	0.2942690	-6.5493790	0.3658530
C	5.2187230	-1.2433300	-0.3333810	C	5.2704150	-0.1085890	-0.7577380
C	6.7150430	-0.9238320	-0.2777880	C	6.7774210	0.1705820	-0.7470710
C	7.3667130	-1.0263700	-1.6583250	C	7.2961440	0.4973000	-2.1491980
C	8.8212500	-0.5636070	-1.6624370	C	8.7682060	0.8994920	-2.1461150
C	5.0404900	-0.0970820	2.2969450	C	5.1348640	0.4091840	2.0840990
C	4.3014220	0.1280740	3.6142970	C	4.1374690	0.6023680	3.2271480
C	4.7012420	1.4447690	4.2802320	C	4.5880250	1.6871580	4.2047230
C	3.9802300	1.6795060	5.6045490	C	3.6045160	1.8730760	5.3575460
C	1.5024480	2.8215350	-1.7184280	C	0.2951270	3.2844950	-1.0675200
C	1.4372900	4.3107730	-2.0620290	C	-0.3296770	4.6826600	-1.1331450
C	0.6624150	4.5656950	-3.3556450	C	-0.6527640	5.0756700	-2.5764780

C	0.7040440	6.0285710	-3.7898780
C	0.8697930	2.8690780	1.0604930
C	1.1586210	2.6081420	2.5392030
C	-0.0882150	2.7986340	3.4033320
C	0.1934720	2.6342370	4.8937520
C	-3.1739880	3.6983980	-1.3657400
C	-1.9976130	4.4071530	-0.6900240
C	-1.8669900	5.8775510	-1.0944070
C	-2.9699360	6.7764930	-0.5369230
C	-4.5419650	1.2309240	-2.0822350
C	-3.9310010	1.0361860	-3.4710820
C	-4.7858580	0.1556910	-4.3898690
C	-4.8826440	-1.2998520	-3.9318230
H	-0.4356810	0.5153990	-3.1509050
H	2.7685860	-1.6107660	-1.2596340
H	-2.0656860	-1.0916300	-2.2402580
H	2.7565800	-3.2258280	-2.5481430
H	1.1205510	-3.4326100	-0.6539790
H	2.1557810	-1.6657360	-4.4145920
H	-0.3108240	-1.3821180	-4.9115660
H	-1.8828030	-2.7299650	-3.4938290
H	-1.1794520	-3.2047760	-1.1445320
H	2.0024260	0.2315490	-2.6851900
H	-0.5012140	-4.2436500	-4.8641700
H	-0.9248550	-4.9644100	-3.3200250
H	1.7540830	-4.5354610	-4.3607140
H	1.3275380	-5.2078840	-2.7950370
H	0.6623590	3.9331040	0.8904390
H	-0.0245780	2.3191680	0.7361210
H	1.5311350	1.5844830	2.6654420
H	1.9524940	3.2779310	2.8952670
H	-0.5148310	3.7924120	3.2124730
H	-0.8458940	2.0696750	3.0871220
H	-0.7019240	2.8148790	5.4950270
H	0.5417790	1.6190960	5.1136260
H	0.9683320	3.3339820	5.2256600
H	2.0912490	2.2982970	-2.4771560
H	0.5005880	2.3683900	-1.7317330
H	0.9740110	4.8817380	-1.2471990
H	2.4541380	4.7057410	-2.1734010
H	1.0771950	3.9335020	-4.1507730
H	-0.3780950	4.2434060	-3.2200860
H	0.1135950	6.1924270	-4.6959980
H	0.3060980	6.6824780	-3.0070690
H	1.7319970	6.3449360	-3.9962420
H	3.3558460	4.4771480	-0.0300440
H	3.7029240	3.4651850	1.3646760
H	5.7386870	3.6705860	0.0836420
H	5.1776930	2.0038470	-0.0004650
H	4.3347870	2.3351080	-2.2791660
H	4.7202030	4.0516290	-2.2202540
H	6.7831230	1.7853620	-1.9871770
H	6.4871950	2.7346260	-3.4507760
H	7.1581540	3.5118600	-2.0082900
H	5.0493540	-2.1593140	-0.9105310
H	4.6778740	-0.4334520	-0.8346130
H	6.8594970	0.0907190	0.1148280
H	7.2390890	-1.5986760	0.4119760
H	7.3042220	-2.0650010	-2.0059870
H	6.7864170	-0.4304630	-2.3742770
H	8.8976610	0.4881290	-1.3651420
H	9.4222340	-1.1513530	-0.9603870
H	9.2692920	-0.6646040	-2.6550800
H	5.0511840	-2.8744840	3.0881080
H	6.0339390	-3.1486250	1.6563000
H	4.5754770	-5.1102850	2.2061350
H	3.1452200	-4.0870770	2.0889230
H	3.5506820	-3.7299270	-0.3209700
H	5.0174590	-4.7077720	-0.2472100
H	3.0844940	-6.0338740	-1.1272340
H	3.6048720	-6.7041510	0.4276730
H	2.1599630	-5.6855790	0.3395110
H	4.9415510	0.7963150	1.6712020
H	6.1098040	-0.2702930	2.4664820
H	3.2223460	0.1313230	3.4213450
H	4.4914050	-0.7051640	4.3036930
H	5.7869300	1.4610770	4.4393890
H	4.4788900	2.2693170	3.5895500
H	2.8955110	1.6923850	5.4585130
H	4.2101570	0.8856080	6.3232060
H	4.2694370	2.6339460	6.0543640
H	-3.2502560	-4.3335410	1.8160920
H	-2.6280600	-4.1690390	0.1788490
H	-0.8501820	-2.5257050	1.1819710
H	-1.3584390	-3.1099400	2.7570980
H	-0.2595580	-4.8384090	0.4881440
H	0.6537980	-4.2715690	1.8763360

C	-1.2217440	6.4871970	-2.6878270
C	-0.2370380	2.9441200	1.7717080
C	0.1997650	2.4578670	3.1551710
C	-0.9258490	2.5050890	4.1888070
C	-0.4303260	2.2320670	5.6069410
C	-4.9616730	2.5555930	-1.3881750
C	-3.9397180	3.2756620	-2.2696010
C	-4.4355850	4.6432640	-2.7462160
C	-4.7235320	5.6194420	-1.6044480
C	-5.1614110	-0.3900940	-1.4182070
C	-4.9284490	-0.4610020	-2.9309030
C	-5.4068580	-1.7784400	-3.5505490
C	-4.7318320	-3.0217750	-2.9696730
H	-0.9750100	1.0483260	-3.1492650
H	2.7925800	-0.5037960	-1.7869960
H	-2.0935320	-1.0394790	-2.3658540
H	3.0307690	-1.8882000	-3.3142800
H	1.6767590	-2.7675910	-1.3920980
H	1.9220750	-0.2352160	-4.8322860
H	-0.5732380	-0.5164450	-5.1605080
H	-1.6741310	-2.3545010	-3.8696510
H	-0.6721240	-2.9723180	-1.6561090
H	1.4942170	1.2870570	-2.8176550
H	-0.1659360	-3.3487700	-5.5134550
H	-0.2142600	-4.3155490	-4.0484290
H	2.1356950	-3.0776800	-5.2720740
H	2.0904750	-4.0790300	-3.8295880
H	-0.5423110	3.9968090	1.8068370
H	-1.1017000	2.3793340	1.4027190
H	0.5848940	1.4349500	3.0738150
H	1.0327000	3.0748720	3.5131680
H	-1.4020360	3.4926020	4.1584950
H	-1.6988670	1.7760160	3.9165250
H	-1.2583540	2.2349080	6.3208840
H	0.0686310	1.2609370	5.6752440
H	0.2882980	2.9960220	5.9216560
H	1.0775760	3.1905590	-1.8266510
H	-0.4654470	2.5247460	-1.2853020
H	-1.2424310	4.7144270	-0.5254140
H	0.3511230	5.4328870	-0.7148100
H	0.2634470	5.0020740	-3.1750010
H	-1.3564380	4.3500100	-3.0022180
H	-1.4686180	6.7317080	-3.7247480
H	-2.1329200	6.5962720	-2.0918810
H	-0.4995100	7.2281080	-2.3298370
H	1.9696970	5.0230240	0.6558930
H	2.3908100	4.0438380	2.0500400
H	4.3985630	4.5495670	0.7746420
H	4.0946780	2.8194680	0.7913150
H	3.3323040	2.8291450	-1.5122220
H	3.2978270	4.5893030	-1.5695580
H	5.8538270	2.9362930	-1.1331290
H	5.3558160	3.6125870	-2.6916040
H	5.7723790	4.6852720	-1.3455690
H	5.0298980	-0.8154950	-1.5567170
H	4.7160060	0.8151830	-0.9702110
H	6.9988410	1.0044160	-0.0699890
H	7.3266130	-0.6967050	-0.3619840
H	7.1481500	-0.3773390	-2.7941150
H	6.6930400	1.3050190	-2.5803990
H	8.9256500	1.7995390	-1.5426320
H	9.3927960	0.1031050	-1.7282510
H	9.1225870	1.1079310	-3.1592320
H	5.2494710	-2.5092200	2.2555990
H	6.4036700	-2.3113100	0.9445730
H	5.2023550	-4.4793740	0.7972900
H	3.6460540	-3.6570920	0.7717000
H	4.0969090	-2.7062320	-1.4289280
H	5.7676790	-3.2617500	-1.3974280
H	4.3771410	-4.7358750	-2.8327480
H	4.9344610	-5.6533310	-1.4246900
H	3.2710550	-5.0555130	-1.4930430
H	5.3202440	1.3667190	1.5857800
H	6.1052310	0.0608960	2.4565950
H	3.1625780	0.8753910	2.8066710
H	3.9950470	-0.3430840	3.7633600
H	5.5797610	1.4367370	4.6007590
H	4.7013750	2.6330780	3.6593020
H	2.6099240	2.1408720	4.9858440
H	3.5006610	0.9505520	5.9381200
H	3.9325040	2.6646270	6.0370140
H	-1.9310550	-4.9918930	1.3418350
H	-1.9174650	-4.2602470	-0.2572420
H	0.2994140	-3.0619160	0.5015370
H	0.2880800	-4.1607440	1.8734650
H	0.4140970	-4.9486820	-1.0787910
H	1.7334090	-4.9735810	0.0815560

H	-1.7325400	-6.2120860	1.9843520	H	-0.7630450	-6.7651430	0.1840650
H	-0.0343200	-6.6206020	2.2437050	H	0.8811530	-7.2646180	-0.2164390
H	-0.8824320	-5.5876930	3.4046390	H	0.4970720	-6.7303900	1.4266960
H	-5.8551010	-1.9562910	-0.1624120	H	-4.7506850	-2.1998820	1.6512310
H	-4.9644520	-3.3094380	-0.8419020	H	-4.4767760	-3.4306520	0.4226560
H	-5.4627130	-4.6424900	1.2905080	H	-3.9985450	-5.1128390	2.2941930
H	-6.4650820	-3.3077540	1.8436960	H	-4.4027570	-3.8649160	3.4675590
H	-6.9133800	-4.9607580	-0.6906390	H	-6.2759130	-5.0070770	1.3354910
H	-7.7865020	-5.1343490	0.8231430	H	-6.2824630	-5.4108960	3.0443790
H	-8.6565940	-2.7589330	0.5630770	H	-6.8452400	-2.9875770	3.5855680
H	-7.8751470	-2.6751160	-1.0218520	H	-6.9767670	-2.6605430	1.8511570
H	-9.1724900	-3.8370990	-0.7421450	H	-8.0851850	-3.7969230	2.6168230
H	-5.1923610	-1.0219310	2.3360630	H	-2.7376130	-1.7842320	3.5930030
H	-4.5329590	-2.4548140	3.1194160	H	-1.8840700	-3.3277460	3.6129110
H	-2.3402700	-1.2635440	3.4483280	H	0.1736770	-2.1057850	2.6521330
H	-2.9466960	0.1164810	2.5601650	H	-0.7073630	-0.5968520	2.8353510
H	-4.7604280	0.3991460	4.3046450	H	-0.7955810	-0.8853290	5.2683430
H	-4.0818520	-0.9493490	5.1997540	H	-0.2026720	-2.5343380	5.1481390
H	-3.2601990	1.1959310	6.1647250	H	1.6340060	-0.9674530	5.8472190
H	-1.8971000	0.3389890	5.4280840	H	1.9975870	-1.7408690	4.2953040
H	-2.6445980	1.6970640	4.5836430	H	1.4437100	-0.0684680	4.3399430
H	-3.0208140	3.7013260	-2.4516650	H	-5.8635780	2.2998100	-1.9549750
H	-4.1259410	4.2098460	-1.1819090	H	-5.2849350	3.2079740	-0.5745390
H	-2.0790990	4.3394140	0.4008000	H	-3.0129260	3.4032020	-1.6990510
H	-1.0883240	3.8611160	-0.9511690	H	-3.6857650	2.6480120	-3.1306760
H	-0.8961110	6.2511850	-0.7465510	H	-3.6697240	5.0712500	-3.4025590
H	-1.8496880	5.9510530	-2.1892630	H	-5.3346590	4.5179940	-3.3615300
H	-3.9610290	6.4695500	-0.8867320	H	-5.6168170	5.3390360	-1.0379720
H	-2.9794600	6.7465580	0.5579660	H	-3.8844310	5.6554350	-0.8992870
H	-2.8219670	7.8162380	-0.8426230	H	-4.8844710	6.6315410	-1.9856680
H	-4.6116440	0.8974080	0.8470890	H	-5.0232230	-0.0590240	1.3995810
H	-5.4222920	2.3907300	0.3868000	H	-5.9571970	1.4008720	1.1202000
H	-4.1490590	2.0981520	2.7850380	H	-4.4190110	1.5533380	3.1138900
H	-2.7585360	2.5915500	1.8479670	H	-3.0412590	1.2274190	2.0808520
H	-3.6278310	4.5888220	2.8087630	H	-3.0884970	3.5854510	2.5779670
H	-4.1670650	4.6681290	1.1430010	H	-3.4305760	3.4042860	0.8761660
H	-6.4405740	3.7989040	1.8427030	H	-5.9269130	3.7369000	1.3987770
H	-5.8777850	3.7418810	3.5172010	H	-5.4987330	3.9204840	3.0984480
H	-6.0178070	5.2951630	2.6784890	H	-4.9345500	5.1026810	1.9068970
H	-5.4473740	1.8487430	-2.1354140	H	-6.2317130	-0.3475100	-1.1852640
H	-4.8114790	0.2650550	-1.6428660	H	-4.7540220	-1.2736530	-0.9149590
H	-3.7823880	2.0124940	-3.9477980	H	-5.4415680	0.3746380	-3.4194870
H	-2.9369030	0.5855260	-3.3662740	H	-3.8611260	-0.3318160	-3.1478740
H	-5.7928270	0.5843000	-4.4738600	H	-6.4935430	-1.8632450	-3.4311230
H	-4.3544430	0.1830020	-5.3973280	H	-5.2158740	-1.7327220	-4.6279750
H	-3.8843490	-1.7390750	-3.8283960	H	-3.6409750	-2.9143320	-2.9555560
H	-5.3825260	-1.3930130	-2.9626500	H	-5.0584680	-3.2222580	-1.9447530
H	-5.4438780	-1.9021730	-4.6521880	H	-4.9708210	-3.9077260	-3.5635490
				H	0.6681290	-0.8390300	0.7664010
				H	-0.7088660	0.4656910	0.3287660
**II(Pe_2)$_2$**				**II(Pe_2)$_2$ (diprotonated)**			
C	-4.6691940	5.4631660	1.0722510	C	4.4895940	5.1422910	-1.9061330
C	-4.1983910	4.5313720	-0.0453210	C	4.0647150	4.3904500	-0.6443080
C	-2.6676410	4.4472720	0.1582980	C	2.5197530	4.4331930	-0.7079530
C	-2.4432940	4.6953700	1.6803790	C	2.1666660	4.4739530	-2.2259490
C	-3.8340680	5.0060170	2.2695550	C	3.5158960	4.6168910	-2.9628600
P	-1.9497030	2.8687540	-0.4434460	P	1.7391850	3.0494840	0.1879490
C	-2.7738720	2.5439360	-2.0601500	C	2.6724450	2.8069470	1.7430770
C	-2.1416220	1.3667450	-2.8204990	C	2.0370800	1.7765020	2.6978260
C	-2.7573130	1.4859850	-4.2120520	C	2.6939750	2.0980380	4.0398630
C	-2.6509340	2.9919880	-4.4858620	C	2.6523890	3.6288820	4.0698180
C	-2.9418470	3.6671930	-3.1235270	C	2.9754420	4.0584570	2.6223420
N	-2.2326390	1.7336280	0.6268650	N	1.7866240	1.7649190	-0.7626400
P	-1.6143560	0.1412990	0.5032980	P	1.7027390	0.1764910	-0.6314140
N	-2.9351870	-0.8955930	0.2991510	N	2.8714090	-0.6407520	0.0784590
P	-4.4830910	-0.8805830	0.0829470	P	4.4479320	-0.7953890	0.2265110
C	-4.8994530	-1.0860050	-1.7082360	C	4.8511310	-0.6384060	1.9991450
C	-6.2983740	-1.6388160	-2.1112690	C	6.2858610	-0.9485780	2.4513890
C	-6.0389960	-2.6478990	-3.2548980	C	6.1722950	-0.9685370	3.9895460
C	-4.6254050	-2.3284110	-3.7541020	C	4.6981530	-1.3359320	4.3074210
C	-3.8809190	-1.9568540	-2.4722520	C	4.0021900	-1.5034800	2.9438860
C	-1.4064750	-0.1278790	2.3448590	C	1.4876560	-0.4117180	-2.3318560
C	-1.0043130	-1.5302260	2.8396070	C	1.1250410	-1.8816970	-2.6168260
C	0.5590800	-1.7755670	2.7697340	C	-0.4325230	-2.1560430	-2.5652620
C	1.3988330	-0.5700830	2.2702070	C	-1.3108680	-0.9119550	-2.2782110
C	0.9698700	0.4740960	3.2994220	C	-0.8618790	0.0135170	-3.4112860
C	-0.5738530	0.7313110	3.3095710	C	0.6743150	0.2964420	-3.4239630
C	-1.4284590	-1.3714680	4.3239900	C	1.5969460	-1.9156700	-4.0957760
C	-1.0687270	-2.5335050	5.2423340	C	1.2952150	-3.2110850	-4.8353960
C	0.4416970	-2.8188160	5.1514550	C	-0.2158280	-3.4992350	-4.7799940
C	1.1032480	-1.8021100	4.2292020	C	-0.9279500	-2.3855730	-4.0239390
C	-0.6818180	-0.0667130	4.6614620	C	0.8369410	-0.6881740	-4.6371800
C	0.8498450	-0.3358020	4.6305800	C	-0.6900200	-0.9793220	-4.6097420
P	1.6810470	0.0719390	0.5235150	P	-1.8184570	-0.2175090	-0.6692840
N	2.0844650	-1.4050670	-0.2495610	N	-1.8699700	-1.4076360	0.4017420
P	1.3805240	-1.9245460	-1.5749270	P	-1.2631350	-1.8503430	1.8112920

C	2.5265030	-3.0656860	-2.4527250	C	-2.4661160	-2.9377450	2.6537070
C	3.7418220	-2.3730870	-3.1394750	C	-3.7103880	-2.2077680	3.2460480
C	4.9920870	-3.1008810	-2.6131060	C	-4.9373300	-2.9824340	2.7286770
C	4.4825480	-4.4727610	-2.1709640	C	-4.3983930	-4.3606050	2.3407970
C	3.1404800	-4.1369530	-1.5182250	C	-3.0306540	-4.0452440	1.7310240
N	3.1325420	0.8711980	0.8868710	N	-3.1494630	0.6082740	-0.9677300
P	4.6883810	0.6709540	0.8350130	P	-4.7384800	0.5212740	-0.9207040
C	5.2933850	0.4204150	-0.8836920	C	-5.3148830	0.4432790	0.8105090
C	6.6983980	-0.1449780	-1.1541000	C	-6.7371340	-0.0636000	1.1058050
C	6.9084230	0.0739520	-2.6725350	C	-6.9387720	0.2695850	2.6027550
C	5.8493760	1.1195520	-3.1086810	C	-5.8767780	1.3432940	2.9568290
C	5.2439980	1.6501500	-1.8022150	C	-5.2368070	1.7466190	1.6203690
C	-0.1771850	-2.8972860	-1.3379260	C	0.3118220	-2.7858870	1.6533030
C	-0.6613590	-3.7179460	-2.5686230	C	0.7682750	-3.5142120	2.9498900
C	-0.4171360	-5.1864380	-2.2075020	C	0.5015430	-5.0004490	2.6950600
C	-0.6905880	-5.2257650	-0.7034460	C	0.8077390	-5.1649270	1.2054120
C	-0.0768760	-3.9210590	-0.1714510	C	0.2577920	-3.8850300	0.5533670
C	0.8494320	-0.5668110	-2.7058550	C	-0.8467400	-0.3991700	2.8550410
C	0.4011190	-0.9420720	-4.1401760	C	-0.5446270	-0.6658980	4.3517330
C	0.7988700	0.2655740	-4.9992950	C	-0.9700900	0.6310640	5.0493830
C	2.1357640	0.6930430	-4.3912220	C	-2.2474360	1.0199560	4.3040040
C	1.8903510	0.5759330	-2.8824650	C	-1.9059420	0.7430860	2.8334060
C	5.4478560	2.1592300	1.5901360	C	-5.3443330	1.9906880	-1.8046090
C	4.8545130	3.5105390	1.0996360	C	-4.6243720	3.3123900	-1.4036030
C	6.0569490	4.4708910	0.9695790	C	-5.7396830	4.3759870	-1.3211850
C	7.1773100	3.8150090	1.7859510	C	-6.9190900	3.7759610	-2.0958330
C	6.9771710	2.3243810	1.4979130	C	-6.8549340	2.2904660	-1.7268980
C	5.4745460	-0.7183350	1.7599000	C	-5.4666790	-0.9338370	-1.7477510
C	5.1837780	-0.6936640	3.2753930	C	-5.0721420	-1.0573730	-3.2382870
C	5.2375600	-2.1687410	3.6881160	C	-5.0306040	-2.5700720	-3.4872660
C	4.5768640	-2.8974630	2.5123180	C	-4.4514660	-3.1459390	-2.1905470
C	5.0153940	-2.1168900	1.2563700	C	-5.0491570	-2.2740840	-1.0708140
C	-0.1679580	3.1372320	-0.7764420	C	0.0079600	3.4613880	0.5898170
C	0.2380730	4.0321490	-1.9639250	C	-0.2252020	4.6593500	1.5388570
C	1.6547990	4.4798530	-1.5929630	C	-1.6087080	5.1726960	1.1283360
C	1.5082680	4.8165580	-0.1075920	C	-1.5561890	5.0793150	-0.3975110
C	0.6267150	3.6834560	0.4504500	C	-0.8923330	3.7182560	-0.6614240
C	-5.3178200	-2.2321700	1.0392500	C	4.9516330	-2.4449180	-0.3782700
C	-4.7313390	-2.3132130	2.4816110	C	4.8939160	-2.5776970	-1.9129170
C	-4.1523440	-3.7333600	2.6342610	C	4.7938030	-4.0882740	-2.1276210
C	-3.8907500	-4.1874780	1.1970570	C	3.8121550	-4.5432490	-1.0404640
C	-5.1139370	-3.6510740	0.4531200	C	4.0420920	-3.5882650	0.1564520
C	-5.3753280	0.6633330	0.5370910	C	5.4263960	0.5102480	-0.6068830
C	-6.8901950	0.7260490	0.1909350	C	6.9611450	0.2563680	-0.7151640
C	-7.5679010	1.3960210	1.4087120	C	7.3545190	0.7304470	-2.1329690
C	-6.4261720	2.0697880	2.1776440	C	6.1936440	1.6195600	-2.5899800
C	-5.2664340	1.0839650	2.0140580	C	4.9681800	0.8967200	-2.0296310
H	1.5931630	1.3709150	3.3086350	H	-1.4986430	0.8863310	-3.5617220
H	-2.4502110	0.0302230	2.6534170	H	2.5355990	-0.2882770	-2.6412400
H	2.4326860	-0.8449220	2.5046610	H	-2.3228140	-1.2361020	-2.5509060
H	-2.5120800	-1.2119480	4.3611140	H	2.6771130	-1.7353300	-4.1243880
H	-1.5553020	-2.3278390	2.3328550	H	1.6715670	-2.5877220	-1.9871450
H	-1.0651650	0.4596090	5.5400960	H	1.2395710	-0.2818310	-5.5661910
H	1.4248030	-0.0009150	5.4984140	H	-1.2457970	-0.7729210	-5.5256840
H	2.1808560	-1.9992440	4.2018890	H	-2.0000180	-2.6080950	-4.0008920
H	0.7890240	-2.6960110	2.2271680	H	-0.6649070	-2.9943910	-1.9077290
H	-0.8710420	1.7824590	3.3258550	H	0.9559890	1.3387150	-3.5730180
H	0.9116890	-2.7727300	6.1406690	H	-0.6404480	-3.5732670	-5.7863640
H	0.6285900	-3.8277020	4.7656980	H	-0.4174980	-4.4540050	-4.2836560
H	-1.3622290	-2.2866670	6.2694740	H	1.6438620	-3.1232390	-5.8694430
H	-1.6522570	-3.4168990	4.9585710	H	1.8693340	-4.0242840	-4.3783930
H	0.6243430	-5.4607270	-2.4116990	H	-0.5475980	-5.2423810	2.8986730
H	-1.0518900	-5.8692440	-2.7795830	H	1.1125290	-5.6444620	3.3321220
H	-1.7737730	-5.2242410	-0.5344660	H	1.8921830	-5.2232740	1.0619870
H	-0.2822750	-6.1132090	-0.2111780	H	0.3708420	-6.0682020	0.7726960
H	-0.5981730	-3.5571970	0.7164290	H	0.8460820	-3.5844060	-0.3153110
H	0.9630260	-4.0792640	0.1289720	H	-0.7630210	-4.0442020	0.1959280
H	-0.1877310	-3.4384100	-3.5110650	H	0.2934010	-3.1511870	3.8634680
H	-1.7338260	-3.5529660	-2.7038990	H	1.8429300	-3.3529470	3.0740940
H	0.0662880	1.0737500	-4.8823530	H	-0.2046000	1.4041200	4.9089420
H	-0.6669980	-1.1729500	-4.1915550	H	0.5047470	-0.9195910	4.5184730
H	0.9449620	-1.8202930	-4.5082510	H	-1.1527720	-1.4918220	4.7375540
H	4.3280080	-5.1210800	-3.0422690	H	-4.2745360	-4.9850800	3.2328380
H	1.4592700	1.5078230	-2.5063560	H	-1.4676730	1.6389340	2.3846830
H	5.1675960	-4.9848770	-1.4890990	H	-5.0516880	-4.8934310	1.6451660
H	2.4482460	1.6985150	-4.6872060	H	-2.5532870	2.0555510	4.4691660
H	3.3100830	-3.7058690	-0.5266700	H	-3.1543590	-3.6605910	0.7135170
H	2.8010070	0.4140040	-2.3053770	H	-2.7811060	0.5056390	2.2268470
H	2.4920540	-5.0081430	-1.3985500	H	-2.3756720	-4.9176690	1.6790100
H	5.3792760	-2.5702830	-1.7371930	H	-5.3377180	-2.4892320	1.8372670
H	3.6557600	-2.4765630	-4.2253840	H	-3.6581840	-2.2318800	4.3374120
H	5.7943140	-3.1508910	-3.3544610	H	-5.7409130	-3.0258780	3.4675660
H	3.7969300	-1.3043230	-2.9297860	H	-3.7671500	-1.1559250	2.9622250
H	6.3688830	4.5385930	-0.0793960	H	-6.0324920	4.5309540	-0.2765610
H	5.8212270	5.4850580	1.3027060	H	-5.4184840	5.3436010	-1.7143650
H	4.3152410	3.4084120	0.1551520	H	-4.0779520	3.2252670	-0.4613420
H	4.1800810	-0.2935950	3.4658170	H	-4.0823240	-0.6206780	-3.4188020

H	5.8958650	-0.0691950	3.8230120	H	-5.7763330	-0.5353960	-3.8904270
H	4.8491550	-3.9556970	2.4636000	H	-4.6840300	-4.2048900	-2.0559530
H	6.2824530	-2.4869320	3.7877760	H	-6.0486210	-2.9461090	-3.6379730
H	4.7403700	-2.3588810	4.6445870	H	-4.4460900	-2.8327850	-4.3734900
H	3.4868940	-2.8485030	2.6125450	H	-3.3589070	-3.0584000	-2.1870780
H	5.8453840	-2.6218870	0.7516470	H	-5.9286000	-2.7524440	-0.6317000
H	4.1845470	-2.0379920	0.5469350	H	-4.3233740	-2.1292270	-0.2652410
H	8.1742110	4.1732450	1.5144370	H	-7.8781230	4.2332050	-1.8402790
H	7.3364050	2.1126590	0.4857360	H	-7.2331860	2.1637940	-0.7075800
H	7.0307940	4.0085450	2.8555270	H	-6.7657150	3.8956220	-3.1746090
H	7.4540830	0.4059850	-0.5872330	H	-7.4712220	0.4702940	0.4959160
H	6.7911340	-1.1947080	-0.8660750	H	-6.8575400	-1.1282970	0.8931230
H	6.7720800	-0.8628200	-3.2198520	H	-6.7977460	-0.6253520	3.2147960
H	7.9249290	0.4191590	-2.8790270	H	-7.9544600	0.6266980	2.7864600
H	6.2726930	1.9163830	-3.7256310	H	-6.3034060	2.2015220	3.4803670
H	4.2371900	2.0575900	-1.9269950	H	-4.2205870	2.1347800	1.7263360
H	5.8762500	2.4471390	-1.3920320	H	-5.8381970	2.5226440	1.1342660
H	7.5146230	1.6662830	2.1865310	H	-7.4444000	1.6466500	-2.3844190
H	5.0639210	0.6366690	-3.7006220	H	-5.1118620	0.9147690	3.6126580
H	0.8587250	0.0235650	-6.0640980	H	-1.1171350	0.4985430	6.1236200
H	2.9199440	-0.0051160	-4.7087240	H	-3.0688800	0.3734700	4.6333670
H	4.1275350	3.8761360	1.8291480	H	-3.8830070	3.5637340	-2.1655780
H	4.5579580	-0.3099910	-1.2497680	H	-4.6042940	-0.2739770	1.2457560
H	6.5554400	-0.6115970	1.6060200	H	-6.5523020	-0.8060810	-1.6709240
H	5.1925660	2.0467450	2.6513080	H	-5.0951240	1.7691070	-2.8495320
H	1.9400720	-3.5754310	-3.2232740	H	-1.9180770	-3.4115270	3.4742980
H	-0.9196190	-2.1327110	-1.0832730	H	1.0546650	-2.0271630	1.3802310
H	-0.0026350	-0.1718060	-2.1418950	H	0.0717240	-0.0356530	2.3841380
H	2.0147120	5.3159890	-2.1990350	H	-1.8094280	6.1812700	1.4973340
H	2.3544160	3.6423810	-1.7186230	H	-2.3874750	4.5065880	1.5205820
H	2.4645420	4.8999050	0.4141770	H	-2.5368230	5.1627940	-0.8717880
H	0.9934140	5.7802200	-0.0078970	H	-0.9277320	5.8870840	-0.7906320
H	1.2418000	2.8730130	0.8525330	H	-1.6504540	2.9315000	-0.7238880
H	-0.0102940	4.0388450	1.2616190	H	-0.3440300	3.6995280	-1.6047400
H	-0.3999310	4.9219020	-2.0294230	H	0.5067500	5.4548840	1.3609170
H	0.1763840	3.5121360	-2.9253350	H	-0.1530860	4.3722700	2.5914390
H	-3.8087860	1.1719750	-4.1829320	H	3.7324080	1.7442230	4.0425650
H	-2.3152020	0.4136550	-2.3187450	H	2.1938230	0.7495800	2.3585230
H	-1.0574600	1.5046170	-2.8988840	H	0.9577320	1.9481150	2.7886770
H	-3.7927120	5.7448900	3.0742880	H	3.4426040	5.2651630	-3.8390910
H	-3.9646010	4.0514110	-3.0932570	H	4.0315910	4.3183260	2.5224950
H	-4.2696630	4.0899570	2.6851200	H	3.8535000	3.6333990	-3.3092770
H	-3.3249220	3.3366170	-5.2742320	H	3.3451850	4.0641390	4.7930520
H	-2.0055410	3.8200180	2.1655290	H	1.6509990	3.5664500	-2.5467640
H	-2.2826340	4.5211060	-2.9561440	H	2.4061530	4.9429940	2.3312870
H	-1.7600010	5.5379240	1.8211180	H	1.5074900	5.3221060	-2.4264810
H	-5.7474590	5.4023050	1.2489890	H	5.5382810	4.9726670	-2.1645870
H	-4.4692540	4.8818260	-1.0435410	H	4.4568690	4.8257960	0.2768890
H	-4.4306170	6.5026870	0.8167960	H	4.3500710	6.2194350	-1.7592850
H	-4.6533110	3.5440810	0.0978190	H	4.4234750	3.3560910	-0.7067120
H	-3.2542070	-3.7432900	3.2585280	H	4.4606530	-4.3456440	-3.1363130
H	-4.8874720	-4.3996020	3.1006200	H	5.7763620	-4.5469530	-1.9710830
H	-3.9267200	-1.5830080	2.5983720	H	3.9863300	-2.0992750	-2.2988930
H	-7.3281110	-0.2579440	0.0049850	H	7.2342600	-0.7908880	-0.5617040
H	-7.0399660	1.3079540	-0.7232260	H	7.4831890	0.8328240	0.0515570
H	-6.1715120	3.0273420	1.7072020	H	6.2764760	2.6161760	-2.1388430
H	-8.3594300	2.0916470	1.1171280	H	8.3181390	1.2445640	-2.1416780
H	-8.0258930	0.6285150	2.0428940	H	7.4369210	-0.1309040	-2.8044350
H	-6.6745880	2.2719840	3.2236280	H	6.1491720	1.7461160	-3.6743610
H	-4.2806190	1.5070490	2.2187790	H	4.0607070	1.5052670	-2.0177550
H	-5.4243730	0.2312750	2.6839700	H	4.7718710	0.0089430	-2.6384550
H	-2.9904870	-3.6957630	0.8108260	H	2.7842540	-4.4450570	-1.4066740
H	-5.0067160	-3.6774910	-0.6322080	H	3.0893600	-3.1922800	0.5185470
H	-3.7681970	-5.2701520	1.0988590	H	3.9518190	-5.5903090	-0.7623880
H	-6.8148710	-2.1257800	-1.2798070	H	6.5862560	-1.9360680	2.0830870
H	-6.9425770	-0.8206050	-2.4429870	H	7.0185570	-0.2225080	2.0942100
H	-6.7961020	-2.5899480	-4.0413140	H	6.4103120	0.0209460	4.3901430
H	-6.0601260	-3.6703530	-2.8605290	H	6.8812410	-1.6729490	4.4302450
H	-4.1617920	-3.1607430	-4.2917450	H	4.6170520	-2.2462130	4.9057430
H	-2.9379580	-1.4372920	-2.6483740	H	2.9496380	-1.2113910	2.9557990
H	-3.6349940	-2.8601220	-1.9040930	H	4.0512670	-2.5497820	2.6272850
H	-5.9905080	-4.2624030	0.6990440	H	4.5267120	-4.0977390	0.9925270
H	-4.6473120	-1.4648800	-4.4306790	H	4.2193240	-0.5343020	4.8771060
H	-2.2484200	0.8744410	-4.9630410	H	2.1817240	1.6323040	4.8858580
H	-1.6284640	3.2247530	-4.8063280	H	1.6430360	3.9569300	4.3425740
H	-5.4934290	-2.0954460	3.2348130	H	5.7549870	-2.1232810	-2.4094710
H	-4.8065340	-0.0636380	-2.1016380	H	4.6375080	0.4188510	2.2167530
H	-4.8401670	1.4229350	-0.0421980	H	5.2706870	1.3871340	0.0352330
H	-6.3828180	-1.9817150	1.0656910	H	5.9822850	-2.6159460	-0.0506800
H	-2.1914210	5.2485490	-0.4193010	H	2.1760270	5.3542830	-0.2250340
H	0.1958510	2.1147070	-0.9487280	H	-0.3504270	2.5534140	1.0854680
H	-3.7765240	2.2244040	-1.7482640	H	3.6266360	2.3959500	1.3915430
				H	-0.9254140	0.7964300	-0.2953700
				H	0.6073880	-0.2323620	0.1360650
**II(Pf$_2$)$_2$**				**II(Pf$_2$)$_2$ (diprotonated)**			
C	-1.3837320	-0.4467160	2.0383120	C	-1.4998370	-0.9795940	1.9887340

C	-0.4118720	-1.4763960	2.6624590
C	-0.8898110	-1.3400290	4.1349850
C	-0.7691610	0.1879390	4.3016390
C	-1.0005780	0.7854130	2.8685300
C	0.7317530	0.5877030	4.2307130
C	1.5883270	-0.6755490	4.0184040
C	1.3942290	-1.7418640	5.0884590
C	-0.0928900	-2.1338110	5.1641400
C	1.1161490	-1.0671310	2.5902920
C	1.3789670	0.2947860	1.9054960
C	0.5107300	1.1860610	2.7983160
P	1.4411240	0.4679670	0.0407690
N	2.6987490	-0.5960800	-0.2628890
P	4.1958340	-1.0057030	-0.3560330
C	5.4557620	0.3017560	0.0390160
C	5.1446800	1.0812630	1.3198780
P	-1.7811220	-0.3442860	0.1959180
N	-3.5039060	-0.4235730	0.3336640
P	-4.4887410	0.7696820	-0.0015460
C	-4.0772790	2.3271840	0.9262670
C	-4.6501880	3.6445220	0.3946840
N	-1.3463960	-1.9335660	-0.1634300
P	-1.4440020	-2.8640360	-1.4181770
C	-2.8488080	-4.0719120	-1.3106870
C	-4.1931940	-3.3657180	-1.5196510
C	-1.6844520	-1.9882460	-3.0339360
C	-0.5940590	-0.9337950	-3.2412900
C	0.1160440	-3.8584430	-1.5938290
C	-0.0544270	-5.2234940	-2.2713960
C	-1.8313370	-2.8872780	-4.2642040
N	2.1269000	2.0413790	0.0051000
P	1.5870650	3.2487090	-0.8658460
C	3.0029960	4.2372280	-1.5464550
C	4.2467850	4.1401320	-0.6583200
C	0.5381070	2.7495870	-2.3154000
C	-0.1507550	3.8886330	-3.0730380
C	0.5390550	4.4229610	0.1136820
C	1.2899560	4.8739410	1.3717500
C	1.3237720	1.8544450	-3.2795600
C	-6.1955260	0.1820590	0.4011240
C	-7.2861040	1.2484560	0.2628770
C	-4.6192450	1.3178450	-1.7776220
C	-3.2621340	1.7777600	-2.3241980
C	-6.2768040	-0.5413310	1.7493340
C	-5.2108630	0.2192750	-2.6671080
C	-4.3534570	2.1699740	2.4258410
C	0.8500530	-3.9939610	-0.2562350
C	-2.8200940	-4.7784770	0.0484190
C	4.6230530	-1.5188700	-2.0905610
C	4.7385270	-0.3111130	-3.0269520
C	4.4988450	-2.5286340	0.6696660
C	5.6209120	-3.4681500	0.2135720
C	3.5552320	-2.4942650	-2.5964050
C	4.6345130	-2.1934300	2.1590370
C	6.9054970	-0.1931550	0.0057190
C	-0.7923730	3.7496570	0.4629990
C	2.6681410	5.6990080	-1.8687090
H	0.6961470	2.2544370	2.6795280
H	-2.3821760	-0.7424090	2.3880920
H	2.4201020	0.5540720	2.1442800
H	-1.9406070	-1.6451260	4.1924240
H	-0.5582960	-2.4758900	2.2500620
H	-1.3461150	0.6008170	5.1339590
H	1.0971400	1.2546100	5.0164960
H	2.6495000	-0.4032380	3.9788680
H	1.7082940	-1.8767690	2.1552620
H	-1.6942680	1.6261510	2.7910510
H	1.7513400	-1.3604060	6.0521620
H	2.0191560	-2.6096650	4.8471110
H	-0.4999920	-1.9418060	6.1638970
H	-0.2271430	-3.2049590	4.9752150
H	-2.5470770	0.9512030	-2.3450230
H	-3.3839810	2.1603500	-3.3429670
H	-2.8152380	2.5725850	-1.7201180
H	-5.3036250	2.1743780	-1.7876250
H	-6.2697830	0.0425950	-2.4611070
H	-4.6773840	-0.7259460	-2.5321530
H	-5.1242200	0.5070470	-3.7196000
H	-2.9873130	2.3539190	0.7965270
H	-4.3263020	3.8593700	-0.6264130
H	-5.7435210	3.6548960	0.4127290
H	-4.3028170	4.4687490	1.0267480
H	-5.4220960	2.2629630	2.6434060
H	-4.0055540	1.2042270	2.8022400
H	-3.8349530	2.9581230	2.9816460
H	-6.3578730	-0.5788080	-0.3721420
H	-7.1996930	2.0089770	1.0449580

C	-0.5167030	-2.1244310	2.3321770
C	-0.9746170	-2.3172890	3.8053850
C	-0.8467600	-0.8657000	4.3106060
C	-1.1094350	0.0439820	3.0630820
C	0.6510300	-0.4506240	4.2988100
C	1.5022770	-1.6358870	3.8011560
C	1.3209180	-2.9114970	4.6113940
C	-0.1619430	-3.3231270	4.6093500
C	1.0109350	-1.7098340	2.3265730
C	1.2618770	-0.2235940	1.9773080
C	0.3965640	0.4583510	3.0434630
P	1.5892380	0.4100510	0.2973490
N	2.7764700	-0.4532790	-0.3065030
P	4.3028240	-0.8237180	-0.4921130
C	5.4566480	0.3970560	0.2653320
C	5.1186490	0.7615750	1.7148800
P	-1.8571110	-0.4555900	0.2845640
N	-3.4413130	-0.2646930	0.1421330
P	-4.4952730	0.9322820	0.0546410
C	-4.0770610	2.2838100	1.2406840
C	-4.6787670	3.6565430	0.9209010
N	-1.1586590	-1.4750720	-0.7147930
P	-1.4548800	-2.4849610	-1.9094520
C	-2.8428240	-3.6496100	-1.5729290
C	-4.2207480	-2.9994640	-1.7441300
C	-1.8437200	-1.5725960	-3.4579230
C	-0.7904350	-0.4923310	-3.7196010
C	0.0810850	-3.4656660	-2.2252160
C	-0.1908600	-4.8568010	-2.8136300
C	-2.0523180	-2.4730090	-4.6789660
N	1.7859160	1.9866520	0.5176860
P	1.5487400	3.3134830	-0.3405710
C	3.1673510	3.9868070	-0.9064030
C	4.2275750	3.9831910	0.2029560
C	0.4843300	3.0178380	-1.8275970
C	-0.2284830	4.2524310	-2.3940330
C	0.6569970	4.5217000	0.7183330
C	1.4643290	4.8815450	1.9707290
C	1.2459700	2.2686200	-2.9272660
C	-6.1455210	0.1873560	0.3589630
C	-7.2746970	1.2219600	0.4037090
C	-4.5693650	1.6975170	-1.6211950
C	-3.1751860	2.1715540	-2.0445050
C	-6.1683940	-0.7602120	1.5633010
C	-5.1670010	0.7384420	-2.6565700
C	-4.3645810	1.8685310	2.6891140
C	0.9755180	-3.5520970	-0.9859050
C	-2.6914800	-4.2553340	-0.1723610
C	4.6821890	-0.8658380	-2.2936960
C	4.7611210	0.5443560	-2.8868490
C	4.5687360	-2.5386140	0.1370060
C	5.7481370	-3.2966750	-0.4831810
C	3.6194080	-1.7114570	-3.0078360
C	4.6117750	-2.5952600	1.6679330
C	6.9296870	0.0012960	0.1099740
C	-0.7107680	3.9436820	1.1058660
C	3.0778900	5.3544520	-1.5902310
H	0.5884900	1.5265130	3.1488030
H	-2.4990440	-1.3494490	2.2570440
H	2.2920820	-0.0279880	2.3151860
H	-2.0245400	-2.6275590	3.8072770
H	-0.6747190	-3.0120400	1.7177670
H	-1.4075840	-0.6520320	5.2217520
H	1.0318210	0.0305720	5.2009820
H	2.5633520	-1.3640140	3.8084030
H	1.5946980	-2.4028690	1.7172300
H	-1.8058440	0.8737880	3.1890490
H	1.6822780	-2.7406770	5.6307300
H	1.9506410	-3.6985760	4.1834400
H	-0.5597620	-3.3704400	5.6283240
H	-0.2941630	-4.3193500	4.1749000
H	-2.4907260	1.3240520	-2.1537420
H	-3.2353920	2.6729960	-3.0136240
H	-2.7424540	2.8826640	-1.3343980
H	-5.2251680	2.5706160	-1.5336620
H	-6.2386250	0.5874800	-2.5084860
H	-4.6772470	-0.2390610	-2.6247360
H	-5.0267530	1.1516630	-3.6588340
H	-2.9876690	2.3586060	1.1241410
H	-4.3447160	4.0450360	-0.0437860
H	-5.7715310	3.6354660	0.9234790
H	-4.3597400	4.3671690	1.6885190
H	-5.4332850	1.9327050	2.9110670
H	-4.0313730	0.8501140	2.9052480
H	-3.8435750	2.5473870	3.3696840
H	-6.2789390	-0.4336780	-0.5352420
H	-7.2142800	1.8404020	1.3037050

H	-7.2633500	1.7532520	-0.7071780	H	-7.2770370	1.8819670	-0.4685030
H	-8.2704870	0.7823050	0.3692440	H	-8.2362940	0.7028310	0.4268450
H	-7.2187220	-1.0963360	1.8075330	H	-7.0824010	-1.3590720	1.5220660
H	-5.4463860	-1.2413690	1.8603420	H	-5.3114460	-1.4377630	1.5484360
H	-2.6304820	-1.4549230	-2.8765020	H	-2.7889470	-1.0684700	-3.2240440
H	-0.5999040	-5.9209690	-1.6279560	H	-0.6435550	-5.5206770	-2.0712010
H	-0.5817850	-5.1647710	-3.2263070	H	-0.8389090	-4.8327260	-3.6926330
H	0.9315620	-5.6585150	-2.4639230	H	0.7599500	-5.3025150	-3.1176230
H	1.0113390	-3.0163640	0.2013800	H	1.2765230	-2.5613800	-0.6418180
H	-0.8835050	-3.3777050	-4.5076630	H	-1.1149150	-2.9468530	-4.9851590
H	-2.1149600	-2.2813470	-5.1309260	H	-2.4026760	-1.8668340	-5.5186360
H	-2.5939610	-3.6609640	-4.1369740	H	-2.7946860	-3.2565590	-4.5034620
H	0.3694110	-1.3958580	-3.4853490	H	0.1642810	-0.9225800	-4.0386260
H	-0.4566730	-0.3099140	-2.3523610	H	-0.6050830	0.1097810	-2.8268110
H	-2.7073640	-4.8174660	-2.1014940	H	-2.7411000	-4.4484790	-2.3153010
H	-2.9247620	-4.0467070	0.8542280	H	-2.8165970	-3.4885810	0.5982140
H	-3.6477180	-5.4917940	0.1167970	H	-3.4647450	-5.0128860	-0.0203650
H	-1.8891150	-5.3286060	0.2134530	H	-1.7202020	-4.7346990	-0.0227480
H	-4.3209970	-3.0274470	-2.5524370	H	-4.4001420	-2.6731560	-2.7720470
H	-4.2783220	-2.4966780	-0.8554360	H	-4.3322390	-2.1427680	-1.0733290
H	-5.0136910	-4.0566990	-1.2987400	H	-4.9920030	-3.7339310	-1.4951550
H	0.2888460	-4.6149260	0.4495230	H	0.4814710	-4.0726450	-0.1602360
H	1.8208000	-4.4751290	-0.4235120	H	1.8743630	-4.1214070	-1.2445670
H	0.7415980	-3.2329770	-2.2433760	H	0.6173250	-2.8768020	-2.9799440
H	-0.8657680	-0.2879680	-4.0833690	H	-1.1370730	0.1725870	-4.5155920
H	-6.2531610	0.1592850	2.5877700	H	-6.1700600	-0.2174910	2.5108760
H	6.5987120	-2.9790130	0.2233320	H	6.7053850	-2.8092340	-0.2828620
H	5.6751110	-4.3223680	0.8967270	H	5.7908820	-4.2976550	-0.0443930
H	5.4495130	-3.8641720	-0.7902600	H	5.6432720	-3.4181990	-1.5634820
H	3.5415340	-3.0474220	0.5350960	H	3.6407670	-3.0237890	-0.1872620
H	5.6254730	-1.7886340	2.3877050	H	5.5806280	-2.2634940	2.0516700
H	4.5035990	-3.1009380	2.7573450	H	4.4613520	-3.6271460	1.9965200
H	3.8836060	-1.4684120	2.4803770	H	3.8303000	-1.9847970	2.1257790
H	5.3175510	1.0045500	-0.7921990	H	5.2729980	1.2932390	-0.3404540
H	5.2279940	0.4594140	2.2146570	H	5.2849150	-0.0690290	2.4030340
H	5.8619990	1.9038050	1.4217340	H	5.7675130	1.5848070	2.0285400
H	4.1401530	1.5088360	1.2671530	H	4.0860330	1.1034360	1.8182510
H	7.1240230	-0.8448830	0.8570630	H	7.1861540	-0.8375990	0.7630990
H	7.1445870	-0.7402340	-0.9111490	H	7.1897710	-0.2691860	-0.9171800
H	7.5866760	0.6616520	0.0671080	H	7.5604120	0.8463730	0.3985190
H	5.5912100	-2.0309370	-2.0614300	H	5.6593300	-1.3473640	-2.4050990
H	5.6375580	0.2792140	-2.8307380	H	5.6329090	1.0978840	-2.5295160
H	4.7877660	-0.6490190	-4.0669980	H	4.8340000	0.4789250	-3.9757280
H	3.8687510	0.3473950	-2.9315340	H	3.8625360	1.1210040	-2.6496160
H	2.5820510	-1.9946130	-2.6158300	H	2.6306220	-1.2578530	-2.8879610
H	3.7976900	-2.8322420	-3.6086440	H	3.8504430	-1.7663510	-4.0747870
H	-0.2302100	2.1237990	-1.8434050	H	-0.2876820	2.3435060	-1.4317550
H	2.1527220	5.5011980	1.1311350	H	2.3457270	5.4792670	1.7334290
H	0.6247900	5.4571930	2.0162760	H	0.8374560	5.4715600	2.6447940
H	1.6522600	4.0109860	1.9368850	H	1.7840730	3.9809660	2.5031560
H	-0.6363420	2.7473470	0.8741710	H	-0.5891050	3.0059170	1.6569530
H	1.7141780	0.9698070	-2.7710190	H	1.6936710	1.3381920	-2.5652420
H	2.1564250	2.3895180	-3.7485310	H	2.0353270	2.8876580	-3.3636160
H	0.6633310	1.5155400	-4.0832790	H	0.5544120	2.0098540	-3.7328410
H	-0.6823290	4.5828660	-2.4166860	H	-0.8352550	4.7735530	-1.6505890
H	-0.8851180	3.4663560	-3.7670700	H	-0.8978830	3.9304760	-3.1967850
H	3.2385720	3.7281760	-2.4893480	H	3.4761070	3.2409080	-1.6498000
H	3.5069970	6.1543200	-2.4042040	H	4.0843850	5.6796610	-1.8673580
H	1.7795400	5.8055550	-2.4943470	H	2.4801040	5.3308480	-2.5030330
H	2.5085440	6.2812800	-0.9561210	H	2.6597650	6.1124220	-0.9205940
H	4.4841210	3.1003470	-0.4324280	H	4.1442360	3.1024730	0.8406150
H	4.1048090	4.6635870	0.2921860	H	4.1511450	4.8716470	0.8333590
H	5.0989540	4.5997440	-1.1696070	H	5.2213190	3.9893500	-0.2538310
H	-1.3362750	4.3415330	1.2068910	H	-1.2400440	4.6513300	1.7491780
H	-1.4362710	3.6519510	-0.4155270	H	-1.3452490	3.7568540	0.2353190
H	0.3391210	5.3022200	-0.5088340	H	0.5098880	5.4239160	0.1140110
H	0.5658660	4.4623440	-3.6686510	H	0.4723600	4.9713750	-2.8223900
H	3.4592310	-3.3785230	-1.9580130	H	3.5698390	-2.7341780	-2.6221760
				H	0.5402280	0.1687690	-0.5927790
				H	-1.2320370	0.8065490	0.1439640
**$II(Pg_2)_2$**				**$II(Pg_2)_2$ (diprotonated)**			
C	-0.6056090	-1.5723430	5.3701980	C	-0.7390070	-1.5517390	5.4459110
C	-1.2158040	-0.7047830	4.2760890	C	-1.2603600	-0.6808490	4.3111240
C	-0.8197580	-1.0839810	2.8217860	C	-0.8162720	-1.1116590	2.8842420
C	0.7574810	-1.0547670	2.6972370	C	0.7631080	-1.1520840	2.8323590
C	1.3455590	-0.6676270	4.0834330	C	1.3065540	-0.7608020	4.2371700
C	0.9286570	-1.5646530	5.2425490	C	0.7970600	-1.6182540	5.3872290
C	1.3328380	0.1455540	1.9008820	C	1.4308010	0.0183600	2.0628150
C	0.7144330	1.2920810	2.7149520	C	0.8301730	1.2125930	2.8169230
C	-0.8473100	1.2628010	2.8167860	C	-0.7318190	1.2530400	2.8381920
C	-1.5224430	0.0862240	2.1054880	C	-1.4344180	0.0861090	2.1329750
C	0.8211460	0.7791600	4.1915380	C	0.8422270	0.7094650	4.2987600
C	-0.7303810	0.7573480	4.2991690	C	-0.7112690	0.7589530	4.3299880
P	-1.8509370	0.0887850	0.2704860	P	-1.9305420	0.0230170	0.3896100
N	-3.0569300	1.2957870	0.2684930	N	-2.8372380	1.2946800	0.0609470
P	-2.9783460	2.7853380	-0.1744760	P	-2.7898100	2.8381940	-0.2351880

N	-1.5943880	3.4927220	-0.8200980
C	-0.4450600	3.7554360	0.0415910
P	1.5007480	0.3283360	0.0266110
N	1.4858150	-1.2538710	-0.5540690
P	2.2654480	-2.5925970	-0.4419970
N	1.5489450	-3.7094470	0.5873390
C	0.0979500	-3.7855480	0.6145650
N	-2.7358570	-1.3654650	0.2835920
P	-3.2890250	-2.0875080	-0.9844060
N	-2.5165250	-2.0254140	-2.4797100
C	-2.5247650	-0.7744090	-3.2314090
N	-3.3028300	-3.7392750	-0.6663800
C	-3.9196410	-4.6680170	-1.5980630
N	-4.8218180	-1.5064820	-1.4038010
C	-5.6944030	-1.0939310	-0.3114500
C	-3.2536750	-4.2266330	0.7012690
N	3.1734520	0.7707150	0.0737550
P	3.7930970	1.8665910	-0.8560230
N	3.1891730	3.4214050	-0.5753640
C	3.4311510	4.5616610	-1.4477800
N	5.4668130	1.8148710	-0.6524670
C	6.0254900	1.3326200	0.6014140
N	3.6577340	1.7837890	-2.5404200
C	2.3249600	1.9513770	-3.1148090
C	6.2643040	2.8709050	-1.2606010
N	2.3026690	-3.2806650	-1.9782050
C	2.8174380	-4.6210960	-2.1868410
N	3.8639290	-2.6433980	0.1103980
C	4.8907070	-2.1424900	-0.7984220
C	2.2451020	-2.4789340	-3.1844570
C	4.1079430	-2.2212490	1.4883580
C	2.2001460	-4.8708480	1.1669820
N	-4.0889890	3.0591920	-1.4119030
C	-4.4014380	4.4022260	-1.8673720
N	-3.2447540	3.7921470	1.1546500
C	-3.2338310	5.2429770	1.0662410
C	-5.0978080	2.0688030	-1.7403380
C	-3.9951180	3.2900390	2.2935390
C	-1.1997580	3.1516580	-2.1810980
C	-5.5528820	-1.9912230	-2.5652360
C	-1.2706380	-2.7680700	-2.6535920
C	4.4135290	0.7388510	-3.2235760
C	3.0154740	3.8124550	0.8156840
H	1.1302130	2.2802810	2.5058030
H	-2.5643690	0.0761500	2.4585700
H	2.4010730	0.1570840	2.1457850
H	-2.3075700	-0.7427620	4.3609730
H	-1.2161900	-2.0557370	2.5200380
H	-1.1729960	1.3693920	5.0899370
H	1.3530470	1.3998570	4.9178320
H	2.4392930	-0.6704190	4.0126650
H	1.1307490	-2.0152910	2.3385930
H	-1.3405290	2.2232510	2.6492940
H	1.3996160	-1.2081630	6.1660310
H	1.3137160	-2.5762050	5.0680260
H	-0.9214680	-1.1957980	6.3501910
H	-1.0019610	-2.5906970	5.2866780
H	3.2341090	-2.3752080	-3.6578310
H	5.0691690	-2.6273450	1.8225390
H	1.5731280	-2.9483570	-3.9138100
H	4.1292280	-1.1274600	1.5702750
H	3.3271420	-2.6030840	2.1485790
H	2.2523440	-5.1106120	-2.9883990
H	3.8798650	-4.6202770	-2.4746290
H	2.7092130	-5.2255530	-1.2870990
H	4.9204930	-1.0453640	-0.8139970
H	5.8668770	-2.5185720	-0.4729000
H	4.7123500	-2.5061120	-1.8122950
H	1.8251320	-5.8049580	0.7236890
H	1.9994700	-4.9107590	2.2460700
H	3.2780400	-4.8195870	1.0152610
H	-0.3464660	-2.8334430	0.3214400
H	-0.2349180	-4.0135370	1.6343970
H	3.6463350	4.2299210	-2.4627670
H	4.2739600	5.1734360	-1.0953060
H	2.5382110	5.1986690	-1.4706080
H	2.1510670	4.4793940	0.9123050
H	3.8993170	4.3425660	1.2019830
H	2.8403300	2.9282180	1.4274090
H	5.3830320	0.5532250	1.0065500
H	7.0234220	0.9184360	0.4170500
H	6.1231530	2.1371010	1.3465770
H	5.8441980	3.1555030	-2.2267840
H	1.7408110	1.0204790	-3.0769760
H	7.2829140	2.5042980	-1.4265100
H	1.7700830	2.7187520	-2.5752890
H	6.3244830	3.7668550	-0.6244310

N	-1.4416340	3.5256710	-0.9211610
C	-0.2546210	3.8497310	-0.1359660
P	1.7162670	0.1262310	0.2641890
N	1.4104800	-1.2348610	-0.5324610
P	2.1588140	-2.6424990	-0.5224720
N	1.4222760	-3.7111830	0.5102490
C	-0.0344240	-3.7709260	0.5215940
N	-2.7004560	-1.3847560	0.2841300
P	-3.3545550	-1.9863090	-1.0339820
N	-2.6092030	-1.7159580	-2.4983320
C	-2.7255400	-0.4132500	-3.1544560
N	-3.2968150	-3.6414090	-0.8616380
C	-3.8241190	-4.5018800	-1.9167690
N	-4.8832950	-1.3496440	-1.2572780
C	-5.6838860	-1.0187090	-0.0794660
C	-3.3656190	-4.2417770	0.4669140
N	3.2045420	0.7387490	0.1579680
P	3.8298560	1.7981010	-0.8451920
N	3.1931230	3.3168940	-0.5392470
C	3.3629510	4.4432110	-1.4565740
N	5.4780640	1.7065610	-0.6673240
C	6.0968830	1.1457520	0.5282130
N	3.5807410	1.6286790	-2.4831050
C	2.2179210	1.7762390	-2.9896960
C	6.2939930	2.7255400	-1.3250730
N	2.0744430	-3.2191020	-2.0757180
C	2.4128300	-4.6100820	-2.3524510
N	3.7489390	-2.6890750	-0.0233030
C	4.7965880	-2.2864420	-0.9574620
C	2.0725620	-2.3666670	-3.2538820
C	4.0864160	-2.4007700	1.3698880
C	2.0573390	-4.9029500	1.0651500
N	-3.9823860	3.1372630	-1.3584340
C	-4.2883750	4.5139200	-1.7329970
N	-2.9428470	3.6834010	1.1996490
C	-2.8845140	5.1434660	1.2356800
C	-5.0940910	2.2142940	-1.5479790
C	-3.7816030	3.1326240	2.2611520
C	-1.1566150	3.2956180	-2.3342560
C	-5.6944310	-1.7550890	-2.4037300
C	-1.3066540	-2.3437340	-2.7303280
C	4.3812450	0.6795010	-3.2537980
C	3.1427560	3.7455220	0.8580130
H	1.3043280	2.1729310	2.6128610
H	-2.4762400	0.1324020	2.4896850
H	2.4877750	-0.0118400	2.3555010
H	-2.3545060	-0.6732750	4.3463990
H	-1.2454950	-2.0669830	2.5796370
H	-1.1644870	1.4075940	5.0812610
H	1.3675230	1.3181510	5.0361280
H	2.4001540	-0.8121130	4.2175910
H	1.1088620	-2.1331450	2.5016730
H	-1.1716830	2.2305630	2.6365020
H	1.2442070	-1.2640230	6.3216750
H	1.1425120	-2.6475750	5.2449550
H	-1.0801210	-1.1351810	6.3993940
H	-1.1805090	-2.5501490	5.3644520
H	3.0531110	-2.3586570	-3.7487070
H	4.9744900	-2.9749050	1.6500890
H	1.3354730	-2.7391100	-3.9725480
H	4.2958870	-1.3346050	1.5150710
H	3.2713910	-2.6911860	2.0339440
H	1.7686550	-4.9802330	-3.1554260
H	3.4588400	-4.7216240	-2.6661060
H	2.2445630	-5.2333640	-1.4751520
H	4.9504880	-1.1999640	-0.9495060
H	5.7319560	-2.7748870	-0.6699290
H	4.5499170	-2.6037830	-1.9713070
H	1.6207120	-5.8120870	0.6335890
H	1.9004550	-4.9300450	2.1493140
H	3.1278300	-4.9003490	0.8641500
H	-0.4664650	-2.8075240	0.2508750
H	-0.3728910	-4.0247210	1.5312310
H	3.5046480	4.0933950	-2.4780750
H	4.2248980	5.0608650	-1.1747670
H	2.4644420	5.0681410	-1.4221580
H	2.2741910	4.3937760	1.0116030
H	4.0449870	4.3059460	1.1352040
H	3.0532670	2.8812740	1.5151960
H	5.4352780	0.4078730	0.9760650
H	7.0357290	0.6578780	0.2483390
H	6.3153830	1.9251220	1.2697020
H	5.8162990	3.0667510	-2.2450940
H	1.6395810	0.8461020	-2.8946580
H	7.2631070	2.2918930	-1.5882540
H	1.6953070	2.5664550	-2.4510660
H	6.4648840	3.5907560	-0.6715660

H	3.9363950	-0.2455960	-3.1263240	H	3.9475620	-0.3262310	-3.2334800
H	5.4225540	0.6756420	-2.8173120	H	5.3948580	0.6283750	-2.8595970
H	4.4753310	0.9860000	-4.2884260	H	4.4230560	1.0186430	-4.2925350
H	2.4215690	2.2595640	-4.1614640	H	2.2551810	2.0517850	-4.0469160
H	-0.2819460	-4.5765620	-0.0496010	H	-0.4102250	-4.5356800	-0.1710870
H	1.8588940	-1.4900050	-2.9373560	H	1.8022050	-1.3504380	-2.9721590
H	-4.7530180	1.0819960	-1.4331340	H	-4.7775960	1.2001220	-1.3084080
H	-3.5728500	3.6988480	3.2199100	H	-3.3630600	3.4145660	3.2327680
H	-6.0610890	2.2782440	-1.2502190	H	-5.9552320	2.4804300	-0.9200140
H	-5.0559500	3.5793930	2.2476530	H	-4.8085310	3.5162790	2.2010590
H	-3.9211960	2.2032070	2.3212870	H	-3.8097400	2.0460420	2.1951480
H	-3.5367220	5.0561010	-1.7451190	H	-3.3960570	5.1377890	-1.6629440
H	-4.6512920	4.3755980	-2.9344230	H	-4.6322970	4.5333670	-2.7717430
H	-5.2555820	4.8405450	-1.3306150	H	-5.0749350	4.9449510	-1.1000090
H	-2.8363940	5.6597030	1.9996130	H	-2.4395330	5.4581130	2.1849410
H	-2.5893270	5.5707110	0.2500680	H	-2.2660380	5.5284110	0.4253090
H	-4.2395130	5.6599170	0.9117280	H	-3.8842070	5.5892430	1.1573330
H	-0.6168730	3.9785580	-2.6043760	H	-0.5715740	4.1353840	-2.7210790
H	-2.0838420	2.9964730	-2.8000770	H	-2.0827650	3.2221570	-2.9030700
H	-0.5810900	2.2451440	-2.2114890	H	-0.5815000	2.3720300	-2.4829350
H	-0.7771030	3.9859910	1.0542270	H	-0.4675850	3.7968830	0.9315760
H	0.1038510	4.6210370	-0.3513140	H	0.0821720	4.8651110	-0.3751710
H	-2.5147160	-0.9940880	-4.3057300	H	-2.6459020	-0.5523500	-4.2364960
H	-1.6466810	-0.1583500	-2.9952680	H	-1.9269690	0.2702710	-2.8351940
H	-3.4220220	-0.1985510	-3.0025840	H	-3.6849800	0.0488040	-2.9294920
H	-0.4018230	-2.2198230	-2.2667370	H	-0.4912710	-1.7472940	-2.3011280
H	-1.1194480	-2.9588140	-3.7224130	H	-1.1498510	-2.4368650	-3.8088740
H	-1.3305290	-3.7245650	-2.1344390	H	-1.2764150	-3.3381230	-2.2858320
H	-3.8442630	-4.2893790	-2.6185150	H	-3.7087310	-4.0253810	-2.8910420
H	-3.3932250	-5.6286140	-1.5549930	H	-3.2576750	-5.4383070	-1.9260830
H	-4.9792550	-4.8514230	-1.3666690	H	-4.8839820	-4.7401700	-1.7593980
H	-2.6804950	-5.1612440	0.7357050	H	-2.8083280	-5.1839990	0.4594530
H	-6.4694850	-0.4287770	-0.7077700	H	-6.4342890	-0.2754360	-0.3650770
H	-4.2586340	-4.4285360	1.1017250	H	-4.4014840	-4.4555270	0.7618780
H	-6.1960410	-1.9487810	0.1681100	H	-6.2057320	-1.8980250	0.3210280
H	-2.7630970	-3.4839010	1.3290420	H	-2.9165050	-3.5725290	1.1993720
H	-6.2059120	-2.8423400	-2.3234840	H	-6.3338930	-2.6147110	-2.1658880
H	-4.8602100	-2.2956270	-3.3503200	H	-5.0592250	-2.0137560	-3.2513780
H	-6.1822100	-1.1833720	-2.9574760	H	-6.3351890	-0.9177620	-2.6984690
H	-5.1114460	-0.5458790	0.4284640	H	-5.0500880	-0.5895030	0.6952320
H	0.2392870	2.8979710	0.0781310	H	0.5681890	3.1608930	-0.3616740
H	-5.2674810	2.0661440	-2.8247580	H	-5.4100640	2.2493750	-2.5961340
				H	-0.8380140	-0.0008940	-0.4889760
				H	0.8033160	1.0573770	-0.2560980
**II(Ph$_2$)$_2$**				**II(Ph$_2$)$_2$ (diprotonated)**			
C	0.7789160	4.2516820	0.5088540	C	4.6534120	3.9697450	-1.5199480
N	2.1642290	4.0719750	0.0748370	N	3.5394040	3.0487120	-1.2899870
C	2.9467550	5.3011590	0.1912160	C	2.8672220	2.8073580	-2.5733720
C	2.0066660	6.2636980	0.9268100	C	3.9766620	3.0208050	-3.6308630
C	0.6156420	5.7719170	0.5179950	C	5.2254660	3.4562560	-2.8393390
P	2.8180670	2.6190190	-0.4176800	P	2.8417080	2.7332790	0.1805570
N	3.2598310	2.9298180	-2.0213340	N	4.1405140	2.4165220	1.1499140
C	2.2473070	3.6284060	-2.8412060	C	4.1647020	2.6619550	2.6072430
C	2.4448100	3.0711950	-4.2517450	C	5.4962380	2.0544640	3.0522350
C	2.8653420	1.6274080	-3.9756250	C	5.6937570	0.9003480	2.0699980
C	3.8108000	1.7874930	-2.7855870	C	5.2277260	1.4982090	0.7460140
N	1.7627750	1.5244640	-0.1142930	N	1.6979010	1.6572870	-0.0875060
P	1.7013600	-0.1617670	-0.1282090	P	1.6397180	0.1308200	0.3938060
C	1.4993080	-0.4434160	1.7245790	N	2.9316960	-0.7503150	0.0817630
C	0.8004770	0.5045560	2.7279500	P	3.5245750	-1.9513690	-0.7394730
C	-0.7773590	0.3942160	2.7551990	N	4.8605420	-1.4318900	-1.5699090
C	-1.3350650	-0.6305930	1.7493650	C	4.7339130	-0.2896340	-2.4922210
C	-0.5979190	-1.8857330	2.2305210	C	6.1282060	-0.1836680	-3.1024800
C	0.9622130	-1.7848980	2.2293920	C	6.5524150	-1.6500360	-3.2111460
C	0.9507050	-1.6064760	3.7868400	C	6.0443320	-2.2540560	-1.9003120
C	1.3534070	-0.1375150	4.0315720	C	1.4317180	0.0527810	2.1986260
C	-0.6015230	-1.7184550	3.7915290	C	0.8982070	-1.2168560	2.8918750
C	-1.2107410	-0.3281980	4.0590420	C	-0.6847050	-1.2609140	2.9819340
C	0.8040840	0.4428240	5.3301980	C	-1.4200630	-0.0224310	2.4019070
C	-0.7312310	0.3169960	5.3526590	C	-0.7615330	1.0917060	3.2150260
P	-1.5454720	-0.1851130	-0.0538730	C	0.7922160	1.1383550	3.0792010
N	-2.0943920	-1.6757710	-0.6275730	C	-0.6224190	0.4416370	4.6319500
P	-3.2759060	-2.6419000	-0.2932530	C	-1.0875700	-1.0205970	4.4658060
N	-2.6442300	-4.1804300	0.0205040	C	0.9266290	0.5054730	4.5114910
C	-1.3245880	-4.5390490	-0.4946600	C	1.4762070	-0.9199050	4.3030640
C	-1.5998430	-5.3177600	-1.7950830	C	-0.4666560	-1.9851400	5.4674230
C	-3.0227920	-5.8995230	-1.5987180	C	1.0683040	-1.9036420	5.3903210
C	-3.4651740	-5.3695220	-0.2166840	P	-1.8306600	0.2626040	0.6542910
N	3.3420280	-0.6328940	-0.2476990	N	-3.1722950	1.1449860	0.7057480
P	3.9105010	-2.0322200	-0.6240110	P	-3.7550880	2.1293080	-0.4040820
N	5.4611420	-1.7857460	-1.1954440	N	-4.2134710	3.5138330	0.3994250
C	6.1473630	-0.4945870	-1.1029850	C	-5.1465520	4.4461590	-0.2479800
C	7.5405260	-0.8741850	-0.6086650	C	-6.5067600	4.1229000	0.3845220
C	7.7966260	-2.1904080	-1.3505050	C	-6.1523130	3.6513510	1.8149550
C	6.4229280	-2.8830550	-1.3431160	C	-4.6127450	3.4909060	1.8146950
N	3.2312080	-3.0082130	-1.8200070	N	-1.8214990	-1.1073450	-0.1824740

C	1.8758060	-3.5524900	-1.5828000
C	1.0139750	-3.1112450	-2.7890080
C	1.8665680	-2.0594740	-3.5061460
C	3.2908390	-2.5206050	-3.2059890
N	3.8823810	-3.1117140	0.6651740
C	4.0527010	-4.5669530	0.5802550
C	5.1040560	-4.9352360	1.6597660
C	5.5477950	-3.5913610	2.2643350
C	4.3645250	-2.6668260	1.9738100
N	4.3179990	2.4273130	0.2787680
C	5.5947670	2.9650310	-0.2060470
C	6.5853450	2.6262250	0.9171160
C	5.7070100	2.5679740	2.1697930
C	4.4288720	1.9142380	1.6475150
N	-2.9434710	0.7637000	0.1456100
P	-3.3556780	2.2187510	-0.2017610
N	-3.6529100	3.2836840	1.0714680
C	-4.6952780	2.8638640	2.0307220
C	-3.9251750	2.4876870	3.2940100
C	-2.8198470	3.5435850	3.3228340
C	-2.4766210	3.7554120	1.8371450
N	-2.2491210	3.1015800	-1.1110600
C	-2.3721220	4.5570470	-1.3237260
C	-1.5335680	4.8229910	-2.5749930
C	-1.7062480	3.5380180	-3.3839590
C	-1.6526480	2.4598400	-2.3031690
N	-4.8714880	2.1579680	-0.9172730
C	-5.2677170	1.0817460	-1.8185580
C	-6.7982510	1.2148300	-1.8539370
C	-7.0755710	2.7050660	-1.5315230
C	-5.6842240	3.3150460	-1.2737210
N	-4.3274890	-2.4920670	0.9972720
C	-4.0824950	-3.0878770	2.3200670
C	-5.2035960	-2.5091880	3.1834950
C	-5.3874860	-1.1084830	2.5991170
C	-5.2381040	-1.3335800	1.0941210
N	-4.3561790	-2.6200740	-1.5749080
C	-3.9928610	-2.1421460	-2.9090410
C	-5.3443750	-1.9895000	-3.6503060
C	-6.4201510	-2.3969800	-2.6267620
C	-5.6525200	-3.2818630	-1.6469520
H	-1.0019160	-2.8159100	1.8263930
H	2.5599530	-0.4093010	1.9998860
H	-2.3903570	-0.7752460	2.0187190
H	2.4454100	-0.0418300	4.0337550
H	1.1163500	1.5430060	2.5991850
H	1.4958070	-2.3318040	4.3978730
H	-1.0317520	-2.5231510	4.3948860
H	-2.3054990	-0.3935690	4.0759970
H	-1.2356930	1.3800550	2.6545020
H	1.4825340	-2.6581370	1.8267290
H	-1.0652110	-0.2848690	6.2058730
H	-1.2056110	1.2994460	5.4619060
H	1.2538800	-0.0817960	6.1811980
H	1.1127150	1.4912020	5.4136900
H	-0.4803750	4.9504020	-2.2993850
H	-1.8564300	5.7225970	-3.1033960
H	-2.6846900	3.5323380	-3.8775310
H	-0.9359720	3.3987260	-4.1468980
H	-2.1964430	1.5534510	-2.5844620
H	-0.6189350	2.1663710	-2.0905260
H	-1.9967310	5.1119030	-0.4602050
H	-3.4129280	4.8595690	-1.4862300
H	-7.2415760	0.5729580	-1.0869890
H	-4.9182870	0.1168930	-1.4491850
H	-4.8499790	1.2352800	-2.8282990
H	-3.1991440	4.4730400	3.7596040
H	-5.6719970	4.0585580	-0.4742420
H	-1.9444560	3.2362040	3.9013060
H	-7.7009830	2.7951950	-0.6399190
H	-1.5851900	3.1892730	1.5494100
H	-5.3108670	3.7933910	-2.1935450
H	-2.2759660	4.8106280	1.6260280
H	-3.4890260	1.4919590	3.1650070
H	-5.3734750	3.7053200	2.2248810
H	-4.5478780	2.4863430	4.1920320
H	-5.2874090	2.0434550	1.6204190
H	-3.0409630	-6.9916710	-1.6333640
H	-3.6966640	-5.5331470	-2.3787970
H	-3.2388380	-6.1080680	0.5630290
H	-3.1040790	-2.7958420	2.7223360
H	-4.1117960	-4.1790120	2.2702260
H	-6.3411970	-0.6441290	2.8616020
H	-6.1201980	-3.0954870	3.0545940
H	-4.9449350	-2.5087810	4.2451680
H	-4.5815330	-0.4537840	2.9490540
H	-6.2076320	-1.5689610	0.6322480

P	-2.7020690	-2.4165010	-0.3362750
N	-4.1823480	-2.3777990	0.3979170
C	-5.3752690	-3.1004030	-0.0919310
C	-6.3901250	-2.9063390	1.0353410
C	-5.5174330	-2.8632940	2.2906290
C	-4.2999860	-2.0589260	1.8334930
N	-1.8633090	-3.7061320	0.2651690
C	-0.3994040	-3.8343370	0.2836740
C	-0.1685310	-5.2978150	-0.0844350
C	-1.3454450	-5.9970510	0.6002350
C	-2.5132320	-5.0268010	0.3860820
N	-3.0742130	-2.7594120	-1.9175420
C	-2.1286010	-3.5366590	-2.7459500
C	-2.4531390	-3.1037190	-4.1771090
C	-2.8945590	-1.6496740	-4.0013440
C	-3.7441720	-1.7289110	-2.7353180
N	2.1080170	4.0119820	0.9497220
C	0.6577750	4.2498150	0.7839830
C	0.5184110	5.7672280	0.5377480
C	1.9364110	6.2299830	0.1842170
C	2.7920360	5.3109440	1.0507080
N	-4.9701970	1.3423050	-1.2136910
C	-5.8844220	0.4542140	-0.4653550
C	-7.1055100	0.3019300	-1.3829570
C	-6.5781450	0.6632540	-2.7747020
C	-5.5825500	1.7812950	-2.4771110
N	-2.7821540	2.6901570	-1.6144310
C	-2.0892370	3.9928140	-1.5575220
C	-0.9577070	3.8630950	-2.5865310
C	-0.7090270	2.3552100	-2.6885150
C	-2.1173090	1.7766660	-2.5618570
N	4.1171190	-3.2086260	0.1716240
C	5.1728180	-2.8466870	1.1486630
C	4.4658940	-2.9155150	2.4985780
C	3.5646700	-4.1394970	2.3295290
C	3.1269040	-4.0754550	0.8552090
N	2.3627650	-2.6358900	-1.7233100
C	2.7284530	-3.8034950	-2.5556750
C	1.6403700	-3.8428440	-3.6261900
C	1.3499370	-2.3603930	-3.8622030
C	1.4062100	-1.7671570	-2.4549890
H	1.2039770	2.1361100	2.9232170
H	-2.4505890	-0.0966140	2.7727840
H	2.5064240	0.0956790	2.4340860
H	-2.1780080	-1.0810090	4.5473060
H	-1.0692700	-2.2039420	2.5866700
H	-1.0706060	0.9635420	5.4786590
H	1.4524870	1.0852310	5.2718820
H	2.5692010	-0.8991770	4.2346440
H	1.2930390	-2.1419000	2.4629020
H	-1.2623840	2.0587920	3.1482890
H	1.4955660	-1.5781430	6.3444230
H	1.5052260	-2.8833020	5.1698900
H	-0.8249350	-1.7361500	6.4714410
H	-0.8188370	-2.9991670	5.2515540
H	0.7517050	-4.3544220	-3.2410090
H	1.9689770	-4.3676700	-4.5247450
H	2.1349050	-1.9224190	-4.4877290
H	0.3890920	-2.1712810	-4.3457500
H	1.7228510	-0.7199890	-2.4651460
H	0.4204360	-1.8011910	-1.9746160
H	2.7635460	-4.7118980	-1.9499890
H	3.7110930	-3.6606420	-3.0243180
H	6.7935070	0.3527520	-2.4167710
H	4.4260270	0.6131440	-1.9577960
H	3.9930210	-0.4938050	-3.2794670
H	4.1354590	-5.0537970	2.5155640
H	6.7966400	-2.1536390	-1.1089590
H	2.7061600	-4.1384600	3.0056770
H	7.6287940	-1.7832640	-3.3343270
H	2.1271180	-3.6470530	0.7598180
H	5.7929640	-3.3131270	-1.9867540
H	3.1117950	-5.0652680	0.3906750
H	3.8598770	-2.0122340	2.6320470
H	5.9856560	-3.5786820	1.0875910
H	5.1581310	-2.9992390	3.3386670
H	5.5866170	-1.8601390	0.9290460
H	2.1054180	7.2895980	0.3864670
H	2.1515160	6.0383960	-0.8735480
H	2.7853120	5.6475400	2.0950590
H	3.3303910	2.1604010	3.1093780
H	4.0974040	3.7288600	2.8290410
H	6.7221070	0.5360820	2.0235920
H	6.3015900	2.7869310	2.9364980
H	5.4683280	1.7390700	4.0969750
H	5.0417660	0.0603330	2.3376230
H	6.0368660	2.0554270	0.2576250

H	-4.8047010	-0.4572780	0.6050210
H	-0.8471370	-6.0893340	-1.9775070
H	-0.8294300	-5.1868790	0.2413760
H	-1.5808630	-4.6313970	-2.6470970
H	-5.5664760	-4.3036560	-2.0508280
H	-6.1104800	-3.3460390	-0.6581260
H	-6.7976420	-1.5194950	-2.0947890
H	-7.2692020	-2.9103540	-3.0845890
H	-5.3737580	-2.6495460	-4.5215190
H	-3.4416360	-1.2045160	-2.8241280
H	-3.3455640	-2.8612930	-3.4322000
H	-0.7245200	-3.6408120	-0.6278650
H	-5.4926320	-0.9679100	-4.0098920
H	-7.2070930	0.9085970	-2.8201640
H	-7.5874770	3.2209410	-2.3472960
H	-4.5301060	-5.1413710	-0.1493320
H	5.9437260	-5.4909890	1.2346820
H	4.6489500	-5.5685750	2.4262790
H	5.7774150	-3.6623040	3.3304550
H	6.4388740	-3.2121780	1.7527540
H	3.5762350	-2.7937100	2.7293490
H	4.6271910	-1.6074430	1.9478390
H	4.3553320	-4.8572840	-0.4277410
H	3.0980790	-5.0665770	0.7893530
H	0.0479220	-2.7132330	-2.4674110
H	1.4759020	-3.1680340	-0.6400290
H	1.9149210	-4.6451020	-1.5014500
H	8.1055270	-1.9787170	-2.3795260
H	4.0416880	-1.7337360	-3.3045360
H	8.5696190	-2.8092070	-0.8892450
H	1.7031790	-1.0812820	-3.0440850
H	6.3491600	-3.5816070	-0.5013070
H	3.5792660	-3.3465290	-3.8703180
H	6.2433950	-3.4543590	-2.2613800
H	7.5148710	-1.0440920	0.4744580
H	6.2137330	-0.0187940	-2.0934320
H	8.2920530	-0.1090630	-0.8200020
H	5.5890530	0.1773710	-0.4500970
H	7.0350530	1.6441850	0.7363900
H	7.3940020	3.3574820	0.9855800
H	5.8901190	2.5049780	-1.1544230
H	2.3815190	4.7136080	-2.7892720
H	1.2300110	3.4011330	-2.4941830
H	1.9900580	1.0400950	-3.6760810
H	1.5398690	3.1569180	-4.8584730
H	3.2507460	3.6074150	-4.7649420
H	3.3356280	1.1325600	-4.8290160
H	3.8666640	0.8897140	-2.1612040
H	4.8259050	2.0259920	-3.1322990
H	6.1625920	2.0067160	2.9892130
H	4.5059330	0.8189130	1.6308610
H	5.4906920	3.5812480	2.5267190
H	3.8737840	5.1319640	0.7503970
H	3.2229270	5.6934120	-0.7990340
H	2.1940790	7.3081080	0.6680020
H	2.1354080	6.1535390	2.0088530
H	-0.1754490	6.1040500	1.1951030
H	0.0763660	3.7486860	-0.1611270
H	0.6201940	3.8391640	1.5163120
H	3.5454440	2.1750790	2.2391500
H	0.3735220	6.1282390	-0.4899960
H	0.8236510	-3.9651360	-3.4471170
H	1.6546360	-1.9882920	-4.5762530
H	5.5397160	4.0484260	-0.3694840

H	4.8633210	0.7291140	0.0667010
H	0.1801680	6.2675680	1.4495920
H	0.1149250	3.9335060	1.6807850
H	-0.2071980	5.9892620	-0.2484110
H	4.2956130	5.0039520	-1.6318350
H	5.3638980	3.9390790	-0.6918090
H	5.8738970	2.5988180	-2.6382710
H	5.8148610	4.2131130	-3.3605840
H	3.6748630	3.7964140	-4.3384410
H	2.4455150	1.8014250	-2.5858670
H	2.0436120	3.5155380	-2.7283420
H	0.2810360	3.6550360	-0.0488390
H	4.1614630	2.1097580	-4.2050300
H	6.1226480	0.3412020	-4.0600100
H	6.0481830	-2.1205470	-4.0620330
H	3.8339470	5.2372630	0.7335910
H	-6.9889460	3.3152290	-0.1760390
H	-7.1801040	4.9823420	0.3732400
H	-6.4644720	4.3756830	2.5706280
H	-6.6451890	2.7040680	2.0487360
H	-4.1392420	4.3441500	2.3129170
H	-4.2616080	2.5779690	2.2938170
H	-5.1245100	4.3480660	-1.3349420
H	-4.8345530	5.4679690	-0.0023900
H	-0.0712520	4.4263550	-2.2816040
H	-1.7108500	4.1786540	-0.5496580
H	-2.7712650	4.8098300	-1.8085110
H	-6.0613210	-0.1883300	-3.2259220
H	-2.1185240	0.7479340	-2.1905310
H	-7.3649610	0.9859340	-3.4592040
H	-0.0782720	2.0147120	-1.8594020
H	-6.1168660	2.7343950	-2.3680570
H	-2.6335360	1.7834220	-3.5305500
H	-4.8251200	1.9020100	-3.2532910
H	-7.8874610	1.0094140	-1.0888320
H	-5.3781560	-0.4937460	-0.2763200
H	-7.5309840	-0.7030580	-1.3331080
H	-6.1636890	0.8827280	0.5041510
H	-6.9161300	-1.9539200	0.9105850
H	-7.1343140	-3.7045190	1.0526780
H	-5.7236640	-2.6828200	-1.0397760
H	-2.2617400	-4.6110510	-2.5920830
H	-1.0889750	-3.2858610	-2.5038400
H	-2.0214330	-1.0095920	-3.8275070
H	-1.5987040	-3.2293030	-4.8453420
H	-3.2810200	-3.7011210	-4.5718270
H	-3.4493510	-1.2539700	-4.8542970
H	-3.8154010	-0.7768570	-2.1994570
H	-4.7634170	-2.0556560	-2.9777750
H	-6.0140470	-2.4057330	3.1481150
H	-4.4609090	-0.9815680	1.9622460
H	-5.2125090	-3.8764870	2.5718660
H	-3.2222510	-5.0366810	1.2194150
H	-3.0643970	-5.2749370	-0.5296930
H	-1.5590290	-6.9868560	0.1934470
H	-1.1400530	-6.1059250	1.6697330
H	0.8009270	-5.6690860	0.2512350
H	0.0822230	-3.1324570	-0.3986710
H	-0.0197960	-3.6307700	1.2934830
H	-3.3933760	-2.3459150	2.3768420
H	-0.2258230	-5.4234920	-1.1712000
H	-1.2840930	4.2565950	-3.5537320
H	-0.2261090	2.0648260	-3.6243600
H	-5.1632100	-4.1637490	-0.2486850
H	-0.8245780	1.0543380	0.0813950
H	0.5302410	-0.4446920	-0.2308220

III($(Pa_2)_2$)

C	1.7131370	0.7160110	0.1328130
C	1.7226300	1.6425230	-1.0796950
C	2.4432020	2.8707070	-0.5022730
C	2.1964560	2.8403120	1.0420250
C	1.2973560	1.6084860	1.3312500
C	0.4114090	2.1499880	-1.7513150
C	0.5043980	3.7244830	-1.7850450
C	1.8099270	4.1623550	-1.0391820
C	-1.0687200	1.7576070	-1.3808050
C	-1.7965330	3.0996340	-1.0989190
C	-0.9058800	4.2987720	-1.4647790
C	-1.5983090	0.9351420	-0.1767220
C	-1.4846740	1.8616930	1.0300380
C	-2.0362470	3.1711350	0.4452880
C	-1.2374990	4.3704840	0.9755570
C	-0.0012420	3.7677540	1.7246260
C	-0.1175750	2.1944990	1.6992530
C	1.4725790	4.1486980	1.4013510
C	-1.1242670	5.2506730	-0.2807300

III($(Pa_2)_2$) (diprotonated)

C	-0.1460300	-2.0225170	0.2291980
C	0.5247370	-2.5279080	-1.0495160
C	1.3409580	-3.6962810	-0.4810650
C	1.6082760	-3.3632890	1.0224340
C	0.9793320	-1.9709590	1.3022480
C	1.4959770	-1.6310990	-1.8788980
C	2.8294240	-2.4638380	-2.0276810
C	2.7035620	-3.7746050	-1.1800860
C	1.8959700	-0.1164750	-1.6590980
C	3.4470240	-0.1094800	-1.5603070
C	4.0305920	-1.4839780	-1.9173930
C	1.6128160	0.8433950	-0.4656690
C	2.4860750	0.3508770	0.6872340
C	3.8144240	0.1594840	-0.0617170
C	4.5445390	-1.0830620	0.4590510
C	3.5221980	-1.8267830	1.3818980
C	2.1947320	-0.9748710	1.4570610
C	3.1330230	-3.3207360	1.2036150
C	5.1091050	-1.6762410	-0.8431060

C	1.8150840	5.0566250	0.2122710	C	3.6309440	-4.1255670	-0.0039820
P	-1.5451550	-0.9099120	0.1878240	P	0.1920560	1.9122060	-0.0597310
N	-2.0835270	-1.4782040	-1.3310960	N	-0.4685040	2.3382260	-1.4651750
P	-1.3453020	-2.3940520	-2.3813600	P	-1.9797500	2.7112880	-1.8417350
C	-0.4889370	-3.8818510	-1.7599590	C	-3.1269420	2.7211180	-0.4383070
P	1.4127030	-1.1079100	-0.2200290	P	-1.6078090	-0.9903640	-0.0756110
N	1.8673230	-1.7435790	1.2982460	N	-2.3076770	-0.3470290	1.2143810
P	1.0181420	-2.3911800	2.4559820	P	-2.2488080	-0.2834390	2.8046710
C	-0.0011480	-1.2822110	3.4888220	C	-0.8382120	0.6460110	3.4609210
N	-2.9544220	-0.9643490	1.1613120	N	0.7253270	3.0741120	0.9202730
P	-4.4409140	-1.2137420	0.6850760	P	1.6134660	4.3814190	0.6794390
C	-5.5695850	-0.9832100	2.0899800	C	1.6708320	5.3064900	2.2268650
C	-5.1292890	-0.1485780	-0.6326350	C	3.3386530	4.0584110	0.2156960
C	-4.8003560	-2.9007340	0.0880610	C	0.9560730	5.4922930	-0.5912530
C	-0.1312060	-1.5999820	-3.4913500	C	-2.6380820	1.5374430	-3.0524490
C	-2.5954130	-3.0485130	-3.5310100	C	-2.0726910	4.3401850	-2.6156000
N	2.8087670	-1.3636150	-1.1797550	N	-2.5701240	-1.8754300	-1.0125440
P	4.2409690	-1.8154630	-0.6858230	P	-4.1391580	-2.1627980	-0.9107800
C	5.0604010	-0.8463010	0.6309810	C	-4.6141800	-3.2053660	0.4926370
C	4.3544130	-3.5324720	-0.0776580	C	-5.1705360	-0.6772840	-0.7886150
C	5.4036780	-1.7546730	-2.0807490	C	-4.6384020	-3.0375810	-2.4065480
C	2.1814390	-3.1293410	3.6456990	C	-2.2245530	-1.9028210	3.6161600
C	-0.1062100	-3.7688550	2.0371220	C	-3.7314750	0.5663280	3.3823710
H	-2.1493020	1.4832620	1.8110200	H	2.5758530	1.1448780	1.4327430
H	-1.4856520	1.2877250	-2.2768020	H	1.5732430	0.4108700	-2.5593040
H	-1.8219640	4.9202640	1.7203650	H	5.3877320	-0.7920780	1.0916200
H	-1.2741910	4.7870550	-2.3728820	H	4.5170110	-1.4487880	-2.8962340
H	-2.6753230	0.9679470	-0.3978390	H	2.1894080	1.7133590	-0.8278330
H	-0.4442610	6.0961570	-0.2237650	H	5.5380030	-2.6713370	-0.7803430
H	-2.1217290	5.6888350	-0.4225560	H	5.9414950	-1.0186590	-1.1260220
H	1.2545240	5.9852080	0.1504780	H	4.6987700	-4.0780930	-0.1922740
H	2.8621850	5.3585020	0.3521520	H	3.4238190	-5.1788380	0.2263860
H	2.7853220	0.6059820	0.3531800	H	-0.7188850	-2.9024020	0.5718960
H	2.4616240	4.6258550	-1.7868810	H	2.7214980	-4.6102250	-1.8852230
H	2.3299150	1.1756270	-1.8595060	H	-0.2452000	-2.8961600	-1.7302620
H	3.5170850	2.8298490	-0.7117320	H	0.7850590	-4.6338310	-0.5651240
H	3.1510740	2.7522070	1.5704700	H	1.1464750	-4.1202290	1.6620060
H	1.9028450	4.5885850	2.3069380	H	3.4232320	-3.8471470	2.1171090
H	1.6502100	1.0942980	2.2303810	H	0.4941960	-1.9895920	2.2811980
H	-3.1060110	3.2741070	0.6545980	H	4.4355780	1.0564410	0.0162510
H	-2.7546360	3.1360180	-1.6271260	H	3.8611900	0.6808550	-2.1928290
H	0.4970950	1.8165670	-2.7897970	H	1.0391840	-1.6039130	-2.8702660
H	0.6890960	4.0013410	-2.8270040	H	2.8491450	-2.8181870	-3.0610350
H	-0.1472190	4.0723310	2.7650030	H	3.9723580	-1.7698680	2.3757490
H	-0.2463300	1.8798780	2.7390960	H	2.1046590	-0.6549550	2.4980980
H	-4.5052390	-0.2718820	-1.5204400	H	3.3864990	3.6247820	-0.7860140
H	-6.1629110	-0.4250360	-0.8600080	H	3.9057040	4.9926050	0.2210060
H	-5.0958690	0.8963170	-0.3132610	H	3.7813740	3.3634570	0.9329680
H	-4.5160310	-3.6218370	0.8584040	H	-0.0420290	5.8217370	-0.2943430
H	-4.1928340	-3.0711310	-0.8024950	H	0.8905140	4.9503870	-1.5373940
H	-5.8601010	-3.0230500	-0.1514290	H	1.6053070	6.3624660	-0.7096860
H	-5.5020820	0.0480970	2.4436900	H	2.1180650	4.6803270	3.0013910
H	-5.2807430	-1.6555250	2.9008470	H	0.6542790	5.5731780	2.5220280
H	-6.6002480	-1.1965700	1.7950250	H	2.2662080	6.2135640	2.1029410
H	0.1191700	-2.2687090	-4.3200550	H	-3.6548480	1.8177150	-3.3379260
H	-0.5711900	-0.6820460	-3.8903370	H	-1.9986170	1.5455580	-3.9377240
H	0.7737350	-1.3565120	-2.9306740	H	-2.6409210	0.5288480	-2.6304540
H	-2.1291930	-3.6628160	-4.3054860	H	-3.0868470	4.5300450	-2.9738180
H	-3.3173020	-3.6549190	-2.9792290	H	-1.7963530	5.1053790	-1.8881090
H	-3.1217880	-2.2149150	-4.0016530	H	-1.3766760	4.3754410	-3.4562670
H	-1.1915570	-4.4823620	-1.1764620	H	-2.8216770	3.4927690	0.2727910
H	0.3353290	-3.5627130	-1.1181180	H	-3.1126740	1.7492810	0.0655840
H	-0.1014720	-4.4796380	-2.5895860	H	-4.1397250	2.9323790	-0.7887550
H	3.7091660	-3.6148900	0.7988040	H	-4.8907870	-0.1286000	0.1137130
H	3.9921540	-4.2110430	-0.8539660	H	-5.0054240	-0.0446910	-1.6634520
H	5.3824570	-3.7963070	0.1849920	H	-6.2260550	-0.9539310	-0.7370170
H	5.0321560	-2.3873390	-2.8898330	H	-4.4100050	-2.4177120	-3.2757170
H	6.3921190	-2.1061980	-1.7737560	H	-5.7096270	-3.2472880	-2.3777880
H	5.4827950	-0.7269110	-2.4421920	H	-4.0840960	-3.9754610	-2.4773350
H	4.4179930	-0.8719990	1.5137510	H	-4.4623150	-2.6483940	1.4188550
H	5.1775430	0.1898170	0.3028370	H	-3.9918600	-4.1023510	0.5034340
H	6.0427380	-1.2638170	0.8700190	H	-5.6663320	-3.4892720	0.4156780
H	1.6472470	-3.5794580	4.4862950	H	-2.2190010	-1.7762500	4.7011150
H	2.7736450	-3.8979890	3.1437020	H	-3.1120150	-2.4675460	3.3248410
H	2.8542240	-2.3543530	4.0200340	H	-1.3364240	-2.4633830	3.3180160
H	-0.5160530	-4.2199660	2.9452370	H	-3.7155710	0.6550700	4.4707650
H	-0.9182290	-3.3798880	1.4193860	H	-3.7678740	1.5617050	2.9348820
H	0.4477870	-4.5252180	1.4752250	H	-4.6136160	0.0011750	3.0755100
H	0.6154400	-0.4420890	3.8187030	H	0.0971890	0.1562510	3.1817920
H	-0.3773270	-1.8194960	4.3642610	H	-0.9041160	0.6940770	4.5504770
H	-0.8416550	-0.9082320	2.9001000	H	-0.8417050	1.6602060	3.0539880
				H	-1.1822660	0.0535270	-0.9043980
				H	-0.7216040	1.2592670	0.7691750

**III(Pb_2)$_2$**				**III(Pb_2)$_2$ (diprotonated)**			
C	-1.6347060	-5.5214640	-0.6413510	C	-1.5499970	1.5774580	0.0975620
C	-1.4753200	-4.4510520	-1.7306750	C	-1.3616300	2.4207110	-1.1692840

C	-2.1669760	-3.2320110	-1.1005510	C	-1.8463540	3.7815320	-0.6318710
C	-2.1884070	-3.4707660	0.4461540	C	-1.6025670	3.7669850	0.9149980
C	-1.4710400	-4.8025100	0.7076210	C	-0.9515160	2.4036230	1.2620620
C	-1.3484770	-1.9376280	-1.3458860	C	-0.6441060	4.9241680	1.2276820
C	0.0158460	-2.4091360	-1.9683920	C	-0.8302720	5.8392790	0.0088810
C	-0.0714100	-3.9674350	-2.2010760	C	-0.9976080	4.9196000	-1.2112800
C	1.2721790	-4.6149030	-1.7297440	C	0.0206840	2.6714030	-1.8577930
C	2.1121810	-3.4714050	-1.1407030	C	0.2076330	4.2376810	-1.9347220
C	1.4517550	-2.1126010	-1.4370400	C	0.5588630	2.7115510	1.5807270
C	2.0955220	-3.6417060	0.4141440	C	0.7329450	4.2811120	1.5653210
C	1.3988240	-2.3862040	0.9947190	C	1.6970930	4.5696650	-1.6255960
C	1.7186300	-1.3742510	-0.1270740	C	2.3697870	3.2453810	-1.2381500
C	1.3487510	-5.6709950	-0.6150960	C	1.4217130	2.0493910	-1.5085410
C	1.2791240	-4.9092290	0.7162390	C	2.6217700	3.2950530	0.3053890
C	-0.0782030	-4.4304780	1.3128070	C	1.8434220	2.1158590	0.9172360
C	-0.0134850	-2.8636400	1.4913850	C	1.8149910	1.1762590	-0.2940730
C	-1.4105210	-2.3002490	1.0755950	C	2.0570670	4.6282920	0.8116270
C	-1.6187700	-1.2254740	0.0036570	C	2.0945470	5.4887010	-0.4615260
P	1.4737460	0.4790500	-0.0688440	P	1.5842990	-0.6049370	0.0040680
N	2.6073340	0.9003640	-1.2723570	N	1.5358450	-1.4304530	-1.3524650
P	4.1068230	1.3453150	-1.0404160	P	0.9101390	-1.8335180	-2.7513750
C	4.8513900	1.6930620	-2.6745940	C	-0.3295430	-3.1611210	-2.6204850
C	4.0767900	2.7224090	-3.4979630	C	0.1476480	-4.3923660	-1.8531820
P	-1.5144090	0.6034010	0.5420240	P	-1.8021670	-0.2209300	-0.0257630
N	-2.9822530	0.6048860	1.4407440	N	-1.9673970	-0.8600940	1.4167410
P	-4.4256040	0.9359670	0.8806730	P	-1.5252430	-1.3383320	2.8591380
C	-5.6528030	0.6571970	2.2099380	C	-1.1680860	-3.1213570	2.8717790
C	-5.3990500	1.4628200	3.4839800	C	-0.0168410	-3.4839100	1.9330950
N	-1.9935960	1.3522190	-0.9137800	N	2.7106090	-1.0573160	1.0530060
P	-1.2620950	2.0646950	-2.1096050	P	4.2093540	-1.5840880	0.8724660
C	-2.5382410	2.8983190	-3.1251880	C	5.3470120	-0.3126530	0.2256750
C	-3.5762980	1.9422560	-3.7099400	C	6.8272730	-0.7017480	0.2075470
C	-0.0491930	3.3971900	-1.7575760	C	4.3435200	-3.0064060	-0.2601630
C	-0.6034870	4.5569160	-0.9332690	C	3.4576520	-4.1907180	0.1264210
C	-0.3559210	0.9674450	-3.2719100	C	4.8276500	-2.0584770	2.5195490
C	0.0788070	1.5790350	-4.6048550	C	3.7627570	-2.6765000	3.4281410
N	2.3243010	0.7572070	1.3805870	N	-3.0475250	-0.4385040	-1.0197780
P	1.9455130	1.1530340	2.8518000	P	-4.5014430	-1.0225020	-0.6884050
C	3.4094930	0.8704760	3.9119850	C	-5.4478740	0.0298620	0.4604610
C	3.8773230	-0.5841890	3.9071020	C	-6.8530270	-0.4645140	0.8106680
C	1.5600350	2.9234210	3.1434470	C	-4.5020790	-2.6937310	0.0633430
C	0.2697460	3.3745570	2.4614370	C	-3.3567710	-3.6031520	-0.3822930
C	0.5763920	0.2129070	3.6243110	C	-5.4216980	-1.0833600	-2.2524460
C	0.1965080	0.6148160	5.0491380	C	-4.7493840	-1.9321040	-3.3337450
C	-5.0507630	-0.0963650	-0.5110280	C	0.0668230	-0.4756170	-3.6341980
C	-6.3816090	0.3243490	-1.1378450	C	-0.5882020	-0.8653980	-4.9615600
C	-4.6858210	2.6620420	0.2890570	C	2.2558630	-2.4389110	-3.8116150
C	-3.8493410	3.6942520	1.0415680	C	3.3408480	-1.3859740	-4.0454600
C	5.2304690	0.1119610	-0.2687420	C	-0.0511920	-0.4950690	3.5305430
C	6.6829750	0.5423880	-0.0540660	C	0.3229890	-0.9009180	4.9582680
C	4.3776580	2.8607600	-0.0227810	C	-2.8946910	-1.0517820	4.0174740
C	3.2294090	3.8655430	-0.0824210	C	-3.2426570	0.4310050	4.1613130
H	1.9355860	-2.0423470	1.8841130	H	-1.3896460	2.0160800	2.1858520
H	1.9916290	-1.5904100	-2.2321990	H	-2.0464740	2.0495070	-1.9364780
H	1.7768080	-5.5116400	1.4831580	H	-0.9775950	5.4639930	2.1183490
H	1.7575160	-5.0270120	-2.6204300	H	-1.5610960	5.4645980	-1.9737550
H	2.8193770	-1.3621330	-0.0793420	H	-2.6267370	1.7318130	0.2986220
H	0.6826980	-6.5241150	-0.7095660	H	-0.1143640	6.6489660	-0.0905380
H	2.3639590	-6.0872300	-0.6720000	H	-1.8038130	6.3280660	0.1454380
H	-1.0552700	-6.4300460	-0.7798090	H	1.5823100	6.4444480	-0.4146520
H	-2.6859030	-5.8385440	-0.6810040	H	3.1527950	5.7344150	-0.6200580
H	-2.7134300	-1.2017960	-0.0456940	H	2.8923950	1.0506090	-0.5028400
H	-2.0198470	-4.7831170	-2.6206380	H	2.1387070	4.9661120	-2.5441550
H	-1.8422300	-1.3337710	-2.1129810	H	1.7587960	1.5191420	-2.4019720
H	-3.1899700	-3.1138700	-1.4726840	H	3.3166060	3.1037550	-1.7664590
H	-3.2218870	-3.4903480	0.8075090	H	3.6910900	3.2002360	0.5139500
H	-2.0046900	-5.3826810	1.4674680	H	2.7343710	5.0750400	1.5448480
H	-1.9091830	-1.9630430	1.9900370	H	2.4276300	1.6413070	1.7097070
H	3.1195670	-3.7242930	0.7921840	H	-2.5535680	3.8761490	1.4435260
H	3.1424720	-3.4959020	-1.5101470	H	-2.9104220	3.9199190	-0.8418570
H	0.0048690	-1.9533050	-2.9623830	H	-0.1432110	2.3374970	-2.8847620
H	-0.0751290	-4.1185910	-3.2844410	H	0.0764140	4.5052150	-2.9858470
H	-0.1023880	-4.8554250	2.3204420	H	0.9286030	4.5724780	2.5999460
H	0.0288840	-2.6859940	2.5699570	H	0.6522110	2.4106970	2.6267690
H	-0.2691370	0.3251650	2.9357770	H	0.7856150	-0.6941140	2.8529240
H	0.8731510	-0.8403300	3.5812330	H	-0.2533180	0.5783370	3.4722380
H	3.1825680	1.2095460	4.9284390	H	-2.6313710	-1.4956370	4.9824090
H	4.1952240	1.5275250	3.5228880	H	-3.7457150	-1.6174820	3.6248510
H	1.5275000	3.1111050	4.2225020	H	-0.9564990	-3.4258580	3.9013530
H	2.4194880	3.4763310	2.7474740	H	-2.0925470	-3.6238040	2.5691780
H	5.3214080	3.3182200	-0.3409220	H	-5.4735600	-3.1478310	-0.1585480
H	4.5065690	2.5163590	1.0077550	H	-4.4579480	-2.5437070	1.1465800
H	5.8872770	2.0152750	-2.5255230	H	-6.4301060	-1.4495100	-2.0364490
H	4.8847670	0.7319930	-3.1994690	H	-5.5163190	-0.0428670	-2.5799490
H	5.1764640	-0.7860090	-0.8948590	H	-5.4794170	1.0273300	0.0096580
H	4.7465470	-0.1318380	0.6829390	H	-4.8270440	0.1049160	1.3607220
H	0.5140770	0.6030640	-2.7158240	H	-0.6826510	-0.0690410	-2.9467060

H	-1.0129690	0.1097480	-3.4449230	H	0.8136460	0.3068900	-3.7932420
H	-2.0351980	3.4644140	-3.9169110	H	1.8246480	-2.7868960	-4.7553470
H	-3.0221020	3.6303950	-2.4697540	H	2.6685930	-3.3167320	-3.3036160
H	0.3369670	3.7629590	-2.7159350	H	-0.6253950	-3.4323540	-3.6389150
H	0.7820810	2.9012310	-1.2483780	H	-1.2099170	-2.7120200	-2.1455680
H	-5.7552020	2.8954720	0.3418630	H	5.3963150	-3.3001080	-0.3152500
H	-4.3937990	2.6556560	-0.7648130	H	4.0551710	-2.6211380	-1.2434420
H	-6.6501260	0.8704540	1.8102280	H	5.6719920	-2.7399170	2.3732140
H	-5.6160320	-0.4163910	2.4251770	H	5.2278830	-1.1469780	2.9760610
H	-5.1065930	-1.1237910	-0.1324340	H	5.1836360	0.5758880	0.8455900
H	-4.2460900	-0.0742630	-1.2529790	H	5.0003280	-0.0651730	-0.7843920
H	4.8105290	-0.6924720	4.4660340	H	0.4347690	-0.7784600	0.7719540
H	3.1344990	-1.2436960	4.3666570	H	-4.1371250	0.5475400	4.7763440
H	4.0436990	-0.9274540	2.8823010	H	-2.4332200	0.9892120	4.6405280
H	-0.6070680	-0.0279360	5.4190480	H	-3.4401770	0.8856170	3.1859140
H	1.0393030	0.5203160	5.7409820	H	1.2099050	-0.3451630	5.2706290
H	-0.1620980	1.6473500	5.0942120	H	-0.4753510	-0.6791460	5.6710770
H	0.1592840	4.4612380	2.5178460	H	0.5600200	-1.9660380	5.0276570
H	0.2624780	3.0765050	1.4098430	H	0.1260510	-4.5662710	1.9073840
H	-0.6092510	2.9158220	2.9234940	H	-0.2339380	-3.1511090	0.9137150
H	-6.0753180	1.1422920	4.2812120	H	0.9267580	-3.0238190	2.2417990
H	-4.3705040	1.3263470	3.8272840	H	4.1982040	-2.8991530	4.4048500
H	-5.5649730	2.5310210	3.3187250	H	2.9304630	-1.9832420	3.5687020
H	-3.9229370	4.6730050	0.5581310	H	3.3622730	-3.6059470	3.0167410
H	-4.1699280	3.8068550	2.0806520	H	3.4415540	-4.9241480	-0.6830990
H	-2.8010130	3.3872110	1.0438410	H	3.8257020	-4.6915630	1.0253100
H	-6.6592990	-0.3703210	-1.9356610	H	2.4309080	-3.8635190	0.3068000
H	-7.1963850	0.3292760	-0.4078270	H	7.4207520	0.1315500	-0.1747940
H	-6.3195990	1.3234540	-1.5785880	H	7.1945140	-0.9383840	1.2095650
H	-4.3339210	2.4911940	-4.2758110	H	7.0118820	-1.5636750	-0.4384080
H	-3.1156580	1.2147470	-4.3859810	H	4.1820170	-1.8288590	-4.5824030
H	-4.0784200	1.3875230	-2.9134590	H	2.9666260	-0.5489740	-4.6412570
H	0.6308090	0.8396400	-5.1922940	H	3.7120180	-0.9878940	-3.0964390
H	-0.7761090	1.9047930	-5.2052200	H	-1.0037100	0.0272180	-5.4340140
H	0.7377620	2.4410010	-4.4656850	H	0.1306080	-1.3047370	-5.6582540
H	0.2027790	5.2372180	-0.6428270	H	-1.4063510	-1.5748210	-4.8178220
H	-1.3425440	5.1359220	-1.4955970	H	-0.6777500	-5.0975220	-1.7313830
H	-1.0838000	4.1989570	-0.0203230	H	0.9540570	-4.9067010	-2.3824200
H	3.4511590	4.7349450	0.5439410	H	0.5138630	-4.1226100	-0.8613020
H	3.0401730	4.2208510	-1.0989560	H	-3.4479270	-4.5764040	0.1058520
H	2.3109590	3.4031560	0.2858730	H	-3.3531540	-3.7677830	-1.4622210
H	4.4829180	2.7883340	-4.5109510	H	-2.3918620	-3.1763000	-0.0969340
H	3.0222730	2.4425260	-3.5636740	H	-5.2743830	-1.8053630	-4.2829850
H	4.1382480	3.7182880	-3.0499040	H	-3.7079240	-1.6317030	-3.4748500
H	7.2483050	-0.2620970	0.4246720	H	-4.7732290	-2.9949840	-3.0807220
H	7.1830120	0.7785130	-0.9977810	H	-7.3197760	0.2265710	1.5160140
H	6.7504810	1.4217660	0.5927550	H	-7.4947950	-0.5201330	-0.0722850
				H	-6.8290730	-1.4514300	1.2801050
				H	-0.7557530	-0.7976240	-0.7474500
**III(Pc$_2$)$_2$**				**III(Pc$_2$)$_2$ (diprotonated)**			
C	-1.3397490	1.9939810	-0.1978990	C	-1.2053660	1.7471100	-0.3177090
C	-1.1355370	2.5140530	-1.6169630	C	-0.9047790	2.0967690	-1.7889570
C	-1.3749790	4.0195050	-1.4211810	C	-1.3869510	3.5678030	-1.7722840
C	-1.0520070	4.3258370	0.0767540	C	-1.2220620	4.0819560	-0.3021020
C	-0.5978830	2.9912380	0.7203220	C	-0.6457100	2.9103980	0.5250150
C	0.0916010	5.3544240	0.0853390	C	-0.2357400	5.2547650	-0.3406360
C	-0.0092580	5.9723550	-1.3164440	C	-0.3487450	5.7162300	-1.8023160
C	-0.3866210	4.8299990	-2.2737410	C	-0.4944670	4.4473060	-2.6556110
C	0.2205200	2.3541030	-2.3709100	C	0.5004190	2.1105810	-2.4865600
C	0.6528260	3.8005290	-2.8307580	C	0.7284360	3.5680340	-3.0523770
C	0.9507370	3.1267770	0.9497290	C	0.8636850	3.2478200	0.7838390
C	1.3691380	4.5933360	0.5475090	C	1.0968500	4.7259830	0.2751700
C	2.1918880	3.9338240	-2.6311140	C	2.2087580	3.9823070	-2.7817190
C	2.6548470	2.6451450	-1.9336350	C	2.8348980	2.8369440	-1.9760320
C	1.5112220	1.5996870	-1.8760050	C	1.8775780	1.6268140	-1.9292130
C	2.9969760	3.0265970	-0.4561090	C	3.0006120	3.3466480	-0.5043160
C	2.0764710	2.1724410	0.4331880	C	2.1389080	2.4250210	0.3876310
C	1.8290240	0.9733020	-0.4916190	C	2.1609440	1.1641360	-0.4939770
C	2.6864830	4.5199350	-0.2901300	C	2.4681650	4.7848310	-0.4645050
C	2.7937930	5.0277040	-1.7374180	C	2.5851980	5.2092940	-1.9360260
P	1.3822650	-0.7087470	0.3023270	P	1.7130430	-0.4927810	0.1089440
N	1.3968870	-1.6760600	-1.0970330	N	0.9471290	-1.1999700	-1.0872010
P	0.2769990	-2.6251600	-1.6750420	P	0.3666710	-2.5321840	-1.7334460
C	-0.8385800	-1.9319920	-2.9505420	C	-0.6047930	-2.0365890	-3.1823800
C	-0.1300870	-1.1517330	-4.0568120	C	0.2195100	-1.3144890	-4.2529830
C	-1.1293070	-0.4228700	-4.9534250	C	-0.6791860	-0.5970590	-5.2570320
P	-1.4814870	0.1455030	-0.0049220	P	-1.7402270	0.1257290	0.3403620
N	-2.1166470	0.1078700	1.5813530	N	-2.3937300	0.4406340	1.7862160
P	-1.4152390	-0.2503690	2.9490790	P	-1.7740520	0.1339620	3.2335170
C	0.0665620	0.7480470	3.3619100	C	-0.3491670	1.2041310	3.6286460
C	0.9853890	0.2585950	4.4813130	C	0.4416170	0.8737710	4.8976450
C	2.1701160	1.2049050	4.6708500	C	1.5884510	1.8641020	5.0962820
N	2.8995380	-1.0284880	1.0249430	N	2.9515060	-1.2486830	0.7914500
P	4.3821340	-1.2574260	0.5256840	P	4.5456580	-1.2697110	0.7216100
C	4.6757090	-2.0346070	-1.1139240	C	5.2341520	-1.6942880	-0.9146870
C	4.3894560	-1.1552330	-2.3332970	C	4.5916240	-0.9469210	-2.0873300

C	4.6255600	-1.9107740	-3.6393860	C	5.1322880	-1.4222530	-3.4332240
C	5.1954310	-2.3938230	1.7110000	C	5.1095070	-2.5515130	1.8753760
C	6.7171990	-2.5414880	1.6340060	C	6.6281640	-2.6871890	2.0258770
C	7.2368850	-3.5766880	2.6307140	C	6.9920760	-3.8218430	2.9814170
C	5.4639240	0.2294410	0.4854300	C	5.2950830	0.3123680	1.2361300
C	5.5342910	0.9814930	1.8151100	C	4.8132970	0.7695790	2.6181690
C	6.4089480	2.2303800	1.7303130	C	5.2156590	2.2110800	2.9178680
N	-2.8414550	-0.0942210	-1.0002370	N	-2.7293190	-0.5050280	-0.7574740
P	-4.3560560	-0.2185050	-0.5697490	P	-4.3294980	-0.6160770	-0.7516880
C	-5.0879610	1.2560000	0.2361590	C	-5.1594290	1.0002100	-0.6538780
C	-5.0668980	2.4987880	-0.6628440	C	-4.8631290	1.9042860	-1.8569590
C	-5.1213700	3.7942070	0.1431240	C	-5.2308260	3.3597160	-1.5810780
C	-4.6981560	-1.6095210	0.5706000	C	-4.9514600	-1.6267850	0.6273460
C	-6.1536070	-2.0006830	0.8239450	C	-6.4476140	-1.9516250	0.6158900
C	-6.2468490	-3.1547140	1.8219700	C	-6.8244040	-2.8273130	1.8096530
C	-5.3546480	-0.5294250	-2.0636690	C	-4.7935160	-1.4106520	-2.3137960
C	-4.9538690	-1.8143850	-2.7944470	C	-4.2739270	-2.8491100	-2.4206620
C	-5.7344420	-2.0113740	-4.0917490	C	-4.3849470	-3.3975460	-3.8400420
C	1.1200680	-4.0084170	-2.5311500	C	1.6685240	-3.6783610	-2.2782710
C	1.8785590	-4.9390320	-1.5817660	C	2.4491470	-4.3043660	-1.1179760
C	2.8970180	-5.8123210	-2.3107660	C	3.6055590	-5.1705440	-1.6106040
C	-0.8619630	-3.4229070	-0.4732320	C	-0.7606770	-3.4558860	-0.6400670
C	-1.9774420	-4.2722070	-1.0948080	C	-1.2632570	-4.7931350	-1.1979230
C	-2.7336610	-5.1094640	-0.0654440	C	-2.3034620	-5.4267000	-0.2777140
C	-2.6183700	0.0722040	4.2878340	C	-3.0648910	0.4284100	4.4709190
C	-3.1302240	1.5139600	4.3290550	C	-3.7377970	1.7987860	4.3371340
C	-4.2598760	1.6956580	5.3405720	C	-4.8574200	1.9756290	5.3601300
C	-0.9119870	-1.9950560	3.2098620	C	-1.1888470	-1.5817260	3.4465420
C	-2.0491740	-3.0048490	3.0498070	C	-2.1644600	-2.6427390	2.9274790
C	-1.5603830	-4.4424960	3.2067590	C	-1.6697640	-4.0552490	3.2248230
H	-1.0439270	2.8903580	1.7144660	H	-1.1367640	2.8768690	1.4996190
H	-1.9141180	2.0804900	-2.2511160	H	-1.5596640	1.4826050	-2.4134640
H	-0.1043470	6.1397050	0.8225920	H	-0.5858390	6.0671610	0.3021650
H	-0.8960520	5.2673690	-3.1384650	H	-1.0166530	4.7127700	-3.5790910
H	-2.3868690	2.2545580	0.0218280	H	-2.2780900	2.0285270	-0.2863870
H	0.8226770	6.6029860	-1.6172330	H	0.3905080	6.4365150	-2.1379870
H	-0.8836970	6.6370510	-1.2918570	H	-1.3130470	6.2361580	-1.8744180
H	2.4432860	6.0390350	-1.9237840	H	2.1024090	6.1436960	-2.2033360
H	3.8686210	5.0461570	-1.9642380	H	3.6557950	5.3750240	-2.1140690
H	2.8592780	0.6602450	-0.7048630	H	3.2416880	0.9790360	-0.5608290
H	2.6432750	4.0146370	-3.6252220	H	2.6994180	4.0751340	-3.7545330
H	1.7032060	0.8146250	-2.6116940	H	2.2300030	0.8432850	-2.6042100
H	3.5372920	2.2178770	-2.4218350	H	3.8077400	2.5419000	-2.3803050
H	4.0498780	2.8148880	-0.2467430	H	4.0531110	3.2972220	-0.2077670
H	3.4683720	5.0094450	0.2997130	H	3.1300890	5.4197060	0.1309940
H	2.6228200	1.8152960	1.3119970	H	2.6678550	2.2038260	1.3195310
H	-1.9435570	4.7141210	0.5793500	H	-2.1935860	4.3839370	0.1001620
H	-2.4139710	4.2858530	-1.6425630	H	-2.4356250	3.6348330	-2.0740780
H	-0.0457920	1.8111250	-3.2826760	H	0.3436040	1.4704170	-3.3563710
H	0.5091670	3.8366030	-3.9144930	H	0.6642770	3.4824770	-4.1397460
H	1.6692120	5.0922470	1.4734660	H	1.2429510	5.3327540	1.1717770
H	1.0454450	3.0770980	2.0377700	H	0.9258300	3.2907490	1.8741000
H	-3.4592590	-0.6116830	4.1227270	H	-3.8041630	-0.3720880	4.3554140
H	-2.1599860	-0.2082300	5.2443410	H	-2.6112120	0.3064760	5.4610030
H	-0.4676960	-2.0851580	4.2082440	H	-0.9957230	-1.7265830	4.5158090
H	-0.1191250	-2.1833370	2.4753880	H	-0.2200070	-1.6744400	2.9395580
H	0.6283400	0.7981790	2.4254560	H	0.3156910	1.1550650	2.7578470
H	-0.2901850	1.7621650	3.5763950	H	-0.7285270	2.2318510	3.6681810
H	-4.1904340	-1.3336270	1.5003660	H	-4.6687740	-1.0897980	1.5400260
H	-4.1426540	-2.4610220	0.1583520	H	-4.3619270	-2.5508880	0.6132360
H	-6.4144790	-0.5517170	-1.7821490	H	-5.8820080	-1.3683570	-2.4280020
H	-5.2149410	0.3370970	-2.7201600	H	-4.3574990	-0.7887130	-3.1039770
H	-4.4520970	1.4076510	1.1169190	H	-4.7966770	1.4544350	0.2774760
H	-6.1031190	1.0421810	0.5896500	H	-6.2374740	0.8461330	-0.5380530
H	0.3996220	-4.5698350	-3.1365380	H	1.2005600	-4.4491830	-2.9015840
H	1.8167140	-3.5304690	-3.2298960	H	2.3369750	-3.1024060	-2.9289140
H	-1.2805430	-2.6100450	0.1298450	H	-1.6029010	-2.7772120	-0.4578870
H	-0.2370470	-4.0283570	0.1945150	H	-0.2459040	-3.6016370	0.3167730
H	-1.5544820	-1.2877220	-2.4236390	H	-1.3810910	-1.3754970	-2.7747730
H	-1.4083640	-2.7640730	-3.3836580	H	-1.1027430	-2.9173950	-3.6024440
H	4.7097270	-3.3670330	1.5671160	H	4.6673650	-3.4892960	1.5186610
H	4.8969320	-2.0453700	2.7063090	H	4.6391220	-2.3288150	2.8392920
H	6.4656130	-0.0792830	0.1598060	H	6.3846970	0.2032470	1.2118900
H	5.0709210	0.8911050	-0.2941850	H	5.0452970	1.0606250	0.4748490
H	4.0296710	-2.9181590	-1.1434950	H	5.0966680	-2.7749580	-1.0360180
H	5.7165120	-2.3819670	-1.1338200	H	6.3141760	-1.5128700	-0.8841230
H	-2.8341600	-2.8087150	3.7896590	H	0.8573170	-0.2913510	1.2058540
H	-2.5120630	-2.8728880	2.0670030	H	-3.1528980	-2.4995890	3.3782490
H	-2.3064610	2.1930090	4.5780580	H	-2.2975750	-2.5180920	1.8475730
H	-3.4741670	1.7939320	3.3276080	H	-2.9929870	2.5917000	4.4687760
H	0.4296150	0.1614360	5.4220530	H	-4.1351020	1.9031890	3.3228310
H	1.3663510	-0.7377780	4.2332450	H	-0.2197310	0.8954300	5.7707140
H	4.5201530	1.2606530	2.1184460	H	0.8459870	-0.1413200	4.8290400
H	5.9202070	0.3184360	2.5991740	H	3.7226910	0.6692350	2.6730500
H	7.0197770	-2.8315240	0.6213250	H	5.2226290	0.1033080	3.3855180
H	7.1945790	-1.5758340	1.8352970	H	7.0871720	-2.8725450	1.0486300
H	3.3452970	-0.8340930	-2.2843050	H	7.0494270	-1.7477690	2.4004810

H	5.0223020	-0.2599770	-2.3130880	H	3.5061060	-1.0964690	-2.0649240
H	-2.6820220	-3.6134370	-1.6151550	H	4.7681280	0.1307540	-1.9861860
H	-1.5621530	-4.9432320	-1.8568010	H	-1.7019110	-4.6464720	-2.1913120
H	2.3882920	-4.3363300	-0.8209540	H	-0.4226660	-5.4827990	-1.3295170
H	1.1636520	-5.5750450	-1.0465380	H	2.8212290	-3.5078410	-0.4647750
H	0.5500760	-0.4263500	-3.6023850	H	1.7756460	-4.9135050	-0.5044130
H	0.4909080	-1.8251620	-4.6606680	H	0.8827430	-0.5901380	-3.7685910
H	-6.7147110	-1.1407610	1.2075210	H	0.8616130	-2.0334460	-4.7730990
H	-6.6356920	-2.2928720	-0.1163810	H	-7.0340120	-1.0272480	0.6413020
H	-3.8796500	-1.7797050	-3.0054360	H	-6.7114680	-2.4672970	-0.3143610
H	-5.1152020	-2.6773770	-2.1358340	H	-3.2262500	-2.8717890	-2.1047400
H	-4.1524880	2.4948280	-1.2682410	H	-4.8269580	-3.4938010	-1.7282050
H	-5.9066730	2.4582990	-1.3657510	H	-3.7999850	1.8391220	-2.1153620
H	2.4152570	-6.4178430	-3.0855280	H	-5.4150260	1.5398370	-2.7295350
H	3.6594860	-5.1938560	-2.7972120	H	3.2396360	-6.0105250	-2.2087200
H	3.4054790	-6.4906210	-1.6193720	H	4.2930820	-4.5939580	-2.2389570
H	-2.0605160	-5.8045700	0.4462180	H	4.1752980	-5.5751730	-0.7694870
H	-3.1963870	-4.4777480	0.6991500	H	-1.8758770	-5.6366730	0.7067490
H	-3.5268220	-5.6930960	-0.5421530	H	-3.1613240	-4.7602490	-0.1377970
H	-0.6228190	0.1442060	-5.7404740	H	-2.6721360	-6.3669330	-0.6953450
H	-1.8179840	-1.1272140	-5.4330600	H	-0.0862470	-0.0956860	-6.0266000
H	-1.7297710	0.2777830	-4.3627640	H	-1.3556170	-1.3002770	-5.7533690
H	-5.4341430	-2.9345980	-4.5957740	H	-1.2928080	0.1596210	-4.7556240
H	-6.8111520	-2.0664490	-3.8999090	H	-4.0197520	-4.4272620	-3.8874490
H	-5.5600060	-1.1788600	-4.7813770	H	-5.4226620	-3.3900770	-4.1870430
H	-5.1214150	4.6705220	-0.5121300	H	-3.7920460	-2.7947790	-4.5358680
H	-6.0194840	3.8369670	0.7682180	H	-5.0333250	3.9843350	-2.4565480
H	-4.2497580	3.8675870	0.8034690	H	-6.2906330	3.4572340	-1.3267600
H	-5.7184740	-4.0371070	1.4450770	H	-4.6468910	3.7569810	-0.7437810
H	-5.7912680	-2.8782580	2.7787610	H	-6.2709800	-3.7715700	1.7895990
H	-7.2863890	-3.4381030	2.0099810	H	-6.5920650	-2.3214430	2.7522550
H	-0.7963010	-4.6778020	2.4586920	H	-7.8923830	-3.0597260	1.8032810
H	-1.1192980	-4.5997960	4.1965890	H	-0.7046380	-4.2448330	2.7430760
H	-2.3804500	-5.1558030	3.0829920	H	-1.5419230	-4.2069320	4.3009150
H	1.8325260	2.2176840	4.9148210	H	-2.3832710	-4.7990190	2.8612240
H	2.8297400	0.8636930	5.4739860	H	1.2105180	2.8851610	5.2066860
H	2.7606340	1.2606710	3.7498570	H	2.1683900	1.6175180	5.9892160
H	-4.6086180	2.7322570	5.3604750	H	2.2685480	1.8504950	4.2375910
H	-5.1139180	1.0575120	5.0901650	H	-5.3291700	2.9559170	5.2530430
H	-3.9309490	1.4311650	6.3510350	H	-5.6307660	1.2121070	5.2290060
H	6.4425570	2.7545480	2.6898010	H	-4.4741760	1.8943610	6.3820740
H	7.4355400	1.9727680	1.4494080	H	4.8718240	2.5116610	3.9112620
H	6.0232880	2.9286120	0.9796730	H	6.3024450	2.3324380	2.8833490
H	3.9799970	-2.7942980	-3.6976930	H	4.7782370	2.9002220	2.1870970
H	4.4070520	-1.2798300	-4.5062330	H	4.9395590	-2.4905740	-3.5770430
H	5.6629230	-2.2529520	-3.7228300	H	4.6540780	-0.8800920	-4.2531760
H	6.8049240	-4.5628180	2.4316350	H	6.2124290	-1.2637270	-3.5033800
H	8.3254150	-3.6670340	2.5745310	H	6.6111910	-4.7796890	2.6138040
H	6.9734690	-3.2974010	3.6560820	H	8.0764960	-3.9069360	3.0882190
				H	6.5659650	-3.6480800	3.9743760
				H	-0.7499520	-0.8336080	0.4925070
	**III(Pd$_2$)$_2$**				**III(Pd$_2$)$_2$ (diprotonated)**		
C	-2.6193720	-2.2602060	5.4191610	C	-0.8875610	-3.6891030	5.3307850
C	-2.3116420	-0.7860600	5.1199810	C	-0.2324500	-2.2999610	5.2611080
C	-2.8618330	-0.6162710	3.6943870	C	0.7230380	-2.4670860	4.0726570
C	-2.9461250	-2.0453080	3.0670690	C	0.1528860	-3.6194080	3.1798240
C	-2.3951740	-3.0311720	4.1074580	C	-1.1068530	-4.1474490	3.8816220
C	-1.8822370	0.2008060	2.8146770	C	0.7159030	-1.1962040	3.2062430
C	-0.5750740	0.3714110	3.6744130	C	-0.4989890	-0.3278900	3.6607530
C	-0.8497280	-0.2500640	5.0980940	C	-1.0401290	-0.9887680	4.9894860
C	0.8825580	-0.0239110	3.2849570	C	-2.5946270	-0.9291630	4.9690390
C	1.3583780	-1.0560280	4.3194330	C	-2.9917480	-0.3615580	3.6009660
C	0.3937680	-1.1047830	5.5135060	C	-1.7505500	0.1573480	2.8295900
C	1.2847700	-2.4476560	3.6119130	C	-3.5877600	-1.5476600	2.7685110
C	0.3214320	-3.3187000	4.4338650	C	-2.7067190	-1.6903490	1.5116210
C	0.3213480	-2.6143420	5.7982270	C	-2.2194270	-0.2403620	1.4037520
C	1.2157090	-0.7428120	1.9785720	C	-3.4525470	-2.2000880	5.0544130
C	0.7317010	-2.2006610	2.1843430	C	-3.4901650	-2.8035760	3.6407020
C	-0.7324550	-2.7749810	2.1900610	C	-2.3227900	-3.6536140	3.0435330
C	-0.9802030	-3.4569330	3.5910830	C	-1.7764360	-2.9261250	1.7520880
C	-2.0477110	-2.0248490	1.8193970	C	-0.2061900	-2.9966880	1.8083220
C	-2.0776850	-0.5398260	1.4694940	C	0.7975270	-1.8249610	1.8196000
P	1.3525340	0.3714850	0.4527360	P	-1.8625880	0.5330820	-0.2177080
N	2.8122330	1.1428980	0.9086760	N	-3.1955600	0.2348420	-1.0568860
P	4.3244770	0.7308620	0.6992350	P	-4.0273170	1.0977540	-2.1128960
C	5.3959420	2.0294960	1.4018480	C	-3.1259460	1.5360330	-3.6383860
C	5.1215050	2.3300150	2.8766500	C	-1.9976160	2.5605880	-3.4735310
C	5.9523690	3.4943570	3.4259600	C	-1.1363230	2.7056390	-4.7313730
C	5.6275420	4.8407780	2.7786250	C	-1.8955970	3.2121060	-5.9556730
P	-1.5419210	-0.0227200	-0.2519960	P	1.2150940	-0.7524750	0.4329240
N	-2.6027530	-1.0432440	-1.0988290	N	2.6317650	-0.0493390	0.6504490
P	-3.9498180	-0.8382420	-1.8791750	P	4.1722100	-0.4398730	0.7865920
C	-5.2446220	0.1208460	-1.0094870	C	4.4868910	-2.1214910	1.4112970
C	-5.7406240	-0.5826130	0.2630660	C	3.9754600	-3.2102280	0.4541990
C	-6.1447560	0.3996220	1.3622920	C	3.7421500	-4.5512630	1.1490780
C	-6.6784030	-0.3000970	2.6093240	C	3.1038300	-5.5739810	0.2127880

N	1.8539560	-0.7885960	-0.6864520
P	1.3499220	-1.3557850	-2.0617300
C	2.8231150	-2.0807800	-2.8735390
C	2.6199210	-2.8166620	-4.1971490
C	3.9490190	-3.2160430	-4.8484770
C	4.7906840	-4.1662300	-3.9967960
C	0.6432670	-0.1880970	-3.2898880
C	1.6172240	0.9088440	-3.7204400
C	0.9806550	1.9531290	-4.6417750
C	0.6717870	1.4223050	-6.0407010
C	0.1199570	-2.7116560	-2.0540480
C	0.5371000	-3.9249090	-1.2265100
C	-0.6050620	-4.9207800	-1.0248960
C	-0.2046570	-6.0925350	-0.1320150
N	-2.2875160	1.5131540	-0.2816210
P	-1.5988850	2.9340740	-0.2410290
C	-1.2597710	3.5640250	1.4510450
C	-2.5466190	3.8372160	2.2411310
C	-2.3241480	3.9559030	3.7518180
C	-1.3222520	5.0405080	4.1431390
C	-2.7644570	4.1486770	-0.9538850
C	-2.2836410	5.6009910	-0.9659620
C	-3.3180360	6.5496860	-1.5730080
C	-2.8433710	8.0006140	-1.5948910
C	-0.0591770	3.1392770	-1.2318910
C	1.0585060	3.9808740	-0.6146000
C	2.3373080	3.9510870	-1.4481330
C	3.4381200	4.8282610	-0.8570860
C	-3.7993690	-0.0599180	-3.5361300
C	-3.2975750	1.3856640	-3.5075370
C	-2.9146500	1.9312480	-4.8844460
C	-4.0771390	2.0085350	-5.8722790
C	-4.6764060	-2.4776590	-2.2271630
C	-3.7260650	-3.4040490	-2.9955420
C	-4.1435900	-4.8760090	-2.9519250
C	-5.5105450	-5.1516330	-3.5760050
C	4.9299430	-0.8438140	1.4562460
C	4.5068110	-2.0968980	0.6810800
C	4.6763630	-3.3939030	1.4675690
C	4.1424430	-4.5969030	0.6917040
C	4.8217900	0.6117590	-1.0546720
C	6.2847960	0.2885670	-1.3746630
C	6.4492140	-0.3247000	-2.7706780
C	5.9074800	0.5509610	-3.8991190
H	-2.5207030	-2.5726050	1.0001430
H	-2.2773290	1.2080960	2.6701890
H	-3.0084620	-3.9375700	4.1391750
H	-2.8754810	-0.1663040	5.8248230
H	-3.1499150	-0.3532790	1.3036620
H	-2.1530740	-2.6705660	6.3105500
H	-3.7007140	-2.3106110	5.6075610
H	-0.4415080	-2.9404360	6.4996690
H	1.2794380	-2.8704800	6.2710260
H	2.2964160	-0.9071940	2.0785210
H	0.8388540	-0.6166810	6.3865890
H	1.4943800	0.8816400	3.3435540
H	2.3857840	-0.8487070	4.6365880
H	2.2830150	-2.8940550	3.5646880
H	0.7326950	-4.3248150	4.5656510
H	1.2962860	-2.8087850	1.4720760
H	-3.9819660	-2.2801390	2.7987540
H	-3.8519130	-0.1487310	3.6992560
H	-0.5191100	1.4538790	3.8243370
H	-0.8487350	0.5848300	5.8051660
H	-1.0769510	-4.5299270	3.3999650
H	-0.6788050	-3.5888100	1.4621150
H	-0.6447880	4.4671600	1.3946670
H	-0.6528300	2.7930050	1.9406990
H	-3.0042070	4.7653620	1.8751180
H	-3.2728340	3.0381710	2.0502830
H	-1.9864430	2.9882050	4.1436890
H	-3.2891760	4.1603810	4.2298900
H	-1.6160680	6.0122990	3.7306640
H	-1.2571600	5.1422040	5.2302350
H	-0.3187180	4.8106470	3.7716030
H	-2.9641910	3.8058590	-1.9754730
H	-3.7062470	4.0556360	-0.4016370
H	-1.3491360	5.6801400	-1.5360020
H	-2.0509630	5.9325960	0.0541750
H	-3.5517130	6.2203280	-2.5932090
H	-4.2520340	6.4739170	-1.0022690
H	-2.6308050	8.3583370	-0.5818020
H	-1.9253920	8.1037040	-2.1832400
H	-3.5981660	8.6604180	-2.0322050
H	-0.3574500	3.5390130	-2.2097100
H	0.3034760	2.1185280	-1.3797580
H	0.7288190	5.0197190	-0.4785240

N	0.9534580	-1.5931450	-0.8797490
P	0.7169610	-1.8671120	-2.4178680
C	0.9482000	-0.4225860	-3.4975620
C	2.3599420	0.1669550	-3.4421440
C	2.5076130	1.4377550	-4.2834140
C	2.2898920	1.2209850	-5.7792190
C	-0.9833210	-2.4456350	-2.7097180
C	-1.2853550	-3.7685620	-1.9971780
C	-2.7854970	-4.0387560	-1.8852310
C	-3.0928090	-5.3813840	-1.2278660
C	1.8542710	-3.1777580	-2.9465770
C	1.7266290	-3.6177200	-4.4072760
C	2.6720480	-4.7725930	-4.7548800
C	2.3730200	-6.0635100	-3.9934900
N	-1.5152240	2.0818280	-0.0173060
P	-0.3347390	3.1147500	0.2380660
C	-0.9917290	4.7625180	-0.1453840
C	0.0008400	5.9210630	-0.0197370
C	-0.6766780	7.2746000	-0.2370760
C	0.3059450	8.4385410	-0.1394970
C	1.1244210	2.8638710	-0.8416550
C	2.4762410	3.3513450	-0.3036770
C	3.5304310	3.3793180	-1.4098350
C	4.9114750	3.8034220	-0.9207330
C	0.2233200	3.1356240	1.9737040
C	-0.7987680	3.7858640	2.9188830
C	-0.5398690	3.4810710	4.3986620
C	0.8958440	3.7487310	4.8468730
C	4.9989030	-0.2828060	-0.8203520
C	6.5201160	-0.4450410	-0.8287950
C	7.1024550	-0.3085280	-2.2401730
C	6.8475180	1.0543470	-2.8835790
C	4.9350120	0.7639940	1.9138260
C	4.0856680	1.0538280	3.1566520
C	4.5930650	2.2536920	3.9631770
C	4.4672560	3.5869770	3.2256400
C	-5.4192440	0.0614280	-2.6440430
C	-4.9613450	-1.2302520	-3.3356540
C	-6.0666810	-2.2842700	-3.4318660
C	-7.2785850	-1.8360870	-4.2466080
C	-4.7185340	2.6344730	-1.4233610
C	-5.4612890	2.4191170	-0.0856450
C	-4.6907320	2.9603390	1.1209960
C	-5.3616570	2.6132100	2.4461790
H	-3.3252530	-1.9109960	0.6381510
H	-1.7338350	1.2483280	2.8628560
H	-4.4008460	-3.4022710	3.5505520
H	-2.9045330	-0.2488750	5.7672870
H	-3.1888890	0.2921340	1.3968710
H	-3.2144840	-2.8801940	5.8660000
H	-4.4763420	-1.8577080	5.2545130
H	-1.7400620	-3.7896990	5.9947840
H	-0.1186830	-4.3553650	5.7434890
H	1.7527550	-2.3738310	1.8527180
H	0.3312770	-2.1422640	6.1847860
H	1.6185950	-0.6060720	3.3847880
H	1.7391380	-2.7093180	4.3967420
H	0.9039780	-4.4050870	3.0569130
H	-1.1222620	-5.2407870	3.8644720
H	0.1021760	-3.6660710	1.0011710
H	-4.6211180	-1.3248940	2.4870300
H	-3.7313290	0.4375260	3.6980240
H	-0.0322020	0.6130860	3.9608770
H	-0.7456300	-0.3177190	5.8003400
H	-2.8079900	-4.5675130	2.6916080
H	-2.0143890	-3.5816570	0.9123470
H	1.1815900	3.6595280	2.0295860
H	0.4215940	2.0919460	2.2461300
H	-0.7712630	4.8713150	2.7677440
H	-1.8147200	3.4626270	2.6605680
H	-0.7969650	2.4358530	4.6076890
H	-1.2314100	4.0863560	4.9940810
H	1.2084840	4.7670620	4.5911400
H	0.9946430	3.6270610	5.9286840
H	1.5993630	3.0547200	4.3746480
H	-1.3813990	4.6954350	-1.1674350
H	-1.8564520	4.9087140	0.5116150
H	0.8054360	5.8016300	-0.7551710
H	0.4750740	5.9139200	0.9701520
H	-1.1620680	7.2786540	-1.2205360
H	-1.4748580	7.3981680	0.5048200
H	0.7831740	8.4669710	0.8456080
H	1.0968220	8.3480920	-0.8913370
H	-0.1987060	9.3957680	-0.2960560
H	0.8782380	3.3673050	-1.7849880
H	1.1906120	1.7954830	-1.0670920
H	2.3909780	4.3560690	0.1257420

H	1.3008890	3.5785110	0.3747990
H	2.1236780	4.2578120	-2.4804890
H	2.6843220	2.9125710	-1.4850250
H	3.6407710	4.5432900	0.1814380
H	3.1486390	5.8847560	-0.8582090
H	4.3723700	4.7346390	-1.4195530
H	-6.0710670	0.3537810	-1.6902470
H	-4.7363560	1.0567980	-0.7509110
H	-4.9507950	-1.2332420	0.6616540
H	-6.5857810	-1.2381860	0.0201360
H	-6.8975150	1.0973230	0.9741140
H	-5.2666990	1.0050320	1.6250400
H	-7.5776760	-0.8817630	2.3800190
H	-5.9317180	-0.9905020	3.0173880
H	-6.9333020	0.4185270	3.3937480
H	-3.0928640	-0.6870930	-4.0948240
H	-4.7701270	-0.1388690	-4.0399270
H	-2.4338690	1.4470090	-2.8382720
H	-4.0679320	2.0278000	-3.0612130
H	-2.4831360	2.9317120	-4.7532290
H	-2.1158500	1.3071470	-5.3044890
H	-3.7628240	2.4613410	-6.8171830
H	-4.4781340	1.0157170	-6.0994450
H	-4.8953190	2.6129250	-5.4650950
H	-4.9127030	-2.9144840	-1.2492190
H	-5.6253780	-2.3378520	-2.7571510
H	-3.6613910	-3.0769540	-4.0412570
H	-2.7219360	-3.3065550	-2.5705260
H	-4.1395280	-5.2153280	-1.9078810
H	-3.3809380	-5.4691320	-3.4706070
H	-5.7362160	-6.2219670	-3.5765870
H	-6.3113790	-4.6453410	-3.0278300
H	-5.5434930	-4.8002100	-4.6132710
H	3.2710380	-2.7358440	-2.1185400
H	3.5304490	-1.2571510	-3.0193240
H	2.0118810	-3.7159930	-4.0368410
H	2.0614040	-2.1813160	-4.8954410
H	4.5274030	-2.3076160	-5.0633980
H	3.7395860	-3.6864770	-5.8159190
H	4.2153940	-5.0573970	-3.7215810
H	5.6816270	-4.4947580	-4.5393680
H	5.1275740	-3.6900780	-3.0702170
H	0.2909430	-0.7698660	-4.1494920
H	-0.2419990	0.2440560	-2.8077480
H	2.4789040	0.4615050	-4.2344320
H	2.0117220	1.4006320	-2.8256390
H	0.0591100	2.3251460	-4.1765300
H	1.6553820	2.8133860	-4.7240900
H	0.2028210	2.1914960	-6.6613860
H	1.5884550	1.0944110	-6.5429800
H	-0.0103770	0.5666070	-6.0070610
H	-0.0597920	-3.0003910	-3.0980220
H	-0.8174210	-2.2834430	-1.6788260
H	1.3887490	-4.4364130	-1.6965080
H	0.8870970	-3.5850360	-0.2462570
H	-0.9476420	-5.2907220	-2.0001890
H	-1.4592060	-4.3934430	-0.5801190
H	0.6393850	-6.6435730	-0.5608770
H	-1.0316370	-6.7954140	0.0046790
H	0.1017130	-5.7371000	0.8589160
H	5.2202610	2.9166890	0.7835050
H	6.4420690	1.7355380	1.2523110
H	5.3237910	1.4316320	3.4724790
H	4.0555700	2.5540410	2.9987760
H	7.0197910	3.2725070	3.2999660
H	5.7774350	3.5656440	4.5057160
H	5.8829140	4.8543300	1.7143540
H	4.5589010	5.0665560	2.8665480
H	6.1828380	5.6517850	3.2586380
H	6.0204210	-0.7962090	1.5655580
H	4.5133270	-0.8648490	2.4710250
H	5.0882770	-2.1628240	-0.2492120
H	3.4614710	-1.9925570	0.3682590
H	5.7327800	-3.5484040	1.7227110
H	4.1376390	-3.3071530	2.4200720
H	4.2726470	-5.5296380	1.2484290
H	3.0737810	-4.4759610	0.4806790
H	4.6577400	-4.7040250	-0.2697960
H	4.1494380	-0.1558760	-1.4454390
H	4.5126830	1.5547520	-1.5186650
H	6.8909260	1.1994480	-1.2964850
H	6.6931210	-0.4161400	-0.6410080
H	5.9373330	-1.2964220	-2.7887690
H	7.5108530	-0.5322500	-2.9468680
H	6.3885520	1.5352830	-3.8981140
H	6.0851620	0.0893770	-4.8748960
H	4.8286060	0.7112810	-3.8002960
H	2.7964770	2.6799010	0.5017240
H	3.1965130	4.0586080	-2.2035250
H	3.5905840	2.3863720	-1.8702950
H	5.3264280	3.0782200	-0.2125190
H	4.8707830	4.7739350	-0.4160650
H	5.6145390	3.8834540	-1.7540700
H	-5.3690310	3.0621720	-2.1931090
H	-3.8808380	3.3265330	-1.2879660
H	-5.6611200	1.3529280	0.0810440
H	-6.4417260	2.9027980	-0.1383160
H	-4.5924780	4.0491520	1.0234080
H	-3.6695420	2.5654090	1.1033060
H	-6.3669870	3.0426980	2.5099070
H	-5.4595570	1.5271880	2.5588700
H	-4.7807230	2.9871190	3.2946340
H	-2.7238360	0.5920510	-4.0263260
H	-3.8733190	1.8865040	-4.3589960
H	-1.3563490	2.2690440	-2.6355450
H	-2.4229780	3.5370380	-3.2116540
H	-0.3127920	3.3934150	-4.5068520
H	-0.6752650	1.7370340	-4.9617310
H	-1.2145200	3.3669120	-6.7968890
H	-2.6620590	2.5017030	-6.2800530
H	-2.3902340	4.1656050	-5.7420630
H	-5.9890590	-0.1735820	-1.7376800
H	-6.0655360	0.6635810	-3.2905500
H	-4.5939400	-0.9955440	-4.3419440
H	-4.1193560	-1.6522120	-2.7780050
H	-6.3830220	-2.5618020	-2.4185450
H	-5.6397320	-3.1885130	-3.8807150
H	-8.0083360	-2.6449650	-4.3407980
H	-7.7866670	-0.9868410	-3.7789190
H	-6.9799970	-1.5324520	-5.2558430
H	1.6648280	-4.0080980	-2.2580670
H	2.8686750	-2.8243140	-2.7304440
H	0.6948070	-3.9276900	-4.6151870
H	1.9407130	-2.7685950	-5.0661640
H	3.7064820	-4.4605040	-4.5660700
H	2.5965120	-4.9622630	-5.8308710
H	1.3293710	-6.3658670	-4.1330930
H	3.0095220	-6.8792250	-4.3467730
H	2.5486140	-5.9561100	-2.9184880
H	0.6763110	-0.7290390	-4.5133370
H	0.2075810	0.3236050	-3.1836380
H	3.0875750	-0.5786140	-3.7872250
H	2.6103810	0.3878230	-2.3974800
H	1.8094540	2.1983650	-3.9111490
H	3.5138410	1.8413290	-4.1246020
H	2.4787830	2.1433270	-6.3348000
H	2.9662560	0.4500600	-6.1634840
H	1.2656120	0.9089900	-6.0053880
H	-1.1494590	-2.5266740	-3.7894710
H	-1.6477350	-1.6520910	-2.3449510
H	-0.8000900	-4.5947720	-2.5313170
H	-0.8511660	-3.7477270	-0.9907460
H	-3.2391980	-4.0021880	-2.8835210
H	-3.2464060	-3.2283770	-1.3055070
H	-2.6746200	-6.2082780	-1.8108660
H	-4.1710300	-5.5402430	-1.1413680
H	-2.6641910	-5.4356080	-0.2208180
H	5.0622410	1.6743950	1.3189350
H	5.9367860	0.4147040	2.1843040
H	4.0617080	0.1649340	3.7973840
H	3.0531790	1.2452110	2.8401830
H	5.6366960	2.0879930	4.2561720
H	4.0153810	2.3051830	4.8929470
H	5.0975440	3.6282110	2.3317660
H	3.4327690	3.7625490	2.9097850
H	4.7627430	4.4184720	3.8710450
H	5.5596970	-2.2302570	1.6018350
H	3.9900970	-2.1865740	2.3867700
H	4.6904880	-3.3391830	-0.3667930
H	3.0328950	-2.9009730	-0.0148720
H	4.6914030	-4.9340360	1.5410480
H	3.0889230	-4.3958360	2.0180360
H	2.9630440	-6.5371670	0.7100400
H	2.1212500	-5.2258630	-0.1266630
H	3.7267660	-5.7392120	-0.6729070
H	4.5252100	-1.0112790	-1.4891930
H	4.7090370	0.7087660	-1.1823830
H	6.9761320	0.3093710	-0.1761910
H	6.7923000	-1.4252160	-0.4215760
H	6.6887100	-1.1004850	-2.8764280
H	8.1815860	-0.4864240	-2.1841520
H	7.2025040	1.8647520	-2.2377090
H	7.3667240	1.1353070	-3.8421840
H	5.7825280	1.2231680	-3.0760720

				H	0.3773630	0.3606430	0.5484960
				H	-0.8488410	-0.1203990	-0.9171290
**III(Pe$_2$)$_2$**				**III(Pe$_2$)$_2$ (diprotonated)**			
C	2.1073870	1.5991520	-4.3577810	C	-2.4986830	2.3225520	4.4771200
C	1.1867610	0.3897720	-4.0887130	C	-1.5796570	1.2839760	3.7968310
C	2.1100470	-0.8618830	-4.0492660	C	-2.2007410	-0.0870540	4.1193310
C	3.5428480	-0.2998510	-4.0853490	C	-3.7073880	0.2056520	4.1446550
C	3.4077420	0.9928460	-4.8937950	C	-3.7962440	1.5668560	4.8503920
P	1.7477090	-1.9179200	-2.5743940	P	-1.6299320	-1.3823870	2.9542560
C	-0.0710580	-2.2157820	-2.7240610	C	0.1799090	-1.5103130	3.2607890
C	-0.6416890	-3.1787020	-1.6472430	C	0.8875760	-2.5716270	2.3719690
C	-1.2827820	-4.3622220	-2.4067040	C	1.4788210	-3.6315760	3.3312480
C	-0.7399220	-4.2872300	-3.8395590	C	0.8385050	-3.3754660	4.7018700
C	-0.5515320	-2.7865290	-4.0796330	C	0.5731870	-1.8680080	4.7149260
N	2.2550880	-1.3518650	-1.1898010	N	-1.9687220	-1.0701520	1.4322220
P	1.6487660	0.0096770	-0.3335050	P	-1.7343180	-0.0895290	0.1926110
N	2.9612470	1.0458980	-0.0959130	N	-2.8911070	0.9522230	-0.1733760
P	4.4951990	1.1346480	0.2224550	P	-4.4627320	0.9951560	-0.4519240
C	5.1506940	2.6739000	-0.5451430	C	-5.0561160	2.6167660	0.1384840
C	4.2011600	3.8897220	-0.3313160	C	-4.1231970	3.7932260	-0.2797880
C	3.8410610	4.3997120	-1.7385710	C	-3.7649720	4.5279760	1.0279430
C	4.9942760	3.9266700	-2.6258370	C	-4.8819370	4.1469010	2.0015120
C	5.2888080	2.5287710	-2.0782090	C	-5.1050740	2.6692910	1.6822250
C	1.6743150	-0.8945230	1.3462140	C	-1.5348180	-1.2311150	-1.2295330
C	1.5791110	-0.1856770	2.7055380	C	-1.4425780	-0.8111910	-2.7061220
C	2.3010780	-1.2227300	3.5905760	C	-2.1585090	-2.0272220	-3.3344770
C	2.0951580	-2.6115080	2.8989970	C	-1.9396500	-3.2329700	-2.3572960
C	1.2415550	-2.3523400	1.6343330	C	-1.0843000	-2.7091690	-1.1779240
C	0.2577200	0.1918410	3.4588550	C	-0.1209360	-0.6048020	-3.5235440
C	0.3375930	-0.4697350	4.8914260	C	-0.2016080	-1.5688410	-4.7737280
C	1.6532980	-1.3058730	4.9785740	C	-1.5131940	-2.4072530	-4.6711850
C	-1.2241430	0.0476710	2.9604850	C	1.3645450	-0.6345210	-3.0239860
C	-1.9491380	-0.8283490	4.0089190	C	2.0914060	-1.7224720	-3.8464880
C	-1.0689440	-1.0393130	5.2501230	C	1.2048020	-2.2034440	-5.0027700
C	-1.7181720	-0.6409650	1.6748050	C	1.8673630	-1.0325960	-1.6266230
C	-1.5601120	-2.1412330	1.9333510	C	1.7197920	-2.5536930	-1.5247660
C	-2.1553460	-2.2343880	3.3522560	C	2.3098730	-2.9475180	-2.8947280
C	-1.3755810	-3.2481470	4.2017600	C	1.5311660	-4.1259990	-3.4927180
C	-0.1115460	-3.6306770	3.3670250	C	0.2715630	-4.3198720	-2.5911630
C	-0.1942930	-2.9027820	1.9678810	C	0.3552100	-3.3049020	-1.3826350
C	1.3464230	-3.5009920	3.9023550	C	-1.1866470	-4.3139660	-3.1416120
C	-1.2896530	-2.5282040	5.5571560	C	1.4341360	-3.7225460	-4.9724150
C	1.6627970	-2.8202420	5.2426350	C	-1.5125230	-3.9422820	-4.5965790
P	-1.6333280	0.0711090	-0.0570330	P	1.8105420	0.0398700	-0.1716140
N	-2.1902010	1.6218490	0.4106410	N	1.6400840	1.5068730	-0.7620030
P	-1.8182930	3.0237740	-0.2093870	P	1.4866960	3.0577120	-0.4370160
C	-0.0478680	3.5382060	-0.0489270	C	-0.2463710	3.5742860	-0.7318430
C	0.2603040	5.0428540	-0.2660540	C	-0.4793100	5.1065480	-0.8331620
C	0.4354710	5.6330610	1.1374080	C	-0.6555810	5.3940780	-2.3285660
C	1.0994970	4.5041970	1.9307350	C	-1.3746690	4.1514560	-2.8575830
C	0.4929070	3.2025640	1.3725400	C	-0.7591510	2.9763510	-2.0780910
N	-2.9549200	-0.7139940	-0.7554730	N	3.0189200	-0.2760450	0.8285240
P	-4.5117480	-0.8643270	-0.5997820	P	4.6013420	-0.4752820	0.8383020
C	-5.2283630	-1.0057730	-2.2892350	C	5.1524880	-0.2037360	2.5549940
C	-4.3719580	-1.9107490	-3.2247810	C	4.2569480	-0.9219490	3.6095520
C	-3.9963650	-1.0298610	-4.4322340	C	3.7697240	0.1839610	4.5653000
C	-5.0868130	0.0417450	-4.4749950	C	4.8022780	1.3033900	4.4227320
C	-5.2821410	0.3717550	-2.9944860	C	5.1023180	1.3005720	2.9232930
C	-2.2964560	3.1719690	-1.9810340	C	2.0068490	3.4454190	1.2670560
C	-2.1206840	4.4841550	-2.7555720	C	1.8056500	4.8664850	1.8125830
C	-2.4767340	4.0688550	-4.1918360	C	2.1135890	4.6687330	3.3001320
C	-1.9487130	2.6177290	-4.3321280	C	1.4097600	3.3438970	3.6496540
C	-1.6020560	2.1536940	-2.8989100	C	1.3414550	2.5391340	2.3251750
C	-2.8066150	4.3049340	0.6617700	C	2.5312320	4.0505530	-1.5648620
C	-2.8106120	4.1256530	2.1993720	C	2.5680500	3.5268080	-3.0233300
C	-4.1963330	4.6236650	2.6859610	C	3.9783430	3.8842910	-3.5596590
C	-4.9344740	5.1096510	1.4260510	C	4.6567550	4.6934040	-2.4430770
C	-4.2974940	4.3099830	0.2874800	C	4.0100910	4.1646630	-1.1615860
C	-4.9698740	-2.3776910	0.3372310	C	5.0421230	-2.1550530	0.2750530
C	-4.4234010	-3.6849380	-0.2544800	C	4.4793630	-3.3022310	1.1283770
C	-5.0325570	-4.7472110	0.6690130	C	5.1247460	-4.5323140	0.4801690
C	-6.4295190	-4.1957750	1.0533920	C	6.5353830	-4.0660130	0.0430640
C	-6.4470140	-2.7171950	0.5884420	C	6.5317890	-2.5202740	0.1538590
C	-5.4249660	0.5341930	0.1751600	C	5.4899140	0.7365580	-0.1955160
C	-6.9722390	0.5757690	-0.0128310	C	7.0367020	0.8191280	-0.0173580
C	-7.5559760	0.8690530	1.3839260	C	7.6140630	0.8149310	-1.4468720
C	-6.4016490	1.5200560	2.1503010	C	6.4581420	1.2888610	-2.3312090
C	-5.1791940	0.7229890	1.6874230	C	5.2406240	0.5954370	-1.7141950
C	2.6157880	-3.5002560	-2.9129290	C	-2.4451720	-2.9505830	3.4181830
C	4.1662630	-3.3535420	-2.9208230	C	-3.9938720	-2.9260770	3.2399900
C	4.7165400	-4.6078810	-2.2010210	C	-4.3700940	-4.2939680	2.6264230
C	3.5319490	-5.5738380	-2.1114540	C	-3.1365250	-5.1749970	2.8290060
C	2.3446410	-4.6337650	-1.9044450	C	-1.9858120	-4.1915140	2.6234330
C	4.8595030	1.2481070	2.0215430	C	-4.8186080	0.8119510	-2.2331470
C	4.2602020	2.4682470	2.7363520	C	-4.2435900	1.9002850	-3.1518410
C	4.7764540	2.2941930	4.1700270	C	-4.8396280	1.5239630	-4.5139170

C	6.1886990	1.6723160	4.0150500
C	6.3106120	1.2608610	2.5260480
C	5.5471650	-0.2251200	-0.4342870
C	7.0833380	0.0230450	-0.5188760
C	7.7442560	-1.2564250	0.0360060
C	6.6575170	-2.3292770	-0.0769930
C	5.3783190	-1.5747560	0.2945460
H	-2.1968080	-2.6740130	1.2219830
H	-1.6459950	1.0559760	2.9506970
H	-1.9598780	-4.1640040	4.3394220
H	-1.4497120	-0.4558340	6.0948230
H	-2.8040800	-0.5078450	1.7960200
H	-0.6250810	-2.9676100	6.2957790
H	-2.2955660	-2.5980200	5.9937510
H	1.0917390	-3.1647280	6.1003330
H	2.7076300	-3.0745590	5.4685610
H	2.7619640	-1.0558200	1.2747900
H	2.2910770	-0.8257790	5.7284030
H	2.1694510	0.7301060	2.6456400
H	3.3711720	-0.9996670	3.6663980
H	3.0653670	-3.0398700	2.6259760
H	1.7773580	-4.5074510	3.9197110
H	1.6120740	-2.9519260	0.7996650
H	-3.2232340	-2.4781860	3.3140980
H	-2.9189430	-0.3929460	4.2715180
H	0.3604680	1.2687740	3.6188770
H	0.4911410	0.3490620	5.6005140
H	-0.2357890	-4.6987940	3.1657680
H	-0.3294120	-3.6953490	1.2272530
H	-2.0547760	4.7470810	-4.9382230
H	-3.5650210	4.0865800	-4.3149280
H	-2.7067860	1.9682840	-4.7804350
H	-1.0671980	2.5693230	-4.9782130
H	-1.9086600	1.1266160	-2.6863820
H	-0.5193310	2.2095370	-2.7432610
H	-1.0728970	4.8010330	-2.7174550
H	-2.7361600	5.3056700	-2.3767060
H	-4.1118500	5.4131600	3.4374470
H	-1.9853340	4.6668210	2.6691530
H	-2.6831430	3.0647960	2.4321040
H	2.1801010	4.5251680	1.7538910
H	-4.4911070	4.7380810	-0.7001280
H	0.9502600	4.6008890	3.0105850
H	-4.7492070	6.1782410	1.2665370
H	-0.3086540	2.8338890	2.0183430
H	-4.6796380	3.2834080	0.2943420
H	1.2350610	2.4037180	1.3014030
H	-0.5418900	5.8766070	1.5683000
H	1.1997960	5.1328170	-0.8226670
H	1.0236350	6.5554220	1.1307720
H	-0.4992220	5.5770570	-0.8420390
H	-5.0833450	-5.7324190	0.1981000
H	-4.4071200	-4.8459460	1.5632330
H	-4.7980200	-3.8143870	-1.2773250
H	-7.3859530	-0.3447040	-0.4314660
H	-7.2301960	1.3780940	-0.7102300
H	-6.2992010	2.5687740	1.8453720
H	-8.4521120	1.4939440	1.3383170
H	-7.8381450	-0.0672590	1.8795130
H	-6.5386230	1.5024460	3.2354940
H	-4.2223880	1.2227310	1.8584240
H	-5.1598470	-0.2413980	2.2121410
H	-7.2315750	-4.7619670	0.5722520
H	-7.0157760	-2.6279010	-0.3440440
H	-6.5925900	-4.2672600	2.1322150
H	-3.4773800	-2.2775290	-2.7168020
H	-4.9606300	-2.7763240	-3.5441870
H	-3.9095290	-1.6013700	-5.3606900
H	-3.0278820	-0.5469680	-4.2490280
H	-4.8134420	0.9169730	-5.0723860
H	-6.2074840	0.9136680	-2.7848810
H	-4.4427260	0.9959360	-2.6615830
H	-6.9127950	-2.0526880	1.3200740
H	-6.0112600	-0.3740590	-4.8940920
H	-4.7458130	3.7980470	3.1491380
H	-6.0182420	4.9711360	1.4879190
H	-3.3323310	-3.7125430	-0.2945240
H	-6.2438860	-1.4080550	-2.2084710
H	-4.9809550	1.3971990	-0.3318020
H	-4.4763660	-2.2280290	1.3084780
H	0.4835860	2.9230610	-0.7829140
H	-3.3702950	2.9334450	-1.9567510
H	-2.3881450	5.2842610	0.3996680
H	5.0336290	-4.3412350	-1.1874890
H	5.5836940	-5.0364500	-2.7106890
H	3.4093130	-6.1216140	-3.0539580
H	3.6395810	-6.3098330	-1.3095530
C	-6.2480280	0.9614830	-4.2007740
C	-6.2938140	0.7482360	-2.6674070
C	-5.4045690	-0.2937790	0.4399320
C	-6.9441730	-0.0710400	0.5526250
C	-7.5852040	-1.4241360	0.1843110
C	-6.4728030	-2.4514370	0.4050410
C	-5.2205460	-1.7297780	-0.1023530
H	2.3624430	-2.9154700	-0.7180330
H	1.7818800	0.3494330	-3.2457330
H	2.1195660	-5.0454790	-3.4261430
H	1.5805090	-1.8195860	-5.9553820
H	2.9548970	-0.9225260	-1.7770730
H	0.7714730	-4.3193850	-5.5909070
H	2.4380360	-3.8800230	-5.3882090
H	-0.9437010	-4.4608480	-5.3616700
H	-2.5554880	-4.2466020	-4.7549940
H	-2.6252700	-1.4091660	-1.1601630
H	-2.1553860	-2.1051380	-5.5034080
H	-2.0405790	0.0921740	-2.8450080
H	-3.2291770	-1.8297580	-3.4504300
H	-2.9043260	-3.5966730	-1.9910720
H	-1.6134590	-5.2996170	-2.9361110
H	-1.4575000	-3.1168990	-0.2374810
H	3.3781460	-3.1691920	-2.8056750
H	3.0564110	-1.3499640	-4.2017520
H	-0.2275370	0.4110100	-3.9111380
H	-0.3575640	-0.9246750	-5.6423890
H	0.3982110	-5.3149670	-2.1581360
H	0.4954200	-3.9128200	-0.4854250
H	1.7741210	5.5036140	3.9173410
H	3.1967330	4.5722460	3.4363140
H	1.9369410	2.7942910	4.4341060
H	0.3994130	3.5420930	4.0193060
H	1.8500450	1.5729040	2.3863340
H	0.2988260	2.3402120	2.0582620
H	0.7613560	5.1759820	1.6958610
H	2.4410520	5.6121390	1.3274400
H	3.9311760	4.4397060	-4.4988620
H	1.7719400	3.9731610	-3.6234760
H	2.4155140	2.4441420	-3.0365070
H	-2.4448890	4.2336330	-2.6433250
H	4.1704790	4.8108320	-0.2952070
H	-1.2701700	4.0248340	-3.9383350
H	4.4254210	5.7589640	-2.5502260
H	0.0540300	2.5213560	-2.6493340
H	4.4138550	3.1748230	-0.9268420
H	-1.4775920	2.1803830	-1.8746430
H	0.3200770	5.5037100	-2.8147750
H	-1.4008700	5.3513920	-0.2965410
H	-1.2106260	6.3181380	-2.5075110
H	0.3122310	5.7114550	-0.3843230
H	5.1537120	-5.3879560	1.1583770
H	4.5349560	-4.8273520	-0.3942320
H	4.8207490	-3.1980530	2.1644330
H	7.4512000	0.0079300	0.5847360
H	7.2854990	1.7496360	0.4987390
H	6.3474960	2.3761950	-2.2481180
H	8.5044870	1.4415740	-1.5333440
H	7.9009590	-0.2020870	-1.7364040
H	6.5917310	1.0452200	-3.3878970
H	4.2851470	1.0356070	-2.0165860
H	5.2368480	-0.4575790	-2.0222410
H	7.3136680	-4.4916870	0.6806910
H	7.0686780	-2.2101770	1.0567330
H	6.7504170	-4.3817340	-0.9805740
H	3.4111960	-1.4365040	3.1497820
H	4.8490850	-1.6697150	4.1432720
H	3.6595460	-0.1724650	5.5924950
H	2.7893890	0.5498070	4.2361270
H	4.4386080	2.2730420	4.7739990
H	6.0219170	1.8258580	2.6560950
H	4.2708830	1.7884750	2.4005110
H	7.0131060	-2.0374100	-0.6991200
H	5.7084510	1.0582180	4.9880840
H	4.5436810	2.9680030	-3.7557350
H	5.7453720	4.5927650	-2.4416850
H	3.3883820	-3.3415980	1.1367530
H	6.1838490	-0.5631760	2.6307190
H	5.0558040	1.6860850	0.1340760
H	4.5853720	-2.2272410	-0.7226050
H	-0.8230930	3.1554410	0.1014350
H	3.0855660	3.2385610	1.2589160
H	2.1091520	5.0618490	-1.5502520
H	-4.5547750	-4.1826830	1.5529620
H	-5.2766570	-4.7102080	3.0718270
H	-3.1071340	-5.5718110	3.8504550
H	-3.0958840	-6.0207600	2.1381720

H	1.3800820	-5.1222060	-2.0557630	H	-1.0226880	-4.5835360	2.9506350
H	2.3645700	-4.2312340	-0.8845570	H	-1.9102290	-3.9435920	1.5589900
H	4.4753940	-2.4450140	-2.3967390	H	-4.3124630	-2.1093360	2.5879330
H	4.5358490	-3.2836680	-3.9471660	H	-4.4786650	-2.7771500	4.2067420
H	-2.3717840	-4.2436670	-2.4267060	H	2.5623540	-3.4984180	3.4085640
H	-1.3862590	-2.6420250	-1.0539440	H	1.6788640	-2.0857570	1.7978630
H	0.1341850	-3.5129590	-0.9558680	H	0.2043100	-3.0130340	1.6429010
H	3.3049280	0.7557190	-5.9597740	H	-3.8423050	1.4109480	5.9322840
H	-1.5182650	-2.3341120	-4.3259790	H	1.4959290	-1.3330310	4.9586110
H	4.2701150	1.6577190	-4.7837020	H	-4.6987850	2.1139530	4.5650430
H	-1.4103700	-4.7465970	-4.5719210	H	1.4819630	-3.6850830	5.5289300
H	3.8591840	-0.0677860	-3.0620970	H	-4.0787230	0.2839330	3.1184160
H	0.1163550	-2.5749250	-4.9193370	H	-0.1640560	-1.5758740	5.4668700
H	4.2640650	-1.0013840	-4.5107280	H	-4.2877030	-0.5663150	4.6515260
H	2.3113050	2.1160750	-3.4123660	H	-2.6957500	3.1573710	3.7987020
H	0.4222140	0.2753880	-4.8630180	H	-0.5418350	1.3546090	4.1292870
H	1.6589350	2.3286150	-5.0381370	H	-2.0224200	2.7422480	5.3665780
H	0.6624710	0.5244310	-3.1414020	H	-1.5959160	1.4434630	2.7118510
H	4.7881950	3.2340280	4.7281020	H	-4.8665780	2.3737450	-5.1996180
H	4.1161830	1.6040620	4.7074420	H	-4.2170200	0.7505200	-4.9760410
H	4.6662560	3.3886420	2.2998680	H	-4.6075430	2.8848090	-2.8373390
H	7.4109890	0.9076600	0.0320890	H	-7.3164790	0.7324180	-0.0867120
H	7.3686860	0.1837750	-1.5623160	H	-7.1895390	0.2080380	1.5802110
H	6.5907000	-2.6787860	-1.1146150	H	-6.3711500	-2.6637280	1.4754590
H	8.6622270	-1.5160820	-0.4975450	H	-8.4798280	-1.6298040	0.7764350
H	8.0106670	-1.1171270	1.0901290	H	-7.8848060	-1.4274320	-0.8693200
H	6.8429980	-3.2015010	0.5566920	H	-6.6497340	-3.3996650	-0.1084330
H	4.4530920	-2.0660590	-0.0163430	H	-4.2847400	-2.1774970	0.2458080
H	5.3412120	-1.4401980	1.3835090	H	-5.2172900	-1.7497410	-1.1985050
H	6.9746460	2.3814690	4.2882620	H	-7.0354830	1.6508860	-4.5140780
H	6.8919870	2.0113860	1.9782900	H	-6.8586250	1.5595240	-2.1958490
H	6.3033300	0.8023450	4.6675720	H	-6.4170900	0.0196100	-4.7282490
H	3.3020950	3.6014190	0.2158220	H	-3.2228400	3.4357910	-0.7819410
H	4.7145880	4.6660370	0.2443790	H	-4.6510850	4.4546280	-0.9715500
H	3.6875060	5.4823130	-1.7674050	H	-3.6646300	5.6067650	0.8852330
H	2.9095890	3.9263320	-2.0720550	H	-2.8076260	4.1532550	1.4127720
H	4.7426270	3.9186230	-3.6909660	H	-4.6200610	4.3165660	3.0497460
H	6.2644120	2.1375060	-2.3774010	H	-6.0312450	2.2595620	2.0914810
H	4.5231970	1.8399070	-2.4505320	H	-4.2661110	2.0972480	2.0945260
H	6.8130360	0.2993520	2.3974870	H	-6.7741060	-0.1920490	-2.3895570
H	5.8670330	4.5772240	-2.4917540	H	-5.7900970	4.7207670	1.7848230
H	-1.0776470	-5.3258230	-1.9294800	H	1.3126230	-4.6508370	2.9720930
H	0.2225010	-4.8053880	-3.9150380	H	-0.1007200	-3.9282390	4.8047660
H	3.1716520	2.5217570	2.6669650	H	-3.1532190	1.9327350	-3.1584330
H	6.1320460	2.8855980	-0.1070530	H	-6.0618500	2.7719340	-0.2649180
H	5.1555310	-0.3615750	-1.4481820	H	-4.9726800	-0.2639190	1.4451670
H	4.3656470	0.3562480	2.4284290	H	-4.3351990	-0.1384600	-2.4989780
H	1.9487830	-1.4671620	-4.9480570	H	-1.8905580	-0.4011490	5.1226250
H	2.2827700	-3.8292300	-3.9057190	H	-2.2086460	-3.1009970	4.4786530
H	-0.4933330	-1.2200000	-2.5499410	H	0.5438290	-0.5066980	3.0077660
				H	0.7311210	-0.3625700	0.6176440
				H	-0.6641620	0.7847110	0.3896590
**III(Pf_2)$_2$**				**III(Pf_2)$_2$ (diprotonated)**			
C	1.6466790	5.8426140	0.5347780	C	1.6362380	1.6635270	0.7359570
C	1.8506540	4.9523060	-0.7026960	C	1.9639760	2.5647840	-0.4491190
C	2.4312130	3.6765030	-0.0770110	C	2.3686740	3.8485530	0.2884120
C	1.9679340	3.6373420	1.4152490	C	1.6381000	3.8162250	1.6703590
C	1.1596270	4.9215500	1.6624320	C	0.7758930	2.5245320	1.7018750
C	1.0773520	2.3779490	1.5752360	C	0.9045690	2.9495660	-1.5226710
C	1.6889830	1.4997680	0.4549730	C	0.9043990	4.5268940	-1.5879130
C	1.8332420	2.4294580	-0.7472080	C	1.8591820	5.0763580	-0.4747420
C	0.6805470	4.4836820	-1.6301720	C	-0.5901720	2.4596060	-1.6455730
C	0.6265880	2.9060990	-1.6110260	C	-1.4493140	3.7543370	-1.6643770
C	-0.3327020	4.4916840	1.7683350	C	-0.5627690	5.0038210	-1.7712540
C	-0.3945960	2.9149170	1.7226480	C	-1.4655370	1.6398420	-0.6550740
C	-0.7760790	5.0206940	-1.5157430	C	-1.7805540	2.5851900	0.5024670
C	-1.6768150	3.7957530	-1.2900430	C	-2.1804730	3.8417380	-0.2844700
C	-0.8814480	2.4772900	-1.4716850	C	-1.6689390	5.0971730	0.4297390
C	-2.1381150	3.8432650	0.2040940	C	-0.7153350	4.5892420	1.5632890
C	-1.6349200	2.5431280	0.8535920	C	-0.7180870	3.0103750	1.5597590
C	-1.5649150	1.6305030	-0.3695770	C	0.7528720	5.0704890	1.7280170
C	-1.4647740	5.0617350	0.8502400	C	-1.2094420	5.9588980	-0.7594500
C	-1.1946420	5.9555470	-0.3719540	C	1.4013890	5.9843920	0.6800950
P	1.5787910	-0.3613310	0.1421160	P	-1.6503500	-0.1514160	-0.3388710
N	1.6017560	-0.8160020	1.7658630	N	-3.1857920	-0.3512150	0.0422030
P	2.3958710	-1.7971010	2.6882440	P	-4.3675090	-0.4059930	1.0954870
C	4.2249320	-1.5085030	2.6539000	C	-4.0032320	0.4417130	2.6961220
C	4.5639150	-0.0882510	3.1176840	C	-2.6024360	0.1421810	3.2386160
P	-1.6475890	-0.2356240	-0.0844530	P	1.6955780	-0.1318070	0.4535870
N	-1.6764550	-0.7289770	-1.6934810	N	3.1848240	-0.4980810	0.0103090
P	-2.6102350	-1.3876010	-2.7565720	P	4.3441110	-0.5472540	-1.0704510
C	-3.4777060	-0.1946430	-3.8902850	C	4.8095840	-2.3094790	-1.3484310
C	-4.2894200	0.8060300	-3.0629090	C	6.2314050	-2.5598760	-1.8644220
N	-3.2942720	-0.2011030	0.4106310	N	1.1719600	-0.8304740	1.7932350
P	-4.0377790	-0.6605660	1.7213020	P	1.6158800	-2.0084600	2.7746020
C	-4.1118480	-2.5048930	1.9254970	C	1.3265950	-3.6794400	2.0496740

C	-5.1925320	-3.0809670	2.8460480	C	-0.1307430	-3.8165890	1.5943620
C	-3.4435910	0.1299090	3.2958630	C	0.5158790	-1.8719980	4.2418310
C	-4.2206590	-0.2422980	4.5627910	C	0.4056900	-0.4356760	4.7637930
C	-5.8058800	-0.1361130	1.5823950	C	3.4077180	-1.9078850	3.1859860
C	-6.4680200	-0.7848980	0.3618650	C	3.9881270	-3.1218770	3.9197260
C	-1.9357460	-0.0203910	3.5186550	C	0.8398230	-2.8612400	5.3655570
N	3.1999680	-0.5242180	-0.3894010	C	3.7479030	-0.6001440	3.9094260
P	4.0040030	-0.7347790	-1.7190620	C	2.2942060	-3.9314900	0.8883100
C	5.6784540	0.0361770	-1.5323000	C	3.9429160	0.2954980	-2.6674190
C	6.4416740	-0.6025470	-0.3662670	C	5.0141980	0.0936720	-3.7459830
C	4.3429120	-2.5308170	-2.0544390	C	5.8348750	0.2898870	-0.3901520
C	3.0717500	-3.2454080	-2.5250130	C	5.6082260	1.7928790	-0.2013530
C	3.3182820	0.0543480	-3.2628840	C	2.5415420	-0.0199830	-3.2045370
C	1.8153040	-0.1771030	-3.4425900	C	6.2259020	-0.3542680	0.9461470
C	5.5407400	-2.8870400	-2.9422660	C	3.7671070	-3.0301110	-2.2101600
C	2.1847450	-3.6007060	2.2856070	N	-1.1354780	-0.9861270	-1.6032840
C	2.9195830	-3.9521930	0.9882980	P	-1.7411670	-1.8347870	-2.8114070
C	1.7156520	-1.6320390	4.4030120	C	-3.3247180	-2.6766880	-2.3717460
C	2.5386280	-2.3294640	5.4900980	C	-4.1843310	-3.1147130	-3.5630410
C	0.6947880	-3.9249670	2.1361930	C	-0.4279130	-3.0162500	-3.3235490
C	1.4287900	-0.1734380	4.7685050	C	-0.8478660	-3.9526150	-4.4606170
C	5.1328200	-2.5430010	3.3269480	C	-2.0927440	-0.7824630	-4.2817600
C	4.0826540	-0.2634600	-4.5522260	C	-0.8086760	-0.1651300	-4.8476790
C	5.5566610	1.5467230	-1.3072010	C	0.1764130	-3.7782930	-2.1411650
C	-3.9655820	-2.4245330	-2.0270210	C	-3.1160180	0.3032830	-3.9283890
C	-3.4151580	-3.7245570	-1.4309790	C	-3.1026260	-3.8309390	-1.3881050
C	-1.5519770	-2.3882240	-3.9032210	C	-5.8256130	0.4454520	0.3655790
C	-0.5575940	-3.2835100	-3.1578320	C	-5.5080490	1.9146800	0.0669180
C	-5.1896570	-2.6903570	-2.9077020	C	-4.8437260	-2.1660700	1.3517930
C	-2.3213580	-3.1591640	-4.9793830	C	-3.7412540	-2.9341120	2.0870830
C	-2.4897540	0.5387770	-4.8040690	C	-6.2748770	-0.2679400	-0.9160440
C	-5.9255970	1.3891120	1.5026440	C	-6.2263220	-2.4261250	1.9596700
C	-2.7298600	-3.0823990	2.2459600	C	-5.0734620	0.2086310	3.7692330
H	-2.3976690	2.1489520	1.5301320	H	-2.6466530	2.2049490	1.0465050
H	-1.1476720	2.0093950	-2.4233050	H	-0.6571890	1.9560600	-2.6127350
H	-2.1693150	5.5872840	1.5030880	H	-2.4953960	5.6167510	0.9225820
H	-1.0206950	5.5073390	-2.4655990	H	-0.6435440	5.4452350	-2.7685720
H	-2.6046740	1.6704810	-0.7271810	H	-2.4190620	1.6552400	-1.2126770
H	-0.5580240	6.8210860	-0.2107940	H	-0.6374060	6.8509740	-0.5247170
H	-2.1734550	6.3644090	-0.6583100	H	-2.1328080	6.3214980	-1.2298500
H	1.0759520	6.7555260	0.3889150	H	0.8317910	6.8682810	0.4109080
H	2.6529230	6.1730320	0.8272350	H	2.3256130	6.3627580	1.1361990
H	2.7279760	1.4340700	0.8167460	H	2.5880400	1.6532640	1.2987800
H	2.5930350	5.4349140	-1.3467650	H	2.6864790	5.5748940	-0.9875490
H	2.5674810	1.9957900	-1.4290820	H	2.8277100	2.1604850	-0.9776850
H	3.5245170	3.6628950	-0.1322990	H	3.4514320	3.8873070	0.4362810
H	2.8393690	3.5781250	2.0755130	H	2.3719250	3.8016960	2.4808580
H	1.4387380	5.3725580	2.6203700	H	0.8341840	5.5504800	2.7073330
H	1.3122020	1.8757970	2.5163810	H	0.8472030	2.0514350	2.6838120
H	-3.2298370	3.9079680	0.2582380	H	-3.2629350	3.8758710	-0.4349220
H	-2.5496190	3.8119050	-1.9512750	H	-2.1832110	3.7090280	-2.4743180
H	0.8810500	2.5797890	-2.6240090	H	1.3421300	2.6051340	-2.4629680
H	1.0053490	4.7701350	-2.6347160	H	1.4063600	4.7904800	-2.5218960
H	-0.6361320	4.7841890	2.7778100	H	-1.2166860	4.8902600	2.4862460
H	-0.6718430	2.5914210	2.7303550	H	-1.1563930	2.7032430	2.5125250
H	2.8041020	-3.3580370	5.2300840	H	0.9627280	-3.8876710	5.0100700
H	3.4625900	-1.7807520	5.6958330	H	1.7486010	-2.5683470	5.8983920
H	1.9642250	-2.3607580	6.4211630	H	0.0210300	-2.8584660	6.0899650
H	0.7467700	-2.1413830	4.3290070	H	-0.4596380	-2.1375670	3.8151550
H	2.3534330	0.3900180	4.9257920	H	1.3115680	-0.1246730	5.2890910
H	0.8495760	-0.1331570	5.6971240	H	-0.4258110	-0.3775490	5.4719540
H	0.8614750	0.3159290	3.9754150	H	0.2170460	0.2667470	3.9500330
H	4.3856570	-1.5378470	1.5681330	H	3.8497010	-1.8573160	2.1820290
H	4.4793440	0.0080220	4.2055050	H	3.4184960	-0.6269390	4.9518090
H	5.5976390	0.1556340	2.8486150	H	4.8326890	-0.4604380	3.9141420
H	3.9054830	0.6487460	2.6501720	H	3.2946840	0.2687550	3.4240260
H	6.1675740	-2.3725120	3.0101120	H	5.0755660	-3.0144630	3.9682170
H	5.1084040	-2.4591450	4.4161280	H	3.6188850	-3.1924710	4.9450190
H	4.8764220	-3.5729790	3.0669100	H	3.7739640	-4.0674290	3.4165510
H	2.6023650	-4.1881670	3.1125330	H	1.5209300	-4.4113910	2.8416740
H	4.0047810	-3.8577610	1.0771710	H	3.3336700	-4.0037210	1.2174450
H	2.6965190	-4.9830590	0.6951370	H	2.0360660	-4.8693480	0.3893010
H	2.5931740	-3.2893830	0.1827980	H	2.2324980	-3.1299730	0.1483590
H	0.1284310	-3.7557960	3.0560460	H	-0.8290730	-3.7981650	2.4342600
H	0.2532330	-3.3035750	1.3503880	H	-0.3988080	-3.0138260	0.8987420
H	4.5628540	-2.8827060	-1.0390750	H	4.7383200	-2.7100560	-0.3298800
H	1.4403380	0.4679020	-4.2449890	H	2.3243570	0.6556730	-4.0370860
H	1.5913420	-1.2117250	-3.7171910	H	2.4695810	-1.0419580	-3.5830780
H	1.2596570	0.0514600	-2.5318350	H	1.7581390	0.1196230	-2.4554980
H	3.7395010	0.4043450	-5.3489090	H	4.8099740	0.7712970	-4.5792570
H	5.3933990	-2.5764750	-3.9786050	H	6.3862390	-2.1470390	-2.8634100
H	5.6734960	-3.9741540	-2.9427780	H	6.3941940	-3.6396690	-1.9260870
H	6.4743810	-2.4443340	-2.5872790	H	6.9975680	-2.1502690	-1.2029280
H	2.1910930	-2.9216220	-1.9631450	H	2.7469280	-2.7529010	-1.9365700
H	3.1762790	-4.3271300	-2.3940860	H	3.8656500	-4.1111580	-2.0784140
H	6.2287970	-0.1466830	-2.4627370	H	6.6394850	0.1371500	-1.1181720
H	4.8991910	1.7446050	-0.4538540	H	4.7679870	1.9714860	0.4771840

H	6.5415300	1.9719300	-1.0897460	H	6.4986870	2.2379040	0.2505400
H	5.1552870	2.0724490	-2.1778320	H	5.4189520	2.3192110	-1.1401010
H	7.4492780	-0.1797370	-0.3052880	H	7.1472340	0.1035150	1.3155300
H	6.5396930	-1.6876160	-0.4610400	H	6.3948240	-1.4318660	0.8726440
H	5.9264150	-0.3923870	0.5743980	H	5.4393790	-0.1809950	1.6852740
H	5.1633780	-0.1300250	-4.4527500	H	6.0228060	0.3106760	-3.3848490
H	3.8922620	-1.2881850	-4.8842290	H	4.9982820	-0.9253310	-4.1410570
H	3.4504190	1.1227890	-3.0564100	H	3.9519270	1.3559020	-2.3930690
H	2.8829730	-3.0571860	-3.5866530	H	3.9085950	-2.8103050	-3.2721130
H	0.5657660	-4.9755120	1.8568360	H	-0.2653450	-4.7694040	1.0749240
H	0.2447110	-3.5895240	-3.8376860	H	1.0823040	-4.2934780	-2.4722110
H	-0.1134470	-2.7459780	-2.3167220	H	0.4421300	-3.0943780	-1.3324860
H	-1.0311800	-4.1914380	-2.7759970	H	-0.5029150	-4.5336800	-1.7423720
H	-0.9586190	-1.6075400	-4.3955780	H	0.3512050	-2.3408610	-3.6984590
H	-1.6185450	-3.5929600	-5.6977260	H	0.0305540	-4.4944070	-4.8212590
H	-3.0148030	-2.5237590	-5.5381120	H	-1.2783050	-3.4184610	-5.3120590
H	-2.8932590	-3.9846340	-4.5442140	H	-1.5726470	-4.6961130	-4.1170630
H	-4.1639790	-0.7775890	-4.5152970	H	-2.5291330	-1.4449690	-5.0375140
H	-4.8819290	1.4469250	-3.7233670	H	-3.3909150	0.8539570	-4.8314620
H	-4.9700880	0.3145390	-2.3622430	H	-4.0332800	-0.1021720	-3.4933210
H	-3.6200470	1.4404180	-2.4747470	H	-2.6921930	1.0198360	-3.2197160
H	-2.9976400	1.3626530	-5.3156350	H	-1.0657550	0.5643770	-5.6204110
H	-1.6627190	0.9637320	-4.2252110	H	-0.2460950	0.3559730	-4.0669270
H	-2.0696570	-0.1162080	-5.5720910	H	-0.1542460	-0.9119180	-5.3038320
H	-4.2661660	-1.7801900	-1.1925170	H	-3.8518730	-1.8804340	-1.8329740
H	-2.5337450	-3.5452780	-0.8078790	H	-2.4608410	-3.5429400	-0.5511370
H	-4.1794690	-4.1991390	-0.8058120	H	-4.0662040	-4.1503540	-0.9801950
H	-3.1472670	-4.4432640	-2.2114170	H	-2.6611380	-4.6990610	-1.8844670
H	-5.9114750	-3.2936910	-2.3464740	H	-5.1017130	-3.5719920	-3.1810690
H	-5.6972100	-1.7679740	-3.2009960	H	-4.4788110	-2.2767840	-4.1985310
H	-3.6278900	1.1910290	3.0856180	H	-4.0299560	1.5035980	2.4264860
H	-6.4228450	-1.8777070	0.3835150	H	-6.5522460	-1.3123510	-0.7533640
H	-7.5221820	-0.4955710	0.3129570	H	-7.1456430	0.2458660	-1.3313050
H	-5.9775450	-0.4440950	-0.5540120	H	-5.4772490	-0.2348500	-1.6641860
H	-5.3340510	1.7699390	0.6644710	H	-4.6745140	1.9876230	-0.6391100
H	-1.6711810	-1.0293960	3.8466730	H	-2.5035170	-0.8971560	3.5607500
H	-1.6138860	0.6743500	4.3015780	H	-2.4171800	0.7777400	4.1090160
H	-1.3612040	0.1943980	2.6150780	H	-1.8133830	0.3476820	2.5119680
H	-5.3046920	-0.1738370	4.4381590	H	-6.0900260	0.3626190	3.3982480
H	-3.9377480	0.4367880	5.3735220	H	-4.9112640	0.9126170	4.5896970
H	-4.3573500	-2.8039690	0.9001010	H	-4.8596610	-2.5209810	0.3145200
H	-2.4927590	-2.9730320	3.3089360	H	-3.7674700	-2.7363000	3.1626180
H	-2.7056250	-4.1516750	2.0111860	H	-3.8819810	-4.0089560	1.9435000
H	-1.9430730	-2.5884020	1.6677840	H	-2.7483150	-2.6740170	1.7112150
H	-5.0218760	-2.8234680	3.8936380	H	-6.2699750	-2.1505020	3.0146520
H	-5.1798000	-4.1735500	2.7718000	H	-6.4418160	-3.4961440	1.8902350
H	-6.1970970	-2.7466000	2.5747570	H	-7.0251200	-1.8961540	1.4357170
H	-6.9708320	1.6711740	1.3419600	H	-6.3795270	2.3873160	-0.3936810
H	-5.5874840	1.8832060	2.4175120	H	-5.2580490	2.4887840	0.9626250
H	-6.3168240	-0.4850280	2.4869520	H	-6.6275940	0.3940380	1.1108260
H	-3.9763060	-1.2566700	4.8907760	H	-5.0039250	-0.8000700	4.1847460
H	-4.9373000	-3.2428270	-3.8174840	H	-3.6811070	-3.8575380	-4.1868080
				H	-0.8592970	-0.5198450	0.7579170
				H	0.8427580	-0.4628080	-0.6087650

**III(Pg$_2$)$_2$**				**III(Pg$_2$)$_2$ (diprotonated)**			
C	1.3381220	1.7502790	-0.0406640	C	1.2723420	1.8495840	0.0174440
C	1.7086140	2.2740730	-1.4289510	C	1.5142850	2.4430590	-1.3728880
C	2.0935780	3.7243440	-1.1007940	C	1.8337460	3.8930220	-0.9717140
C	1.3194370	4.0976580	0.2041060	C	1.1103010	4.1488030	0.3928680
C	0.4625030	2.8638990	0.5932200	C	0.3404680	2.8503460	0.7576320
C	0.6875650	2.3263640	-2.6063110	C	0.4181990	2.5021160	-2.4852760
C	0.6788790	3.8143700	-3.1306090	C	0.3123540	4.0181620	-2.9176710
C	1.6055060	4.6724660	-2.2055580	C	1.2406010	4.8710710	-1.9912360
C	-0.7982740	1.8031820	-2.6123040	C	-1.0447270	1.9133290	-2.4709420
C	-1.6692340	3.0164220	-3.0323710	C	-1.9897340	3.1083540	-2.7616850
C	-0.7898710	4.1982210	-3.4752470	C	-1.1864880	4.3523420	-3.1684530
C	-1.6469060	1.2826100	-1.4269760	C	-1.8335170	1.2946970	-1.2915470
C	-2.0365520	2.5196360	-0.6303780	C	-2.2188270	2.4481730	-0.3751930
C	-2.4419800	3.4832750	-1.7552140	C	-2.7183720	3.4587240	-1.4206690
C	-1.9676070	4.9074010	-1.4302170	C	-2.2913630	4.8780800	-1.0277330
C	-1.0356440	4.7776050	-0.1784670	C	-1.2915130	4.7150990	0.1647430
C	-1.0262600	3.2698020	0.2843060	C	-1.1847840	3.1826870	0.5302790
C	0.4285290	5.3042450	-0.1351960	C	0.1417230	5.3185270	0.1701110
C	-1.4781310	5.3951670	-2.8042670	C	-1.8952980	5.4702440	-2.3909020
C	1.1067970	5.8760670	-1.3880770	C	0.7264880	5.9961850	-1.0770280
P	-1.4738280	-0.2939970	-0.4499460	P	-1.6542540	-0.3421220	-0.5583090
N	-3.1168710	-0.4278730	-0.0077280	N	-3.0514230	-0.7961290	0.0605510
P	-4.0090730	-0.3902200	1.2554500	P	-4.0485120	-0.7573980	1.2712020
N	-3.2426840	-0.6596490	2.7405950	N	-3.2799410	-1.0171910	2.7404090
C	-4.0581550	-0.7839760	3.9429090	C	-4.1161830	-1.1687790	3.9340670
P	1.4038290	-0.1318650	0.1161530	P	1.6145140	0.0694020	0.2537300
N	3.1232790	-0.1528200	0.0328080	N	3.1907840	-0.0104870	-0.0073780
P	4.0642630	-1.1371950	-0.7125210	P	4.2761720	-0.8992130	-0.7226780
N	3.6743710	-2.7632300	-0.5158420	N	3.9101740	-2.5222530	-0.6681620
C	3.3187640	-3.2368630	0.8134730	C	3.4618980	-3.1311830	0.5795670
N	-1.3371220	-1.3177120	-1.7942160	N	-1.0736120	-1.2866190	-1.7119420

P	-1.6016470	-2.8143030	-2.0845970
N	-3.0584170	-3.1081350	-2.8555820
C	-4.1486670	-2.1535710	-2.7506730
N	-0.5068800	-3.4833510	-3.1991120
C	-0.3475630	-2.7042490	-4.4235610
N	-1.5557090	-3.8085370	-0.7268920
C	-0.5501010	-3.6020810	0.3048830
C	0.7701710	-3.9356560	-2.6573050
C	-2.1381760	-5.1374100	-0.7253420
C	-3.3106200	-4.2020170	-3.7752490
N	-5.2728420	-1.4956260	1.0260130
C	-6.4784860	-1.4964470	1.8405380
N	-4.8745340	1.0213600	1.5863310
C	-5.7531650	1.5091480	0.5305290
C	-4.8941460	-2.8335740	0.5896360
C	-4.2802650	2.1024830	2.3622320
C	-2.0924320	-1.5546270	2.7663740
N	1.0900390	-0.4350530	1.7420730
P	1.6206440	-0.0161050	3.1352520
N	3.2049300	0.5029240	3.4058490
C	4.2650260	-0.4937770	3.2872700
N	0.7636840	1.2887720	3.7866290
C	-0.6562330	1.3727150	3.4793370
N	1.4928710	-1.3567910	4.1417900
C	1.8652190	-1.3208690	5.5424650
C	1.0994110	1.9038490	5.0607000
C	1.0712800	-2.6572950	3.6636670
C	3.6207000	1.7928940	2.8645920
N	5.6280230	-0.9298360	-0.1431000
C	6.5773860	-1.9966570	0.0912650
N	4.1803340	-0.8980670	-2.3751380
C	2.9656560	-0.8192270	-3.1673320
C	6.1751960	0.4087790	-0.0252970
C	5.3902920	-1.0807850	-3.1531020
C	4.0429760	-3.8092710	-1.4516400
H	-2.9051910	2.2633460	-0.0206660
H	-0.8393630	1.0270450	-3.3808500
H	-2.8145410	5.5337860	-1.1317210
H	-0.8463600	4.3322200	-4.5604580
H	-2.5831160	1.0165260	-1.9446780
H	-0.9227290	6.3287880	-2.8196590
H	-2.3883270	5.5886310	-3.3884400
H	0.5426810	6.6314580	-1.9281650
H	2.0133480	6.3871850	-1.0358200
H	2.2689040	1.9135560	0.5177190
H	2.4461370	5.0080630	-2.8216040
H	2.5848260	1.7213200	-1.7801110
H	3.1724340	3.8152080	-0.9374630
H	2.0293380	4.3367900	1.0029280
H	0.4816810	6.0560370	0.6591090
H	0.5171740	2.7162500	1.6728370
H	-3.5218670	3.4481070	-1.9337040
H	-2.3789530	2.7301030	-3.8148990
H	1.1580650	1.7283740	-3.3925110
H	1.1983910	3.8065840	-4.0932540
H	-1.5585590	5.3386640	0.6016880
H	-1.4951780	3.2486160	1.2731220
H	0.6124050	-4.7224430	-1.9167520
H	1.3558320	-3.1283080	-2.1929690
H	1.3665660	-4.3615990	-3.4701500
H	0.2469120	-1.7928900	-4.2631420
H	0.1477590	-3.3177810	-5.1832930
H	-1.3276510	-2.4100430	-4.8065800
H	-2.4625300	-5.3861210	0.2916660
H	-1.4280510	-5.9120110	-1.0537340
H	-3.0154190	-5.1680740	-1.3720630
H	0.2832950	-4.3147260	0.2145210
H	-0.1476440	-2.5862700	0.2605800
H	-1.0048010	-3.7474400	1.2911480
H	-3.5181890	-3.8193260	-4.7848490
H	-2.4424280	-4.8578780	-3.8305460
H	-4.1821650	-4.7885770	-3.4537680
H	-5.0701900	-2.6620090	-2.4382150
H	-3.9107510	-1.3874830	-2.0092880
H	-4.3387240	-1.6755110	-3.7227940
H	-4.7217700	-3.5062600	1.4449180
H	-5.6975030	-3.2660370	-0.0189000
H	-3.9851450	-2.7926760	-0.0117480
H	-7.3230570	-1.8501190	1.2367850
H	-6.3914100	-2.1593790	2.7141620
H	-6.7018840	-0.4891620	2.1914250
H	-3.8191160	2.8639960	1.7172820
H	-3.5139770	1.7133340	3.0319540
H	-5.0571020	2.5939070	2.9597250
H	-6.5585020	2.1053410	0.9734250
H	-5.2139270	2.1391740	-0.1927750
H	-6.1997930	0.6709840	-0.0064550

P	-1.3400150	-2.7033160	-2.3565300
N	-2.6607540	-2.7703180	-3.3468030
C	-3.8948220	-2.0671220	-3.0200850
N	-0.0803970	-3.1000870	-3.3850310
C	0.1924950	-2.1231880	-4.4415960
N	-1.4757990	-3.8658580	-1.1762490
C	-0.6886350	-3.8211190	0.0483720
C	1.1349650	-3.6108290	-2.7544550
C	-2.0624040	-5.1695480	-1.4580820
C	-2.7690120	-3.6422800	-4.5111940
N	-5.2220140	-1.8868680	0.9102280
C	-6.4598020	-1.9776120	1.6776770
N	-4.9073740	0.6357330	1.5363210
C	-5.8417950	1.0918970	0.5108430
C	-4.8245900	-3.1572620	0.3075710
C	-4.4889680	1.6853440	2.4602100
C	-2.1162680	-1.8968050	2.7876690
N	1.0926490	-0.4784630	1.6754590
P	1.5761670	-0.2100220	3.1634160
N	3.1396140	0.2912100	3.4319820
C	4.2269910	-0.6831270	3.3805620
N	0.6900250	1.0223960	3.8558500
C	-0.7402540	1.0971680	3.5826900
N	1.3913630	-1.6470690	3.9654090
C	1.5880300	-1.7232940	5.4073520
C	1.0515560	1.6488290	5.1254140
C	1.1218150	-2.9238590	3.3241300
C	3.5611060	1.6294480	3.0183530
N	5.7301030	-0.6301270	0.0209990
C	6.7111470	-1.6609640	0.3180080
N	4.4571920	-0.4736480	-2.3171720
C	3.2944290	-0.4012930	-3.1888160
C	6.1876060	0.7350410	0.2463030
C	5.7336990	-0.4885650	-3.0182460
C	4.4204950	-3.4731600	-1.6490530
H	-3.0422280	2.1228870	0.2633850
H	-1.0864430	1.1890700	-3.2872270
H	-3.1481540	5.4423680	-0.6488130
H	-1.3029460	4.5478100	-4.2382110
H	-2.7891760	1.0524650	-1.7917560
H	-1.3882680	6.4298440	-2.3752440
H	-2.8418560	5.6509310	-2.9170730
H	0.1016610	6.7532530	-1.5401530
H	1.6241550	6.5296860	-0.7377570
H	2.2211340	2.1021580	0.5208740
H	2.0296000	5.2845730	-2.6257280
H	2.4009610	1.9730290	-1.8071760
H	2.9125080	4.0295190	-0.8549880
H	1.8475890	4.3785640	1.1676730
H	0.1967040	6.0229220	1.0049400
H	0.4505610	2.6485530	1.8249710
H	-3.8017070	3.3803580	-1.5500730
H	-2.7241140	2.8346060	-3.5240510
H	0.8755490	1.9728310	-3.3250460
H	0.7827050	4.0854480	-3.9016580
H	-1.8000440	5.1912950	1.0065580
H	-1.6022750	3.0787650	1.5346230
H	0.9068770	-4.4757150	-2.1292210
H	1.6495710	-2.8535970	-2.1432390
H	1.8208580	-3.9393900	-3.5396040
H	0.6729010	-1.2177760	-4.0487300
H	0.8481190	-2.5803490	-5.1872640
H	-0.7366000	-1.8311440	-4.9338540
H	-2.6044370	-5.5142570	-0.5717790
H	-1.2999550	-5.9168390	-1.7127880
H	-2.7749150	-5.0963800	-2.2795440
H	0.1014990	-4.5830270	0.0458020
H	-0.2182090	-2.8431980	0.1683120
H	-1.3384700	-4.0054590	0.9097710
H	-2.9899720	-3.0456200	-5.4038100
H	-1.8360150	-4.1812200	-4.6697940
H	-3.5809630	-4.3668990	-4.3736530
H	-4.7073920	-2.7837750	-2.8506420
H	-3.7833620	-1.4686240	-2.1155690
H	-4.1791100	-1.4093040	-3.8491010
H	-4.6962700	-3.9366980	1.0710330
H	-5.6014660	-3.4861930	-0.3906420
H	-3.8883460	-3.0418750	-0.2394440
H	-7.2718720	-2.2824680	1.0096930
H	-6.3844120	-2.7160290	2.4864650
H	-6.7159960	-1.0097470	2.1089640
H	-4.2108630	2.5943960	1.9131690
H	-3.6313910	1.3609350	3.0478940
H	-5.3116610	1.9353060	3.1389530
H	-6.7395680	1.4977790	0.9875140
H	-5.3909100	1.8766890	-0.1107170
H	-6.1364740	0.2620690	-0.1319830

H	-2.3927950	-2.6141280	2.7302440	H	-2.3988150	-2.9584610	2.7644270
H	-1.4053040	-1.3311690	1.9481660	H	-1.4302690	-1.6897560	1.9688330
H	-1.5431920	-1.3942130	3.7003870	H	-1.5739540	-1.7104450	3.7198510
H	-4.9276250	-0.1272100	3.8926140	H	-5.0093840	-0.5473010	3.8680140
H	-3.4597930	-0.4948170	4.8147970	H	-3.5446080	-0.8515560	4.8116160
H	-4.4087320	-1.8152660	4.0983450	H	-4.4256110	-2.2120020	4.0787290
H	2.1743270	1.8549150	5.2371330	H	2.1169930	1.5355630	5.3224560
H	0.5800920	1.4244930	5.9045830	H	0.4873770	1.2137060	5.9602600
H	0.8023580	2.9591780	5.0406460	H	0.8205960	2.7176560	5.0729890
H	-0.8537660	0.9347250	2.5000260	H	-0.9627320	0.7054040	2.5906140
H	-1.2731280	0.8532690	4.2289290	H	-1.3265760	0.5373140	4.3233130
H	-0.9636970	2.4260060	3.4640770	H	-1.0560710	2.1449600	3.6202760
H	0.2253520	-3.0329880	4.2555920	H	0.2602860	-3.4059010	3.8001840
H	1.8895400	-3.3896530	3.7400550	H	1.9849130	-3.5965060	3.4126180
H	0.7641420	-2.5691880	2.6221850	H	0.8980040	-2.7670700	2.2707140
H	2.3375450	-0.3713060	5.7939050	H	1.9424340	-0.7727370	5.8059190
H	0.9934610	-1.4635240	6.1955990	H	0.6528120	-1.9894820	5.9125500
H	2.5880570	-2.1180040	5.7655820	H	2.3417070	-2.4830130	5.6444970
H	3.9778440	1.6847800	1.8319460	H	3.9997330	1.6037670	2.0135250
H	2.7853100	2.4953300	2.8754380	H	2.7133660	2.3166000	3.0190090
H	4.4292960	2.2024370	3.4819360	H	4.3096580	2.0061920	3.7222790
H	5.1335630	-0.1632360	3.8690530	H	5.0230350	-0.3637980	4.0600240
H	3.9299020	-1.4517760	3.6894910	H	3.8765160	-1.6648330	3.7018200
H	4.5656160	-0.6328050	2.2401450	H	4.6384940	-0.7632730	2.3661140
H	5.3760010	1.1415090	-0.1411650	H	5.3936550	1.4432780	0.0063000
H	6.6216620	0.5478300	0.9679430	H	6.4607430	0.8672600	1.2995420
H	6.9518170	0.5986380	-0.7807290	H	7.0633760	0.9636190	-0.3735900
H	7.0478040	-1.8659710	1.0742420	H	7.0533850	-1.5504080	1.3528850
H	6.0803190	-2.9657930	0.0852370	H	6.2758390	-2.6534620	0.2120900
H	7.3777440	-2.0162420	-0.6638630	H	7.5839570	-1.5911970	-0.3437080
H	5.5082750	-0.2488950	-3.8585370	H	5.8473930	0.4374200	-3.5914280
H	5.3742370	-2.0133410	-3.7373080	H	5.8015870	-1.3346800	-3.7142350
H	6.2636290	-1.1004040	-2.5011130	H	6.5581770	-0.5582660	-2.3094830
H	2.7892060	-1.7452520	-3.7352760	H	3.2134910	-1.2896780	-3.8292170
H	2.0999200	-0.6420900	-2.5228240	H	2.3773290	-0.3176200	-2.6023710
H	3.0341750	0.0098290	-3.8834640	H	3.3644230	0.4832040	-3.8309570
H	3.2478200	-4.5629550	-1.4835360	H	3.6744050	-4.2581810	-1.8030610
H	4.1578710	-3.4000390	-2.4556110	H	4.5969550	-2.9802940	-2.6048970
H	4.9762290	-4.3230220	-1.1763650	H	5.3521400	-3.9520170	-1.3219810
H	2.5385160	-4.0027080	0.7326810	H	2.6740420	-3.8605560	0.3646840
H	2.9211270	-2.4093390	1.4027840	H	3.0491340	-2.3698940	1.2408880
H	4.1764890	-3.6798450	1.3424440	H	4.2791360	-3.6462260	1.1009190
				H	0.9537690	-0.7050730	-0.7094020
				H	-0.7494470	-0.2663410	0.5039370
**III(Ph$_2$)$_2$**				**III(Ph$_2$)$_2$ (diprotonated)**			
C	-3.9094920	-2.0284370	-3.8897570	C	4.6417070	-3.3707000	-1.5114570
N	-2.8433620	-1.4579560	-3.0651380	N	5.1784690	-2.1331860	-0.9323870
C	-2.0774290	-0.5440920	-3.9068610	C	5.7430860	-2.4805730	0.3884840
C	-2.0395780	-1.2401300	-5.2706890	C	5.0574520	-3.8054230	0.8134000
C	-3.3944530	-1.9722950	-5.3510130	C	4.0599660	-4.1225500	-0.3135570
P	-2.1426790	-2.1839680	-1.7231010	P	4.3366520	-0.7009830	-1.0567660
N	-3.5084250	-2.8826770	-1.0415160	N	5.5430100	0.4198690	-1.0493180
C	-3.4731440	-4.1605680	-0.3184200	C	6.9715620	0.1956300	-1.3288510
C	-4.8579260	-4.2500280	0.3284390	C	7.5284270	1.6168350	-1.4912680
C	-5.2020070	-2.7846530	0.6035240	C	6.5775160	2.4814760	-0.6565260
C	-4.6675540	-2.0716010	-0.6378420	C	5.2248320	1.8439490	-0.9557870
N	-1.1832420	-1.2180650	-0.9740300	N	3.3233200	-0.4822690	0.1247830
P	-1.2910560	-0.0295060	0.2220490	P	1.9522430	0.1266560	0.6648130
N	-2.9011610	0.5736760	0.1395000	N	1.8983050	1.6989740	0.9270160
P	-3.4810700	1.9471310	-0.3095040	P	1.5079670	3.1695850	0.5535660
N	-2.6628860	2.7752230	-1.5038700	N	-0.0913040	3.4327820	0.9347010
C	-3.1832710	3.8888750	-2.2943160	C	-0.7466430	2.8898310	2.1340530
C	-2.4802640	3.7074670	-3.6378210	C	-1.3445130	4.1077460	2.8793600
C	-1.0770830	3.2588790	-3.2162250	C	-1.0706090	5.3215790	1.9709400
C	-1.2959920	2.4359330	-1.9324120	C	-0.7606040	4.6988170	0.6085610
C	-1.5817840	-1.1303570	1.7060200	C	1.7628390	-0.6576540	2.2891240
C	-1.7148480	-0.4720580	3.0764920	C	1.0291270	-0.1542680	3.5559230
C	-2.3696300	-1.6148960	3.8719670	C	1.7906860	-1.0134500	4.5998130
C	-1.9419220	-2.9432120	3.1629160	C	2.2772780	-2.3085930	3.8652370
C	-1.0175100	-2.5402270	1.9891300	C	1.8222690	-2.1793530	2.4008530
C	-1.8100870	-1.6645470	5.3005710	C	0.8507550	-1.4668640	5.7257430
C	-0.5984050	-0.6743310	5.3241380	C	-0.5911240	-1.0835790	5.2823310
C	-0.4923650	0.0270800	3.9130320	C	-0.4958930	-0.2685790	3.9328870
C	1.0301100	0.0472800	3.5012830	C	-1.6907490	-0.7435880	3.0454810
C	1.7719480	-0.7541680	4.6023980	C	-2.3030040	-1.9775450	3.7357950
C	0.8351660	-1.0812720	5.7757340	C	-1.7609170	-2.1140150	5.1633480
C	2.1906390	-2.1186260	3.9617720	C	-1.8086400	-3.2299720	2.9349270
C	1.7098410	-2.0740290	2.5019470	C	-0.8856530	-2.7099060	1.8049950
C	1.7129280	-0.5664720	2.2538990	C	-1.5250920	-1.3112140	1.6354190
C	1.1941580	-2.5415230	6.0868400	C	-1.5575590	-3.6337100	5.2774190
C	1.4616850	-3.2258380	4.7358870	C	-1.0258490	-4.1105690	3.9182780
C	0.3224460	-3.7328330	3.7914370	C	0.4769180	-3.9737380	3.5365890
C	0.4343590	-2.9656680	2.4163490	C	0.5782780	-3.1024780	2.2234720
C	-1.1792820	-3.7791620	4.2026620	C	1.5810800	-3.4984370	4.5362590
C	-1.6661530	-3.1787180	5.5293770	C	1.2660080	-2.9308290	5.9297730
P	1.7999130	0.0489850	0.4765880	P	-1.4682800	-0.2132240	0.1887050

N	2.3407750	1.6250020	0.8710510
P	2.0638890	3.0631430	0.3498180
N	2.3208130	3.3019690	-1.2822770
C	2.2290650	2.1957670	-2.2417810
C	2.7523210	2.7887210	-3.5661090
C	3.4395050	4.0983220	-3.1618460
C	2.5959710	4.5570870	-1.9750260
N	3.2364290	-0.8213540	0.1628470
P	4.0706870	-1.2149430	-1.0851080
N	5.4578430	-0.3048180	-1.2563560
C	6.7862340	-0.7519880	-1.6862590
C	7.6041160	0.5495670	-1.7596920
C	6.8622290	1.5072100	-0.8198510
C	5.4065320	1.1439440	-1.0922000
N	4.7432090	-2.7687540	-1.0268120
C	5.3854840	-3.1717670	0.2355070
C	4.4745780	-4.2558440	0.8732660
C	3.3377890	-4.4681570	-0.1433200
C	3.9224240	-3.9089760	-1.4443170
N	3.3021340	-1.1129060	-2.5655690
C	4.0634750	-1.0240160	-3.8178010
C	2.9982860	-1.1360910	-4.9188930
C	1.6979980	-0.7203100	-4.2237180
C	1.8688550	-1.2911970	-2.8185050
N	-1.2313070	-3.5005330	-2.2118020
C	-0.1186600	-4.0357100	-1.4248490
C	0.4930940	-5.0683770	-2.3670160
C	-0.7432900	-5.6615660	-3.0489550
C	-1.6624420	-4.4471480	-3.2450480
N	0.5328000	3.6300290	0.7159570
C	-0.2697380	3.1605570	1.8466000
C	-0.7955490	4.4400780	2.5394730
C	-0.3566030	5.6109880	1.6373360
C	0.0270080	4.9369410	0.3149380
N	3.0957590	4.2389000	1.0073100
C	3.0105930	4.4601600	2.4565760
C	4.1124600	3.5823020	3.0856380
C	5.1333480	3.3649560	1.9444210
C	4.5353230	4.1242830	0.7425410
N	-3.6960320	2.9917130	0.9906940
C	-4.2255470	4.3502570	0.8055120
C	-4.7251010	4.7648850	2.1961090
C	-5.0668690	3.4311530	2.8659160
C	-3.9718820	2.4996590	2.3463860
N	-5.0195990	1.8448230	-0.9879060
C	-5.1674670	1.1683760	-2.2865600
C	-6.6715780	0.9261460	-2.3957750
C	-7.0808000	0.6398890	-0.9488740
C	-6.1809280	1.5695480	-0.1233010
H	1.3420750	1.0950960	3.5426650
H	2.4590300	-2.5104120	1.8400410
H	1.0871940	-0.4716930	6.6496810
H	2.1309500	-4.0741700	4.9128990
H	2.7621430	-0.3070080	2.4692140
H	0.5270700	-3.0589210	6.7706400
H	2.1663250	-2.5105310	6.5978710
H	-1.1215080	-3.4863690	6.4176190
H	-2.6894940	-3.5524210	5.6722430
H	-2.6263660	-1.3836610	1.4622130
H	-1.4972600	-4.8260790	4.1614030
H	-1.2424260	-3.1395870	1.1066220
H	-2.8273760	-3.4721700	2.7925110
H	-3.4607120	-1.5201060	3.8668540
H	-2.5472690	-1.2835550	6.0148260
H	-2.4045320	0.3694180	2.9859810
H	2.6606350	-0.2104030	4.9387320
H	3.2779580	-2.2398120	4.0106680
H	0.6929120	-3.7218980	1.6672400
H	0.5875820	-4.7744370	3.5875470
H	-0.9028960	0.1020270	6.0320890
H	-0.7109950	1.0861470	4.0829140
H	-1.0921120	-0.3626260	-3.4750240
H	-2.6001120	0.4218870	-3.9759450
H	-1.8814980	-0.5425860	-6.0970190
H	-1.2177750	-1.9640400	-5.2817600
H	-4.1011860	-1.4261900	-5.9813820
H	-3.2846040	-2.9731400	-5.7763020
H	-4.1582630	-3.0362980	-3.5549590
H	-4.8185720	-1.4198530	-3.7882410
H	-1.5370630	-4.0096610	-4.2438670
H	-2.7189000	-4.7247970	-3.1402450
H	-0.5222250	-6.1707990	-3.9894730
H	-1.2208220	-6.3837820	-2.3779670
H	1.0964850	-5.8122650	-1.8418070
H	1.1296320	-4.5699840	-3.1076150
H	0.5620680	-3.2327960	-1.1365110
H	-4.3642690	-1.0409300	-0.4263120

N	-0.9984840	-1.0425580	-1.1065980
P	-1.7355820	-2.1591040	-1.9656350
N	-2.9662800	-3.0163380	-1.2619140
C	-2.9004130	-4.4431790	-0.8893380
C	-4.3259010	-4.7617680	-0.4274140
C	-4.8418480	-3.4193200	0.0943560
C	-4.2740170	-2.4314240	-0.9195650
N	-2.9111690	0.4750440	0.1728900
P	-3.7443290	1.7359750	-0.2724680
N	-2.9861100	2.6839030	-1.3889310
C	-3.6290600	3.7483460	-2.1726560
C	-2.8741850	3.7007900	-3.4985790
C	-1.4441080	3.3849950	-3.0529040
C	-1.6198450	2.4192910	-1.8736380
N	-5.1883810	1.3623290	-0.9991470
C	-6.3688040	0.9331670	-0.2227780
C	-7.0616410	-0.0981280	-1.1197700
C	-6.6362850	0.3052720	-2.5335380
C	-5.1930070	0.7739260	-2.3516720
N	-4.1132140	2.6161580	1.0849160
C	-4.8051510	3.9144370	0.9718980
C	-5.3379770	4.1761380	2.3840560
C	-5.5682550	2.7698240	2.9425900
C	-4.3796480	1.9839550	2.3916650
N	3.6136690	-0.6283740	-2.5432430
C	4.4232210	-0.4349870	-3.7627230
C	3.4535820	-0.7596680	-4.9054250
C	2.0720780	-0.4901790	-4.3034630
C	2.2262840	-0.9845430	-2.8686330
N	-0.5774530	-3.2507800	-2.4119690
C	0.4518520	-3.7606430	-1.4924680
C	1.2406340	-4.7373270	-2.3622070
C	0.1593040	-5.3302230	-3.2674900
C	-0.7249890	-4.1241860	-3.5919440
N	-2.4495860	-1.4696550	-3.2910650
C	-3.4456960	-2.1250170	-4.1472190
C	-2.8610650	-2.0766020	-5.5754800
C	-1.6854620	-1.0753380	-5.5031420
C	-1.7851850	-0.4483270	-4.1059290
N	2.3968100	4.3415960	1.3509300
C	2.2844410	4.4182500	2.8188670
C	3.4621320	3.5888540	3.3615870
C	4.5190980	3.6325020	2.2333010
C	3.8460680	4.4357660	1.1025930
N	1.7585990	3.5085040	-1.0420150
C	1.8893410	2.4480030	-2.0555160
C	2.3956770	3.1803330	-3.3117540
C	2.9283000	4.5197660	-2.7918210
C	1.9758800	4.8312110	-1.6420390
H	1.2990200	0.8981350	3.6785940
H	2.6005590	-2.5408260	1.7255880
H	1.0653500	-0.9159740	6.6458390
H	2.2807560	-4.3302470	4.6576980
H	2.7989310	-0.3790550	2.5572800
H	0.6114700	-3.5255840	6.5591630
H	2.2276270	-2.9002440	6.4587710
H	-1.0141340	-3.9857080	6.1485570
H	-2.5653780	-4.0587870	5.3727020
H	-2.5619510	-1.6130620	1.4001270
H	-1.3004390	-5.1612870	3.7891860
H	-1.0875640	-3.2541750	0.8827080
H	-2.6633400	-3.7656020	2.5102690
H	-3.3954710	-1.9263510	3.7162040
H	-2.5225510	-1.8175440	5.8900160
H	-2.4170840	0.0710440	3.0116420
H	2.6497280	-0.4600570	4.9892290
H	3.3667670	-2.3868140	3.9249210
H	0.8791320	-3.7877810	1.4267460
H	0.7862380	-4.9830960	3.2547090
H	-0.9354720	-0.3743790	6.0388290
H	-0.7600630	0.7610330	4.1856170
H	-0.8177590	-0.1754310	-3.6794840
H	-2.4143210	0.4513330	-4.1292160
H	-1.7270010	-0.3170620	-6.2875040
H	-0.7335870	-1.6034110	-5.6068500
H	-3.6241330	-1.7585670	-6.2886860
H	-2.5122570	-3.0634040	-5.8881890
H	-3.6598570	-3.1360240	-3.7967200
H	-4.3816050	-1.5543720	-4.1001500
H	-0.3723490	-3.6031270	-4.4891680
H	-1.7657670	-4.4182590	-3.7612910
H	0.5574040	-5.8016600	-4.1675330
H	-0.4175880	-6.0796430	-2.7156810
H	1.7642680	-5.4866490	-1.7664130
H	1.9805540	-4.2032800	-2.9678110
H	1.0458450	-2.9362820	-1.0881000
H	-4.1618070	-1.4218350	-0.5170030

H	-4.6628660	-2.4325160	1.4914440	H	-4.4321480	-3.2107410	1.0894010
H	-0.4640480	-4.5288710	-0.5017240	H	0.0055510	-4.3020780	-0.6474900
H	-2.6983980	-4.1727990	0.4580930	H	-2.1896120	-4.6066590	-0.0708950
H	-4.8515710	-4.8742640	1.2253390	H	-4.3407890	-5.5546490	0.3225760
H	-3.2755080	-4.9982400	-0.9955300	H	-2.5921210	-5.0675980	-1.7317850
H	-5.5774040	-4.6765740	-0.3790970	H	-4.9315880	-5.0851550	-1.2798160
H	-4.2728530	3.8448300	-2.3538240	H	-4.7019780	3.5669500	-2.2674200
H	-2.9081730	4.8595430	-1.8533620	H	-3.4912960	4.7242480	-1.6883830
H	-0.5667850	2.6774000	-3.9890810	H	-0.8347330	2.9471580	-3.8471100
H	-2.4775960	4.6180350	-4.2418410	H	-2.9527230	4.6348490	-4.0579290
H	-0.4543290	4.1330860	-2.9964750	H	-0.9490290	4.3010710	-2.7148440
H	-2.9793540	2.9164420	-4.2086890	H	-3.2669430	2.8911520	-4.1233300
H	-0.5575560	2.6902680	-1.1686650	H	-0.8823400	2.6060180	-1.0918820
H	-1.2220460	1.3612890	-2.1185600	H	-1.5309480	1.3784490	-2.1958050
H	-3.4467520	5.0267800	0.4352830	H	-4.1116800	4.6935010	0.6405080
H	-5.0467360	4.3539960	0.0756790	H	-5.6278180	3.8590900	0.2469980
H	-5.5735850	5.4502640	2.1385420	H	-6.2409780	4.7890020	2.3705520
H	-3.9268980	5.2700060	2.7503440	H	-4.5844980	4.7017590	2.9797120
H	-6.0464930	3.0791330	2.5249930	H	-6.5030920	2.3567350	2.5494050
H	-5.0853740	3.4896310	3.9567250	H	-5.6127390	2.7413850	4.0330110
H	-4.2686000	1.4485690	2.3327880	H	-4.5787840	0.9147340	2.2901770
H	-3.0702070	2.5697970	2.9720180	H	-3.5048730	2.0894540	3.0465030
H	-4.7911010	1.8005460	-3.0968750	H	-4.9047300	1.5163740	-3.1004340
H	-6.6946490	2.5075990	0.1245420	H	-7.0181960	1.7944490	-0.0266760
H	-5.8913280	1.0923160	0.8190170	H	-6.0900720	0.4947780	0.7396550
H	-4.6067060	0.2248950	-2.3106710	H	-4.4839100	-0.0610610	-2.4187850
H	-6.8643520	-0.4032740	-0.7004590	H	-6.6942800	-1.1016970	-0.8862930
H	-6.9109340	0.1072860	-3.0790620	H	-6.7156660	-0.5111910	-3.2548840
H	-8.1419360	0.8157230	-0.7582270	H	-8.1444520	-0.0986540	-0.9817070
H	-7.1713070	1.8304030	-2.7596980	H	-7.2531120	1.1356460	-2.8912490
H	-6.2696270	-2.6115830	0.7623240	H	-5.9309200	-3.3709370	0.1577540
H	-5.4214640	-2.0660010	-1.4397070	H	-4.9168300	-2.3809250	-1.8094490
H	1.2031430	1.8284630	-2.3522500	H	0.9374390	1.9398910	-2.2458470
H	2.8316820	1.3497050	-1.9092460	H	2.6050000	1.6930840	-1.7232390
H	3.4243920	2.0978300	-4.0818660	H	3.1547490	2.5970370	-3.8383090
H	1.9160320	3.0024400	-4.2399920	H	1.5682370	3.3539730	-4.0060660
H	4.4647920	3.9072390	-2.8259170	H	3.9472160	4.4080080	-2.4053720
H	3.4720520	4.8322030	-3.9706220	H	2.9341430	5.3000120	-3.5550510
H	1.6712580	5.0377540	-2.3321190	H	1.0351510	5.2480790	-2.0262530
H	3.1082790	5.2593470	-1.3149960	H	2.3820300	5.5288250	-0.9086940
H	-0.8592660	4.8113920	-0.3265550	H	-1.6852570	4.4725040	0.0622070
H	0.7737710	5.5017720	-0.2453060	H	-0.1369130	5.3330590	-0.0226920
H	-1.1402310	6.3627280	1.5087820	H	-1.9156230	6.0124370	1.9260430
H	0.5222040	6.1107300	2.0559860	H	-0.1977350	5.8789290	2.3225280
H	-0.3992500	4.5466950	3.5531190	H	-0.9028570	4.2355720	3.8704690
H	-1.8834340	4.3966260	2.6172120	H	-2.4172860	3.9634260	3.0194700
H	-1.1029890	2.5498990	1.4831590	H	-1.5519330	2.2130330	1.8308040
H	4.7129440	3.6365850	-0.2174070	H	4.0954820	4.0856230	0.1004240
H	5.2180360	2.3012400	1.7117400	H	4.7557830	2.6197700	1.8975510
H	0.3355550	2.5284520	2.4964390	H	-0.0351790	2.3168630	2.7287300
H	2.0124110	4.2339610	2.8343890	H	1.3149530	4.0694350	3.1712250
H	3.6899900	2.6255580	3.4015750	H	3.1401180	2.5592700	3.5385200
H	3.2061830	5.5215260	2.6566120	H	2.3816750	5.4705240	3.1080060
H	4.5551060	4.0644630	3.9611450	H	3.8331940	3.9943270	4.3051110
H	3.1710190	-3.6068460	-2.1745580	H	3.9219000	-3.1542180	-2.3013600
H	4.5695340	-4.6560670	-1.9236270	H	5.4667070	-3.9370060	-1.9599040
H	4.0908390	-3.9372630	1.8457130	H	4.5632290	-3.7105690	1.7833010
H	3.0350720	-5.5150490	-0.2299520	H	3.9554370	-5.1942440	-0.4964200
H	5.0382760	-5.1799240	1.0291610	H	5.8016930	-4.6008770	0.9016180
H	2.4584960	-3.8802870	0.1376030	H	3.0695670	-3.7186070	-0.0780850
H	6.3780200	-3.5853630	0.0190280	H	6.8259790	-2.6110470	0.2969390
H	5.5187700	-2.3053850	0.8866020	H	5.5594890	-1.6737430	1.1016820
H	7.2148680	-1.4471920	-0.9549170	H	7.4456050	-0.3218340	-0.4875360
H	6.7601780	-1.2678960	-2.6523640	H	7.1208680	-0.4121550	-2.2251210
H	7.5783550	0.9480000	-2.7797280	H	7.4736310	1.9195290	-2.5420680
H	8.6504430	0.3934490	-1.4873750	H	8.5699910	1.6862520	-1.1721590
H	7.0815890	2.5597980	-1.0162670	H	6.6089410	3.5399470	-0.9238640
H	7.1102970	1.2916010	0.2252780	H	6.8068640	2.3863010	0.4099830
H	5.0633590	1.6443290	-2.0135410	H	4.8267560	2.2261660	-1.9083930
H	4.7082850	1.4060910	-0.2920640	H	4.4715870	2.0083140	-0.1810240
H	4.6003610	-0.0673830	-3.8641490	H	4.7814130	0.6002960	-3.8120560
H	1.2607510	-0.7975710	-2.0556090	H	1.5128090	-0.5224620	-2.1839480
H	1.5841110	-2.3537650	-2.8253450	H	2.0647480	-2.0675420	-2.8297920
H	4.8097900	-1.8239180	-3.9008260	H	5.2975260	-1.0941340	-3.7745880
H	0.8095270	-1.1117540	-4.7238170	H	1.2702590	-1.0124860	-4.8287990
H	2.9180290	-2.1757790	-5.2534070	H	3.5376050	-1.8175770	-5.1735680
H	1.6122750	0.3699840	-4.1838970	H	1.8459690	0.5809560	-4.3143360
H	3.2404470	-0.5222250	-5.7895340	H	3.6593310	-0.1664100	-5.7980760
H	6.1308480	3.7363510	2.1934240	H	5.4521410	4.1049180	2.5477400
H	4.9550000	5.1370550	0.6850580	H	4.1251560	5.4934940	1.1620060
				H	-0.5204230	0.7960880	0.3642490
				H	0.9767800	-0.2914640	-0.2413610
**I-Pa$_2$ TS1**				**I-Pa$_2$ TS2**			
C	0.2496520	3.8819880	-1.7525360	C	-1.6640470	0.6865630	-1.2043120
C	-0.1661500	4.2692560	-0.3092820	C	-2.3084080	1.7402810	-0.2821040
C	1.0237470	5.0834180	0.2003240	C	-3.1080980	2.6290740	-1.2733010

C	2.0655000	4.0192550	-0.1454750	C	-2.0781950	2.7693820	-2.4217240
C	1.7906090	3.6999670	-1.6356950	C	-1.0549520	1.6052130	-2.2672410
C	1.5264320	2.7300600	0.5252780	C	-1.0371100	3.7702070	-1.8398320
C	-0.0386560	2.8924840	0.4049060	C	-1.6050270	4.0814520	-0.4339400
C	0.0702040	2.3390870	-1.8862440	C	-3.1207810	4.0155710	-0.6278400
C	-0.7059050	1.9590640	-0.6236230	C	-1.2635870	2.7649050	0.3105170
C	1.6200530	2.1528600	-1.7596970	C	0.1642350	2.4637610	-0.1663560
C	2.0486890	1.6165820	-0.3909490	C	-0.0051040	2.6103920	-1.6833080
P	1.3642410	-0.0071370	0.0819220	P	0.8193860	0.8604880	0.3927720
N	2.1608680	-0.3870690	1.4818810	N	2.2466600	0.6005880	-0.3311440
P	1.5503340	-0.6504420	2.9301030	P	3.7085190	0.8522320	0.2617350
C	2.9095580	-1.1051860	4.0333380	C	4.0128960	2.5584950	0.7988900
P	-1.0150530	0.1298560	-0.2440800	P	-0.5454450	-0.6743090	-0.4755800
N	-1.5769560	0.1593690	1.3102930	N	-0.3080630	-1.3833430	-1.9530820
P	-3.0863330	0.4091630	1.7388560	P	0.9740990	-1.7392440	-2.8105580
C	-3.8390260	1.9468710	1.1259930	C	0.4648240	-2.8197750	-4.1718520
N	-2.2834800	0.0657300	-1.3813250	N	-1.6154590	-1.2479560	0.6460980
P	-2.3669240	-0.9338240	-2.6088790	P	-2.9318540	-2.1085290	0.5061670
C	-1.0289820	-0.7466110	-3.8292380	C	-2.9539070	-3.2786690	-0.8806160
N	1.7005650	-1.0809320	-1.1203370	C	-3.1520270	-3.0905450	2.0117920
P	3.0374350	-1.9285060	-1.3086410	C	-4.4750650	-1.1527500	0.3422450
C	2.9516540	-2.7721730	-2.9050810	N	0.9119700	0.8808090	2.0064490
C	4.5744820	-0.9582560	-1.3114970	P	0.1084530	0.3713810	3.2823320
C	3.2969590	-3.2188100	-0.0586720	C	-1.6412150	0.8376320	3.3580620
C	-2.3405860	-2.7234220	-2.2434710	C	0.1212430	-1.4203540	3.5310930
C	-3.9181720	-0.6589960	-3.5000750	C	0.8925020	1.0986990	4.7408310
C	-4.2636620	-0.8937030	1.2729300	C	4.1388870	-0.1934850	1.6763750
C	-3.1306490	0.5048280	3.5451060	C	4.9003360	0.4885060	-1.0451200
C	0.7273220	0.7766010	3.6883810	C	1.7765420	-0.3046140	-3.5842620
C	0.3431380	-2.0015290	3.0250580	C	2.3426120	-2.6174190	-1.9932590
O	-2.1624090	-3.6773020	1.2000560	O	0.1560300	-3.8858280	0.5841710
C	-1.1454290	-3.9557890	0.7065620	C	1.0582330	-3.1839720	0.8841960
O	-0.1299080	-4.2434770	0.2138250	O	2.1000920	-2.8905400	1.3634910
H	1.1669430	6.0217270	-0.3459870	H	-3.5030400	4.7976030	-1.2923290
H	0.9705970	5.3014460	1.2721860	H	-3.6766560	4.0485570	0.3151650
H	2.0980170	1.6561350	-2.6053080	H	0.9336600	2.6850060	-2.2339750
H	-1.7303450	2.3222720	-0.7635850	H	-2.4787800	0.1291010	-1.6900430
H	3.1440430	1.5685600	-0.3293630	H	0.8523710	3.2303420	0.2147640
H	-1.1550630	4.7242300	-0.2192390	H	-4.0727810	2.2075390	-1.5653710
H	-0.5230760	2.8646600	1.3838440	H	-2.9309870	1.3095250	0.5046020
H	-0.1403880	4.5260340	-2.5421780	H	-2.4975900	2.9734300	-3.4081910
H	2.4304260	4.2144530	-2.3541160	H	-0.7576900	4.6390230	-2.4374330
H	3.1071250	4.2419420	0.0964250	H	-1.2005310	4.9774980	0.0420000
H	1.8433640	2.6189950	1.5644530	H	-1.3455960	2.8563620	1.3945770
H	-0.3678090	1.9669300	-2.8130980	H	-0.7442710	1.0900820	-3.1752770
H	3.3832780	-2.0186770	3.6675100	H	-2.0949600	0.4085450	4.2551350
H	3.6473620	-0.3004660	4.0481670	H	-1.7440360	1.9238090	3.3875000
H	2.5338460	-1.2721210	5.0455830	H	-2.1311940	0.4328860	2.4692990
H	0.7933330	-2.9063260	2.6094170	H	0.8670590	2.1869890	4.6575120
H	0.0440450	-2.1847970	4.0601920	H	0.3634070	0.7903440	5.6453820
H	-0.5300060	-1.6964250	2.4393370	H	1.9316500	0.7679060	4.7973990
H	-0.0995860	1.0580930	3.0299210	H	-0.3386410	-1.6676570	4.4912610
H	0.3390510	0.5169470	4.6767140	H	-0.4543060	-1.8640830	2.7141640
H	1.4358080	1.6028070	3.7801130	H	1.1489620	-1.7859130	3.5056400
H	2.8714180	-2.0286500	-3.7007540	H	3.8139350	3.2400260	-0.0312130
H	3.8424200	-3.3836320	-3.0652890	H	3.3356770	2.7898210	1.6249530
H	2.0645730	-3.4090830	-2.9215750	H	5.0461100	2.6802860	1.1327800
H	4.5040140	-0.1707690	-2.0654070	H	3.4846960	0.0706540	2.5096770
H	4.6990790	-0.5018480	-0.3262200	H	3.9629240	-1.2379520	1.4129830
H	5.4362410	-1.5947040	-1.5267730	H	5.1839740	-0.0441430	1.9573090
H	3.4110660	-2.7302690	0.9111700	H	4.7057160	1.1384500	-1.9005360
H	2.4199830	-3.8683830	-0.0289370	H	5.9184880	0.6513140	-0.6850950
H	4.1876840	-3.8083970	-0.2890020	H	4.7866080	-0.5526270	-1.3545590
H	-0.0742090	-0.8748150	-3.3073360	H	0.0467180	-3.7421590	-3.7629910
H	-1.1136430	-1.4916980	-4.6245490	H	-0.2981790	-2.3142470	-4.7671360
H	-1.0665860	0.2540250	-4.2654090	H	1.3207710	-3.0593720	-4.8071960
H	-4.7581070	-0.8684030	-2.8338660	H	2.6897090	-2.0210440	-1.1482120
H	-3.9682040	0.3835810	-3.8201000	H	1.9985990	-3.5916230	-1.6396580
H	-3.9774930	-1.3120210	-4.3740280	H	3.1603760	-2.7641200	-2.7035810
H	-1.3378080	-3.0106750	-1.9187860	H	1.0669120	0.2041100	-4.2399560
H	-3.0595250	-2.9460310	-1.4516720	H	2.0910760	0.3716700	-2.7844460
H	-2.5966370	-3.2965650	-3.1381490	H	2.6454360	-0.6200840	-4.1677740
H	-3.1985100	2.7928870	1.3865990	H	-2.0979370	-3.9484230	-0.7824380
H	-3.9185310	1.8758690	0.0386540	H	-3.8835860	-3.8527020	-0.8807020
H	-4.8317190	2.0912930	1.5595830	H	-2.8576750	-2.7254220	-1.8171060
H	-4.3023560	-0.9372510	0.1824580	H	-2.3027360	-3.7703430	2.1078370
H	-3.9086870	-1.8523700	1.6558300	H	-3.1721970	-2.4199100	2.8741990
H	-5.2577460	-0.6795600	1.6731990	H	-4.0820690	-3.6623450	1.9744540
H	-4.1570670	0.6269760	3.8978690	H	-4.5775800	-0.4865480	1.2024070
H	-2.7091270	-0.4133760	3.9601440	H	-4.4381610	-0.5491830	-0.5677610
H	-2.5286300	1.3535730	3.8765440	H	-5.3400380	-1.8195000	0.2984590
H	-0.7114820	-1.2732700	-0.4270970	H	0.5455560	-1.6228260	0.1673660
**II-Pa_2 TS1**				**II-Pa_2 TS2**			
C	1.9454350	-1.1871470	-1.8529090	C	5.3403480	0.8809240	-0.3999550
C	2.4395810	0.2911070	-1.9391540	C	4.0126370	1.0419080	-1.1329160
C	2.1512110	1.0446040	-0.6371220	C	2.7704540	1.0563580	-0.2080820

C	3.0055690	0.2212670	0.3513000	C	2.7505660	-0.2714680	0.6481000
C	2.5355230	-1.2860580	0.4299140	C	3.9847240	-1.1267600	0.2711250
C	1.3336640	-1.5708530	-0.4963460	C	5.3271510	-0.4194220	0.4303580
C	3.9184400	-0.1830300	-1.7943410	C	1.6237260	-1.2285730	0.1980020
C	4.3611530	0.2873040	-0.3973080	C	2.0716440	-1.4806590	-1.2522800
C	3.5906750	-2.1738580	-0.2768410	C	2.1025750	-0.1751750	-2.1115860
C	3.4362970	-1.6621720	-1.7199550	C	1.6681340	1.0459200	-1.2920080
C	5.0106020	-2.0430310	0.2658580	C	3.6370200	-1.4614730	-1.1880270
C	5.4639010	-0.5699180	0.2138960	C	3.6613230	-0.1542480	-2.0343230
P	0.3601360	1.1912500	-0.3232770	P	-0.0547280	1.0104650	-0.7136100
P	-0.4115810	-1.0377020	0.0051530	N	-1.0314090	1.0209170	-2.0025830
N	-1.2488120	-1.3417940	-1.3903070	P	-1.8560570	-0.0043250	-2.9003420
P	-2.3439940	-2.4800000	-1.5542880	C	-2.4651450	0.9072260	-4.3385890
C	-1.7187340	-4.1818680	-1.4430710	P	-0.1871510	-0.7803830	0.5930300
N	-0.5382080	-2.3326420	1.1105410	N	0.0912400	-0.7510500	2.2256320
P	-0.7896700	-2.2896300	2.6685720	P	-0.1240380	0.3364080	3.3560560
C	-1.8651870	-0.9640770	3.3155130	C	1.0931900	1.6851600	3.3339090
C	-1.5599720	-3.8376240	3.2146800	N	-0.2901420	2.2679870	0.2812240
C	0.7180290	-2.1255970	3.6773240	P	-0.9371970	3.6886710	-0.0557580
N	-0.1442130	2.2835310	-1.4582250	C	-0.7582990	4.7289360	1.4096800
P	-1.2179060	2.0745410	-2.6178310	C	-0.1462300	4.5644240	-1.4338260
C	-0.7812530	0.8233000	-3.8555340	C	-2.7021410	3.6323970	-0.4573230
N	0.1281430	1.7510940	1.2081280	N	-0.8853870	-2.0598130	-0.1863330
P	0.3732150	3.2251660	1.7668150	P	-0.9986500	-3.5911260	0.1799550
C	2.0230190	3.9233470	1.4486460	C	-2.5110540	-4.2622970	-0.5565890
C	-2.8841760	1.6318750	-2.0514720	C	0.3404060	-4.6470410	-0.4629460
C	-1.3845800	3.6413100	-3.5064500	C	-1.0796020	-3.9657400	1.9537090
C	-0.7839650	4.4755640	1.1393660	C	-1.7266900	1.1950440	3.4333640
C	0.1805100	3.1896640	3.5635660	C	0.0575280	-0.4746290	4.9651150
C	-3.7356920	-2.4125700	-0.3856080	C	-0.9257020	-1.4202670	-3.5438850
C	-3.0696530	-2.3093790	-3.2031710	C	-3.3067950	-0.7255260	-2.0950890
C	-3.5037220	1.6252100	1.8523470	O	-3.4360780	-1.4450680	1.6340770
O	-3.1064050	2.3556640	2.6684710	C	-3.3926890	-0.3526810	1.1855490
O	-3.9201950	0.8972550	1.0454880	O	-3.7992980	0.7032660	0.8391830
H	1.3725450	-1.5429200	-2.7103080	H	1.5989150	-2.3396730	-1.7303380
H	2.5587360	2.0612660	-0.7009040	H	1.7513160	1.9575900	-1.8986850
H	1.1882570	-2.6564860	-0.5062750	H	1.7676800	-2.1625150	0.7591570
H	4.6892590	1.3323800	-0.4408750	H	4.0277690	1.9655110	-1.7230580
H	3.0508650	0.6683950	1.3481860	H	2.7260780	1.9421580	0.4307200
H	4.6182110	0.0631950	-2.5957870	H	4.2175080	-0.1898220	-2.9732890
H	3.8259840	-2.3380020	-2.4844780	H	4.1781690	-2.3157670	-1.6010520
H	3.2719340	-3.2194810	-0.2070330	H	3.9779230	-2.0376900	0.8800510
H	2.3637740	-1.5900950	1.4645030	H	2.7103480	-0.0465800	1.7149680
H	2.1625530	0.8339290	-2.8439000	H	1.6495800	-0.2372070	-3.1010220
H	5.6796890	-2.6794520	-0.3237530	H	6.1307020	-1.0934800	0.1137450
H	5.0409270	-2.4258350	1.2917060	H	5.4988670	-0.2067480	1.4910870
H	6.3777690	-0.4607950	-0.3800090	H	6.1557770	0.8714400	-1.1312260
H	5.6974170	-0.1958350	1.2166450	H	5.5066570	1.7536740	0.2406860
H	2.7876250	3.2126700	1.7707060	H	0.9199660	4.6778810	-1.2258550
H	2.1488610	4.8637070	1.9910420	H	-0.5981440	5.5487830	-1.5767750
H	2.1367050	4.1124960	0.3786340	H	-0.2748670	3.9710900	-2.3426340
H	-1.8019940	4.1930990	1.4132190	H	-3.2337810	3.1138510	0.3425060
H	-0.6982260	4.4983020	0.0506970	H	-2.8245450	3.0645560	-1.3819690
H	-0.5530490	5.4585640	1.5567930	H	-3.0989880	4.6425130	-0.5826020
H	0.3199990	4.1880280	3.9841950	H	-1.1958550	5.7141390	1.2353550
H	0.9198840	2.5077780	3.9889820	H	0.3025070	4.8360040	1.6454320
H	-0.8215790	2.8285810	3.8023610	H	-1.2639210	4.2498100	2.2509010
H	-1.5552320	0.7565400	-4.6247990	H	-1.5947840	-2.0728490	-4.1107350
H	-0.6954870	-0.1338900	-3.3310890	H	-0.5174300	-1.9658180	-2.6894810
H	0.1719090	1.0876360	-4.3191440	H	-0.1198290	-1.0777370	-4.1952270
H	-3.5690820	1.5306810	-2.8975880	H	-3.8798060	-1.3176870	-2.8130180
H	-2.8104120	0.6800990	-1.5194690	H	-2.9419900	-1.3661840	-1.2873880
H	-3.2542110	2.4035640	-1.3732440	H	-3.9316080	0.0711610	-1.6882370
H	-1.7156960	4.4168330	-2.8124900	H	-3.1029210	1.7273900	-4.0023150
H	-0.4158230	3.9241960	-3.9232560	H	-1.6170550	1.3161290	-4.8913660
H	-2.1121970	3.5428770	-4.3152980	H	-3.0393200	0.2447480	-4.9902320
H	-2.5202650	-4.9070400	-1.6048060	H	0.1622360	-5.6952680	-0.2097440
H	-1.2869760	-4.3096940	-0.4475600	H	1.2948040	-4.3300410	-0.0361560
H	-0.9382100	-4.3244480	-2.1941570	H	0.3884360	-4.5423980	-1.5496040
H	-4.1899560	-1.4200560	-0.4195770	H	-1.9388110	-3.4451580	2.3801320
H	-3.3500490	-2.5907530	0.6200510	H	-0.1728110	-3.5963470	2.4372970
H	-4.4843760	-3.1697510	-0.6305970	H	-1.1758530	-5.0425880	2.1114650
H	-3.8251090	-3.0800910	-3.3719470	H	-2.5973930	-5.3336520	-0.3614930
H	-2.2808570	-2.4021170	-3.9526840	H	-2.4904140	-4.0896390	-1.6354340
H	-3.5295200	-1.3227000	-3.2924440	H	-3.3674080	-3.7382280	-0.1268390
H	-1.9917480	-1.0837930	4.3942530	H	-1.7097200	1.9299260	4.2423970
H	-2.8417310	-1.0150100	2.8283320	H	-2.5238450	0.4733140	3.6240550
H	-1.4069850	0.0066780	3.1082210	H	-1.9059880	1.6961340	2.4806700
H	-0.9213490	-4.6766590	2.9305330	H	1.0432620	-0.9403740	5.0249650
H	-1.6987370	-3.8382820	4.2984890	H	-0.0492530	0.2534530	5.7726280
H	-2.5307360	-3.9495410	2.7263190	H	-0.7090000	-1.2457280	5.0679570
H	1.4267330	-2.9098570	3.4017630	H	2.0979960	1.2770370	3.4618520
H	1.1716220	-1.1504360	3.4793210	H	1.0168850	2.1840480	2.3636140
H	0.4864910	-2.2027650	4.7428130	H	0.8886250	2.3997470	4.1354170
H	-1.3467790	-0.2589660	0.7771470	H	-1.6389130	-0.1944630	0.8181670
**III-Pa$_2$ TS1**				**III-Pa$_2$ TS2**			

C	5.6893820	0.9513950	0.0198880
C	4.7768370	0.9239720	1.2559870
C	3.6679520	1.9130670	0.8563370
C	3.6483330	1.9751110	-0.7042340
C	4.7682120	1.0442980	-1.2044600
C	2.2703370	1.4461690	-1.1622560
C	1.4267650	1.7211700	0.0935520
C	2.2970660	1.3689720	1.2979320
C	4.0551340	-0.3728170	1.7505910
C	2.5040020	-0.1053510	1.7439650
C	4.0712470	-0.2165560	-1.8019960
C	2.5158370	0.0331770	-1.7871820
C	4.2995090	-1.7900410	1.1521890
C	2.9347080	-2.3016530	0.6592000
C	1.7952520	-1.3582020	1.1226810
C	2.9694070	-2.2487970	-0.9033200
C	1.8378020	-1.2989540	-1.3409930
C	0.8993030	-1.4328160	-0.1375830
C	4.3361900	-1.6764710	-1.3084410
C	5.1952600	-2.0016470	-0.0763400
P	-0.3513120	1.3236630	0.1964300
P	-0.9367400	-0.9873020	-0.2160020
N	-1.4895630	-1.4527590	1.2694090
P	-1.9395880	-2.9219770	1.6781040
C	-2.3085940	-2.8944180	3.4495020
N	-1.2240100	-2.2147530	-1.3744920
P	-1.8577710	-1.9422910	-2.8029440
C	-1.8946070	-3.4859650	-3.7470380
N	-1.0078540	1.9042010	-1.2036180
P	-1.4150120	3.3941940	-1.5824210
C	-0.0889150	4.6361380	-1.4659530
N	-0.8353010	2.2388620	1.4928380
P	-1.5101460	1.7626940	2.8527750
C	-0.5640150	0.5346810	3.7950480
C	-2.7760940	4.0934270	-0.6055050
C	-1.9662800	3.4032930	-3.3051790
C	-3.5742340	-1.3191000	-2.8425930
C	-0.9557900	-0.7284210	-3.8183940
C	-0.7068790	-4.2392540	1.4392110
C	-3.4307250	-3.5573540	0.8582360
C	-1.6821310	3.2087770	3.9270570
C	-3.1778690	1.0672710	2.6813450
C	-5.0405660	0.2926940	-0.1037410
O	-4.8184860	1.2620880	-0.7097570
O	-5.2699340	-0.6752840	0.5004060
H	1.3213520	-1.7127290	-2.2102020
H	1.2299820	-1.8208370	1.9365110
H	4.7422400	-2.2301140	-2.1605160
H	4.6722590	-2.4231700	1.9631560
H	0.6870070	-2.5087860	-0.1685120
H	6.1747670	-1.5339990	-0.0337950
H	5.3901460	-3.0812420	-0.1270670
H	6.4620440	0.1892910	-0.0251720
H	6.2264680	1.9080980	0.0679740
H	1.3797450	2.8207240	0.1276280
H	5.3450100	1.3138430	2.1059860
H	1.9218760	1.9256480	2.1615740
H	3.8490530	2.9089130	1.2707400
H	3.8016660	3.0045580	-1.0409010
H	5.3238470	1.5210290	-2.0177030
H	1.8701090	2.0730010	-1.9638720
H	2.8144110	-3.2505920	-1.3146510
H	2.7462690	-3.3263850	0.9938360
H	2.1832730	-0.1234280	2.7885290
H	4.3497480	-0.4604980	2.7996600
H	4.3739330	-0.2278890	-2.8523830
H	2.2081560	0.1540790	-2.8296770
H	-0.8911330	0.2064990	-3.2494660
H	0.0538910	-1.0936490	-4.0204690
H	-1.4712980	-0.5463380	-4.7649110
H	-2.5136180	-4.2148060	-3.2190390
H	-0.8789150	-3.8769130	-3.8338750
H	-2.3066200	-3.3152610	-4.7443920
H	-4.2030610	-1.9407200	-2.2004400
H	-3.9670730	-1.3369860	-3.8623890
H	-3.5890760	-0.2889830	-2.4788870
H	-3.7053730	-4.5337970	1.2650910
H	-4.2513640	-2.8524500	1.0014120
H	-3.2143890	-3.6450540	-0.2084800
H	-1.4040960	-2.6285120	4.0006130
H	-3.0757400	-2.1411810	3.6420910
H	-2.6647330	-3.8711490	3.7841070
H	-0.5389250	-4.3625060	0.3667650
H	-1.0684220	-5.1800200	1.8622970
H	0.2312580	-3.9588970	1.9242070
H	-3.0829590	0.1441340	2.1017920
H	-3.6147090	0.8469390	3.6588020

C	5.4423200	-1.2688500	-0.6179380
C	4.5543240	-0.5673400	-1.6559370
C	3.3288940	-1.4976500	-1.7168910
C	3.2927520	-2.2958350	-0.3754550
C	4.5009980	-1.8289720	0.4576810
C	1.9801510	-1.9178720	0.3429270
C	1.1223680	-1.4572070	-0.8442210
C	2.0356450	-0.6580490	-1.7809390
C	4.0056670	0.8804600	-1.4466110
C	2.4359200	0.8202980	-1.5129250
C	3.9343970	-0.9382570	1.6082150
C	2.3641350	-0.9907490	1.5347690
C	4.3978650	1.8067470	-0.2537940
C	3.0866570	2.1761630	0.4629740
C	1.8657750	1.7073870	-0.3595270
C	3.0614730	1.3789780	1.8042510
C	1.8258290	0.4568430	1.7544440
C	0.9242550	1.2216460	0.7652660
C	4.3535920	0.5454200	1.8595820
C	5.2777300	1.3146840	0.9041590
P	-0.6290200	-1.0133240	-0.6245300
N	-1.2664710	-0.9788930	-2.1113400
P	-1.6801010	0.0741200	-3.2279190
C	-0.4392880	1.3407080	-3.6040520
P	-0.9381830	0.8468380	0.5506370
N	-1.2585820	2.1466790	-0.4238540
P	-1.4421000	3.6920940	-0.1664240
C	-1.4204330	4.5369580	-1.7690140
N	-1.2160780	0.8962320	2.1848750
P	-1.7664230	-0.1437940	3.2425480
C	-2.0191520	0.7249400	4.8115440
N	-1.2763810	-2.1547060	0.3304060
P	-1.7967600	-3.6148190	-0.0459770
C	-0.6226410	-4.6345640	-0.9870080
C	-3.3294270	-3.6482790	-1.0094750
C	-2.1190020	-4.4990640	1.4964360
C	-3.3523010	-0.9657250	2.8968620
C	-0.6303430	-1.5133500	3.6114680
C	-0.1811330	4.5369700	0.8450480
C	-3.0048510	4.1771890	0.6230520
C	-1.9607380	-0.8415660	-4.7626480
C	-3.2091860	0.9891110	-2.9087520
O	-4.4518510	1.4068890	0.7114860
C	-4.2066780	0.3776370	0.1849370
O	-4.4234460	-0.6473600	-0.3630500
H	1.3179820	0.4653080	2.7208520
H	1.3862140	2.5623170	-0.8433760
H	4.7897720	0.5862570	2.8623930
H	4.8627480	2.6999270	-0.6823580
H	0.7716340	2.1529420	1.3280280
H	6.1999390	0.8141050	0.6223330
H	5.5901820	2.2153130	1.4493770
H	6.2925280	-0.6986690	-0.2549060
H	5.8716680	-2.1409830	-1.1287280
H	0.9606750	-2.3891240	-1.4096900
H	5.0808970	-0.5724720	-2.6150010
H	1.6043910	-0.7076640	-2.7848710
H	3.3900030	-2.1873480	-2.5633530
H	3.3333210	-3.3703130	-0.5761430
H	4.9902390	-2.6869890	0.9284540
H	1.5052760	-2.8066450	0.7682430
H	2.9957700	2.0703640	2.6497220
H	3.0324470	3.2509020	0.6646080
H	2.1481700	1.3488380	-2.4245020
H	4.3335870	1.4253340	-2.3357240
H	4.2213300	-1.4612330	2.5242770
H	2.0306370	-1.5431050	2.4168000
H	-0.4265340	-2.0370020	2.6735720
H	0.3001450	-1.1162680	4.0228970
H	-1.0791940	-2.2027260	4.3311760
H	-2.7589170	1.5160400	4.6717010
H	-1.0755010	1.1698060	5.1335940
H	-2.3719840	0.0292490	5.5767230
H	-4.1478480	-0.2204650	2.8369090
H	-3.5827940	-1.6737890	3.6970050
H	-3.2679610	-1.4941530	1.9456040
H	-3.0618210	5.2648400	0.7151570
H	-3.8407250	3.8071670	0.0280540
H	-3.0598480	3.7206600	1.6134710
H	-0.4450000	4.3957400	-2.2397320
H	-2.1912620	4.1046310	-2.4109150
H	-1.6106930	5.6051450	-1.6430970
H	-0.2030830	4.1410980	1.8632510
H	-0.3926510	5.6089530	0.8766950
H	0.8120060	4.3781290	0.4194100
H	-3.0468160	1.6006310	-2.0174310
H	-3.4443180	1.6266210	-3.7649330

H	-3.8076250	1.7894330	2.1554530	H	-4.0244670	0.2852200	-2.7359340
H	-1.0888920	0.2608270	4.7139590	H	-0.8333260	2.0438570	-4.3421670
H	0.4164730	0.9481940	4.0429080	H	0.4637360	0.8707090	-3.9984540
H	-0.4514850	-0.3466810	3.1565010	H	-0.2154430	1.8698560	-2.6740910
H	-2.1414920	2.9228000	4.8761310	H	-2.2508010	-0.1576390	-5.5635560
H	-2.3079170	3.9544800	3.4318760	H	-2.7563410	-1.5725900	-4.6033180
H	-0.6959730	3.6380490	4.1155180	H	-1.0442820	-1.3644750	-5.0434580
H	-2.2956600	4.4021610	-3.6001650	H	-2.5000610	-5.5011510	1.2885580
H	-1.1431480	3.0850180	-3.9484660	H	-1.1913470	-4.5720350	2.0678760
H	-2.7945780	2.6995660	-3.4128620	H	-2.8555420	-3.9449840	2.0818910
H	-0.4251260	5.5968490	-1.8638970	H	-1.0094530	-5.6500360	-1.1018860
H	0.1883550	4.7632370	-0.4165720	H	-0.4789980	-4.1951070	-1.9773030
H	0.7831760	4.2950790	-2.0287930	H	0.3360210	-4.6696600	-0.4639630
H	-3.0293600	5.0949880	-0.9622290	H	-3.6474150	-4.6785470	-1.1857360
H	-2.4570840	4.1337860	0.4380570	H	-3.1447920	-3.1435270	-1.9603700
H	-3.6470200	3.4406700	-0.6855710	H	-4.1020500	-3.0990150	-0.4701510
H	-2.0076860	-0.1811140	-0.7361820	H	-2.4197230	0.3567730	0.3128240
**I-Pa$_2$ min2**				**II-Pa$_2$ min2**			
C	0.2147030	-0.4152080	2.9023140	C	1.4158780	2.5685230	-2.7115400
C	-0.9228340	0.6067730	2.5578340	C	0.5112020	3.5709620	-1.9745760
C	-1.5631480	0.3799230	1.1834590	C	1.2321330	4.8484710	-1.5578300
C	-2.0795530	-1.0610190	1.3731980	C	2.4491050	4.4986290	-0.6767250
C	-0.9418840	-2.1005700	1.7224240	C	2.5427120	2.9868650	-0.4911180
C	0.4425820	-1.4396550	1.7859890	C	2.6392790	2.2117050	-1.8164000
C	-1.8713320	-0.1247340	3.5568590	C	1.2807190	2.3211420	0.1131140
C	-2.8587160	-0.9710970	2.7137910	C	0.0382330	2.6880680	-0.7929060
C	-1.2271160	-2.4413880	3.2083760	C	0.7970270	1.1625620	-2.4288350
C	-0.7427430	-1.1414730	3.8965590	C	-0.4554520	1.4647100	-1.5957680
C	-2.7503910	-2.3696820	3.3227000	C	2.0203280	0.8025650	-1.5227560
P	-0.5436320	0.8513960	-0.3723060	C	1.6906570	0.8340970	-0.0211320
P	0.9745500	-0.7253450	0.2033450	P	0.5990140	-0.5314640	0.7730160
N	1.1832460	-1.9505530	-0.8519920	N	1.5148270	-1.8180110	0.2229190
P	0.4383190	-2.4688420	-2.1613140	P	2.9485060	-2.3519620	0.5999850
C	1.3383700	-3.9218230	-2.7551630	C	3.3164560	-2.3348080	2.3809460
N	2.3520140	0.1140520	0.4519530	P	-1.0620310	0.0582800	-0.6237940
P	3.8339370	-0.3104930	0.0420350	N	-2.2561110	0.5853460	0.3553940
C	4.9681840	0.9298990	0.7045210	P	-3.8251550	0.3610080	0.1720720
C	4.3723720	-1.9198890	0.6840990	C	-4.3696310	-1.3615890	0.3457770
C	4.1340410	-0.3800110	-1.7466490	N	-1.5832080	-1.0815820	-1.6657070
N	-0.4396110	2.4375600	0.1374290	P	-1.0596130	-2.5119850	-2.1349830
P	0.7902970	3.4374610	0.1283260	C	-0.9642290	-3.7628940	-0.8269020
C	1.9774630	3.3379930	-1.2500270	C	-2.2333990	-3.1386530	-3.3605130
N	-1.6773750	0.3526110	-1.4974570	C	0.5667710	-2.5090310	-2.9347170
P	-3.1428870	0.8386650	-1.8243410	N	0.8130330	0.1381800	2.2887230
C	-4.5155880	0.1658960	-0.8251900	P	-0.2413990	0.3935660	3.4446340
C	0.1709380	5.1406750	0.1022760	C	0.6273370	0.4517230	5.0343430
C	1.8221730	3.3062660	1.6169930	C	-1.0989590	1.9865890	3.2831800
C	-3.3791760	2.6399760	-1.7523860	C	-1.5860120	-0.8148070	3.6729050
C	-3.5539980	0.3327690	-3.5158780	C	-4.6715820	1.3015220	1.4618310
C	-1.2770780	-2.9983320	-1.9104890	C	-4.4932620	0.9157740	-1.4212800
C	0.3871310	-1.2923280	-3.5391230	C	3.0855290	-4.0768440	0.0591940
H	-3.1065070	-2.4195740	4.3572280	C	4.3907860	-1.5377460	-0.1688240
H	-3.2628380	-3.1380400	2.7339860	H	2.5864090	-0.0717210	-1.8463280
H	1.1305860	0.0205210	3.3038400	H	-1.2844880	1.7679990	-2.2489650
H	-2.4427440	1.0399770	1.1422880	H	2.6369890	0.7354810	0.5277460
H	1.1973350	-2.1777480	2.0888730	H	-0.3547160	3.8238990	-2.5975700
H	-3.8621380	-0.5496270	2.6147170	H	-0.7561890	3.1550200	-0.2050690
H	-2.6921210	-1.4211320	0.5448300	H	1.6294680	2.8322110	-3.7496340
H	-2.3185190	0.4451880	4.3731850	H	3.6197540	2.2532720	-2.2968130
H	-0.4320600	-1.2483930	4.9369940	H	3.4021530	2.7521810	0.1468930
H	-0.7423950	-3.3571710	3.5544560	H	1.1026250	2.6134480	1.1502510
H	-0.9723560	-2.9739280	1.0673650	H	0.6220030	0.5043800	-3.2812160
H	-0.6776940	1.6489600	2.7635110	H	3.3772340	4.8654440	-1.1290850
H	-1.7034230	-3.3595080	-2.8499180	H	2.3707780	4.9781690	0.3051100
H	-1.3116070	-3.7961660	-1.1655360	H	1.5409010	5.3969240	-2.4544500
H	-1.8407530	-2.1293760	-1.5612700	H	0.5327900	5.4984400	-1.0207860
H	1.3445540	-4.6844940	-1.9738650	H	-4.2612530	1.9738900	-1.5608940
H	0.8596720	-4.3233360	-3.6511460	H	-5.5752010	0.7693890	-1.4621040
H	2.3679770	-3.6428670	-2.9891450	H	-4.0135850	0.3340960	-2.2122620
H	-0.0273280	-1.7725030	-4.4296200	H	-4.0204970	-1.7591860	1.3011960
H	-0.2462050	-0.4537040	-3.2340870	H	-3.9299260	-1.9430520	-0.4667390
H	1.3985450	-0.9395440	-3.7529400	H	-5.4594020	-1.4271140	0.2999160
H	4.2766560	-1.9222910	1.7721460	H	-5.7530090	1.1715430	1.3819330
H	3.7217080	-2.6904930	0.2637270	H	-4.4216330	2.3591500	1.3559860
H	5.4098550	-2.1221550	0.4075920	H	-4.3339890	0.9523040	2.4402450
H	3.5059820	-1.1666460	-2.1681340	H	0.8420270	-3.5228630	-3.2367220
H	3.8622400	0.5757970	-2.2000630	H	1.2902080	-2.1308120	-2.2073090
H	5.1848810	-0.5964230	-1.9531940	H	0.5414850	-1.8622110	-3.8142380
H	4.8706250	0.9606950	1.7915660	H	-0.7108310	-4.7373720	-1.2531070
H	5.9984180	0.6861360	0.4364920	H	-0.1877550	-3.4458050	-0.1242370
H	4.7080780	1.9088040	0.2959350	H	-1.9283310	-3.8265670	-0.3172110
H	-0.3822920	5.3101840	-0.8241660	H	-3.2224510	-3.2203180	-2.9045010
H	-0.4995090	5.2925340	0.9507180	H	-2.2835990	-2.4429780	-4.2005520
H	1.0002930	5.8492840	0.1651300	H	-1.9174380	-4.1206500	-3.7196910
H	2.4618330	2.3611560	-1.2202860	H	5.3150350	-2.0262020	0.1509300
H	1.4558510	3.4473270	-2.2030740	H	4.4255070	-0.4837950	0.1141930

H	2.7264920	4.1275790	-1.1479180	H	4.3060620	-1.6086010	-1.2558350
H	1.2019270	3.4778810	2.4998640	H	2.5660760	-2.9305310	2.9055520
H	2.2245200	2.2894140	1.6459090	H	3.2524980	-1.3048720	2.7399490
H	2.6385220	4.0330770	1.5969550	H	4.3117120	-2.7391370	2.5800780
H	-2.6804690	3.1201070	-2.4408890	H	4.0772340	-4.4771160	0.2824490
H	-4.4037000	2.9080180	-2.0212720	H	2.9107080	-4.1248310	-1.0183800
H	-3.1578820	2.9844950	-0.7397870	H	2.3259760	-4.6737740	0.5681080
H	-2.8458870	0.7920810	-4.2084300	H	-2.1962110	-0.5283170	4.5335270
H	-3.4687900	-0.7539980	-3.5908790	H	-1.1710760	-1.8121390	3.8326310
H	-4.5711870	0.6348280	-3.7762250	H	-2.1993610	-0.8253370	2.7707530
H	-4.5148350	-0.9244670	-0.8949610	H	1.4106250	1.2111410	4.9875450
H	-4.4000630	0.4537630	0.2217290	H	-0.0655840	0.6961920	5.8429630
H	-5.4700060	0.5465610	-1.1983980	H	1.0850880	-0.5205570	5.2298970
H	0.4336710	1.0531360	-1.4684490	H	-0.3609510	2.7918930	3.2615870
				H	-1.6455200	1.9666050	2.3355680
				H	-1.7921430	2.1487120	4.1128870
				H	-0.4355670	-1.4803150	1.2557230

III-Pa2 min2

C	5.1170810	-1.7082090	-0.5640200
C	4.2326020	-1.8797450	0.6797360
C	2.9013850	-2.3555100	0.0721910
C	2.8902970	-1.8935260	-1.4197050
C	4.2123540	-1.1485900	-1.6719530
C	1.7063570	-1.6630510	0.7643470
C	2.3192920	-0.5472480	1.6740580
C	3.8862650	-0.6870190	1.6249800
C	1.9869020	0.9729850	1.5907880
C	3.3148210	1.7153670	1.3318730
C	4.4983120	0.7437480	1.4895990
C	3.2982490	2.1778490	-0.1599040
C	4.4737740	1.4751250	-0.8627420
C	5.4028480	1.1427420	0.3141460
C	1.1003390	1.5775890	0.4956040
C	1.9617440	1.7028470	-0.7673440
C	2.2858080	0.5008140	-1.7068260
C	3.8531480	0.3678470	-1.7729870
C	1.6888630	-0.9392130	-1.5820430
C	0.7732890	-1.4262910	-0.4417770
P	-0.6558580	1.1352740	0.3843160
N	-1.2840340	2.0503330	-0.8138270
P	-1.9938390	3.4713040	-0.6747950
C	-2.2727190	4.1170700	-2.3392380
P	-1.0784910	-0.9509810	-0.3133770
N	-1.3314280	-1.4044670	-1.9071530
P	-2.0891950	-0.6517160	-3.0754780
C	-1.0806560	0.6227470	-3.8874550
N	-1.2885780	1.5203460	1.8354660
P	-1.8573460	0.7440810	3.1012920
C	-3.4688380	-0.0388570	2.8250560
C	-0.7741720	-0.5380250	3.7825010
C	-2.1084700	1.9557900	4.4214520
N	-1.3587160	-2.0349230	0.9364920
P	-2.2444240	-3.3351430	0.7870340
C	-3.9104050	-3.1147310	0.0792900
C	-2.4901120	-4.0677500	2.4241560
C	-1.4978590	-4.6325800	-0.2469030
C	-2.5078350	-1.8501770	-4.3691800
C	-3.6557400	0.1890260	-2.6761560
C	-1.0373790	4.7240360	0.2262400
C	-3.6129430	3.4222270	0.1431010
H	1.1648290	-1.1446660	-2.5180590
H	1.1957210	-2.3620490	1.4303130
H	4.6363010	-1.4402360	-2.6380300
H	4.6698220	-2.6651790	1.3042370
H	0.5790250	-2.4623040	-0.7597540
H	6.0625230	-1.1947930	-0.4115490
H	5.3864560	-2.7242140	-0.8826750
H	6.2262000	0.4688590	0.0956840
H	5.8720800	2.0922560	0.6047660
H	0.9771580	2.6233280	0.8232790
H	5.0364480	0.9504360	2.4197500
H	1.5685990	1.2703810	2.5572010
H	3.4149370	2.5811580	1.9927040
H	3.3889690	3.2664760	-0.2163950
H	4.9947860	2.1763780	-1.5217840
H	1.5215990	2.4880810	-1.3878820
H	2.7891880	-2.7607550	-2.0795400
H	2.8037970	-3.4441940	0.1281630
H	2.0149760	-0.8316030	2.6849040
H	4.2000050	-1.0235070	2.6168350
H	4.1481120	0.6517830	-2.7868070
H	1.9578060	0.8421020	-2.6929230
H	-0.8327330	1.3734200	-3.1315740
H	-0.1620360	0.1646310	-4.2621270
H	-1.6220520	1.0882260	-4.7153550
H	-3.1915630	-2.5985690	-3.9622130

H	-1.5963090	-2.3468430	-4.7082230
H	-2.9822430	-1.3476680	-5.2155690
H	-4.3642170	-0.5247630	-2.2498650
H	-4.0825620	0.6334920	-3.5790180
H	-3.4462200	0.9651200	-1.9390740
H	-4.4440300	-4.0679950	0.0524730
H	-4.4781860	-2.3969480	0.6755730
H	-3.8054870	-2.7264690	-0.9364690
H	-1.5166730	-4.2936940	2.8645230
H	-3.0116030	-3.3518110	3.0633320
H	-3.0784350	-4.9852440	2.3507720
H	-1.3729250	-4.2284320	-1.2549660
H	-2.1258640	-5.5263540	-0.2819130
H	-0.5153600	-4.8891390	0.1565500
H	-3.3404390	-0.7693980	2.0246550
H	-3.8227470	-0.5321640	3.7335910
H	-4.1909110	0.7201200	2.5156540
H	-1.2362910	-0.9955710	4.6611680
H	0.1791400	-0.0868310	4.0676130
H	-0.6211470	-1.2921260	3.0038080
H	-2.5319330	1.4710200	5.3041810
H	-2.7898770	2.7349900	4.0734210
H	-1.1493420	2.4090100	4.6802170
H	-2.7775670	5.0845340	-2.2952100
H	-1.3114920	4.2285850	-2.8450680
H	-2.8892390	3.4102460	-2.8991240
H	-1.5629190	5.6818300	0.2336210
H	-0.8959540	4.3777760	1.2531320
H	-0.0622350	4.8462750	-0.2512070
H	-4.0708690	4.4141360	0.1557850
H	-3.4650350	3.0675470	1.1648890
H	-4.2664920	2.7254250	-0.3863630
H	-2.4685140	-0.4091510	-0.2011100
